# Plant mitochondrial RNA editing factors can perform targeted C-to-U editing of nuclear transcripts in human cells

**DOI:** 10.1093/nar/gkac752

**Published:** 2022-09-15

**Authors:** Elena Lesch, Maximilian T Schilling, Sarah Brenner, Yingying Yang, Oliver J Gruss, Volker Knoop, Mareike Schallenberg-Rüdinger

**Affiliations:** IZMB - Institut für Zelluläre und Molekulare Botanik, Abteilung Molekulare Evolution, Universität Bonn, Kirschallee 1, D-53115 Bonn, Germany; Institut für Genetik, Abteilung Zellteilung, Universität Bonn, Karlrobert-Kreiten-Str. 13, D-53115 Bonn, Germany; IZMB - Institut für Zelluläre und Molekulare Botanik, Abteilung Molekulare Evolution, Universität Bonn, Kirschallee 1, D-53115 Bonn, Germany; IZMB - Institut für Zelluläre und Molekulare Botanik, Abteilung Molekulare Evolution, Universität Bonn, Kirschallee 1, D-53115 Bonn, Germany; Institut für Genetik, Abteilung Zellteilung, Universität Bonn, Karlrobert-Kreiten-Str. 13, D-53115 Bonn, Germany; IZMB - Institut für Zelluläre und Molekulare Botanik, Abteilung Molekulare Evolution, Universität Bonn, Kirschallee 1, D-53115 Bonn, Germany; IZMB - Institut für Zelluläre und Molekulare Botanik, Abteilung Molekulare Evolution, Universität Bonn, Kirschallee 1, D-53115 Bonn, Germany

## Abstract

RNA editing processes are strikingly different in animals and plants. Up to thousands of specific cytidines are converted into uridines in plant chloroplasts and mitochondria whereas up to millions of adenosines are converted into inosines in animal nucleo-cytosolic RNAs. It is unknown whether these two different RNA editing machineries are mutually incompatible. RNA-binding pentatricopeptide repeat (PPR) proteins are the key factors of plant organelle cytidine-to-uridine RNA editing. The complete absence of PPR mediated editing of cytosolic RNAs might be due to a yet unknown barrier that prevents its activity in the cytosol. Here, we transferred two plant mitochondrial PPR-type editing factors into human cell lines to explore whether they could operate in the nucleo-cytosolic environment. PPR56 and PPR65 not only faithfully edited their native, co-transcribed targets but also different sets of off-targets in the human background transcriptome. More than 900 of such off-targets with editing efficiencies up to 91%, largely explained by known PPR-RNA binding properties, were identified for PPR56. Engineering two crucial amino acid positions in its PPR array led to predictable shifts in target recognition. We conclude that plant PPR editing factors can operate in the entirely different genetic environment of the human nucleo-cytosol and can be intentionally re-engineered towards new targets.

## Graphical Abstract

**Graphical Abstract:**
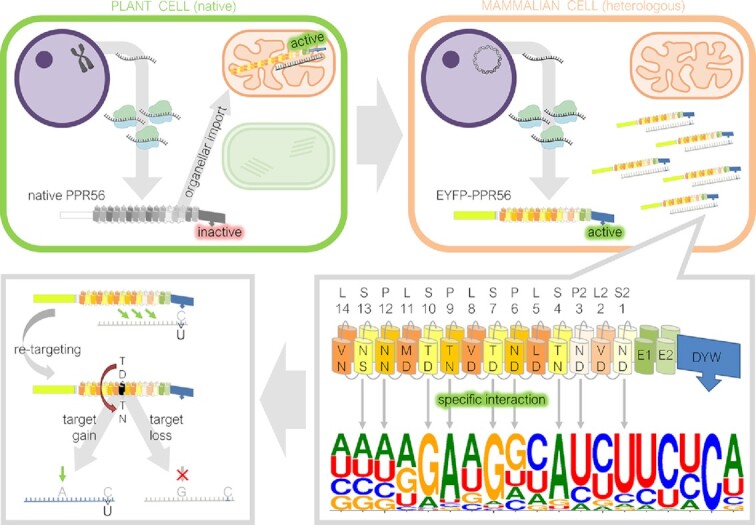
Plant mitochondrial PPR-DYW deaminase specifically edits cytidines in nuclear-cytosolic transcripts in human cells. The editing enzyme can be re-directed to new targets via modification of single binding motifs.

## INTRODUCTION

The term RNA editing had originally been coined for the seminal discovery of post-transcriptional uridine insertions into mitochondrial pre-mRNAs of trypanosomes, reconstituting proper reading frames (1). Numerous diverse systems of correcting primary transcripts by inserting, deleting or substituting nucleotides, not only in coding regions but also in introns, UTRs, tRNAs and rRNAs have since been discovered in many different groups of organisms (2–5). Particular abundant, but entirely different types of RNA editing are present in the animal and in the plant world.

After the original discovery of cytidine-to-uridine (C-to-U) RNA editing in plant mitochondria more than three decades ago (6–8) it quickly became evident that C-to-U editing affects transcripts not only in mitochondria but also in chloroplasts of (nearly) all land plants, mostly to reconstitute evolutionarily conserved codons and thereby amino acid identities (9,10). Despite only comparatively small transcriptomes in the two endosymbiotic organelles of plants, thousands of sites may be affected in chloroplasts or mitochondria in some plant lineages (11–13).

At around the same time when plant organelle C-to-U editing was discovered, two different types of RNA editing were also reported in the animal kingdom. The discovery of a cytidine-to-uridine exchange introducing an early stop codon in the apolipoprotein B mRNA in the intestine even antedated the reports on plant organellar RNA editing (14,15). It is now understood that the eponymous A1 ‘activation-induced deaminase’ (AID) is only one member of the APOBEC (Apolipoprotein B mRNA editing catalytic polypeptide-like) family with altogether 11 similar enzymes variably acting on (native or foreign/infectious) RNAs or DNAs (16). Much more prominent than the C-to-U-type of RNA editing in animals, however, is adenosine-to-inosine (A-to-I) RNA editing in metazoa, which was originally reported for codon sense changes in glutamate-gated ion channels (17). Since then, it became evident that the A-to-I type of RNA editing is extremely frequent in animals, possibly even affecting millions of sites to variable degrees in thousands of RNAs in humans (18–20). Key players for A-to-I editing in the animal editing world are ADARs, the ‘Adenosine Deaminases Acting on RNAs’ targeting adenosines in specific RNA secondary structures (21).

There is no doubt that the diverse types of RNA editing in the living world had entirely different evolutionary trajectories and considerable progress has been made to elucidate their biochemical mechanisms (2,5). In the case of plant organelle C-to-U RNA editing, the RNA-binding pentatricopeptide repeat (PPR) proteins encoded by large and diversified gene families in plants are at the core of the RNA editing machinery (22,23). Individual PPRs can recognize individual ribonucleotides on a 1:1 basis following a PPR-RNA binding code (24–26). When equipped with a carboxyterminal ‘DYW domain’, which is named after its conserved terminal aspartate-tyrosine-tryptophane tripeptide motif and meantime clearly characterized as cytidine deaminase (27–29), plant PPR-type editing factors may combine recognition of RNA sequences and the enzymatic activity for C-to-U conversion in a single protein. For instance, all RNA editing factors in the model moss *Physcomitrium patens* are of this type, while much more diverse and complex editosomes requiring assembly of multiple interacting proteins have evolved in flowering plants (30–32). Accordingly, *Physcomitrium* played a significant model role by representing a simple, and likely evolutionary early, state of plant organellar RNA editing with its only 13 organelle RNA editing sites assigned to its only nine DYW-type editing factors (33–36).

It is puzzling why the one versus the other biochemical machinery was established as the dominating RNA editing process modifying transcriptomes in the two large clades of multicellular eukaryotes, both of which are older than 500 million years. The only common denominator of A-to-I and C-to-U RNA editing is the biochemically quite simple deamination of the respective nucleobase, either converting adenosine into inosine in animal nuclear transcripts or converting cytidine into uridine in plant organelle RNAs. Notably, and despite 500 million years of evolution and PPR gene families extended to hundreds or even thousands of members in some plant species (13,37), there is no known example of a PPR protein acting as an RNA editing factor in the cytosol. Given that loss of organellar targeting signals at the N-terminus would be a small evolutionary step, it is surprising that plant C-to-U RNA editing has not expanded from the two endosymbiotic organelles into the nucleo-cytosolic environment. A biochemical necessity of unfolding translated PPR proteins and refolding them upon import through mitochondrial or chloroplast membranes by specific organelle chaperones, respectively, could have provided one possible explanation. Alternatively, yet unknown factors present in the eukaryotic cytosol may be incompatible with the functions of DYW-type cytidine deamination factors.

**Figure 1.**
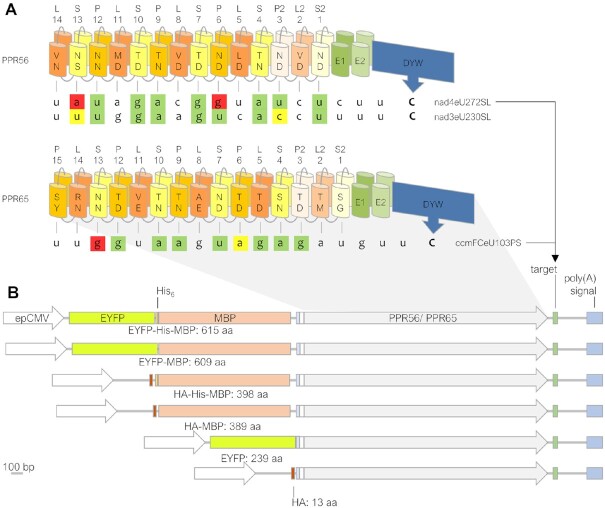
Design of constructs for expressing plant RNA editing factors in human cells. (**A**) PPR56 and PPR65 are typical pentatricopeptide repeat (PPR) proteins acting as organelle RNA editing factors, featuring a ‘PLS-type’ array of PPRs allowing them to recognize their native targets nad4eU272SL or ccmFCeU103PS, respectively, followed by E1 and E2 extensions and the carboxyterminal DYW cytidine deaminase domain. Positions 5 and L of the P-and S-type PPRs are key positions for RNA binding according to a core PPR-RNA code (24–26) for combinations 5 + L as follows: T/S + N: A, T/S + D: G, N + S: C, N + D: U, N + N: C/U. PPRs are labeled to indicate the respective PPR-type and positions 5 and L with backward numbering starting with S2-1 (here S2-1ND and S2-1SG for PPR56 and PPR65, respectively), as previously suggested (84). Editing sites are labeled with target gene name (*nad* genes encode for subunits of the NADH ubiquinone oxidoreductase and *ccmFC* encodes for subunit FC of the cytochrome *c* maturation machinery) followed by eU, coding sequence position, and resulting amino acid change. Nucleotide shading indicates matches to the corresponding PPR in green, transitions in yellow and mismatches in red. (**B**) PPR56 and PPR65 were cloned with different combinations of up to three out of four N-terminal tags (EYFP: Enhanced Yellow Fluorescent Protein, His_6_: 6 x Histidine tag, MBP: Maltose Binding Protein, HA: Hemagglutinin tag). Small grey and white rectangles indicate a TEV recognition site (Tobacco Etch Virus protease) and a short stretch of native editing factor sequence upstream of the first clearly defined PPR, respectively. Protein coding sequences were transcribed from the enhanced Cytomegalovirus promoter (epCMV) together with their respective 46 bp targets cloned downstream followed by the polyadenylation signal.

We here address the question whether plant organelle C-to-U RNA editing can be functionally transferred into the nucleo-cytosolic environment of animal cells. To that end we engineered two different plant mitochondrial DYW-type RNA editing factors of *P. patens* for expression in human cells. PPR56 and PPR65 have recently been shown to faithfully perform RNA editing of their cognate mitochondrial targets, nad4eU272SL and ccmFCeU103PS, in *Escherichia coli* as a heterologous bacterial expression system (26, for RNA editing site nomenclature see Figure 1). Depending on specific constructs and cell lines we were consistently able to observe efficient RNA editing of up to 72% C-to-U conversion at the cognate target sites of PPR56 and PPR65. Using extensive transcriptome analyses we found that PPR56 affects ca. 900 RNA off-targets with significant similarity to its native targets in the endogenous human transcriptome. Moreover, specific single amino acid exchanges in the pentatricopeptide repeat array of PPR56 resulted in predictable re-targeting to a modified target and in fundamental shifts in the respective sets of off-targets. We conclude (i) that the function of native plant PPR-type RNA editing factors is not restricted to genetic systems of a prokaryotic type, but that they can faithfully operate in the nucleo-cytosolic environment of human cells and (ii) that their RNA targeting behavior can be easily manipulated. Accordingly, they may in the future possibly even be employed for designed RNA editing of human, and likely also other eukaryotic transcriptomes and may be developed into a valuable tool complementing genome editing approaches.

## MATERIALS AND METHODS

### Cloning of N-terminally tagged PPR protein constructs

Plasmids based on pETG_41K_MCS harboring the coding sequences of wild-type *Physcomitrium patens* editing factors PPR56 and PPR65 and their 46 bp targets (28) were used to amplify PPR protein target combinations using Q5 polymerase (New England Biolabs) and primers with restriction site overhangs (Supplementary Table S1, Integrated DNA Technologies). After digest (FastDigest enzymes *ApaI* and *ScaI*, Thermo Fisher Scientific; CutSmart enzymes *MscI* and *NotI*, New England Biolabs), the constructs were introduced into the dephosphorylated (FastAP, Thermo Fisher Scientific) eukaryotic expression vectors pEYFP-C1 and pCMV-HA (Clontech TaKaRa), respectively, to create the final fusion protein coding sequences. Plasmid DNA for all constructs was isolated and purified using the NucleoBond® Xtra Midi kit (Macherey Nagel). Construct sequences were verified by Sanger Sequencing (Macrogen Europe). To generate constructs with coding sequence mutations, rolling-circle (38) or overlap extension PCRs (39) were performed (for oligonucleotides see Supplementary Table S1). *E. coli* RNA editing experiments with mutated PPR protein versions inserted in petG_41K_MCS with their 46 bp targets were performed as outlined in (28).

### Expression of PPR fusion constructs in human cell cytosol

HeLa, HEK-293 and MCF-7 cells were grown in DMEM and IMR-90 cells in MEM (Pan Biotechnologies) media, supplemented with 10% fetal calf serum and 1% Penicillin/Streptomycin, respectively, and incubated at 37°C and 5% CO_2_. The day before transfection, cells were seeded into six-well plates. Medium was replaced and supplemented with 25 μM zinc sulfate and cells were transfected with 3 μg plasmid using 12 μl PEI MAX (Polyscience) following the PEI MAX user manual. Cells were incubated at 37°C and 5% CO_2_ until harvest. Expression of EYFP constructs was verified via fluorescence microscopy. Cells were harvested by trypsinization (Trypsin/ EDTA 0.5%, Pan Biotechnologies) after 14, 20 or 40 h. Samples were pelleted at 300 g for 5 min at 4°C, washed with cold PBS, frozen in liquid nitrogen and stored at −80°C.

### Immunofluorescence and imaging

To verify the expression of recombinant proteins in human cells, cells were seeded on 12 mm cover slips. After transfection, cells were fixed in 4% paraformaldehyde (PFA) in PBS, pH 7.4 at room temperature for 15 min. Next, cells were permeabilized with 0.5% Triton in PBS for 5 min and blocked in Roti Immunoblock (Roth) at 4°C overnight. To stain HA (Hemagglutinin peptide)-tagged or EYFP (Enhanced Yellow Fluorescent Protein)-tagged proteins, respectively, the fixed cells were incubated for 1 h in a 1:500 diluted solution of primary antibody (α-HA rabbit, abcam ab9110, or monoclonal α-GFP mouse, Roth, respectively), washed in PBS and afterwards incubated for 1 h in a 1:1000 diluted Alexa594 labelled secondary antibody (Thermo Fisher) solution and DAPI (4,6-diamidino-2-phenylindole). After final washing, the cells were mounted on microscope slides using Fluoromont-G mounting medium (Southernbiotech). The localization of EYFP- and HA-tagged PPR proteins was examined on a Nikon Eclipse Ti2 system, equipped with a PlanFluor 40× Oil objective (NA 1.3) using the NIS-Elements AR software and ImageJ/Fiji version 1.53c for Windows. Transfection rates were calculated using Fiji as outlined in Supplementary Figure S1.

### Western blot

Cell transfection was executed as described above. After harvesting, half of the cells were pelleted and frozen. The pellets were resuspended in 50 μl 4× sample buffer (30 mM Tris, 1% SDS, 5% glycerol, 0.005% bromphenolblue, 50 mM DTT), vortexed, heated for 10 min at 98°C under continuous shaking and centrifuged for 10 minutes at 11 000 g. 5 μl were used for SDS polyacrylamide gel electrophoresis (SDS-PAGE). Proteins were transferred to a nitrocellulose membrane via wet electroblotting. After test-staining of total protein with Ponceau, the membrane was blocked with 1:10 RotiBlock for 60 min. For HA-tagged constructs or EYFP-tagged constructs, membranes were incubated with primary α-HA antibody (rabbit, abcam ab9110) or monoclonal α-GFP mouse (Roth) antibody, respectively, diluted 1:2000 in 1:10 RotiBlock, at 4°C overnight or at room temperature for 1 h under careful shaking. After washing with TBS-T (10% TBS, 0.05% Tween 20) for 5 min three times, membranes were incubated with the respective peroxidase conjugated secondary antibody (Jackson ImmunoResearch), diluted 1:10 000 in PBS, for 30–60 min at room temperature under careful shaking. Washing was repeated. The immunoblots were overlayed with a 1:1 Luminol mixture (Solution A and B Amersham ECL start Western blotting detection reagent) and chemiluminescence was imaged via ImageQuant LAS 4000 mini and the AxioVision software. Subsequently, the membranes were incubated at room temperature for one hour with a primary α-tubulin rabbit (Cell Signaling cat. #2125) antibody, diluted 1:1000 in 1:10 RotiBlock, washed three times for 5 min in TBS-T and further incubated for 30 min with a peroxidase conjugated secondary antibody (Jackson ImmunoResearch), diluted 1:10 000 in PBS. Initiation and imaging of chemiluminescence was repeated.

### Detection of C-to-U RNA editing

To investigate construct transcription and RNA editing, total RNA was isolated using an RNA extraction kit (Macherey Nagel or BLIRT). A DNase I (Thermo Fisher Scientific) treatment was executed to prevent DNA carryover. For reverse transcription (RevertAid reverse transcriptase, Thermo Fisher Scientific), Oligo dT_18_ primers (10 μM per assay, Integrated DNA Technologies) were used. A reverse primer binding downstream of the target and a forward primer binding in the PPR protein coding region were used for RT-PCR amplification (for oligonucleotides see Supplementary Table S1). PCR assays contained a cDNA amount that corresponded to 55 ng of RNA, 0.2 μM of each primer, 1× recommended PCR buffer, 0.2 mM dNTPs, 1 U GoTaq polymerase (Promega) and double-distilled water in a total volume of 25 μl. Amplification assays included 5 min initial denaturation at 94 °C followed by 35 cycles each with 30 s denaturation at 94°C, 30 s annealing at 52°C, 2.30 min synthesis at 72 °C, and a final step of synthesis for 5 min at 72°C. PCR products were gel-purified (BLIRT kit) and Sanger sequenced (Macrogen Europe). Sequencing chromatograms were analyzed with MEGA 7 (40) and Bioedit 7.0.5.3 (41). RNA editing was quantified by the ratio of the thymidine peak to the sum of thymidine and cytidine peaks in the editing position. For each construct and experimental condition, at least three independent replicates were investigated, when editing was detected. Presented editing rates are mean values of all replicates with standard deviations as indicated. The absence of RNA editing was confirmed by the evaluation of at least two replicates. All individual RNA editing experiments are listed in Supplementary Table S2.

### Total RNA sequencing

To investigate off-targets of EYFP-PPR56 and HA-His_6_-MBP-PPR65 in the IMR-90 transcriptome, 1.2 × 10^6^ IMR-90 cells were seeded on 10 cm culture dishes. The next day, media was replaced (MEM Eagle, Pan Biotechnologies, supplemented with 10% FCS, Thermo Fisher Scientific, 1% Penicillin, 1% Streptomycin and 25 μM zinc sulfate) and cells were transfected with 18 μg of plasmid DNA using 72 μl of PEI MAX reagent (Polysciences) per plate. An EYFP expression plasmid was co-transfected with the HA-His_6_-MBP-PPR65 expression plasmid in a 2:8 p(EYFP):p(HA-His_6_-MBP-PPR65) ratio to allow Fluorescence Activated Cell Sorting (FACS) of transfected cells. As control, cells were transfected with plasmids, encoding only the EYFP or EYFP and HA-His_6_-MBP protein tags, respectively. The media was exchanged after 6 h. 20 h after transfection, the cells were trypsinized, pelleted and taken up in cold PBS. 100 μl aliquots of the cell suspensions were retrieved prior to cell sorting, to confirm the editing efficiencies as described above. EYFP-positive cells were FACS-sorted at the Flow Cytometry Core Facility of the University of Bonn. The EYFP positive cells were pelleted, frozen in liquid nitrogen and stored at -80°C. RNA isolation was carried out using an RNA purification kit (Macherey Nagel) and subsequent DNase I treatment was executed as described above. Library preparation (polyA enrichment) and Illumina sequencing (150 bp paired-end) were performed by Novogene. Total RNA was sequenced for a minimum of three independent replicates per functional PPR56 and PPR65 construct, respectively. A total list of RNASeq samples is given in Supplementary Table S3.

### Identification of off-targets

RNASeq raw data was quality-checked through FastQC analyses (www.bioinformatics.babraham.ac.uk/projects/fastqc). Adaptors and low-quality sequences were trimmed, using Trimmomatic 0.39 (42) with the options ILLUMINACLIP:2:30:10, LEADING:15, TRAILING:15, SLIDINGWINDOW:4:24 and MINLEN:80. Overrepresented plasmid sequences were removed using BBDuk of the BBTools suite (bbduk.sh, Bushnell B., sourceforge.net/projects/bbmap) with default settings and the options out/outm, *k* = 31 and hdist = 2.

To generate specific transcriptome references for the IMR-90 cell lines, control samples expressing EYFP (PPR56 reference) or EYFP and HA-His_6_-MBP (PPR65 reference) were sequenced. For that purpose, the control RNA reads were mapped to the NCBI human GRCH38 RefSeq Transcripts (www.ncbi.nlm.nih.gov/genome/guide/human, last accessed 24.11.2021) via BBMap with default settings and options outu/outm, maxindel = 200k, mdtag = true, sam = 1.4 and pairedonly = true. The SAM output file was converted into BAM format via samtools (v1.13) view (43). The abundance of reads per transcript was estimated by an alignment-based Salmon quantification with standard settings (44). All transcripts of the GRCH38 RefSeq Transcripts FASTA, which had an estimate of at least 10 mapped read pairs (NumReads ≥ 10), were combined in one FASTA file, using R v4.1.1 (R Core Team, 2021, www.R-project.org) and RStudio v1.2.5033 (RStudio Team, 2019, www.rstudio.com) to be used as customized transcriptome in subsequent analyses.

The trimmed reads of each sample were mapped to the generated customized transcriptome individually using bbmap.sh (BBMap, Bushnell B., sourceforge.net/projects/bbmap/) with default settings and the options outu/outm, maxindel = 200k, mdtag = true, sam = 1.4, subfilter = 3 and pairedonly = true. The output SAM files were converted into BAM format (samtools view), deduplicated (samtools fixmate, samtools markdup), coordinate-sorted (samtools sort) and indexed (samtools index) via Samtools 1.10.

SNPs between the sample RNAs and the two customized reference transcriptomes were called through JACUSA v2.0.2 (45,46). The call-2 method using both the sample and reference reads simultaneously was applied (options -m 3; -q 25; -T 1.56; -a H:condition = 1,M,B,Y and -f V). Only SNPs that were called in at least two replicates of a given sample, but not in any sample expressing the respective other editing factor or the DYW domain mutant protein with lost editing functionality (PPR56 with DCH modified into DAH, see Figure 3) were considered further. RNA editing rates were defined as the ratio of edited to total RNA reads at a specific site, added up for the respective replicates. Only SNPs with (i) a clean cytidine background >99%, (ii) a low thymidine background <0.5% in the mapping references, (iii) coverage by at least 20 RNA reads each in samples and the respective reference and (iv) editing efficiencies of at least 1.5% were considered further. Selected SNP positions were evaluated manually via Tablet (47) and SNPs in questionable mapping regions were excluded. Off-targets were extracted together with 30 nucleotides upstream and five nucleotides of downstream sequence with a custom-made bash script (kindly provided by P. Gerke). Labeling of off-targets was automatized with a custom-made R script (established with kind help of S. Zumkeller) using R v4.1.1 (R Core Team, 2021, www.R-project.org) and RStudio v1.2.5033 (RStudio Team, 2019, www.rstudio.com).

### Identification of candidate off-targets

To scan the customized transcriptome based on NCBI human GRCH38 RefSeq Transcripts for putative editing targets of PPR56, the TargetScan tool of PREPACT (48) was employed. The weight matrix used to search for putative targets is shown in Supplementary Figure S2. The top scoring predicted editing targets were compared to the actual detected off-targets.

## RESULTS

### Molecular cloning of RNA editing factors PPR56 and PPR65 fused to different protein tags

Pentatricopeptide repeat (PPR) proteins PPR56 and PPR65 are C-to-U RNA editing factors functionally characterized in the model moss *Physcomitrium patens* (34,49). Both proteins feature the typical arrays of canonical P-type PPRs along with ‘long’ L-type and ‘short’ S-type PPR variants, mostly arranged in PLS-type triplets that are responsible for RNA target recognition. The PLS-type PPR arrays are followed by ‘extension’ domains E1 and E2 of presently still unclear function and the carboxyterminal DYW-type cytidine deaminase domain (Figure 1A). Importantly, both editing factors were recently shown to faithfully edit their co-transcribed targets in a bacterial setup and assay system using *E. coli* (28), which made them prime candidates also for testing in other heterologous setups.

Because PPR proteins are generally known, at least in bacterial systems, to be recalcitrant against functional heterologous expression, likely owing to the repetitive structure of their PPR arrays (e.g. 50,51), and because success appears to be particularly dependent on specific N-terminal makeups in recombinant constructs (Lesch *et al.*, unpublished findings), we first wished to test different arrangements of N-terminal tags for expression in human cell lines (Figure 1B). Both RNA editing factors were cloned behind variably combined sets of protein tags including the small Hemagglutinin (HA) and/or His_6_ tags, the Maltose Binding Protein (MBP) that proved successful for expression in *E. coli* and EYFP, the Enhanced Yellow Fluorescent Protein (Figure 1B). The respective native RNA editing targets with sequences 40 bp upstream and 5 bp downstream of the editing position as used previously (28) were cloned downstream of the recombinant protein sequences, now followed by a eukaryotic polyadenylation signal sequence derived from the SV40 virus. Expression of the combined transcript in human cells was driven by the strong eukaryotic CMV (Cytomegalovirus) promoter.

### Testing PPR56 and PPR65 constructs for editing their native targets in HeLa cells

**Figure 2.**
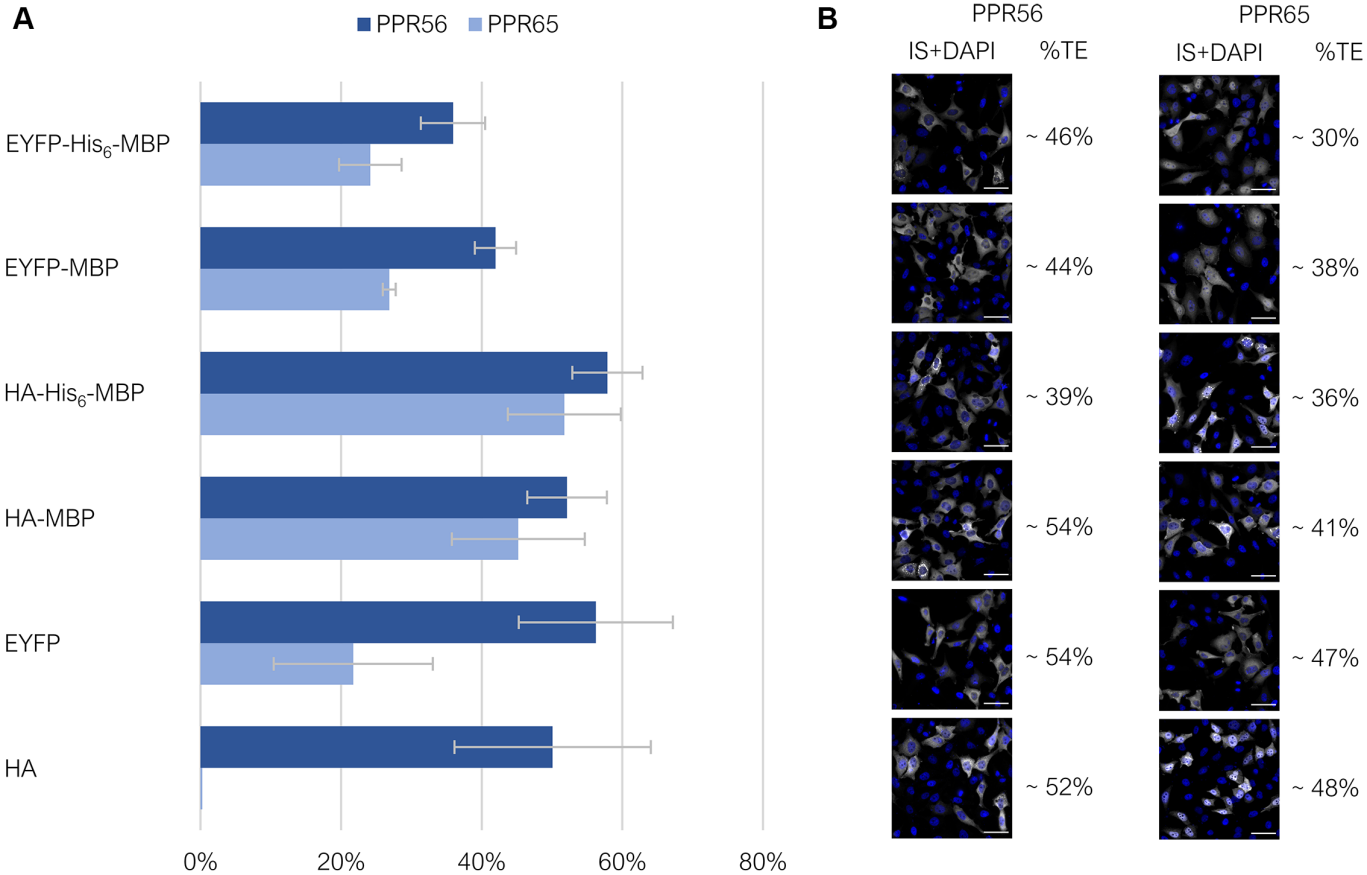
RNA editing by PPR56 and PPR65 constructs of their co-provided native targets in HeLa cells. RNA editing of the respective targets ccmFCeU103PS and nad4eU272SL, respectively, was determined from bulk sequencing of RT-PCR products after transfection of the differently tagged PPR56 and PPR65 constructs (see Figure 1B) into HeLa cells and incubation for 20 h. (**A**) Observed RNA editing efficiencies varied widely and reached up to 58% for the HA-His_6_-MBP-PPR56 construct. Data are based on a minimum of three biological replicates (independently transfected cells) for each construct (Supplementary Table S2) (**B**) Approximate transfection efficiencies (%TE) were determined from the ratio of immuno-stained HA- and EYFP-positive cells (IS) to DAPI-signals. Shown are example images, scale bar: 50 μm. For more extensive documentation see Supplementary Figure S1.

All PPR56 and PPR65 construct variants (Figure 1B) were initially transfected into human HeLa cells with comparable transfection rates and incubated for 20 hours prior to harvesting. RNA editing of the provided native targets was assessed after cDNA synthesis and specific RT-PCRs covering the respective sequences (Figure 2, Supplementary Table 2 including data on all individual RNA editing assay replicates). RNA editing of up to 58% was indeed observed for eleven out of twelve recombinant constructs, the only exception being PPR65 equipped with the HA tag alone. Notably, an evidently lower protein expression was detected for the HA-PPR65 construct in our accompanying immunoblot analyses (Supplementary Figure S3). For both editing factors, the most efficient editing results were obtained for the MBP constructs in combination with the small HA and His_6_ tags. Addition of EYFP resulted in reduced editing efficiencies for PPR65. This, however, was not an effect of EYFP *per se* since the EYFP-PPR56 construct revealed RNA editing efficiency comparable to the medium-size MBP constructs. Overall, PPR56 was confirmed to be a more robust and efficient editing factor in comparison to PPR65 as we will discuss below, congruent with results obtained in the *E. coli* setup (28 and unpublished findings).

### DYW domain mutations confirm its role as the functional cytidine deaminase

**Figure 3.**
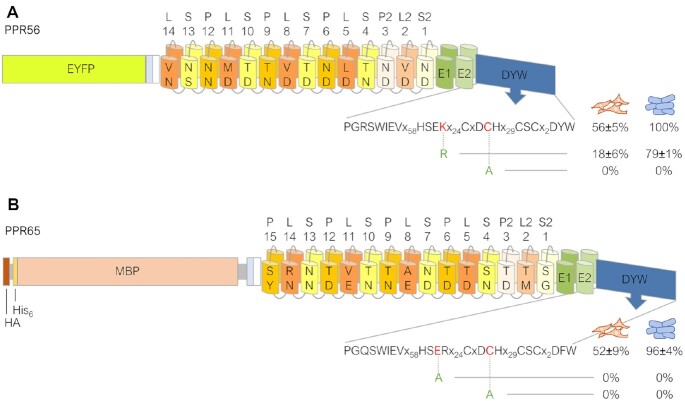
Mutations in the DYW domains of PPR56 and PPR65 result in strong reduction of editing. Constructs EYFP-PPR56 (**A**) and HA-His_6_-MBP-PPR65 (**B**) were selected for mutating key residues in their respective DYW domains. Exchanging the highly conserved cysteine in the DCH motif for alanine abolishes editing activity of both factors completely. Likewise, replacing the highly conserved glutamate in the HSER motif with alanine destroys editing activity of PPR65. The conservative exchange of lysine by arginine in the HSEK motif of PPR56 reduces editing from 56% to 18%. RNA editing efficiencies and standard deviations are shown for at least three replicates each (in case of absence of editing for at least two replicates). For a complete list of results from individual replicates see Supplementary Table S2. The effects of the corresponding mutations observed in the *E. coli* setup with recombinant His_6_-MBP tagged PPR proteins are indicated to the right (blue cell icons) of the human cell (orange cell icons) data.

The RNA editing activities observed for the PPR56 and PPR65 constructs could theoretically be due to cytidine deaminase activities endogenously present in the human cells used here (e.g. of the APOBEC-type) that were secondarily targeted to the introduced artificial targets by promiscuous protein-protein (a/o protein-RNA) interactions. To explore this possibility, we introduced two single amino acid mutations each into the DYW domains of both RNA editing factors that could be expected to affect the known Zn^2+^-binding centers in the DYW domains and thus abolish cytidine deaminase activity (Figure 3). Changing the highly conserved cysteines (C) in the DCH motifs to alanine as well as replacing the highly conserved glutamate (E) by alanine in the DYW domain of PPR65 indeed abolished RNA editing of PPR56 and PPR65 completely. In contrast, the much more conservative exchange of the basic lysine (K) to arginine (R) in the HSEK motif of PPR56 did not abolish RNA editing activity completely but reduced it significantly from 56% to 18%. Notably arginine is frequently found at this position in native plant DYW domains, including the one of PPR65 (Figure 3). Aside from the overall higher editing activities in the bacterial setup, congruent data were obtained for the *E. coli* assay system (28).

### RNA editing dependent on incubation time and zinc supplements

**Figure 4.**
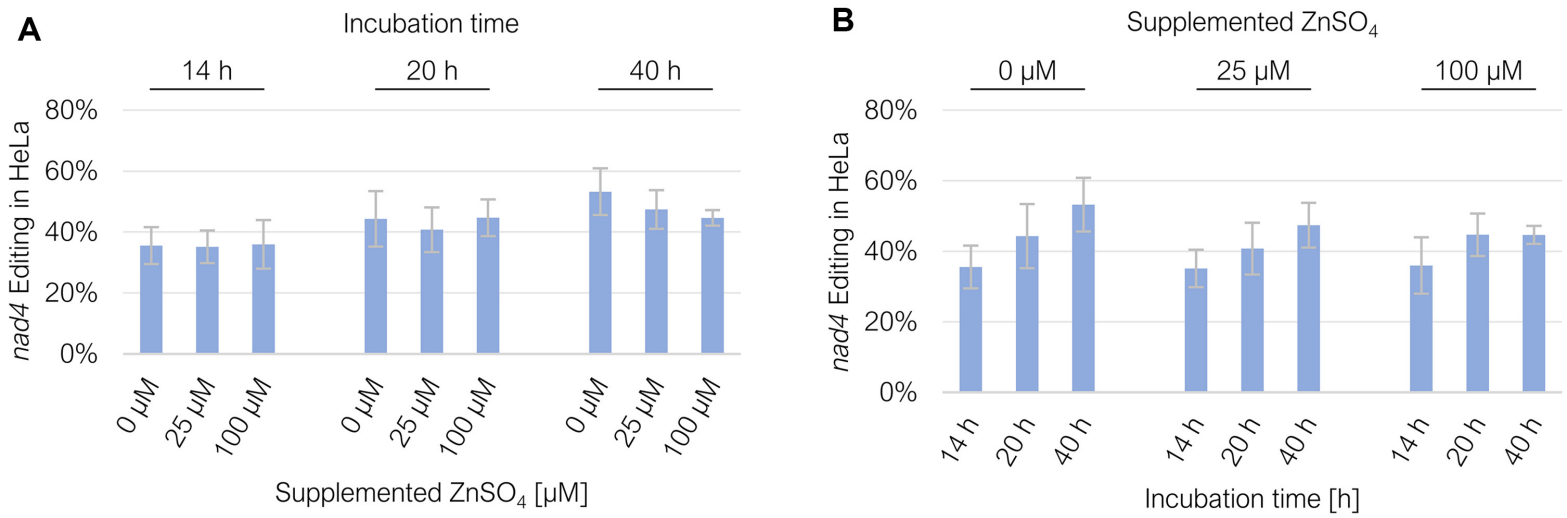
Exploring variable experimental conditions to detect RNA editing in HeLa cells. RNA editing in HeLa cells was tested for the EYFP-PPR56 construct and its native *nad4* target under different incubation times of 14, 20 and 40 h after transfection, and with variable Zn^2+^ supplementations of 0, 25 and 100 μM ZnSO_4_. No beneficial effect was observed for added Zn^2+^ at any of the three incubation times (**A**), whereas increased incubation times yielded higher editing efficiencies (**B**). For a complete list of results from individual replicates see Supplementary Table S2.

To potentially optimize the degree of RNA editing in the human cell line assays, we tested alternative incubation times (14, 20 and 40 h post transfection and prior to harvest, respectively) and the addition of zinc supplements (25 and 100 μM, respectively) to the HeLa cell culture media (Figure 4). The supplementation of ZnSO_4_ in the standard culture media (*a priori* containing approximately 4 μM zinc) could not enhance RNA editing. It rather yielded the opposite effect that became visible with the presence of 100 μM Zn^2+^ at extended incubation times of 40 h, likely reflecting an overall zinc toxicity at this concentration after extended incubation times (Figure 4A). This is in accord with *in vitro* studies confirming that low zinc concentrations are sufficient to supply the two Zn^2+^ ions coordinated in an active DYW domain (27,29). In contrast, extended incubation times increased the percentage of detected C-to-U-edited RNAs (Figure 4B), likely indicating that the artificially induced RNA editing is a moderately slow process in the dividing human cells before a stationary phase may eventually be reached in the steady state transcriptome. We conclude that supplementation of zinc to the rich standard culture media is not fundamentally necessary, but that an extension of incubation time to ca. two days may be helpful, especially for scoring low-efficiency editing events. For practical reasons, all further experimentation was performed with incubation times of 20 h and addition of 25 μM zinc sulfate.

### Testing RNA editing capacities in different human cell lines

**Figure 5.**
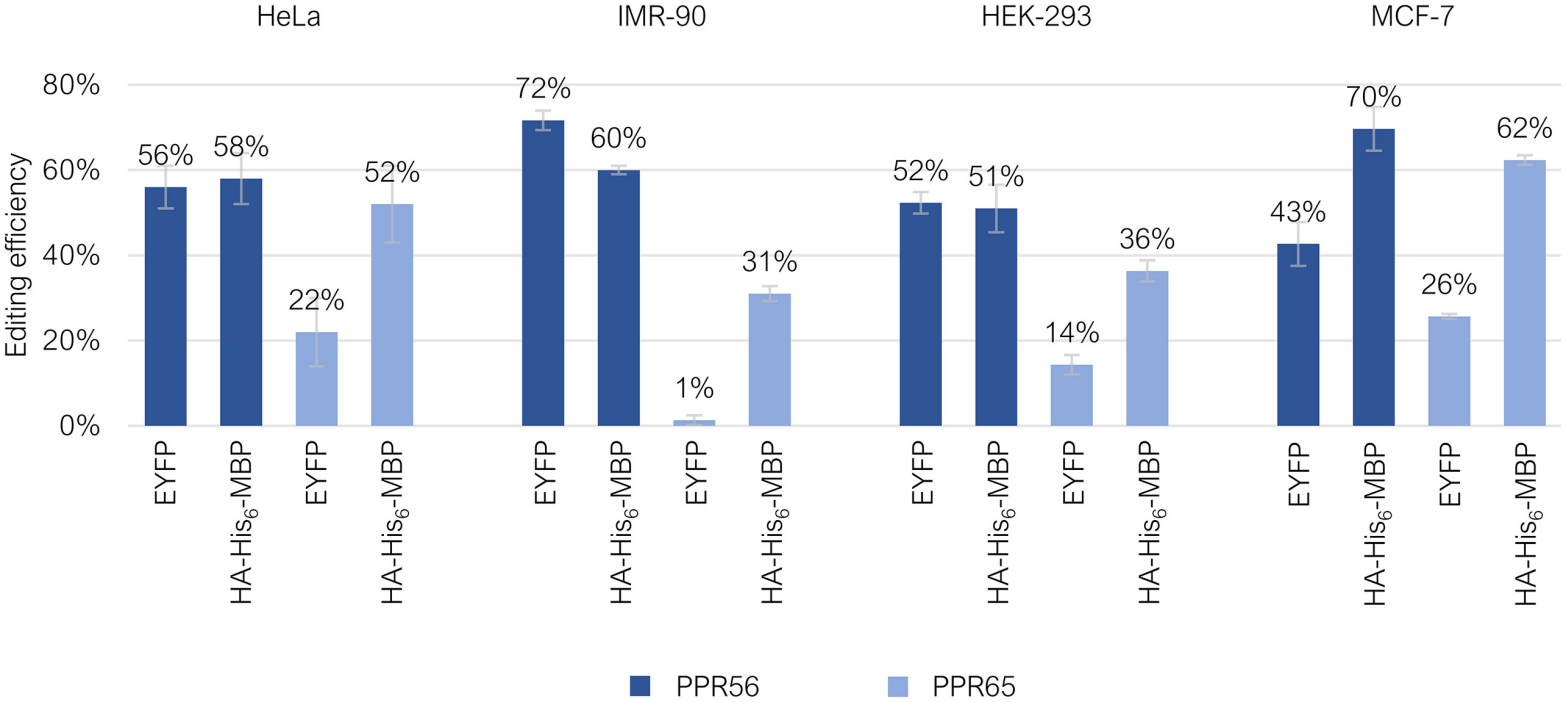
Exploring RNA editing in different human cell lines. To test for editing in different human cell lines, two selected constructs of RNA editing factors PPR56 and PPR65, each, in fusion with either the EYFP or the HA-His_6_-MBP-tag, which initially showed different frequencies of RNA editing in HeLa cells, were alternatively transfected into IMR-90, HEK-293 and MCF-7 cells. RNA editing efficiencies and standard deviations are shown for at least three replicates each. For a complete list of results from individual replicates see Supplementary Table 2.

To check whether plant-type RNA editing could similarly be recapitulated in other human cell lines, four selected constructs (EYFP-PPR56/PPR65 and HA-His_6_-MBP-PPR56/PPR65) showing different frequencies of RNA editing in the initially tested HeLa cells were alternatively transfected into IMR-90 (non-transformed human lung fibroblast cell line), HEK-293 (human embryogenic kidney cells) and MCF-7 (Michigan Cancer Foundation-7, a breast cell cancer cell line) cells (Figure 5). All three alternative human cell lines likewise enabled the detection of RNA editing at the respective native targets of PPR56 and PPR65. The PPR56 constructs consistently yielded higher efficiencies in all cell lines, reaching 72% of editing for the EYFP-PPR56 construct in the IMR-90 cell line. *Vice versa*, the EYFP-PPR65 construct consistently showed lowest editing efficiencies, even dropping to only 1% in the IMR-90 cell line assays. We selected the IMR-90 cell line with the EYFP-PPR56 construct for further downstream experimentation by site-directed mutagenesis given the efficient RNA editing of the native *nad4* target and for scoring off-targets in the human cell transcriptomes, because it conveniently allowed for straightforward enrichment of successfully transfected cells by FACS (Fluorescence Activated Cell Sorting).

### More than 900 off-targets of PPR56 in the human cell background transcriptome

**Figure 6.**
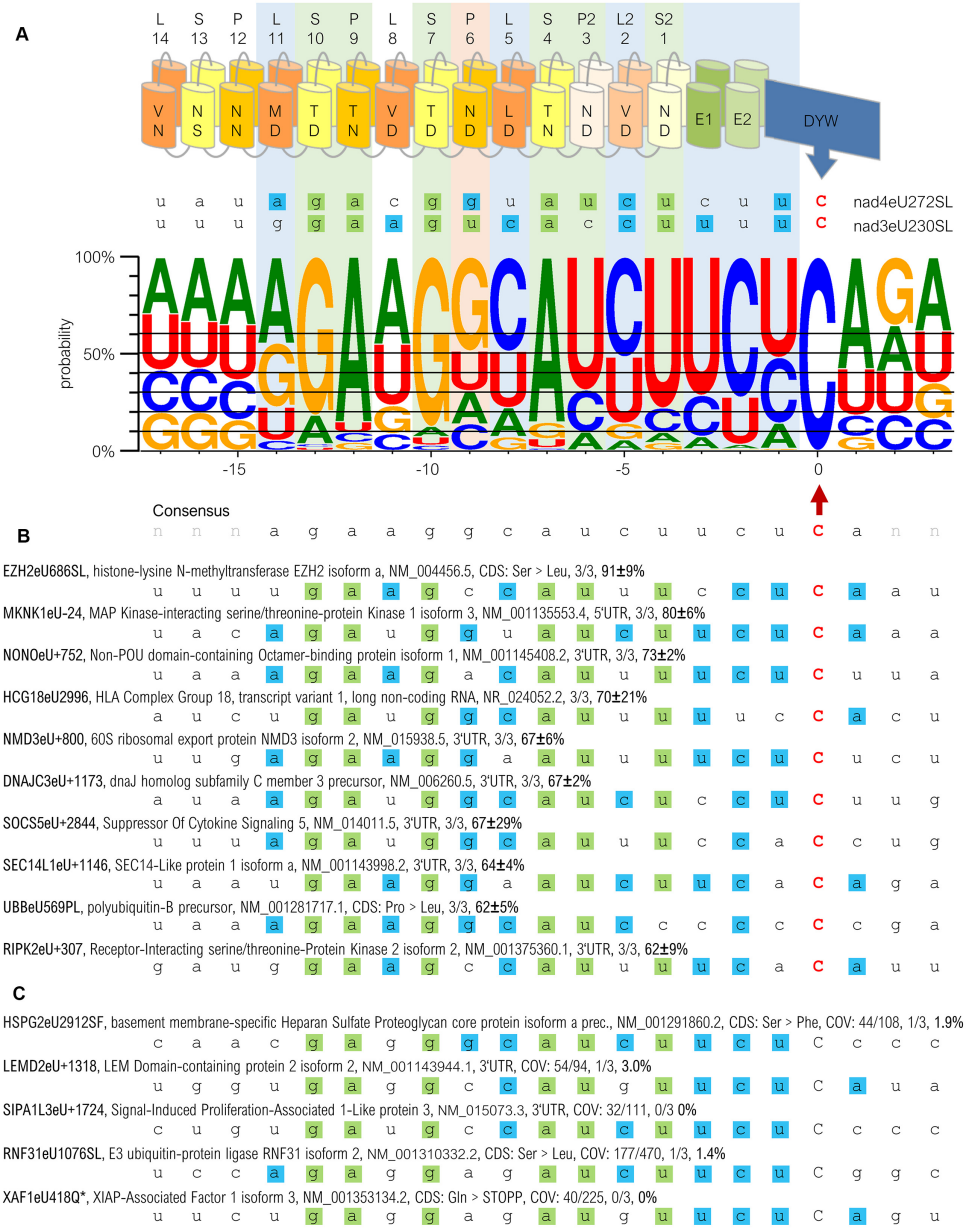
Off-targets of PPR56 in FACS-sorted IMR-90 cells. (**A**) Expression of EYFP-PPR56 causes off-target C-to-U RNA editing in the endogenous transcriptome of IMR-90 cells, summarized with a WebLogo (97) created from the sequence environments of 759 edited off-targets (74 putative binding shift candidates were excluded and identical target sequences in different transcript variants of a given gene were only counted once). Targets were juxtaposed with the PPR array of PPR56 aligning the terminal PPR S2-1ND with position −4 upstream of the edited sites (position 0). The horizontal lines indicate arbitrary cut-offs in steps of 10%, starting with 40% for a nucleotide dominating in a given position. Matches according to the established PPR-RNA code (see Figure 1) are shaded green. Evident nucleotide preferences in positions −3, −2 and −1 and in positions −14, −8 and −5 opposite of the L-type PPRs L-11MD, L-5LD and L2-2VD are highlighted by light blue shading. Orange shading of P-6ND highlights its conceptual misfit to a dominating guanidine instead of an expected uridine. (**B**) A detailed listing of the 10 top-edited off-targets reveals their good matches to the overall consensus profile. Labeling also of the off-target edits uses our proposed nomenclature indicating positions within a coding sequence of the respective gene product and the resulting codon changes with capital letters or with the plus or minus symbol indicating edits in 3’- or 5’-UTRs, respectively (52,98). Description also includes protein name, NCBI accession number, number of replicates with detected editing and average percentage of editing. A complete list of off-targets is available as Supplementary Table S4A. (**C**) Five exemplary candidate sites are shown, that were not found to be edited or only to very limited degree below our thresholds in single replicates, despite good overall matches to the PPR array of PPR56 (and the consensus off-target profile) in positions −13 to + 1. Coverage of transcripts (COV) in the control sample / in sum of PPR56 replicates is listed in addition. Sites were investigated using IGV Version 2.3.98 (99) for transcriptome analyses.

To evaluate if the RNA editing activity of PPR proteins expressed in human cells goes beyond the co-expressed native RNA targets, we analyzed possible RNA editing of endogenous human transcripts by RNAseq analysis. Indeed, >900 off-targets reflecting events of C-to-U RNA editing in the endogenous transcriptome were detected in IMR-90 cells after transfection of the EYFP-PPR56 construct. A WebLogo created for the off-targets (Figure 6A, Supplementary Table S4) confirms expectations according to the PPR-RNA recognition code, most notably for S-10TD:G, P-9TN:A, S-7TD:G, S-4TN:A, P2-3ND:U and S2-1ND:U (for nomenclature see Figure 1). Surprisingly, no strong preference for U was observed contrary to what was expected for nucleotide position -9 opposite of the canonical PPR P-6ND. Intriguingly though, this matches the native situation for PPR56 *in planta* where a G is observed in position -9 of the nad4eU272SL target (Figure 6A), which is found to be more efficiently edited than its alternative nad3eU230SL target (52) despite the conceptually better fit of the latter with a uridine matching P-6ND.

Using an arbitrary threshold for a nucleotide occurring in at least 40% of off-targets identified significant bias in additional positions (Figure 6A). Most notably, there seem to be unexplained preferences for pyrimidines opposite of the L-type PPRs L-5LD and L-2VD and in positions −3, −2 and −1 directly upstream of the editing sites, again in perfect congruence with the two native targets in the moss (Figure 6A). Bias in the positions −3 to −1 may be caused by yet unrecognized selectivity exerted by the E1, E2 and/or the DYW domain (53–55). Furthermore, there is an evident preference for purines in position −14 juxtaposed with L-11MD, which likewise excellently matches A or G present in the native targets. The preferences for A and U in positions −11 of the off-target collection in contrast would favor the A present in the *nad3* target alone over the corresponding C in the *nad4* target. Finally, the somewhat weaker preferences (below our arbitrary 40% threshold) for A or U in positions −17, −16 and −15, likewise fit the native targets of PPR56 (Figure 6A).

Editing frequencies exceeded 60% for the top-scoring off-targets (Figure 6B). Most of these highly edited off-targets were found located in non-coding regions (3’ or 5’ UTRs) and are unlikely to have any functional consequences. Exceptions include an efficiently edited serine-to-leucine exchange in EZH2 and a proline-to-leucine exchange in the UBB coding sequence (Figure 6B). All of the top-edited off-targets excellently fit to the overall consensus.

*Vice versa*, we also checked for good candidate off-target sites that were not found to be edited. Such candidate positions were identified using the TargetScan module of the PREPACT software (48). Examples of non-efficient editing of consensus sites are show in Figure 6C, where we double-checked for missed editing events with our restrictive thresholds, but where we could not confirm any, or only marginal, C-to-U editing in single replicates (Figure 6C). Evidently this suggests further restrictions beyond the immediate binding preferences of PPR56 such as limited accessibility due to RNA secondary structure or protection by other RNA-binding proteins. Intriguingly, most of these non-edited candidate off-targets lacked matches to the overall ‘mild’ consensus in positions −14 and −11 juxtaposed to PPRs L-11 and L-8, for which no explanation is presently available.

### Single amino acid exchanges in its PPR array result in re-targeting of PPR56

**Figure 7.**
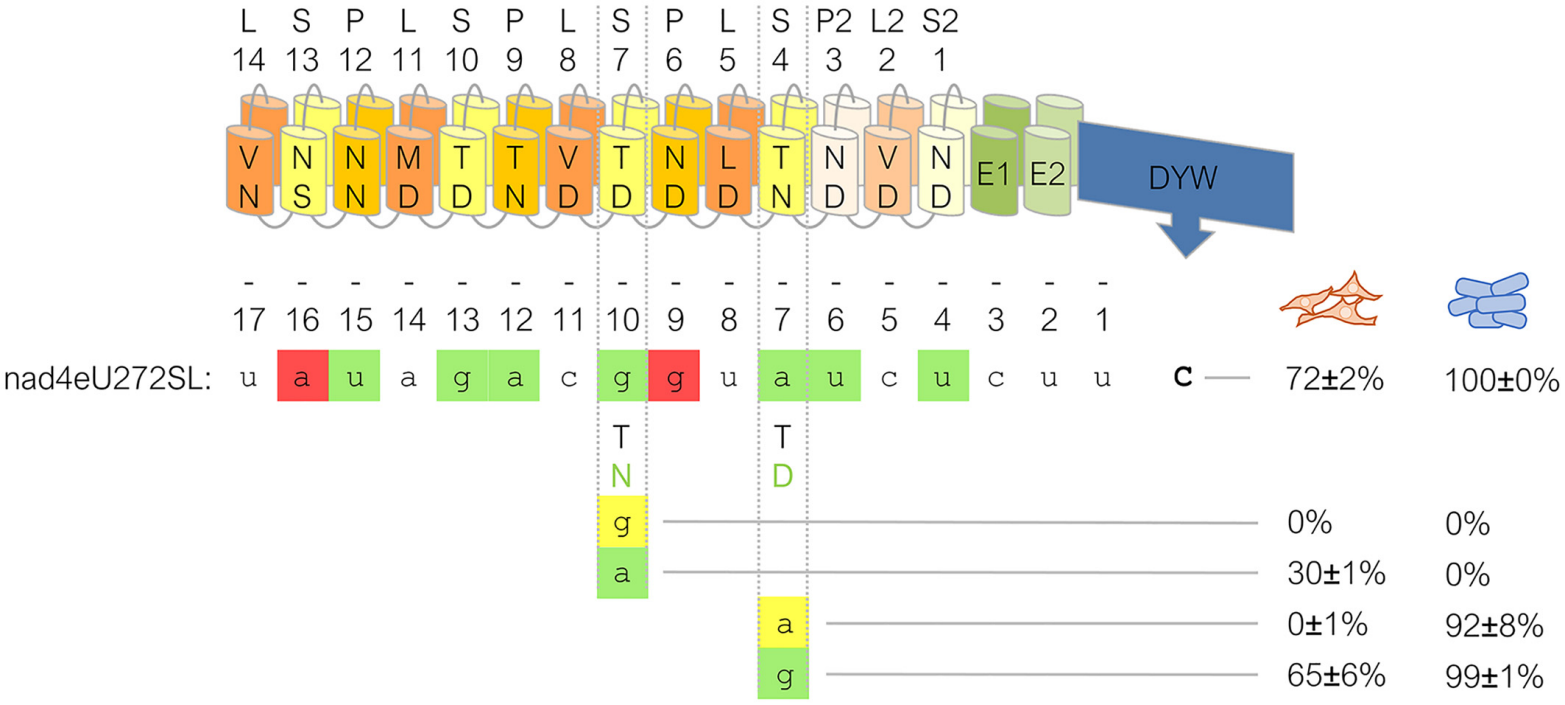
Retargeting of PPR56 by single amino acid exchanges in its PPR array. Single-site mutations were introduced into the ‘Last’ positions of the two PPRs S-7 and S-4 of PPR56 to change their preference for the one vs. the other purine nucleotide: S-7TD > TN and S-4TN > TD changing their (conceptual) preference from G to A and A to G, respectively. A resulting loss of editing at the native *nad4* target sequence in the human cells (orange cell icons) in both cases could be compensated by complementing nucleotide changes from G to A in position -10 and from A to G in position -7 upstream of the nad4eU272SL editing site (position 0) resulting in regain of 30% and 65% in the respective transcript populations. The effects of the corresponding mutations observed in the *E. coli* setup with recombinant His_6_-MBP-PPR56 are indicated to the right (blue cell icons). Shading of nucleotides is as in Figure 1, scoring of editing efficiencies as in Figure 3.

To explore the malleability of its RNA binding properties, we modified the codons for the crucial terminal (‘Last’) amino acids, whose identities are known to distinguish between keto and amino nucleotides, in two selected PPRs in PPR56 towards changing their preference for purine nucleotides. PPR S-7TD was changed to S-7TN changing its conceptual preference from G to A and, conversely, PPR S-4TN was altered to S-4TD changing its predicted preference from A to G (Figure 7). The two mutated PPR56 versions were first tested on the original native *nad4* target and both mutants were found to have lost their capacity to edit the nad4eU272SL site. Introducing a complementary mutation from G to A in position -10 of the *nad4* target sequence re-established RNA editing to 30% in the S-7TD > TN mutant. A complementary mutation exchanging A against G in position -7 re-established RNA editing to even 65% for the S-4TN > TD mutant (Figure 7).

For comparison, we introduced the same mutations into recombinant MBP-PPR56 for testing in the previously established assay system in *E. coli*. While RNA editing at the native *nad4* target was equally lost for the S-7TD > TN mutant in the bacterial setup, it could not be rescued by the corresponding G-to-A exchange in the target sequence (Figure 7). Moreover, and in contrast to the observation in the human cell line, RNA editing was only slightly reduced in the S-4TN > TD mutant. The reasons for these discrepancies are unclear. They could be due to the differences in the recombinant protein fusion constructs or to the different RNA or protein turnover rates in the prokaryotic vs. the eukaryotic setup. In the light of the differences in RNA editing efficiencies, however, now observed even between different human cell lines (Fig. 5), we consider effects of other cellular background elements, e.g. differing abundancies of other RNA binding proteins, a more likely explanation.

### Dramatic changes in the off-target spectrum for the single site PPR56 mutants

**Figure 8.**
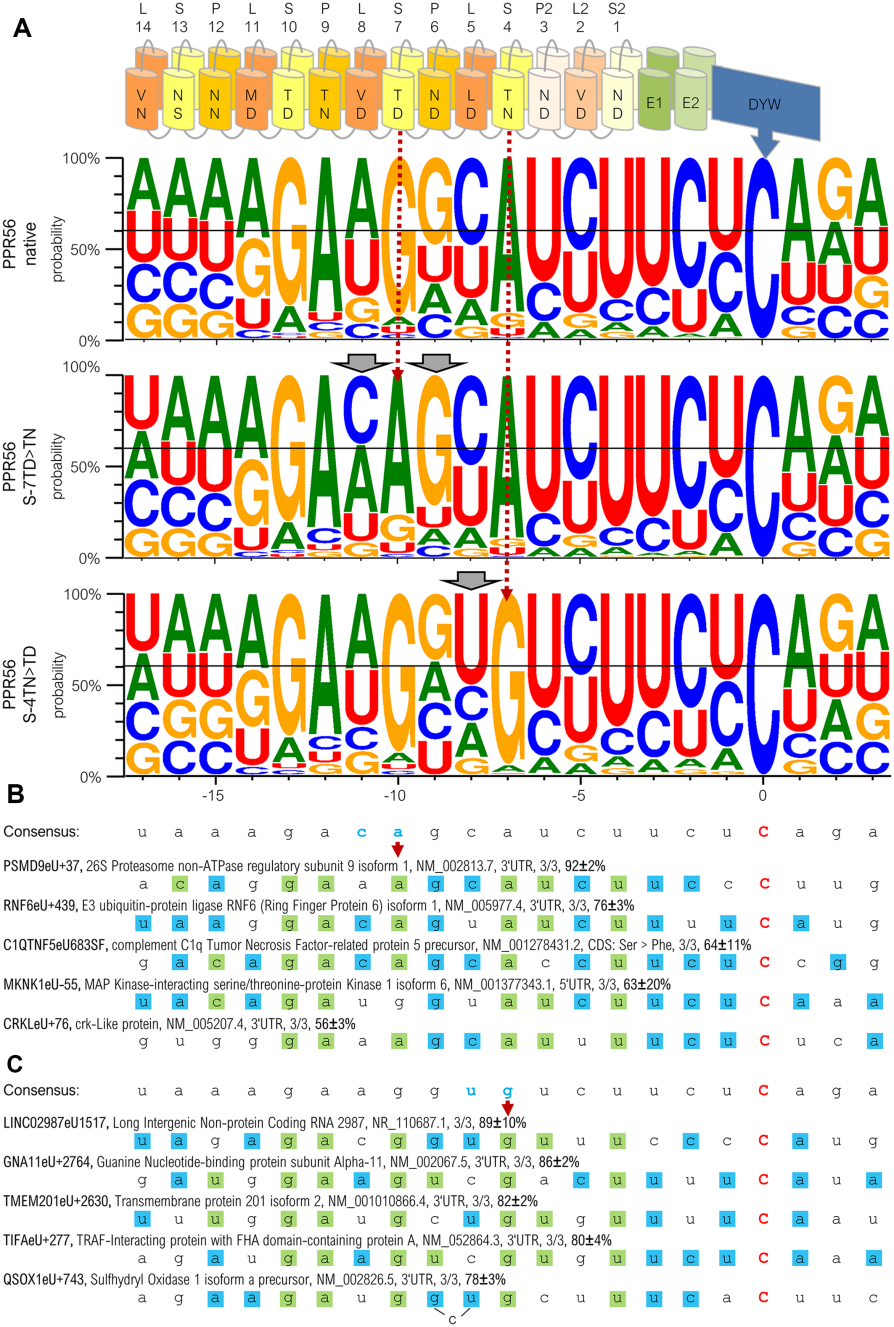
Significant shifts in the off-target sets of two PPR56 mutants. (**A**) WebLogo profiles (97) are shown for sets of RNA editing off-targets of the native PPR56 (top) and the two PPR mutants S-7TD > TN (middle) and S-4TN > TD (bottom) in IMR-90 cells (for a complete list of off-targets see Supplementary Table S4). Dramatic shifts are identified for the identities of nucleotides in positions −10 and −7 juxtaposed with the mutated PPRs (stippled dark red arrows), as expected. Additional shifts are also seen for neighboring nucleotide identities in positions −11 and −9 for the S-7TN mutant and in position −8 for the S-4TD mutant (grey arrowheads). Five most efficiently edited off-targets each in the analyzed transcriptomes for the S-7TN mutant and the S-4TD mutant are shown in panels **B** and **C**, respectively. Labeling includes protein name, NCBI accession number, number of replicates with detected editing and average percentage of editing. A bulged C in position −9 would improve the overall match of the S-4TD mutant to target QSOX1eU + 743.

The successful functional redirection of the RNA editing activity of PPR56 by the single amino acid exchanges in selected PPRs (Figure 7) led us to assume that this would also result in significant shifts of the respective off-target sets for the two protein mutants. Accordingly, we performed transcriptome analyses to investigate the changes in the off-target spectra and indeed found significant redirection in the set of off-targets that could be identified for the two mutated PPR56 variants (Figure 8). The intended changes of the purine identities are clearly seen for position **−**10 changing from G to A for the S-7TN mutant and, yet more drastically, from A to G for the S-4TD mutant. While all other positions likely affected by the PPR array and up to position + 3 behind the editing site showed no major changes in the conservation profiles, there were three notable exceptions. Most significantly, a cytidine in position -11 was shifted from minority occurrence in the case of the wild-type PPR56 protein (and the S-4TD mutant) to being the dominant nucleotide in the S-7TN mutant off-target collection. Similarly, although less dramatically, also in the case of the S-4TD mutant off-target collection we found a shift in the conservation profile for the nucleotide directly upstream of the one targeted by the mutation introduced into the PPR. Finally, we found that the moderate, and non-canonical selectivity for G exerted by P-6ND is significantly more pronounced in case of the S-7TN mutant.

Prime examples for off-targets efficiently edited by the S-7TN mutant protein (Figure 8B) or by the S-4TD mutant protein (Figure 8C), respectively, are perfectly in accord with the overall changes in the consensus profiles. A notable exception is off-target editing site −55 in the 5’-UTR of transcript MKNK1 isoform 6 featuring a G in position −10 instead of an A, which is, however, one of the most efficiently edited off-targets of the S-7TN mutant protein, but also edited by the S-4TD mutant and the native PPR56 (Figure 8B, Supplementary Table S4). This is a rather exceptional case, as only 32 off-targets are edited by all three PPR56 variants (off-targets counted, when detected in a minimum of two replicates per construct, Supplementary Table S4).

**Figure 9.**
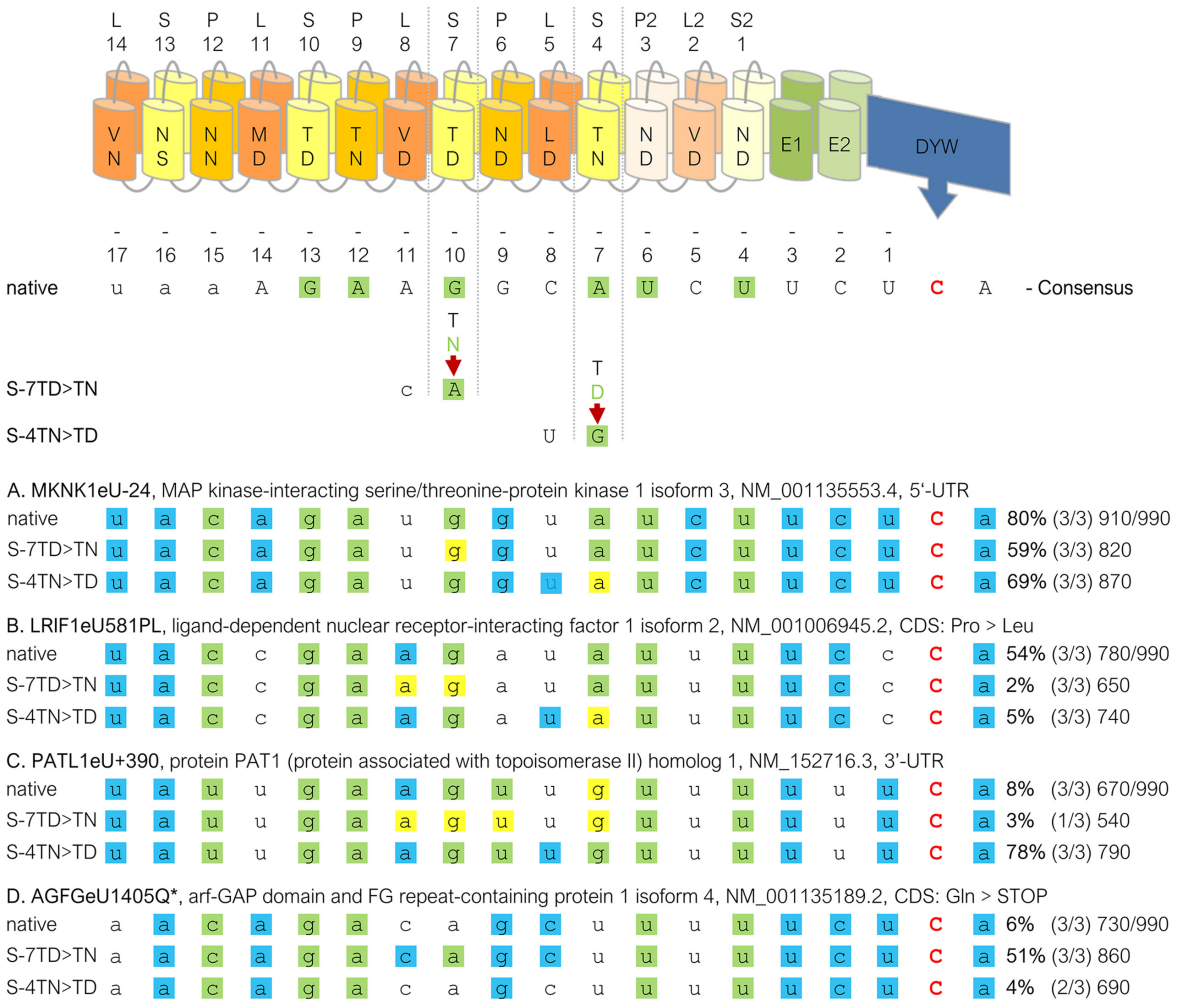
Shifts of editing efficiencies in PPR56 mutants. Examples of four selected off-targets in mRNAs for MKNK1 isoform 3 (**A**), LRIF1 isoform 2 (**B**), PATL1 (**C**) and AGFG isoform 4 (**D**) that are shared between the native PPR56 and the two mutant data sets S-7TD > TN and S-4TN > TD, respectively. The PPR array indicating P-, L- and S-type PPRs and the crucial positions 5 and L for each PPR are indicated on top. A consensus derived from the off-target conservation profile (Figure 6) of native PPR56 is shown below with nucleotide preferences below the 40% threshold shown with small letters. Differences between the two mutants are shown below and matches according to the PPR-RNA binding code are highlighted in green. For the selected off-targets, green shading indicates fit to the PPR-RNA code, blue shading indicates additional fits to the respective PPR56 consensus and yellow shadings indicate disfavored fit by the three different PPR56 proteins. Numbers indicate average percentage of RNA editing observed (bold) in a respective number of up to three replicates (Supplementary Table S4). Arbitrary total matching scores are indicated at the ends of the respective lines. Matching scores are calculated as the sum of individual percentages for each position in the consensus profile that is occupied with a nucleotide dominating at least 40% in steps of 10% (see Figure 6A), resulting e.g. in a score of 40 for an A in position −14 or a score of 70 for a G in position −13. The total maximum possible score of 990 is indicated behind the slash.

Quite expectedly, the changes in the off-target profiles are not only of a simple yes-or-no quality but also expressed in the different frequencies of RNA editing at some off-targets shared between the three data sets (Figure 9). An editing event in the 5’-UTR of the MKNK1 isoform 3 mRNA (Figure 9A) shows only moderate reduction in the two PPR56 variants with drops in editing efficiencies from 80% to 69% or 59%, correlating well with reduced arbitrary overall matching scores from 910 to 870 or 810 for the S-4TD and the S-7TN mutant, respectively (scoring as explained in Figure 9). Notably, the reduced match with an A in position -7 opposite of the mutated PPR S-4TD seems to be compensated by the collaterally improved match with the upstream uridine observed in the off-target consensus for the S-4TD mutant. More significant than the changes for the MKNK1 isoform 3 off-target are the more drastically reduced editing efficiencies for the codon-changing edit in the LRIF1 isoform 2 mRNA (Figure 9B) from 54% to only 5% or even only 2%, again in full accord with mismatches and the overall reduced matching scores (780 > 740 > 650) in the two PPR56 mutants.

A significant increase to an editing efficiency of 78% is found for an editing site in the 3’-UTR of the PATL1 mRNA for the S-4TD variant of PPR56 (Figure 9C) while the S7-TN variant at the same time reveals a drop in editing efficiency, again in full accord with PPR-RNA matches and the overall matching score. The opposite is found for improved efficiency in the S-7TN variant at an editing position creating a stop codon in the AGFG isoform 4 mRNA with a corresponding slight reduction of editing efficiency in the S-4TD mutant. Intriguingly, the improved editing of the S-7TN variant may not only be due to the intended matching change with an A in position -10 but also to the concomitantly improved fit for a preferred cytidine directly upstream in position −11.

A somewhat surprising collateral result of our extensive off-target analyses are the overall numbers of off-targets, which are strongly decreased to less than 350 for the S7-TN mutant but significantly increased to >2200 for the S-4TD mutant (Supplementary Table S4).

To identify differential contributions by the nine PPRs of PPR56 that likely participate in target selection following the established PPR-RNA code, we scored matches, transitions and transversion mismatches separately for the three off-target data sets of the native protein and the two mutants investigated here (Supplementary Figure S4). Characteristically, very similar patterns emerge for all three data sets with strong selection exerted by PPRs S2-1, P2-3, S-4, S-7, P-9 and S-10 and significantly less matches at PPRs P-6 and the most amino-terminal PPRs P-12 and S-13.

Finally, we analyzed the correlation of RNA editing efficiencies and the respective matching scores as defined for the conceptual fits between PPRs at the off-target sequences. A general trend for higher RNA editing efficiencies with improved matches can expectedly be seen, but the overall correlation is only moderate (Supplementary Figure S5). Notably, wide spectra of different RNA editing efficiencies are seen for off-targets of the same matching score. Neither the lowest nor best conceptual matches correlate with particularly low or highly efficient editing at the respective off-targets. In average, slightly higher RNA editing efficiencies are seen for off-targets located in non-coding versus coding regions (Supplementary Figure S6).

## DISCUSSION

### Plant C-to-U RNA editing: why restricted to the organelles?

As of today, and despite ca. 100 meantime functionally characterized plant C-to-U RNA editing factors (48,56), there is no known example of a plant PPR protein acting as a C-to-U RNA editing factor on a target in the nucleo-cytosolic environment and, in fact, no clear evidence for RNA editing of nuclear transcripts in plants at all. On the other hand, there is at least one organelle PPR-type RNA editing factor simultaneously operating on targets in both mitochondria and chloroplasts (57,58). One possible explanation for the lack of plant nucleo-cytosolic C-to-U RNA editing could have been that the post-translational import of the proteins through the membrane envelopes of the endosymbiotic organelles would have been a prerequisite for proper folding and function of PPR-type editing factors. The results presented here clearly argue against this. Not only do the two RNA editing factors investigated, PPR56 and PPR65, show unequivocal functionality in the nucleo-cytosolic environment, but even more so, they faithfully perform RNA editing in the evolutionary distant system of a metazoan cell setup.

### Plant PPR proteins in heterologous environments and the power of off-target analyses

The present study on heterologous expression of the plant RNA editing factors PPR56 and PPR65 in human cell environments has corroborated and significantly extended what emerged from the earlier study on heterologous expression in *E. coli* (28). PPR56 not only yields superior editing efficiencies over PPR65 but also features a high number of off-targets in the heterologous eukaryotic system that are helpful to further characterize PPR-RNA binding features. Given the manifold larger transcriptome in the human cell environment than in the bacterium, a much larger data set can be expected, and was indeed obtained, for evaluation. We have focused on off-target analyses of PPR56 given that the EYFP-PPR56 construct yielded particularly high RNA editing rates and was directly amenable to sorting of the transfected IMR-90 cells via FACS. Nevertheless, we also used cells expressing HA-His_6_-MBP-PPR65 constructs (Figure 5) that were co-transfected with ‘pure’ EYFP encoding plasmids for sorting purposes in a preliminary approach to also determine off-targets of PPR65. To compensate for the inferior sorting approach, double amounts of RNA-Seq raw data were analyzed for this PPR65 transcriptome analysis. Notably, editing of its delivered *ccmFC* target did not exceed 20%, pointing to a lower detection of PPR65 editing capacity using this approach. Accordingly, the results are not immediately comparable to the PPR56 dataset, but four of the seven identified PPR65 off-targets matched expectations for the fit of its PPRs to RNA sequences well (Supplementary Table S5). However, with this low number of detected off-targets, PPR65 yielded much less than PPR56 and, with the above caveat on different sorting approaches and limited editing observed for PPR65 in the tested setup, this would be in accordance with the surprisingly few off-targets previously observed in *E. coli* (28). On the one hand, this different behavior of the two editing factors may well be caused by differential and stronger sequence selectivity exerted not only by the P- and S-type PPRs alone, but also by the respective L-type PPRs and the E1/E2 and DYW domains, which are meantime known to contribute to target selection in a presently not understood way (53–55,59). However, taking into account that we also observed dramatically reduced or significantly increased overall numbers of off-targets upon the single amino acid exchanges in PPR56 (PPR-7TD > TN and PPR-4TN > TD, respectively), we conclude that even such small changes may have significant overall impacts on PPR-type editing factor functionality, possibly reducing (or enhancing) structural flexibility for binding to RNA targets or having an impact on the thermodynamics of the protein-RNA interactions.

Evidently, targeting of PLS-type proteins involves more than just the matches of P- and S-type PPRs to RNA nucleotides according to the ‘core’ PPR-RNA binding code (24–26). Altogether, our extensive analysis of PPR56 off-targets makes several clear points against a simple co-linearity of PPRs and their RNA targets. Most significantly, the site-directed mutagenesis of single amino acids in two PPRs yielded impressive shifts in the off-target spectrum largely as expected, but evidently does not only affect the nucleotide juxtaposed with the corresponding PPR but also the neighboring, predominantly upstream, positions (Figure 8). Even more so, the unexpected non-canonical preference of PPR P-6ND for guanosine instead of the expected uridine in PPR56 appears to be even enhanced by mutation of the upstream PPR S-7TD > TN (Figure 8). Notable additional elements evidently contributing to positional nucleotide preferences in the target RNA are selectivity also influenced by L-type PPRs (28,59) and the evident preferences in positions -3 to -1 upstream of editing sites as documented in our off-target studies and likely due to the specific makeup of the E and DYW domains, in full accord with earlier reports (53–55).

The RNA-binding properties of PLS-type editing factors likely differ from those of P-type PPR proteins. The former supposedly bind to their targets to allow for C-to-U base conversion and disassociate for translation of the affected transcript region. The latter likely bind more stably in 5’- or 3’-UTRs to define transcript ends for nucleolytic processing and stabilization of RNAs (60–62). Nevertheless, the large set of *in vivo* off-targets of PPR56 and its mutant versions mirrors what has previously been explored for P-type PPR10, an RNA-binding and stabilization factor in plant chloroplasts (63) and artificial PPR proteins built from consensus scaffolds (64). The *in vitro* ‘bind-n-seq’ experiments using pools of target variants largely showed binding affinities in full accordance with the PPR-RNA code, but also revealed features that are not explained by the code alone. This includes conserved nucleotides in positions outside of PPR10-RNA contacts previously defined in structural analyses (65), which likely have an impact on the RNA structure important for other nucleotide-PPR interactions. An emerging difference between P-type and PLS-type PPR arrays confirmed with this study may be that N-terminal PPRs are contributing less significantly to binding their targets in the latter (28) whereas the C-terminal PPRs are less relevant in the former (64).

Although the large sets of identified off-targets argue for freely diffusing RNA editing factors in the cytosol, our experimental setup may generally favor the delivered native targets located on the same transcript behind their respective coding regions. First evidence in that direction comes from a very recent study indeed finding somewhat lower RNA editing efficiencies when modifying the previously established bacterial assay system (28) by providing the RNA editing targets on a separate second vector (66).

As discussed previously (34,50,63,64), RNA secondary structures may also contribute to target identification and RNA editing by PPR proteins beyond primary PPR-RNA interactions. In how far such RNA structures may contribute to off-target selection by PPR56 or its two mutants can presently not be answered for lack of a comparative setup and since reasonable secondary structure predictions are imprecise for the full-length transcripts of the human cell transcriptome. A preference for stretches of uridines upstream of editing sites with their alternative, and moderately weak, binding to both purines may play a role why editing factors like PPR56 (see Fig. 1) feature particularly large sets of off-targets.

### Biotech approaches—manipulating RNA with PPR proteins?

Their capacity for site-specific RNA binding has raised speculations to use PPR proteins for specific manipulation of RNA targets (67–70). These studies have been based on variations of natural PPR proteins (71,72) or alternatively used synthetic PPR proteins based on consensus profiles of the widely distributed P-type PPRs (64,68,73–77). A very recent new approach has focused on particular S-type PPRs (78) modeling the binding properties of the widely studied natural CLB19 editing factor (50,78–85). This work extended and complemented an earlier attempt using a synthetic PLS-type PPR array as a surrogate for the PPR array of CLB19, which, however, turned out to require MORF2, a ‘Multiple Organelle RNA editing Factor’, as a necessary co-factor for obtaining moderate RNA editing efficiency (86). Using the *E. coli* assay setup, the synthetic PLS-type PPR protein did not cause an off-target editing, while the artificial S-type approach resulted in 50% of RNA editing at its natural CLB19 *rpoA* target and only weakly affected one single off-target (*tufB*) in *E. coli* (78).

An important issue to be considered, however, may be the variable RNA editing efficiencies observed in the different cell lines that we have explored here. The evidently different degrees of RNA editing in the four human cell lines (Figure 5) may indicate that the introduced heterologous RNA editing is also dependent on the genetic setup of the respective host cell environment, and it is likely that similar or even larger differences would be seen in different tissues or other cell types. These findings may implicate that target recognition could be influenced by the respective differentiation state of a eukaryotic cell. Certainly, cell-type dependent RNA editing through PPR-type editing factors may at the same time be an advantage, very much like their independence from co-delivered sgRNAs needed for CRISPR-based approaches (see below), which could also allow for straightforward RNA editing after import into mitochondria.

Along the same lines, the strength of expression of the respective editing tool may play an important role. Notably, more than 4,000 off-targets have been found upon APOBEC3A overexpression (87), which is certainly a critical issues given the involvement of APOBECs in processes including cancer development (16). Intriguingly, the complementation of many plant PPR editing factor KO lines with constructs driven from strong promoters have re-established proper RNA editing functions without causing evident collateral effects (50,54,88,89), although natural expression levels of RNA editing factors are very low *in planta* (90,91).

### CRISPR- versus PPR-based approaches and general considerations

The prime alternative to PPR-based approaches for targeted transcriptome editing uses CRISPR-Cas13 variants with engineered nucleobase deaminases specifically targeted to RNAs by guiding RNAs (92–94). An important recent development is ‘CURE’ (for C-U RNA Editor) for cytidine-specific deamination (95) as opposed to the earlier ‘RESCUE-S’ approach for adenine and cytidine deamination (94) with a S375A mutation in ADAR2 to suppress off-targets.

Any approach targeting specific RNAs or transcriptomes for editing should take possible collateral effects also on the genome into account. The capacity of binding not only to RNA but also to single-stranded DNA is evident for designer P-type PPR proteins (96). Hence, it may be advisable that all promising engineering approaches should finally be thoroughly inspected for variable outcomes not only in different cell types of a target organisms but ideally at the transcriptomic and genomic level alike.

Whatever biotechnological developments may prove to be the most efficient and/or most specific approach, extensive and careful off-target analysis will be mandatory. The DYW domain cytidine deaminase could be promising also for fusion to Cas proteins in CRISPR-based approaches. Controlled cytidine deamination by the DYW domain might be enabled by the just recently identified autoinhibited ground state of the DYW domain caused by a conserved gating domain, which regulates the active site (27).

The successful functional expression of plant C-to-U RNA editing factors in human cells reported here significantly complements and extends the insights gained from the previously established *E. coli* assay setup. One notable case in point are characteristic differences found for the eukaryotic versus the prokaryotic setup for PPR56 upon mutation of key PPR residues (Figure 7). It remains to be seen whether the notoriously difficult heterologous expression of PPR proteins in *E. coli* may even prove to be generally superior in eukaryotic systems. In any case, the much larger endogenous background transcriptome of the eukaryotic cells will allow for much larger off-target data sets that are helpful to understand RNA binding properties and editing efficiencies. Our observations of collateral changes in the off-target profiles also for positions next to the ones directly juxtaposed with specifically mutated PPRs (Figure 8) are prime examples along those lines. If particular plant C-to-U RNA editing factors may be optimized to only address selected targets rather than a high number of off-targets and thereby becoming suitable for precise manipulation remains to be investigated.

## DATA AVAILABILITY

FastQC analyses is available via www.bioinformatics.babraham.ac.uk/projects/fastqc. BBTools suite is an open-source initiative and accessible via sourceforge.net/projects/bbmap. NCBI human GRCH38 RefSeq Transcripts can be downloaded via www.ncbi.nlm.nih.gov/genome/guide/human. R v4.1.1 is available at https://www.r-project.org and RStudio v1.2.5033 at www.rstudio.com. The TargetScan tool of PREPACT is an online tool accessible via http://www.prepact.de/prepact-main.php. The RNAseq data have been deposited in the SRA archive as BioProject PRJNA832818.

## Supplementary Material

gkac752_Supplemental_File
