# Supplementary material for: Plant mitochondrial RNA editing factors can perform targeted C-to-U editing of nuclear transcripts in human cells
Source: Nucleic Acids Res. 2022 Sep 15;50(17):9966–83. doi: 10.1093/nar/gkac752 (PMC9508816; doi:10.1093/nar/gkac752)
Supplement: gkac752_Supplemental_File [file gkac752_supplemental_file.pdf]

Plant mitochondrial RNA editing factors can perform targeted C-to-U editing of nuclear transcripts in human cells

Elena Lesch[1], Maximilian T. Schilling[2], Sarah Brenner[1], Yingying Yang[1], Oliver J. Gruss[2],
Volker Knoop[1*] and Mareike Schallenberg-Rüdinger[1*]

[1] IZMB - Institut für Zelluläre und Molekulare Botanik, Abteilung Molekulare Evolution, Universität Bonn, Kirschallee 1, D-53115 Bonn, Germany

[2] Institut für Genetik, Abteilung Zellteilung,
Universität Bonn, Karlrobert-Kreiten-Str. 13, D-53115 Bonn, Germany

* To whom correspondence should be addressed. Mareike Schallenberg-Rüdinger: Tel: +49-(0)-228-736464; Fax: +49-(0)-228-73-6467; Email: mrueding@uni-bonn.de

Correspondence may also be addressed to Volker Knoop: Tel: +49-(0)-228-73-6466; Fax: +49-(0)-228-73-6467; Email: volker.knoop@uni-bonn.de

Supplementary Material

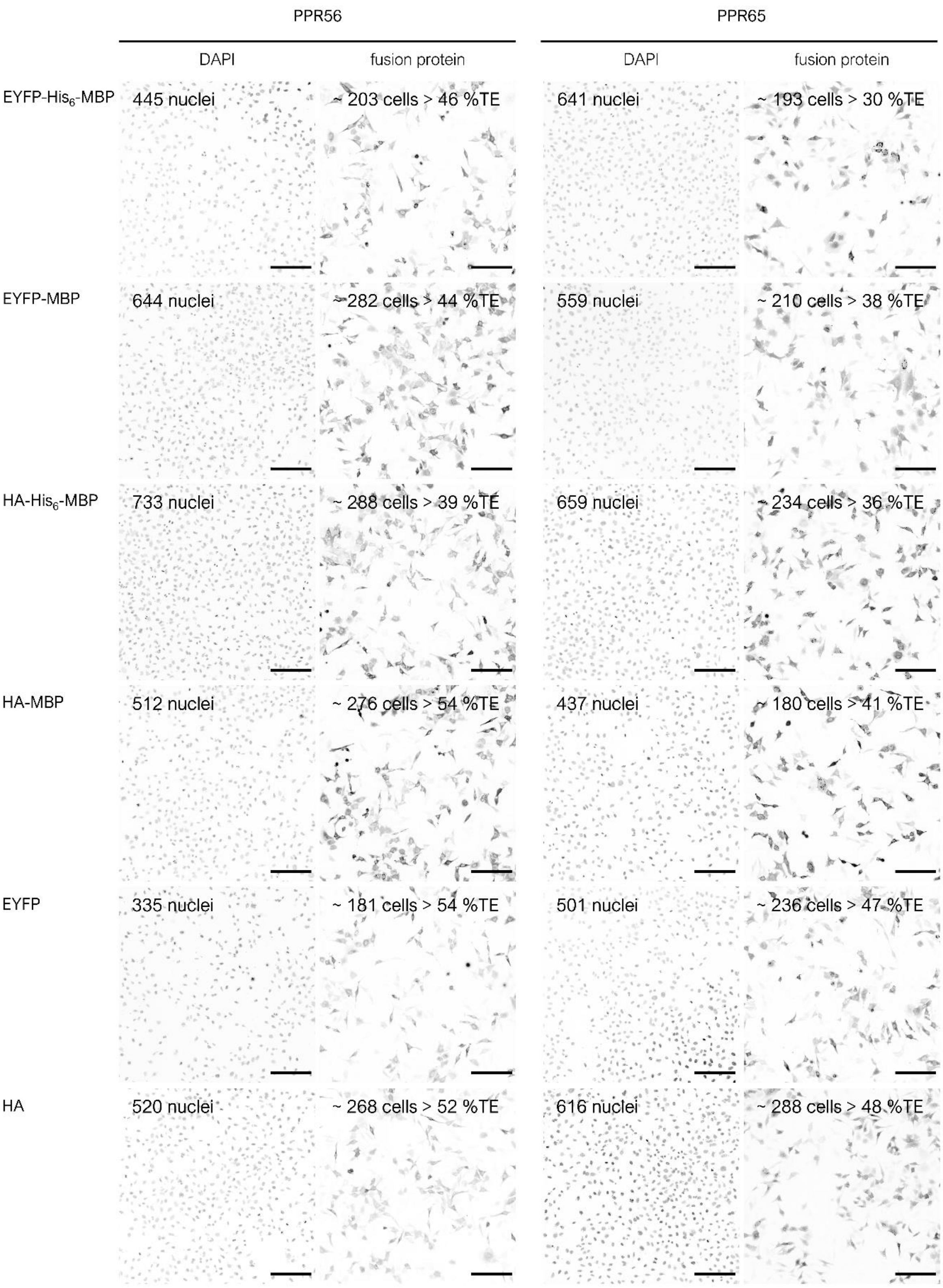
PPR56
PPR65
DAPI
fusion protein
DAPI
fusion protein
EYFP-His$_6$-MBP
445 nuclei
~ 203 cells > 46 %TE
641 nuclei
~ 193 cells > 30 %TE
EYFP-MBP
644 nuclei
~ 282 cells > 44 %TE
559 nuclei
~ 210 cells > 38 %TE
HA-His$_6$-MBP
733 nuclei
~ 288 cells > 39 %TE
659 nuclei
~ 234 cells > 36 %TE
HA-MBP
512 nuclei
~ 276 cells > 54 %TE
437 nuclei
~ 180 cells > 41 %TE
EYFP
335 nuclei
~ 181 cells > 54 %TE
501 nuclei
~ 236 cells > 47 %TE
HA
520 nuclei
~ 268 cells > 52 %TE
616 nuclei
~ 288 cells > 48 %TE

Supplementary Figure S1. Transfection rate calculation via HA and EYFP immunostained fusion proteins per DAPI stained nuclei.

After fixation and immunostaining of HeLa cells, expressing N-terminally tagged PPR56 or PPR65, 960.93x960.93 µm overview images were taken with a Nikon Eclipse Ti2 system equipped with a PlanFluor 40x Oil objective (NA 1.3) and 1.5x Zoom using the NIS-Elements AR software. In ImageJ/Fiji version 1.53c for Windows, the color channels were split. The DAPI image was transformed into a B&W binary and close nuclei were separated using the 'Watershed' and counted using the 'Analyze Particles' function with a size threshold of 1000 μm^2. Alexa594 fluorescence was displayed as inverted grayscale for counting fusion protein expressing cell. Transfection efficiencies (%TE) were defined as the number of fusion proteins expressing cells per total nuclei. Scale bars = 200 µm.

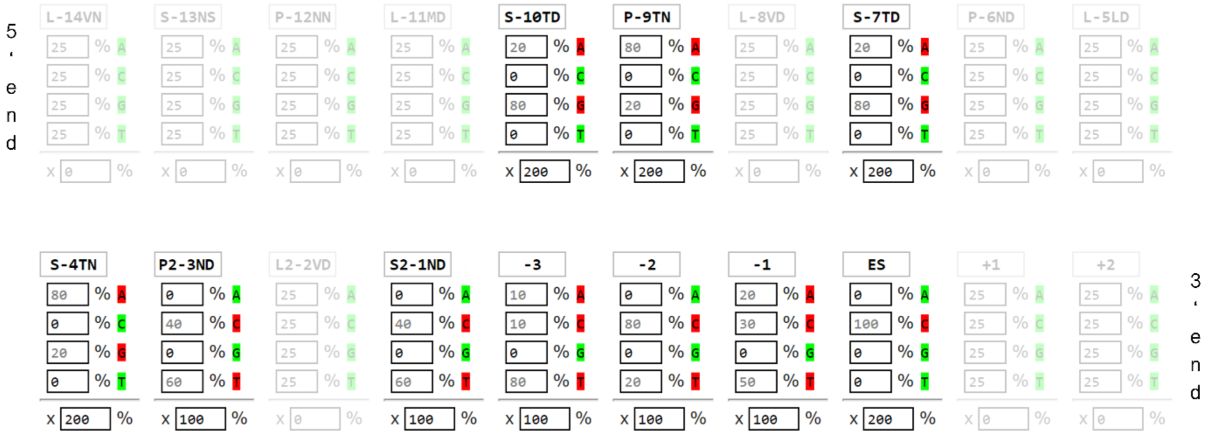

Supplementary Figure S2. Weight matrix used for scanning the custom IMR-90 transcriptome for putative PPR56 editing sites by the TargetScan tool of PREPACT (1).

Each column corresponds to one nucleotide position and is labelled either (i) with type, position counting from S2, and fifth and last amino acid of the corresponding PPR or (ii) with the position relative to the editing site (ES). Nucleotide distributions were set according to the published PPR-RNA binding code (2) and specific knowledge about PPR56 off-target sequence conservation (3). Positional weighs are shown below. Positions with no given weight (0%) and no influence on target selection are shaded in light grey.

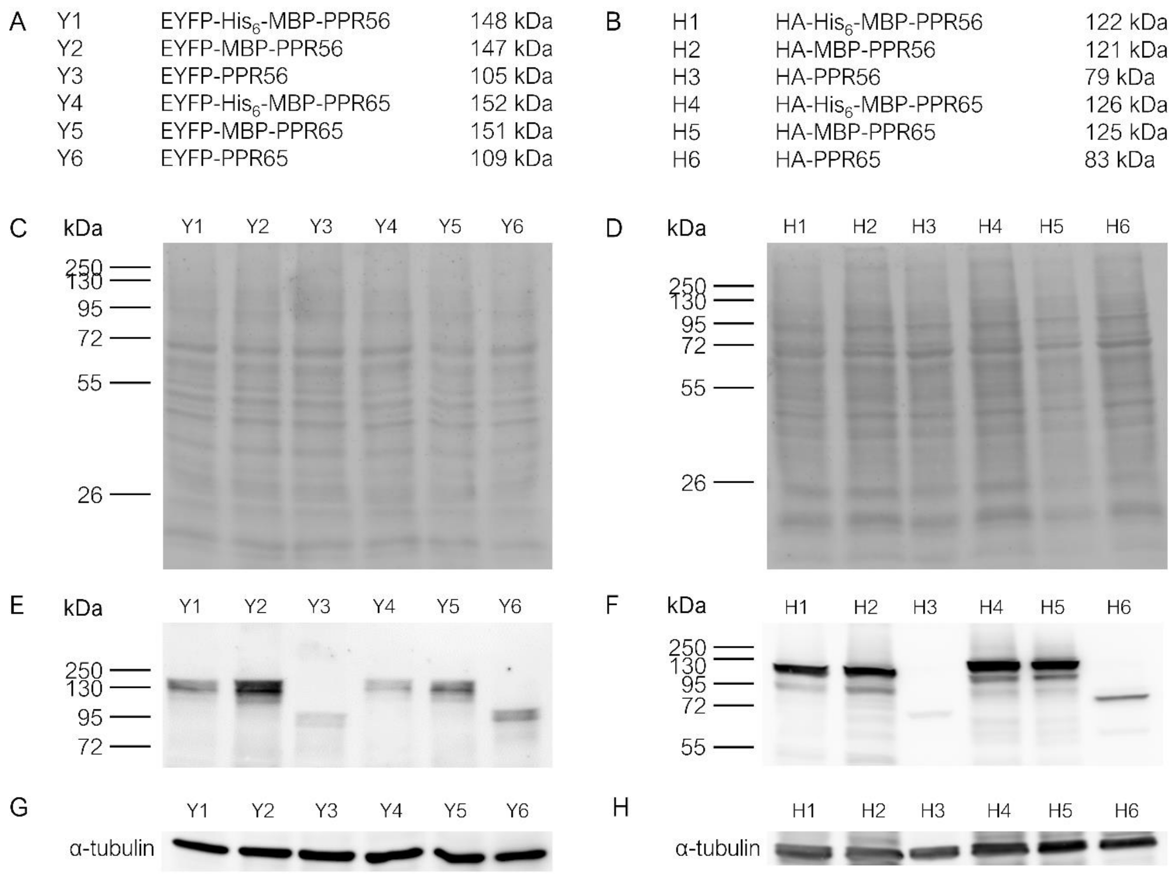

Supplementary Figure S3. Western Blot analyses of fusion protein expression in HeLa cells.

A, B: Investigated fusion proteins and corresponding molecular weights in kDa (calculated with BCCM Genecorner, https://www.genecorner.ugent.be/protein_mw.html). C, D: Total protein stained with Ponceau. E: Immunodetection of EYFP-tagged PPR proteins with α-GFP (mouse, Roth) and peroxidase labeled secondary antibody (Jackson ImmunoResearch). F: Immunodetection of HA-tagged PPR proteins with α-HA (rabbit, abcam ab9110) and peroxidase labeled secondary antibody (Jackson ImmunoResearch). G, H: Immunodetection of α-tubulin with α- α-tubulin (rabbit, Cell Signaling cat. #2125) and peroxidase labeled secondary antibody (Jackson ImmunoResearch) as loading control.

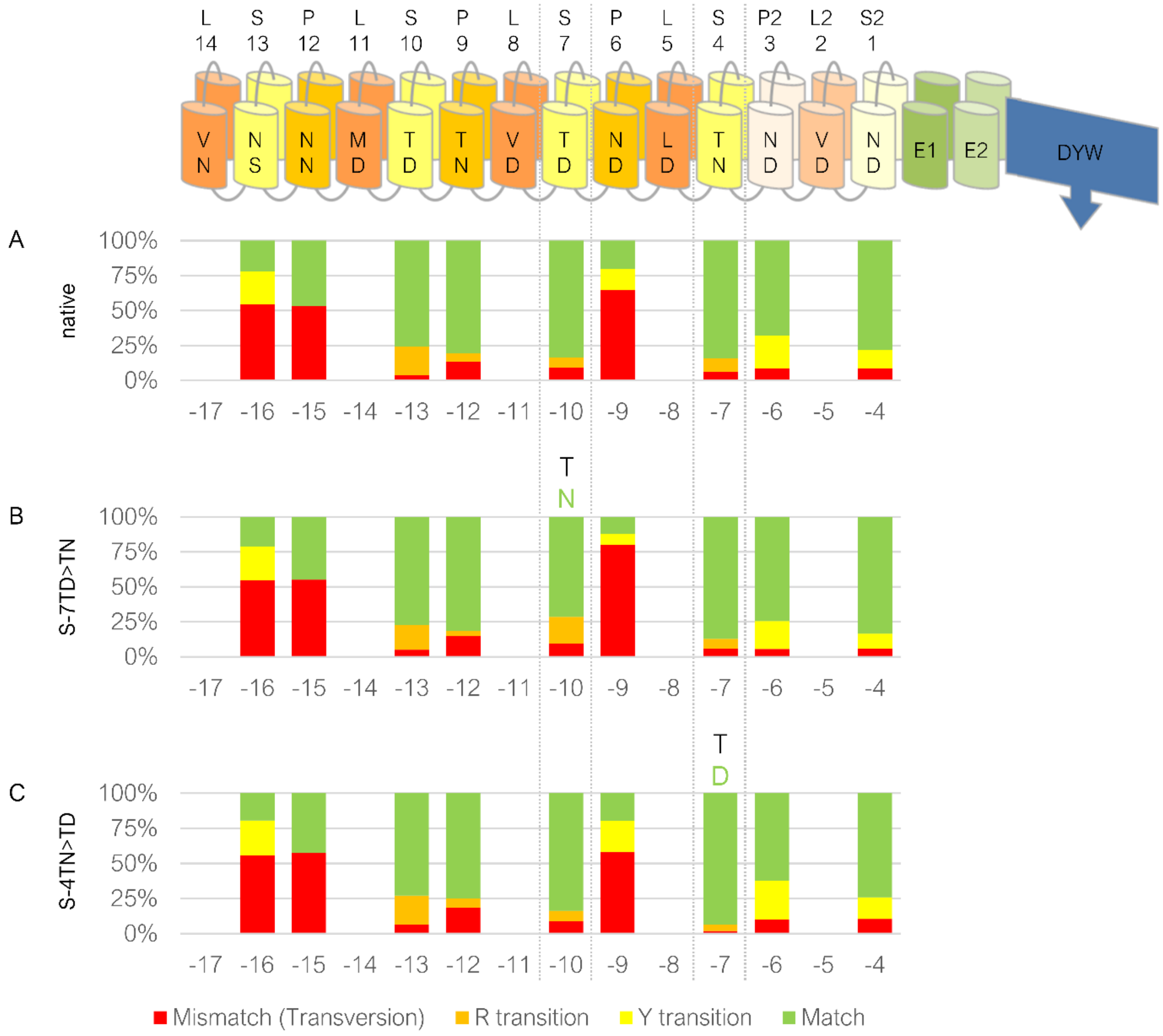

Supplementary Figure S4. Matches of individual PPRs and their corresponding nucleotides for the off-target data sets of PPR56 and the two investigated mutants S-4TN-TD and S-7TD-TN

The bar charts show the relative amounts of conceptual matches between PPRs and their target nucleotides (green), pyrimidine and purine transitions (yellow and orange, respectively) and transversion mismatches (red) at the respective positions observed for the off-targets determined for the native PPR56 protein (A) and the two investigated mutants (B and C).

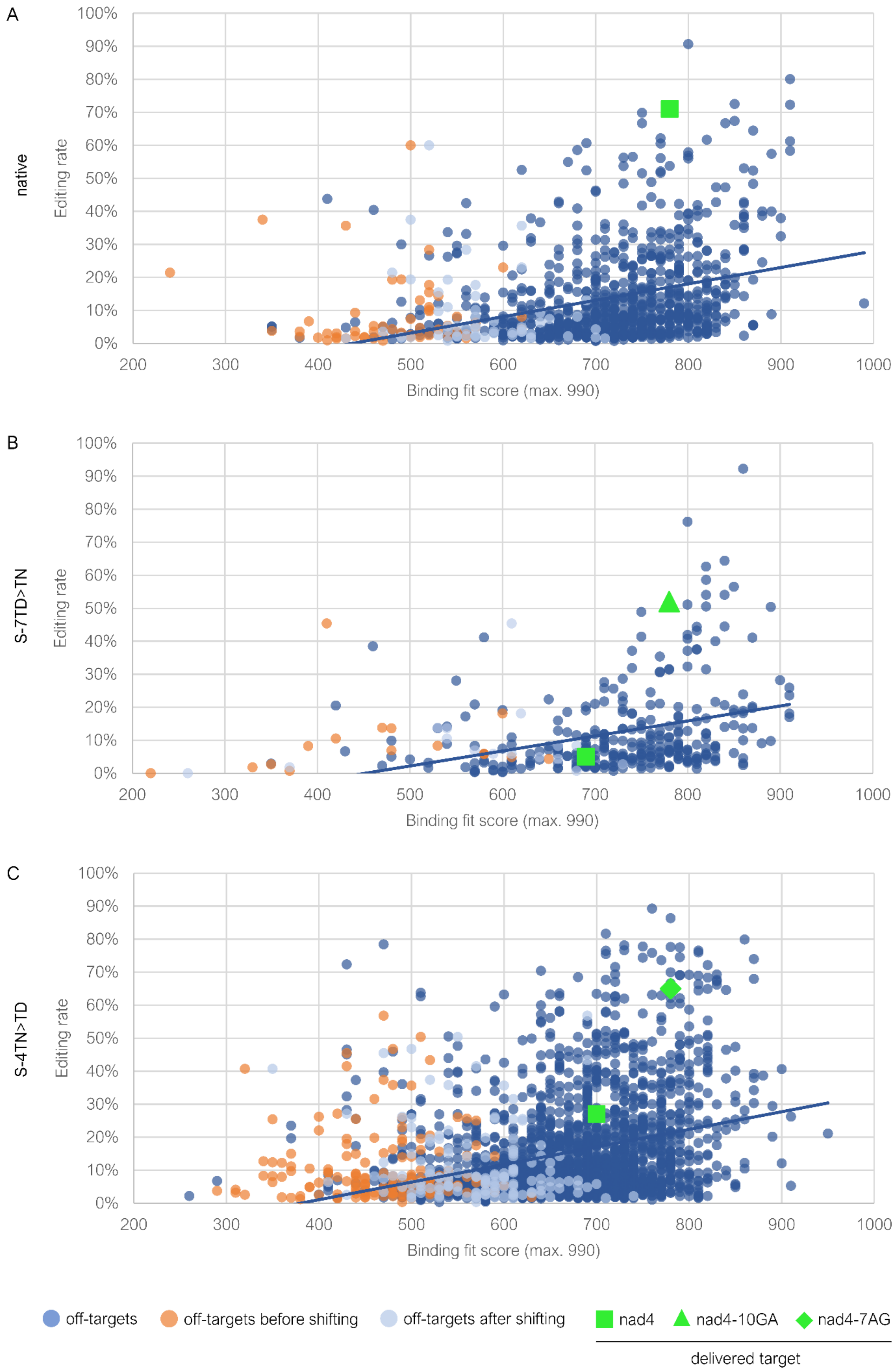

A
native
Editing rate
100%
90%
80%
70%
60%
50%
40%
30%
20%
10%
0%
200
300
400
500
600
700
800
900
1000
Binding fit score (max. 990)
B
S-7TD>TN
Editing rate
Binding fit score (max. 990)
C
S-4TN>TD
Editing rate
Binding fit score (max. 990)
off-targets
off-targets before shifting
off-targets after shifting
nad4
nad4-10GA
nad4-7AG
delivered target

Supplementary Figure S5. Scatter Plots correlating RNA editing efficiencies with the conceptual overall binding score at the off-targets for wild-type and the two mutant variants of PPR56.

Scatter plot of RNA editing efficiencies on the y-axis displayed in relation to conceptual matching scores (as described in figure 9) on the x-axis for the off-targets (dark blue dots) determined for the native PPR56 protein (A) and the two investigated mutants (B and C). Better matching scores are observed for ca. 5-6 % of off-targets (orange dots; 59 of 906 for PPR56, 16 of 323 for the S-7TN mutant and 147 of 2265 for the S-4TD mutant, respectively) after one- or two-nucleotide-shifts (light blue dots), away from the canonical juxtaposition of the terminal S2-type PPR with position -4 upstream of the editing sites. Editing at the co-delivered targets (green symbols) are added for reference.

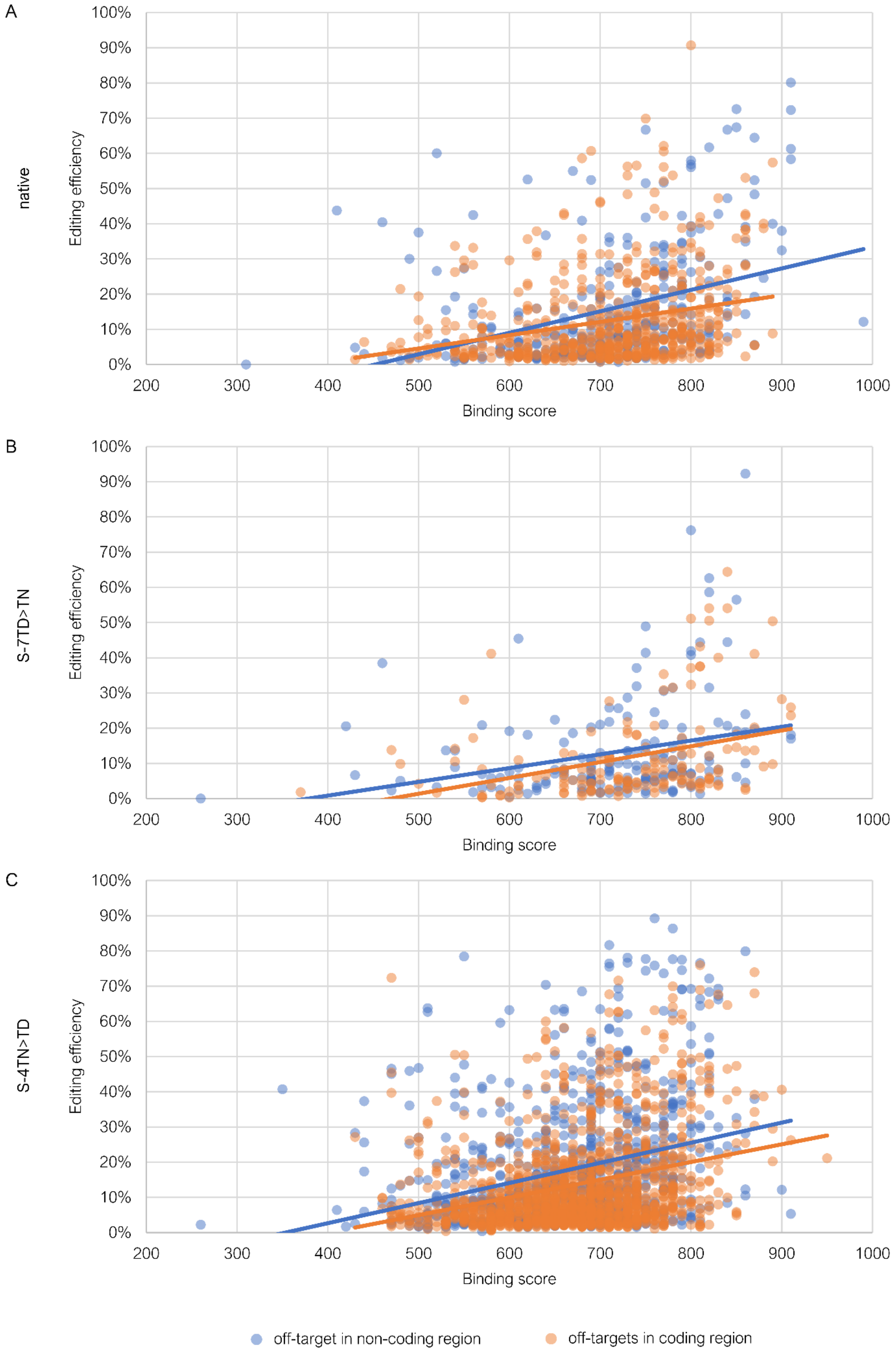

A
native
B
S-7TD>TN
C
S-4TN>TD
Editing efficiency
Binding score
100%
90%
80%
70%
60%
50%
40%
30%
20%
10%
0%
200
300
400
500
600
700
800
900
1000
off-target in non-coding region
off-targets in coding region

Supplementary Figure S6. Scatter Plots distinguishing off-targets occurring in coding vs. non-coding transcript regions.

Off-targets as analyzed in supplementary figure S5 (after nucleotide shifting for improved matching scores where applicable) classified by their occurrence in coding sequences (orange) or in non-coding regions (blue).

Supplementary Table S1

Oligonucleotides/ gBlocks used in this study.

#	Name	Purpose/ target	Sequence (5’-3’)
P1	PPR56_P9_R	Cloning of PPR56 S motif mutant versions	GTTTGAATCCTTCCTGTTCC
P2	PPR56_P6_F	Cloning of PPR56 S motif mutant versions	GGCATGAAGCATATGATCTG
P3	PPR56_P6_R	Cloning of PPR56 S motif mutant versions	CAGATCATATGCTTCATGCC
P4	PPR56_P3_F	Cloning of PPR56 S motif mutant versions	GCTTGGCACAACATGGTC
P5	Gw_PPR56_-n_for	Cloning PPR into petG41K	GGGGACAAGTTTGTACAAAAAAGCAGGCTTAGAGAATCTTTATTTTCAGGGCGTGGTTCTGATGAACCGTC
P6	Gw_PPR56_-n_rev	Cloning PPR into petG41K	GGGGACCACTTTGTACAAGAAAGCTGGGTCTCACCAGTAATCACCACATG
P7	175641Ppfor	Cloning PPR into petG41K	GGGGACAAGTTTGTACAAAAAAGCAGGCTTAGAGAATCTTTATTTTCAGGGCTCTCACGATCAGATGCCAGGG
P8	175641Ppcomprev	Cloning PPR into petG41K	GGGGACCACTTTGTACAAGAAAGCTGGGTCTCACCAAAAATCCCCACAAGAACATAC
P9	EGFP_MBP_for_ScaI	Cloning PPR-target expression cassette	GCCAGTACTCCATGAAAATCGAAGAAGG
P10	EGFP_TEV_for_ScaI	Cloning PPR-target expression cassette	GCCAGTACTTAGAGAATCTTTATTTTCAGG
P11	EGFP_tT7_rev_ApaI	Cloning PPR-target expression cassette	ATGGGCCCTAGTTATTGCTCAGCGGTGG
P12	CMV_His_for_MscI	Cloning PPR-target expression cassette	ATTATATGGCCAAACATCACCATCACCATCACCCC
P13	CMV_MBP_for_MscI	Cloning PPR-target expression cassette	ATTATATGGCCACCATGAAAATCGAAGAAGG
P14	CMV_TEV_for_MscI	Cloning PPR-target expression cassette	ACTATATGGCCATAGAGAATCTTTATTTTCAGG
P15	CMV_tT7_rev_NotI	Cloning PPR-target expression cassette	ATTATAGCGGCCGCTAGTTATTGCTCAGCGGTGG
P16	pCMV-HA_seq_for	Cloning PPR-target expression cassette	CGGAATTGTACCCGCGGGCC
P17	pCMV-HA_seq_rev	Cloning PPR-target expression cassette	ATTCTAGTTGTGGTTTGTCC
P18	EGFP_His_for_ScaI_MOD	Cloning PPR-target expression cassette	GCCAGTACTTACATCACCATCACCATCACCCC
P19	PPR56_4-7ag_F	Cloning target	AAATATCAATTTTTATATAGgtatagacggtGtctcttcATTTTGG
P20	PPR56_4-7ag_R	Cloning target	CGCGCCAAAATGAAGAGACACCGTCTATACCTATATAAAAATTGATATTT
P21	PPR56_4-10ga_F	Cloning target	AAATATCAATTTTTATATAGgtatagacAgtatctcttcATTTTGG
P22	PPR56_4-10ga_R	Cloning target	CGCGCCAAAATGAAGAGATACTGTCTATACCTATATAAAAATTGATATTT
P23	PPR56_nad4_F	Cloning target	TCAAACATCAATTTTTATATAGgtatagacggtatctcttcATTTT
P24	PPR56_nad4_R	Cloning target	CGCGAAAATGAAGAGATACCGTCTATACCTATATAAAAATTGATGTTTGA
P25	HindIIIccmFC_for	Cloning target	agcttATACTATTTCAATGGTTGGTAAGTAGAGATGTTCCCACAtt
P26	Bsp119iccmFC_rev	Cloning target	CGAATGTGGGAACATCTCTACTTACCAACCATTGAAATAGTATA
P27	petG_30K40Kseqrev	Editing analysis/ Sequencing	GGTTATGCTAGTTATTGCTCAGC
P28	PPR56_CTDAH_R	PPR mutagenesis DYW domain	CTTAGTTGCTCCATGAGCATCGGTGCACACCCG
P29	PPR56_CTDAH_F	PPR mutagenesis DYW domain	CGGGTGTGCACCGATGCTCATGGAGCAACTAAG
P30	PPR65_HSARLAfor	PPR mutagenesis DYW domain	CTTTTCTCTCACAGTGCGAGGCTGGCGATCAC
P31	PPR65_HSARLArev	PPR mutagenesis DYW domain	GTGATCGCCAGCCTCGCACTGTGAGAGAAAAG
P32	PPR65_CGDAH_for	PPR mutagenesis DYW domain	CGAGTGTGTGGGGATGCTCATACGGCGACGAAG
P33	PPR65_CGDAH_rev	PPR mutagenesis DYW domain	CTTCGTCGCCGTATGAGCATCCCCACACACTCG
P34	PPR56_HSERLA_F	PPR mutagenesis DYW domain	CTCACAGTGAGaggTTGGCAATTGTG
P35	PPR56_HSERLA_R	PPR mutagenesis DYW domain	CACAATTGCCAACCTCTCACTGTGAG
P36	PPR56_S4TNTD_for	PPR mutagenesis S4 motif (gBlock IDT)	GTGGGCATGAAGCATATGATCTGTTTCTTCAGATGCAGACAGAGGGCTGTAAGCCAGATGCAATCATGTTTTTGAGCATCTTGAATGCATGTGCAAGTGCAGGGGCATTGGAGTGGGTGAAGAAGATTCACAGGCATGCCTTGGATTCAGGGCTTGAGGTTGATGTGCGTGTGGGCACTGCACTTGTTCACATGTACTCAAAGAGTGGTAGCATTGATGATGCGCGAGTGGTGTTTGACAGAATGAAGGTGCGCgATGTTGTATCATGGAATGCGATGATTTCTGGCTTGGCACAACATGGTCTTGGGCAGGACGCTTTG
P37	PPR56_S-7TDTN_for	PPR mutagenesis S7 motif (gBlock IDT)	TGGAACAGGAAGGATTCAAACCAAATGCAATCACTTATGTGAGCATCTTGAACGCATGTGCAAGTGAGGGGGCATTGAAGTGGGTGAAGAGGGTTCACAGGCATGCCTTGGATGCAGGGCTTGAGTTGGATGTGCGTGTGGGCACTGCACTTGTTCAAATGTACGCAAAGAGTGGTAGCATTGATGATGCACGAGTGGTGTTTGACAGAATGAAGGTGCGTaATGTTGTATCATGGAATGTGATGATAGGCGCATTTGCAGAACATGGGCGTGGGCATGAAGCATATGATCTGTTTCTTC
P38	PPR56_int_for	PPR56 internal/ Sequencing	TTGAATGCATGTGCAAGTGC
P39	PPR65_intseq_rev	PPR65 internal/ Sequencing	CCAGACTTGCGTCATCCATGC
P40	175641KOfor	PPR65 internal/ Sequencing	ATGGAAATTGTTGAAGCTGGACTGG

P41	PPR65_PAbox_rev	PPR65 internal/ Sequencing	CTTCAATCCAGCTCTGCGCCGGCTCCTTGACAACATCC
P42	PPR65edit_f	PPR65 internal/ Sequencing	TATCTACGCTGCTGCTGGCC
P43	pEGFP-C3_seq_for	Sequencing	GATCACTCTCGGCATGGACG
P44	pEGFP-CR_seq_rev	Sequencing	CTCTACAAATGTGGTATGGC
P45	mbppetG40Kseqfor	Sequencing	GAT GTC CGC TTT CTG GTA TGC
P46	TEVrev	Sequencing	CCTGAAAATAAAGATTCTC
P47	EYFPfor	Sequencing	CACCTTGACCTACGGCGTGC

Supplementary Table S2

Results of all individual RNA editing experiments in the heterologous human cell and *E. coli* setups. The experimental conditions (type and number of cells seeded for transfection [human cells]/ of cells induced by addition of 0.4 mM isopropyl β-D-1-thiogalactopyranoside (IPTG) [*E. coli*], incubation time after transfection [human cells]/ after induction [*E. coli*], media and zinc supplementation) and C-U RNA editing rate of each individual replicate is listed.

#	Organism	Experiment	Cell line	Construct	Incubation	Cell number	Media	Zinc	Editing	Study
1	Human	Cell line	HEK-293	HA-His_6-MBP-PPR56+nad4_native	20h	$2x10^5$	DMEM+FCS+Penstrep	25µM	46%	This study
2	Human	Cell line	HEK-293	HA-His_6-MBP-PPR56+nad4_native	20h	$2x10^5$	DMEM+FCS+Penstrep	25µM	57%	This study
3	Human	Cell line	HEK-293	HA-His_6-MBP-PPR56+nad4_native	20h	$2x10^5$	DMEM+FCS+Penstrep	25µM	50%	This study
4	Human	Cell line	HEK-293	HA-His_6-MBP-PPR65+ccmFC_native	20h	$2x10^5$	DMEM+FCS+Penstrep	25µM	34%	This study
5	Human	Cell line	HEK-293	HA-His_6-MBP-PPR65+ccmFC_native	20h	$2x10^5$	DMEM+FCS+Penstrep	25µM	39%	This study
6	Human	Cell line	HEK-293	HA-His_6-MBP-PPR65+ccmFC_native	20h	$2x10^5$	DMEM+FCS+Penstrep	25µM	36%	This study
7	Human	Cell line	HEK-293	EYFP-PPR56+nad4_native	20h	$2x10^5$	DMEM+FCS+Penstrep	25µM	50%	This study
8	Human	Cell line	HEK-293	EYFP-PPR56+nad4_native	20h	$2x10^5$	DMEM+FCS+Penstrep	25µM	52%	This study
9	Human	Cell line	HEK-293	EYFP-PPR56+nad4_native	20h	$2x10^5$	DMEM+FCS+Penstrep	25µM	55%	This study
10	Human	Cell line	HEK-293	EYFP-PPR65+ccmFC_native	20h	$2x10^5$	DMEM+FCS+Penstrep	25µM	17%	This study
11	Human	Cell line	HEK-293	EYFP-PPR65+ccmFC_native	20h	$2x10^5$	DMEM+FCS+Penstrep	25µM	13%	This study
12	Human	Cell line	HEK-293	EYFP-PPR65+ccmFC_native	20h	$2x10^5$	DMEM+FCS+Penstrep	25µM	13%	This study
13	Human	Cell line	IMR-90	HA-His_6-MBP-PPR56+nad4_native	20h	$1.8x10^5$	MEM+FCS+Penstrep	25µM	59%	This study
14	Human	Cell line	IMR-90	HA-His_6-MBP-PPR56+nad4_native	20h	$1.8x10^5$	MEM+FCS+Penstrep	25µM	60%	This study
15	Human	Cell line	IMR-90	HA-His_6-MBP-PPR56+nad4_native	20h	$1.8x10^5$	MEM+FCS+Penstrep	25µM	61%	This study
16	Human	Cell line	IMR-90	HA-His_6-MBP-PPR65+ccmFC_native	20h	$1.8x10^5$	MEM+FCS+Penstrep	25µM	33%	This study
17	Human	Cell line	IMR-90	HA-His_6-MBP-PPR65+ccmFC_native	20h	$1.8x10^5$	MEM+FCS+Penstrep	25µM	30%	This study
18	Human	Cell line	IMR-90	HA-His_6-MBP-PPR65+ccmFC_native	20h	$1.8x10^5$	MEM+FCS+Penstrep	25µM	30%	This study
19	Human	Cell line	IMR-90	EYFP-PPR56+nad4_native	20h	$1.8x10^5$	MEM+FCS+Penstrep	25µM	69%	This study
20	Human	Cell line	IMR-90	EYFP-PPR56+nad4_native	20h	$1.8x10^5$	MEM+FCS+Penstrep	25µM	73%	This study
21	Human	Cell line	IMR-90	EYFP-PPR56+nad4_native	20h	$1.8x10^5$	MEM+FCS+Penstrep	25µM	73%	This study
22	Human	Cell line	IMR-90	EYFP-PPR65+ccmFC_native	20h	$1.8x10^5$	MEM+FCS+Penstrep	25µM	2%	This study
23	Human	Cell line	IMR-90	EYFP-PPR65+ccmFC_native	20h	$1.8x10^5$	MEM+FCS+Penstrep	25µM	2%	This study
24	Human	Cell line	IMR-90	EYFP-PPR65+ccmFC_native	20h	$1.8x10^5$	MEM+FCS+Penstrep	25µM	0%	This study
25	Human	Cell line	MCF-7	HA-His_6-MBP-PPR56+nad4_native	20h	$2x10^5$	DMEM+FCS+Penstrep	25µM	64%	This study
26	Human	Cell line	MCF-7	HA-His_6-MBP-PPR56+nad4_native	20h	$2x10^5$	DMEM+FCS+Penstrep	25µM	71%	This study
27	Human	Cell line	MCF-7	HA-His_6-MBP-PPR56+nad4_native	20h	$2x10^5$	DMEM+FCS+Penstrep	25µM	74%	This study
28	Human	Cell line	MCF-7	HA-His_6-MBP-PPR65+ccmFC_native	20h	$2x10^5$	DMEM+FCS+Penstrep	25µM	61%	This study
29	Human	Cell line	MCF-7	HA-His_6-MBP-PPR65+ccmFC_native	20h	$2x10^5$	DMEM+FCS+Penstrep	25µM	63%	This study
30	Human	Cell line	MCF-7	HA-His_6-MBP-PPR65+ccmFC_native	20h	$2x10^5$	DMEM+FCS+Penstrep	25µM	63%	This study
31	Human	Cell line	MCF-7	EYFP-PPR56+nad4_native	20h	$2x10^5$	DMEM+FCS+Penstrep	25µM	37%	This study
32	Human	Cell line	MCF-7	EYFP-PPR56+nad4_native	20h	$2x10^5$	DMEM+FCS+Penstrep	25µM	47%	This study
33	Human	Cell line	MCF-7	EYFP-PPR56+nad4_native	20h	$2x10^5$	DMEM+FCS+Penstrep	25µM	44%	This study
34	Human	Cell line	MCF-7	EYFP-PPR65+ccmFC_native	20h	$2x10^5$	DMEM+FCS+Penstrep	25µM	25%	This study

35	Human	Cell line	MCF-7	EYFP-PPR65+ccmFC_native	20h	$2x10^5$	DMEM+FCS+Penstrep	25µM	26%	This study
36	Human	Cell line	MCF-7	EYFP-PPR65+ccmFC_native	20h	$2x10^5$	DMEM+FCS+Penstrep	25µM	26%	This study
37	Human	Cell line/ Construct	HeLa	HA-His_6-MBP-PPR56+nad4_native	20h	$2x10^5$	DMEM+FCS+Penstrep	25µM	55%	This study
38	Human	Cell line/ Construct	HeLa	HA-His_6-MBP-PPR56+nad4_native	20h	$2x10^5$	DMEM+FCS+Penstrep	25µM	67%	This study
39	Human	Cell line/ Construct	HeLa	HA-His_6-MBP-PPR56+nad4_native	20h	$2x10^5$	DMEM+FCS+Penstrep	25µM	56%	This study
40	Human	Cell line/ Construct	HeLa	HA-His_6-MBP-PPR56+nad4_native	20h	$2x10^5$	DMEM+FCS+Penstrep	25µM	52%	This study
41	Human	Cell line/ Construct	HeLa	HA-His_6-MBP-PPR56+nad4_native	20h	$2x10^5$	DMEM+FCS+Penstrep	25µM	65%	This study
42	Human	Cell line/ Construct	HeLa	HA-His_6-MBP-PPR56+nad4_native	20h	$2x10^5$	DMEM+FCS+Penstrep	25µM	60%	This study
43	Human	Cell line/ Construct	HeLa	HA-His_6-MBP-PPR56+nad4_native	20h	$2x10^5$	DMEM+FCS+Penstrep	25µM	52%	This study
44	Human	Cell line/ Construct	HeLa	HA-His_6-MBP-PPR56+nad4_native	20h	$2x10^5$	DMEM+FCS+Penstrep	25µM	56%	This study
45	Human	Cell line/ Construct	HeLa	HA-His_6-MBP-PPR65+ccmFC_native	20h	$2x10^5$	DMEM+FCS+Penstrep	25µM	52%	This study
46	Human	Cell line/ Construct	HeLa	HA-His_6-MBP-PPR65+ccmFC_native	20h	$2x10^5$	DMEM+FCS+Penstrep	25µM	63%	This study
47	Human	Cell line/ Construct	HeLa	HA-His_6-MBP-PPR65+ccmFC_native	20h	$2x10^5$	DMEM+FCS+Penstrep	25µM	62%	This study
48	Human	Cell line/ Construct	HeLa	HA-His_6-MBP-PPR65+ccmFC_native	20h	$2x10^5$	DMEM+FCS+Penstrep	25µM	47%	This study
49	Human	Cell line/ Construct	HeLa	HA-His_6-MBP-PPR65+ccmFC_native	20h	$2x10^5$	DMEM+FCS+Penstrep	25µM	55%	This study
50	Human	Cell line/ Construct	HeLa	HA-His_6-MBP-PPR65+ccmFC_native	20h	$2x10^5$	DMEM+FCS+Penstrep	25µM	57%	This study
51	Human	Cell line/ Construct	HeLa	HA-His_6-MBP-PPR65+ccmFC_native	20h	$2x10^5$	DMEM+FCS+Penstrep	25µM	41%	This study
52	Human	Cell line/ Construct	HeLa	HA-His_6-MBP-PPR65+ccmFC_native	20h	$2x10^5$	DMEM+FCS+Penstrep	25µM	37%	This study
53	Human	Cell line/ Construct	HeLa	EYFP-PPR56+nad4_native	20h	$2x10^5$	DMEM+FCS+Penstrep	25µM	51%	This study
54	Human	Cell line/ Construct	HeLa	EYFP-PPR56+nad4_native	20h	$2x10^5$	DMEM+FCS+Penstrep	25µM	54%	This study
55	Human	Cell line/ Construct	HeLa	EYFP-PPR56+nad4_native	20h	$2x10^5$	DMEM+FCS+Penstrep	25µM	55%	This study
56	Human	Cell line/ Construct	HeLa	EYFP-PPR56+nad4_native	20h	$2x10^5$	DMEM+FCS+Penstrep	25µM	63%	This study
57	Human	Cell line/ Construct	HeLa	EYFP-PPR56+nad4_native	20h	$2x10^5$	DMEM+FCS+Penstrep	25µM	51%	This study
58	Human	Cell line/ Construct	HeLa	EYFP-PPR56+nad4_native	20h	$2x10^5$	DMEM+FCS+Penstrep	25µM	63%	This study
59	Human	Cell line/ Construct	HeLa	EYFP-PPR56+nad4_native	20h	$2x10^5$	DMEM+FCS+Penstrep	25µM	57%	This study
60	Human	Cell line/ Construct	HeLa	EYFP-PPR65+ccmFC_native	20h	$2x10^5$	DMEM+FCS+Penstrep	25µM	24%	This study
61	Human	Cell line/ Construct	HeLa	EYFP-PPR65+ccmFC_native	20h	$2x10^5$	DMEM+FCS+Penstrep	25µM	24%	This study
62	Human	Cell line/ Construct	HeLa	EYFP-PPR65+ccmFC_native	20h	$2x10^5$	DMEM+FCS+Penstrep	25µM	27%	This study
63	Human	Construct	HeLa	EYFP-MBP-PPR56+nad4_native	20h	$2x10^5$	DMEM+FCS+Penstrep	25µM	42%	This study
64	Human	Construct	HeLa	EYFP-MBP-PPR56+nad4_native	20h	$2x10^5$	DMEM+FCS+Penstrep	25µM	43%	This study
65	Human	Construct	HeLa	EYFP-MBP-PPR56+nad4_native	20h	$2x10^5$	DMEM+FCS+Penstrep	25µM	38%	This study
66	Human	Construct	HeLa	EYFP-MBP-PPR56+nad4_native	20h	$2x10^5$	DMEM+FCS+Penstrep	25µM	45%	This study
67	Human	Construct	HeLa	HA-MBP-PPR56+nad4_native	20h	$2x10^5$	DMEM+FCS+Penstrep	25µM	60%	This study
68	Human	Construct	HeLa	HA-MBP-PPR56+nad4_native	20h	$2x10^5$	DMEM+FCS+Penstrep	25µM	62%	This study
69	Human	Construct	HeLa	HA-MBP-PPR56+nad4_native	20h	$2x10^5$	DMEM+FCS+Penstrep	25µM	49%	This study
70	Human	Construct	HeLa	HA-MBP-PPR56+nad4_native	20h	$2x10^5$	DMEM+FCS+Penstrep	25µM	38%	This study
71	Human	Construct	HeLa	EYFP-MBP-PPR65+ccmFC_native	20h	$2x10^5$	DMEM+FCS+Penstrep	25µM	28%	This study
72	Human	Construct	HeLa	EYFP-MBP-PPR65+ccmFC_native	20h	$2x10^5$	DMEM+FCS+Penstrep	25µM	26%	This study
73	Human	Construct	HeLa	EYFP-MBP-PPR65+ccmFC_native	20h	$2x10^5$	DMEM+FCS+Penstrep	25µM	27%	This study
74	Human	Construct	HeLa	EYFP-MBP-PPR65+ccmFC_native	20h	$2x10^5$	DMEM+FCS+Penstrep	25µM	26%	This study
75	Human	Construct	HeLa	HA-MBP-PPR65+ccmFC_native	20h	$2x10^5$	DMEM+FCS+Penstrep	25µM	52%	This study
76	Human	Construct	HeLa	HA-MBP-PPR65+ccmFC_native	20h	$2x10^5$	DMEM+FCS+Penstrep	25µM	57%	This study

77	Human	Construct	HeLa	HA-MBP-PPR65+ccmFC_native	20h	$2x10^5$	DMEM+FCS+Penstrep	25µM	32%	This study
78	Human	Construct	HeLa	HA-MBP-PPR65+ccmFC_native	20h	$2x10^5$	DMEM+FCS+Penstrep	25µM	40%	This study
79	Human	Construct	HeLa	HA-PPR56+nad4_native	20h	$2x10^5$	DMEM+FCS+Penstrep	25µM	62%	This study
80	Human	Construct	HeLa	HA-PPR56+nad4_native	20h	$2x10^5$	DMEM+FCS+Penstrep	25µM	62%	This study
81	Human	Construct	HeLa	HA-PPR56+nad4_native	20h	$2x10^5$	DMEM+FCS+Penstrep	25µM	38%	This study
82	Human	Construct	HeLa	HA-PPR56+nad4_native	20h	$2x10^5$	DMEM+FCS+Penstrep	25µM	38%	This study
83	Human	Construct	HeLa	EYFP-His_6-MBP-PPR65+ccmFC_native	20h	$2x10^5$	DMEM+FCS+Penstrep	25µM	27%	This study
84	Human	Construct	HeLa	EYFP-His_6-MBP-PPR65+ccmFC_native	20h	$2x10^5$	DMEM+FCS+Penstrep	25µM	29%	This study
85	Human	Construct	HeLa	EYFP-His_6-MBP-PPR65+ccmFC_native	20h	$2x10^5$	DMEM+FCS+Penstrep	25µM	22%	This study
86	Human	Construct	HeLa	EYFP-His_6-MBP-PPR65+ccmFC_native	20h	$2x10^5$	DMEM+FCS+Penstrep	25µM	19%	This study
87	Human	Construct	HeLa	EYFP-PPR65+ccmFC_native	20h	$2x10^5$	DMEM+FCS+Penstrep	25µM	5%	This study
88	Human	Construct	HeLa	EYFP-PPR65+ccmFC_native	20h	$2x10^5$	DMEM+FCS+Penstrep	25µM	29%	This study
89	Human	Construct	HeLa	EYFP-PPR65+ccmFC_native	20h	$2x10^5$	DMEM+FCS+Penstrep	25µM	28%	This study
90	Human	Construct	HeLa	EYFP-PPR65+ccmFC_native	20h	$2x10^5$	DMEM+FCS+Penstrep	25µM	18%	This study
91	Human	Construct	HeLa	EYFP-PPR65+ccmFC_native	20h	$2x10^5$	DMEM+FCS+Penstrep	25µM	19%	This study
92	Human	Construct	HeLa	HA-PPR65+ccmFC_native	20h	$2x10^5$	DMEM+FCS+Penstrep	25µM	1%	This study
93	Human	Construct	HeLa	HA-PPR65+ccmFC_native	20h	$2x10^5$	DMEM+FCS+Penstrep	25µM	0%	This study
94	Human	Construct	HeLa	HA-PPR65+ccmFC_native	20h	$2x10^5$	DMEM+FCS+Penstrep	25µM	0%	This study
95	Human	Construct	HeLa	HA-PPR65+ccmFC_native	20h	$2x10^5$	DMEM+FCS+Penstrep	25µM	0%	This study
96	Human	Construct	HeLa	EYFP-His_6-MBP-PPR56+nad4_native	20h	$2x10^5$	DMEM+FCS+Penstrep	25µM	30%	This study
97	Human	Construct	HeLa	EYFP-His_6-MBP-PPR56+nad4_native	20h	$2x10^5$	DMEM+FCS+Penstrep	25µM	42%	This study
98	Human	Construct	HeLa	EYFP-His_6-MBP-PPR56+nad4_native	20h	$2x10^5$	DMEM+FCS+Penstrep	25µM	38%	This study
99	Human	Construct	HeLa	EYFP-His_6-MBP-PPR56+nad4_native	20h	$2x10^5$	DMEM+FCS+Penstrep	25µM	37%	This study
100	Human	Construct	HeLa	EYFP-His_6-MBP-PPR56+nad4_native	20h	$2x10^5$	DMEM+FCS+Penstrep	25µM	33%	This study
101	Human	DYW domain mutants	HeLa	EYFP-PPR56_CxDAH+nad4_native	20h	$2x10^5$	DMEM+FCS+Penstrep	25µM	0%	This study
102	Human	DYW domain mutants	HeLa	EYFP-PPR56_CxDAH+nad4_native	20h	$2x10^5$	DMEM+FCS+Penstrep	25µM	0%	This study
103	Human	DYW domain mutants	HeLa	EYFP-PPR56_CxDAH+nad4_native	20h	$2x10^5$	DMEM+FCS+Penstrep	25µM	0%	This study
104	Human	DYW domain mutants	HeLa	EYFP-PPR56_HSERLA+nad4_native	20h	$2x10^5$	DMEM+FCS+Penstrep	25µM	20%	This study
105	Human	DYW domain mutants	HeLa	EYFP-PPR56_HSERLA+nad4_native	20h	$2x10^5$	DMEM+FCS+Penstrep	25µM	11%	This study
106	Human	DYW domain mutants	HeLa	EYFP-PPR56_HSERLA+nad4_native	20h	$2x10^5$	DMEM+FCS+Penstrep	25µM	22%	This study
107	Human	DYW domain mutants	HeLa	HA-His_6-MBP-PPR65_CxDAH+ccmFC_native	20h	$2x10^5$	DMEM+FCS+Penstrep	25µM	0%	This study
108	Human	DYW domain mutants	HeLa	HA-His_6-MBP-PPR65_CxDAH+ccmFC_native	20h	$2x10^5$	DMEM+FCS+Penstrep	25µM	0%	This study
109	Human	DYW domain mutants	HeLa	HA-His_6-MBP-PPR65_CxDAH+ccmFC_native	20h	$2x10^5$	DMEM+FCS+Penstrep	25µM	0%	This study
110	Human	DYW domain mutants	HeLa	HA-His_6-MBP-PPR65_HSARLA+ccmFC_native	20h	$2x10^5$	DMEM+FCS+Penstrep	25µM	0%	This study
111	Human	DYW domain mutants	HeLa	HA-His_6-MBP-PPR65_HSARLA+ccmFC_native	20h	$2x10^5$	DMEM+FCS+Penstrep	25µM	0%	This study
112	Human	DYW domain mutants	HeLa	HA-His_6-MBP-PPR65_HSARLA+ccmFC_native	20h	$2x10^5$	DMEM+FCS+Penstrep	25µM	0%	This study
113	Human	DYW domain mutants	IMR-90	EYFP-PPR56_CxDAH+nad4_native	20h	$2x10^5$	MEM+FCS+Penstrep	25µM	0%	This study
114	Human	DYW domain mutants	IMR-90	EYFP-PPR56_CxDAH+nad4_native	20h	$2x10^5$	MEM+FCS+Penstrep	25µM	0%	This study
115	Human	S motif mutants	IMR-90	EYFP-PPR56_S4mut+nad4_native	20h	$2x10^5$	MEM+FCS+Penstrep	25µM	0%	This study
116	Human	S motif mutants	IMR-90	EYFP-PPR56_S4mut+nad4_native	20h	$2x10^5$	MEM+FCS+Penstrep	25µM	0%	This study
117	Human	S motif mutants	IMR-90	EYFP-PPR56_S4mut+nad4_native	20h	$2x10^5$	MEM+FCS+Penstrep	25µM	1%	This study
118	Human	S motif mutants	IMR-90	EYFP-PPR56_S7mut+nad4_native	20h	$2x10^5$	MEM+FCS+Penstrep	25µM	0%	This study

119	Human	S motif mutants	IMR-90	EYFP-PPR56_S7mut+nad4_native	20h	$2x10^5$	MEM+FCS+Penstrep	25µM	0%	This study
120	Human	S motif mutants	IMR-90	EYFP-PPR56_S7mut+nad4_native	20h	$2x10^5$	MEM+FCS+Penstrep	25µM	0%	This study
121	Human	S motif mutants	IMR-90	EYFP-PPR56_S4mut+nad4_-7ag	20h	$2x10^5$	MEM+FCS+Penstrep	25µM	70%	This study
122	Human	S motif mutants	IMR-90	EYFP-PPR56_S4mut+nad4_-7ag	20h	$2x10^5$	MEM+FCS+Penstrep	25µM	67%	This study
123	Human	S motif mutants	IMR-90	EYFP-PPR56_S4mut+nad4_-7ag	20h	$2x10^5$	MEM+FCS+Penstrep	25µM	58%	This study
124	Human	S motif mutants	IMR-90	EYFP-PPR56_S7mut+nad4_-10ga	20h	$2x10^5$	MEM+FCS+Penstrep	25µM	31%	This study
125	Human	S motif mutants	IMR-90	EYFP-PPR56_S7mut+nad4_-10ga	20h	$2x10^5$	MEM+FCS+Penstrep	25µM	29%	This study
126	Human	S motif mutants	IMR-90	EYFP-PPR56_S7mut+nad4_-10ga	20h	$2x10^5$	MEM+FCS+Penstrep	25µM	30%	This study
127	Human	Zinc/time	HeLa	EYFP-PPR56+nad4_native	14h	$1x10^5$	DMEM+FCS+Penstrep	100µM	39%	This study
128	Human	Zinc/time	HeLa	EYFP-PPR56+nad4_native	14h	$1x10^5$	DMEM+FCS+Penstrep	100µM	42%	This study
129	Human	Zinc/time	HeLa	EYFP-PPR56+nad4_native	14h	$1x10^5$	DMEM+FCS+Penstrep	100µM	27%	This study
130	Human	Zinc/time	HeLa	EYFP-PPR56+nad4_native	20h	$1x10^5$	DMEM+FCS+Penstrep	100µM	51%	This study
131	Human	Zinc/time	HeLa	EYFP-PPR56+nad4_native	20h	$1x10^5$	DMEM+FCS+Penstrep	100µM	44%	This study
132	Human	Zinc/time	HeLa	EYFP-PPR56+nad4_native	20h	$1x10^5$	DMEM+FCS+Penstrep	100µM	39%	This study
133	Human	Zinc/time	HeLa	EYFP-PPR56+nad4_native	40h	$1x10^5$	DMEM+FCS+Penstrep	100µM	45%	This study
134	Human	Zinc/time	HeLa	EYFP-PPR56+nad4_native	40h	$1x10^5$	DMEM+FCS+Penstrep	100µM	47%	This study
135	Human	Zinc/time	HeLa	EYFP-PPR56+nad4_native	40h	$1x10^5$	DMEM+FCS+Penstrep	100µM	42%	This study
136	Human	Zinc/time	HeLa	EYFP-PPR56+nad4_native	14h	$1x10^5$	DMEM+FCS+Penstrep	25µM	40%	This study
137	Human	Zinc/time	HeLa	EYFP-PPR56+nad4_native	14h	$1x10^5$	DMEM+FCS+Penstrep	25µM	36%	This study
138	Human	Zinc/time	HeLa	EYFP-PPR56+nad4_native	14h	$1x10^5$	DMEM+FCS+Penstrep	25µM	29%	This study
139	Human	Zinc/time	HeLa	EYFP-PPR56+nad4_native	20h	$1x10^5$	DMEM+FCS+Penstrep	25µM	45%	This study
140	Human	Zinc/time	HeLa	EYFP-PPR56+nad4_native	20h	$1x10^5$	DMEM+FCS+Penstrep	25µM	45%	This study
141	Human	Zinc/time	HeLa	EYFP-PPR56+nad4_native	20h	$1x10^5$	DMEM+FCS+Penstrep	25µM	32%	This study
142	Human	Zinc/time	HeLa	EYFP-PPR56+nad4_native	40h	$1x10^5$	DMEM+FCS+Penstrep	25µM	49%	This study
143	Human	Zinc/time	HeLa	EYFP-PPR56+nad4_native	40h	$1x10^5$	DMEM+FCS+Penstrep	25µM	46%	This study
144	Human	Zinc/time	HeLa	EYFP-PPR56+nad4_native	40h	$1x10^5$	DMEM+FCS+Penstrep	25µM	47%	This study
145	Human	Zinc/time	HeLa	EYFP-PPR56+nad4_native	14h	$1x10^5$	DMEM+FCS+Penstrep	no	42%	This study
146	Human	Zinc/time	HeLa	EYFP-PPR56+nad4_native	14h	$1x10^5$	DMEM+FCS+Penstrep	no	30%	This study
147	Human	Zinc/time	HeLa	EYFP-PPR56+nad4_native	14h	$1x10^5$	DMEM+FCS+Penstrep	no	35%	This study
148	Human	Zinc/time	HeLa	EYFP-PPR56+nad4_native	20h	$1x10^5$	DMEM+FCS+Penstrep	no	43%	This study
149	Human	Zinc/time	HeLa	EYFP-PPR56+nad4_native	20h	$1x10^5$	DMEM+FCS+Penstrep	no	54%	This study
150	Human	Zinc/time	HeLa	EYFP-PPR56+nad4_native	20h	$1x10^5$	DMEM+FCS+Penstrep	no	36%	This study
151	Human	Zinc/time	HeLa	EYFP-PPR56+nad4_native	40h	$1x10^5$	DMEM+FCS+Penstrep	no	48%	This study
152	Human	Zinc/time	HeLa	EYFP-PPR56+nad4_native	40h	$1x10^5$	DMEM+FCS+Penstrep	no	62%	This study
153	Human	Zinc/time	HeLa	EYFP-PPR56+nad4_native	40h	$1x10^5$	DMEM+FCS+Penstrep	no	50%	This study
154	*Escherichia coli*	S motif mutants	Rosetta 2 (DE3)	His_6-MBP-PPR56_S4mut+nad4_native	20h	$3.2x10^8$ to $4.6x10^8$	LB+KanCam	0.4mM	98%	This study
155	*Escherichia coli*	S motif mutants	Rosetta 2 (DE3)	His_6-MBP-PPR56_S4mut+nad4_native	20h	$3.2x10^8$ to $4.6x10^8$	LB+KanCam	0.4mM	98%	This study
156	*Escherichia coli*	S motif mutants	Rosetta 2 (DE3)	His_6-MBP-PPR56_S4mut+nad4_native	20h	$3.2x10^8$ to $4.6x10^8$	LB+KanCam	0.4mM	81%	This study
157	*Escherichia coli*	S motif mutants	Rosetta 2 (DE3)	His_6-MBP-PPR56_S7mut+nad4_native	20h	$3.2x10^8$ to $4.6x10^8$	LB+KanCam	0.4mM	0%	This study
158	*Escherichia coli*	S motif mutants	Rosetta 2 (DE3)	His_6-MBP-PPR56_S7mut+nad4_native	20h	$3.2x10^8$ to $4.6x10^8$	LB+KanCam	0.4mM	0%	This study
159	*Escherichia coli*	S motif mutants	Rosetta 2 (DE3)	His_6-MBP-PPR56_S7mut+nad4_native	20h	$3.2x10^8$ to $4.6x10^8$	LB+KanCam	0.4mM	0%	This study
160	*Escherichia coli*	S motif mutants	Rosetta 2 (DE3)	His_6-MBP-PPR56_S7mut+nad4_-10ga	20h	$3.2x10^8$ to $4.6x10^8$	LB+KanCam	0.4mM	0%	This study

161	*Escherichia coli*	S motif mutants	Rosetta 2 (DE3)	His_6-MBP-PPR56_S7mut+nad4_-10ga	20h	$3.2x10^8$ to $4.6x10^8$	LB+KanCam	0.4mM	0%	This study
162	*Escherichia coli*	S motif mutants	Rosetta 2 (DE3)	His_6-MBP-PPR56_S7mut+nad4_-10ga	20h	$3.2x10^8$ to $4.6x10^8$	LB+KanCam	0.4mM	0%	This study
163	*Escherichia coli*	S motif mutants	Rosetta 2 (DE3)	His_6-MBP-PPR56_S4mut+nad4_-7ag	20h	$3.2x10^8$ to $4.6x10^8$	LB+KanCam	0.4mM	100%	This study
164	*Escherichia coli*	S motif mutants	Rosetta 2 (DE3)	His_6-MBP-PPR56_S4mut+nad4_-7ag	20h	$3.2x10^8$ to $4.6x10^8$	LB+KanCam	0.4mM	100%	This study
165	*Escherichia coli*	S motif mutants	Rosetta 2 (DE3)	His_6-MBP-PPR56_S4mut+nad4_-7ag	20h	$3.2x10^8$ to $4.6x10^8$	LB+KanCam	0.4mM	98%	This study
166	*Escherichia coli*	DYW domain mutants	Rosetta 2 (DE3)	His_6-MBP-PPR56_HSERLA+nad4_native	20h	$3.2x10^8$ to $4.6x10^8$	LB+KanCam	0.4mM	79%	This study
167	*Escherichia coli*	DYW domain mutants	Rosetta 2 (DE3)	His_6-MBP-PPR56_HSERLA+nad4_native	20h	$3.2x10^8$ to $4.6x10^8$	LB+KanCam	0.4mM	80%	This study
168	*Escherichia coli*	DYW domain mutants	Rosetta 2 (DE3)	His_6-MBP-PPR56_HSERLA+nad4_native	20h	$3.2x10^8$ to $4.6x10^8$	LB+KanCam	0.4mM	78%	This study
169	*Escherichia coli*	DYW domain mutants	Rosetta 2 (DE3)	His_6-MBP-PPR56_CxDAH+nad4_native	20h	$3.2x10^8$ to $4.6x10^8$	LB+KanCam	0.4mM	0%	This study
170	*Escherichia coli*	DYW domain mutants	Rosetta 2 (DE3)	His_6-MBP-PPR56_CxDAH+nad4_native	20h	$3.2x10^8$ to $4.6x10^8$	LB+KanCam	0.4mM	0%	This study
171	*Escherichia coli*	DYW domain mutants	Rosetta 2 (DE3)	His_6-MBP-PPR56_CxDAH+nad4_native	20h	$3.2x10^8$ to $4.6x10^8$	LB+KanCam	0.4mM	0%	This study
172	*Escherichia coli*	DYW domain mutants	Rosetta 2 (DE3)	His_6-MBP-PPR56+nad4_native	20h	$3.2x10^8$ to $4.6x10^8$	LB+KanCam	0.4mM	100±0%	(3)
173	*Escherichia coli*	DYW domain mutants	Rosetta 2 (DE3)	His_6-MBP-PPR65+ccmFC_native	20h	$3.2x10^8$ to $4.6x10^8$	LB+KanCam	0.4mM	96±4%	(3)
174	*Escherichia coli*	DYW domain mutants	Rosetta 2 (DE3)	His_6-MBP-PPR65_HSARLA+ccmFC_native	20h	$3.2x10^8$ to $4.6x10^8$	LB+KanCam	0.4mM	0±0%	(3)
175	*Escherichia coli*	DYW domain mutants	Rosetta 2 (DE3)	His_6-MBP-PPR65_CxDAH+ccmFC_native	20h	$3.2x10^8$ to $4.6x10^8$	LB+KanCam	0.4mM	0±0%	(3)

Supplementary Table S3

Transfected human IMR-90 cell lines sent for RNA sequencing. The following information is included: cell line, RNASeq batch, expressed fusion protein, delivered target, replicate number of respective constructs, FACS sorting prior to RNA preparation, amounts of raw reads, reads left for mapping after quality trimming and numbers of removed plasmid matching reads (in brackets are percentages of mapped reads to EYFP-plasmid vs. HA-His$_6$-MBP-PPR65 plasmid for co-transfected cells), editing efficiencies of the respective delivered targets (%editing) and the fraction of off-targets, which are confirmed in the individual replicates. The corresponding SRA data is deposited within BioProject PRJNA832818.

Name	Cell line	RNASeq batch	Expressed protein	Delivered target	Replicate	FACS sorted	Raw reads	After quality trimming > % of raw reads	Matching plasmid > % of clean reads	Reads for mapping	%editing > delivered target	Off-targets confirmed
IMR1	IMR-90	2	EYFP-PPR56	nad4	1	Yes	99106154	87380226 (88.2%)	32.4%	59007884	71%	727/906 (80.2%)
IMR2	IMR-90	1	EYFP-PPR56	nad4	2	Yes	117428040	101532360 (86.5%)	27.2%	73903770	71%	839/906 (92.6%)
IMR3	IMR-90	1	EYFP-PPR56	nad4	3	Yes	110474636	95441720 (86.4%)	28.4%	68253350	70%	814/906 (89.8%)
ELIM1	IMR-90	2	EYFP-PPR56	nad4	4	No	115482810	103838880 (89.9%)	26.1%	76716486	72%	533/906 (58.8%)
IMR5	IMR-90	1	EYFP-PPR56-CxDAH	nad4	1	Yes	124316786	107041332 (86.1%)	27.0%	78065000	0%	/
I-1-2	IMR-90	3	EYFP-PPR56_S4TN>TD	nad4	1	Yes	128657316	115191558 (89.5%)	38.3%	70994072	27%	2063/2265 (91.1%)
I-3	IMR-90	3	EYFP-PPR56_S4TN>TD	nad4-7AG	2	Yes	112121016	100559526 (89.7%)	37.4%	62881814	65%	1987/2265 (87.7%)
I-4	IMR-90	3	EYFP-PPR56_S4TN>TD	nad4-7AG	3	Yes	122435238	108661546 (88.8%)	38.5%	66747124	64%	2028/2265 (89.5%)
I-6-2	IMR-90	3	EYFP-PPR56_S7TD>TN	nad4	1	Yes	126830966	113777666 (89.7%)	39.2%	69104234	5%	284/323 (87.9%)
I-7	IMR-90	3	EYFP-PPR56_S7TD>TN	nad4-10GA	2	Yes	104658340	93086300 (88.9%)	44.6%	51480558	53%	261/323 (80.8%)
I-8	IMR-90	3	EYFP-PPR56_S7TD>TN	nad4-10GA	3	Yes	133100572	118817108 (89.3%)	44.4%	65931130	50%	290/323 (89.8%)
I-9	IMR-90	3	EYFP + HA-His$_6$-MBP-PPR65 (co-transfection)	ccmFC	1	Yes	249859798	221774196 (88.8%)	30.1% (5.6%/ 24.5%)	154861296	19%	4/7 (57.1%)
I-10	IMR-90	3	EYFP + HA-His$_6$-MBP-PPR65 (co-transfection)	ccmFC	2	Yes	274709688	242595378 (88.3%)	30.5% (4.9%/ 25.6%)	168492352	20%	7/7 (100%)
I-12	IMR-90	3	EYFP + HA-His$_6$-MBP-PPR65 (co-transfection)	ccmFC	3	Yes	255509484	225227710 (88.2%)	30.6% (6.5%/ 24.1%)	156244988	20%	5/7 (71.4%)
IMR8	IMR-90	1	EYFP	none	Control PPR56	Yes	110753644	98081586 (88.6%)	19.4%	78948974	no PPR/ target	/
I-13	IMR-90	3	EYFP + HA-His$_6$-MBP (co-transfection)	none	Control PPR65	Yes	139760184	124347896 (89.0%)	25.0% (5.5%/ 19.5%)	93136262	no PPR/ target	/

Supplementary Table S4

PPR56_native, PPR56_S7TD>TN and PPR56_S4TN>TD off-targets detected in the IMR-90 transcriptome. Off-targets verified in at least two replicates of EYFP-PPR56_native (A, 56-N), EYFP-PPR56_S7TD>TN (B, 56-S7) and EYFP-PPR56_S4TN>TD (C, 56-S4) in the IMR-90 transcriptome. The off-target label reveals the gene ID, the transcript accession with the position of the edited C, the corresponding protein accession with the position of the affected amino acid and the induced amino acid change. Off-targets in non-coding transcripts are labeled with 'ncr' and for sites in 3' or 5' UTRs the distance to the respective CDS is displayed. For comparative reasons, the number of replicates in which the site was called (Rep.) and the mean C-U conversion rates (%CU) with standard deviations (STABW) of off-targets identified for PPR56_native (A), EYFP-PPR56_S7TD>TN (B) and EYFP-PPR56_S4TN>TD (C), respectively, are highlighted in bold and, in case of shared off-targets, presented together with the respective information of the other PPR56 versions. For PPR56_native, rates excluding (56-N-3) and including the unsorted sample (65-N-4) are depicted. Nucleotides from position -30 up- to +5 downstream of the editing sites are listed. Nucleotide shading indicates binding code matches to the corresponding PPR in green and transitions in yellow (see Figure 1). PPR-target match scoring was applied as explained in Figure 9. Alternative matching scores were calculated for PPR-RNA binding shifted one up to two positions up (+1/+2) or down (-1/-2). C-U conversion rates of individual replicates (labeled as in Supplementary table S3) are shown on the right. Sites detected in the same position of a gene, but in different transcript/ protein variants are colored in blue.

A) PPR56_native

>total: 906

Offtarget<br>blue > same position in gene	Rep. 56-N	%CU 56-N-4	STABW 56-N-4	%CU 56-N-3	STABW 56-N-3	Rep. 56-S7	%CU 56 S7	STABW 56-S7	Rep. 56-S4	%CU 56-S4	STABW 56-S4	PPR56 native Scoring -2	-1	Match	+1	+2	Binding region	PPR56-native %IMR1	%IMR2	%IMR3	%ELIM1	PPR56-S7mut %I6-2	%I7	%I8	PPR56-S4mut %I1-2	%I3	%I4
ABCB7-NM_001271696.3eU1398-NP_001258625.1aa463LF	4	39%	11%	44%	6%	1	4%		3	7%	3%	260	160	760	310	290	C A A A T T A A A G A C A A A G T G A T G G C A T C T C C C C T T C A G	48%	37%	48%	24%		4%		4%	9%	7%
ABCC1-NM_004996.4eU5943-3'UTR+1211	4	3%	0%	3%	0%	0			0			310	320	410	440	420	C T T T T C C T T G T T T T T A G A T G T T A A T T C T C T C C C T T G	3%	3%	3%	2%						
ABHD2-NM_007011.8eU1421-NP_008942.3aa270SL	3	6%	4%	8%	4%	0			0			480	200	580	330	400	T G G C T G A C A A C A T G A A G A A G A T C A T C C T C T C G C A C A	5%	10%		3%						
ABHD3-NM_001308256.2eU996-NP_001295185.1aa296SL	4	8%	3%	9%	2%	0			3	67%	3%	410	130	700	290	410	T G T G T C T A A A T T C T G T G G A T G A T G T T T T C T C A C C C A	10%	7%	11%	4%				70%	64%	66%
ACAA2-NM_006111.3eU1436-3'UTR+193	4	8%	4%	10%	1%	0			0			340	220	580	400	500	A T A G G A G C C C A T G C T A G A A G T A C A T T C T C T C A G A T T	11%	10%	9%	3%						
ACOT8-NM_005469.4eU203-NP_005460.2aa42FF	4	3%	1%	3%	2%	0			0			610	270	490	420	230	C A A C C T C G A G C C G C T G G A C G A G G A T C T C T T C A G A G G	3%	5%	2%	2%						
ACTR1A-NM_005736.4eU1238-3'UTR+42	4	3%	1%	3%	1%	0			0			260	160	660	210	210	C A T C T T C A C C T C T C T C T G A A G T T A A C T C C A C T T T A A	4%	2%	3%	2%						
ACTR2-NM_001005386.3eU312-NP_001005386.1aa78PL	3	5%	1%	5%	1%	0			0			210	280	700	240	240	A A T T A C G A T C A A T G T T A G A A G T T A A C T A C C C T A T G G	5%	5%	6%							
ACTR2-NM_005722.4eU297-NP_005713.1aa73PL	4	4%	2%	5%	0%	0			0			210	280	700	240	240	A A T T A C G A T C A A T G T T A G A A G T T A A C T A C C C T A T G G	5%	5%	5%	1%						
ADAR-NM_001025107.3eU2198-NP_001020278.1aa404SL	4	3%	2%	4%	0%	0			0			220	240	790	80	270	C T T C T G A T A A C C A G C C T G A A G G T A T G A T C T C A G A G T	5%	4%	4%	1%						
ADAR-NM_001025107.3eU4034-3'UTR+251	4	36%	13%	42%	2%	2	2%	0%	3	2%	0%	340	210	790	220	250	A A G A G G C A G A A A C C T A A G A A G T T A T C T T T T C T T T C T	45%	41%	40%	16%	1%		2%	3%	2%	2%
AFDN-NM_001040000.3eU3958-NP_001035089.1aa1246Q*	2	4%	1%	4%	1%	0			0			370	90	710	400	100	T C C A A A T C C C A G G A T C G G A T G G C T C C T C C T C A G A A C	4%	4%								
AGFG1-NM_001135189.2eU1671-NP_001128661.1aa469Q*	4	5%	2%	6%	1%	3	51%	7%	2	4%	1%	310	310	730	230	390	G C A G C T T T C C C T C A A C A G A C A G C T T T T T C T C A A C A G	7%	6%	4%	3%	51%	57%	43%	4%		3%
AGFG2-NM_006076.5eU585-NP_006067.3aa147PP	3	9%	6%	9%	6%	0			0			370	260	710	310	430	A A A A T A T G A G A A G A A G A G A T G G T A T G T C C C C C C A G A	15%	10%	3%							
AGGF1-NM_018046.5eU2385-NP_060516.2aa674LL	3	5%	1%	5%	1%	0			0			360	260	510	400	260	A C C A T C C T C A T T T G A A G A T G T T C A C C T T C T C C A A A A	4%	5%	4%							
AGPAT5-NM_018361.5eU2324-3'UTR+1202	4	34%	7%	37%	4%	0			1	8%		350	160	640	440	260	C A T T T G T C T G T G T C A A G A A G T T C A C C T T C T C A A G C C	33%	36%	41%	25%					8%	
AGPS-NM_003659.4eU966-NP_003650.1aa317RC	3	2%	0%	2%	0%	0			0			370	130	600	350	430	T T C A G T A C T G T A G G A G G A T G G T A T C T A C T C G C G C A		2%	3%	2%						
AHI1-NM_001134830.2eU374-NP_001128302.1aa54HH	4	19%	10%	22%	9%	0			0			210	200	820	260	340	C T C A C C T G A C A C T A T T A G A A G C A A T C T T C A C T A T A T	23%	31%	13%	9%						
AHI1-NM_001134830.2eU428-NP_001128302.1aa72PP	4	35%	16%	40%	17%	0			2	6%	1%	170	370	820	360	290	T G A T C C C G A C A C T A T T A G A A G C A A T C T T C C C C A T A T	38%	57%	24%	23%				6%		7%
AHSA1-NM_001321441.2eU1234-3'UTR+181	4	30%	10%	34%	3%	0			0			350	300	790	300	320	T C T G C T G G G T G G G T T C A G A G G G C A A T T T C T C T T T T A	37%	34%	32%	15%						
AHSA1-NM_001321441.2eU809-NP_001308370.1aa123SF	4	6%	2%	7%	1%	0			0			210	240	770	80	220	G A A A G T T C C A C A T G G T A G A T G G C A A C G T C T C T G G G G	8%	6%	7%	4%						
AIDA-NM_022831.4eU2690-3'UTR+1595	4	14%	5%	16%	3%	2	2%	0%	0			270	440	560	420	520	G C T C T T C A A G G A G C A A G A G G C G C A T T T T C T C C G T G T	19%	13%	16%	7%	2%		2%			
AJUBA-NM_032876.6eU2234-3'UTR+332	4	6%	3%	6%	4%	0			0			310	200	740	310	270	A T T T T G T T T G T T T T A G A G A T G G G A T C T C A C C A T G T T	4%	11%	5%	4%						
AK4-NM_001005353.3eU1502-3'UTR+495	4	16%	5%	18%	3%	0			0			320	140	700	300	260	C T G G T A A C A G T G A A A T T G A A G C T A C T T A C T C A T A G T	15%	20%	20%	10%						
AKAP12-NM_005100.4eU586-NP_005091.2aa105TI	4	9%	3%	10%	1%	0			0			160	210	790	190	400	A C A G C C A G G A G G A A G A A G A A G T C A T T G T C A C A G A G G	10%	10%	11%	4%						
AKAP13-NM_001270546.1eU8455-3'UTR+4008	3	7%	1%	7%	1%	0			0			240	460	760	400	190	G G G G A G G A A G G G A A A A T A A A G G C A T C T T T C C A A G T A	9%	6%	7%							
ALDH18A1-NM_001017423.2eU2239-NP_001017423.1aa699TI	2	3%	1%	3%		0			0			160	70	760	160	300	A T G G C A G C T C C C A C A C G G A T G T C A T C G T C A C A G A G G		3%		2%						
ALS2-NM_001135745.2eU109-5'UTR-10	3	7%	2%	8%	2%	0			3	22%	3%	310	230	700	320	290	A G A A G G A A T G A T T T G G T G A T G G A G T G T T C C C A C T G A	10%	7%		5%				20%	20%	25%
ANAPC13-NM_001242374.1eU989-3'UTR+33	4	9%	2%	10%	2%	0			0			330	470	690	130	440	C T T G G C T C C T T T C T T G T G G A T G G A T T T T C T C A A A G T	11%	11%	8%	6%						
ANKRD11-NM_001256182.2eU6183-NP_001243111.1aa1884SL	4	5%	1%	6%	1%	0			3	21%	4%	350	210	870	230	330	C T G T C A C C C C G T C T C C A G A G G G C G T C T T C T C A A G T T	6%	5%	5%	4%				17%	23%	24%
ANKRD33B-NM_001164440.2eU6171-3'UTR+4288	3	6%	2%	6%	2%	3	19%	6%	0			230	280	690	310	240	T A G T G A A T T T C A A G T T G A C A G G T A C C T T C T C A G G G A	7%	8%	4%		26%	17%	14%			
ANTXR2-NM_001286780.2eU1438-3'UTR+45	3	47%	10%	52%	8%	0			0			570	220	770	270	120	G A C C A A G A A G G T A C G A A G A T G G C A C A T T T T C A C A T A	57%		46%	36%						
AOPEP-XM_017015231.2eU6068-3'UTR+3687	2	40%	5%	44%		0			0			110	40	410	40	390	A C G G C G A G G T G A G C T G A G A C T T C T G A G A A C A G T T A			44%	36%						
AP1G1-NM_001030007.2eU381-NP_001025178.1aa102LL	4	19%	7%	22%	3%	0			1	5%		270	110	620	390	330	G T T A G A T G A A A G A C A A G A T G T C C A T C T T C T C A T G A C	20%	25%	22%	9%					5%	
AP2B1-NM_001030006.2eU2707-NP_001025177.1aa859PP	3	2%	0%	2%	0%	0			0			380	520	650	200	300	G G T C T T C C T T G C A A C A T G G A A G G A T A T T C C C A A T G A		1%	2%	1%						
AP3D1-NM_001261826.3eU2737-NP_001248755.1aa835PP	3	3%	0%	4%	0%	0			0			340	510	510	370	270	G A C C T C A A A A T C C C C T G A G A A G G A C G T T C C C A T G G T		4%	3%	3%						
AP3D1-NM_001374799.1eU2737-NP_001361728.1aa835PP	3	9%	1%	9%	1%	0			0			340	510	510	370	270	G A C C T C A A A A T C C C C T G A G A A G G A C G T T C C C A T G G T	10%	8%	8%							
AP4E1-NM_001252127.2eU2270-NP_001239056.1aa642PP	4	18%	7%	20%	6%	0			2	15%	6%	250	230	710	570	390	G A A A T T G T G G G G G A A A G A A G G C T A T C T T C C C A A G A A	14%	20%	26%	12%					11%	19%
APBB1IP-NM_019043.4eU2436-3'UTR+118	3	15%	4%	15%	4%	0			0			370	280	650	430	220	A T T T C A G A T A A A A T G T G A T G G G A A C T T C T C A C T G A	14%	20%	13%							
APBB1IP-NM_019043.4eU2547-3'UTR+229	3	15%	9%	15%	9%	3	29%	5%	0			210	310	730	280	310	G T A T T T T A A C C T A A A T G G A A T G T A T C T T C C C T T C C A	15%	25%	6%		34%	25%	27%			
APMAP-NM_020531.3eU1568-3'UTR+254	3	2%	0%	2%	0%	0			0			320	250	730	330	390	G G A A C A T A A A T T C A T G T A A A G C C A T T T T C T C T T A A A	2%	2%	2%							
ARFGAP2-NM_001242832.2eU1123-NP_001229761.1aa366SL	2	2%	0%	2%	0%	0			0			300	240	780	130	220	T A G A G G A G A A G G A G C C A G A A G T G A C C A T C T C A A G C A	2%		2%							
ARFIP1-NM_001025593.3eU763-NP_001020764.1aa161Q*	3	13%	1%	13%	1%	0			0			340	210	700	290	240	T C G A C C C A G C T T T T C C A G A T G G T A C A T A C C C A A A G G	13%	13%	14%							
ARHGAP11A-NM_001286479.3eU1814-NP_001273408.1aa422HH	4	13%	5%	15%	2%	0			0			320	150	780	300	260	A G C G T T T T C T G A A T C T G G A A G T A A T C T T C A C G C A T T	16%	17%	14%	6%						
ARHGAP11A-NM_001286479.3eU2227-NP_001273408.1aa560PL	2	3%	1%	3%	1%	0			0			220	440	690	320	140	A G A A T G A G A A T A T G A T G G A A G G T A A C T T A C C G A A G T		4%	3%							
ARHGAP29-NM_001328664.2eU417-NP_001315593.1aa68SL	4	41%	12%	47%	2%	0			3	4%	1%	430	210	830	260	230	T G T T A C T A T A T T T G A A A G A A G C A T A T T T T C A G A C T	45%	49%	48%	24%				3%	4%	5%
ARHGEF17-NM_014786.4eU6974-3'UTR+419	4	23%	8%	27%	2%	0			1	7%		250	320	520	500	280	T T T A T G T T T C T T T T T A G A T G T G T A C C T T C C C A A G C A	28%	24%	28%	12%					7%	
ARL1-NM_001177.6eU1226-3'UTR+553	2	6%	2%	6%	2%	0			1	5%		300	190	520	350	330	G C A T G A A T A T T G T T T T G A T G G T T A A C T T C A C T G A C T		4%	7%							5%
ARMCX6-NM_001009584.2eU1201-3'UTR+36	4	10%	4%	12%	3%	0			0			260	210	700	340	320	C C T C T C G A A C A C A A T G G G A C G G A A T C C A C C C A C A A C	13%	9%	14%	5%						
ARMCX6-NR_033669.2eU372-ncr	3	11%	0%	11%	0%	0			0			260	210	700	340	320	C C T C T C G A A C A C A A T G G G A C G G A A T C C A C C C A C A A C	12%	11%	11%							

ARNT-NM_001197325.2eU1108-NP_001184254.1aa359TI	3	9%	4%	9%	6%	0			0			290	160	780	230	400	TCTCCCGACACAACATTGAGGGTATCTTCACTTTTG		13%	5%	10%						
ASAH2B-NM_001079516.4eU550-NP_001072984.1aa158PL	4	49%	28%	61%	17%	0			1	7%		260	300	690	430	160	CTGTCATACTTTCATTTGAAGGCACTTCCCCGGCTT	72%	70%	41%	13%					7%	
ASB16-NM_080863.5eU1914-3'UTR+466	2	22%	11%	30%		0			3	25%	4%	220	110	490	200	200	GGCTTCTTGAGACACCTGCTGACCTCTAGCCGGACC			30%	14%				23%	29%	23%
ASCC3-NM_006828.4eU6781-NP_006819.2aa2157YY	4	5%	1%	4%	1%	0			0			520	230	690	330	180	TACCCCTGAAATACCTGGAAGGTATATCTACACATT	5%	3%	5%	6%						
ASH1L-NM_001366177.2eU4968-NP_001353106.1aa1406PP	3	18%	4%	19%	4%	0			0			210	360	800	260	380	AGGATTAGGTTACTATGGAAGGTATCCTCCCACTCT		22%	16%	14%						
ASXL2-NM_001369346.1eU3253-NP_001356275.1aa1044TI	2	8%	4%	8%	4%	0			0			260	130	810	290	300	ACTCAGGTTTCCAGCTGGAAGACATCTCCACAAGCC		5%	10%							
ATG101-NM_001098673.2eU437-NP_001092143.1aa46SF	2	2%	1%	2%	1%	0			0			350	240	740	400	180	AGTTCCACTACAAGAAGGAGGGCACCTACTCCATTG	1%	2%								
ATG14-NM_014924.5eU986-NP_055739.2aa326PP	4	28%	14%	34%	11%	0			2	7%	1%	300	230	540	490	370	TCTGTCTCATATACTTGATGTAAATCTTCCCAAAAA	45%	24%	32%	12%					7%	6%
ATIC-NM_004044.7eU625-NP_004035.2aa181HY	3	2%	1%	2%	1%	0			0			220	280	770	150	300	AGACGCCAGTTAGCCTTGAAGGCATTCACTCATACG	2%	2%	3%							
ATP13A3-NM_001367549.1eU1382-NP_001354478.1aa286SF	3	8%	4%	10%	3%	0			0			380	260	790	260	310	GAGTAAATGAAGAAATAGAAGAAATCTTTTCTACCG		13%	8%	4%						
ATP5F1A-NM_001001935.3eU1572-NP_001001935.1aa428PP	4	2%	1%	2%	1%	3	28%	3%	0			190	380	620	330	440	TGAGTTGCTGAAGCAAGGACAGTATTCTCCCATGGC	2%	2%	1%	1%	24%	30%	29%			
ATP5PO-NM_001697.3eU505-NP_001688.1aa155SF	4	8%	4%	10%	1%	0			0			380	200	720	210	290	CTGCATCTCCTTTAGAAGAAGCCACACTCTCTGAAT	11%	10%	10%	2%						
ATP6V1D-NM_015994.4eU309-NP_057078.1aa68SL	4	8%	3%	9%	1%	0			3	70%	1%	480	210	700	270	300	TGGGCGAAGTGATGAGAGAAGCTGCCTTTTCACTAG	10%	9%	8%	3%				70%	71%	69%
ATPSCKMT-NM_199133.4eU470-NP_954584.2aa152SL	3	10%	0%	10%	0%	0			0			570	230	520	550	320	ATATTTCAGATTTGTGGAAGGTTACTTTTTCGCAGT	10%		10%	10%						
ATRX-NM_000489.6eU10170-3'UTR+2476	3	10%	4%	10%	4%	0			0			390	220	620	410	180	TTAAGTACTGTAGAAAAGATGTCACTTCTTCCTTTA	6%	15%	10%							
ATXN3-NM_001127696.2eU1694-3'UTR+623	3	27%	11%	27%	11%	0			1	9%		430	170	550	510	230	CACAATTCTTTTGAAAGATGGTAATCTTTTCTGAGG	22%	40%	20%					9%		
AVL9-NM_015060.3eU666-NP_055875.1aa149Q*	4	23%	9%	27%	3%	0			3	19%	2%	220	420	780	450	470	CATGCATATTTTGAAGAGAAGGATTTTTCCCAAATT	30%	28%	23%	10%				20%	16%	20%
B3GALT9-NM_001386823.1eU841-NP_001373752.1aa68SS	3	28%	9%	32%	8%	0			0			340	260	740	470	150	GAACATCGAACCCCTAAGAAGTAATCTCTCCAAATA		26%	38%	19%						
BAD-NM_004322.3eU727-NP_004313.1aa131RC	4	5%	2%	6%	1%	0			0			510	270	730	370	240	TTTGTGGACTCCTTTAAGAAGGGACTTCCTCGCCCG	7%	7%	5%	2%						
BANF1-NM_001143985.1eU464-NP_001137457.1aa5Q*	4	12%	5%	14%	2%	0			1	2%		200	320	740	220	370	AGATTAAGCCTGATCAAGATGACAACCTCCCAAAAG	15%	16%	12%	4%					2%	
BANF1-NM_003860.4eU97-NP_003851.1aa5Q*	4	12%	5%	14%	1%	0			1	1%		200	320	740	220	370	GGATTAAGCCTGATCAAGATGACAACCTCCCAAAAG	16%	13%	14%	5%					1%	
BANF1-XM_017018514.1eU514-XP_016874003.1aa5Q*	3	15%	5%	15%	5%	0			0			200	320	740	220	370	TAATTAAGCCTGATCAAGATGACAACCTCCCAAAAG	19%	16%	10%							
BAX-NM_001291429.2eU1107-3'UTR+448	2	2%	0%	2%	0%	0			0			220	350	710	200	330	GATGTCCCTGTCTCCAGGACGGCCTCCTCTCCTACT		2%	2%							
BBX-NM_001142568.3eU2664-NP_001136040.1aa788SL	4	15%	8%	16%	9%	0			0			430	210	830	220	230	ATGCCATTCACCCTACAGAAGCCATATTTTCAGAAG	27%	12%	9%	11%						
BBX-NM_020235.7eU2574-NP_064620.2aa758SL	4	14%	7%	15%	8%	0			0			430	210	830	220	230	ATGCCATTCACCCTACAGAAGCCATATTTTCAGAAG	24%	9%	13%	11%						
BCAR1-NM_001170714.3eU868-NP_001164185.1aa195SL	3	3%	1%	3%	1%	3	28%	8%	0			280	240	550	350	180	CTCCCCCAGCCAAGCAGACATCCACCTTCTCGAAGC	2%	4%	3%		25%	37%	22%			
BCAS2-NM_005872.3eU792-3'UTR+90	4	5%	2%	6%	1%	3	17%	4%	0			300	360	700	320	250	AATGAACTTTGAAGGACAACAGCATCTTCCCAAAAC	5%	7%	5%	2%	13%	18%	20%			
BCL2L12-NM_001040668.1eU1096-NP_001035758.1aa138PP	4	4%	1%	4%	2%	0			3	60%	5%	290	220	700	240	350	CTTCCTGAGCCGCCTTCGAAGATGTCTTCCCTGCTC	6%	3%	4%	3%				66%	58%	56%
BCL2L12-NM_001282516.1eU1099-NP_001269445.1aa139PP	3	5%	1%	5%	1%	0			3	64%	3%	290	220	700	240	350	CTTCCTGAGCCGCCTTCGAAGATGTCTTCCCTGCTC	5%	4%	4%					67%	64%	61%
BCOR-NM_001123385.2eU792-NP_001116857.1aa3SL	3	26%	15%	26%	15%	0			0			210	120	790	120	440	AAGCTGCCATCCCAGAAGACGACATGCTCTCAGCAA	43%	15%	19%							
BIRC2-NM_001166.5eU1932-NP_001157.1aa171SL	4	5%	1%	6%	1%	0			0			390	140	780	250	150	TTAATTCTAGAGCAGTTGAAGACATCTCTTCATCGA	5%	6%	5%	4%						
BLMH-NM_000386.4eU1294-NP_000377.1aa390TI	4	10%	4%	12%	2%	0			3	63%	1%	300	270	640	380	310	CAGAGAAGGATGATCAGGATGGTGCTTTCACAAAAT	14%	9%	12%	5%				62%	62%	64%
BMP1-NM_006129.5eU3253-3'UTR+258	4	3%	1%	3%	1%	0			0			320	280	680	220	110	CTTCCACAAGACATTTCGAAGTCATCATTCCTCTCT	4%	5%	2%	3%						
BNIP3-NM_004052.4eU1380-3'UTR+678	4	38%	10%	43%	5%	0			3	7%	3%	270	170	830	190	100	ATGATCAGTTTTCTGCTGAAGGCACCTACTCAGTAT	48%	38%	42%	26%				4%	6%	10%
BOK-NM_032515.5eU2362-3'UTR+1445	2	2%	0%	2%	0%	0			0			240	450	630	320	240	ACATTTCTAGGCACATGTGAGGCATCTTTCCTGGAG		2%	2%							
BRD9-NM_001009877.3eU1383-NP_001009877.2aa413PP	2	3%	1%	3%	1%	0			0			210	220	820	320	490	CTTCCAGCTGAAGCAGAGAAGAAATGTTCCCATGAA		2%	4%							
BUB1B-NM_001211.6eU2547-NP_001202.5aa799Q*	4	9%	6%	11%	4%	0			0			230	330	830	260	350	GCAGAATTAACAGTAATAAAGGTATCTTCTCAACCT	16%	9%	8%	2%						
BUB3-NM_001007793.3eU140-NP_001007794.1aa19SF	4	6%	2%	7%	0%	0			1	1%		280	190	800	330	170	TGAACCAGCCACCCGAGGATGGCATCTCCTCCGTGA	6%	7%	6%	3%				1%		
C14orf119-NM_017924.4eU797-3'UTR+258	2	2%	0%	2%	0%	0			0			280	130	740	210	300	TTTGCTTCTTTCCGAGAGATGTCAAGTCCTCAAGAA	2%		2%							
C16orf72-NM_014117.3eU1323-3'UTR+94	4	7%	3%	8%	2%	0			0			290	140	780	410	250	AATTGCAAAGGAAACATGAGGCCATCTTCCCTTGTT	8%	7%	10%	4%						
C1QTNF5-NM_001278431.2eU915-NP_001265360.1aa228SF	2	27%	22%	27%	22%	3	64%	11%	0			350	300	750	150	260	ATGCCAGCATCAAGACAGACAGCACCTTCTCCGGAT		43%	12%		54%	63%	76%			
C1R-NM_001354346.2eU2020-NP_001341275.1aa648SF	2	2%	1%	2%	1%	0			3	25%	4%	540	110	490	350	550	TCCGGGGAAAGAATAGGATGGATGTGTTCTCTCAAA	3%	2%						22%	29%	24%
C2CD5-NM_001286174.3eU1119-NP_001273103.1aa358PS	3	13%	9%	17%	6%	0			1	4%		280	250	750	280	230	TTTCCATTTTTTACCTTGACGGCATTTCCTCCTGGA		13%	21%	4%				4%		
C8orf33-NM_023080.3eU919-3'UTR+204	2	2%	0%	2%	0%	0			0			410	70	730	220	290	GACCCCGTTCGTTTTCTGATGAAATGTTCTCTCTTT		2%	2%							
C9orf40-NM_017998.3eU993-3'UTR+107	4	29%	12%	35%	7%	0			0			370	330	710	280	170	AGTTATTTTCCATACTTGAAGTTATATTTCCAAACC	30%	32%	43%	13%						
CAMTA2-NM_001171166.2eU2604-NP_001164637.1aa853SF	4	7%	3%	7%	3%	0			0			340	320	730	310	40	CGCCCTCGGAGCTGTCGGATGGCACCTTTTCCGTCA	11%	5%	6%	5%						
CARHSP1-NM_001042476.2eU515-NP_001035941.1aa52SL	2	2%	1%	2%	1%	0			0			480	280	510	360	300	CACTGCCCACTCGCCGGACGAGGACCTTCTCGGCGA	3%		2%							
CARHSP1-NM_001042476.2eU888-3'UTR+84	4	2%	1%	2%	1%	0			0			370	310	680	290	480	AGGCAGCAGACACTGGAGATGACATTCTTCCACACG	2%	3%	3%	2%						
CASC3-NM_007359.5eU3702-3'UTR+1578	3	8%	0%	8%	0%	0			3	77%	3%	360	130	730	210	310	CCCTTTGCCTTAAACCTGAAGATGTCTCCTCAAGCC	8%	9%	8%					78%	73%	79%
CASP10-NM_001206524.2eU558-NP_001193453.1aa128SL	2	15%	9%	21%		0			0			220	230	810	150	310	TGCTCTACGAACTGTCAGAAGGCATTGACTCAGAGA			21%	9%						
CASP2-NM_001224.5eU1427-3'UTR+400	4	9%	4%	11%	1%	0			0			260	70	710	400	100	AGGACACCCTCCCACATGATGTCACCTCCCCATCAT	9%	11%	12%	3%						
CASZ1-NM_001079843.3eU1614-NP_001073312.1aa433SF	2	7%	1%	8%		0			0			400	260	550	520	220	ACACTCCCGAGTACCTGAAGTCAACCTTCTCCAAAA		8%		6%						
CAVIN2-NM_004657.6eU905-NP_004648.1aa248RC	3	5%	0%	5%	0%	3	50%	4%	0			410	340	800	210	350	AAAGTGGATAGCCTCAAGAAAGCATTTTCTCGCCAG	5%	6%	5%		47%	49%	55%			
CBX5-NM_001127321.1eU7552-3'UTR+6856	2	19%	13%	19%	13%	0			0			280	350	700	470	250	CTTTGGTTGTTTTCTAAGATGGAATTTTTCCTGTTC		28%	10%							
CBX6-NM_001303494.2eU3119-3'UTR+1908	4	13%	6%	16%	3%	0			3	77%	5%	390	210	710	220	290	CGTGACTTTTTTGTAAAGAAGTTGTGTTTTCAGAGG	18%	16%	13%	5%				83%	73%	77%
CBY1-NM_001002880.4eU337-NP_001002880.3aa16RW	4	12%	6%	15%	5%	0			1	7%		410	80	780	300	260	AATACGTTCAGTCCGAAGAAGACACCTCCTCGGAAG	17%	18%	9%	6%				7%		
CBY1-NM_015373.4eU197-NP_056188.1aa16RW	4	27%	11%	32%	5%	0			0			410	80	780	300	260	AATACGTTCAGTCCGAAGAAGACACCTCCTCGGAAG	31%	37%	28%	12%						
CCDC186-NM_001321829.1eU5740-3'UTR+2651	3	23%	8%	26%	8%	0			0			430	310	700	330	170	GTGGTTTATTTGAGCTGGAAGTGATTTTTTCTGTGT	21%		32%	16%						
CCDC43-NM_001099225.2eU1675-3'UTR+1187	3	4%	1%	4%	1%	3	41%	0%	0			400	340	660	290	240	TTGTCTAGAAGCCTAAGGACAGCAATTTCTCTGACA		4%	3%	4%	41%	42%	41%			
CCDC71L-NM_175884.6eU1351-3'UTR+381	2	5%	3%	5%	3%	3	22%	6%	0			370	350	620	350	310	TCTGGAAGTCCTCCGAAGACATTATTTTTCCAAAGG	7%	3%			17%	28%	20%			
CCND1-NM_053056.3eU227-NP_444284.1aa23LL	3	5%	1%	5%	1%	0			0			360	90	460	550	220	CCGCCGCGCGTACCCCGATGCCAACCTCCTCAACGA	6%	6%	4%							
CCNE2-NM_057749.3eU118-NP_477097.1aa23Q*	4	19%	7%	23%	1%	0			0			160	260	740	330	320	CAGCCCCAGCCCAGCCAGACGGAATCCCCCCAAGAA	24%	23%	22%	8%						
CCNF-NM_001323538.2eU2173-NP_001310467.1aa441Q*	2	4%	1%	4%	1%	0			0			240	210	750	300	160	TGCCACCATCAGGCCAGGAAGTCATGTTTACAGTGT	5%		4%							
CCP110-NM_001199022.3eU779-NP_001185951.2aa206SL	3	14%	6%	14%	6%	0			3	6%	1%	270	200	730	190	250	AAGAAACTCTTATTTCTGATGGTCCCTTCTCAGTAA	14%	20%	8%					6%	7%	5%
CCT6A-NM_001009186.2eU1653-3'UTR+108	4	1%	0%	1%	0%	3	5%	1%	0			260	320	700	550	390	AGCTTCAAATAATTTTGAAAGAAATTTTCCCATATG	2%	1%	1%	1%	5%	5%	4%			
CCT6P1-NR_003110.2eU321-ncr	2	6%	1%	6%	1%	0			0			240	180	600	530	290	AGATCTCTACATTTCTGAAGGCCTTCATCCCAGAAT		7%	5%							
CD44-NM_000610.4eU642-NP_000601.3aa170PL	4	3%	1%	3%	1%	0			0			210	300	760	240	250	AATACAGAACGAATCCTGAAGACATCTACCCCAGCA	3%	2%	3%	2%						
CD44-NM_001001391.2eU642-NP_001001391.1aa170PL	3	3%	1%	3%	1%	0			0			210	300	760	240	250	AATACAGAACGAATCCTGAAGACATCTACCCCAGCA	4%	2%	2%							
CD55-NM_000574.5eU503-NP_000565.1aa139LL	2	3%	1%	3%		0			0			470	290	590	520	220	TGCCGTCCAGGTTACAGAAGAGAACCTTCTCTATCA		3%		2%						
CDC25A-NM_001789.3eU3224-3'UTR+1179	3	3%	1%	3%	1%	3	32%	4%	0			310	260	730	330	270	TGGCATGGACATGACTGGATAGAATTTTCTCAGGAG	1%	4%	4%		30%	36%	29%			
CDC40-NM_015891.3eU849-NP_056975.1aa277PP	2	6%	2%	6%	2%	1	1%		0			180	310	670	280	600	AACTATGCCACCTGAGAAGTGTTATCTTCCCAAAAA		7%	4%		1%					
CDC42EP1-NM_152243.3eU694-NP_689449.1aa103PS	4	3%	1%	4%	1%	0			0			220	260	760	430	170	GGGGCGCCCCCCCGGAGGATGGCATCTCCCCTGCA	4%	2%	4%	1%						
CDC42EP3-NM_001270436.2eU2327-3'UTR+1224	3	10%	1%	10%	1%	0			0			210	120	740	170	250	TTTTTTAAGTCAGCACTGATGCCACCCTCTCAGTGG	9%	11%	10%							
CDC42-NM_001039802.2eU1359-3'UTR+573	2	1%	0%	1%	0%	3	5%	1%	0			290	410	720	200	370	TTCCTAGATCTAGTTTAGAAAACATGTTCCCCATCT	1%	1%			6%	5%	4%			
CDC42-NM_001039802.2eU626-NP_001034891.1aa139PL	3	2%	1%	2%	1%	0			0			360	170	420	580	250	TTGCCAAGAACAAACAGAAGCCTATCACTCCAGAGA	3%	1%	2%							
CDC73-NM_024529.5eU2004-3'UTR+238	2	6%	2%	6%	2%	0			0			290	270	610	480	310	AATTGTATATTTTGATAGAAGTTTTTCTCCATTGG	8%	5%								
CDCA7-NM_031942.5eU776-NP_114148.3aa228LL	2	2%	0%	2%		0			0			410	230	730	230	320	CCCTGGCTCGTTCCGTGGAAGACATCCCCTCCCAGG			2%	2%						
CDCP1-NM_022842.5eU1734-NP_073753.3aa548TM	4	7%	3%	8%	1%	0			3	46%	3%	210	300	730	330	320	TACCTTATTTCAAAGAGGAAGGCGTTTTCACGGTGA	10%	8%	7%	3%				43%	49%	46%
CDH2-NM_001792.5eU276-NP_001783.2aa56LL	3	3%	1%	3%	1%	3	40%	2%	0			310	290	740	220	300	GAAGGATGTGCATGAAGGACAGCCTCTTCTCAATGT	3%	4%	2%		39%	42%	39%			
CDK2-NM_001798.5eU394-NP_001789.2aa72TI	4	4%	1%	4%	0%	0			0			160	110	650	230	350	ATATTGTCAAGCTGCTGGATGTCATTCACACAGAAA	4%	5%	4%	2%						
CDK5R1-NM_003885.3eU956-NP_003876.1aa214SF	4	12%	4%	10%	2%	0			0			330	100	720	290	350	TCTACATGCTCTGCAGGGATGTTATCTCCTCCGAGG	13%	10%	8%	18%						

CENPJ-NM_018451.5eU190-NP_060921.3aa8SL	2	7%	1%	7%	1%	0			0			480	100	390	590	120	GGACTAAGATGTTCCTGATGCCAACCTCTTCAGAGT	8%	6%								
CENPJ-NM_018451.5eU3964-NP_060921.3aa1266PL	3	8%	3%	8%	3%	3	28%	2%	0			300	410	810	310	350	TTCCTGATGGACAAGAAGAAAGCATTTTCCCAGATG	5%	11%	7%		25%	29%	30%			
CENPO-NM_001199803.3eU2536-3'UTR+1602	3	18%	4%	18%	4%	0			0			420	80	840	250	250	CCTTGTTATGGGCCTCAGAAGCCATCTCCTCTCCAG	22%	15%	16%							
CEP120-NM_001375405.1eU468-NP_001362334.1aa21PP	3	16%	8%	21%	1%	0			0			230	270	680	460	270	CGTGTCCATCCTAGAAGGTCGGCATTTCCCCAAACG		20%	22%	7%						
CEP192-NM_032142.4eU5326-NP_115518.3aa1749SL	2	8%	2%	8%	2%	0			0			350	200	730	320	210	AGGTAGTGTTAAAAGGCGAAGTCATTTCTTCAGGAA	9%	7%								
CEP57L1-NM_001083535.3eU354-NP_001077004.1aa44PL	4	27%	9%	31%	5%	0			0			420	260	630	400	140	GCCATCCTGCAAACTTAGAAGTTACCTCTCCTAAGA	36%	27%	30%	14%						
CEP57L1-NM_001271852.3eU203-NP_001258781.1aa44PL	3	38%	21%	38%	21%	0			0			420	260	630	400	140	GCCATCCTGCAAACTTAGAAGTTACCTCTCCTAAGA	63%	26%	25%							
CEP57L1-NM_001350652.2eU458-NP_001337581.1aa44PL	2	32%	7%	32%	7%	0			0			420	260	630	400	140	GCCATCCTGCAAACTTAGAAGTTACCTCTCCTAAGA	37%	27%								
CFL1-NM_005507.3eU785-3'UTR+203	4	1%	0%	1%	0%	0			3	4%	1%	350	230	480	290	220	CCCTCCATCCCTTGACGGTTCTGGCCTTCCCAAACT	1%	1%	1%	0%				4%	4%	5%
CHAC2-NM_001008708.4eU578-NP_001008708.1aa165FF	4	24%	4%	26%	3%	2	6%	1%	0			520	300	540	390	190	TGTGCCAGAAGAAGCAGATGAGCATCTTTTCGCTTT	24%	29%	26%	19%	7%		5%			
CHAMP1-NM_001164144.3eU2989-3'UTR+234	4	7%	2%	8%	1%	0			0			190	310	640	180	230	TTCTGTATAGTCCAGATGGATTAAACTTCTCATTTC	8%	8%	9%	5%						
CHCHD3-NM_001317177.2eU274-NP_001304106.1aa41PS	4	7%	3%	9%	2%	0			0			170	440	790	200	320	AATGTGATTGATCGAATGAAGGAATCCTCTCCATCT	9%	7%	9%	3%						
CHCHD3-NM_017812.4eU274-NP_060282.1aa41PS	3	9%	5%	9%	5%	0			0			170	440	790	200	320	AATGTGATTGATCGAATGAAGGAATCCTCTCCATCT	14%	9%	4%							
CHSY1-NM_014918.5eU3917-3'UTR+916	2	2%	0%	2%	0%	0			0			230	220	690	170	430	TTAAATTTCAAGAAGGATTTGGCATTTTCTCTTCTT	2%		2%							
CIART-NM_001300838.2eU1274-NP_001287767.1aa383LL	2	17%	0%	17%	0%	0			0			270	160	620	310	330	TCCTCCAGTTGCTGCTGATGCTCATCTTCTCAACCT	16%		17%							
CKAP2L-NM_001304361.2eU793-NP_001291290.1aa116HY	3	8%	1%	8%	1%	0			3	7%	5%	310	210	660	310	310	GGAGTAAAACCCTCAAGGACGGTTCCCTCTCACTTT	9%	7%	7%					13%	3%	4%
CLINT1-NM_001195555.2eU1600-NP_001182484.1aa475LL	4	7%	2%	7%	0%	0			0			170	290	500	160	650	TAGCACAGTGCTGCAGAAGCCTAATCCTCTCTATAA	7%	7%	7%	4%						
CLNS1A-NM_001293.3eU658-NP_001284.1aa193SF	4	3%	1%	3%	1%	0			0			430	350	710	200	260	CCACACTGGAGAGATTAGAAGGAATGCTTTCTCAGT	3%	5%	2%	2%						
CLNS1A-NM_001293.3eU660-NP_001284.1aa194Q*	3	2%	1%	2%	1%	0			3	22%	1%	470	290	430	350	710	ACACTGGAGAGATTAGAAGGAATGCTTTCTCAGTCT	2%	1%	1%					21%	24%	22%
CMC4-NM_001018024.3eU133-5'UTR-322	2	13%	5%	16%		0			0			310	50	760	250	330	GGGGGACAGCCCTTTAAGACGACATCTCCGCGAACG	16%			10%						
CMPK2-NM_207315.4eU2764-3'UTR+1203	2	6%	2%	6%	2%	0			0			320	320	660	400	290	TATCTACCATTTCTTAAGAAGACATTACTCCAAAAT		7%	4%							
CMTR1-NM_015050.3eU408-NP_055865.1aa82SF	3	2%	1%	3%	1%	0			0			530	290	620	530	60	AAGCAGACTCTCTTGTGGAAGGAACTTCTTCTCGCT		3%	2%	2%						
CMTR1-NM_015050.3eU410-NP_055865.1aa83RC	2	4%	1%	4%	1%	0			0			490	230	530	290	620	GCAGACTCTCTTGTGGAAGGAACTTCTTCTCGCTAT	5%	4%								
CNIH1-NM_005776.3eU2507-3'UTR+2025	3	39%	20%	39%	20%	0			0			230	210	860	330	270	ACTGGTTGGGATTTGTGGAAGCCATTTTCTCATGTT	62%	24%	32%							
CNKSR3-NM_001368116.1eU603-NP_001355045.1aa99HY	2	19%	10%	19%	10%	3	41%	8%	0			480	270	780	290	290	AACTTGGTTCTGAAACTGAGAGCATCTTCCCACAAT	26%		12%		39%	50%	35%			
CNOT1-NM_001265612.2eU2396-NP_001252541.1aa708PL	4	4%	1%	4%	0%	0			0			330	190	640	460	210	CTAGTGCTAGCAGCTTAGATGCCATTTCTCCTGTTC	4%	4%	4%	2%						
CNOT6-NM_001370472.1eU4251-3'UTR+2190	4	32%	10%	37%	1%	1	7%		2	5%	1%	350	130	800	300	110	TGTATTTGTTAATGGAGGATGTCATCTTTTCATAGA	36%	38%	39%	17%			7%		5%	6%
CNPPD1-NM_001321389.1eU472-NP_001308318.1aa15SF	4	35%	9%	39%	3%	2	17%	2%	3	37%	10%	370	340	880	230	220	GGCTCCTGCTGGACGAAGAAGGCACCTTCTCCCTCG	37%	42%	38%	22%	16%		19%	41%	44%	25%
CNPPD1-NM_015680.6eU141-NP_056495.4aa15SF	4	33%	14%	40%	5%	2	15%	2%	2	25%	3%	370	340	880	230	220	GGCTCCTGCTGGACGAAGAAGGCACCTTCTCCCTCG	43%	35%	42%	12%		16%	13%	28%		23%
CNPY2-NM_001190991.3eU720-NP_001177920.1aa57RW	4	21%	10%	25%	3%	0			1	7%		380	330	750	280	190	CCCAAGAAGACCATTCAGATGGGATCTTTCCGGATC	22%	26%	28%	6%						7%
CNPY2-NM_014255.7eU362-NP_055070.1aa57RW	4	24%	9%	29%	0%	1	2%		2	3%	1%	380	330	750	280	190	CCCAAGAAGACCATTCAGATGGGATCTTTCCGGATC	29%	29%	29%	11%			2%	2%	3%	
CNPY3-NM_001318842.1eU1348-NP_001305771.1aa303LF	4	19%	6%	22%	1%	0			1	4%		410	220	810	150	320	GAGGATGAGGGCATCCAGAAGGCATCCCCTCTCACA	23%	22%	20%	10%					4%	
COL5A1-NM_000093.5eU6316-3'UTR+414	3	3%	1%	3%	1%	0			0			350	260	420	590	410	CTAAATTCAACTTGAAGATGTGTATTTCCCCTGACC	3%	3%	2%							
COL6A1-NM_001848.3eU3255-3'UTR+87	4	4%	1%	4%	1%	0			0			210	380	480	560	390	AAACAGAGTAAAATGTGATGCGAATTTTCCCGACCA	3%	5%	5%	3%						
COL8A1-NM_001850.5eU2222-NP_001841.2aa654TI	3	3%	1%	4%	0%	0			0			130	380	700	300	300	AGAACTACAACCCGCAGACAGGCATCTTCACCTGTG		4%	4%	3%						
CPSF7-NM_001136040.4eU1585-3'UTR+52	4	3%	1%	3%	1%	3	7%	1%	0			340	130	600	290	290	AAATGTTTTTTTAATGGACTTGCATCTCCTCACCTT	4%	2%	3%	2%	6%	8%	8%			
CREBL2-NM_001310.4eU2718-3'UTR+2058	2	6%	2%	6%	2%	0			0			390	110	530	410	430	CGTCTTTATTGTGCTTGAAGTATTTCTTCTCTGGTT		8%	4%							
CRKL-NM_005207.4eU1516-3'UTR+76	3	16%	2%	16%	2%	3	56%	3%	0			500	340	760	250	350	AGTCTCCTTTGAAGTGGGAAAGCATTTTCTCTCATA	14%	18%	17%		56%	60%	54%			
CSE1L-NM_001256135.2eU1172-NP_001243064.1aa350SF	2	1%	0%	1%	0%	3	4%	1%	0			230	280	700	340	250	TTTTTGAGGGACCTGTGACAGGAATCTTCTCTGGTT		1%	1%		4%	3%	4%			
CSF1-NM_000757.6eU437-NP_000748.4aa88LL	4	13%	6%	15%	3%	0			0			330	360	720	460	210	CCAGTGTGCTACCTTAAGAAGGCATTTCTCCTGGTA	15%	12%	18%	5%						
CSTF2-NM_001306206.2eU136-NP_001293135.1aa37SF	4	10%	3%	11%	2%	0			0			510	170	670	430	270	CTACTGAAGAGCAGTTGAAGGACATCTTTTCTGAGG	13%	11%	9%	7%						
CTBP1-NM_001012614.2eU1934-3'UTR+241	4	13%	7%	16%	2%	0			0			70	330	670	210	480	TTAAGCAGAAGAAGTCAGTAGTTATTCTCCCATGAA	18%	14%	17%	3%						
CTBP2-NM_001083914.3eU1773-3'UTR+26	4	5%	1%	5%	0%	0			0			220	360	710	150	370	AATAGCAGAGAATGCCAGAAGGTAATCACTCAGATA	5%	6%	5%	3%						
CTDP1-NM_001202504.1eU579-NP_001189433.1aa150HY	3	12%	9%	12%	9%	0			2	12%	2%	330	210	780	350	410	TTTTTAGACCCCGAGAAGAAGCTTTTTTCTCACCGA	22%	6%	8%					11%		14%
CTDSPL2-NM_016396.3eU2942-3'UTR+1373	2	5%	1%	5%	1%	0			0			430	370	600	500	180	TGTAAAGGATGTCAAAGAACGGCACTTTTTCTAAAG	6%		4%							
CTNNA1-NM_001290307.3eU3348-3'UTR+734	4	28%	8%	32%	2%	3	1%	0%	3	7%	1%	220	310	900	240	370	AGGAAGCAGTCCCTGGAGAAGGCATGTTCCCAGAAA	34%	30%	34%	16%	1%	2%	1%	6%	8%	8%
CTTN-NM_001184740.2eU2120-3'UTR+32	2	9%	8%	15%		0			0			270	340	610	380	440	GCGTGTTTTTGCTCCTGAGGTGCATTTTCTCATCAT			15%	4%						
CTTN-NM_005231.4eU3114-3'UTR+1278	4	5%	1%	4%	1%	0			0			270	340	610	380	440	GCGTGTTTTTGCTCCTGAGGTGCATTTTCTCATCAT	3%	5%	4%	6%						
CUEDC2-NM_024040.3eU157-NP_076945.2aa33SF	2	3%	2%	3%	2%	0			0			360	170	820	270	250	ACCTCAGTGGCTTGGATGAGGTCATCTTCTCCTATG	4%		2%							
CYB561A3-NM_001161452.2eU1095-NP_001154924.1aa165SF	2	6%	2%	6%	2%	0			0			270	120	710	80	440	TCCACGTCTTTTTTGGAGCCGCCATCCTCTCTCTGT	8%	5%								
CYBRD1-NM_001127383.2eU1367-3'UTR+821	4	7%	4%	9%	3%	0			0			400	190	820	140	290	GATTAGAGCAGGTGGTTGAAGAGATCTTCTCTGGTC	13%	8%	6%	2%						
CYP51A1-NM_000786.4eU1571-NP_000777.1aa486PP	4	16%	4%	18%	2%	0			2	3%	1%	390	240	520	570	420	TGAATTTGATCTCATTGATGGATACTTTCCCACTGT	15%	19%	19%	10%				2%	4%	
CZIB-NM_001304759.2eU788-3'UTR+303	4	25%	11%	30%	4%	0			3	7%	2%	220	160	810	310	390	AAGAGTATTTTTAAGGAGATGGTATCTCCCCAGTCA	28%	28%	35%	10%				8%	8%	5%
DAD1-NM_001344.4eU336-NP_001335.1aa90PL	4	3%	1%	3%	1%	0			0			270	240	770	310	170	AGAACAAAGCGGATTTCCAAGGCATCTCCCCAGAGC	4%	3%	3%	2%						
DDHD2-NM_001164232.2eU3536-3'UTR+1200	2	6%	1%	6%	1%	0			0			400	300	800	180	190	CTGCACACTAATTTTGTCAAGGCATCTTTTCACTAC		5%	7%							
DDX21-NM_001256910.2eU581-NP_001243839.1aa121SF	4	23%	6%	26%	2%	0			3	68%	1%	400	350	750	300	320	TACCTGTGGAACAAAAAGAAGGCGCTTTCTCTAATT	28%	25%	23%	15%				68%	68%	66%
DDX46-NM_001300860.2eU1839-NP_001287789.1aa560SL	4	24%	10%	30%	2%	0			2	3%	1%	340	170	550	430	250	TTCGTCCTGATCGACAGACGGTTATGTTTTCAGCTA	27%	32%	30%	9%				3%		4%
DDX46-NR_125341.2eU1812-ncr	3	28%	7%	28%	7%	0			0			340	170	550	430	250	TTCGTCCTGATCGACAGACGGTTATGTTTTCAGCTA	33%	19%	30%							
DDX50-NM_024045.2eU489-NP_076950.1aa140SF	4	7%	3%	8%	3%	0			3	46%	7%	360	390	760	310	260	TAACACGTGAACAGAAAGAAGGAGCCTTCTCCAATT	11%	7%	5%	5%				42%	44%	54%
DDX54-NM_001111322.2eU1976-NP_001104792.1aa650SL	4	11%	5%	12%	5%	0			1	2%		400	130	820	380	390	AGGCGGGAGAGAGTGTGGAGGACATTTTCTCAGAGG	16%	14%	7%	8%				2%		
DDX5-NM_001320595.2eU560-NP_001307524.1aa102PL	4	4%	2%	5%	1%	0			0			210	360	710	390	200	CAGTTCTAAATTTTTATGAAGCCAATTTCCCTGCAA	6%	6%	4%	1%						
DEPDC7-NM_001077242.2eU308-NP_001070710.1aa77SF	2	20%	5%	20%	5%	0			0			490	190	700	330	170	TTGGTTCAGAAGCTGTGGATGTCATTTTTTCTCACC		24%	17%							
DHCR7-NM_001163817.2eU1876-3'UTR+236	4	12%	4%	14%	2%	0			0			370	510	560	250	440	TGCCGTATTGCCAGCACAGACGGATTTTCTCTAATC	12%	16%	15%	6%						
DKK1-NM_012242.4eU125-5'UTR-31	4	11%	5%	14%	1%	1	1%		3	2%	0%	310	170	610	420	350	CCCAGGCTTGCAAAGTGACGGTCATTTTCTCTTTCT	14%	13%	14%	5%		1%		2%	2%	3%
DLGAP5-NM_001146015.2eU987-NP_001139487.1aa288SL	3	3%	2%	3%	2%	0			3	38%	1%	320	210	760	220	190	CAAGTGGAATGAATCCAGATGGAGTCTTATCAAAAA	5%	2%	2%					38%	39%	36%
DMTF1-NM_001142326.2eU1955-NP_001135798.1aa525PL	2	4%	1%	4%	1%	0			0			260	280	720	360	360	CTCCAGACGCCCTAGAAGCAGACACTTTCCCAGATG		4%	3%							
DNAI4-NM_024763.5eU2083-NP_079039.4aa686HH	3	17%	7%	17%	7%	0			0			380	160	700	430	380	TTTGGCTGGCACTGAAGAAGGTCATATTCACAAATG	21%	10%	21%							
DNAJA2-NM_005880.4eU368-NP_005871.1aa89SF	4	7%	2%	8%	1%	0			0			460	130	730	290	450	GCGGAGGTGGTGGCATGGATGATATTTTCTCTCACA	9%	8%	7%	4%						
DNAJB1-NM_001300914.2eU1104-NP_001287843.1aa223PL	4	24%	5%	26%	1%	1	1%		3	2%	0%	310	360	780	240	210	ACCTCATTATTGAGTTTGAAGTGATCTTCCCCGAAA	26%	27%	25%	16%	1%			1%	2%	2%
DNAJB1-NM_001300914.2eU695-NP_001287843.1aa87HY	4	2%	1%	3%	0%	0			0			300	330	650	370	430	AGCGGCTGTACCAAGAAGATGAAAATCTCCCACAAG	3%	2%	3%	1%						
DNAJC13-NM_001329126.2eU1462-NP_001316055.1aa397SL	4	20%	6%	22%	2%	0			2	20%	9%	280	200	830	180	400	TACATGCAGTAACACAAGATGGTCTCTTCTCAGAAA	21%	25%	21%	11%				26%		14%
DNAJC3-NM_006260.5eU2805-3'UTR+1173	4	55%	23%	67%	2%	1	8%		3	23%	6%	320	90	840	250	250	GTTGTTTCATTTAATAAGATGGCATCTCCTCTTGAG	69%	65%	66%	21%	8%			27%	25%	16%
DNM2-NM_001005360.3eU391-NP_001005360.1aa76SL	3	3%	0%	3%	0%	0			0			360	120	700	270	290	GGCGGCCTCTCATTCTGCAGCTCATCTTCTCAAAAA	3%	2%	3%							
DPY19L4-NM_181787.3eU1932-NP_861452.2aa611LL	4	12%	8%	14%	9%	0			0			440	240	580	310	330	TTTGCCTCTTTACAATGATGATGATCTTCTCAAGAG	6%	23%	12%	5%						
DR1-NM_001938.3eU1241-NP_001929.1aa168Q*	4	5%	1%	6%	1%	0			0			280	280	830	100	380	GCCAGTGCATCTAATCAGGCGGGATCTTCTCAGGAT	5%	6%	7%	4%						
DSTYK-NM_015375.3eU1504-NP_056190.1aa446PL	2	7%	0%	7%	0%	0			0			200	200	700	290	390	CTAACATGGAGTTTAAAGACGTCATTGTCCCTGAGA		7%	7%							
DUSP12-NM_007240.3eU289-NP_009171.1aa91LL	2	3%	1%	2%		0			0			320	330	540	270	270	AGCGCTGGACAAACCCGAGACGGACCTACTCAGCCA		2%		4%						
DYNC2I2-NM_052844.4eU566-NP_443076.2aa149LL	3	2%	0%	2%	0%	0			3	27%	1%	310	160	710	140	250	TGGACCGAGCAGCAGCAGATGGTGTCTTGTCTGTAT	2%	2%	1%					26%	28%	27%
EBNA1BP2-NM_001159936.1eU328-NP_001153408.1aa60PL	2	4%	1%	4%	1%	0			0			380	220	380	620	390	GACGTGCGTGCGGCGAGATGGACACTCCCCCGCTCT	4%	3%								
EBNA1BP2-NM_006824.3eU186-NP_006815.2aa5PL	4	1%	1%	2%	0%	0			0			380	220	380	620	390	GACGTGCGTGCGGCGAGATGGACACTCCCCCGCTCT	2%	2%	1%	1%						
EDC4-NM_014329.5eU2414-NP_055144.3aa735SL	4	36%	13%	42%	6%	1	9%		3	9%	4%	280	0	810	210	170	GCCGCACTCGTTCCCCTGATGTCATCTCCTCAGCTT	47%	35%	44%	19%			9%	14%	6%	7%

EGFR-NM_001346897.2eU408-NP_001333826.1aa49LL	4	20%	11%	24%	9%	0		0			390	210	650	420	350	G C A G T T G G G C A C T T T T G A A G A T C A T T T T C T C A G C C T	33%	24%	15%	9%				
EID2-NM_153232.4eU747-NP_694964.3aa222PL	2	4%	2%	4%	2%	0		0			250	150	650	180	230	T A G C T C T G A C T G C C T C T G A A G T T A T C A A C C C T C T G A		2%	5%					
EIF1AD-NM_001242481.2eU571-NP_001229410.1aa69LL	2	5%	1%	6%		0		0			440	260	800	350	440	G A A C A T C T G G A T C A A G A G A G G G A C T T T C T C A T T G T		6%		4%				
EIF2AK2-NM_001135651.3eU1767-NP_001129123.1aa504SL	4	21%	9%	26%	1%	0		3	5%	2%	220	160	840	160	290	T T T T C A C A G A C C T A C G G G A T G G C A T C A T C T C A G A T A	26%	26%	25%	8%		6%	7%	3%
EIF2AK2-NM_001135651.3eU882-NP_001129123.1aa209SL	4	6%	3%	7%	1%	0		3	47%	4%	270	320	770	140	200	C T T C T G A A T C A T C A T C T G A A G G T G A C T T C T C A G C A G	7%	6%	8%	2%		45%	46%	52%
EIF2A-NM_001319043.2eU1206-NP_001305972.1aa397PL	4	4%	1%	4%	1%	0		0			320	290	710	440	110	C T T G G C A G C C A T T T T T G G A T G G A A T A T T T C C A G C A A	5%	5%	3%	2%				
EIF2S3-NM_001415.4eU110-NP_001406.1aa33HY	4	10%	4%	12%	1%	0		2	1%	1%	310	130	720	300	250	T T G G A T G T T A C C A A G T T G A C G C C A C T T T C A C A C G A A	12%	11%	12%	4%		1%		2%
EIF3J-NM_001284335.2eU1268-3'UTR+605	3	2%	0%	2%	0%	0		0			360	320	450	500	490	A A C T T T G A A G C T A A A G A A G G T A A A C C T T T C C A T T A T		2%	2%	1%				
EIF4G1-NM_001194946.2eU3101-NP_001181875.2aa981RC	3	2%	0%	2%	0%	0		3	48%	2%	340	220	710	220	410	A A A A T C A T T A A A G A A A A A G A A G A C G T C A T C C C G C A T C	2%	1%	2%			49%	46%	49%
EIF5A-NM_001970.5eU367-NP_001961.1aa74PL	3	2%	0%	2%	0%	0		0			210	150	720	280	310	T T A C T G G G A A G A A A T A T G A A G A T A T C T G C C C G T C A A	2%	2%	1%					
ELAVL1-NM_001419.3eU543-NP_001410.2aa127SF	4	17%	6%	20%	2%	0		2	2%	0%	410	150	850	140	370	T G A C C C A G A A G G A C G T A G A A G A C A T G T T C T C T C G G T	22%	18%	19%	8%		2%	2%	
ELL2-NM_012081.6eU1079-NP_036213.2aa317PS	4	2%	1%	2%	1%	0		0			190	340	610	60	560	G T A T G T T C T A G T A G A G A C G C T G T A T C T T C T C C T C A G	3%	2%	1%	1%				
EMC1-NM_001271427.2eU3137-3'UTR+143	2	2%	0%	2%	0%	0		0			570	230	470	200	520	A A G A A C T G T A G G G A A G A T G G A T G A C C T T C A C G C A G A	2%	2%						
EMP1-NM_001423.3eU1297-3'UTR+634	4	4%	2%	4%	2%	0		1	3%		700	210	620	520	240	G G G T T A G C T A A A C C A A G A A G G A G A C C T T T C A C A A T	4%	3%	6%	2%		3%		
ENGASE-NM_001042573.3eU4196-3'UTR+1843	2	32%	8%	38%		0		0			330	270	340	500	230	A C T C G A C T T T T T T T C A G C T G T G A C C A T T C C C G G G A G	38%			27%				
EPB41L3-NM_001281533.2eU361-NP_001268462.1aa89Q*	4	7%	2%	8%	2%	0		0			320	290	550	260	570	T A T C A G C A A T T A G A A G A C G A T A A A C T T T C T C A G A A A	8%	10%	6%	5%				
EPS8-NM_004447.6eU3448-3'UTR+730	4	4%	1%	4%	1%	0		0			620	60	680	380	230	A A A G C A C A A A T G C G A A A G A G G T C A T G T C T T C T C A A A	3%	5%	4%	3%				
EPS8-NM_004447.6eU3450-3'UTR+732	4	7%	3%	8%	3%	0		3	39%	3%	320	200	620	60	680	A G C A C A A A T G C G A A A G A G G T C A T G T C T T C T C A A A A T	5%	11%	7%	5%		38%	37%	42%
ERBIN-NM_001006600.3eU695-NP_001006600.1aa136Q*	2	3%	1%	3%	1%	0		0			280	420	610	130	360	C C T A T T T C C A A G C T C C C T G A T G G A T T T T C T C A G C T G		4%	3%					
ERCC3-NM_000122.2eU335-NP_000113.1aa91PL	2	2%	0%	2%	0%	0		0			290	210	660	530	70	A T G G C C A T A T C T T C T T G G A A G C C T T C T C T C C A G T T T		2%	2%					
ERMAP-NM_001017922.2eU2020-3'UTR+339	2	19%	7%	24%		0		0			350	210	790	260	360	A C T C C A G G G G T T A T T T A G A A G A C A C T T T C T C T G C C T		24%		14%				
ESF1-NM_001276380.2eU2167-NP_001263309.1aa695PL	4	16%	3%	17%	2%	0		1	2%		460	220	670	440	120	C G G T A A A A T C T G C A A A A G A T G G C A C A T C T C C A G A A G	17%	19%	15%	13%		2%		
EXO5-NM_001346946.2eU1568-3'UTR+17	4	30%	9%	34%	4%	0		1	8%		240	400	760	120	360	A A A A A G C C A A A T G A A T A G A A G G T A T G C T T T C A A G A A	38%	29%	34%	17%		8%		
EZH2-NM_001203247.2eU821-NP_001190176.1aa229SL	4	30%	10%	35%	3%	0		3	3%	1%	280	190	800	320	230	C T T C T G A T A A A A T T T T T G A A G C C A T T T C C T C A A T G T	38%	32%	34%	15%		4%	2%	4%
EZH2-NM_004456.5eU821-NP_004447.2aa229SL	4	88%	9%	91%	9%	0		1	50%		280	190	800	320	230	C T T C T G A T A A A A T T T T T G A A G C C A T T T C C T C A A T G T	100%	81%	90%	80%				50%
EZR-NM_001111077.2eU327-NP_001104547.1aa68Q*	4	1%	1%	2%	0%	0		3	31%	3%	210	240	770	280	310	T G G C T G A A G C T G G A T A A G A A G G T G T C T G C C C A G G A G	1%	2%	1%	1%		29%	34%	29%
FAF2-NM_014613.3eU1247-NP_055428.1aa408PP	4	5%	3%	6%	2%	0		0			250	240	570	640	390	A G A A A A G T T T C A G A T T G A A G C C A A T T T T C C C A G G C G	8%	4%	7%	2%				
FAF2-NM_014613.3eU2540-3'UTR+1179	4	22%	7%	26%	3%	0		0			420	300	770	310	40	G C C T G T G T A A T C T A T T G G A A G G C A C A T T T T C A T T T C	28%	26%	23%	12%				
FAM111B-NM_001142703.2eU1577-NP_001136175.1aa488TI	3	2%	0%	2%	0%	0		0			380	110	600	520	270	T A A T T A G C A A A T G T G C G A A G G T A A C C T T C A C T T A T A	2%	1%	2%					
FAM118B-NM_001330446.1eU255-NP_001317375.1aa24PP	3	8%	3%	8%	3%	0		0			340	350	500	430	330	T T T T G G A T T C T T C A A C G A T G G C G A A C C T C C C A C C A A	11%	6%	6%					
FAM217B-NM_001190827.2eU203-5'UTR-163	4	31%	9%	35%	9%	0		1	19%		260	150	790	400	220	A A T A T A A G A G G A A T A A A G A A G T C A C C T C C C C A G C T G	43%	26%	35%	22%		19%		
FAM72B-NM_001100910.2eU1037-NP_001094380.1aa62TT	2	5%	2%	5%	2%	0		0			270	180	650	460	270	A G T G G A C T T C A C T G G A A G A T G C T A T T T C A C C A A A A T		6%	4%					
FAM91A1-NM_001317917.2eU2730-NP_001304846.2aa578SL	3	6%	1%	6%	1%	0		3	65%	4%	320	160	760	180	290	A A A A C T T A A T A T T T A A A G A T G G T G T C T T A T C A G A A T	5%	6%	8%			64%	61%	68%
FAM98B-NM_173611.4eU583-NP_775882.2aa183SL	2	2%	0%	2%	0%	0		0			260	350	670	310	520	A A G T G G A A T C A A A G G T G A A A G A T A T T C T C T C A A A G G		2%	2%					
FANCA-NM_000135.4eU474-NP_000126.2aa148LL	3	15%	1%	15%	1%	0		3	40%	7%	250	220	740	320	390	A C T G T G G A G C A G A G A A A G A A G C T G T C T T C C C T G T T A	16%	13%	15%			39%	34%	47%
FASTKD2-NM_001136193.2eU1118-NP_001129665.1aa309TI	4	8%	5%	9%	4%	0		3	74%	1%	250	150	790	140	520	A G C A A G T T T G G A A A A T A G A A G A T G T C T T C A C A T T A C	14%	9%	5%	3%		73%	74%	75%
FASTK-NM_006712.5eU626-NP_006703.1aa195PS	2	2%	0%	2%	0%	0		0			260	250	700	400	100	A G G C T C C G C C T C C C T C C G A A G C C A C C T C C C C C T T T G		2%	2%					
FAT1-NM_005245.4eU9222-NP_005236.2aa3004SL	4	13%	4%	15%	2%	0		0			270	160	850	190	180	T T A C T A T C A C G G C A A C T G A T G G C A C C T T C T C A T C A A	15%	17%	13%	7%				
FDFT1-NM_001287742.2eU1818-NP_001274671.1aa372PP	4	8%	4%	9%	3%	0		1	3%		210	400	620	240	460	C A T C T C C A C C A T C C G G A C G C A G A A T C T T C C C A A C T G	12%	11%	6%	4%		3%		
FDFT1-NM_001287742.2eU2033-3'UTR+77	2	2%	0%	2%	0%	0		3	77%	1%	310	70	650	220	310	T T T T T T C T T T A A G G A T G G A T G T T G T G T T C T C T T T A T		3%	2%			75%	76%	78%
FDX2-NM_001031734.4eU559-NP_001026904.2aa183PP	2	3%	1%	3%	1%	0		0			260	160	560	560	330	C A G G A A C T T C T A C G T G G A T G G C A T G T C C C C A A G C C	3%		2%					
FGD1-NM_004463.3eU3472-NP_004454.2aa890FF	3	9%	3%	10%	1%	0		0			350	280	810	250	190	A G G G G A G C G G C C T G A C A G A A G G C A T G T C T T C A A G A T		10%	11%	6%				
FMR1-NM_001185075.2eU1916-3'UTR+41	4	4%	1%	5%	1%	0		0			360	330	660	280	170	C T A A A C T G C A T A A T T C T G A A G T A T A T T T C C T A T A C	4%	5%	5%	2%				
FN1-NM_001306129.2eU191-5'UTR-77	2	2%	0%	2%	0%	0		0			330	570	740	350	250	A C C C C C G G G A G G C A T T A G A A G G G A T T T T T C C C G C A G	2%	2%						
FNBP1L-NM_001024948.3eU1760-NP_001020119.1aa532PP	3	23%	16%	23%	16%	0		1	7%		390	180	600	620	320	G A G A C A G A A C G G T G A A G A A G G C T A C G T T C C C A C G T C	41%	19%	9%				7%	
FNBP1L-NM_001164473.3eU1934-NP_001157945.1aa590PP	4	13%	7%	15%	7%	0		0			390	180	600	620	320	G A G A C A G A A C G G T G A A G A A G G C T A C G T T C C C A C G T C	11%	24%	12%	7%				
FNDC3B-NM_001135095.2eU4233-3'UTR+496	2	4%	2%	4%	2%	0		0			400	150	770	140	520	A G C A A C A G C A A C A T A C A G A A G A T A T G T T C A C T C A G A		3%	5%					
FOCAD-NM_001375567.1eU3130-NP_001362496.1aa1021LL	2	7%	3%	7%	3%	0		0			450	240	840	230	260	T A G C C A T T A C C A A C C C A G A G G G C A A C T T C T C T C C T G	9%	5%						
FRA10AC1-NM_001347712.2eU623-NP_001334641.1aa68LL	4	14%	4%	14%	4%	0		2	15%	8%	220	240	800	140	390	G G A A G A A G C A A G A A A T A G A A G G T T T C A T C T C A T A G C	19%	13%	11%	13%		9%		20%
FRA10AC1-NM_145246.5eU422-NP_660289.2aa68LL	2	19%	3%	19%	3%	0		0			220	240	800	140	390	G G A A G A A G C A A G A A A T A G A A G G T T T C A T C T C A T A G C		17%	21%					
FUCA2-NM_032020.5eU1123-NP_114409.2aa346SF	4	4%	2%	5%	1%	0		0			320	220	700	170	120	A T A T T G G G C C C A C A C T A G A T G G C A C C A T T T C T G T A G	7%	4%	6%	1%				
FURIN-NM_001289823.2eU3488-3'UTR+878	2	3%	0%	3%	0%	0		0			600	130	710	270	290	G A T C T C A G G G G C T G T T T G A G G A T A T A T T T T C A C T T T		2%	3%					
FXR1-NM_001013438.3eU1017-NP_001013456.1aa334PL	4	3%	1%	4%	1%	0		0			180	290	680	260	250	A T A A A T T A C C C A G A G A A G A C G G T A T G G T T C C A T T T G	3%	5%	4%	2%				
FXYD5-NM_001164605.2eU1066-3'UTR+231	3	6%	3%	6%	3%	0		0			320	80	680	210	230	C C T T G G G G G C T G T C C C T C A A G T T A T C T C C T C T G C T A	4%	6%	9%					
GBP1-NM_002053.3eU656-NP_002044.2aa196LL	4	3%	2%	3%	1%	0		0			450	170	520	400	330	C C T G G A C T T G G A A G C A G A T G G A C A A C C C C T C A C A C C	4%	4%	2%	1%				
GCLM-NM_001308253.2eU1480-3'UTR+454	4	17%	12%	19%	13%	0		0			320	340	700	390	390	A A A C T G A A A T A C T G G A A G A A G A T A T T T T T C C T G T C A	27%	4%	27%	9%				
GDPD5-NM_001351167.2eU2959-NP_001338096.1aa360LL	2	6%	0%	6%	0%	0		0			330	300	440	370	420	C T G G A T C A T G C C C C C G G A C G A G T A C T G T C T C A T G T G	7%	6%						
GLRB-NM_000824.5eU959-NP_000815.1aa261TI	2	28%	20%	28%	20%	0		0			270	250	820	220	260	G C T A C T A C A C A T G C G T G G A A G T C A T C T T C A C C C T G A		42%	14%					
GLUD1-NM_001318900.1eU744-NP_001305829.1aa212PL	4	2%	0%	2%	0%	0		0			160	270	690	250	370	G G A G T A T A T G G A A T C C A G A T G G T A T T G A C C C A A A G G	2%	2%	2%	2%				
GM2A-NM_000405.5eU469-NP_000396.2aa146SL	4	20%	10%	24%	7%	0		0			370	170	830	310	220	G C C A C T G T C C C T T C A A A G A A G G A A C C T A C T C A C T G C	16%	29%	26%	7%				
GNB1-NM_002074.5eU455-NP_002065.1aa31SF	2	2%	0%	2%	0%	0		0			400	180	640	320	350	C C A G G A A A G C A T G T G C A G A T G C A A C T C T C T C T C A G A		2%	2%					
GOSR2-NM_001321134.2eU2066-3'UTR+1487	4	16%	8%	19%	5%	0		0			270	210	680	330	310	A T T A A C T G A G T T T C C T G G A T G C T A A T T T C A C A C T T T	22%	23%	13%	6%				
GPCPD1-NM_019593.5eU206-NP_062539.1aa5Q*	4	23%	13%	26%	15%	1	12%	0			310	110	730	390	260	T A A A A T A A A T C C A T C A G A A T G A C A C C T T C T C A G G T T	15%	43%	20%	15%	12%			
GRB2-NM_002086.5eU474-NP_002077.1aa49PP	4	8%	4%	9%	3%	0		3	4%	1%	330	230	440	640	320	A G A G C T T A A T G G A A A A G A C G G C T T C A T T C C C A A G A A	12%	6%	9%	3%		2%	4%	5%
GRWD1-NM_031485.4eU649-NP_113673.3aa209SF	2	2%	0%	2%	0%	0		0			310	210	650	390	370	A T G A G C A G G C C C A A A T G A A G C C C A T C T T C T C C T T C G		2%	1%					
GSDME-NM_001127453.2eU1986-3'UTR+407	4	14%	5%	16%	4%	0		0			270	380	790	290	280	C A T T G T G A A T G C T G T G G G A A G G C A A T T T C T C T G C A C	12%	21%	14%	8%				
GSPT1-NM_001130006.2eU5329-3'UTR+3182	3	8%	0%	8%	0%	0		0			130	210	760	40	280	G A T G T A G T T C A A C A G T T G C C G G C A T C C T C T C A G C T G		8%	8%	7%				
GSR-NM_000637.5eU2265-3'UTR+685	3	10%	1%	10%	1%	0		0			500	290	670	320	60	T A A T T T T C A C A G A A G T T G A T G G C A A T T C T T C A C A T G	11%	9%	9%					
GSTK1-NM_001143679.2eU948-3'UTR+26	3	2%	0%	2%	0%	0		0			400	220	630	490	0	T T T A A G A T T G C C C G G A G G A A G C A A A C T C T T C G T A T A	2%	2%	2%					
GTF2F1-NM_002096.3eU1163-NP_002087.2aa332PS	3	3%	0%	3%	0%	0		0			150	390	710	270	250	G A G G A G G A G G A G G A G A A G A A G G C A C C C A C C C C G C A G	3%	3%	3%					
GTF3C4-NM_012204.4eU4155-3'UTR+1551	3	16%	1%	16%	1%	0		0			110	510	690	220	210	T A C T T A G A T G A T A C C A T G A C G G C A A C C T C C C C A C C C	17%	15%	15%					
GTF3C4-NM_012204.4eU4156-3'UTR+1552	4	12%	4%	13%	3%	0		0			400	110	510	690	220	A C T T A G A T G A T A C C A T G A C G G C A A C C T C C C C A C C C C	12%	16%	11%	7%				
H2AX-NM_002105.3eU313-NP_002096.1aa81PL	4	3%	1%	4%	0%	0		0			250	370	460	470	370	C C C G C G A C A A C A A G A A G A C G C G A A T C A T C C C C C G C C	4%	3%	4%	2%				
H2BC12-NM_080593.2eU586-3'UTR+145	3	4%	1%	4%	1%	0		0			480	590	440	400	230	A G A A T G C A A C C A T A C C T A G A T G G A C T T T C C A C A A G	4%	2%	5%					
HBEGF-NM_001945.3eU1342-3'UTR+440	4	14%	1%	15%	1%	0		0			230	130	840	180	330	A A A G C A A C A A A G A T T G A G A A G C C A T G T A C T C A A G T A	14%	15%	15%	13%				
HCG18-NR_024052.2eU2996-ncr	3	70%	21%	70%	21%	0		0			340	350	750	350	170	A C T G T A T A C T A A C A T C T G A T G G C A T T T T T C C A C T G T	83%	80%	46%					
HDAC2-NM_001527.4eU838-NP_001518.3aa200HY	4	10%	3%	11%	1%	0		1	1%		230	290	690	260	210	A C A A C A G A T C G T G T A A T G A C G G T A T C A T T C C A T A A A	11%	10%	12%	6%			1%	
HDAC3-NM_001355039.2eU636-NP_001341968.1aa193HY	4	2%	1%	3%	1%	0		3	33%	1%	370	340	610	260	240	C T C A C T G A C C G G G T C A T G A C G G T G T C C T T C C A C A A A	4%	3%	2%	2%		32%	33%	33%
HEATR6-NM_022070.5eU1158-NP_071353.4aa384SL	4	6%	3%	7%	3%	0		3	40%	9%	320	90	770	330	290	G T G G A G C T G C A G A A A A A G A T G G A G T C T C C T C A T C C T	7%	4%	10%	4%		34%	37%	50%
HERC5-NM_016323.4eU682-NP_057407.2aa177PP	2	3%	1%	3%	1%	0		0			360	450	710	470	200	T G G G C A G C T T G G A G T T G G A A G G A A A T T T C C C T C A A C	2%		3%					
HIBADH-NM_152740.4eU901-NP_689953.1aa269SL	2	3%	0%	3%	0%	0		3	36%	3%	260	200	600	300	340	C T G T A C C T G G A G T G A T G G A T G G C G T T C C C T C G G C T A		3%	2%			39%	34%	34%

HIF1A-NM_001243084.2eU2903-3'UTR+211	3	2%	1%	2%	1%	3	11%	2%	0			310	130	690	260	550	TAATGCTGGATCACAGACAGCTCATTTTCTCAGTTT	3%	1%	1%		12%	11%	9%			
HIF1A-NM_001243084.2eU718-NP_001230013.1aa193LL	2	2%	1%	2%	1%	0			1	2%		280	210	740	310	450	AGAACAAAACACACAGCGAAGCTTTTTTCTCAGAAT	3%	2%							2%	
HIF1AN-NM_017902.3eU2893-3'UTR+1827	3	17%	8%	22%	0%	2	12%	1%	0			270	210	770	190	400	CAGTGTGGATGTGAAGATATGGTACCTTCTCAAGTG	22%	22%		8%	12%	11%				
HIP1R-NM_003959.3eU3279-NP_003950.1aa1062PL	4	35%	11%	40%	4%	0			3	17%	5%	170	160	810	360	210	ACCAGCTTGACAAAAAGGATGGCATCTACCCAGCTC	43%	43%	35%	20%				14%	14%	23%
HLTF-NM_001318934.2eU3067-NP_001305863.1aa957TI	3	12%	3%	12%	3%	0			0			160	200	800	120	400	GACTTGGTCAGAAGCAAGAAGTTATCATCACAAAAT	9%	16%	10%							
HLTF-NM_001318934.2eU331-NP_001305863.1aa45PL	4	13%	6%	16%	2%	0			0			280	180	630	330	280	CACGTTTTGAATTCCAAGATGTTATCCCTCCAGATG	18%	16%	14%	5%						
HMGB1-NM_001313892.2eU1143-3'UTR+314	2	1%	0%	1%	0%	0			3	40%	2%	380	230	470	540	330	ATTAGTTATATATGGGGATGGTAGTTTTTTCATCTT		1%	1%					41%	40%	38%
HMGCS1-NM_001098272.3eU500-NP_001091742.1aa125TI	3	2%	1%	3%	1%	0			0			110	220	780	160	340	CTGGGAATACAGATATAGAAGGAATCGACACAACTA	3%	3%		1%						
HNRNPA0-NM_006805.4eU310-NP_006796.1aa5Q*	2	1%	0%	1%	0%	0			3	21%	2%	350	240	720	220	300	AAGGCCCGGGCCTTGGAGATGGAGAATTCTCAGTTG	1%	2%						19%	23%	21%
HNRNPC-NM_001077442.2eU294-NP_001070910.1aa38SL	4	4%	2%	5%	1%	0			1	1%		430	180	710	470	150	TCAAGAAATCTGATGTGGAGGCAATCTTTTCGAAGT	6%	6%	4%	2%				1%		
HNRNPC-NM_001077443.2eU294-NP_001070911.1aa38SL	4	4%	1%	4%	0%	0			0			430	180	710	470	150	TCAAGAAATCTGATGTGGAGGCAATCTTTTCGAAGT	4%	5%	5%	3%						
HNRNPC-NM_004500.4eU320-NP_004491.2aa38SL	4	5%	2%	6%	1%	0			0			430	180	710	470	150	TCAAGAAATCTGATGTGGAGGCAATCTTTTCGAAGT	7%	6%	5%	2%						
HNRNPC-XM_006720125.3eU690-5'UTR-212	4	31%	5%	33%	3%	0			2	5%	1%	260	290	770	310	350	TTAACACTTATGATCGTGATGGCATTTTCCCTGAAA	30%	34%	36%	24%				6%	4%	
HNRNPK-NM_001318186.2eU202-NP_001305115.1aa11PL	4	22%	9%	26%	3%	1	1%		3	2%	1%	340	270	760	370	300	TGGAAACTGAACAGCCAGAAGAAACCTTCCCTAACA	30%	25%	25%	10%		1%		2%	3%	2%
HNRNPK-NM_002140.5eU252-NP_002131.2aa11PL	4	23%	9%	27%	3%	1	1%		3	2%	0%	340	270	760	370	300	TGGAAACTGAACAGCCAGAAGAAACCTTCCCTAACA	30%	27%	25%	10%		1%		1%	2%	2%
HNRNPK-XM_005251960.2eU237-XP_005252017.1aa11PL	4	23%	8%	27%	3%	0			0			340	270	760	370	300	TGGAAACTGAACAGCCAGAAGAAACCTTCCCTAACA	27%	23%	29%	11%						
HNRNPK-XM_005251963.4eU267-XP_005252020.1aa11PL	2	21%	7%	21%	7%	0			0			340	270	760	370	300	TGGAAACTGAACAGCCAGAAGAAACCTTCCCTAACA		17%	26%							
HNRNPLL-NM_001142650.2eU1458-NP_001136122.1aa466TI	4	26%	9%	31%	2%	0			1	2%		390	240	810	190	390	TTCCATTGTGTGTCACAGAAGAGACCTTCACAAAGT	33%	31%	29%	12%						2%
HOMER1-NM_001277077.1eU2452-3'UTR+715	2	20%	4%	20%	4%	0			0			360	310	740	150	570	ATTCCATGTATTTATGAGAAGATATTGTCTCCCATT		17%	23%							
HOXB2-NM_002145.4eU1085-NP_002136.1aa296SL	3	3%	0%	3%	0%	0			3	41%	7%	310	150	820	140	370	AGCCCGGGCCATTGCCAGAAGACGTCTTCTCGGGGC	3%	3%	3%					34%	40%	48%
HP1BP3-NM_001372052.1eU682-NP_001358981.1aa157PS	4	3%	1%	4%	1%	0			1	4%		170	380	740	300	250	GCCCAGAAACAAACACCGATGGCTTCTTCCCCACGT	5%	4%	3%	2%						4%
HPF1-NM_017867.3eU169-NP_060337.2aa34SS	2	3%	1%	2%		0			0			460	210	440	560	190	GAAAAGTAAATTCTGTGAAGCTGATGTCTCCAGTGA			2%	3%						
HRH1-NM_000861.3eU1023-NP_000852.1aa278Q*	2	7%	5%	7%	5%	0			0			200	270	740	230	140	GGTGGTGGATCTGTCTTGAAGTCACCATCCCAAACC		10%	3%							
HSBP1-NM_001537.4eU515-3'UTR+185	4	6%	3%	7%	1%	0			0			170	160	790	180	400	CCCCCCAGTTTCTTGAACATGGTATCTTCACATCTT	8%	7%	6%	2%						
HSDL1-NM_001146051.2eU2775-3'UTR+1781	3	7%	3%	7%	3%	0			0			370	150	720	260	140	CACACCTTGTCAGTGATGATGTAAACTTCTCTCCTT	11%	5%	7%							
HSP90AA1-NM_001017963.3eU3402-3'UTR+492	4	7%	2%	8%	1%	1	0%		2	0%	0%	570	460	570	310	160	AACTGAAGCAACTTGATGGAAGGATCTCTCCACAGG	8%	8%	7%	5%	0%			0%	0%	
HSP90AA1-XM_011536718.2eU3399-3'UTR+492	2	11%	7%	11%	7%	0			0			570	460	570	310	160	AACTGAAGCAACTTGATGGAAGGATCTCTCCACAGG		16%	6%							
HSPA13-NM_006948.5eU3529-3'UTR+2086	3	10%	4%	10%	4%	0			0			350	280	730	370	110	TACACCTTTAATAAAATGAAGGAATGTCTTCAATCA	10%	15%	6%							
HSPA1A-NM_005345.6eU1500-NP_005336.3aa429TI	4	3%	1%	3%	0%	0			0			340	300	490	350	460	CCATCCCCACCAAGCAGACGCAGATCTTCACCACCT	3%	3%	3%	2%						
HTT-NM_001388492.1eU9111-NP_001375421.1aa2989PL	2	4%	2%	4%	2%	0			1	6%		310	140	790	300	370	TCCTGCCCCAGTTTCTAGACGACTTCTTCCCACCCC		3%	6%							6%
HVCN1-NM_001040107.2eU1015-3'UTR+38	2	20%	2%	19%		0			0			250	210	770	240	400	CCAGCTCCCCTCAAAAAGAAGACACTGTCTCATGGG		19%		21%						
HYPK-NM_016400.4eU403-3'UTR+13	2	5%	2%	5%	2%	0			3	64%	4%	400	310	430	430	380	ATTGCCCTAACCAACTGATGCGTGCTTTCTCAAATA	4%		6%					68%	63%	61%
IARS2-NM_018060.4eU1353-NP_060530.3aa423TI	3	4%	1%	4%	1%	0			3	43%	7%	260	220	740	330	400	ATTGTCTAGTGGACGAAGATGGAGTTTTCACAGATG	3%	5%	4%					38%	50%	40%
ICAM1-NM_000201.3eU1353-NP_000192.2aa438PL	3	11%	5%	13%	3%	0			1	8%		310	290	760	480	240	AGCTCAAGTGTCTAAAGGATGGCACTTTCCCACTGC		11%	15%	6%						8%
IFI16-NM_001206567.2eU707-NP_001193496.1aa144LL	3	16%	4%	16%	4%	0			0			320	300	810	220	370	ATGTCAGGTAACTCCCAGAAGAAATGTTCTCCAAAA	13%	20%	14%							
IFI16-NM_001364867.2eU1199-NP_001351796.1aa200LL	4	12%	4%	14%	1%	0			0			320	300	810	220	370	ATGTCAGGTAACTCCCAGAAGAAATGTTCTCCAAAA	14%	13%	13%	6%						
IFT52-NM_001303458.3eU1181-NP_001290387.1aa361SF	2	2%	0%	2%	0%	0			0			310	170	710	270	300	TGGAGCTATTTGATTTAGATGAAACGTTCTCCTCTG		1%	2%							
IKBIP-NM_201612.4eU964-NP_963906.1aa293RC	3	6%	1%	6%	1%	0			0			170	410	730	430	440	AAGGTGTATAATCTAAAGAAGGACTTTTCCCGTTTA	5%	7%	5%							
IL13RA1-NM_001560.3eU2783-3'UTR+1466	3	8%	5%	8%	5%	0			0			180	240	740	100	570	TTTCTAAACAGAGATGAGGTGGTATCTTCACGTAGA	5%	14%	5%							
IL6ST-NM_001190981.2eU2166-NP_001177910.1aa628TI	4	12%	3%	13%	3%	0			0			260	300	750	210	330	AAGATCAAATGTATTCAGATGGCAATTTCACTGATG	12%	17%	12%	9%						
ILK-NM_001014794.3eU112-NP_001014794.1aa6TI	4	19%	8%	23%	3%	0			2	3%	0%	400	130	730	290	440	GCCGGGACGCTGCTATGGACGACATTTTCACTCAGT	21%	27%	22%	7%				3%	2%	
ILK-NM_001014795.3eU515-NP_001014795.1aa6TI	4	21%	7%	24%	5%	0			0			400	130	730	290	440	GCCGGGACGCTGCTATGGACGACATTTTCACTCAGT	24%	30%	19%	13%						
ILK-NM_001278441.2eU158-NP_001265370.1aa6TI	4	25%	10%	29%	7%	0			2	5%	0%	400	130	730	290	440	GCCGGGACGCTGCTATGGACGACATTTTCACTCAGT	37%	28%	23%	13%				5%		5%
ILK-XM_005252904.5eU184-XP_005252961.1aa6TI	4	21%	7%	23%	7%	0			0			400	130	730	290	440	GCCGGGACGCTGCTATGGACGACATTTTCACTCAGT	30%	24%	16%	15%						
IPO7-NM_006391.3eU1179-NP_006382.1aa356PL	4	27%	10%	31%	2%	0			1	3%		240	260	710	390	310	TACAAGGCATTATCCAAGATGTTATTTTTCCATTGA	33%	30%	31%	12%						3%
IRAK1-NM_001025242.2eU2185-3'UTR+46	2	2%	1%	2%	1%	0			0			310	190	550	470	300	AGATCCCCCAAATCCGGAAGTCAAAGTTCTCATGGT	1%	2%								
IRAK2-NM_001570.4eU546-NP_001561.3aa170PP	4	19%	9%	22%	9%	0			1	6%		450	190	750	400	260	TGCCCCTCATTCCTTGAGAAGCGACCTCCCCACTTC	30%	13%	23%	10%				6%		
ITFG1-NM_001305002.2eU658-NP_001291931.1aa132SF	4	12%	6%	14%	5%	0			0			270	240	760	300	140	AAATATGGAAAAATTTGGATGGAAACTTCTCTGTCA	20%	10%	12%	6%						
ITFG1-NM_030790.5eU213-NP_110417.2aa65FF	4	4%	2%	4%	2%	0			0			400	390	480	250	360	CCTCAACTCCGACAAGCAGACGGATCTCTTCGTGCT	4%	6%	3%	2%						
ITGB1BP1-NM_001319070.2eU167-5'UTR-26	3	4%	2%	4%	2%	0			0			440	200	820	230	330	GATGGGCCCGCGGCTGCGAGGGGATCTTCTCTGGAT	4%	2%	6%							
ITGB1BP1-NM_004763.5eU167-5'UTR-26	3	4%	1%	4%	1%	0			0			440	200	820	230	330	GATGGGCCCGCGGCTGCGAGGGGATCTTCTCTGGAT	5%	3%	4%							
JMJD1C-NM_001282948.2eU4504-NP_001269877.1aa1237SF	3	6%	3%	6%	3%	0			0			320	140	750	360	310	CCAGCTGGGGTGGTTCAGAAGTAATTTCCTCTTTAT	4%	10%	5%							
JMJD1C-NM_001282948.2eU7324-NP_001269877.1aa2177SL	3	5%	1%	5%	1%	1	2%		0			220	360	750	230	540	TTGGCATAGCAAAAGGAAATGGCATTCTCTCAAAAG	4%	6%	5%		2%					
JPT2-NM_144570.3eU144-NP_653171.1aa37SF	2	2%	0%	2%	0%	0			0			330	400	550	390	270	TTTTTGGAAGTCCAGAAGAAGCTACTCCTTCCAGCA		2%	2%							
KAT7-NM_001199155.2eU3107-3'UTR+1207	3	5%	2%	5%	2%	0			0			440	260	730	360	330	GTGTTGTTCAAGTGAAGGAAGAGATGTTCCCTCTAA	8%	5%	3%							
KATNA1-NM_007044.4eU614-NP_008975.1aa119Q*	2	7%	1%	7%	1%	0			0			270	110	620	390	450	TCACCAGGACCTAGAAAACGCCAATCTTCTCAGTAC	8%	7%								
KCTD10-NM_001317395.2eU2274-3'UTR+1291	4	4%	2%	5%	2%	0			3	24%	4%	320	360	650	60	420	GGCGCAGCGCCTATGTAGGACGGGTGTTCTCAGTGA	3%	4%	7%	3%				28%	24%	20%
KDM1B-NM_001364614.2eU462-NP_001351543.1aa85LL	3	11%	1%	11%	1%	0			0			310	160	750	80	270	AAATGGCTACACCTCCCGATGGTATCATCTCTCCTG	10%	11%	12%							
KIDINS220-NM_001348729.2eU3980-NP_001335658.1aa1270FF	2	2%	0%	2%	0%	0			0			350	260	620	390	80	GAATATGAATTTTGGAGACTGGCACCTTTTCAGAAG		3%	2%							
KIF1B-NM_001365953.1eU3475-NP_001352882.1aa1054LL	2	2%	0%	2%	0%	0			0			170	310	790	270	500	ACCTCAACTACGTTGGAGAAGCAATTCTCTCAATAA		2%	2%							
KIF20B-NM_001284259.2eU480-NP_001271188.1aa130Q*	2	12%	8%	12%	8%	0			0			240	410	760	270	360	GGCCCAGCAACTACACAGAAGGAATTCTTTCAGGGT		7%	18%							
KIF2A-NM_001098511.3eU449-NP_001091981.1aa61SL	2	7%	3%	7%	3%	3	54%	6%	0			560	260	750	310	150	GCAAAGAGATTGACCTGGAGAGCATCTTTTCACTTA		4%	9%		50%	61%	51%			
KIF5B-NM_004521.3eU2127-NP_004512.1aa559LL	3	5%	2%	5%	2%	0			0			330	270	750	220	160	AAACGAGCAGCTGAGATGATGGCATCTTTACTAAAA	7%	4%	5%							
KIF5B-NM_004521.3eU5771-3'UTR+2427	3	8%	2%	8%	2%	0			0			380	200	720	190	330	GTTTATTTTCAGTGCTTGAAGATACATTCACAAATA	10%	7%	8%							
KIFAP3-NM_001204514.2eU2537-NP_001191443.1aa637LL	2	15%	8%	15%	8%	0			0			570	200	650	390	410	TGAGCCATACATTCATGAAGGAGATATTCTCGAAAG	21%	9%								
KIZ-NM_001163022.3eU562-NP_001156494.1aa128Q*	3	15%	3%	13%	2%	0			0			400	270	740	410	190	TCTGACAACATAGATGGAAAGGCATCTCTTCAGATT		15%	12%	17%						
KLC1-NM_001130107.2eU773-NP_001123579.1aa163PL	3	5%	1%	5%	1%	0			0			260	130	720	420	310	AGCTAAAAAAATATGATGACGACATTTCCCCATCCG	6%	5%	4%							
KLHDC3-NM_057161.4eU306-NP_476502.1aa49FF	4	5%	2%	6%	1%	0			0			460	310	480	440	120	GACACTGCGTCAGATAGATGTGCACATTTTCAATGC	7%	5%	7%	2%						
KLHL9-NM_018847.4eU736-NP_061335.1aa64PL	2	9%	4%	12%		0			0			250	140	780	320	450	TGGTACCAGGTGATGGAGATGAAATCTTCCCTGTTC			12%	6%						
KMT2B-NM_014727.3eU5696-NP_055542.1aa1894LL	3	2%	0%	2%	0%	0			0			340	110	610	390	420	CCCCTGCCCTCCCCTGGAAGTCCATCTTCACTGACC	2%	2%	2%							
KNSTRN-NM_001142761.1eU484-NP_001136233.1aa123SS	2	2%	0%	2%	0%	0			0			460	330	500	570	230	ACCTGTTAGGTCCAAAGAAGTCGATGTTTCCAAACA		2%	2%							
KPNA2-NM_001320611.2eU1143-NP_001307540.1aa175HY	4	2%	1%	3%	1%	0			0			180	160	730	270	170	CCAGCATTCATTTCTCTGTTGGCATCTCCCCATGCT	3%	1%	4%	1%						
KPNB1-NM_001276453.2eU1307-NP_001263382.1aa220PL	4	4%	2%	4%	1%	0			0			300	200	750	290	430	TGGCCACCTGCTGTGAAGATGACATTGTCCCACATG	5%	5%	3%	2%						
KRT10-AS1-NR_160886.1eU332-ncr	4	12%	5%	14%	3%	0			0			170	280	720	220	320	GGGAGAGAGTTGAAATGGTTGGCATCCTCCCACTCT	11%	18%	14%	5%						
KRT10-AS1-NR_160888.1eU301-ncr	3	12%	4%	12%	4%	0			0			170	280	720	220	320	GGGAGAGAGTTGAAATGGTTGGCATCCTCCCACTCT	15%	14%	8%							
KYAT3-NM_001008661.3eU1115-NP_001008661.1aa357LF	3	6%	2%	6%	2%	0			0			320	220	600	310	150	GAAGTAAAAAGAGATCGGATGGTACGTTTACTTGAA	4%	6%	8%							
LEO1-NM_001286430.2eU1001-NP_001273359.1aa323SL	4	11%	6%	12%	6%	0			0			390	60	740	250	290	ATTTATTTGGAGGTGCAGATGATATCTCTTCAGGGA	16%	5%	15%	5%						
LIMA1-NM_001113546.2eU137-NP_001107018.1aa5PS	3	21%	4%	21%	4%	0			1	6%		170	310	790	200	290	TCAGTGTCTGTAGACAAGATGGAATCATCTCCATTT	23%	17%	24%							6%
LIN54-NM_001115007.3eU1512-NP_001108479.1aa444Q*	4	33%	15%	39%	11%	0			2	8%	5%	220	250	800	120	340	CAAACAGCAGCCAAGACGAAGTTATCCTCTCAAATT	52%	35%	31%	14%				4%		12%
LINC00641-NR_038970.1eU4413-ncr	2	7%	5%	7%	5%	0			0			210	230	720	260	290	TGAGGGATGCCCCCAAGGAAGTTAACTTCACTGCAA	3%	10%								
LINC02987-NR_110687.1eU1517-ncr	4	51%	17%	59%	12%	0			3	89%	10%	260	220	680	380	490	TTTGTCTTTTTAGTAGAGACGGTGTTTCCCCATGTT	72%	52%	52%	30%				100%	87%	81%

LINS1-NR_148019.2eU1555-ncr	3	11%	2%	12%	1%	0			0			410	260	530	370	340	CAGTCCTGGTGCAACAGATGGAGATCCTTTCAGGGA		13%	12%	9%						
LPCAT1-NM_024830.5eU1282-NP_079106.3aa389SL	4	19%	12%	22%	11%	0			0			440	210	790	260	190	CCGTTTCTGACTTGCTGGAAGACATGTTTTCACTGT	11%	22%	34%	8%						
LPCAT3-NM_005768.6eU823-NP_005759.4aa257LL	4	24%	11%	30%	2%	2	4%	1%	3	5%	1%	540	80	600	500	310	CAGCCCCCACATCACAGAAGACTATCTCCTCACTGA	27%	31%	31%	8%		5%	4%	4%	5%	6%
LPP-NM_001167671.3eU6485-3'UTR+4333	2	24%	5%	24%	5%	0			0			300	290	820	180	240	AAGAAAAGAAAGAGAGAGAAGTCAACTTTTCATCTT		27%	20%							
LRIF1-NM_001006945.2eU1130-3'UTR+114	2	2%	1%	2%	1%	0			3	39%	13%	420	230	460	460	390	AAGTGTCTTTGAATATGAAGCTTGTTTTTTCATCAG	1%	2%						33%	32%	54%
LRIF1-NM_001006945.2eU895-NP_001006946.1aa194PL	4	46%	16%	54%	5%	3	2%	0%	3	5%	2%	210	280	780	310	450	TCAATAAAAATTATACCGAAGATATTTTCCCAGTGA	59%	50%	51%	23%	2%	2%	3%	5%	3%	7%
LRRC37A2-NM_001006607.2eU4244-NP_001006608.2aa1414SF	2	11%	6%	11%	6%	0			0			300	240	770	210	220	AAAACACTAACATGCCAGAAGGAACCATCTCTGAAA		15%	6%							
LSG1-NM_018385.3eU116-NP_060855.2aa29HH	2	5%	1%	5%	1%	0			0			210	150	800	120	260	TCAGACTCAGCGGAGCCGAAGCCATCGTCACACTGA	6%		4%							
LSM14B-NM_144703.3eU1898-3'UTR+548	3	7%	3%	9%	0%	0			3	51%	6%	300	410	740	390	310	GGTGGGTGGAGGGCACGGAAGGGGTTTTCCCATGGA		9%	9%	4%				57%	46%	48%
LUZP6-NM_001128619.3eU377-5'UTR-2716	4	1%	1%	2%	1%	0			0			510	100	560	630	240	CGGACACTAGAAGGTGGAAGGAAACCTCTTCATTAT	1%	1%	2%	1%						
LY6E-NM_001127213.2eU106-5'UTR-6	4	4%	2%	5%	1%	0			1	2%		210	310	590	330	480	TGTGACCTCCAGGCAGGACGGCCATCCTCTCCAGAA	6%	4%	4%	1%						2%
LY6E-NM_002346.3eU111-5'UTR-6	4	4%	2%	5%	1%	0			2	1%	0%	210	310	590	330	480	TGTGACCTCCAGGCAGGACGGCCATCCTCTCCAGAA	6%	3%	4%	2%				1%		2%
LYRM1-NM_001128301.3eU920-3'UTR+200	3	6%	1%	6%	1%	0			0			150	340	700	330	270	ATCTTTCTTCCTGAGTGGATGGCATTATCCCTAGAG	6%	7%	5%							
M6PR-NM_001207024.2eU881-3'UTR+146	4	3%	1%	3%	1%	3	8%	2%	0			290	200	660	220	250	TTTCCAGTTTGCTTTTGATTTGCATCTTCTCACTAG	4%	4%	3%	1%	7%	10%	7%			
MACROH2A1-NM_001040158.1eU281-NP_001035248.1aa24PL	3	2%	1%	2%	1%	0			0			200	460	710	240	280	CCAGGTCTGCCAAAGCAGGAGTCATCTTTCCCGTGG	2%	1%	3%							
MACROH2A1-NM_001040158.1eU596-NP_001035248.1aa129TI	4	7%	3%	8%	2%	0			0			210	150	800	200	260	GATCCAAAGGAAAGTTGGAAGCCATCATCACACCAC	11%	7%	7%	4%						
MACROH2A1-NM_138609.3eU589-NP_613075.1aa129TI	3	8%	2%	9%	2%	0			0			210	150	800	200	260	GATCCAAAGGAAAGTTGGAAGCCATCATCACACCAC		7%	10%	7%						
MACROH2A1-NM_138610.3eU589-NP_613258.2aa129TI	2	9%	2%	9%	2%	0			0			210	150	800	200	260	GATCCAAAGGAAAGTTGGAAGCCATCATCACACCAC		10%	8%							
MAF1-NM_032272.5eU483-NP_115648.2aa14SL	4	3%	1%	4%	1%	0			0			320	80	780	120	170	TGGAGAACTCGAGCTTTGAAGCCATCAACTCACAGC	5%	3%	4%	1%						
MAML1-NM_014757.5eU1201-NP_055572.1aa304PS	4	50%	13%	57%	4%	0			3	14%	2%	170	420	740	270	420	CAGGACATTAATATTAAGACGGAATTCTCTCCAGCA	60%	54%	55%	31%				14%	12%	17%
MANF-NM_006010.6eU207-NP_006001.5aa52SL	4	29%	8%	33%	3%	0			3	3%	0%	450	70	800	190	340	AGGACCTCAAAGACAGAGATGTCACATTCTCACCAG	37%	31%	32%	17%				2%	3%	3%
MAP1B-NM_001324255.2eU4415-NP_001311184.1aa1401SL	2	14%	5%	14%	5%	0			0			260	300	730	200	260	CTAAGATGTCCATTTCTGAAGGTACTGTCTCAGACA		17%	11%							
MAP3K13-NR_038322.2eU1239-ncr	4	27%	12%	30%	13%	0			3	5%	1%	270	190	730	300	300	GTCCTGTTACACAGTTGGAAGTCATTCCCTCAGATT	43%	30%	18%	16%				5%	5%	6%
MAP3K20-NM_133646.3eU6882-3'UTR+5323	3	18%	1%	17%	0%	0			0			150	220	740	150	440	AGTGTGAGCTTTGAATAGATGGCATTATCACTTTAT	17%	17%		19%						
MAP3K2-NM_001371910.2eU4761-3'UTR+2312	3	16%	3%	16%	3%	0			0			420	270	790	310	190	CTTTATCTTTTATGAATGAGGGCATCTTTTCTACTT	14%	15%	20%							
MAP4K2-NM_001307990.2eU1573-NP_001294919.1aa503TI	3	18%	4%	18%	4%	0			1	13%		270	170	850	260	220	TGGTGGTAGGGGCCGAGGAAGGCATCTACACACTCA	21%	14%	18%							13%
MAP4K5-NM_006575.6eU1451-NP_006566.2aa407PL	2	2%	0%	2%	0%	0			0			330	360	690	280	120	GGATAAGCAGTTACCCTGAAGACAACTTTCCGGATG		2%	2%							
MAP4K5-NM_006575.6eU1835-NP_006566.2aa535TI	4	16%	4%	18%	2%	0			0			270	150	760	250	420	TTATTTTTGGAACTGAAGATGGTATTTACACACTGA	17%	17%	20%	10%						
MAP4K5-XM_011536377.3eU1833-XP_011534679.1aa535TI	4	17%	5%	15%	4%	0			0			270	150	760	250	420	TTATTTTTGGAACTGAAGATGGTATTTACACACTGA	20%	13%	12%	24%						
MAPK7-NM_002749.4eU1553-NP_002740.2aa472SL	2	4%	0%	4%	0%	0			3	22%	6%	300	160	700	170	320	CTGCCCCACCAAAGAAAGATGGTGCCATCTCAGACA	4%		3%					15%	28%	22%
MAPRE2-NM_001143826.3eU2312-3'UTR+1335	3	12%	3%	12%	3%	0			1	4%		290	300	660	190	320	TATTTTTTCCTTTGACAGATGGTATCCCTTCCTGGA	16%	10%	10%							4%
MARF1-NM_001184998.2eU6995-3'UTR+1571	3	10%	1%	10%	1%	0			0			320	280	790	280	230	ACTCTCCGAATCCTCCTGAAGGCATTTCCTCTACAG	9%	11%	11%							
MARK3-NM_001128918.3eU1970-NP_001122390.2aa452SF	2	3%	0%	3%	0%	0			0			380	250	750	400	310	ATAGTGACCTCAAAGAAGATGGAATTTCCTCCCGGA	3%		2%							
MBD4-NM_001276270.2eU1311-NP_001263199.1aa399PL	2	5%	1%	5%	1%	0			0			290	220	700	230	320	CCAGGAAAGACTTCACTGAAGATACCATCCCACGAA	5%	5%								
MBD4-NM_001276271.2eU1329-NP_001263200.1aa405PL	2	8%	3%	8%	3%	0			0			290	220	740	270	360	GTGAGAAAATATTTCAAGAAGATACCATCCCACGAA		6%	10%							
MBIP-NM_001144891.2eU918-NP_001138363.1aa290PL	4	47%	6%	48%	6%	1	9%		2	25%	2%	380	300	730	430	70	ATAAAATCCTTGAATTGGAAGGCATCTCTCCTGAAT	56%	45%	45%	43%			9%		26%	23%
MBIP-NM_016586.3eU918-NP_057670.2aa290PL	4	52%	11%	56%	10%	2	7%	1%	3	21%	5%	380	300	730	430	70	ATAAAATCCTTGAATTGGAAGGCATCTCTCCTGAAT	67%	52%	49%	40%	6%		7%	15%	22%	25%
MBIP-XR_001750370.2eU957-ncr	3	54%	12%	54%	12%	0			0			380	300	730	430	70	ATAAAATCCTTGAATTGGAAGGCATCTCTCCTGAAT	67%	44%	50%							
MCL1-NM_001197320.2eU2469-3'UTR+1795	3	4%	1%	4%	1%	1	2%		0			260	310	640	300	290	AAGTCCCTTTTCCTTGGACTGGTATCTTTTCAACTA	4%	4%	5%				2%			
MCM6-NM_005915.6eU1809-NP_005906.2aa577LL	4	24%	9%	28%	3%	0			3	3%	1%	310	150	860	140	430	TTCCCTCGATGATATCAGAAGATATCTTCTCTTTGC	30%	25%	30%	10%				3%	4%	3%
ME1-NM_002395.6eU319-NP_002386.1aa74LL	3	11%	3%	11%	3%	3	11%	5%	0			280	280	700	260	310	TCTGAACTCTGACTTTGACAGGTATCTTCTCTTAAT	13%	7%	12%		9%	17%	7%			
MED18-NM_001127350.2eU329-NP_001120822.1aa60LF	4	9%	3%	10%	2%	0			0			320	330	570	360	420	ACTTTCCTTGACCATGAGATGGTATTCCTCCTTAAG	9%	13%	10%	6%						
MED4-NM_001270629.2eU354-NP_001257558.1aa99SF	3	3%	1%	3%	1%	0			0			330	80	680	250	440	TAGAAAAAGCAAGAAAAGGTGCTATCTCCTCTGAAG	4%	4%	2%							
METTL16-NM_024086.4eU520-NP_076991.3aa130LL	4	9%	4%	9%	5%	0			0			390	150	630	320	330	TGGAGCAACCTTGAATGGCTGGTATTTCCTCGCAAC	5%	15%	7%	8%						
METTL3-NM_019852.5eU1841-NP_062826.2aa575SF	4	18%	12%	22%	12%	0			0			260	210	790	80	350	TCAAGCAAAGGTACCCAGATGGTATCATCTCTAAAC	29%	8%	29%	8%						
MGAT5-NM_001371457.1eU533-NP_001358386.1aa12Q*	2	18%	9%	18%	9%	0			3	36%	8%	260	200	720	160	340	GCTCTCTTCACTCCGTGGAAGTTGTCCTCTCAGAAG		11%	24%					43%	27%	38%
MGC70870-NR_003682.1eU72-ncr	2	25%	8%	25%	8%	0			0			410	340	770	280	180	CCAGAACCAATTTCATTGAAGCGAACTTCCCACATC	31%		20%							
MGST3-NM_004528.4eU277-NP_004519.1aa70PP	3	3%	1%	3%	1%	1	2%		0			230	320	540	390	500	CCACCAGAACACGTTGGAAGTGTATCCTCCCTTCTT	4%	4%	2%			2%				
MGST3-NM_004528.4eU81-NP_004519.1aa5SF	4	4%	2%	5%	1%	0			3	33%	3%	340	210	470	370	460	GGTGATCCAGACGCAAGATGGCTGTCCTCTCTAAGG	6%	4%	5%	2%				37%	32%	31%
MICA-NM_000247.3eU82-NP_000238.1aa15PL	4	12%	4%	14%	1%	0			2	4%	0%	170	230	740	280	210	CGGTCTTCCTGCTTCTGGCTGGCATCTTCCCTTTTG	14%	14%	15%	6%				4%		4%
MIER1-NM_001077700.3eU3801-3'UTR+2064	4	6%	3%	7%	2%	0			0			170	230	770	250	480	ATTGTTGCAGTGTTGGAGATGCCATTTTCACCTTTT	8%	5%	8%	3%						
MIR100HG-NR_024430.2eU648-ncr	4	29%	14%	34%	12%	0			0			400	80	740	260	180	AAAGAAAGTGATCACCTGAAGACACCTCCTCTGCTA	20%	38%	43%	14%						
MKNK1-NM_001135553.4eU231-5'UTR-25	4	69%	22%	80%	6%	3	59%	8%	3	69%	2%	280	210	910	140	390	GGGATTTCAAGTTTACAGATGGTATCTTCTCAAAAG	87%	76%	77%	36%	50%	65%	61%	71%	67%	70%
MKNK1-NM_001377341.1eU189-5'UTR-108	2	58%	12%	58%	12%	1	80%		1	42%		280	210	910	140	390	GGGATTTCAAGTTTACAGATGGTATCTTCTCAAAAG	50%	67%					80%	42%		
MKNK1-NM_001377343.1eU189-5'UTR-56	4	63%	20%	72%	8%	3	63%	20%	3	67%	8%	280	210	910	140	390	GGGATTTCAAGTTTACAGATGGTATCTTCTCAAAAG	64%	78%	75%	35%	78%	40%	70%	67%	75%	60%
MLXIP-NM_014938.6eU4598-3'UTR+1740	2	8%	3%	8%	3%	0			0			160	420	790	290	270	GACCCGGGGATGAGCATGAAGGCATTGTCCCAAAGG		9%	6%							
MMD-NM_012329.3eU745-NP_036461.2aa202PL	2	5%	1%	5%	1%	0			0			180	290	730	220	230	TTGTGTTCTTCAAGAGTGATGGCATCATTCCATTTG		6%	5%							
MOB1A-NM_001317110.2eU3652-3'UTR+2799	4	41%	13%	47%	4%	3	1%	0%	3	9%	1%	470	80	840	250	250	TTCCCCCTTGCTAATCAGAAGCCATCTCCTCTCAGT	52%	44%	45%	21%	2%	1%	2%	7%	9%	10%
MOB1A-NM_001317110.2eU815-NP_001304039.1aa204Q*	4	4%	1%	4%	1%	0			0			350	200	680	260	160	CTGATTGATAGGCGTGAGCTGGCACCTCTTCAAGAA	4%	4%	6%	2%						
MOCOS-NM_017947.4eU1005-NP_060417.4aa321SL	2	5%	1%	5%	1%	0			0			210	160	820	170	220	TAGCTCAGAGGTTTGAAGATGGCACCATCTCATTCC		6%	5%							
MOCS3-NM_014484.5eU1398-NP_055299.1aa458PL	2	10%	5%	10%	5%	0			0			510	290	650	410	80	CCTGGGCTGCCAAAATCGATGGAACATTTCCACAGT	6%	13%								
MON2-NM_001278469.3eU3602-NP_001265398.1aa1074HY	2	7%	1%	7%	1%	0			1	6%		280	410	660	320	230	TGGCACACTGTTATCTGGAAGGTACTCTTTCATCTA		8%	6%							6%
MPHOSPH8-NM_017520.4eU539-NP_059990.2aa150PL	4	16%	8%	20%	7%	0			0			350	260	700	470	420	AGCAAAGTGAGACAAAAGAAGATACTTCCCCAAAGA	14%	28%	17%	7%						
MR1-NM_001194999.2eU1428-3'UTR+338	2	14%	4%	14%	4%	0			0			300	350	840	380	110	GGAGATTCTCAGAGCAGGAAGGAATCTTTTCAACCA	17%		11%							
MRFAP1L1-NM_203462.3eU714-3'UTR+94	2	2%	1%	2%	1%	0			0			230	130	610	290	270	TGAGGAAACTGAAACAGCTTGTCATTTTCTCTGTTT		3%	2%							
MRPL20-NM_001318485.2eU849-3'UTR+464	4	14%	7%	17%	5%	0			0			300	510	780	250	170	GGGATGGGAAGGAACCTGAAGGCATTTTTTCCAGAG	17%	22%	12%	6%						
MRPL20-NM_017971.4eU504-NP_060441.2aa143SF	4	19%	5%	21%	3%	0			0			300	510	780	250	170	GGGATGGGAAGGAACCTGAAGGCATTTTTTCCAGAG	24%	18%	21%	12%						
MRPL24-NM_024540.4eU596-NP_078816.2aa167PL	4	8%	3%	9%	1%	0			0			200	230	750	180	210	CCGAATTTCCCAGAGCTGATGGCATCGTCCCTGAAA	10%	8%	8%	4%						
MRPL37-NM_001330602.1eU1377-NP_001317531.1aa434LF	3	11%	2%	11%	2%	0			0			430	180	520	540	360	CAAATATTTAAAGTGAGATGGATACCTTCCCTCCCA	11%	9%	13%							
MSH3-NM_002439.5eU2886-NP_002430.3aa937TI	4	16%	6%	19%	2%	0			0			170	270	820	290	320	CGACAATTGGGATTGTGGATGGCATTTTCACAAGGA	19%	17%	21%	7%						
MSH5-NM_002441.5eU2127-NP_002432.1aa633TI	2	5%	0%	5%	0%	0			0			270	70	870	180	340	CCGAAATTGGGGCAGTAGACGCCATCTTCACACGAA		5%	6%							
MSI2-NM_001322250.2eU647-NP_001309179.1aa171PL	4	26%	11%	28%	12%	0			2	6%	1%	270	220	860	280	330	AGAAAGCTCAGCCGAAAGAAGTCATGTTCCCACCTG	15%	29%	39%	20%				5%	6%	
MSI2-NM_001322251.2eU798-NP_001309180.1aa193PL	3	43%	3%	43%	3%	0			0			270	220	860	280	330	AGAAAGCTCAGCCGAAAGAAGTCATGTTCCCACCTG	45%	40%	43%							
MSL3-NM_001193270.2eU1589-NP_001180199.1aa488PL	2	3%	2%	3%	2%	0			0			260	140	750	340	290	TTTTAGCAGAATACCACGATGACTTCTTCCCAGAGT		2%	4%							
MSL3P1-NR_024322.1eU1845-ncr	2	22%	1%	22%	1%	0			3	17%	9%	310	160	680	250	200	AGCTATAATTTGCTTCTGACGGTTCCTTCTCTGTGA	22%	21%						10%	27%	15%
MTF2-NM_001164391.2eU2879-3'UTR+933	3	6%	1%	6%	1%	0			0			340	260	730	310	120	AACATTATCTCCTACTAGAAGTAACGTTTTCAAGTT	7%	6%	4%							
MTMR6-NM_001385230.1eU2004-NP_001372159.1aa588TI	4	27%	12%	32%	7%	0			0			160	360	790	80	450	GCAAAAATAAGCAAACAGATGGCATCCTCACCAAGG	30%	40%	26%	11%						
MXRA8-NM_001282582.2eU430-NP_001269511.1aa121SL	4	7%	3%	9%	2%	0			0			330	240	800	180	140	CGGCCTCGGCCTTCGACGACGGCAACTTCTCGCTGC	11%	9%	7%	3%						
MYBBP1A-NM_001105538.2eU905-NP_001099008.1aa282PL	2	4%	2%	5%		0			0			310	300	760	360	260	TCCGCCTGGCACTCAAGGAAGACAAGTTCCCACGGT	5%			3%						
MYBL1-NM_001080416.4eU4180-3'UTR+1541	4	12%	4%	14%	2%	0			0			230	290	800	180	180	AAAGATAAAAAACAAATGATGGCAAGTTCTCAAGTT	14%	16%	12%	7%						
MYD88-NM_001172566.2eU747-3'UTR+266	4	3%	2%	4%	2%	0			0			220	150	810	180	350	ACCTCTCAATTCCTGGAGATGCCAACTTCACAGACA	4%	5%	2%	2%						

MYH10-NM_001256012.3eU2201-NP_001242941.1aa700R*	3	6%	3%	8%	1%	0			0			340	350	630	350	0	GAATCTCTCACCAAGCTGATGGCAACTCTCCGAAAC		7%	9%	4%						
MYH10-NM_001256012.3eU3306-NP_001242941.1aa1068SL	4	3%	2%	3%	2%	0			0			310	190	790	120	290	AAATCAGGAATAAGCAAGAAGTGATGATCTCAGATT	5%	2%	2%	3%						
MYH10-NM_001256095.2eU2135-NP_001243024.1aa678R*	2	12%	2%	12%	2%	0			0			340	350	630	350	0	GAATCTCTCACCAAGCTGATGGCAACTCTCCGAAAC		13%	10%							
MYL6B-NM_001199629.2eU179-NP_001186558.1aa8PP	2	4%	0%	4%	0%	0			0			300	510	560	240	420	GGACATCATGCCTCCCAAGAAGGATGTTCCCGTGAA		3%	4%							
MYL6B-NM_002475.5eU115-NP_002466.1aa8PP	4	3%	1%	3%	2%	0			0			300	510	560	240	420	GGACATCATGCCTCCCAAGAAGGATGTTCCCGTGAA	4%	1%	4%	3%						
MYOF-NM_013451.4eU2713-NP_038479.1aa858TI	4	4%	2%	4%	1%	0			0			210	400	750	340	330	AGTTTAACAGCTTCGCAGAAGGAACTTTCACCGTCT	3%	4%	6%	1%						
MYOF-NM_013451.4eU2838-NP_038479.1aa900LL	2	2%	0%	2%	0%	0			0			420	280	740	460	290	GGAAAAAATAAAACTCAAGAGGGAATTTTTCTGCCT		2%	2%							
MYOM3-NM_152372.4eU3764-NP_689585.3aa1214TI	2	11%	2%	9%		0			0			220	170	780	260	260	TCACGGGTGACGCCCTGGATGCCATCTTCACCGAGT		9%		12%						
NACC1-NM_052876.4eU2066-3'UTR+283	3	3%	0%	3%	0%	0			0			280	0	730	170	300	CCCCAACCTAACCCCTAGCCGTCATCTCCACACTCA	3%	3%	3%							
NAIF1-NM_197956.4eU257-NP_931045.1aa13SL	3	6%	2%	7%	1%	0			0			530	270	600	350	340	TCCCAGCCAAGAAAAGGAAGATGAACTTCTCAGAGC	8%	7%		4%						
NARS1-NM_004539.4eU1814-3'UTR+48	2	2%	0%	2%	0%	0			0			330	210	540	370	180	GTGGAGGAAAGATTATGAAAGGAACAGGCTCTTTAA	2%		2%							
NASP-NM_001195193.2eU2028-NP_001182122.1aa641SL	4	3%	1%	4%	1%	0			3	40%	2%	360	150	660	330	340	CCAGTAGAAAGCCAACAGACGGTGCTTCCTCATCAA	3%	5%	4%	1%				37%	41%	41%
NASP-XM_011541509.2eU2290-XP_011539811.1aa707SL	2	7%	2%	7%	2%	0			3	46%	17%	360	150	660	330	340	CCAGTAGAAAGCCAACAGACGGTGCTTCCTCATCAA		8%	6%					29%	44%	63%
NBPF9-NM_001037675.4eU4135-NP_001032764.2aa941PL	4	10%	4%	10%	4%	0			0			400	220	820	320	250	ACCTGGTGTTCCAGATGGAAGTCATATTCCCACAAT	15%	7%	9%	10%						
NCOR2-NM_001077261.4eU8581-3'UTR+735	3	2%	0%	2%	0%	0			0			340	460	570	320	330	TCAGATGTTTTACGCAAGGAAGGACTTACCCAGTAT	2%	2%	1%							
NDC1-NM_001168551.2eU933-NP_001162023.1aa300Q*	4	8%	3%	9%	2%	0			3	72%	1%	310	180	640	260	260	TTAGCCTTGCAGGACCTGATGTTGCTTTCTCAATAT	11%	8%	9%	3%				71%	71%	72%
NDE1-XM_017023349.2eU1083-XP_016878838.1aa329HY	2	4%	1%	4%	1%	0			3	35%	6%	400	150	730	310	270	AAGCGCCTGGAATTTGGGAAGCCGCCTTCACACATG	4%		3%					34%	30%	41%
NDUFB7-NM_004146.6eU143-NP_004137.2aa26PL	4	31%	9%	36%	2%	0			3	8%	1%	350	180	430	620	260	AGCCCGACCCCCTGCAGATGCCAACCTTCCCGCCAG	37%	35%	34%	18%				9%	7%	9%
NDUFS1-NM_001199981.2eU1754-NP_001186910.1aa549PL	3	3%	1%	3%	1%	0			0			110	250	670	250	400	TTGGGGCTCCCATAGCTGATGTTATTCTCCCAGGAG	2%	2%	4%							
NDUFS3-NM_004551.3eU302-NP_004542.1aa95PL	4	6%	2%	7%	1%	0			3	49%	2%	150	230	720	180	210	AGGTCTGTATCCATCCTGATGGCGTCATCCCAGTGC	8%	5%	7%	3%				50%	47%	51%
NEDD8-NM_006156.3eU213-NP_006147.1aa38PL	4	2%	1%	2%	0%	0			0			390	210	740	460	400	AGCGTGTGGAGGAGAAAGAGGGAATCCCCCCACAAC	3%	3%	2%	1%						
NEK7-NM_133494.3eU3154-3'UTR+1938	3	4%	1%	4%	1%	0			0			310	70	770	220	450	TAATGTAATATAATTGAGATGAAATGTTCTCTGGTT	5%	4%	3%							
NFE2L2-NM_001145412.3eU2460-NP_001138884.1aa576FF	3	2%	1%	2%	1%	0			0			340	270	500	550	190	CCTGCAGCAAACAAGAGATGGCAATGTTTTCCTTGT	2%	1%	2%							
NHLRC2-NM_198514.4eU1033-NP_940916.2aa274SL	4	20%	14%	24%	15%	0			0			430	220	790	300	230	CCAACCCTGGAAGAAAAGATGGAATATTTTCAGAAT	38%	9%	25%	11%						
NHP2-NM_001034833.2eU525-3'UTR+184	4	41%	14%	48%	1%	3	7%	1%	3	19%	2%	170	230	870	240	250	TCCTGCCCACCCACACTGACGGCATCTTCCCAGTTC	49%	49%	47%	20%	8%	6%	8%	19%	18%	21%
NHP2-NM_017838.4eU631-3'UTR+101	4	46%	13%	52%	2%	3	7%	3%	3	26%	5%	170	230	870	240	250	TCCTGCCCACCCACACTGACGGCATCTTCCCAGTTC	55%	52%	51%	27%	4%	9%	8%	23%	23%	31%
NICN1-NM_032316.3eU198-NP_115692.1aa38PL	3	19%	11%	26%	4%	0			0			210	290	720	290	180	CTGGCGTGCTGGTCATCGATGTCACCTTCCCCAGCG	29%	23%		7%						
NMD3-NM_015938.5eU2431-3'UTR+800	4	59%	17%	67%	6%	3	5%	2%	3	21%	2%	370	300	850	290	430	TTTTGTTTATAAATTGAGAAGGAATTTTCTCTCTAT	73%	62%	67%	35%	7%	5%	3%	23%	22%	20%
NNT-NM_001331026.2eU2278-NP_001317955.1aa649LL	2	6%	4%	8%		0			0			270	280	780	340	160	CCCTCTCCTACTGCCTGGAAGGCACTTACTCAATGC	8%			3%						
NOL8-NM_001256394.2eU4002-3'UTR+301	3	11%	2%	11%	2%	3	8%	3%	1	3%		310	110	750	270	450	TCTATCCTGAGTAACTAATGGACATCTTCTCATGCA	9%	12%	13%		10%	5%	8%	3%		
NOLC1-NM_001284388.2eU2990-3'UTR+838	4	9%	3%	10%	2%	3	21%	2%	1	1%		230	160	770	220	210	TTGAGTTCTAAGTTGAGGACTGCATCTTCTCGTTTT	8%	12%	11%	5%	20%	23%	20%			1%
NONO-NM_001145408.2eU2435-3'UTR+752	4	63%	19%	73%	2%	3	6%	1%	3	20%	0%	360	210	850	250	470	CCCCTTCATGTCCTAAAGAAGACATTTTCTCTTAGA	74%	73%	70%	35%	7%	7%	6%	21%	21%	20%
NPIPA1-NM_006985.4eU323-NP_008916.2aa108TI	3	3%	1%	3%	1%	0			0			320	210	750	260	240	GAATTAAAATTGTCCTGGAAGACATCTTTACTTTAT	3%	3%	4%							
NPIPA5-NM_001277325.2eU323-NP_001264254.1aa108TI	3	2%	0%	2%	0%	0			0			320	210	750	260	240	GAATTAAAATTGTCCTGGAAGACATCTTTACTTTAT	2%	2%	3%							
NPPB-NM_002521.3eU137-NP_002512.1aa9RW	4	4%	1%	5%	0%	3	37%	1%	0			290	270	710	250	220	AGAGACATGGATCCCCAGACAGCACCTTCCCGGGCG	5%	5%	5%	2%	36%	37%	38%			
NPPB-NM_002521.3eU658-3'UTR+141	3	2%	0%	2%	0%	0			0			210	230	670	340	250	TTGATTGTTTTATATAAGATGGTTTCTTACCTTTGA	2%	2%	2%							
NR1D2-NM_001145425.2eU1210-NP_001138897.1aa290Q*	4	11%	3%	13%	2%	0			0			330	460	610	250	400	TGTGATAGAGTTCCGATAGATGGATTTTCTCAGAAT	15%	12%	11%	7%						
NR2C2-NM_001291694.2eU2735-3'UTR+581	3	11%	3%	11%	3%	0			0			270	420	760	260	230	ATTTATGTTTAAGAAAAGAAGGCATCATTCCTAATT	11%	14%	8%							
NRF1-NM_001040110.2eU1063-NP_001035199.1aa318SL	2	8%	0%	8%	0%	0			0			310	220	730	200	200	AGACTTTTAGTAACCCTGATGGCACTGTCTCACTTA		8%	8%							
NSFL1C-NM_001206736.2eU1307-3'UTR+163	2	2%	0%	2%	0%	0			3	46%	1%	310	270	400	380	490	TCTTAGATCTTGGTTGGACGTTTGTTTTCTCCTTAG	2%		2%					47%	45%	47%
NT5C2-NM_001134373.3eU1571-NP_001127845.1aa504LF	2	3%	1%	3%	1%	0			3	32%	6%	360	90	720	290	330	CACGTAGATATCAATGAGATGGAGTCTCCTCTTGCC		2%	4%					25%	35%	37%
NUBP1-NM_001278506.2eU1093-3'UTR+141	4	23%	6%	26%	1%	0			2	4%	0%	320	210	800	300	200	AGATGCTGGTGTGGTCCGAAGCCACTTTCTCAGAGA	27%	27%	25%	14%					4%	4%
NUBP1-NM_001323594.2eU973-3'UTR+141	2	56%	19%	56%	19%	0			0			320	210	800	300	200	AGATGCTGGTGTGGTCCGAAGCCACTTTCTCAGAGA	43%	69%								
NUDT5-NM_001321647.2eU194-NP_001308576.1aa11Q*	4	6%	2%	7%	1%	0			0			280	200	790	300	290	ATGGAGAGCCAAGAACCAACGGAATCTTCTCAGAAT	6%	7%	8%	3%						
NUFIP2-NM_020772.3eU3300-3'UTR+1123	2	5%	2%	5%	2%	0			3	66%	3%	340	300	750	180	250	AATAAACCCTGACTGTAGAAGGTGTGTTTTCATGCA		7%	4%					69%	66%	63%
NUP62-NM_012346.5eU165-5'UTR-69	3	19%	7%	19%	7%	0			0			210	270	870	360	410	GGCCTCGGCTGAAGAAAGAAGAAATCTTCCCAAGGC	12%	23%	24%							
OCRL-NM_000276.4eU3586-3'UTR+699	4	21%	11%	26%	4%	0			0			280	140	770	210	270	CTGTAGGGACTATTAGAGATGGCATCTCATCGATGA	27%	30%	23%	6%						
OGDH-NM_001003941.3eU127-NP_001003941.1aa25Q*	2	3%	0%	3%	0%	0			0			260	260	740	210	480	ACGGCTTCCCAGACTGTTAAGACATTTTCACAAAAC		3%	2%							
ORC2-NM_006190.5eU97-5'UTR-135	3	7%	3%	7%	3%	0			0			250	310	760	290	200	CTTTTCCCGTTGGGGCCGAAGGTACCTTCCCTGCGG	9%	7%	4%							
OSBPL9-NM_001330580.2eU2421-3'UTR+253	4	5%	2%	5%	1%	0			3	68%	5%	310	170	740	140	430	TCAGGTTTTGAAAATCAGATGATGTCTTCTCCTTTT	5%	7%	4%	3%				68%	64%	73%
OSBPL9-NM_001350208.2eU225-5'UTR-538	3	4%	2%	5%	1%	3	44%	4%	0			290	250	720	190	190	GTATAGACGATGAGGACGACAGCACCTTCACAATAA	5%	4%		2%	40%	46%	47%			
OSGIN1-NM_182981.3eU309-NP_892026.1aa49PL	4	10%	4%	11%	3%	0			0			260	120	740	220	320	CACCCTACACGAAGCCAGATGCCATCCACCCACACC	13%	14%	8%	5%						
OXA1L-NM_005015.5eU630-NP_005006.4aa206Q*	4	5%	3%	6%	4%	0			0			260	290	730	360	260	TACAAGGCTTCCTCGGAGATGGCACTTTACCAGAAA	10%	4%	3%	3%						
PAN2-NM_001127460.4eU534-NP_001120932.2aa63SF	3	8%	2%	9%	2%	0			3	39%	6%	360	170	720	220	270	AATCAGTGCACATAATGGAAGGTGTCTACTCTGAAT		8%	11%	7%				43%	32%	42%
PAPOLA-NM_001252006.1eU607-NP_001238935.1aa130FF	4	2%	1%	2%	1%	0			0			530	310	690	300	140	ACCAAGACATGTTGATCGAAGTGACTTTTTCACCTC	3%	2%	2%	2%						
PAPOLA-NM_001293627.1eU3572-3'UTR+1120	3	3%	1%	3%	1%	0			0			280	490	810	330	390	GTTTTAAATGTGGGCAAAAAGGCATTTTCTCCAAGA	4%	3%	3%							
PARP14-NM_017554.3eU3871-NP_060024.2aa1276Q*	2	2%	0%	2%	0%	0			0			310	200	780	390	490	GCTGGACAAAATGTAGAAAGGGAATGTTCTCAGCAA		2%	2%							
PARP9-NM_001146103.2eU525-NP_001139575.1aa30PP	2	4%	1%	5%		0			0			210	310	720	200	260	TCTTGGAGAAAACTATAGTTGGCAAATTCCCATTAA			5%	4%						
PARP9-NM_001146106.3eU4814-3'UTR+2402	3	40%	11%	40%	11%	0			0			170	170	890	180	300	TTGGTTGAGTGCAGCAAGAAGGCATCTACACATGCA	44%	48%	28%							
PATL1-NM_152716.3eU2845-3'UTR+390	4	8%	4%	10%	0%	1	3%		3	78%	2%	340	270	670	250	270	CCTTCCCTCATCCTATTGAAGTTGTTTTTTCATTGC	10%	9%	10%	3%		3%		80%	76%	77%
PAWR-NM_001354732.2eU1585-3'UTR+389	4	30%	8%	34%	5%	0			2	6%	1%	210	70	730	270	330	AGTTCAAATACTTTTGGGATGTTACCTTCACATGTC	35%	39%	29%	20%				6%		5%
PAXBP1-NM_016631.4eU2459-NP_057715.2aa791SL	4	10%	3%	8%	1%	1	4%		0			280	270	780	290	350	GCAATTTTCTTCAGTGGTATGGCATTTTCTCAAATA	10%	7%	8%	13%			4%			
PBX2-NM_002586.5eU2743-3'UTR+1163	3	12%	1%	12%	1%	0			0			170	240	820	320	290	ATTTTGAGGAAATTGAAGATGCCATCTTCCCCTGTG	13%	12%	10%							
PCLAF-NM_001029989.3eU762-3'UTR+494	4	44%	18%	53%	6%	0			3	21%	16%	350	110	620	350	510	ATGTTGGAAAACCTTGGATGGATATCTTCTCTTTAA	59%	47%	52%	17%				8%	38%	16%
PCLO-NM_014510.3eU14767-NP_055325.2aa4825LL	4	7%	4%	9%	4%	0			0			180	340	760	80	510	CCTCGATAACACTCCAAGGTGGTATCCTCTCAAAGA	10%	12%	4%	2%						
PCLO-NM_014510.3eU14874-NP_055325.2aa4861PL	4	3%	2%	4%	2%	0			0			260	230	700	240	390	TTATCAAAAGCAGAAGCCATGGTATCTTCCCTGACC	5%	2%	5%	2%						
PCSK9-NM_174936.4eU2921-3'UTR+552	3	2%	0%	2%	0%	0			0			220	160	740	320	70	GGAGGCTTAGCTTTCTGGATGGCATCTAGCCAGAGG	2%		2%	2%						
PCYT1A-NM_001312673.2eU727-NP_001299602.1aa202TI	4	32%	8%	36%	2%	0			3	12%	4%	220	170	850	210	400	CTCCAACACAGAGGACAGAAGGTATCTCCACATCAG	38%	35%	34%	21%				13%	15%	7%
PDCD10-NM_007217.4eU714-NP_009148.2aa77TM	3	10%	0%	10%	0%	0			0			220	230	720	220	250	TAGAGAAAAAAAAGCGTGGAAGTAACTTCACGGAGT	10%	10%	10%							
PDE4DIP-NM_001002811.2eU2147-NP_001002811.2aa497PL	2	3%	1%	2%		0			0			410	280	630	560	240	GACAACTTCACAGCTCAGAGGGTACTTCTCCAGCTC		2%		3%						
PDHB-NM_000925.4eU1300-3'UTR+197	2	3%	1%	3%	1%	0			0			220	250	630	260	260	TCCACTCTCCTCCCCTAGATGCCATGCTTCCTTTTG		2%	3%							
PDHB-NM_000925.4eU189-NP_000916.2aa56LF	3	4%	2%	4%	2%	0			0			330	290	680	320	350	GAGCTGGAAAGAGATGAGAAGGTATTTCTGCTTGGA	3%	4%	6%							
PDLIM5-NM_001011513.4eU1401-NP_001011513.4aa435PP	3	6%	2%	6%	2%	0			0			310	320	680	390	240	TGGTACTATATGCCATGGATGTGAATTTCCCATAGA	6%	4%	8%							
PEG10-NM_001040152.2eU6371-3'UTR+4959	4	2%	1%	2%	0%	0			0			380	300	690	260	140	TGAACTTGTCAATGATGGATGGTAAATTTTCATGGA	2%	2%	2%	1%						
PERP-NM_022121.5eU1349-3'UTR+688	4	23%	7%	26%	5%	0			0			310	130	770	290	430	TAAGTGTGAAATATTTAGATGAAATTTTCTCTTTTA	31%	27%	20%	14%						
PEX13-NM_002618.4eU495-NP_002609.1aa152SL	3	9%	2%	9%	3%	0			0			340	130	700	350	140	CTGTCAGTATGATGATGGATGCTACCTTTTCAGCTG	7%		11%	8%						
PEX1-NM_000466.3eU1586-NP_000457.1aa501LL	2	26%	5%	26%	5%	0			1	9%		320	300	810	290	310	GAAACTAAAGATGGACTGAAGGAATTTTCTCTGAGT		23%	30%					9%		
PGBD4-NM_152595.5eU2024-NP_689808.2aa525PP	2	32%	25%	32%	25%	0			0			260	260	760	460	310	ACCTCTTCGTCTGTCTGGAAGACATTTCCCCAAGAG	50%		14%							
PHF12-NM_001033561.2eU4243-3'UTR+670	3	16%	3%	16%	3%	0			0			340	240	750	250	370	GTGTAAATACATCTAGTGATGACATTTTTTCAATGT	19%	15%	15%							
PHF20-NM_016436.5eU447-NP_057520.2aa117TI	2	5%	0%	5%		0			0			250	150	610	380	310	TCACTGCTGTTAACAAGGATGGTACTTACACTGTGA		5%		6%						
PHLDB1-NM_001144759.3eU4032-NP_001138231.1aa1309TI	3	5%	2%	5%	2%	0			0			110	130	710	230	360	CCATGCGTATCTGGATGGATGTCATTGTCACAGGGG	6%	3%	6%							

PHOSPHO2-NM_001199290.3eU2840-3'UTR+803	2	29%	4%	29%	4%	0			0			350	210	860	350	370	GGAATTTGACATTTAAAGAGGGAATCTTCTCTACCT	31%		26%							
PI4K2B-NM_018323.4eU499-NP_060793.2aa123PL	2	6%	0%	6%	0%	0			0			250	350	670	430	250	CAGAGCAAGCAATAGAAGTTGGAATTTTTCCAGAAA	6%	6%								
PIAS3-NM_006099.3eU715-NP_006090.2aa208PP	3	10%	8%	10%	8%	0			0			430	380	520	520	530	GACCAGCTGCCCCCAGGAAGATTATTTTCCCCCCAA	19%	5%	5%							
PIBF1-NM_001349655.2eU371-NP_001336584.1aa36SL	4	22%	11%	27%	3%	0			0			320	60	720	360	410	AAACAACAGTTCCTACGGATGATATTTCCTCATCAG	30%	29%	24%	7%						
PIM3-NM_001001852.4eU1690-3'UTR+533	4	8%	3%	9%	1%	0			0			450	60	670	370	330	TGTTTAATTTATTTGTTGAGGTTATTTCCTCTGAGC	7%	10%	10%	3%						
PLAAT3-NM_001128203.2eU567-NP_001121675.1aa155SL	4	8%	3%	9%	2%	0			1	1%		190	280	780	100	340	CAGCCATGAGCCTTATTGGAGTCATGTTCTCAAGAA	6%	8%	11%	4%				1%		
PLCXD1-NM_001370370.1eU4325-3'UTR+3090	2	15%	2%	15%	2%	0			0			360	530	520	390	400	TTTTTTTTGTTTTTTTGAGATGGATTTTCCCTCTTG	14%	17%								
PLK2-NM_001252226.2eU2014-NP_001239155.1aa629LL	4	3%	1%	3%	0%	0			0			570	150	600	400	400	CATCTGTAGCCAAAATGAAGAATACCTTCTCACCTA	3%	3%	2%	2%						
PLPBP-NM_001349346.2eU2322-3'UTR+1449	4	24%	9%	28%	5%	0			3	24%	6%	320	220	770	270	350	TTGTTTTTTATTTTCCAGATGGCTTTTTCTCTTATT	34%	24%	26%	13%				30%	19%	24%
PLXNB2-NM_001376864.1eU1412-NP_001363793.1aa427SL	4	12%	8%	15%	7%	1	4%		3	7%	2%	370	90	830	260	180	AGGTGTACCTCACCCCAGATGGCACCTCCTCAGAGT	22%	16%	8%	4%	4%			5%	6%	9%
PM20D2-NM_001010853.3eU2104-3'UTR+703	4	10%	6%	13%	4%	0			0			220	220	730	380	330	TTTGTATTTTTAGTAGAGACGGCATTTCACCATGTT	16%	9%	13%	3%						
PMEPA1-NM_001255976.2eU1712-3'UTR+664	3	29%	12%	29%	12%	0			0			270	240	760	220	140	TATGTCAGTGCTTGCTTGATGGAAACTTCTCTTGTG	35%	16%	36%							
PNISR-NM_001322405.2eU1935-NP_001309334.1aa578RR	2	3%	1%	3%	1%	0			0			160	260	750	190	480	TAGACGTAGCAGGAGTAGAAGCTATTCTCGCAGAAT		3%	2%							
PNO1-NM_001329916.2eU339-NP_001316845.1aa98PS	3	2%	0%	2%	0%	0			0			320	240	710	250	370	TTGAAAGAAAACTGGATGAAGATATTTACTCCTATT	1%	1%	2%							
PNPT1-NM_033109.5eU856-NP_149100.2aa279PS	4	8%	3%	9%	1%	0			0			170	310	710	310	370	ACCAAGAGGACACCTCAGAAGTTATTTACCCCTTCG	9%	10%	9%	4%						
POLDIP2-NM_001290145.2eU198-NP_001277074.1aa40SL	2	1%	0%	1%	0%	0			3	11%	2%	370	160	640	230	300	TCTGTGCGGCGGCCGGAGCTGGAGCCTTCTCGCCAG	1%	1%						14%	9%	10%
POLDIP2-NM_001290145.2eU573-NP_001277074.1aa165PL	4	6%	2%	7%	2%	0			1	1%		200	370	750	220	360	TGGACTATGTCAGCCATGAAGACATCCTCCCCTACA	9%	6%	5%	5%				1%		
POLE-NM_006231.4eU5024-NP_006222.2aa1666TI	4	2%	0%	2%	0%	0			0			340	0	810	300	420	CCATTGGGAACCTACCAGAGGACATCTCCACATTCG	3%	2%	2%	2%						
POLK-NM_001345921.2eU558-NP_001332850.1aa157II	3	9%	7%	12%	8%	0			0			330	280	770	320	310	TTGGCCTGAGGATAAAAGAAGGTATTTCATCAAAAT		18%	7%	4%						
POLM-NM_001284331.2eU1392-NP_001271260.1aa434Q*	4	33%	18%	39%	16%	0			0			280	390	790	350	330	CTGTTTGACCCGGAGCAGAAGACATTTTTCCAAGCG	36%	25%	56%	14%						
POLR1D-NM_001206559.2eU645-NP_001193488.1aa63YY	4	6%	2%	7%	2%	0			0			180	310	770	160	350	AAACCCGAAGAAACACAGAAGCCATCCTTACAAGCA	5%	7%	8%	3%						
POLR2C-NM_032940.3eU216-NP_116558.1aa57SL	4	3%	1%	3%	1%	0			0			280	140	700	320	200	TTGACTGGGTTCAGATTGATGCCAATTCCTCAGTTC	3%	2%	5%	3%						
POU2F2-NM_001207025.4eU5695-3'UTR+4220	4	37%	19%	40%	22%	3	38%	8%	1	35%		390	80	460	190	90	GACCTGACTAGTTTTTCTAACCCACATGAACACCAG	40%	19%	63%	27%	38%	47%	31%	35%		
PPP1R12B-NM_001197131.1eU7564-3'UTR+6977	4	12%	5%	11%	5%	0			3	63%	13%	290	270	580	490	350	TTTATAGCTTAAAACAGAAGTGTGTCTTCCCAGCAC	12%	15%	6%	16%				69%	48%	71%
PPP1R15B-NM_032833.5eU1648-NP_116222.4aa414PP	4	15%	9%	18%	8%	0			3	6%	2%	380	220	650	580	220	TGATTACTCATACCTAGAAGGTGACCTTCCCATTTC	27%	16%	11%	5%				4%	6%	8%
PPP1R2-NM_001291504.2eU3310-3'UTR+2389	4	30%	10%	34%	7%	0			0			170	350	770	390	350	GTTGAAGATTAAAGCAAGATGCCATTTTCCCCATGA	43%	29%	30%	19%						
PPP3R1-NM_000945.4eU729-NP_000936.1aa108SS	4	4%	1%	5%	1%	0			0			360	290	510	570	290	CTATGACATGGATAAAGATGGCTATATTTCCAATGG	6%	3%	5%	3%						
PPP4R3A-NM_001284280.2eU1301-NP_001271209.1aa213SF	4	54%	13%	61%	3%	0			3	8%	3%	320	150	770	140	270	ATCGAACTGCTCTTTTTGAAGTTATGTTCTCTGAAG	60%	64%	58%	34%				7%	11%	6%
PPRC1-NM_001288727.2eU3141-NP_001275656.1aa1038LF	2	2%	0%	2%	0%	0			0			450	160	620	420	100	AATGTACTTCCCTTGTCGATGGCTCCTCCCCTCAGT		2%	2%							
PRELID3A-NM_001142405.2eU439-NP_001135877.1aa131TI	2	11%	1%	11%	1%	0			0			150	150	790	160	340	TGACCGTGCTCACACAAGAAGCCATCATCACTGTGA	12%	10%								
PREX1-NM_020820.4eU6261-3'UTR+1142	4	34%	9%	39%	5%	0			2	17%	4%	280	240	810	140	200	CTGTGATTTATTTTCTTGATGGTAACTTCTCAGAGC	41%	33%	42%	22%				14%		20%
PRIM2-NM_000947.5eU1480-NP_000938.2aa467Q*	4	4%	2%	5%	1%	0			0			280	350	720	450	120	GGTGGTAAAGACATAAAGAAGGAACCTATCCAACCA	6%	4%	5%	2%						
PRKAG1-NM_001206709.2eU56-NP_001193638.1aa7SL	3	14%	7%	14%	7%	0			0			440	160	530	570	210	CGCCTCTTGCAATGGAGACGGTCATTTCTTCAGATA	11%	22%	10%							
PRKAR1A-NM_001276289.2eU722-NP_001263218.1aa155SL	4	7%	2%	8%	1%	0			0			300	130	700	220	110	AGAGAAGTGATATTTTTGATGCCATGTTTTCGGTCT	8%	9%	8%	4%						
PRPF39-NM_017922.4eU1662-NP_060392.3aa503FF	3	4%	1%	4%	1%	0			0			310	270	680	260	110	TTATGCTGTCAAACTAGCCCGGCATCTTTTCAAAAT	5%	4%	3%							
PRPF4B-NM_003913.5eU407-NP_003904.3aa94PL	4	11%	6%	14%	2%	0			0			370	220	690	480	290	TTGATGCTTCTGATAAAGAGGGTATGTCTCCAGCAA	16%	11%	14%	3%						
PRPF8-NM_006445.4eU287-NP_006436.3aa63PL	4	6%	2%	7%	1%	0			0			250	200	690	370	360	TTGTGGATGCCCAGAAGGAAGACATGCCCCCAGAAC	6%	8%	7%	3%						
PRPS1-NM_001204402.2eU1624-3'UTR+956	4	4%	1%	4%	2%	0			0			210	130	660	150	370	TCCTGTTAAAAAAGATTGATGTTATTGTCTCTTGTA	2%	5%	4%	5%						
PRR11-NM_018304.4eU208-NP_060774.2aa48SL	4	14%	7%	16%	6%	0			0			310	260	650	280	390	CACCCTCACCAGAAAGAGTCGGTATTTCTTCAATAG	23%	11%	14%	7%						
PRRC2B-NM_001384818.1eU1926-NP_001371747.1aa565Q*	4	8%	3%	9%	3%	1	2%		3	4%	1%	220	240	890	310	330	TGGTCTCCAAGTGCTGAGAAGGCATCTCCCCAGGAA	12%	8%	7%	4%			2%	4%	5%	3%
PRRC2B-NM_001384818.1eU230-5'UTR-5	3	7%	2%	7%	2%	0			0			470	60	680	280	290	GAAAAAATTCCTTACTAGATGACATTTCATCGCAAT	6%	7%	9%							
PSMD2-NM_001278708.2eU596-NP_001265637.1aa153TI	4	2%	1%	2%	1%	0			0			210	250	860	140	420	ATGACATGGAGTTGGTAGAAGACATCTTCACCTCCT	2%	3%	2%	2%						
PSMD6-NM_001271779.2eU444-NP_001258708.1aa123LL	2	3%	0%	3%	0%	0			0			400	220	500	400	180	CCTCGACTGGCAGATAGACGTGGACCTACTCAATAA		3%	3%							
PSMD6-NM_014814.3eU285-NP_055629.1aa70LL	3	3%	1%	3%	1%	0			0			400	220	500	400	180	CCTCGACTGGCAGATAGACGTGGACCTACTCAATAA	2%	4%	1%							
PTBP1-NM_002819.5eU63-NP_002810.1aa6PL	4	4%	3%	5%	2%	0			1	1%		160	290	750	330	310	CTGCTCTGTGTGCCATGGACGGCATTGTCCCAGATA	7%	3%	5%	1%					1%	
PTEN-NM_000314.8eU2338-3'UTR+281	4	48%	9%	52%	4%	0			3	30%	8%	210	290	750	400	320	CAATTAGGATTAATAAAGATGGCACTTTCCCGTTTT	56%	50%	48%	36%				35%	33%	21%
PTGES2-NM_001256335.2eU1183-3'UTR+334	3	3%	0%	3%	0%	0			3	3%	1%	300	220	670	360	170	CCTCGACAGGACAGCAGGACGGTTTGTTTTCAGTGG	3%	3%	4%					3%	3%	4%
PTPN12-NM_001131008.2eU1632-NP_001124480.1aa437SL	2	3%	2%	3%	2%	0			0			330	310	780	280	160	CCATACCTGATTTATCTGAAGGCAATTCCTCAGATA		4%	2%							
PTPN14-NM_005401.5eU8217-3'UTR+4079	2	11%	2%	11%	2%	0			0			340	190	690	420	210	TGTGGGTCCCAACACCAGATGTCATTTCTCCAGAGC		13%	10%							
PUS7-NM_001318163.1eU1642-NP_001305092.1aa545PL	4	6%	2%	7%	1%	0			0			220	120	730	240	230	TGCCCTTGCCTGGTTTCGATGTTATCTACCCAAAGC	8%	7%	6%	4%						
PUS7-NM_001318163.1eU514-NP_001305092.1aa169TI	4	6%	2%	7%	1%	0			0			360	210	790	180	320	ATGAGGAGGACCCTTCAGAAGACATATTTACAGTTT	7%	8%	7%	3%						
QRICH1-NM_001320580.2eU2146-NP_001307509.1aa570SF	4	12%	6%	15%	2%	0			0			320	230	810	210	430	TTGAAAATGGAAGGGTAGATGACATTTTCTCCGATC	13%	17%	14%	5%						
RAB35-NM_001167606.2eU204-NP_001161078.1aa34SL	3	4%	1%	4%	1%	3	32%	5%	0			390	170	710	220	360	TACTGTTGCGTTTTGCAGACAACACTTTCTCAGGCA		5%	4%	3%	36%	35%	26%			
RAB3IP-NM_001024647.3eU712-NP_001019818.1aa125PL	4	43%	12%	49%	3%	0			3	9%	1%	280	280	760	400	250	TAGACAAAATCTACCAGGAAGATATCTTTCCATGTT	48%	52%	47%	25%				7%	9%	10%
RABGAP1-NM_012197.4eU683-NP_036329.3aa187SL	2	5%	2%	5%	2%	0			0			280	180	670	170	250	AGTGTCAGATTTCACTAGATGTTACCCTTTCAGTGC		6%	4%							
RABIF-NM_002871.5eU1793-3'UTR+1370	3	19%	2%	19%	2%	0			2	16%	10%	360	260	480	540	320	CCTACTCCCCTGGAAAGATGGCTTCCTTCTCCAATT	19%	18%	21%					24%	9%	
RABIF-NM_002871.5eU246-NP_002862.2aa65LL	4	10%	4%	11%	2%	0			0			410	230	560	460	250	TGACGGCAGCAATCCTGACGGCGATCTCCTCCAGGA	13%	9%	13%	5%						
RACGAP1-NM_001126103.3eU2433-3'UTR+243	2	3%	0%	3%	0%	0			0			240	360	680	240	480	TGTTGGGAGCTTTTAGATGAGACATCTTTCCAGGGG		2%	3%							
RAD51C-NM_002876.4eU249-NP_002867.1aa69LL	2	3%	1%	3%	1%	0			0			450	330	730	150	360	AACTCTGCAAATTATCAGAAGAGAATGTCTCACAAA		3%	2%							
RAD51-NM_001164269.2eU1136-NP_001157741.1aa329AV	2	2%	0%	2%	0%	0			0			170	300	730	180	270	CCTGTCTTCCTGAAGCTGAAGCTATGTTCGCCATTA	3%		2%							
RAF1-NM_001354689.3eU477-NP_001341618.1aa49TI	2	3%	1%	3%	1%	0			0			240	290	690	120	260	AGCGCCGGGCATCAGATGATGGCAAACTCACAGATC	2%	3%								
RALGDS-NM_001042368.3eU2822-3'UTR+12	3	8%	2%	8%	2%	0			0			190	280	800	270	320	TGCCAAGGGCATCTTCTGAGGGCATCCTCCCAGGGT	10%	8%	5%							
RALY-NM_007367.4eU441-NP_031393.2aa43SF	4	1%	0%	1%	0%	3	5%	0%	0			480	160	730	310	250	TGAAGAAATCAGATGTGGAGACCATCTTCTCTAAGT	1%	2%	1%	1%	5%	6%	5%			
RAP1B-NM_001010942.3eU1307-3'UTR+581	2	2%	0%	2%	0%	0			0			400	150	550	390	190	TTAAAAAACCTGTGGAGACGGTGATCTTGTCTTTAA		2%	2%							
RAPGEF6-NM_001164386.2eU5581-3'UTR+552	2	13%	7%	18%		0			0			290	210	840	180	280	TCTGGTCTGACCAAACAGAAGTCATCTTTACAGAGG		18%		9%						
RASSF8-NM_001164746.2eU3165-3'UTR+1364	2	19%	4%	21%		0			0			440	80	690	300	240	GTAGTTTTAATTCTGAAGAAGTTACATCCTCTGTAT	21%			16%						
RBL1-NM_001323281.2eU1923-NP_001310210.1aa167PL	2	4%	1%	4%	1%	0			0			300	270	820	280	350	AAGTTCCTACCTGTGAAGAAGTTATATTCCCAAATA		4%	5%							
RBM39-NM_001242599.2eU1876-3'UTR+16	4	3%	1%	3%	0%	0			0			320	220	660	300	390	CCAAGTAGACGATGAAGGAAGATATAGTCCCTTATG	3%	4%	4%	1%						
RBM39-NM_001242599.2eU2397-3'UTR+537	4	49%	16%	57%	4%	3	9%	1%	3	23%	4%	300	270	800	180	170	TCAAAATTATATTTCTTGATGGTATCTTTTCAATTA	53%	61%	56%	26%	8%	10%	8%	28%	22%	21%
RBMXL1-NM_001162536.3eU3871-3'UTR+2313	3	11%	1%	11%	1%	0			0			290	300	800	160	230	GGTCCAGAGGGAGAAAAGAAGGCATGATTTCAGAAT	11%	10%	11%							
RBSN-NM_001302378.2eU770-NP_001289307.1aa104SS	2	13%	3%	15%		0			0			240	330	790	320	270	GGAGCTTGGTGCTGTGAGAAGCCATCTTTCCGACTT	15%			11%						
RCC2-NM_001136204.3eU1479-NP_001129676.1aa486SL	4	31%	10%	36%	3%	3	5%	1%	3	21%	1%	280	220	860	290	270	AGGAGGTAAAGACTCTGGATGGCATTTTCTCAGAGC	39%	32%	36%	17%	5%	6%	3%	20%	21%	22%
RCE1-NM_001032279.2eU352-NP_001027450.1aa10PL	2	6%	1%	6%	1%	0			0			250	290	820	480	310	TGATGGGCTTCAGGCTGGAGGGCATTTTCCCAGCGG	5%	6%								
RCE1-NM_005133.3eU356-NP_005124.1aa114PL	2	5%	2%	5%	2%	0			0			250	290	820	480	310	TGATGGGCTTCAGGCTGGAGGGCATTTTCCCAGCGG	4%	7%								
RCOR1-NM_015156.4eU4248-3'UTR+2552	4	28%	8%	31%	4%	0			0			260	160	770	220	340	GTGTGATAACTGCTTCAGATGGAATGTTCACTTAAG	35%	27%	32%	17%						
RCOR1-NM_015156.4eU4690-3'UTR+2994	4	19%	6%	20%	7%	0			1	8%		370	110	740	370	320	TGTACAGTGTTTGTGGAGACGCCACTTCCTCAAAAT	16%	28%	15%	16%						8%
RDX-NM_001260494.2eU3112-3'UTR+1519	4	9%	4%	11%	2%	0			0			280	280	760	360	230	TTCGGTTATAGCTTACTGAAGAAATCTTTCCAGTTA	12%	9%	11%	3%						
RELCH-NM_001346229.2eU4323-3'UTR+319	2	12%	0%	12%	0%	0			0			390	250	780	220	210	GTTTTTTTTAATTTTTGGATGGGATATTCGCAAATA	12%	12%								
REV3L-NM_001286431.2eU7848-NP_001273360.1aa2309HY	3	5%	1%	6%	0%	0			0			340	360	810	300	260	ACCTATGCTGCTGATGAGAAGGCACTTTTTCATGAA		5%	6%	4%						
RFX3-NM_001282116.2eU4129-3'UTR+1616	3	49%	19%	55%	23%	0			0			380	260	670	390	190	TAAGCAGGTACTTATCGAAGTGCATCTTTTCAGTCT		71%	38%	38%						
RILPL1-NM_001319243.2eU671-NP_001306172.1aa145SL	2	17%	9%	17%	9%	0			0			280	210	760	330	240	CCAACCTCTCCCACAAGGATGTCAATTTCTCAGAGG	11%	23%								

RILPL2-NM_145058.3eU951-3'UTR+51	3	8%	2%	8%	2%	0			0			220	460	710	220	270	C T A T G G C T C T G A T T C T A G A A G A C A A C C T T C C A A G A T	11%	7%	8%							
RIN3-NM_001319987.2eU1989-NP_001306916.1aa622SL	2	7%	0%	7%	0%	0			0			170	80	780	240	210	T C C T G A A G C C C C T G A A G G A A G C C A T C A A C T C A T G C C		7%	7%							
RIPK2-NM_001375360.1eU2021-3'UTR+307	4	53%	20%	62%	9%	0			0			170	210	820	330	360	A T G A T G T A T T T C T G A T G G A A G C C A T T T T C A C A T T C A	71%	54%	60%	25%						
RNF11-NM_014372.5eU2172-3'UTR+1223	4	7%	2%	8%	3%	0			0			330	360	680	540	250	G T T G T G A T C T C A A A A A A G A A G G A A T T T C T C C T T T G T	7%	11%	6%	5%						
RNF141-NM_016422.4eU110-5'UTR-5	2	12%	5%	12%	5%	0			0			340	250	760	340	230	C A A C A C T C T G G A T T T T G A A A G A C A T C T T T T C A T C A T	16%	9%								
RNF24-NM_001134337.3eU1224-3'UTR+648	4	18%	14%	21%	15%	0			0			370	140	790	320	310	T T G G A A A G G C C T A G T C A G A A G C C A T T T C C T C T T A T C	38%	19%	7%	10%						
RNF6-NM_005977.4eU2488-3'UTR+17	4	7%	5%	9%	5%	0			0			370	180	810	140	250	C A A A C A A T G G G T A A G G T G A T G G G A T C T A C T C A A A T A	15%	5%	6%	4%						
RNF6-NM_005977.4eU2910-3'UTR+439	4	22%	10%	26%	7%	3	76%	3%	0			380	260	710	260	210	T A A C T T T T T A T T T T A A G G A C A G T A T C T T T T C A T G A A	34%	24%	20%	11%	73%	79%	77%			
RNGTT-NM_001286426.2eU732-NP_001273355.1aa175RC	3	7%	2%	7%	2%	0			0			340	360	680	380	100	T A C A A G G G T G A T T A T T T G A A G G A A C T T T T T C G T C G G	5%	7%	9%							
RPAP2-NM_024813.3eU1256-NP_079089.2aa414SL	4	12%	5%	14%	2%	0			0			340	70	790	120	370	A A G A A G A A C T T G A T G A A G A T G A C A T A A T C T C A G A T C	15%	12%	16%	4%						
RPL14-NM_001034996.3eU674-NP_001030168.1aa181Q*	4	2%	0%	2%	0%	0			3	2%	1%	260	240	710	350	240	A A A A A G G C T C C A G C C C A G A A G G T T C C T G C C C A G A A A	2%	2%	2%	1%				3%	1%	2%
RPL8-NM_000973.5eU515-NP_000964.1aa160SL	4	5%	2%	6%	1%	3	0%	0%	3	2%	0%	360	40	690	500	350	T G C C C T C C G G C T C C A A G A A G G T T A T C T C C T C A G C C A	7%	6%	6%	3%	0%	0%	0%	2%	2%	2%
RPLP1-NM_001003.3eU1-5'UTR-132	2	4%	5%	0%		2	0%	0%	1	25%		250	310	310	170	120	C A C A T A A G A G G C T G C G T A T A G G C G C G A G A G C C C C T T	0%			8%		0%	0%	25%		
RPRD1A-NM_001303411.2eU3450-3'UTR+2297	4	22%	7%	25%	5%	0			0			300	210	880	180	190	C A A T A C A G T C T G A T T T A G A A G T C A T C T T T T C A G C C G	29%	25%	20%	13%						
RPS11-NM_001015.5eU547-3'UTR+47	4	8%	3%	10%	0%	1	2%		0			230	250	740	330	410	G C T C C C C A C A A T G A A A T A A A G T T A T T T T C T C A T T C C	10%	9%	10%	3%			2%			
RPS21-NM_001024.4eU291-NP_001015.1aa80SL	4	12%	4%	14%	2%	0			3	1%	0%	220	210	840	80	210	T C C G A T T G G C C A A G G C C G A T G G C A T C G T C T C A A A G A	12%	16%	13%	6%				1%	1%	1%
RPS21-XM_024451959.1eU1513-3'UTR+360	4	18%	3%	19%	1%	0			3	2%	0%	220	210	840	80	210	T C C G A T T G G C C A A G G C C G A T G G C A T C G T C T C A A A G T	19%	20%	20%	13%				3%	2%	2%
RPS21-XM_024451960.1eU1047-XP_024307728.1aa325SL	2	27%	3%	27%	3%	0			0			220	210	840	80	210	T C C G A T T G G C C A A G G C C G A T G G C A T C G T C T C A A A G A	29%	25%								
RRAGA-NM_006570.5eU1348-3'UTR+173	4	11%	6%	14%	1%	0			0			230	60	720	330	290	C C A G T C T T T A A A C A T T G G A C G C T A T T T A C T C A G C T A	14%	13%	14%	3%						
RTF1-NM_015138.5eU1403-NP_055953.3aa461SF	3	7%	3%	7%	4%	0			0			360	190	810	220	330	A G T T T A T G A A G T G G A A A G A A G C G A T G T T C T C T G C T G	11%		4%	5%						
S100PBP-NM_001256121.2eU2612-3'UTR+1108	2	7%	1%	7%	1%	0			0			210	70	700	210	200	G G T G T C T T T A G G C C C T G G A T G T T A C C A T C T C A T T T G	6%	7%								
SCARB2-NM_001204255.2eU925-NP_001191184.1aa215PL	2	3%	0%	3%	0%	0			0			160	340	770	180	360	G G T T T G T T T C T G C C A T A G A A G G C A T G C A C C C A A A T C		3%	3%							
SCCPDH-NM_016002.3eU941-NP_057086.2aa309LL	4	50%	15%	57%	3%	3	6%	1%	3	14%	2%	280	320	890	220	140	T G T G A G G T T T G G A A T T G G A A G G C A A C T T C T C A T A A A	59%	60%	53%	27%	7%	5%	7%	13%	16%	13%
SCD-NM_005063.5eU2775-3'UTR+1423	4	8%	3%	10%	2%	0			3	9%	0%	440	300	740	410	150	G A A A T G A G T A A A A A C T A G A A G G C T T C T C T C C A C A G T	11%	10%	8%	4%				8%	9%	9%
SCHIP1-NM_001197107.2eU2324-3'UTR+322	2	2%	0%	2%	0%	3	13%	1%	0			250	370	610	430	450	T A G A G A T T T T A A G T T G G A A C A G T A T T T T C C C A T T G A		2%	2%		13%	14%	12%			
SCO1-NM_004589.4eU988-3'UTR+56	2	2%	0%	2%	0%	0			0			480	170	520	530	480	A T T C T C T T G C T A A G A G G A A G G A A A C T G T C T C G C A T A	3%		2%							
SCRN1-NM_001145513.1eU2049-3'UTR+627	3	3%	1%	3%	0%	0			3	38%	7%	210	220	790	320	350	C A T C C T G G G C T C C T G A A G A A G C T G T C T T C C C A T T T T		3%	4%	2%				37%	46%	31%
SCRN1-NM_001145513.1eU572-NP_001138985.1aa132SF	4	5%	3%	6%	2%	0			0			170	230	740	150	350	C A G C T A A A G A A G C C T T A G A T G T C A T T G T C T C C T T G T	4%	7%	8%	2%						
SDF4-NM_016176.6eU586-NP_057260.3aa101SF	4	5%	2%	6%	1%	0			2	1%	0%	390	320	590	390	190	G G A G C C G G A G G A A G C T G A T G G T C A T C T T T T C C A A G G	7%	6%	6%	2%				1%		1%
SDHC-NM_003001.5eU217-NP_002992.1aa64PP	3	2%	0%	2%	0%	0			0			130	270	710	200	440	C C A C A T T A C T A T C T A C A G T T G G T C T C T T C C C A T G G C	2%	2%	2%							
SEC14L1-NM_001143998.2eU3568-3'UTR+1146	4	54%	21%	64%	4%	0			3	21%	5%	220	240	870	260	300	A A C T T A G T T T G A G T A A T G A A G G A A T C T T C A C A G A A G	60%	68%	65%	24%				16%	25%	21%
SEC22B-NM_004892.6eU378-NP_004883.3aa80PL	3	3%	1%	3%	1%	0			3	50%	5%	340	270	640	330	280	G T T A T T T G G T T T T A T G T G A A G C T G C C T T C C C T A A G A	3%	4%	2%					45%	52%	53%
SEC23B-NM_001172745.3eU1217-NP_001166216.1aa282PL	3	2%	1%	2%	1%	0			0			410	400	690	570	140	T T G C T G T T G G C T T G C T G G A G G G C A C T T T T C C A A A C A	2%		1%	1%						
SEC24D-NM_001318066.2eU2064-NP_001304995.1aa613Q*	3	8%	4%	8%	4%	0			0			320	340	690	400	440	G T T A A T A C A G A C A A A G A G A A G A T A C T T T T C C A G C C C	5%	12%	7%							
SEC63-NM_007214.5eU3033-3'UTR+551	4	11%	3%	12%	2%	0			0			280	270	750	150	420	A C T T A T C T T G G T C A T A A G T T G G C A T T C T C T C A C A T T	10%	11%	14%	7%						
SEL1L3-NM_001297592.2eU2384-NP_001284521.1aa777PL	3	18%	2%	18%	2%	0			2	8%	3%	210	230	820	320	210	T T G G A G T C C T G C A T T T G G A T G G C A T C T T C C C T G G A G	20%	19%	16%					6%	10%	
SEMA4D-NM_001371194.2eU4086-3'UTR+993	2	7%	1%	7%	1%	0			0			210	210	810	270	310	G A A C C T C C T G T G A G A C A G A T G G A A C C T T C A C A A G G G		6%	8%							
SEPTIN10-NM_001321496.2eU1225-3'UTR+35	2	6%	4%	6%	4%	0			0			190	160	680	370	340	G A A G T T C C A G A G C A C A G A A G G T C A T C A T C A C A A G C A	9%		3%							
SEPTIN7P2-NR_024271.1eU1177-ncr	3	12%	7%	12%	7%	0			0			220	220	690	380	390	G G C A G C T C A C T A A T A G A G A C G G T A T T T C A C C A T G T T	5%	18%	12%							
SERINC1-NM_020755.4eU516-NP_065806.1aa149TI	4	3%	1%	3%	1%	0			0			280	410	730	380	230	G G G C A T T C T T C A T T C C A G A A G G A A C T T T T A C A A C T G	4%	3%	2%	2%						
SERPINH1-NM_001207014.3eU1728-3'UTR+326	4	3%	1%	3%	0%	2	1%	0%	0			330	240	690	270	200	A G T A T T C A T A T T T A T A G C C A G G T A C C T T C T C A C C T G	3%	3%	3%	2%		0%	1%			
SETD2-NM_001349370.3eU4843-NP_001336299.1aa1513TI	4	8%	4%	10%	3%	0			0			240	200	750	160	330	A A C A G C A T G C A G A T G T G G A A G T C A T A C T C A C A G A A A	7%	12%	11%	3%						
SF3B1-NM_012433.4eU1255-NP_036565.2aa409PL	4	29%	9%	33%	4%	0			3	5%	1%	220	140	780	280	350	T T T C T G A T G A G G A A T T A G A T G C T A T G T T C C C A G A A G	36%	34%	29%	17%				5%	5%	6%
SGMS2-NM_001136257.2eU1266-NP_001129729.1aa305Q*	2	8%	3%	8%	3%	0			0			220	280	790	140	370	G C C A A T G A A A A A G A A C T T G A A G G T C T C T T C A C A G A C T		7%	10%							
SGTA-NM_003021.4eU1149-3'UTR+143	3	3%	1%	3%	1%	0			0			220	630	560	310	480	C T C G G A C C T G C A T G T C A A G A T G G A T T T T C C C C T T T T	3%	2%	3%							
SH3BP5-NM_001018009.4eU2283-3'UTR+827	4	19%	6%	22%	2%	0			0			270	210	710	250	340	T G T T A T T A G A T T G A C A A G A T G T T A A T T T C T C T G T A G	24%	24%	20%	10%						
SH3KBP1-NM_001024666.3eU2576-3'UTR+600	4	24%	9%	28%	3%	0			0			370	270	710	220	220	A A T T T T A G C T T A T A T A T G A C G G T A T A T T T A C A A A A A	24%	30%	30%	10%						
SH3RF2-NM_152550.4eU1518-NP_689763.4aa432PL	4	8%	2%	8%	3%	0			0			180	280	830	200	370	C C T T G G T C A C C G G G C G A G T C G G C A T C T T C C C A A A C A	11%	9%	6%	8%						
SH3RF2-NM_152550.4eU951-NP_689763.4aa243PL	2	6%	3%	6%	3%	0			0			170	360	820	240	330	G C A A G T T A G G A G A T A A A G T A G G C A T C T T C C C T A T C T		4%	8%							
SHMT1-NM_001281786.2eU2237-3'UTR+703	3	5%	2%	5%	2%	0			0			260	230	730	370	260	C C G T A A T C A G G A A G C C A A A T G T C A C C T T C C C A A A G A	4%	5%	7%							
SIK2-NM_015191.3eU4921-3'UTR+2025	4	18%	7%	21%	6%	0			3	72%	10%	300	330	740	430	390	A T T T G T T T A G A C T G A A G G A A G A C G T T T T C C C A A A A T	18%	27%	16%	11%				61%	75%	81%
SKP2-NM_001243120.2eU1208-3'UTR+28	3	7%	4%	7%	4%	0			3	48%	2%	430	150	610	370	130	T G A A G T A T T T A T T G C A G G A T G G T G T C T C T T C T T T A G	11%	6%	4%					48%	47%	51%
SLC19A1-NM_001205206.4eU1293-NP_001192135.1aa391LL	3	24%	15%	32%	6%	3	43%	10%	0			270	160	810	100	290	C C C A T C G C C A C C T T T C A G A T T G C A T C T T C T C T G T C T		36%	28%	8%	32%	52%	45%			
SLC19A1-NM_001205207.3eU1201-NP_001192136.1aa351LL	4	20%	10%	24%	7%	3	38%	7%	0			270	160	810	100	290	C C C A T C G C C A C C T T T C A G A T T G C A T C T T C T C T G T C T	21%	18%	32%	7%	32%	45%	36%			
SLC25A17-NM_001282726.2eU1150-3'UTR+75	4	4%	1%	4%	2%	0			0			240	370	700	250	540	T T C C T C C T G A G T G A A G A G A A G T G A T T C T C C C T T G A C	6%	4%	3%	4%						
SLC35A5-NM_001348905.2eU542-NP_001335834.1aa127SL	3	16%	5%	16%	5%	0			0			240	120	750	180	310	A T C T T C A A C C A G C C A T G G C T G T T A T C T T C T C A A A T T	21%	16%	10%							
SLC35B2-NM_001286509.2eU1521-3'UTR+108	3	3%	1%	3%	1%	0			3	37%	5%	230	300	700	210	370	G G C T G A A A G G G C A A A A T G C A G G T G T T T T C T C A G T A T	4%		3%	2%				39%	32%	41%
SLC35C1-NM_001145265.2eU2428-3'UTR+1229	3	17%	5%	17%	5%	0			3	54%	12%	410	160	720	190	320	C T G T T T C C C G A T C C A T A G A T G G T G C C T T C T C G C T G T	22%	13%	17%					44%	67%	50%
SLC35D2-NM_007001.3eU625-NP_008932.2aa183TI	4	8%	2%	8%	3%	0			0			210	110	710	300	440	A T A T T T T T G T A T T C C T G A A T G A T A T C T T C A C A G C A G	5%	9%	10%	7%						
SLC38A10-NM_001037984.3eU1318-NP_001033073.1aa311AV	2	5%	0%	5%	0%	0			0			280	220	780	270	160	G T G A G C A G C A G C A A A A A G A T G G C A C C T T T G C A G C A G		5%	6%							
SLC38A2-NM_001307936.2eU1413-NP_001294865.1aa293PL	4	8%	4%	10%	2%	0			1	1%		130	330	740	270	410	T G A C C C T G A C A G T A C C A G T A G T T A T T T T C C C A A T C C	11%	10%	8%	2%						1%
SLC6A6-NM_001134367.3eU1889-NP_001127839.2aa624SL	3	21%	5%	21%	5%	0			0			290	150	610	60	460	C A G T T C T C T G T G T T G G A T G T T T C A T C T T C T C G C T C G	26%	19%	17%							
SLC8A3-NM_001130417.3eU2557-3'UTR+1322	4	8%	1%	8%	2%	0			0			240	340	710	430	230	T T G T T C T T A A A C T T C A T G A A G C T A T T T T T C C A G C C T	6%	9%	7%	8%						
SLC8A3-NM_033262.5eU4735-3'UTR+1322	2	12%	0%	12%	0%	0			0			240	340	710	430	230	T T G T T C T T A A A C T T C A T G A A G C T A T T T T T C C A G C C T		13%	12%							
SLC9A6-NM_001042537.2eU2411-3'UTR+237	4	23%	12%	28%	11%	0			1	8%		210	210	720	380	310	G A C T G G A T C T G T T T T A G G A A G T T A C T T T C A C A G T G A	39%	27%	17%	11%						8%
SLC9A6-NM_001042537.2eU2476-3'UTR+302	4	36%	15%	42%	7%	0			0			320	420	560	250	440	A G T T A A A G T G T A A A A A G T G A C G G A T T T T C T C T T T C T	38%	51%	38%	15%						
SLFN11-NM_001104587.2eU756-NP_001098057.1aa104YY	2	3%	1%	3%	1%	0			3	57%	10%	280	360	670	370	320	T G A G A C C A A G C A A C A A G G A A G G T G T T T T T A C A T T T T		2%	3%					51%	69%	52%
SLFN11-NM_001104587.2eU943-NP_001098057.1aa167HY	4	5%	1%	5%	2%	0			0			620	500	570	230	330	A A G C C A A A A A T C T T G G A A G A A G G A C C T T T T C A C A A A	6%	3%	5%	5%						
SMAD5-NM_001001419.3eU6012-3'UTR+4168	2	7%	1%	8%		0			0			390	290	720	320	170	A A T C G A A T G T G A G A A T T G A A G G C A T T T C T T C T G C A T			8%	7%						
SMAP1-NM_001044305.3eU1306-NP_001037770.1aa389Q*	2	2%	0%	2%	0%	0			0			370	60	700	370	240	A A T G C A C A A A C T G G T G T G A T G C C A C T T C C T C A G A A C		2%	2%							
SMARCB1-NM_001007468.3eU671-NP_001007469.1aa156PL	4	3%	2%	3%	1%	1	2%		0			470	410	550	620	260	G C A T G G G C C G A G A C A A G A A G A G A A C C T T C C C C C T T T	5%	3%	3%	1%		2%				
SMC6-NM_001142286.2eU3395-NP_001135758.1aa1040Q*	2	3%	1%	4%		0			0			250	230	800	280	220	A T G G A C T T G A T A C T G A A G A T G G C A G A T T C C C A G C G T	4%			2%						
SMNDC1-NM_005871.4eU1772-3'UTR+882	3	3%	1%	3%	1%	0			0			250	280	630	310	340	T T C T T G T G C A T T A A T C T G A C G A T A A T T T C C C T G T A T	3%	2%	3%							
SNAPC5-NM_001329614.2eU337-NP_001316543.1aa14HY	3	13%	5%	12%	7%	0			3	30%	11%	420	70	790	180	430	T C T A G A A G A G G G G A T G A G A T G C T G T C T T C T C A C A C T		17%	8%	14%				21%	42%	28%
SNAPC5-NM_001329615.2eU185-NP_001316544.1aa52HY	4	10%	3%	11%	4%	0			3	34%	3%	420	70	790	180	430	T C T A G A A G A G G G G A T G A G A T G C T G T C T T C T C A C A C T	13%	7%	13%	7%				32%	37%	34%
SNHG29-NR_027158.1eU242-ncr	4	11%	6%	13%	3%	0			1	3%		380	140	740	370	310	A G C C A G G A G C C T G C C A T G A G G T T A T A T T C C C A G A G G	15%	10%	15%	3%				3%		
SNHG29-NR_027159.1eU565-ncr	4	24%	9%	28%	1%	0			3	6%	1%	380	180	660	530	350	G C A T C T C A G C C C A A G A G A A G G T T A T A T T C C C A G A G G	29%	27%	29%	11%				8%	6%	6%
SNHG29-NR_027160.1eU688-ncr	4	23%	7%	27%	2%	0			2	7%	2%	380	180	660	530	350	G C A T C T C A G C C C A A G A G A A G G T T A T A T T C C C A G A G G	24%	28%	27%	13%				8%	5%	
SNHG29-NR_027162.1eU741-ncr	3	23%	8%	23%	8%	0			0			380	180	660	530	350	G C A T C T C A G C C C A A G A G A A G G T T A T A T T C C C A G A G G	24%	30%	14%							
SNHG29-NR_027166.1eU299-ncr	4	36%	16%	43%	8%	0			0			380	180	660	530	350	G C A T C T C A G C C C A A G A G A A G G T T A T A T T C C C A G A G G	34%	50%	44%	14%						
SNHG29-NR_027170.1eU343-ncr	4	34%	17%	42%	8%	0			1	11%		380	180	660	530	350	G C A T C T C A G C C C A A G A G A A G G T T A T A T T C C C A G A G G	34%	50%	43%	10%				11%		

SOCS5-NM_014011.5eU4642-3'UTR+2844	3	67%	29%	67%	29%	0			0			220	250	750	280	360	TTGTTAATATTACTTTAGATGGCATTTCCACCTGAT	100%	50%	50%							
SORT1-NM_001205228.2eU2624-3'UTR+328	4	8%	2%	9%	2%	0			0			530	340	780	230	40	GAAACGAAATGGAATTCGAAGGGACCTTTTCACTAA	11%	8%	8%	6%						
SP100-NM_001080391.2eU1133-NP_001073860.1aa346SL	3	2%	0%	2%	0%	0			2	2%	0%	170	150	800	140	290	ACGTTGATGAGCCCTTAGAAGTCTTCATCTCAGCAC	1%	2%	2%					2%		2%
SP100-NM_003113.4eU3042-3'UTR+306	3	5%	3%	5%	3%	0			3	48%	3%	480	230	470	540	370	ATTAGTTGTATATGAGGATGGTAGTTTTTTCACCTT	8%	3%	4%					50%	48%	45%
SP110-NM_001185015.2eU834-NP_001171944.1aa223LL	2	1%	0%	1%	0%	0			3	17%	3%	420	80	420	300	430	AGAAGACTCAGAAGAGATGCCCAGCCTCCTCACTAG	1%		1%					14%	20%	17%
SPARC-NM_001309443.2eU3038-3'UTR+2065	4	31%	11%	36%	5%	3	1%	0%	3	2%	1%	360	130	710	380	310	ACATACCTAGATCTCCAGATGTCATTTCCCCTCTCT	41%	31%	36%	16%	1%	2%	2%	1%	2%	3%
SPART-NM_001142294.2eU1697-NP_001135766.1aa535LL	4	7%	3%	8%	2%	0			0			400	80	600	340	580	CTTAAAAAAGACAAAGATGGGAAATCTCCTCTGGAT	6%	9%	10%	3%						
SPART-NM_001142294.2eU2310-3'UTR+215	4	23%	11%	28%	5%	0			1	5%		310	200	820	140	350	TCTGGCCTTAGAAATGTGAAGTTATATTCTCAAGTT	34%	26%	25%	7%					5%	
SPCS1-NM_014041.5eU366-NP_054760.4aa75LL	4	14%	5%	16%	3%	0			1	2%		130	260	760	40	320	TCCATGGCCCATCTATCGCCGGCATCCTCTCAAGTG	16%	19%	14%	7%						2%
SPDYE2-XM_017012223.1eU4800-3'UTR+3107	2	43%	24%	60%		2	45%	46%	2	27%	7%	160	370	500	230	520	TGGGTGTCCTCAGACCACATGTACTTCTCACCAACT	60%			26%	13%		78%	22%		32%
SPG21-NM_001127889.5eU859-NP_001121361.1aa257PL	4	3%	2%	4%	1%	0			0			180	530	760	270	410	GAGCTCATCTGAAAACAGGAGGCAATTTCCCATACC	3%	5%	3%	1%						
SPIN1-NM_006717.3eU3875-3'UTR+2833	4	7%	3%	8%	2%	0			3	78%	1%	280	510	650	350	280	CAAATGTAGTGCCAACAGAAGGTGATTTTCCAGTTG	10%	9%	6%	3%				77%	79%	79%
SPIN1-NM_006717.3eU826-NP_006708.2aa191RR	4	4%	3%	4%	3%	0			2	2%	0%	380	150	650	520	260	CTTAGATGATTACAAAGAAGGCGACCTTCGCATTAT	8%	3%	2%	2%					2%	2%
SPIN4-NM_001012968.3eU1750-3'UTR+487	2	8%	3%	8%	3%	0			0			480	60	690	250	290	TTCTCACTTGACATGTAGATGATATCTCTTCTTATC	10%	5%								
SRGN-NM_001321053.2eU451-NP_001307982.1aa88PL	2	1%	0%	1%	0%	2	3%	1%	0			270	270	660	360	350	CGAGAATCCAGGACTTGAATCGTATCTTCCCACTTT	1%		1%		3%		4%			
SRP14-NM_001309434.1eU640-3'UTR+103	2	1%	0%	1%	0%	0			3	37%	3%	220	300	700	170	500	TTTCTGGTTCCCCCACAGTAGGTGTTTTCACATAAG		1%	1%					34%	37%	39%
SRRM1-NM_001303448.2eU587-NP_001290377.1aa188PS	4	9%	3%	10%	2%	0			3	3%	1%	420	360	570	530	290	AGATCTCCTTCCCCTAGAAGACGATCTTCCCCTGTC	10%	12%	8%	4%				2%	2%	4%
SRR-NM_001304803.1eU689-NP_001291732.1aa112TI	4	14%	6%	17%	4%	0			0			250	70	740	220	400	TTATCAGGGACCTTGTGGATGATATCTTCACTGTCA	15%	21%	14%	6%						
SSNA1-NM_003731.3eU512-3'UTR+101	3	2%	0%	2%	0%	0			0			360	220	680	290	280	CTTTGTTCTTCACGGCAGCAGCTACCTTCCCTCACT	2%	2%	2%							
STAM2-NM_005843.6eU4692-3'UTR+2993	4	44%	17%	41%	20%	0			0			380	360	680	380	160	ATGCTTTATTTCTATCGGAAGGTACTTTTTCTTCTT	57%	47%	19%	53%						
STARD3NL-NM_001363340.2eU242-5'UTR-50	3	5%	1%	5%	1%	0			3	43%	17%	310	240	700	260	270	AGTTTGGATAAGTTTAGCAAGGTGTCTTCTCTTTAG	5%	4%		4%				33%	32%	62%
STAT1-NM_001384880.1eU390-NP_001371809.1aa27PP	3	1%	0%	1%	0%	0			3	19%	3%	330	280	410	600	390	GGTTCACCAGCTTTATGATGACAGTTTTCCCATGGA	1%	1%	1%					16%	18%	21%
STIL-NM_001048166.1eU228-NP_001041631.1aa25HY	4	42%	11%	46%	8%	0			3	13%	8%	380	290	700	450	100	AGGTTTCCTTCAAGCAGGATGGTACCTTTCCACTTT	51%	38%	50%	28%				4%	17%	17%
STIL-XM_011541991.2eU968-XP_011540293.1aa25HY	3	40%	14%	46%	14%	0			1	57%		380	290	700	450	100	AGGTTTCCTTCAAGCAGGATGGTACCTTTCCACTTT		56%	36%	29%				57%		
STMN1-NM_001145454.3eU231-NP_001138926.1aa46SS	4	2%	1%	2%	1%	0			0			370	670	540	280	440	CCTTTCCCCTCCAAAGAAGAAGGATCTTTCCCTGGA	3%	3%	1%	1%						
STMN1-NM_001145454.3eU232-NP_001138926.1aa47LL	4	5%	2%	6%	1%	0			3	4%	0%	250	370	670	540	280	CTTTCCCCTCCAAAGAAGAAGGATCTTTCCCTGGAG	7%	5%	5%	3%				3%	4%	3%
STMN1-NM_005563.4eU231-NP_005554.1aa46SS	2	2%	0%	2%	0%	0			0			370	670	540	280	440	CCTTTCCCCTCCAAAGAAGAAGGATCTTTCCCTGGA	2%		2%							
STMN1-NM_005563.4eU232-NP_005554.1aa47LL	3	6%	0%	6%	0%	0			2	6%	0%	250	370	670	540	280	CTTTCCCCTCCAAAGAAGAAGGATCTTTCCCTGGAG	7%	6%	6%						5%	6%
STRIP2-NM_001134336.2eU1088-NP_001127808.1aa349LL	2	3%	0%	3%	0%	0			0			380	250	830	210	100	AGGCCGCCGTGGCTCTCGAAGGCAACTCCTCACTAA		4%	3%							
STXBP4-NM_178509.6eU2700-3'UTR+831	3	33%	23%	42%	26%	0			0			390	230	750	300	80	CATTCCACAGAGATTCAGAAGGCACCTCTTCGGTCA		24%	60%	17%						
STXBP6-NM_001304476.3eU1485-3'UTR+434	3	13%	2%	13%	2%	0			0			280	310	660	350	310	TTAATGTGGTGACATCAGATGTTATTTTTCCTAGAT	15%	11%	14%							
SUGP2-NM_001017392.5eU3766-3'UTR+425	4	16%	10%	18%	11%	0			3	64%	3%	270	220	730	210	450	ATGTGGAAACGATCACAGATGGTGTTTTCTCGTTGT	30%	17%	8%	11%				67%	61%	65%
SUPT5H-NM_001111020.3eU172-NP_001104490.1aa10SF	3	2%	0%	1%	0%	3	19%	3%	0			430	440	640	260	40	AGATGTCGGACAGCGAGGACAGCAACTTTTCCGAGG		1%	1%	2%	15%	21%	19%			
SUPT5H-NM_003169.4eU276-NP_003160.2aa10SF	2	2%	1%	3%		3	19%	3%	0			430	440	640	260	40	AGATGTCGGACAGCGAGGACAGCAACTTTTCCGAGG	3%			2%	16%	22%	21%			
SYNJ1-NM_001160302.1eU2289-NP_001153774.1aa722HY	2	9%	4%	9%	4%	0			0			170	200	740	470	370	AGTTTTCCTATGGGAAGGATGCTATTTTCCCATGAC	12%	6%								
SYNJ1-NM_001160302.1eU6402-3'UTR+2389	4	28%	14%	35%	7%	2	6%	2%	3	16%	3%	270	150	860	220	330	AAAATACTATATTTATAGAAGTAATCTTCTCTGTTA	30%	31%	43%	8%	4%	8%		13%	17%	17%
SZRD1-NM_001114600.3eU175-NP_001108072.1aa53PP	4	3%	2%	4%	2%	0			3	45%	1%	470	220	350	540	340	GCCCATTGTGATTCAGGACGATAGCCTTCCCGCGGG	5%	5%	2%	1%				45%	46%	46%
SZRD1-NM_001271869.2eU172-NP_001258798.1aa52PP	4	5%	1%	5%	1%	0			3	72%	6%	470	220	350	540	340	GCCCATTGTGATTCAGGACGATAGCCTTCCCGCGGG	6%	4%	6%	4%				66%	79%	72%
SZRD1-NR_073502.2eU125-ncr	4	4%	3%	5%	3%	0			3	47%	4%	470	220	350	540	340	GCCCATTGTGATTCAGGACGATAGCCTTCCCGCGGG	8%	5%	3%	2%				43%	47%	50%
SZRD1-NR_073503.2eU122-ncr	3	4%	2%	4%	2%	0			3	45%	5%	470	220	350	540	340	GCCCATTGTGATTCAGGACGATAGCCTTCCCGCGGG	5%	5%	2%					43%	50%	41%
TACC1-NM_001122824.2eU5950-3'UTR+4415	3	7%	2%	7%	2%	0			0			370	230	750	300	280	TAATTTAGAACTGTCAAGATGTCACTTTCTCCCCCT	9%	6%	5%							
TADA3-NM_006354.5eU233-5'UTR-54	4	9%	3%	10%	2%	0			0			300	200	630	290	290	ACTGAACTGGGCTCTTGACGGGCATCATCTCTTAAT	12%	8%	11%	4%						
TAF1B-NM_001318976.1eU1029-NP_001305905.1aa63PL	4	14%	4%	16%	3%	0			0			200	350	690	260	250	GTATGAAATACTTGATGGAAGTCAACCTCCCTGATG	12%	19%	16%	10%						
TAF5L-NM_014409.4eU2607-3'UTR+596	2	8%	3%	8%	3%	0			0			390	160	840	280	260	GAAACCTAGCACTGGCAGAGGGCACCTTCTCTGCTT		6%	10%							
TAF9-NM_001015892.2eU861-NP_001015892.1aa195Q*	3	2%	0%	1%	0%	0			0			230	150	770	240	260	CAAAGGTTTACAGTACAGATGCCTACTTCTCAGTCT	2%		1%	2%						
TAOK3-NM_001346487.2eU2283-NP_001333416.1aa610LL	2	13%	2%	13%	2%	0			0			450	110	640	440	380	CACACAGGCTGAAGAGGAAGCCCACCTTCTCACTCA	14%	11%								
TAP1-NM_000593.6eU897-NP_000584.3aa277Q*	3	1%	0%	1%	0%	0			3	16%	2%	280	400	670	430	330	GCTGTCCTGCGCCAGGAGACGGAGTTTTTCCAACAG	1%	1%	1%					17%	14%	17%
TAPT1-NM_153365.3eU2126-3'UTR+408	4	16%	11%	13%	10%	0			0			240	200	720	400	110	TGTGAAACTTTTACCACGACGTAATCTTTCCAGTCT	25%	7%	7%	26%						
TBCD-NM_005993.5eU654-NP_005984.3aa175LL	3	3%	1%	4%	2%	0			0			460	130	590	550	180	TGATTTTTCTCGCCTTGACGGGAACCTCCTCACCCA		3%	5%	2%						
TCAIM-NM_001029839.3eU168-NP_001025010.1aa19HY	4	24%	6%	26%	3%	0			0			360	140	760	280	620	AGGAGGTTATGTCTAGAGAAGATATTTCCACACTGG	28%	29%	23%	16%						
TCAIM-NM_001029840.3eU290-NP_001025011.1aa19HY	2	21%	1%	21%	1%	0			0			360	140	760	280	620	AGGAGGTTATGTCTAGAGAAGATATTTCCACACTGG		22%	21%							
TCEA1-NM_006756.4eU2026-3'UTR+797	4	7%	4%	8%	3%	0			0			310	220	770	360	340	ATCCCCTCATAGGCAAAGATGAAAACTTCCCACTAT	7%	11%	7%	2%						
TCF4-NM_001083962.2eU6350-3'UTR+4159	2	11%	2%	11%	2%	0			0			170	200	710	350	350	TATTTTACTATTTAAAAGATGTTATTTTACCATGTG		9%	13%							
TDP2-NM_016614.3eU504-NP_057698.2aa156PL	4	17%	6%	20%	2%	0			0			210	260	650	440	360	ATGTGATATTTCTACAGGAAGTTATTCCCCCATATT	22%	19%	20%	8%						
TEAD1-NM_021961.6eU2075-3'UTR+173	4	13%	5%	14%	6%	0			0			300	270	790	250	290	CTAAATCTTGTTTGAGTGAAGTCATTTTTTCATGTG	8%	19%	16%	10%						
TEAD2-NM_001256658.2eU647-NP_001243587.1aa185Q*	2	6%	2%	8%		0			0			160	260	750	190	470	TGGAATGTTCCAGATGTGAAGCCATTCTCACAGACA		8%		4%						
TEX261-NM_144582.3eU3253-3'UTR+2546	3	3%	1%	3%	1%	0			0			260	110	650	440	330	GCTCACTTGCCTGCCTAATGGTCATCTCCCCAGTAG	3%	3%	2%							
TFAM-NM_001270782.2eU151-NP_001257711.1aa5R*	4	2%	1%	2%	1%	0			3	20%	1%	380	330	560	420	250	GCCTCATCCACCGGAGCGATGGCGTTTCTCCGAAGC	3%	2%	2%	1%				19%	22%	20%
TFB2M-NM_022366.3eU710-NP_071761.1aa196PL	2	9%	5%	9%	5%	0			0			130	280	740	280	290	ACATCCCTTTAAAAGTAGTTGGAATGTTCCCAAGTA	12%		5%							
TFIP11-NM_001008697.3eU1122-NP_001008697.1aa265Q*	4	19%	5%	21%	1%	0			1	5%		270	350	840	340	280	AAGCTCACTGCTCCCCAGAAGGAACTTTCTCAAGTC	23%	21%	20%	11%				5%		
TFIP11-NM_001008697.3eU310-5'UTR-21	4	17%	12%	20%	12%	0			0			310	80	790	230	320	AAGAATCTGGGATCTAAGAAGATACCTACTCATTTT	33%	10%	16%	8%						
TFIP11-NM_001008697.3eU3209-3'UTR+366	2	6%	1%	6%	1%	0			0			500	440	610	140	460	CGAGTTCAGTCAGTAGCAGAAGGATCTTCTCTCTTG		6%	7%							
TFIP11-NM_001346861.2eU229-5'UTR-21	3	23%	8%	23%	8%	0			0			310	80	790	230	320	AAGAATCTGGGATCTAAGAAGATACCTACTCATTTT	13%	25%	29%							
TFIP11-NM_012143.4eU233-5'UTR-21	4	28%	7%	30%	6%	0			0			310	80	790	230	320	AAGAATCTGGGATCTAAGAAGATACCTACTCATTTT	37%	26%	28%	20%						
TFRC-NM_001128148.3eU2583-3'UTR+229	3	3%	1%	4%	1%	0			0			300	330	730	360	210	ACTTAAAAAATGTCCATGATGGAATATTCCCCTATC		4%	4%	2%						
TGFB2-NM_001135599.4eU2223-NP_001129071.1aa286TI	4	13%	7%	17%	2%	0			0			310	90	750	260	190	CAAGATTTGCAGGTATTGATGGCACCTCCACATATA	14%	16%	19%	4%						
TGFBI-NM_000358.3eU1345-NP_000349.1aa426PL	4	2%	1%	3%	0%	0			0			270	320	610	460	190	TGAATTCTGTATTCAAAGATGGAACCCCTCCAATTG	3%	3%	3%	2%						
TGOLN2-NM_001206844.2eU5684-3'UTR+4517	2	2%	0%	2%	0%	0			2	1%	0%	430	320	380	410	490	TTTTCCCAGTTAATAGATGGTCACTGTTTCCACAAT	2%	2%						1%	1%	
THADA-NM_001083953.2eU4435-NP_001077422.1aa1340SF	3	8%	0%	7%	0%	0			0			430	210	580	450	160	TCTACGCTTCCCCGATGGATGGTACTTCTTCTGCTC	7%		8%	8%						
THSD1-NM_018676.4eU738-NP_061146.1aa172TI	3	22%	13%	28%	11%	0			0			220	220	780	220	260	AGCCCAACATCGTAGTGGATGTCATCTTCACCAACA		21%	36%	10%						
TICAM1-NM_001385678.1eU1123-NP_001372607.1aa329SF	3	5%	1%	5%	2%	0			0			440	80	670	200	320	CACTCCAACTTTCTGTAGAAGATACCACCTCTCCAA	7%		4%	6%						
TIGAR-NM_020375.3eU2537-3'UTR+1665	3	58%	14%	58%	14%	0			2	9%	4%	340	280	800	280	150	TCCATTCCCACCATACTGAAGTCATCTTTCCACTTT	71%	43%	59%					12%	6%	
TIPRL-NM_152902.5eU880-NP_690866.1aa255PL	2	3%	1%	3%	1%	0			0			450	240	500	610	230	AGGAAGCAGTTTGTGAGAAGCTAATATTTCCAGAAA	2%		3%							
TJP2-NM_001170414.2eU1198-NP_001163885.1aa322HH	4	23%	12%	28%	5%	0			1	7%		300	160	520	560	310	GGGTCTGGCAACTAAAGATGGCAACCTTCACGAAGG	34%	28%	23%	6%						7%
TLNRD1-NM_022566.3eU2493-3'UTR+55	3	8%	5%	8%	5%	0			0			450	140	650	380	340	CCACCCCCAGACTAAAGGAAGATACTTACTCTCTGC	14%	6%	5%							
TMED8-NM_001346131.2eU3593-3'UTR+2559	2	25%	6%	25%	6%	0			0			250	210	770	250	580	GCACAAAATAGCTAAAAGAAGATATTTTCACTGTCT		29%	21%							
TMEM131L-NM_001131007.2eU3577-NP_001124479.1aa1180SF	3	8%	1%	8%	1%	0			0			430	210	780	180	390	TTCACAAAACATCTAGAGAAGACATGTTTTCTGAGA	9%	7%	8%							
TMEM260-NM_017799.4eU763-NP_060269.3aa216LL	3	14%	5%	16%	6%	0			0			190	420	680	420	390	TTTCAACTTTTAAAAAAGAAGGAACTCTCCCTGGGC		20%	12%	12%						
TMEM50A-NM_014313.4eU832-3'UTR+222	2	2%	1%	2%	1%	0			0			330	130	720	430	450	TAACTTATAAAATGTTAGAGGAAACTTTCACATGAA	3%	2%								
TMEM65-NM_194291.3eU2273-3'UTR+1006	4	54%	17%	61%	8%	3	12%	6%	3	30%	12%	230	70	910	180	330	TTTTTTTTCCCTCCAAAGATGTCATCTTCTCATCTG	61%	53%	70%	31%	6%	13%	18%	21%	43%	26%
TNC-NM_002160.4eU5468-NP_002151.2aa1719SL	4	34%	8%	38%	4%	3	3%	1%	3	13%	1%	280	210	860	330	310	CCATGGGCTCCCCAAAGGAAGTCATTTTCTCAGACA	42%	38%	35%	23%	3%	3%	4%	13%	13%	12%

TNC-XM_005251972.4eU5195-XP_005252029.1aa1628SL	3	38%	9%	38%	9%	0			0			280	210	860	330	310	CCATGGGCTCCCCAAAGGAAGTCATTTTCTCAGACA	29%	40%	46%							
TNC-XM_005251973.4eU4376-XP_005252030.1aa1355SL	3	53%	20%	53%	20%	0			1	50%		280	210	860	330	310	CCATGGGCTCCCCAAAGGAAGTCATTTTCTCAGACA	67%	30%	63%					50%		
TNC-XM_005251975.4eU3557-XP_005252032.1aa1082SL	4	36%	12%	42%	2%	0			2	25%	4%	280	210	860	330	310	CCATGGGCTCCCCAAAGGAAGTCATTTTCTCAGACA	44%	42%	41%	18%					27%	22%
TNFAIP3-NM_001270507.2eU3687-3'UTR+1039	2	3%	1%	3%	1%	0			0			390	200	690	320	390	TTCAAGATTATTTAAGTGAAGATATTTCTTCAGCTC	2%		3%							
TNFRSF9-NM_001561.6eU1280-3'UTR+372	3	26%	25%	36%	26%	0			0			220	220	730	420	250	GTTTTTTGTTTGGTAAAGATGGCATTTCACCATGTT	54%	18%		5%						
TOMM22-NM_020243.5eU189-NP_064628.1aa56PL	3	1%	1%	1%	1%	0			0			460	280	460	490	270	AGAGACTATGGGGCCTGACGGAGATGTTTCCGGAGA	1%	1%	2%							
TOMM5-NM_001001790.3eU161-NP_001001790.1aa32LL	3	3%	1%	3%	1%	0			0			270	220	720	340	280	GGATGTGATCTCCTCCATACGGAACTTTCTCATCTA	3%	2%	4%							
TOMM70-NM_014820.5eU903-NP_055635.3aa255Q*	4	12%	5%	14%	3%	0			2	2%	0%	220	0	770	250	220	AAGAATCGTGAACCTCTGATGCCATCTCCACAGTTT	12%	18%	13%	5%				2%	2%	
TOP2A-NM_001067.4eU2962-NP_001058.2aa953LF	4	13%	5%	16%	1%	0			0			550	80	780	260	340	ATGTTGAATGGCACCGAGAAGACACCTCCTCTCATA	15%	15%	17%	6%						
TOP3A-NM_000122.2_2614eU2088-NP_001307688.1aa554PL	4	10%	3%	12%	0%	0			0			210	150	810	280	390	CAGAGTTGGCCCAGCAAGAAGATATCTACCCAGCCA	12%	12%	11%	6%						
TOPORS-NM_001195622.2eU1561-NP_001182551.1aa471HY	2	3%	0%	3%		0			0			360	40	520	420	340	GATAGCGATGTTAGTAGATGCTCATCTCCACACTCT		3%		3%						
TOR1AIP1-NM_001267578.2eU711-NP_001254507.1aa165Q*	3	7%	2%	9%	1%	2	7%	5%	0			260	480	700	160	460	CATTCCTCTGAAGAGGATGAAGCATCTTCCCAAACT		9%	8%	5%	4%	10%				
TOR1AIP1-NM_015602.4eU711-NP_056417.2aa165Q*	3	12%	4%	14%	0%	1	10%		0			260	480	700	160	460	CATTCCTCTGAAGAGGATGAAGCATCTTCCCAAACT	14%	14%		7%			10%			
TP53BP2-NM_001031685.3eU1287-NP_001026855.2aa340PP	2	4%	2%	4%	2%	0			0			250	330	580	530	370	TCTACCAGTTTCATCTGATGGAAATCTCCCCAGCA		2%	5%							
TPM3-NM_001043351.2eU965-3'UTR+119	3	1%	0%	1%	0%	0			3	27%	2%	390	270	510	550	420	GGCTGATCTTTAACTGGAAGGCTGCTTTCTCCTTTC	1%	2%		1%				26%	27%	29%
TPX2-NM_012112.5eU1068-NP_036244.2aa187PS	4	3%	1%	4%	2%	3	9%	2%	0			170	460	810	240	330	CAAGATACTGCTGAAAAGAATGCATCTTCCCCAGAG	3%	2%	5%	2%	7%	9%	11%			
TRA2A-NM_001282757.2eU1245-NP_001269686.1aa162PS	4	9%	3%	10%	1%	0			1	2%		390	250	510	400	260	GATTACCGATACAGAAGACGATCACCTTCTCCTTAT	11%	11%	9%	5%						2%
TRAF3-NM_001199427.2eU1743-3'UTR+41	4	15%	8%	18%	3%	0			0			320	250	820	210	180	GGGAGGTGGATTTAGCAGAAGGCAACTCCTCTGGGG	17%	16%	22%	4%						
TRAF3-NM_001199427.2eU3299-3'UTR+1597	4	32%	12%	38%	1%	0			0			230	270	900	210	350	CAGCCTGTGCCTGCCCAGATGGCATTTTCTCAACTC	38%	37%	39%	14%						
TRAM1-NM_001317804.2eU888-NP_001304733.1aa221LL	3	1%	0%	1%	0%	3	15%	1%	0			440	380	760	170	350	AGCAATGAAAAGTATCAGAAAGGATTTTCTCTGTGG	2%	1%	1%		13%	14%	16%			
TRAPPC14-NM_018275.5eU682-NP_060745.3aa153PL	3	10%	5%	13%	3%	0			0			350	140	830	370	250	CGATTGTGTCCACAGATGAGGTCATCTTCCCACTCA	10%	15%		4%						
TRIM14-NM_014788.4eU1531-3'UTR+168	4	2%	1%	2%	1%	0			2	5%	2%	220	390	710	140	340	CACGCCTACAGTGCTTTGAAGGTTTCCTCTCCTAGG	2%	3%	3%	1%				3%	7%	
TRIM22-NM_001199573.2eU1974-3'UTR+313	4	5%	2%	6%	2%	0			0			330	340	870	140	190	TCTCTCCTTGGCTTGTAGAAGGCATCTTGTCCCTAT	4%	5%	7%	2%						
TRIM24-NM_003852.4eU2628-NP_003843.3aa780HY	2	5%	0%	5%	0%	0			0			390	140	760	300	80	TGGTTGGATCCTTCCCAGAAGTCACCTCTTCATGTT		5%	5%							
TRIP12-NM_001284214.2eU2557-NP_001271143.1aa793AV	3	5%	1%	5%	1%	0			0			240	360	830	290	290	TGCCATGTTTACCAAAAGAAGGCATTTTTGCAGTTG	5%	5%	4%							
TRIP12-NM_001284215.2eU2431-NP_001271144.1aa751AV	2	8%	0%	8%	0%	0			0			240	360	830	290	290	TGCCATGTTTACCAAAAGAAGGCATTTTTGCAGTTG		9%	8%							
TRMT6-NM_001281467.2eU514-5'UTR-16	4	5%	1%	5%	1%	0			0			160	200	690	220	320	GCCATCATTACTGTTGTGAAGCCATCCACCCGTATT	6%	5%	3%	4%						
TRNP1-NM_001013642.3eU1358-3'UTR+579	4	7%	3%	9%	1%	0			0			290	170	660	260	250	GGGTCCCCATTTTAATAGATGTCATTGCTTCAAGTC	7%	10%	9%	4%						
TRUB2-NM_001329861.2eU698-NP_001316790.1aa220TM	4	2%	1%	3%	1%	0			1	3%		270	160	740	200	340	AAGTGCGGCGCACGCGCGACGGCTTCTTCACGCTAG	3%	3%	2%	1%						3%
TSC1-NM_000368.5eU2905-NP_000359.1aa896LL	2	9%	0%	9%	0%	0			0			230	300	850	180	330	AGAGCTAGAAAAAAACAGAAGCCATGTTCTCCAGCA		9%	9%							
TSN-NM_001261401.2eU1070-3'UTR+494	3	6%	1%	6%	1%	0			0			250	170	530	400	350	TTGATGTACTGATTGAGAAGTTCATTGTCTCGTTTG	6%	5%	7%							
TSPAN15-NM_001351263.2eU1369-3'UTR+615	2	16%	2%	16%	2%	0			0			330	220	630	380	410	GTTTCTGACTAATCAAAGCTGGTATTTCCCCGCATG	14%	18%								
TTC12-NM_001318533.2eU975-NP_001305462.1aa308SS	4	12%	2%	12%	2%	0			3	60%	11%	430	430	710	390	310	TGACAACGAGGTCATAAGAAGGTGTTTTTCCACAGC	15%	11%	11%	11%				50%	71%	60%
TTC1-NM_001282500.2eU350-NP_001269429.1aa90SF	2	2%	1%	2%	1%	0			0			460	180	650	320	240	AGAACAAATCTAATGAAGATGTGAATTCCTCTGAAC		2%	1%							
TTC39B-NM_001168339.1eU2107-3'UTR+27	2	6%	1%	6%	1%	0			0			430	350	660	550	210	TTGATCAGAACTTGAAGGATGGAATTTTTCCTCAAT		5%	6%							
TTF1-NM_001205296.2eU758-NP_001192225.1aa177Q*	4	7%	4%	8%	4%	0			3	69%	5%	300	150	730	350	390	GAAGAAGTGATTCTGAAGAAGATGTCTCCCCAGGAG	12%	4%	8%	4%				74%	67%	65%
TUFM-NM_001365360.2eU1492-3'UTR+129	2	2%	0%	2%	0%	0			0			340	170	510	630	390	CAGCAGAGGGCAGCTAGATGGACATTTCCCCTGCTC	2%	2%								
TXNDC12-NM_015913.4eU751-3'UTR+137	3	3%	1%	3%	1%	0			3	51%	5%	350	290	650	290	210	CATTCCTACCTACACTGGAAGGAGCGCTCTCACTGT	3%	3%	2%					48%	49%	57%
TXNDC5-NM_001145549.4eU1836-3'UTR+535	4	7%	2%	8%	1%	0			0			250	150	720	270	330	AAAAACCCACACCTCTGGAAGATACCTTCACGGCCG	9%	8%	7%	5%						
TXNIP-NM_006472.6eU85-5'UTR-230	4	3%	1%	3%	1%	0			2	3%	0%	320	300	810	310	390	CCAGCAATTGGGGGAAAGAAGGCTTTTTCTCTGAAT	2%	4%	3%	3%				3%		3%
TXNL1-NM_004786.3eU847-NP_004777.1aa232PL	3	2%	1%	2%	1%	0			0			280	350	720	330	250	AGGATGATATTAAAGAAGATGGCATTGTTCCACTTC	2%	2%	1%							
TXNRD1-NM_001093771.3eU359-NP_001087240.1aa112SL	3	6%	3%	8%	2%	0			0			260	290	680	290	210	AGGACGGTCGGGCCCTGGAAGGAACGCTCTCGGAAT	9%		6%	2%						
TXNRD1-NM_003330.4eU346-NP_003321.3aa14SL	4	7%	3%	9%	1%	0			0			260	290	680	290	210	AGGACGGTCGGGCCCTGGAAGGAACGCTCTCGGAAT	8%	10%	8%	2%						
TXNRD1-NM_182729.3eU109-5'UTR-117	3	4%	0%	4%	0%	0			0			260	290	680	290	210	AGGACGGTCGGGCCCTGGAAGGAACGCTCTCGGAAT	4%	4%	4%							
UBA3-NM_001363861.1eU1252-NP_001350790.1aa411PL	2	2%	1%	2%	1%	0			0			350	70	640	300	100	AAGAACTGGCGGTTGCTGATGTCACCACCCCACAGA	2%	1%								
UBA6-NM_018227.6eU4922-3'UTR+1727	4	10%	4%	12%	4%	0			2	8%	4%	340	430	750	450	290	AAAAGGACTTTTGCAAAGAAGGCTTTTTTCCACGTT	15%	12%	7%	7%				5%	11%	
UBAP1-NM_001171201.1eU1586-NP_001164672.1aa526Q*	4	2%	1%	2%	1%	0			3	42%	2%	390	210	610	440	130	TGTTCAGAAGAAAAGATGATGGAGTTTCTTCAGTTA	3%	2%	2%	3%				40%	44%	42%
UBB-NM_001281716.2eU377-NP_001268645.1aa38PL	4	28%	7%	31%	1%	3	1%	0%	3	4%	0%	210	450	680	440	340	CCAAGATCCAGGATAAGGAAGGCATTCCCCCCGACC	32%	32%	30%	18%	1%	1%	1%	4%	4%	4%
UBB-NM_001281716.2eU605-NP_001268645.1aa114PL	4	21%	6%	24%	3%	3	1%	0%	3	4%	0%	280	450	700	330	260	CCAAGATCCAGGATAAAGAAGGCATCCCTCCCGACC	27%	25%	21%	13%	1%	2%	1%	4%	4%	4%
UBB-NM_001281716.2eU606-NP_001268645.1aa114PP	4	1%	1%	2%	0%	0			3	3%	0%	230	280	450	700	330	CAAGATCCAGGATAAAGAAGGCATCCCTCCCGACCA	2%	2%	1%	1%				3%	2%	3%
UBB-NM_001281716.2eU833-NP_001268645.1aa190PL	4	46%	13%	52%	3%	3	4%	0%	3	12%	1%	210	390	770	290	360	CCAAGATCCAAGATAAAGAAGGCATCCCCCCCGACC	55%	53%	49%	27%	4%	4%	4%	11%	12%	12%
UBB-NM_001281717.1eU790-NP_001268646.1aa190PL	4	51%	23%	62%	5%	0			2	16%	1%	210	390	770	290	360	CCAAGATCCAAGATAAAGAAGGCATCCCCCCCGACC	57%	67%	63%	18%					17%	16%
UBB-NM_001281718.1eU294-NP_001268647.1aa38PL	4	21%	7%	24%	3%	0			0			210	450	680	440	340	CCAAGATCCAGGATAAGGAAGGCATTCCCCCCGACC	21%	28%	24%	12%						
UBB-NM_001281719.1eU292-NP_001268648.1aa38PL	4	24%	10%	28%	8%	0			1	10%		210	450	680	440	340	CCAAGATCCAGGATAAGGAAGGCATTCCCCCCGACC	35%	30%	20%	13%					10%	
UBB-NM_001281720.2eU372-NP_001268649.1aa114PL	4	28%	7%	31%	4%	1	3%		3	6%	1%	280	450	700	330	260	CCAAGATCCAGGATAAAGAAGGCATCCCTCCCGACC	36%	29%	29%	18%	3%			5%	7%	5%
UBB-NM_001281720.2eU600-NP_001268649.1aa190PL	4	39%	11%	42%	11%	0			0			210	390	770	290	360	CCAAGATCCAAGATAAAGAAGGCATCCCCCCCGACC	37%	55%	35%	29%						
UBB-NM_018955.4eU213-NP_061828.1aa38PL	4	27%	6%	30%	0%	3	1%	0%	3	3%	0%	210	450	680	440	340	CCAAGATCCAGGATAAGGAAGGCATTCCCCCCGACC	30%	30%	31%	18%	1%	1%	1%	4%	3%	4%
UBB-NM_018955.4eU441-NP_061828.1aa114PL	4	19%	9%	22%	8%	0			0			280	450	700	330	260	CCAAGATCCAGGATAAAGAAGGCATCCCTCCCGACC	27%	27%	12%	11%						
UBB-NM_018955.4eU669-NP_061828.1aa190PL	3	56%	21%	56%	21%	0			0			210	390	770	290	360	CCAAGATCCAAGATAAAGAAGGCATCCCCCCCGACC	33%	60%	75%							
UBC-NM_021009.7eU1092-NP_066289.3aa342PL	4	5%	1%	6%	1%	0			3	1%	0%	320	350	660	410	180	CAAAGATCCAAGACAAGGAAGGCATCCCTCCTGACC	6%	6%	5%	4%				1%	1%	1%
UBC-NM_021009.7eU1320-NP_066289.3aa418PL	4	5%	1%	5%	0%	3	0%	0%	3	1%	0%	320	350	660	410	180	CAAAGATCCAAGACAAGGAAGGCATCCCTCCTGACC	6%	5%	5%	3%	0%	1%	0%	1%	1%	1%
UBC-NM_021009.7eU1548-NP_066289.3aa494PL	4	4%	1%	4%	1%	2	0%	0%	3	1%	0%	320	350	660	410	180	CAAAGATCCAAGACAAGGAAGGCATCCCTCCTGACC	5%	4%	4%	3%	0%	0%		1%	1%	1%
UBC-NM_021009.7eU1776-NP_066289.3aa570PL	4	4%	1%	4%	0%	0			3	1%	0%	320	350	660	410	180	CAAAGATCCAAGACAAGGAAGGCATCCCTCCTGACC	5%	4%	4%	2%				1%	1%	1%
UBC-NM_021009.7eU180-NP_066289.3aa38PL	4	17%	3%	19%	1%	3	1%	0%	3	3%	0%	320	350	660	410	180	CAAAGATCCAAGATAAGGAAGGCATCCCTCCTGACC	19%	19%	18%	12%	1%	1%	1%	2%	3%	3%
UBC-NM_021009.7eU2004-NP_066289.3aa646PL	4	4%	1%	4%	0%	0			3	0%	0%	320	350	660	410	180	CAAAGATCCAAGATAAGGAAGGCATCCCTCCTGATC	4%	4%	4%	2%				0%	0%	1%
UBC-NM_021009.7eU408-NP_066289.3aa114PL	4	3%	1%	3%	0%	0			0			320	410	610	480	240	CAAAGATCCAGGACAAGGAAGGCATTCCTCCTGACC	4%	3%	3%	1%						
UBC-NM_021009.7eU636-NP_066289.3aa190PL	4	3%	1%	4%	0%	0			1	0%		320	410	610	480	240	CAAAGATCCAAGATAAGGAAGGCATTCCTCCTGATC	4%	4%	3%	3%				0%		
UBC-NM_021009.7eU864-NP_066289.3aa266PL	4	5%	1%	5%	0%	0			3	0%	0%	320	410	610	480	240	CAAAGATCCAAGACAAGGAAGGCATTCCTCCTGACC	5%	5%	5%	3%				0%	0%	0%
UBE2E3-NM_001278554.2eU1115-3'UTR+46	4	10%	4%	12%	2%	0			3	5%	0%	330	240	990	140	290	TGCAGTGTGAAGGAGCAGAAGGCATCTTCTCACTGT	13%	10%	14%	5%				5%	5%	6%
UBE2K-NM_001111112.2eU2521-3'UTR+1855	2	6%	2%	6%	2%	0			3	9%	4%	290	280	570	560	190	GATTGTGTGAAAATGTGGATGGTTTTTCTCCAGTGT		5%	7%					12%	5%	9%
UBE3C-NM_014671.3eU3560-NP_055486.2aa1071LL	4	2%	1%	2%	1%	0			0			270	280	760	220	220	TGACGAGACACTTTTGCGAAGTAAACTTCTCTATGC	3%	2%	2%	1%						
UBOX5-NM_001267584.2eU131-5'UTR-12	4	34%	14%	39%	10%	1	8%		2	20%	7%	210	200	800	160	420	GCTGCTTCTGATGCTTGGAAGATATCCTCTCAGCCA	38%	50%	31%	17%	8%				25%	15%
UBXN4-NM_014607.4eU2721-3'UTR+1119	4	8%	4%	10%	2%	0			0			320	210	820	410	350	TTATTTTGTAATGGGAGGAAGAAATTTTCTCAGAGC	12%	8%	9%	2%						
UHMK1-NM_001184763.1eU3298-3'UTR+2183	3	4%	0%	4%	0%	0			0			210	110	540	430	380	ATGCTGCTAACATCTTGATGCCAATCTTCACAGCAT		4%	4%	3%						
UHMK1-NM_001184763.1eU7901-3'UTR+6786	4	18%	6%	21%	2%	2	4%	1%	2	2%	0%	280	300	780	330	450	ATGTGTGTCAGTACCAAAAAGTATTTTTCTCAAATC	23%	20%	20%	9%	3%	5%		2%	2%	
UQCRC2-NM_003366.4eU95-NP_003357.2aa11SL	2	3%	1%	3%	1%	0			1	2%		230	270	630	280	400	TGAAGCTACTAACCAGAGCCGGCTCTTTCTCGAGAT	4%	2%								2%
USP16-NM_001001992.2eU2210-NP_001001992.1aa687LF	4	3%	1%	3%	1%	0			0			270	210	710	250	340	ACCAATGCCAAAAAGCAGATGCTAATTTCTCTTGCT	3%	4%	3%	3%						
USP28-NM_001301029.2eU1028-NP_001287958.1aa265Q*	2	5%	0%	5%	0%	0			0			290	150	720	360	310	GAGAAAATTCACAATAAGCTGGAATTTCCTCAGATT	5%		5%							
USP39-NM_001256725.2eU603-NP_001243654.1aa198TI	4	2%	0%	2%	0%	0			0			340	220	550	510	370	ATATCACGTATGTGTTGAAGCCCACTTTCACAAAGC	3%	2%	2%	2%						
USP48-NM_001032730.3eU1727-3'UTR+77	4	17%	5%	20%	3%	0			0			360	260	760	380	280	TGTTCACAAATGTATATGAAGAAACTTTCTCAAACT	22%	17%	20%	10%						
USP5-NM_001098536.2eU1271-NP_001092006.1aa414PL	3	2%	1%	2%	1%	0			0			300	220	680	360	310	AACAGAAGGAAGTTCAAGATGGCATTGCCCCTCGGA	3%	1%	2%							

VAMP3-NM_004781.4eU673-3'UTR+282	2	2%	0%	2%	0%	0	0			330	70	750	220	190	C A G A G T G C T A T A A T C T A G A T G T A A T G T T G T C A C T A A	2%	2%					
VCAN-NM_001126336.3eU3296-3'UTR+1042	4	54%	5%	52%	3%	0	0			260	250	690	390	330	A C T G A T T T A A T T T A T T G G A T G T T A T T T T C C C T A A G A	50%	56%	51%	60%			
VCP-NM_001354927.2eU2170-NP_001341856.1aa568TI	4	6%	3%	7%	0%	0	0			270	140	760	290	260	A G A T C C T G A C A G A A A T G G A T G G C A T G T C C A C A A A A A	7%	7%	8%	2%			
VDAC1-NM_003374.3eU1098-3'UTR+159	4	5%	2%	6%	1%	0	0			270	250	630	310	250	A G T T G A T T C A G C T T T A A G A T G T T A C C C T T C C A G A G G	5%	5%	7%	2%			
VIM-XM_006717500.2eU760-XP_006717563.1aa204Q*	2	27%	8%	21%		0	1	27%		310	480	240	190	410	C T T C A G A G A G A G G A A G C C G A A A A C A C C C T G C A A T C T		21%		33%	27%		
VIRMA-NM_015496.5eU5081-NP_056311.2aa1687RC	2	2%	0%	2%		0	0			210	220	740	300	470	C C A C T C A A A G T A T C A C A G A A G A T T T C T T C C C G T G G T			2%	3%			
VKORC1L1-NM_001284342.3eU4978-3'UTR+4146	2	7%	1%	6%		0	3	76%	12%	150	270	630	260	340	T G T A G A A G T T A C C T G T G G A A G T T G T G C T C C C A T T A T		6%		7%	63%	85%	82%
VPS50-NM_001257998.2eU3752-3'UTR+727	2	23%	7%	23%	7%	0	0			420	270	740	320	180	T G A A A A G T A A T T T C A T T G A G G G T A C C T T T T C A A T G C	29%	18%					
VPS72-NM_001271087.2eU1221-3'UTR+17	2	2%	0%	2%	0%	0	0			330	80	660	270	300	T T G T C A T T A A A T G A A G A G A T G T C T A G T C C T C A G A A A	2%	2%					
VRK3-NM_001025778.2eU622-NP_001020949.1aa151SL	2	3%	1%	3%	1%	0	0			450	190	550	470	340	G T G A C T C A G G A C C A C A G A A G C A A A A G T T C T C A C T C A		2%	4%				
WARS1-NM_004184.4eU610-NP_004175.2aa158LL	4	3%	1%	3%	0%	0	0			270	210	780	290	250	G A T G C C T A T G A A A A T A A G A A G C C A T T T T A T C T G T A C	3%	3%	4%	1%			
WASHC2A-NM_001005751.3eU3951-NP_001005751.1aa1300SF	4	15%	4%	17%	3%	0	2	2%	0%	270	170	820	220	330	T A T T T G A T G A T G A T A T G G A T G A C A T C T T C T C C T C T G	16%	14%	20%	10%		2%	2%
WASHC2C-NM_001169106.2eU3765-NP_001162577.1aa1238SF	4	15%	4%	16%	3%	0	2	3%	0%	270	220	820	220	330	T A T T T G A T G A T G A T A T G G A T G A C A T C T T C T C C A C T G	18%	13%	18%	10%	3%	3%	
WBP1-NM_012477.4eU698-NP_036609.1aa178SF	3	5%	2%	5%	2%	0	3	43%	12%	470	330	670	360	290	T T G A A G G A A C A A A T G T G G A A G G T G T T T C C T C C C A C C	7%	4%	4%		39%	33%	57%
WDR75-NM_001303096.2eU2126-NP_001290025.1aa627PP	4	5%	1%	5%	1%	0	3	49%	1%	560	220	540	570	370	C A A A C A G C T G C T A G C A G A A G A A A G T C T T C C C A C A A C	5%	6%	4%	4%	48%	49%	51%
XRCC1-NM_006297.3eU1939-NP_006288.2aa624HY	4	21%	5%	24%	3%	0	0			420	140	740	330	300	A G T T G C A A T G A G A A G C A G A A G T T A C T T C C T C A C C A G	26%	20%	24%	14%			
XRN2-NM_001317960.1eU2467-NP_001304889.1aa809PL	4	9%	3%	10%	2%	0	1	1%		230	390	720	370	320	A G T T T T C T T T G G A T G A A G A A G C C A T T C T T C C A G A T C	12%	10%	9%	5%		1%	
YEATS2-NM_001351369.2eU6049-3'UTR+1708	4	3%	1%	3%	1%	0	0			490	230	770	260	190	T G A G G G C T G C A A G T A C A G A A G G A A T C T A T T C T C A G C	3%	3%	2%	4%			
YIPF3-NM_015388.4eU556-NP_056203.2aa146PL	4	28%	11%	33%	3%	0	2	8%	0%	360	360	560	490	260	C C A T G A T C C C T A T C A A G A T G G T C A A C T T C C C C C A G A	37%	32%	31%	12%	8%	8%	
ZBTB1-NM_001123329.2eU1202-NP_001116801.1aa336TI	2	6%	2%	6%	2%	0	0			320	250	620	260	470	T T A T T A A G A T G G A G C C A G A A G A T A T T C C T A C A G A T G	8%	5%					
ZBTB40-NM_001083621.2eU1266-NP_001077090.1aa252SL	4	10%	4%	11%	3%	0	3	26%	4%	320	240	830	180	310	A T T T T C T T C T A G A A A A T G A A G G T G T C T T C T C A G A T G	14%	8%	12%	6%	30%	22%	27%
ZC3HAV1-NM_001363491.2eU3235-NP_001350420.1aa949SF	2	3%	0%	3%	0%	0	0			450	160	730	260	190	G A A T T T A C T T T G C A A A A G A T G C C A T C T A T T C C C A C A		2%	3%				
ZC3HAV1-NM_001363491.2eU4598-3'UTR+1134	4	13%	2%	14%	1%	0	3	10%	3%	230	300	860	270	470	A T T A A T A T G A T T A C A G A G A A G G C T T T T T C T C A G G T C	15%	15%	13%	10%	8%	10%	14%
ZC3HC1-NM_001282190.2eU1058-NP_001269119.1aa314SF	4	5%	2%	6%	1%	0	0			370	230	710	420	200	T G A T G A C C C G G A G C C A G G A T G C C A C T T T C T C C C C A G	5%	7%	4%	3%			
ZDHHC12-NM_001318015.2eU849-NP_001304944.2aa272SL	2	3%	1%	3%	1%	0	0			430	80	760	250	250	G C A A C A C C A C C A C C T G G G A A T T C A T C T C C T C A C A C C	4%		2%				
ZDHHC20-NM_001286638.2eU329-NP_001273567.1aa12TI	2	19%	13%	19%	13%	0	0			290	210	490	500	480	T T G T A T G G T C C T A T T G G A T G A C A A T T T T C A C A T C T C		28%	11%				
ZDHHC20-NM_001330059.2eU356-NP_001316988.1aa75TI	4	11%	7%	13%	8%	0	0			290	210	490	500	480	T T G T A T G G T C C T A T T G G A T G A C A A T T T T C A C A T C T C	22%	10%	7%	5%			
ZDHHC4-NM_001134387.2eU866-NP_001127859.1aa146PL	4	13%	5%	15%	2%	0	0			380	370	710	320	110	A T G T T T A T G A A T T T G A T G A A G T G A T G T T T C C A A A G A	15%	17%	13%	6%			
ZNF148-NM_001348424.1eU3186-NP_001335353.1aa785PS	3	14%	3%	14%	3%	0	0			210	170	780	220	420	A A T G A T A A T A G A G C T G G G A T G A C A T C T T C A C C T G A T	10%	17%	14%				
ZNF211-NM_001265597.3eU693-NP_001252526.1aa172Q*	3	13%	3%	11%	2%	0	0			260	260	620	330	580	T T C A G G A C T T C C A A A G A A G G T T C A T C T T C C C A G A A T		10%	13%	16%			
ZNF211-NM_001265600.3eU308-NP_001252529.1aa46Q*	2	13%	4%	16%		0	0			260	260	620	330	580	T T C A G G A C T T C C A A A G A A G G T T C A T C T T C C C A G A A T			16%	10%			
ZNF236-NM_001306089.2eU1063-NP_001293018.1aa338Q*	2	36%	20%	36%	20%	0	0			390	110	680	300	140	A C G G C C A C A C T T T T T C A G A C G T T A C C T C T T C A A C A G		50%	21%				
ZNF367-NM_153695.4eU2549-3'UTR+1158	3	2%	0%	2%	0%	0	0			290	390	750	390	170	A A C T T G T G T G G T G G T T T G A A G C C A T T T T T C C A A A T A	2%	2%	3%				
ZNF44-NM_001164276.2eU760-NP_001157748.1aa191HY	3	5%	0%	6%	0%	0	0			300	80	800	140	420	T G T C A T G A A T A T G C A G A G A A G T C A T A T A C A C A T A A G		6%	5%	5%			
ZNF566-NM_001145343.1eU1270-NP_001138815.1aa379Q*	3	11%	1%	11%	1%	0	2	9%	1%	360	240	820	280	290	G A A T G T A A G A T A T G T G G G A A G G C T T A T T C T C A G A G T	12%	11%	10%		8%	10%	
ZNF566-NM_001145343.1eU4734-3'UTR+3339	2	29%	7%	29%	7%	0	1	23%		240	350	790	350	370	T T T G T A T T T T T A G T A G A G A C G G C A T T T T T C C A T G T T		33%	24%			23%	
ZNF623-NM_001082480.2eU2469-3'UTR+629	3	14%	3%	14%	3%	0	1	7%		310	240	710	390	400	C G C C T T T G T T C T T G T T G A A G G G T C T C T T C T C A G C T A	11%	13%	17%				7%
ZNF644-NM_016620.4eU1321-3'UTR+753	3	6%	4%	6%	4%	0	0			210	130	670	390	410	C C T A C T T T G T C A A C A A A G A T G C T A T T T A C C C T T T A C	11%	4%	4%				
ZNF707-NM_001100598.2eU1042-NP_001094068.1aa276LL	2	5%	1%	4%		0	0			280	190	560	310	510	G A A A G C C T T C A A G C A G A A G T C C A A C C T T C T C A G A C A	4%			5%			
ZNF761-NM_001008401.4eU461-NP_001008401.3aa5Q*	2	5%	0%	5%	0%	0	0			230	220	780	350	390	A G A G C A A A G G A G T C A G G G A T G G C T T T T T C T C A G G G T		5%	6%				
ZNF787-NM_001002836.4eU1605-3'UTR+322	2	2%	0%	2%	0%	0	0			290	240	650	600	430	G A C C A G G C C C C A A G C G G A A G G C C A T T T T C C C T G C T C		2%	2%				
ZNF816-NM_001031665.4eU1726-NP_001026835.1aa469Q*	3	12%	4%	12%	4%	0	0			290	240	710	270	140	G G C A G G A G T T T C A G T C G G A A G T C A T C C C T T C A A T A C	9%	10%	16%				
ZNF93-NM_031218.4eU2568-3'UTR+562	2	8%	1%	8%	1%	0	0			460	90	820	300	190	T C A T C A C T A C A A A T T C A G A G G G C A T C T C A T C A C C C T		9%	7%				
ZSCAN12-NR_028077.2eU3108-ncr	3	16%	4%	16%	4%	0	0			240	440	700	350	310	A A G A A A A G G A A A T G T T A G A A G T T A T T T T T C C C T T C C	17%	19%	11%				
ZSCAN18-NM_001145542.1eU2189-3'UTR+458	3	7%	2%	7%	2%	0	0			210	150	720	140	270	C T T T T C G T A C T T G G C G T G A T G T C A A C T T C A C T T C T A	8%	5%	9%				
ZUP1-NM_001361190.2eU280-5'UTR-147	2	8%	1%	8%	1%	0	0			350	230	740	280	70	A T A G T A C T T T A A A A C A T G A A G G C T T C T A T T C A G A G A		9%	8%				

B) PPR56_S7TD>TN

>total: 323

Offtarget (blue > same position in gene)	Rep. 56-N	%CU 56-N-4	STABW 56-N-4	%CU 56-N-3	STABW 56-N-3	**Rep. 56-S7**	**%CU 56 S7**	**STABW 56-S7**	Rep. 56-S4	%CU 56-S4	STABW 56-S4	PPR56 S7 Scoring -2	-1	Match	+1	+2	Binding region	PPR56-native %IMR1	%IMR2	%IMR3	%ELIM1	PPR56-S7mut %I6-2	%I7	%I8	PPR56-S4mut %I1-2	%I3	%I4
ACAD9-NM_014049.5eU236-NP_054768.2aa55PL	1	3%		3%		2	8%	1%	3	38%	1%	340	410	530	680	520	TAGGCAAAATCAAGAAGAAAGAAGTTTTCCCATTTC		3%			8%	9%		38%	39%	39%
ACIN1-NM_001164814.1eU1604-NP_001158286.1aa426PS	0					3	2%	1%	0			270	420	860	190	330	CCACCTTTACTAACAAAGGAAGCATCTTCTCCACCA					2%	2%	3%			
ADAM9-NM_003816.3eU2489-NP_003807.1aa800Q*	1	1%		1%		3	14%	3%	0			250	190	840	130	400	CCACCTCCACCACAACCGAAAGTATCATCTCAGGGA			1%		11%	17%	15%			
ADAR-NM_001025107.3eU4034-3'UTR+251	4	36%	13%	42%	2%	2	2%	0%	3	2%	0%	340	210	700	310	340	AAGAGGCAGAAACCTAAGAAGTTATCTTTTCTTTCT	45%	41%	40%	16%	1%		2%	3%	2%	2%
ADAR-NM_001025107.3eU953-5'UTR-36	0					3	4%	2%	0			300	120	790	210	320	CAGGTTTGGAACCTGAAGACAGCAACTCCACATCTG					5%	6%	2%			
AGFG1-NM_001135187.2eU1863-NP_001128659.1aa533Q*	1	9%		9%		3	54%	19%	3	10%	3%	310	220	820	230	480	GCAGCTTTCCCTCAACAGACAGCTTTTTCTCAACAG		9%			37%	50%	75%	7%	9%	14%
AGFG1-NM_001135189.2eU1671-NP_001128661.1aa469Q*	4	5%	2%	6%	1%	3	51%	7%	2	4%	1%	310	220	820	230	480	GCAGCTTTCCCTCAACAGACAGCTTTTTCTCAACAG	7%	6%	4%	3%	51%	57%	43%	4%		3%
AGO2-NM_001164623.3eU6412-3'UTR+3807	0					2	12%	5%	0			300	300	760	430	500	ACCTAATGGATATGATAGAAATTATTTCCCCAACTT					8%		15%			
AIDA-NM_022831.4eU2690-3'UTR+1595	4	14%	5%	16%	3%	2	2%	0%	0			270	350	560	330	430	GCTCTTCAAGGAGCAAGAGGCGCATTTTCTCCGTGT	19%	13%	16%	7%	2%		2%			
AK1-NM_001318121.1eU691-NP_001305050.1aa184SF	0					3	5%	2%	3	6%	3%	500	210	740	180	360	ACGCTGAGGGCTCCGTGGACAGTGTCTTCTCCCAGG					3%	6%	5%	3%	10%	5%
AKAP13-NM_006738.6eU2564-NP_006729.4aa786SF	0					2	15%	8%	0			490	170	750	220	430	ACCAGGCAGTAATATCAGACAGTACTTTCTCTCTGG						9%	20%			
ALG3-NM_001006941.2eU1057-NP_001006942.1aa270SL	0					3	4%	0%	0			410	190	790	270	340	GGTGGCACAGGACAGGGGAAAGTATCTTGTCGCTGC					4%	5%	4%			
AMOTL2-NM_001278683.1eU2507-NP_001265612.1aa797TI	0					2	3%	0%	0			340	140	740	220	320	AGACTGAGGGCCCCCCAGACAGCACCTCCACCTGCC					3%		3%			
ANKFY1-NM_001257999.3eU5295-3'UTR+1542	0					2	2%	0%	3	8%	2%	440	40	630	250	320	TTGTTCATTCTTTTCTGGATAGTGTGTCCTCTGTAT					2%	3%		6%	8%	10%
ANKRD33B-NM_001164440.2eU6171-3'UTR+4288	3	6%	2%	6%	2%	3	19%	6%	0			230	190	600	400	240	TAGTGAATTTCAAGTTGACAGGTACCTTCTCAGGGA	7%	8%	4%		26%	17%	14%			
ANXA7-NM_001156.5eU448-NP_001147.1aa134Q*	0					3	10%	2%	3	20%	3%	310	280	480	400	440	GGTGGCTTTCCTGGAGGACAGATGCCTTCTCAGTAT					11%	7%	11%	16%	22%	22%
APBB1IP-NM_019043.4eU2547-3'UTR+229	3	15%	9%	15%	9%	3	29%	5%	0			210	220	730	370	400	GTATTTTAACCTAAATGGAATGTATCTTCCCTTCCA	15%	25%	6%		34%	25%	27%			
APBB2-NM_001166050.2eU2984-3'UTR+153	0					2	8%	1%	0			410	230	690	350	250	TAACCAAGCAGTGTTAGACAAGCATGTTCTCTCGTC					8%		9%			
APEH-NM_001640.4eU1700-NP_001631.3aa550SF	0					3	9%	4%	0			350	260	790	160	370	CCACGGGCTTTGGCCAGGACAGCATCCTCTCCCTCC					12%	5%	9%			
ASXL2-NM_001369346.1eU2451-NP_001356275.1aa777PS	0					3	9%	2%	0			400	340	880	240	390	AGTAGTTGCAGACAGGAGAAAGCACCTTCTCCAACA					9%	11%	7%			
ATAD2-NM_014109.4eU2266-NP_054828.2aa726HY	0					2	2%	1%	0			380	100	760	240	550	ATTTTAGAAGCCCTGCAGAGAGTATTTCCACATGCA						2%	1%			
ATF3-NM_001030287.4eU1316-3'UTR+689	0					3	2%	1%	0			280	290	710	410	430	CTTGTCAGCAAGAGGAGGACAGAATTCTCCCAGCGT					1%	3%	1%			
ATP2B1-NM_001001323.2eU2726-NP_001001323.1aa786SL	0					2	5%	1%	0			340	170	730	160	330	TGGTTAAAGGTATAATTGACAGCACTGTCTCAGACC					4%		6%			
ATP2B4-NM_001001396.3eU7475-3'UTR+3110	0					3	6%	3%	0			500	240	750	320	320	CACCATGCAGATGGATAGACAGAATCTTTCCTGACT					3%	9%	6%			
ATP5F1A-NM_001001935.3eU1572-NP_001001935.1aa428PP	4	2%	1%	2%	1%	3	28%	3%	0			190	290	710	330	530	TGAGTTGCTGAAGCAAGGACAGTATTCTCCCATGGC	2%	2%	1%	1%	24%	30%	29%			
BAG5-NM_001015048.3eU895-NP_001015048.1aa275SS	0					2	4%	1%	0			350	240	730	260	360	TAAAGCATTTGACCTGAGACAGAATCATTCCATTTT					3%	5%				
BCAR1-NM_001170714.3eU868-NP_001164185.1aa195SL	3	3%	1%	3%	1%	3	28%	8%	0			280	240	550	440	180	CTCCCCCAGCCAAGCAGACATCCACCTTCTCGAAGC	2%	4%	3%		25%	37%	22%			
BCAS2-NM_005872.3eU792-3'UTR+90	4	5%	2%	6%	1%	3	17%	4%	0			300	270	790	320	340	AATGAACTTTGAAGGACAACAGCATCTTCCCAAAAC	5%	7%	5%	2%	13%	18%	20%			
BMPR1A-NM_004329.3eU3005-3'UTR+838	0					3	22%	11%	0			300	390	830	210	410	TTATTATTTTTGTCACGGAAAGCATCCTCTCCAAAG					21%	11%	33%			
BMPR2-NM_001204.7eU10980-3'UTR+6715	0					2	24%	9%	0			310	260	860	170	440	TAAAGTAGTTCCTGTCAGATAGGATTTTCTCAAGAG					18%	30%				
BRMS1L-NM_032352.4eU1558-3'UTR+407	0					3	16%	8%	0			450	290	660	320	200	TGTTTGTAAATAAGGCCGATAGAATGTTTCCTATAA					8%	24%	16%			
BUB1B-NM_001211.6eU1858-NP_001202.5aa569SL	0					3	14%	6%	0			350	170	860	200	340	CAGTTCTCAAAACCTCAGAAAGCATCACCTCAAATG					7%	19%	15%			
C1QTNF5-NM_001278431.2eU915-NP_001265360.1aa228SF	2	27%	22%	27%	22%	3	64%	11%	0			350	210	840	150	350	ATGCCAGCATCAAGACAGACAGCACCTTCTCCGGAT		43%	12%		54%	63%	76%			
C2CD3-NM_001286577.2eU7252-NP_001273506.1aa2348Q*	0					2	4%	2%	0			310	110	750	240	400	ACTCTCAGGATTGCACGGATATTTTCTTCTCAGTAC					2%		5%			
C7orf26-NM_001303039.2eU1825-3'UTR+503	0					3	19%	2%	0			310	310	850	270	230	GAATTCCCCTCACCTCGGAAAGGAACTTCTCAGTTT					19%	22%	17%			
CACNB3-NM_000725.4eU1689-NP_000716.2aa419Q*	0					2	7%	1%	0			250	150	830	180	350	AGCTCCCGCCAAGCCTGGACAGGATCTTCACAGCGT					8%		7%			
CALCOCO2-NM_001261390.2eU1415-NP_001248319.1aa454PL	0					2	5%	0%	0			290	310	790	200	460	TCAATTGTCCAATTTGTGACAAGATCTTCCCAGCTA						5%	5%			
CAV2-NM_001206747.2eU691-3'UTR+158	0					3	2%	0%	0			390	470	470	230	230	CATTTAGAAATGTTTATTGACAGGTCTTTTCAAATA					2%	2%	2%			
CAVIN2-NM_004657.6eU151-5'UTR-14	0					3	3%	1%	0			480	260	800	420	430	GAAGCAGCTCCCAGAGAGAAAGAACGTTCCCACCTG					2%	3%	5%			
CAVIN2-NM_004657.6eU905-NP_004648.1aa248RC	3	5%	0%	5%	0%	3	50%	4%	0			410	250	890	300	440	AAAGTGGATAGCCTCAAGAAAGCATTTTCTCGCCAG	5%	6%	5%		47%	49%	55%			
CCDC43-NM_001099225.2eU1675-3'UTR+1187	3	4%	1%	4%	1%	3	41%	0%	0			400	250	750	290	330	TTGTCTAGAAGCCTAAGGACAGCAATTTCTCTGACA		4%	3%	4%	41%	42%	41%			
CCDC71L-NM_175884.6eU1351-3'UTR+381	2	5%	3%	5%	3%	3	22%	6%	0			370	350	710	350	400	TCTGGAAGTCCTCCGAAGACATTATTTTTCCAAAGG	7%	3%			17%	28%	20%			
CCT6A-NM_001009186.2eU1653-3'UTR+108	4	1%	0%	1%	0%	3	5%	1%	0			350	410	610	640	480	AGCTTCAAATAATTTTGAAAGAAATTTTCCCATATG	2%	1%	1%	1%	5%	5%	4%			
CDC25A-NM_001789.3eU3224-3'UTR+1179	3	3%	1%	3%	1%	3	32%	4%	0			400	170	820	330	360	TGGCATGGACATGACTGGATAGAATTTTCTCAGGAG	1%	4%	4%		30%	36%	29%			
CDC42-NM_001039802.2eU1359-3'UTR+573	2	1%	0%	1%	0%	3	5%	1%	0			290	500	810	290	460	TTCCTAGATCTAGTTTAGAAAACATGTTCCCCATCT	1%	1%			6%	5%	4%			
CDH2-NM_001792.5eU276-NP_001783.2aa56LL	3	3%	1%	3%	1%	3	40%	2%	0			310	200	830	220	390	GAAGGATGTGCATGAAGGACAGCCTCTTCTCAATGT	3%	4%	2%		39%	42%	39%			
CDK12-NM_015083.4eU1691-NP_055898.1aa369HY	0					3	13%	5%	0			240	190	660	350	290	AGTCCTGCATATTCAAGACATTCATCTTCTCATAGT					8%	16%	16%			
CENPJ-NM_018451.5eU3964-NP_060921.3aa1266PL	3	8%	3%	8%	3%	3	28%	2%	0			300	320	900	400	440	TTCCTGATGGACAAGAAGAAAGCATTTTCCCAGATG	5%	11%	7%		25%	29%	30%			
CGGBP1-NM_001008390.2eU253-5'UTR-270	1	2%		2%		3	5%	2%	0			400	250	760	470	460	AATCGGGTGCAAGCCCAGAAACTATTTCCCCACCAC			2%		3%	7%	5%			
CGGBP1-NM_001008390.2eU3571-3'UTR+2546	0					2	4%	1%	0			430	250	640	210	260	TGTTTTCTCAAAGTATTGATAGTATGTCTTCCAGAA						5%	4%			
CHAC2-NM_001008708.4eU578-NP_001008708.1aa165FF	4	24%	4%	26%	3%	2	6%	1%	0			520	210	630	300	190	TGTGCCAGAAGAAGCAGATGAGCATCTTTTCGCTTT	24%	29%	26%	19%	7%		5%			
CHSY1-NM_014918.5eU4426-3'UTR+1425	1	2%		2%		2	3%	1%	0			360	270	590	430	330	ATCTGTCATTATACCAGAAAGTTAAATTCTCAGATC		2%			2%		3%			
CIZ1-NM_001131015.2eU643-NP_001124487.1aa201Q*	0					2	2%	0%	0			240	230	700	250	330	ACCACCCCCAATCGAAAGGATTCTTCTTCTCAGACA					2%		2%			
CKAP4-NM_006825.4eU1775-NP_006816.2aa536LL	0					2	2%	0%	0			430	160	660	200	560	CCTCAGGACTTCCTGGACAGACTTTCTTCTCTAGAC					1%	2%				
CLCN6-NM_001256959.2eU4861-3'UTR+2245	0					3	21%	6%	0			310	340	840	140	350	TTCCAGATAAGCCCAAAGACAGCAACTTCTCCACTC					16%	27%	19%			
CMTM6-NM_017801.3eU2832-3'UTR+2173	1	1%		1%		3	13%	3%	0			290	110	770	140	530	TCTTTCTTTTTCAGAAAGATAGTATGTTCACTGGTA	1%				13%	10%	15%			
CNKSR3-NM_001368116.1eU2068-3'UTR+74	1	7%		7%		3	31%	6%	0			310	150	780	180	350	GCCTTGCTTTTACAGTGGACAGCCTCTTCTCGTTTC	7%				28%	39%	28%			
CNKSR3-NM_001368116.1eU603-NP_001355045.1aa99HY	2	19%	10%	19%	10%	3	41%	8%	0			480	180	870	200	380	AACTTGGTTCTGAAACTGAGAGCATCTTCCCACAAT	26%		12%		39%	50%	35%			
CNOT7-NM_001322090.2eU1310-3'UTR+99	0					3	5%	0%	0			460	340	690	290	310	GAATCCCTCGAACAATAGACAGTACCTTTCCCCCCC					5%	5%	5%			
CNPPD1-NM_001321389.1eU472-NP_001308318.1aa15SF	4	35%	9%	39%	3%	2	17%	2%	3	37%	10%	370	250	790	320	310	GGCTCCTGCTGGACGAAGAAGGCACCTTCTCCCTCG	37%	42%	38%	22%	16%		19%	41%	44%	25%
CNPPD1-NM_015680.6eU141-NP_056495.4aa15SF	4	33%	14%	40%	5%	2	15%	2%	2	25%	3%	370	250	790	320	310	GGCTCCTGCTGGACGAAGAAGGCACCTTCTCCCTCG	43%	35%	42%	12%		16%	13%	28%		23%
COPS3-NM_001199125.1eU1506-3'UTR+164	0					3	5%	1%	0			350	290	730	500	400	GTCCTGTGTGCTTTCAGAAAACCATTTTCTCTGCAA					4%	6%	4%			
COX6B1-NM_001863.5eU158-NP_001854.1aa22PP	0					2	1%	0%	3	7%	0%	260	300	370	680	200	CAAGACCGCCCCTTTTGACAGCCGCTTCCCCAACCA					1%		1%	7%	6%	6%
CPSF7-NM_001136040.4eU1585-3'UTR+52	4	3%	1%	3%	1%	3	7%	1%	0			340	40	600	290	290	AAATGTTTTTTTAATGGACTTGCATCTCCTCACCTT	4%	2%	3%	2%	6%	8%	8%			
CRBN-NM_001173482.1eU325-NP_001166953.1aa99Q*	0					3	5%	2%	0			470	100	680	260	270	ATCCTGATTCCCGGACAGACATTACCTCTTCAGCTT					4%	5%	7%			
CRKL-NM_005207.4eU1516-3'UTR+76	3	16%	2%	16%	2%	3	56%	3%	0			500	250	850	340	440	AGTCTCCTTTGAAGTGGGAAAGCATTTTCTCTCATA	14%	18%	17%		56%	60%	54%			
CSE1L-NM_001256135.2eU1172-NP_001243064.1aa350SF	2	1%	0%	1%	0%	3	4%	1%	0			320	190	610	430	250	TTTTTGAGGGACCTGTGACAGGAATCTTCTCTGGTT		1%	1%		4%	3%	4%			
CTNNA1-NM_001290307.3eU3348-3'UTR+734	4	28%	8%	32%	2%	3	1%	0%	3	7%	1%	220	220	810	330	460	AGGAAGCAGTCCCTGGAGAAGGCATGTTCCCAGAAA	34%	30%	34%	16%	1%	2%	1%	6%	8%	8%
CUEDC1-NM_001271875.2eU835-NP_001258804.1aa111PL	0					2	19%	3%	0			290	160	690	330	330	ACAGCTCCGACTCGGAGGACAGCATCCCCCCGGAGA						21%	17%			
CUX1-NM_001202543.2eU5351-3'UTR+776	0					3	14%	13%	0			400	230	780	470	380	TCCTAAATATTTTCAAGAAAAGAATCTTCTCGTTTG					6%	29%	8%			
CWF19L1-NM_001303404.2eU982-NP_001290333.1aa313PS	0					2	4%	1%	0			440	260	650	180	660	TCATCCACAGGTAGAGATAGCAAATCTTCTCCTCAT						5%	4%			
DCAF1-NM_001171904.2eU3174-NP_001165375.1aa1008TI	0					2	12%	2%	0			320	110	710	120	410	CTTCCCCACCTACGCTGGACAGTATAATCACAGAGT					10%		13%			
DCBLD2-NM_080927.4eU4200-3'UTR+1503	0					2	3%	1%	0			410	340	570	520	290	TTGTAGATTTAATGTTGAACAGTACTTTTCCATAAA						3%	3%			
DIABLO-NM_001371333.1eU298-NP_001358262.1aa87TI	0					2	5%	1%	0			410	200	670	260	260	CAGTGTCTTTGGTAACAGATAGCACCTCTACCTTTC					5%	6%				
DIPK1A-NM_001006605.5eU2437-3'UTR+1117	0					2	8%	3%	0			300	270	710	250	430	CTATGTGACCATCACTGGACAGCTTTCTCTCAAAAC					6%		10%			

DLGAP5-NM_001146015.2eU215-NP_001139487.1aa31Q*	0					3	5%	2%	0			340	250	730	370	290	ACTAAAATTGCTCATAGGAAATCACTGTCTCAGAAA					6%	7%	2%			
DPY19L4-NM_181787.3eU3591-3'UTR+1320	0					3	23%	4%	0			450	230	730	420	340	AAATTACTACATTAGAAGAGAGCATTTCTCCATTGT					28%	20%	22%			
DTL-NM_001286229.2eU2252-3'UTR+146	0					3	4%	0%	0			510	310	800	220	360	CTGCCTTTTCATTTTTAGACAAAATCTTTTCAACGC					4%	4%	4%			
EARS2-NM_001083614.2eU2069-3'UTR+490	0					2	9%	4%	3	7%	1%	330	190	540	430	310	CTTTTGTTGGCTTGCAGACAGCTGTCTTCTCACTGT					6%	12%		7%	6%	7%
EHBP1-NM_001142614.2eU3996-3'UTR+25	0					2	6%	2%	0			510	400	730	480	240	CAGTAGCCATCAGATCAGAAAGAATCTCTCCCAACA					7%		5%			
EIF4E2-NM_001276336.2eU2706-3'UTR+1965	0					2	21%	1%	3	16%	7%	300	400	420	390	210	TGTCGTTGTAGTATTTGACAGTTGTCTTTTCCATCA					21%		20%	9%	21%	19%
EIF4G2-NM_001042559.3eU2473-NP_001036024.3aa722PL	0					3	2%	1%	0			460	410	690	350	360	TATATAAATGGATTAAAGATAACATCTCTCCCAAAC					1%	3%	1%			
ELOC-NM_001204857.2eU295-NP_001191786.1aa68HY	0					2	2%	0%	0			210	360	520	440	370	AATGAGGTCAATTTTAGAGAGATACCTTCACATGTG					2%	2%				
ELP2-NM_001242875.3eU837-NP_001229804.1aa268LF	0					2	12%	1%	3	45%	4%	530	250	710	310	430	CAAAATGATCAGTTTCAGAAAGTGCTTTCTCTCTGT					11%	13%		48%	40%	46%
ELP2-NM_001324465.2eU642-NP_001311394.1aa203LF	0					3	10%	1%	3	37%	6%	530	250	710	310	430	CAAAATGATCAGTTTCAGAAAGTGCTTTCTCTCTGT					9%	12%	10%	30%	43%	37%
ENPP2-NM_001040092.3eU1349-NP_001035181.1aa430PP	0					2	8%	2%	0			340	270	770	330	430	CTTTAAGCCTTACTTGAAACAGCACCTTCCCAAACG					10%		7%			
EPHB4-NM_004444.5eU2242-NP_004435.3aa583II	0					2	8%	1%	0			410	40	690	250	260	ATATTCGGACAAACACGGACAGTATCTCATCGGACA					7%		8%			
EPRS1-NM_004446.3eU2335-NP_004437.2aa740LL	0					2	2%	0%	0			440	240	600	310	300	GCTCCTTTTAAGGAAAGACCAACACCTTCTCTGAAT					2%		2%			
ERCC3-NM_000122.2eU2614-3'UTR+202	1	3%		3%		2	9%	1%	0			470	370	750	210	560	TTTATAAAGGAGGAGGAGACAATATTTTTTCAAACT	3%				10%	9%				
EZH1-NM_001321079.2eU310-NP_001308008.1aa96PL	0					2	38%	18%	0			330	240	810	270	520	TCAAAAAGTGTACCATAGAGAGCATTTTCCCGGGAT					50%	25%				
FAM111B-NM_001142703.2eU350-NP_001136175.1aa79SL	0					2	3%	0%	0			390	40	810	100	480	CATATGGTAAACCCAGCGAGAGTATCTACTCAGCCC						3%	4%			
FAM83D-NM_030919.3eU1320-NP_112181.3aa432Q*	0					3	5%	2%	0			300	290	730	160	300	ACTGCAGTCATCACCAGGATAGCAAGCTCTCAAACC					3%	5%	7%			
FANCC-NM_000136.3eU250-5'UTR-14	0					2	6%	0%	0			520	170	690	270	250	ACAGGCTGCTGTGAAGGGACATCACCTTTTCGCTTT					6%		6%			
G3BP2-NM_012297.5eU1778-3'UTR+166	0					3	3%	1%	0			300	220	680	420	380	TCAGTTAAATTGAGGTGGACATTATTTCCCCAATTT					2%	4%	2%			
GIGYF1-NM_001375759.1eU6285-3'UTR+1642	0					3	4%	3%	0			380	360	700	150	690	GTTAGCGTCCCTGAAAAGACAATATTCTCTCTAAAG					7%	2%	3%			
GINM1-NM_138785.5eU667-NP_620140.1aa209II	0					2	3%	1%	0			270	110	760	60	250	TTCACTGCAAACCACTAGCCAGTATCTTATCAGGAA						4%	2%			
GLRX5-NM_016417.3eU878-3'UTR+364	0					3	16%	4%	0			530	210	760	310	480	ATGTGTCAGTCTCCAAAGAGAGTATCTCCCCCCAAA					12%	16%	21%			
GNB4-NM_021629.4eU3403-3'UTR+2213	1	3%		3%		2	7%	0%	0			480	90	800	210	440	TAACCTTAAATATGTAAGAGAGTATCTCCTCTACTT			3%			7%	7%			
GNG12-NM_018841.6eU4289-3'UTR+3870	0					3	2%	1%	0			260	110	780	60	380	AAAGCATGTACTTCACAGCTAGCATGTTCACAGATT					2%	2%	4%			
GOLGA1-NM_002077.4eU3246-3'UTR+624	0					3	26%	9%	0			340	40	720	170	370	AGAAAATGCACCTTTCTGACAGTATCTCCACTTTAC					20%	36%	21%			
GOLGA4-NM_001172713.2eU5642-NP_001166184.1aa1788HY	0					3	8%	3%	0			310	110	830	160	560	GGGCAGGAAAAAGAAGAGACAGTTTCTTCTCATTTT					5%	9%	11%			
GPAM-NM_001244949.2eU4928-3'UTR+2241	0					2	18%	5%	0			250	240	910	230	390	ACAAAAAACAAAAAAACGAAAGCATCTTCACAATGA					22%		14%			
GPBP1-NM_001127235.2eU210-5'UTR-802	0					3	7%	3%	0			390	240	690	240	210	ACTTAAAAAGAGACCCAGGCAGCATTTCTTCAGTAT					4%	10%	7%			
GRINA-NM_000837.2eU642-NP_000828.1aa121PL	0					3	4%	1%	3	3%	2%	210	350	500	370	330	CCAACCCCTATGGACAGCCACAGGTCTTCCCAGGAC					3%	6%	3%	2%	3%	5%
HADH-NM_001184705.4eU1381-3'UTR+341	0					2	6%	0%	0			490	180	690	460	390	CCTTCTCTCATCAACGGGAAAGTACTTCCTCTGAGA						6%	5%			
HIF1A-NM_001243084.2eU2903-3'UTR+211	3	2%	1%	2%	1%	3	11%	2%	0			310	130	690	170	640	TAATGCTGGATCACAGACAGCTCATTTTCTCAGTTT	3%	1%	1%		12%	11%	9%			
HIF1AN-NM_017902.3eU2893-3'UTR+1827	3	17%	8%	22%	0%	2	12%	1%	0			270	120	680	190	490	CAGTGTGGATGTGAAGATATGGTACCTTCTCAAGTG	22%	22%		8%	12%	11%				
HIPK2-NM_001113239.3eU14647-3'UTR+10756	0					2	5%	3%	0			310	210	690	250	540	TGAAAATTATTGCGTGCAACAGTATTTTCTCGTGTA						7%	2%			
HMMR-NM_001142556.2eU2497-3'UTR+258	0					2	3%	1%	0			280	130	770	260	240	GCTGGCTTTCCAGCTTAGAATGCATCTCATCAACTT					3%		2%			
HMOX2-NM_001127204.2eU1370-3'UTR+170	0					2	20%	8%	0			290	210	790	40	370	TGCTAGAGCCTCTGCCTGACAGCATCCTCTCTATGG						26%	14%			
IFRD1-NM_001007245.3eU1099-NP_001007246.1aa220TI	0					3	6%	1%	0			380	330	780	270	490	CAACTCTGGAATGTTTGGAAAATATCTTCACTAAAT					7%	6%	7%			
INPP4A-NM_001134224.2eU5034-3'UTR+1707	0					3	10%	2%	0			380	220	710	200	380	GTCAATTTCTGTTCACAGACAGTTTGTTTTCAATAT					8%	10%	11%			
INTS4-NM_033547.4eU831-NP_291025.3aa269PL	0					3	13%	5%	0			190	370	790	300	360	TCAGTCAGCTCTATCCTGAAAGCATTGTCCCAATTC					18%	12%	8%			
INTS6-NM_001039937.2eU2514-NP_001035026.1aa819TI	0					2	6%	1%	0			380	190	820	310	350	AGCCAGGAAGAAAATATGAAAGAATCTTCACTTTAC					7%		5%			
JMJD1C-NM_001282948.2eU6940-NP_001269877.1aa2049SL	0					3	4%	1%	0			370	220	810	120	320	ATCTCCTGAACTGCAAAGATAGCATCATTTCAAATG					4%	5%	4%			
KCNN4-NM_002250.3eU1869-3'UTR+473	0					3	26%	14%	0			370	300	710	470	260	GGTGGGACAACAAAGAGGACACCATTTTTCCAGAGC					33%	9%	35%			
KIAA1191-NM_001079684.3eU2575-3'UTR+1414	0					3	20%	2%	0			390	110	690	230	190	ACCTGCTTTTTGTTTTGGATAGTACCTTGTCTGTAT					22%	21%	18%			
KIAA1958-XM_011518311.2eU11735-3'UTR+7541	0					3	37%	8%	0			320	380	740	210	390	ATATTACACTGGAATTAGACAGCATTTTTACCATTT					38%	44%	29%			
KIF20A-NM_005733.3eU1266-NP_005724.1aa385SL	0					3	4%	1%	0			310	160	830	100	420	AGAACTCCAGCCGCAGTCACAGCATCTTCTCAATCA					4%	3%	5%			
KIF2A-NM_001098511.3eU449-NP_001091981.1aa61SL	2	7%	3%	7%	3%	3	54%	6%	0			560	170	840	220	240	GCAAAGAGATTGACCTGGAGAGCATCTTTTCACTTA		4%	9%		50%	61%	51%			
KMT5B-NM_001300907.1eU3296-3'UTR+286	1	14%		14%		2	9%	1%	0			470	320	740	240	250	AAGCAAAATTGTACAGTGATAGCAACTTTCCACACA		14%				10%	9%			
LATS2-NM_014572.3eU355-5'UTR-92	0					2	5%	2%	0			320	300	750	230	310	CAAAAGGAAATTATTTTGAAAGTATGTTTACAACAA						4%	6%			
LINC01873-NR_130931.1eU1623-ncr	0					2	14%	6%	0			300	240	530	330	210	GCCGATGCAATCAACTGGAAGAAAGGGTATCAGCGA					10%	18%				
LPCAT3-NM_005768.6eU823-NP_005759.4aa257LL	4	24%	11%	30%	2%	2	4%	1%	3	5%	1%	540	80	690	410	400	CAGCCCCCACATCACAGAAGACTATCTCCTCACTGA	27%	31%	31%	8%		5%	4%	4%	5%	6%
LRIF1-NM_001006945.2eU895-NP_001006946.1aa194PL	4	46%	16%	54%	5%	3	2%	0%	3	5%	2%	210	370	690	400	540	TCAATAAAAAATTATACCGAAGATATTTCCCAGTGA	59%	50%	51%	23%	2%	2%	3%	5%	3%	7%
LRRC58-NM_001099678.2eU6468-3'UTR+5241	0					2	5%	0%	0			320	280	480	460	320	GAAAGACTGTGATCCTGACAGATTCCTTCTCATAAA					5%	5%				
M6PR-NM_001207024.2eU881-3'UTR+146	4	3%	1%	3%	1%	3	8%	2%	0			290	110	660	220	250	TTTCCAGTTTGCTTTTGATTTGCATCTTCTCACTAG	4%	4%	3%	1%	7%	10%	7%			
MAML1-NM_014757.5eU4607-3'UTR+1265	0					3	6%	1%	0			470	170	690	390	450	TGGAAAACGAAGATGGAGAGAGCACTTCCCCGTAAC					6%	5%	7%			
MAML2-NM_032427.4eU6852-3'UTR+2081	0					3	22%	1%	0			380	310	650	380	210	GTTTTCCGATTTTATAAGGCAGCATTTCTCCATACA					22%	21%	24%			
MAP3K2-NM_001371910.2eU5374-3'UTR+2925	1	4%		4%		2	7%	0%	3	6%	1%	300	100	430	410	430	AATGATATGGATCAACAAATGCAATTTGAACTTTTT		4%			7%		7%	6%	5%	6%
MAP4K3-NM_001270425.2eU3164-3'UTR+244	0					2	7%	3%	0			380	310	660	170	350	TAATCTTTGTACATATAGGCAGTATTTTTTCTGTGA						4%	9%			
MAP4-NM_001384675.1eU629-NP_001371604.1aa178PP	0					2	11%	0%	0			260	260	420	540	280	AAATGATCCCTTGAAAGACAGTTACGTTCCCTTAGA					11%		10%			
MARCHF7-NM_001282805.2eU946-NP_001269734.1aa235R*	0					2	2%	0%	0			350	270	660	320	450	AATTTTTCTTCAAGAGAATCAGAATCTTCCCGAAGC					2%	2%				
MBIP-NM_016586.3eU918-NP_057670.2aa290PL	4	52%	11%	56%	10%	2	7%	1%	3	21%	5%	380	210	640	520	160	ATAAAATCCTTGAATTGGAAGGCATCTCTCCTGAAT	67%	52%	49%	40%	6%		7%	15%	22%	25%
MCMBP-NM_001256378.2eU1461-NP_001243307.1aa401TI	0					3	4%	2%	0			340	150	690	310	460	TGAGTGGTTGCCCACGGAATAGTACCTTCACAGAAC					3%	3%	7%			
ME1-NM_002395.6eU319-NP_002386.1aa74LL	3	11%	3%	11%	3%	3	11%	5%	0			280	190	610	350	310	TCTGAACTCTGACTTTGACAGGTATCTTCTCTTAAT	13%	7%	12%		9%	17%	7%			
MGME1-NM_001310338.2eU1873-3'UTR+610	0					3	31%	5%	0			330	260	770	140	530	ACATTGCTGACTATAAAGACAGTATATTCACCATGT					25%	32%	34%			
MGME1-NM_001310338.2eU791-NP_001297267.1aa203SL	0					2	10%	3%	0			500	300	760	250	270	GGTTCCACGAAGCCTTGGAAAGCATACTTTCACCCC					8%		13%			
MGME1-NM_052865.4eU746-NP_443097.1aa188SL	0					2	11%	8%	0			500	300	760	250	270	GGTTCCACGAAGCCTTGGAAAGCATACTTTCACCCC						17%	5%			
MKNK1-NM_001135553.4eU231-5'UTR-25	4	69%	22%	80%	6%	3	59%	8%	3	69%	2%	280	120	820	140	480	GGGATTTCAAGTTTACAGATGGTATCTTCTCAAAAG	87%	76%	77%	36%	50%	65%	61%	71%	67%	70%
MKNK1-NM_001377343.1eU189-5'UTR-56	4	63%	20%	72%	8%	3	63%	20%	3	67%	8%	280	120	820	140	480	GGGATTTCAAGTTTACAGATGGTATCTTCTCAAAAG	64%	78%	75%	35%	78%	40%	70%	67%	75%	60%
MOB1A-NM_001317110.2eU3652-3'UTR+2799	4	41%	13%	47%	4%	3	1%	0%	3	9%	1%	470	80	750	340	340	TTCCCCCTTGCTAATCAGAAGCCATCTCCTCTCAGT	52%	44%	45%	21%	2%	1%	2%	7%	9%	10%
MRE11-NM_005591.4eU2056-NP_005582.1aa633R*	0					2	8%	0%	2	7%	2%	350	310	390	660	220	GCCTTTAAATCTACAAGACAGCAGCCTTCCCGAAAT						8%	9%		6%	9%
MRPL3-NM_007208.4eU1282-3'UTR+85	0					3	2%	1%	0			330	340	780	130	450	TGATATCATAACCACCAGAAATCATACTCTCCTTTC					1%	2%	2%			
MTBP-NM_022045.5eU249-NP_071328.2aa66PL	0					3	12%	4%	0			330	240	710	360	430	CTTCAATTAATCCAGAAGATAGTACTTTCCCTGCCT					8%	15%	13%			
MTHFD1-NM_001364837.1eU884-NP_001351766.1aa270PL	0					2	2%	1%	1	1%		230	250	330	370	110	AGGCCAAAGAGAGGGCGAGCTTCATCACTCCTGTTC					1%	2%		1%		
NAA25-NM_024953.4eU2986-3'UTR+57	0					2	5%	1%	0			250	180	830	280	300	ACTCTGCAACAGAATATGACACCATCTTCCCAGGCA					6%		5%			
NCAPD3-NM_001372065.1eU1093-NP_001358994.1aa351PL	0					3	11%	3%	0			410	180	780	320	440	TTGTGGATGAATTAAAGGAGAGTATATTCCCAGTCG					10%	8%	15%			
NCOA3-NM_001174087.2eU6718-3'UTR+2215	0					2	8%	0%	0			330	260	740	40	510	GTGGCCTGTGCTTTTCAGATAGTATACTCTCCTGTT					8%		8%			
NDUFS3-NM_004551.3eU852-3'UTR+39	0					2	5%	2%	3	16%	3%	430	160	690	150	210	AACGCATGTGGATCCTAGACAGCGCCTTATCTATGA					4%		6%	17%	19%	13%
NFASC-NM_001005388.3eU5206-3'UTR+1153	0					3	17%	5%	0			310	110	910	100	380	CCCTTCCATCTCTAGCAGATATCATCTTCTCAGCCA					23%	14%	14%			
NFAT5-NM_001113178.3eU10364-3'UTR+5381	0					3	6%	1%	0			400	250	850	260	360	TTCAGGAAGAAAGTGTTGAAAGCATTTTCTCTGATG					7%	7%	4%			
NHP2-NM_001034833.2eU525-3'UTR+184	4	41%	14%	48%	1%	3	7%	1%	3	19%	2%	170	140	780	240	340	TCCTGCCCACCCACACTGACGGCATCTTCCCAGTTC	49%	49%	47%	20%	8%	6%	8%	19%	18%	21%
NHP2-NM_017838.4eU631-3'UTR+101	4	46%	13%	52%	2%	3	7%	3%	3	26%	5%	170	140	780	240	340	TCCTGCCCACCCACACTGACGGCATCTTCCCAGTTC	55%	52%	51%	27%	4%	9%	8%	23%	23%	31%
NMD3-NM_015938.5eU2431-3'UTR+800	4	59%	17%	67%	6%	3	5%	2%	3	21%	2%	460	210	760	380	520	TTTTGTTTATAAATTGAGAAGGAATTTTCTCTCTAT	73%	62%	67%	35%	7%	5%	3%	23%	22%	20%
NOL8-NM_001256394.2eU4002-3'UTR+301	3	11%	2%	11%	2%	3	8%	3%	1	3%		310	200	660	180	450	TCTATCCTGAGTAACTAATGGACATCTTCTCATGCA	9%	12%	13%		10%	5%	8%	3%		
NOLC1-NM_001284388.2eU2990-3'UTR+838	4	9%	3%	10%	2%	3	21%	2%	1	1%		230	70	770	220	300	TTGAGTTCTAAGTTGAGGACTGCATCTTCTCGTTTT	8%	12%	11%	5%	20%	23%	20%			1%
NONO-NM_001145408.2eU2435-3'UTR+752	4	63%	19%	73%	2%	3	6%	1%	3	20%	0%	360	300	760	340	560	CCCCTTCATGTCCTAAAGAAGACATTTTCTCTTAGA	74%	73%	70%	35%	7%	7%	6%	21%	21%	20%

NPPB-NM_002521.3eU137-NP_002512.1aa9RW	4	4%	1%	5%	0%	3	37%	1%	0			290	180	800	250	310	AGAGACATGGATCCCCAGACAGCACTTCCCGGGCG	5%	5%	5%	2%	36%	37%	38%			
NR2C2-NM_001291694.2eU2093-NP_001278623.1aa577PL	0					3	4%	1%	0			320	280	740	140	420	TTGGCAATGTTTCGATAGACAGCATAATCCCCTACA					3%	5%	5%			
NRG1-NM_001159995.3eU1145-NP_001153467.1aa332SF	0					2	9%	2%	0			370	400	760	170	310	GGAGCAACGGACACACTGAAAGCATCCTTTCCGAAA						7%	10%			
NUAK2-NM_030952.3eU2390-3'UTR+386	0					3	6%	1%	0			330	230	680	430	270	ACATGGTGCCTTCTAAGGACAGCTCCTTCCCTACTC					7%	5%	6%			
NUP43-NM_198887.3eU2393-3'UTR+1216	0					3	10%	4%	0			370	290	720	320	300	TTTTAAGATAATTTAATGATACTATCTTTCCAAACT					12%	6%	13%			
OBI1-NM_024546.4eU1989-NP_078822.3aa652Q*	0					3	18%	3%	3	19%	4%	380	240	740	350	440	CCAAGCTGCTTGTTTCAGACAGAGTTTTCCCAGGGC					14%	20%	20%	15%	23%	20%
OSBPL2-NM_001278649.3eU1600-3'UTR+61	0					3	12%	4%	0			280	180	790	140	460	ACGACTGAGTTCGCGGAGATAGCATCATCCCTGATC					11%	17%	9%			
OSBPL9-NM_001350208.2eU225-5'UTR-538	3	4%	2%	5%	1%	3	44%	4%	0			290	160	810	190	280	GTATAGACGATGAGGACGACAGCACCTTCACAATAA	5%	4%		2%	40%	46%	47%			
OTUD7B-NM_020205.4eU2588-NP_064590.2aa748SF	0					2	6%	1%	0			510	150	660	270	230	GACCCTACCCCCACCAGGACAGCATCCCTTCTCTGG					5%		7%			
PABPC4-NM_001135653.2eU1785-NP_001129125.1aa316PS	0					3	4%	1%	0			400	350	810	460	400	GATGATGAGAAATTAAGGAAAGAATTTTCTCCTTTT					4%	6%	3%			
PAPOLG-NM_022894.4eU3708-3'UTR+1286	0					2	44%	8%	0			430	220	840	220	280	TTTTGTATGTACTGTAAGATACCATCTTTTCAAAGA					50%	39%				
PARL-NM_001324436.2eU596-NP_001311365.1aa188LL	0					3	7%	2%	0			470	110	760	230	370	CTTGTATTCTGTTTATGGAGAGTACCTTCTCTGCAG					9%	5%	8%			
PCLAF-NM_014736.6eU415-3'UTR+9	0					2	2%	1%	0			440	210	680	500	370	CACAAATGATGAAAAAGAATAGAACTTTCTCATTCA					2%	2%				
PCSK9-NM_174936.4eU279-5'UTR-13	0					3	13%	8%	0			350	340	730	160	300	CTAGGTCTCCTCGCCAGGACAGCAACCTCTCCCCTG					9%	22%	9%			
PCYOX1-NM_016297.4eU5208-3'UTR+3662	0					3	16%	7%	0			230	210	750	120	420	TAGATGAATACATTGTGGACAGCATCCTCACTACCC					8%	22%	17%			
PEG10-NM_001040152.2eU3267-3'UTR+1855	0					2	2%	0%	3	9%	1%	380	300	690	290	540	CATCTCAGGCCTGAAAAGAAAGTGCTCTCTCAGATG					2%		2%	8%	10%	9%
PHACTR2-NM_001100164.2eU6956-3'UTR+4819	0					3	41%	6%	0			240	230	800	180	440	ACCAATGCTTTTTTGAAGACAGTATCATCCCAAATT					39%	36%	47%			
PHF6-NM_001015877.2eU2407-3'UTR+1107	0					3	5%	1%	0			350	290	860	260	490	AAGAACCCAGCTTTTTAGAAAGGATTTTCACACTGG					4%	5%	5%			
PHF6-NM_001015877.2eU4050-3'UTR+2750	0					2	4%	2%	0			400	100	740	220	390	GCAGTTTTAGAATTTCAGATAGTACTTGCTCAGAAG						5%	2%			
PHYKPL-XM_024446242.1eU1589-5'UTR-2097	0					2	21%	6%	0			340	250	570	420	410	AGTGGCTTAAACAAAAGACATTTATTTTCTCACAGT					17%		25%			
PIK3CB-NM_001256045.2eU2677-3'UTR+582	0					2	13%	5%	0			260	240	730	220	200	TTAATATTTATTTCCATGATAGCATCATTCCAGCCA					17%		10%			
PLSCR3-NM_001369421.1eU185-5'UTR-73	0					2	7%	2%	0			300	160	790	80	380	CAGAACTAAGCCACCCAGACACCATCATCTCGAAAA						6%	9%			
PLXNA1-NM_032242.4eU6746-3'UTR+809	0					2	6%	0%	0			370	180	750	250	350	AGGGGCCCCGGGTCGCAGAGAGCACGTTCCCGTTAT					6%		6%			
PMS2-NM_000535.7eU2687-3'UTR+68	0					3	32%	7%	3	34%	5%	470	160	740	220	470	ATTTTTATGTTTTGAAAGACAGAGTCTTCACTAACC					26%	31%	40%	36%	29%	38%
POU2F2-NM_001207025.4eU5695-3'UTR+4220	4	37%	19%	40%	22%	3	38%	8%	1	35%		390	80	460	280	180	GACCTGACTAGTTTTTCTAACCCACATGAACACCAG	40%	19%	63%	27%	38%	47%	31%	35%		
PPP2R3B-NM_013239.5eU1286-NP_037371.2aa357SL	0					3	24%	1%	0			310	150	910	100	420	TTTCTACCAAGATGATAGACAGGATCTTCTCAGGAG					25%	22%	24%			
PPP2R5C-NM_001161725.2eU1123-NP_001155197.1aa313TM	0					2	2%	0%	0			280	160	690	210	390	TGCAGTTTTTAGAAAAGGACAGCACCCTCACGGAAC					3%		2%			
PPP6R3-NM_001352354.2eU135-5'UTR-227	0					2	7%	1%	0			490	100	650	370	370	TGACTTGCTCAAATAGTGACAGAACTTCCTCGGCCA					8%	7%				
PREPL-NM_001042385.2eU829-NP_001035844.1aa265LL	0					2	14%	4%	3	55%	8%	380	180	470	530	310	CCTTTATCTTACAAAAGACAGTCGTTTCCTCACCAT					11%	17%		48%	54%	63%
PRKCA-NM_002737.3eU6655-3'UTR+4397	0					3	9%	2%	0			290	250	610	420	310	AAAGGTGATACCATCTGACAGTCATTTTCTCACGTT					10%	6%	10%			
PRKCI-NM_002740.6eU493-NP_002731.4aa83Q*	0					2	5%	2%	0			250	110	720	120	360	GAGGAAGGAGACCCGTGTACAGTATCATCTCAGTTG					4%		6%			
PRMT6-NM_018137.3eU2095-3'UTR+925	0					2	9%	1%	0			290	410	780	480	540	AAACTCACTTAATTAAGGAAAATATTTTCCCAGCTA					10%		8%			
PRR11-NM_018304.4eU376-NP_060774.2aa104PL	0					2	4%	2%	0			320	240	800	320	320	GTTTAGAAGTATTGAAAGACACCATCTTTCCATCTC						5%	2%			
PSMD9-NM_001261400.3eU461-3'UTR+37	1	2%		2%		3	10%	1%	0			290	260	860	410	300	CTGGGGAACAGTAACAGGAAAGCATCTTCCCTTGCC		2%			9%	10%	11%			
PSMD9-NM_002813.7eU776-3'UTR+37	1	23%		23%		3	92%	2%	0			290	260	860	410	300	CTGGGGAACAGTAACAGGAAAGCATCTTCCCTTGCC			23%		91%	91%	94%			
PSME2-NM_002818.3eU104-NP_002809.2aa25FF	0					3	9%	1%	0			470	320	790	220	280	ACAGGTGGAGGTCTTCAGACAGAATCTTTTCCAGGA					8%	10%	10%			
PSME2-XM_006720213.2eU140-5'UTR-282	0					2	8%	1%	0			470	320	790	220	280	ACAGGTGGAGGTCTTCAGACAGAATCTTTTCCAGGA					7%		8%			
PSMG4-NM_001128591.2eU390-NP_001122063.1aa105AV	0					3	19%	5%	0			250	190	810	100	410	ATAACCTTCAGAACACAGACAGTAACTTCGCATTAC					24%	14%	21%			
PSPC1-NM_001042414.4eU595-NP_001035879.1aa178Q*	0					2	3%	1%	0			270	250	860	220	470	TCCAATGAGCTGCTAGAGCAAGCATTTTCTCAGTTT					2%		4%			
PTPN11-NM_001330437.2eU2201-3'UTR+242	0					2	2%	0%	0			370	230	780	340	290	ATTTCCTCAGATAAGAAGAAATCATCTCTACAATGT					2%	2%				
PXN-XM_024449107.1eU557-5'UTR-308	0					3	13%	3%	0			270	120	750	160	370	GTGTCTCTGTGCCCCAGGACTGCATCCTCTCACCAA					17%	13%	10%			
PYGB-NM_002862.4eU3978-3'UTR+1352	0					3	11%	5%	0			250	330	820	200	300	GGCTGTGGTCGTGCCCTGACAGCATCTTCCCCAGGC					4%	15%	12%			
RAB11FIP5-NM_001371272.1eU5971-3'UTR+1825	0					2	6%	1%	0			430	250	750	230	250	CATTGGGTATTAGAGAAGTAAGTATCTTTTCTGAGA						6%	5%			
RAB35-NM_001167606.2eU204-NP_001161078.1aa34SL	3	4%	1%	4%	1%	3	32%	5%	0			390	260	800	220	450	TACTGTTGCGTTTTGCAGACAACACTTTCTCAGGCA		5%	4%	3%	36%	35%	26%			
RAB35-NM_006861.7eU204-NP_006852.1aa34SL	1	11%		11%		3	51%	9%	0			390	260	800	220	450	TACTGTTGCGTTTTGCAGACAACACTTTCTCAGGCA	11%				45%	47%	61%			
RAB35-XM_024448801.1eU920-5'UTR-381	0					3	42%	7%	0			390	260	800	220	450	TACTGTTGCGTTTTGCAGACAACACTTTCTCAGGCA					40%	50%	36%			
RAB9A-NM_004251.5eU236-5'UTR-42	1	3%		3%		3	8%	3%	0			310	120	820	100	340	CTTTTCCCGTGTCGTTTGAGTGCATCTTCTCAACAA			3%		8%	11%	4%			
RABGEF1-NR_104676.2eU225-ncr	0					2	9%	3%	0			340	110	690	250	290	CCTGGGCAACATAGCAAGACTGCATCTCTACAAAAA					12%		7%			
RAC1-NM_006908.5eU1514-3'UTR+726	0					3	2%	1%	0			300	220	750	210	490	TGTGATCAAAGGACAAAGACAGTATTTTGACAAAAT					3%	1%	2%			
RACK1-NM_006098.5eU204-NP_006089.1aa33SF	0					3	3%	0%	0			250	300	710	120	370	CTACCCCGCAGTTCCCGGACATGATCCTCTCCGCCT					2%	3%	3%			
RAD21-NM_006265.3eU335-NP_006256.1aa46SL	0					3	13%	1%	0			430	110	800	120	400	ATTTAGAGAGCAGCGTGGAGAGTATCATCTCACCAA					15%	12%	12%			
RALY-NM_007367.4eU441-NP_031393.2aa43SF	4	1%	0%	1%	0%	3	5%	0%	0			480	160	820	220	340	TGAAGAAATCAGATGTGGAGACCATCTTCTCTAAGT	1%	2%	1%	1%	5%	6%	5%			
RALY-NM_016732.3eU441-NP_057951.1aa43SF	1	1%		1%		3	4%	1%	0			480	160	820	220	340	TGAAGAAATCAGATGTGGAGACCATCTTCTCTAAGT		1%			3%	5%	5%			
RASSF1-NM_001206957.2eU1237-3'UTR+290	1	1%		1%		3	14%	2%	3	32%	5%	410	260	480	540	290	CAGCAGGAGTGGTGCAGACAGAAGTCTCCTCAATTT		1%			12%	16%	13%	30%	28%	38%
RBM39-NM_001242599.2eU2397-3'UTR+537	4	49%	16%	57%	4%	3	9%	1%	3	23%	4%	300	180	710	180	260	TCAAAATTATATTTCTTGATGGTATCTTTTCAATTA	53%	61%	56%	26%	8%	10%	8%	28%	22%	21%
RCC2-NM_001136204.3eU1479-NP_001129676.1aa486SL	4	31%	10%	36%	3%	3	5%	1%	3	21%	1%	280	130	770	290	360	AGGAGGTAAAGACTCTGGATGGCATTTTCTCAGAGC	39%	32%	36%	17%	5%	6%	3%	20%	21%	22%
REV3L-NM_001286431.2eU7485-NP_001273360.1aa2188Q*	0					2	10%	4%	0			450	270	890	270	350	GCAGTAAATACCCCACAGAAAGAAACTTCTCAGATT					13%		7%			
RNASEH1-NM_001286834.3eU714-NP_001273763.1aa188TM	0					3	7%	2%	0			320	220	700	140	430	AACTGGTTCTGTATACAGACAGTATGTTTACGATAA					6%	9%	7%			
RNASEH1-NR_148533.2eU710-ncr	0					2	21%	2%	0			320	220	700	140	430	AACTGGTTCTGTATACAGACAGTATGTTTACGATAA						23%	19%			
RNF167-NM_001320356.2eU922-NP_001307285.1aa169PL	0					2	2%	0%	0			330	370	720	250	450	GGGTGCTTCTGGTTCCAGACAATACCTTCCCCTTGG					2%		2%			
RNF6-NM_005977.4eU2910-3'UTR+439	4	22%	10%	26%	7%	3	76%	3%	0			380	170	800	260	300	TAACTTTTTATTTTAAGGACAGTATCTTTTCATGAA	34%	24%	20%	11%	73%	79%	77%			
ROCK2-NM_001321643.2eU1082-NP_001308572.1aa267FF	0					3	21%	5%	0			360	220	760	80	350	TGGGGTGGAAGAAATCAGACAGCATCCTTTCTTTAA					21%	26%	15%			
RPL8-NM_000973.5eU515-NP_000964.1aa160SL	4	5%	2%	6%	1%	3	0%	0%	3	2%	0%	360	40	600	410	440	TGCCCTCCGGCTCCAAGAAGGTTATCTCCTCAGCCA	7%	6%	6%	3%	0%	0%	0%	2%	2%	2%
RPLP1-NM_001003.3eU1-5'UTR-132	2	4%	5%	0%		2	0%	0%	1	25%		250	220	220	260	120	CACATAAGAGGCTGCGTATAGGCGCGAGAGCCCCTT	0%			8%		0%	0%	25%		
RPS26-NM_001029.5eU635-3'UTR+38	0					3	2%	0%	0			430	190	730	140	430	AGTTCTTAAAGACTGAAGACAGGCTATTCTCTGGAG					1%	2%	2%			
RPS4X-NM_001007.5eU182-NP_000998.1aa43PP	1	0%		0%		3	2%	1%	3	10%	2%	420	210	680	270	520	CGGTCCCCACAAGTTGAGAGAGTGTCTCCCCCTCAT	0%				1%	2%	1%	10%	9%	12%
SAP130-NM_001145928.2eU1389-NP_001139400.1aa416HY	0					3	12%	1%	0			230	230	700	230	360	AGTACCATTGTTACCATGACAGTACCCTCCCATTCC					12%	11%	13%			
SCCPDH-NM_016002.3eU941-NP_057086.2aa309LL	4	50%	15%	57%	3%	3	6%	1%	3	14%	2%	280	230	800	310	230	TGTGAGGTTTGGAATTGGAAGGCAACTTCTCATAAA	59%	60%	53%	27%	7%	5%	7%	13%	16%	13%
SCHIP1-NM_001197107.2eU2324-3'UTR+322	2	2%	0%	2%	0%	3	13%	1%	0			250	280	700	430	540	TAGAGATTTTAAGTTGGAACAGTATTTTCCCATTGA		2%	2%		13%	14%	12%			
SCO1-NM_004589.4eU690-NP_004580.1aa222PS	0					2	3%	1%	0			550	350	810	340	440	GCCATCGCAAATTATGTGAAAGAATTTTCTCCCAAA					2%		4%			
SEC61A1-NM_013336.4eU2124-3'UTR+577	0					3	6%	2%	0			610	310	580	530	190	AAAGTGATTTTTTTAAGGACAGAACTTCTTCCAAAA					4%	6%	7%			
SELENOT-NM_016275.5eU307-NP_057359.2aa89LL	0					3	2%	1%	0			360	240	710	240	320	CAACCAATATATAGACACATAGCATCTTTCCTGTCA					1%	4%	2%			
SEMA3C-NM_001350120.2eU233-NP_001337049.1aa42Q*	0					3	14%	11%	0			250	300	870	290	380	ATTTGTTCTATCTGTGTGAAAGGATCTTCCCAGCCC					27%	8%	6%			
SEMA3C-NM_001350121.2eU332-5'UTR-194	0					3	20%	7%	0			250	300	870	290	380	ATTTGTTCTATCTGTGTGAAAGGATCTTCCCAGCCC					25%	12%	22%			
SEMA3C-NM_006379.5eU332-NP_006370.1aa24Q*	0					3	20%	11%	0			250	300	870	290	380	ATTTGTTCTATCTGTGTGAAAGGATCTTCCCAGCCC					8%	21%	31%			
SERPINB8-NM_001276490.2eU1590-3'UTR+543	0					3	16%	6%	0			350	110	810	250	310	ACACAGTTTGTAATGAAGACAGCTCCTTCTCATCTT					14%	11%	23%			
SERPINE1-NM_000602.5eU2530-3'UTR+1179	0					3	5%	1%	0			430	390	670	520	290	GCTGCATGACCTACCAGGACAGAACTTTCCCCAATT					5%	6%	6%			
SERPINH1-NM_001207014.3eU1728-3'UTR+326	4	3%	1%	3%	0%	2	1%	0%	0			330	150	600	360	200	AGTATTCATATTTATAGCCAGGTACCTTCTCACCTG	3%	3%	3%	2%		0%	1%			
SFXN1-NM_001322977.2eU2944-3'UTR+1858	0					3	7%	1%	0			420	60	710	320	400	TTCTGACAATGTGACCAGACTGAATTTCCTCATAAA					6%	7%	7%			
SLC19A1-NM_001205206.4eU1293-NP_001192135.1aa391LL	3	24%	15%	32%	6%	3	43%	10%	0			270	70	810	100	380	CCCATCGCCACCTTTCAGATTGCATCTTCTCTGTCT		36%	28%	8%	32%	52%	45%			
SLC19A1-NM_001205207.3eU1201-NP_001192136.1aa351LL	4	20%	10%	24%	7%	3	38%	7%	0			270	70	810	100	380	CCCATCGCCACCTTTCAGATTGCATCTTCTCTGTCT	21%	18%	32%	7%	32%	45%	36%			
SLC25A26-NM_001350991.1eU182-NP_001337920.1aa27LL	0					2	12%	4%	0			400	100	670	240	380	GGTGTTTCTGTTGACTTGATATTATTTCCTCTGGAT					10%	15%				
SLC5A6-NM_021095.4eU339-5'UTR-119	0					2	6%	1%	0			310	170	580	330	220	CCACGGAGCTCACCCTGGACAGTATCACTCCGTGGA					6%		5%			

SMAD4-NM_005359.6eU812-NP_005350.1aa92HY	0					2	4%	2%	0			360	220	800	410	320	CTTCAGGTGGCTGGTCGGAAAGGATTTCCTCATGTG					5%		3%			
SPARC-NM_001309443.2eU3038-3'UTR+2065	4	31%	11%	36%	5%	3	1%	0%	3	2%	1%	360	130	620	380	400	ACATACCTAGATCTCCAGATGTCATTTCCCCTCTCT	41%	31%	36%	16%	1%	2%	2%	1%	2%	3%
SPATS2L-NM_001100422.1eU2338-3'UTR+249	0					3	2%	1%	0			270	180	720	130	370	TTGCAGCATTTTAGCCAGGCAGTATTTACTCATTAT					2%	3%	2%			
SPDL1-NM_001329639.2eU622-NP_001316568.1aa161SL	0					3	4%	1%	0			480	180	770	420	160	TGTCTGAACGTGTGCAGGAAAGCATGTCTTCAGAGA					5%	3%	4%			
SPDYE2-XM_017012223.1eU4800-3'UTR+3107	2	43%	24%	60%		2	45%	46%	2	27%	7%	250	370	410	230	610	TGGGTGTCCTCAGACCACATGTACTTCTCACCAACT	60%			26%	13%		78%	22%		32%
SRGAP2B-NM_001271870.4eU2515-3'UTR+487	0					2	2%	0%	0			470	360	700	420	400	ATAACTTTTATTGTTGGGAAACTATTTTTTCTAATT					2%		2%			
SRGN-NM_001321053.2eU451-NP_001307982.1aa88PL	2	1%	0%	1%	0%	2	3%	1%	0			270	180	660	360	440	CGAGAATCCAGGACTTGAATCGTATCTTCCCACTTT	1%		1%		3%		4%			
SRSF7-NM_001031684.3eU586-NP_001026854.1aa161R*	0					3	5%	1%	0			320	90	720	250	210	AGGGGACGAAGGTCAAGGTCAGCATCTCCTCGACGA					6%	6%	5%			
SRSF7-NM_001195446.2eU586-NP_001182375.1aa161R*	0					2	6%	2%	0			320	90	720	250	210	AGGGGACGAAGGTCAAGGTCAGCATCTCCTCGACGA						7%	4%			
SRSF7-NM_001363802.1eU719-NP_001350731.1aa161R*	0					2	6%	1%	0			320	90	720	250	210	AGGGGACGAAGGTCAAGGTCAGCATCTCCTCGACGA					6%	7%				
STAT5B-NM_012448.4eU235-NP_036580.2aa27PP	0					2	18%	10%	0			210	240	740	310	310	GATGCAAGCGTTATATGGCCAGCATTTTCCCATTGA					11%	25%				
SUPT5H-NM_001111020.3eU172-NP_001104490.1aa10SF	3	2%	0%	1%	0%	3	19%	3%	0			430	350	730	260	130	AGATGTCGGACAGCGAGGGACAGCAACTTTTCCGAGG		1%	1%	2%	15%	21%	19%			
SUPT5H-NM_001111020.3eU173-NP_001104490.1aa10SS	0					3	3%	1%	0			330	430	350	730	260	GATGTCGGACAGCGAGGACAGCAACTTTTCCGAGGA					2%	2%	4%			
SUPT5H-NM_001130825.2eU172-NP_001124297.1aa10SF	0					3	22%	7%	0			430	350	730	260	130	AGATGTCGGACAGCGAGGGACAGCAACTTTTCCGAGG					15%	21%	29%			
SUPT5H-NM_003169.4eU276-NP_003160.2aa10SF	2	2%	1%	3%		3	19%	3%	0			430	350	730	260	130	AGATGTCGGACAGCGAGGGACAGCAACTTTTCCGAGG	3%			2%	16%	22%	21%			
SUPT5H-NM_003169.4eU277-NP_003160.2aa10SS	0					2	3%	1%	0			330	430	350	730	260	GATGTCGGACAGCGAGGACAGCAACTTTTCCGAGGA						4%	2%			
SUZ12P1-NR_024187.2eU481-ncr	0					2	19%	7%	0			320	300	670	370	310	GGATGTATGTTGTCCAATAAGGCAAGTTCCCACAGA					13%		24%			
SYNJ1-NM_001160302.1eU6402-3'UTR+2389	4	28%	14%	35%	7%	2	6%	2%	3	16%	3%	360	150	770	310	420	AAAATACTATATTTATAGAAGTAATCTTCTCTGTTA	30%	31%	43%	8%	4%	8%		13%	17%	17%
TACO1-NM_016360.4eU434-NP_057444.2aa82SF	1	3%		3%		2	4%	0%	0			240	340	610	220	410	CGAAGGACGTCGAAAGGAGTCGCATCTTCTCCAAAC		3%				3%	4%			
TASOR-NM_001112736.2eU599-NP_001106207.1aa114Q*	0					3	31%	3%	0			360	380	770	490	310	AGGAAGAGCAGAGAAAAGAAAGCACTTTTCCAGCCA					29%	30%	34%			
TBX3-NM_005996.4eU3992-3'UTR+845	0					2	4%	1%	0			390	170	630	280	350	GATTCATGTCGTTCTTTGACAGTTCTTTCTCTTTCC						4%	3%			
TCFL5-NM_006602.4eU1930-3'UTR+275	0					3	19%	6%	0			270	300	860	220	350	TTAATTAGTAATAATTTAGTAAGCATTTTCTCAATGC					14%	26%	17%			
TEAD1-NM_021961.6eU4475-3'UTR+2573	0					2	7%	1%	0			210	410	480	520	570	CTAAAGCTTTCTCCAGGACAGATATTTTCCCTGTCT					6%		8%			
TEAD1-NM_021961.6eU5185-3'UTR+3283	0					3	10%	4%	0			410	300	730	400	240	CTTTCATTTTACCCTGTGAAAGCATCTCTCCTAGAG					5%	11%	13%			
TIMM22-NM_013337.4eU715-3'UTR+116	0					2	3%	0%	0			320	320	520	520	350	TGCTTCAGGGCCTGAAGACATTCATTTTCCCTCATG					4%		3%			
TIMP3-NM_000362.5eU1002-3'UTR+69	1	2%		2%		2	6%	1%	0			350	270	580	280	590	GCTGAGCTTCCCTTGGACACTAACTCTTCCCAGATG			2%		5%		7%			
TLCD5-NM_001198670.2eU3637-3'UTR+2509	0					2	25%	18%	0			340	40	760	170	310	GGGCATTGAGATAATCTGACAGCATCTCCACTTCCT						38%	12%			
TMED2-NM_001321445.2eU1730-3'UTR+1018	1	1%		1%		3	8%	1%	0			390	250	710	350	390	CTTGTTTTGTGTAAAATGAAATTACTTTCTCTTGTT	1%				6%	8%	9%			
TMEM104-NM_001321264.3eU443-NP_001308193.1aa99LL	0					3	8%	4%	0			190	230	570	430	250	CTCCTCCACAGCCTCAGACAGCGATGTTCTCATCCG					5%	12%	7%			
TMEM263-NM_001319661.2eU1136-3'UTR+620	0					3	8%	1%	0			510	390	830	270	400	ATTGATGCCTTACAAAAGAAAACATCTTTTCTAATA					8%	7%	9%			
TMEM65-NM_194291.3eU2273-3'UTR+1006	4	54%	17%	61%	8%	3	12%	6%	3	30%	12%	230	70	820	180	420	TTTTTTTTCCCTCCAAAGATGTCATCTTCTCATCTG	61%	53%	70%	31%	6%	13%	18%	21%	43%	26%
TMEM87B-NM_001329914.2eU4508-3'UTR+2517	0					2	11%	6%	0			390	180	780	320	300	TTTTAAGCCTGAGAGATGATAGAATGTTCCCATATT					7%		15%			
TMTC3-NM_001366574.1eU6993-3'UTR+4170	0					3	49%	2%	0			400	240	750	320	240	ATTTCTGTGACTCAATGGATAGCATATTTCCATCAA					47%	50%	50%			
TMX1-NM_030755.5eU3647-3'UTR+2758	0					2	18%	7%	0			510	250	600	620	400	AGTAAATGATGCTTTAGAAAGCAATTTTCTCTCATC					23%		13%			
TNC-NM_002160.4eU5468-NP_002151.2aa1719SL	4	34%	8%	38%	4%	3	3%	1%	3	13%	1%	280	210	770	420	400	CCATGGGCTCCCCAAAGGAAGTCATTTTCTCAGACA	42%	38%	35%	23%	3%	3%	4%	13%	13%	12%
TNFRSF19-NM_001204458.3eU1215-NP_001191387.1aa337LL	0					3	14%	6%	0			130	350	540	360	420	TGAACTCACTGGAGAAGACATTCATTCTCTCAATCC					7%	18%	18%			
TOPBP1-NM_001363889.2eU5157-3'UTR+423	1	5%		5%		3	3%	1%	0			290	110	580	220	310	TATTCATGTTTTCCCTGATTTTTATCTCTCACCAT		5%			3%	4%	3%			
TOR1AIP1-NM_001267578.2eU711-NP_001254507.1aa165Q*	3	7%	2%	9%	1%	2	7%	5%	0			260	390	790	250	370	CATTCCTCTGAAGAGGATGAAGCATCTTCCCAAACT		9%	8%	5%	4%	10%				
TPD52-NM_001025252.3eU1220-3'UTR+168	0					2	7%	1%	0			290	380	780	230	530	TGTGGGTTAAACAAAATGAAAATATCTTCACCTCTA					6%		7%			
TPGS2-NM_001271949.2eU2137-3'UTR+1104	0					2	18%	1%	0			250	320	860	360	440	AAGTTGCCTGTAATTCAGAAATCATTTTCCCATTGA					17%	19%				
TPST2-NM_001008566.3eU1393-NP_001008566.1aa372SF	0					2	7%	2%	0			550	180	660	420	230	ATTTTCAGGTGAACCAGAACAGCACCTCCTCCCACT					6%		9%			
TPX2-NM_012112.5eU1068-NP_036244.2aa187PS	4	3%	1%	4%	2%	3	9%	2%	0			170	370	810	330	420	CAAGATACTGCTGAAAAGAATGCATCTTCCCCAGAG	3%	2%	5%	2%	7%	9%	11%			
TRAM1-NM_001317804.2eU888-NP_001304733.1aa221LL	3	1%	0%	1%	0%	3	15%	1%	0			350	290	850	260	440	AGCAATGAAAAGTATCAGAAAGGATTTTCTCTGTGG	2%	1%	1%		13%	14%	16%			
TRIM16-NM_001348119.1eU1904-NP_001335048.1aa297PL	0					3	5%	1%	0			330	230	760	290	310	AGTTTAAGAACACTGAAGACATCACCTTCCCTAGTG					4%	5%	6%			
TRIM32-NM_001099679.2eU2620-3'UTR+503	0					3	11%	6%	0			380	180	770	240	380	CTTTCAGTGACATTTAAGACATCATATTCCCGTAAC					8%	8%	18%			
TSPAN2-NM_001308315.2eU1816-3'UTR+1154	0					2	17%	8%	0			360	330	820	390	380	GCCAAACCAAAATAAAGAAAAGCATCTTCTCCTAGT						22%	11%			
UBB-NM_001281716.2eU377-NP_001268645.1aa38PL	4	28%	7%	31%	1%	3	1%	0%	3	4%	0%	210	360	590	530	430	CCAAGATCCAGGATAAGGAAGGCATTCCCCCGACC	32%	32%	30%	18%	1%	1%	1%	4%	4%	4%
UBB-NM_001281716.2eU605-NP_001268645.1aa114PL	4	21%	6%	24%	3%	3	1%	0%	3	4%	0%	280	360	610	420	350	CCAAGATCCAGGATAAAGAAGGCATCCCTCCGACC	27%	25%	21%	13%	1%	2%	1%	4%	4%	4%
UBB-NM_001281716.2eU833-NP_001268645.1aa190PL	4	46%	13%	52%	3%	3	4%	0%	3	12%	1%	210	300	680	380	450	CCAAGATCCAAGATAAAGAAGGCATCCCCCCGACC	55%	53%	49%	27%	4%	4%	4%	11%	12%	12%
UBB-NM_018955.4eU213-NP_061828.1aa38PL	4	27%	6%	30%	0%	3	1%	0%	3	3%	0%	210	360	590	530	430	CCAAGATCCAGGATAAGGAAGGCATTCCCCCGACC	30%	30%	31%	18%	1%	1%	1%	4%	3%	4%
UBC-NM_021009.7eU1320-NP_066289.3aa418PL	4	5%	1%	5%	0%	3	0%	0%	3	1%	0%	320	260	570	500	270	CAAAGATCCAAGACAAGGAAGGCATCCCTCCTGACC	6%	5%	5%	3%	0%	1%	0%	1%	1%	1%
UBC-NM_021009.7eU1548-NP_066289.3aa494PL	4	4%	1%	4%	1%	2	0%	0%	3	1%	0%	320	260	570	500	270	CAAAGATCCAAGACAAGGAAGGCATCCCTCCTGACC	5%	4%	4%	3%	0%	0%		1%	1%	1%
UBC-NM_021009.7eU180-NP_066289.3aa38PL	4	17%	3%	19%	1%	3	1%	0%	3	3%	0%	320	260	570	500	270	CAAAGATCCAAGATAAGGAAGGCATCCCTCCTGACC	19%	19%	18%	12%	1%	1%	1%	2%	3%	3%
UBC-NM_021009.7eU494-NP_066289.3aa143LL	0					2	1%	0%	3	3%	0%	420	190	720	370	340	TCTGACTACAACATCCAGAAAGAGTCTACCCTGCAC						1%	1%	3%	3%	3%
UBE2B-NM_003337.4eU770-3'UTR+284	0					2	3%	1%	0			420	40	720	220	310	TGGTGTGGAGTTTTCATGACAGAATATACACATTTT					3%		4%			
UBE2G2-NM_001202489.2eU1481-3'UTR+993	0					3	11%	4%	0			250	290	690	330	540	TGCTGATCACAAGGACAGACAGTTTTTCCCGACCA					7%	15%	10%			
UBE2H-NM_001202498.2eU1547-3'UTR+969	0					2	3%	1%	0			220	170	730	100	430	TGCTTAGAAAGGTTGCAGATTTCATCTTCACCTACC						3%	4%			
UBE2R2-NM_017811.4eU1512-3'UTR+197	0					2	2%	0%	0			540	300	610	250	460	TGGACCTTTAATGGAGAGAGAGTAACCTCCACAGA					2%	3%				
UFL1-NM_015323.5eU2493-3'UTR+40	0					3	7%	1%	0			360	420	770	320	390	TTTACATTTGTCATATAGTAAGCATTTTCCCCCAAG					8%	5%	7%			
UHMK1-NM_001184763.1eU7901-3'UTR+6786	4	18%	6%	21%	2%	2	4%	1%	2	2%	0%	280	300	690	420	540	ATGTGTGTCAGTACCAAAAAGTTATTTTCTCAAATC	23%	20%	20%	9%	3%	5%		2%	2%	
UPF3B-NM_023010.4eU2111-3'UTR+663	0					3	3%	1%	0			380	260	630	180	150	AAACTTCGTGTTTTGTCATCAGCATCTTTTCTATTA					3%	4%	3%			
USP10-NM_001272075.2eU730-NP_001259004.1aa177TI	0					3	35%	3%	0			350	40	770	210	370	TGAAAGATGGTGGCGATGATAGTATCTCCACAGAAG					38%	32%	36%			
VDAC2-NM_001184783.3eU987-NP_001171712.1aa130SL	1	3%		3%		3	10%	2%	0			450	110	770	190	290	GTTTGAAACTGACATTTGATACTACCTCTCACCAA	3%				9%	11%	12%			
VPS13A-NM_001018037.2eU4465-NP_001018047.1aa1432R*	0					2	12%	9%	0			410	250	820	300	270	AAAAGACCTCATGTCAAGAAAGCAACTCCTCGAATG						18%	6%			
WASHC5-NM_001330609.2eU1899-NP_001317538.1aa488TI	0					2	4%	0%	0			320	250	830	230	330	TTTTGCAGATCATCCCAGAAAGCATGTTTACATCTC					4%		3%			
WDR76-NM_001167941.2eU1316-NP_001161413.1aa401II	0					3	5%	1%	0			410	170	790	170	440	TGTCCACCCAGTGCATAGACAGTATTTTATCACTGC					6%	4%	6%			
WWC2-NM_024949.6eU3841-3'UTR+27	1	4%		4%		3	9%	4%	0			380	310	790	340	400	GTGATTACATGACTTAAGAAATTATTTTTTCATCTG			4%		7%	5%	14%			
XPO6-NM_001270940.2eU791-NP_001257869.1aa6HY	0					3	8%	2%	0			570	230	750	330	260	GCATTGGAAAGTCTGATGACAGAATTTTTTCACGAT					9%	9%	7%			
XPO6-NM_015171.4eU655-NP_055986.1aa20HY	0					3	8%	2%	0			570	230	750	330	260	GCATTGGAAAGTCTGATGACAGAATTTTTTCACGAT					6%	9%	7%			
YARS2-NM_001040436.3eU1388-NP_001035526.1aa455LL	0					3	6%	2%	0			430	250	820	180	380	GAGTGTTTTAATTGTTGGACAACATATTCTCAAGAA					5%	4%	8%			
ZBTB21-NM_001098402.2eU1274-NP_001091872.1aa363Q*	0					2	4%	1%	0			260	230	580	280	490	TATTCACCTTCCATAGATTTGAAATCTTCCCAGGGA					3%	4%				
ZC3HC1-NM_001282190.2eU874-NP_001269119.1aa253Q*	0					3	6%	1%	0			290	250	780	100	380	TCCATGCAGCTCTCCCTGATAACATGTTCGCAATGT					7%	6%	5%			
ZFYVE26-NM_015346.4eU1879-NP_056161.2aa580SL	0					2	26%	18%	0			350	280	910	270	380	TGTGCCTGGAGCTTCTGGAAAACATCTTCTCATTGC					13%	38%				
ZMYM6-NM_007167.4eU542-NP_009098.3aa113Q*	0					2	18%	6%	0			310	80	850	140	340	CAAACTGCATATCATAAGACAGGATCTACTCAGCTC					14%		22%			
ZNF10-NM_015394.5eU665-NP_056209.2aa160Q*	0					2	32%	12%	0			350	180	780	350	430	GTGGCATTCACCCAAAAGAAAGTACTTACTCAGGAG					40%		23%			
ZNF224-NM_001321645.3eU772-NP_001308574.1aa149Q*	0					2	41%	8%	0			260	350	580	570	370	TCTGTAATTCACACAAGACAGAAATCTTCCCAGGGC						47%	35%			
ZNF335-NM_022095.4eU4282-3'UTR+121	0					3	7%	4%	0			210	160	800	0	400	TCTCCAGATACTGGATAGCCAGCATCCTCTCATTCC					4%	11%	7%			
ZNF473-NM_001006656.4eU2238-NP_001006657.1aa684TI	0					3	5%	0%	2	6%	3%	530	210	700	180	430	TTAAATGTAGTAAGTGTGACAGAGTCTTCACCCAGA					6%	5%	5%	4%		8%
ZNF598-NM_178167.4eU3339-3'UTR+550	0					2	8%	1%	0			560	150	650	310	300	CCTCTGACCTTTTATAAGACAGTTTATCTTCAAATA					9%	8%				
ZNF609-NM_015042.2eU2418-NP_055857.1aa677TI	0					3	17%	5%	0			170	190	560	480	230	AAATCTACACCTTCCAGACAGCCACCTTCACAGCAG					11%	20%	20%			
ZNF680-NM_178558.5eU1904-3'UTR+194	0					2	13%	6%	0			360	310	740	420	310	CACATCTTATTGCACAGGAAAGCATTTTTACTTGAG						9%	17%			
ZNHIT1-NM_006349.3eU564-3'UTR+68	0					3	4%	2%	0			550	100	730	360	340	TGCCCGGCCTTCAGAAAGACAGAATTTCATCACCCA					7%	4%	3%			

C) PPR56_S4TN>TD

>total: 2265

Offtarget<br>blue > same position in gene	Rep. 56-N	%CU 56-N-4	STABW 56-N-4	%CU 56-N-3	STABW 56-N-3	Rep. 56-S7	%CU 56 S7	STABW 56-S7	Rep. 56-S4	%CU 56-S4	STABW 56-S4	PPR56 S4 Scoring -2	-1	Match	+1	+2	Binding region	%editing in individual replicates<br>PPR56-native<br>%IMR1	%IMR2	%IMR3	%ELIM1	PPR56-S7mut<br>%I6-2	%I7	%I8	PPR56-S4mut<br>%I1-2	%I3	%I4
AACS-NM_001319839.2eU1183-NP_001306768.1aa345LL	0					0			2	7%	1%	370	200	620	270	280	TGGATGATGTGGAACTGGATGGTGTCCCTTCTGGCC									7%	6%
AACS-NM_001319839.2eU1688-NP_001306768.1aa513SF	0					0			3	7%	3%	450	360	510	640	400	ACGGCAACAAGTACAGGAAGGCGTATTTCTCCAAAT								5%	10%	7%
AACS-NM_001319839.2eU1911-NP_001306768.1aa587LL	0					0			3	9%	4%	320	430	710	260	510	GAAAAGCCGTGGAGCAAGGAGGTGCTTTCTCGAACC								6%	8%	14%
AACS-NM_001319839.2eU371-NP_001306768.1aa74SL	0					0			2	5%	1%	410	270	700	60	480	AGTTCTGGAAATTCAGTGGAATTGTCTTCTCACGTG								5%	6%	
AACS-NM_001319840.2eU1991-NP_001306769.1aa614SL	0					0			3	11%	4%	320	430	710	260	510	GAAAAGCCGTGGAGCAAGGAGGTGCTTTCTCGAACC								10%	8%	15%
AAGAB-NM_001271885.2eU279-5'UTR-39	0					0			3	4%	1%	400	130	720	170	310	CACAAAAATCGGGCCTTGATAGTGTCTCCTCATGGC								4%	5%	3%
AAGAB-NM_001271885.2eU450-NP_001258814.1aa45PL	0					0			3	35%	7%	300	140	730	360	200	AGGAGTTGCCTGAGGAGGATGATGACTTCCCAGAAT								43%	29%	34%
AAGAB-NM_001271885.2eU981-3'UTR+44	0					0			3	6%	4%	380	170	350	410	360	GGGTTTGACCAACAAAGATGCTAGCTGTCTCTGAGA								3%	7%	10%
AARS2-NM_020745.4eU1046-NP_065796.2aa341PL	1	8%		8%		0			3	23%	6%	210	230	740	240	290	TCAGTGTCTGCATCTCTGATGGCATCTTCCCTGGGA		8%						22%	17%	30%
AASS-NM_005763.4eU3385-3'UTR+500	0					0			3	11%	3%	350	260	690	460	410	TTGTATTTTTCAGTAGAGACGGGGTTTCACCATGTT								8%	10%	14%
AATF-NM_012138.4eU1067-NP_036270.1aa299Q*	0					0			2	3%	1%	280	460	530	440	450	TTGGTAGGTCTTCAGGAAGAGTTGCTTTTCCAGTAC									2%	3%
ABCB7-NM_001271696.3eU1398-NP_001258625.1aa463LF	4	39%	11%	44%	6%	1	4%		3	7%	3%	260	160	680	310	370	CAAATTAAAGACAAAGTGATGGCATCTCCCCTTCAG	48%	37%	48%	24%		4%		4%	9%	7%
ABCC4-XM_017020319.1eU279-5'UTR-130	0					0			3	47%	18%	390	70	740	260	320	CCACTTGGATAATCCAGGATGATGTCTTCACTCCAA								31%	44%	67%
ABHD16A-NM_001177515.2eU707-NP_001170986.1aa173Q*	0					0			3	10%	4%	360	150	780	270	180	CTCCTCAACCGGGTTAAGAAGCTGCCTTGTCAGATC								9%	7%	14%
ABHD16A-NM_021160.3eU335-NP_066983.1aa98HY	0					0			3	13%	3%	330	210	740	210	480	TTGAGTTTGTCCAAAGTGGTGCCGTTTTCTCACTAT								11%	11%	17%
ABHD2-NM_007011.8eU1943-3'UTR+53	0					0			3	12%	6%	570	150	610	480	160	CCAGCAGCCCTCCTCTGGAAGCTGCGTCCCCTCACC								11%	18%	6%
ABHD3-NM_001308256.2eU996-NP_001295185.1aa296SL	4	8%	3%	9%	2%	0			3	67%	3%	410	130	780	290	330	TGTGTCTAAATTCTGTGGATGATGTTTTCTCACCCA	10%	7%	11%	4%				70%	64%	66%
ABI1-NM_001012750.3eU2833-3'UTR+1292	0					0			3	9%	2%	390	250	570	420	500	AATTGAAGGGAGGGCTGAGGGATGTTTTCTCTTCTT								11%	8%	8%
ABI2-NM_001282925.3eU4740-3'UTR+3077	0					0			3	7%	1%	340	250	630	220	370	CCCCTTAAAAAAAATGAATAGTTGTCTTTTCTTGTC								6%	8%	5%
ABL1-NM_005157.6eU1966-NP_005148.2aa591LL	0					0			3	14%	2%	530	340	550	400	340	GGGCGGCCTGAATGAAGATGAGCGCCTTCTCCCCAA								15%	14%	12%
ACACA-NM_198834.3eU6329-NP_942131.1aa1931TI	0					0			3	3%	0%	340	340	770	290	400	CTGTGTGTGATGACTTTGAAGGGGTTTTCACTGTCC								3%	4%	3%
ACAD8-NM_014384.3eU2021-3'UTR+740	0					0			2	14%	5%	310	320	530	400	260	TTTTTGTTTCCTTTTCTGAAGCTGTTCCTCCTTTTA									10%	17%
ACAD9-NM_014049.5eU236-NP_054768.2aa55PL	1	3%		3%		2	8%	1%	3	38%	1%	250	320	700	510	350	TAGGCAAAATCAAGAAGAAAGAAGTTTTCCCATTTC		3%			8%	9%		38%	39%	39%
ACAP2-NM_012287.6eU3062-3'UTR+552	0					0			3	13%	3%	210	470	720	270	610	GATTTATTAGAACTAGAGAATGTGTTTTCCCCGTTT								11%	16%	12%
ACBD5-NM_001042473.4eU3025-3'UTR+1409	0					0			3	36%	10%	420	320	690	500	410	TTTGTATTTTTAGTAGAGACGGGGTTTCTCCATGTT								25%	44%	38%
ACBD7-NM_001039844.3eU1154-3'UTR+839	0					0			2	24%	9%	300	330	750	390	450	TTTGTATTTTTAGTAGAGACGGGGTTTTGCCATGTT									18%	31%
ACIN1-NM_001164814.1eU545-NP_001158286.1aa73Q*	0					0			3	5%	1%	510	140	520	350	290	GAGGAGGTGACTCTGGACGGGAAGCCTCTTCAGGCG								4%	5%	6%
ACO1-NM_001278352.2eU3162-3'UTR+305	1	4%		4%		0			3	63%	3%	480	190	650	420	160	AAAGTTACTGATCACAGGACGTTGCTTTTTCACTGT		4%						65%	60%	65%
ACSL3-NM_001354158.2eU2373-NP_001341087.1aa668SL	0					0			2	3%	1%	320	270	620	280	160	TACTTAAAGTGCTTTCCGAAGCTGCTATTTCAGCAA								2%		4%
ACSS2-NM_001076552.3eU667-NP_001070020.2aa211LL	0					0			3	18%	12%	230	70	790	140	290	ACGGATCTTGGATTCCAGCTGCAGTCTTCTCATCAC								31%	10%	12%
ACTR10-NM_018477.3eU1239-NP_060947.1aa390LL	0					0			3	11%	1%	170	360	590	270	520	GACGGGCCGTATACCTGATTGGTGTTCTCTCAATAA								11%	13%	10%
ACTR2-NM_001005386.3eU850-NP_001005386.1aa257II	0					0			3	4%	1%	450	90	530	460	150	ATCTTATACACTCCCAGATGGACGTATCATCAAAGT								5%	3%	4%
ACVR2A-NM_001278579.2eU2632-3'UTR+821	0					0			2	16%	3%	380	320	720	160	370	GTGCTGTTTTTTGAAAAGATGGTGTCATTTCCCCCT								14%	18%	
ADAM17-NM_001382777.1eU241-5'UTR-666	0					0			2	3%	1%	450	180	610	520	110	AGCGGGGCCGGGAACATGAGGCAGTCTCTCCTATTC								2%		3%
ADAR-NM_001025107.3eU4034-3'UTR+251	4	36%	13%	42%	2%	2	2%	0%	3	2%	0%	340	210	710	220	250	AAGAGGCAGAAACCTAAGAAGTTATCTTTTCTTTCT	45%	41%	40%	16%	1%		2%	3%	2%	2%
ADGRG2-NM_001079858.3eU3007-NP_001073327.1aa978HH	0					0			3	40%	13%	290	250	470	400	410	TAGTGTTCAGAATGGAGATGTGTGCCTTCACGATTT								35%	29%	55%
ADIPOR1-NM_001290553.2eU180-NP_001277482.1aa4HY	0					0			3	18%	3%	360	220	670	320	270	GGGTATTGAGGTACCAGCCAGATGTCTTCCCACAAA								19%	21%	15%
ADIPOR1-NM_015999.6eU232-NP_057083.2aa4HY	0					0			3	20%	2%	360	220	670	320	270	GGGTATTGAGGTACCAGCCAGATGTCTTCCCACAAA								19%	22%	20%
ADNP-NM_001282531.3eU3788-NP_001269460.1aa971PL	0					0			3	26%	7%	370	280	630	510	320	ATGTTGTTGAGTGGAAAGACGGTGCTTCTCCATCTG								17%	30%	29%
ADSL-NM_000026.4eU1461-NP_000017.1aa468LL	0					0			2	4%	1%	260	320	670	270	600	CAGAGATTCTTAGAAGAGGAGGTGTATCCCCTGTTA									4%	5%
AFDN-NM_001207008.2eU5995-3'UTR+541	0					0			3	10%	4%	490	250	690	180	210	CTCCTCCCATGTCCCCTGATGGTGTCTGTTCCCACA								14%	8%	9%
AFF1-NM_001166693.3eU1314-NP_001160165.1aa350Q*	0					0			3	50%	25%	350	150	810	190	240	GCCAAGCTCACCAAACTGAAGATGCCTTCTCAGTCA								24%	50%	75%
AFF4-NM_014423.4eU466-NP_055238.1aa29PL	0					0			3	7%	1%	390	220	810	290	220	AGGAAATTCAGCAGGGCGAAGACGCCTTCCCACCTA								8%	7%	5%
AFMID-NM_001010982.5eU1467-3'UTR+546	0					0			2	9%	5%	400	190	690	360	460	TTTGTATTTTTAGTAGAGACGGGGTTTCAACATATT								5%		13%
AGBL5-NM_001035507.3eU163-5'UTR-11	0					0			2	8%	2%	370	200	680	570	300	GGGCCAGAGCGGGGCAGGAGGATGCTTTCCCAGCCC								6%	10%	
AGFG1-NM_001135187.2eU1863-NP_001128659.1aa533Q*	1	9%		9%		3	54%	19%	3	10%	3%	310	310	730	230	470	GCAGCTTTCCCTCAACAGACAGCTTTTTCTCAACAG		9%			37%	50%	75%	7%	9%	14%
AGFG1-NM_001135189.2eU1671-NP_001128661.1aa469Q*	4	5%	2%	6%	1%	3	51%	7%	2	4%	1%	310	310	730	230	470	GCAGCTTTCCCTCAACAGACAGCTTTTTCTCAACAG	7%	6%	4%	3%	51%	57%	43%	4%		3%
AGFG2-NM_006076.5eU550-NP_006067.3aa136Q*	0					0			3	17%	5%	330	360	690	420	330	AGGGATCCTCAGAAAGTGAAGGAGTTTCTCCAGGAA								15%	23%	14%
AGO2-NM_001164623.3eU3513-3'UTR+908	0					0			3	46%	8%	340	190	750	290	350	TATTAATGTGATAGATAGATGCTGTTTTTTCATTTT								46%	39%	55%
AGO2-NM_001164623.3eU4471-3'UTR+1866	0					0			3	24%	5%	440	80	760	140	310	GGATGACTATTATTTGTGAAGATGTGTACTCAGAGT								23%	21%	30%
AHCTF1-NM_001323342.2eU4097-NP_001310271.1aa1240PL	0					0			3	9%	5%	240	250	740	220	380	GAATTTCATTTGTGGAAGAAGATGTCCACCCAAAAT								5%	6%	15%
AHCTF1-NM_001323342.2eU696-NP_001310271.1aa106LL	0					0			3	41%	5%	470	200	690	430	370	AGGATTGGAAGAAACAGAAGGGAGTGTTCTCTGTCT								36%	40%	46%
AHCYL1-NM_001242673.2eU1988-NP_001229602.1aa396LL	0					0			3	16%	3%	310	170	650	390	340	AGTTGTCCTCCTGGCAGAGGGTCGTCTACTCAATTT								14%	13%	19%
AHI1-NM_001134830.2eU428-NP_001128302.1aa72PP	4	35%	16%	40%	17%	0			2	6%	1%	170	370	740	280	290	TGATCCCGACACTATTAGAAGCAATCTTCCCCATAT	38%	57%	24%	23%				6%		7%
AHNAK-NM_001346445.2eU6421-NP_001333374.1aa2080SL	0					0			3	13%	1%	210	130	710	150	330	TGAACTTGCCCAAGGCTGATGTTGTTGTCTCAGGAC								13%	14%	11%
AIMP1-NM_001142415.2eU746-NP_001135887.1aa210LF	0					0			3	9%	1%	320	280	600	330	290	GAACAGATGCAAAATCGGATGGTGATTTTACTTTGT								8%	9%	11%
AK1-NM_001318121.1eU691-NP_001305050.1aa184SF	0					3	5%	2%	3	6%	3%	500	300	730	180	350	ACGCTGAGGGCTCCGTGGACAGTGTCTTCTCCCAGG					3%	6%	5%	3%	10%	5%
AK2-NM_001199199.3eU693-NP_001186128.1aa213AV	0					0			3	6%	2%	290	120	780	140	210	ATGCATCCCAGACCCCCGATGTCGTGTTCGCAAGCA								4%	7%	7%
AK2-NM_001319139.3eU835-NP_001306068.1aa173AV	0					0			2	10%	5%	290	120	780	140	210	ATGCATCCCAGACCCCCGATGTCGTGTTCGCAAGCA								7%		14%
AK2-NM_001319140.2eU835-NP_001306069.1aa173AV	0					0			3	13%	3%	290	120	780	140	210	ATGCATCCCAGACCCCCGATGTCGTGTTCGCAAGCA								10%	15%	15%
AK3-NM_001199852.2eU752-3'UTR+73	0					0			3	70%	3%	410	390	640	440	240	CCTAGTCCTTGCATTTAGAAGCTGCTTTTCCTAAGA								68%	70%	74%
AKAP17A-NM_005088.3eU3150-3'UTR+877	0					0			2	13%	2%	340	360	680	290	390	ACGTTCGCGTTAGGAAAGATGGTGTTTATTCCAGTT									14%	12%
AKAP8-NM_005858.4eU1039-NP_005849.1aa328SF	0					0			2	5%	1%	320	400	750	210	400	TGGCCCGGGTTGACAGTGAAGGAGATTTCTCCGAAA								6%		5%
AKAP8-NM_005858.4eU2128-NP_005849.1aa691TI	0					0			3	10%	2%	250	110	650	300	530	CTGATGCAGAGTCTAAAGACGCTGTTCCCACAGAAT								8%	10%	12%
AKIP1-NM_001206646.2eU951-3'UTR+358	0					0			3	14%	3%	350	260	690	460	410	TTTGTATTTTTAGTAGAGACGGGGTTTCACCATGTT								17%	11%	15%
AKIRIN1-NM_001136275.2eU857-3'UTR+308	0					0			2	3%	0%	260	310	680	460	470	TTCAGTGGCATTCAACAAATGGAGTTTCCCCAAGCA								3%	3%	
AKIRIN2-NM_018064.4eU770-NP_060534.1aa74SF	0					0			3	6%	3%	420	180	680	170	260	TGGAGCCATCCCCCTTCGGCGACGTCTCCTCCCGCC								8%	3%	7%
ALDH1A2-NM_001206897.2eU1510-NP_001193826.1aa430TI	0					0			3	15%	4%	330	260	670	180	300	CAGACTTTGGACTCGTAGCAGCTGTCTTTACTAATG								10%	17%	17%
ALDH1A3-NM_000693.4eU1411-NP_000684.2aa445TI	0					0			3	8%	3%	300	200	780	140	380	CCGACTATGGACTCACAGCAGCCGTGTTCACAAAAA								6%	11%	7%
ALDH2-NM_000690.4eU1399-NP_000681.2aa450TI	0					0			3	4%	1%	220	200	750	140	320	CCACGTACGGGCTGGCCGCAGCTGTCTTCACAAAGG								4%	3%	5%
ALDH7A1-NM_001182.5eU1358-NP_001173.2aa446AV	0					0			3	7%	4%	410	250	800	300	190	TTAAATTCAAGAATGAAGAAGAGGTCTTTGCATGGA								3%	10%	10%
ALKBH6-NM_001297701.2eU729-NP_001284630.1aa197SL	0					0			3	24%	13%	460	0	700	380	100	CCCGCGTAGACGCGCTGGACGCCGCCTCCTCGCCGC								20%	14%	39%
ALMS1-NM_001378454.1eU10007-NP_001365383.1aa3325SL	0					0			2	8%	2%	250	120	740	80	470	AGGTGCTAGGCACAAGAGATGATGACCTCTCAGCCA								7%	9%	
ALS2-NM_001135745.2eU109-5'UTR-10	3	7%	2%	8%	2%	0			3	22%	3%	390	230	780	240	370	AGAAGGAATGATTTGGTGATGGAGTGTTCCCACTGA	10%	7%		5%				20%	20%	25%
ALS2-NM_020919.4eU1367-NP_065970.2aa417SL	0					0			3	10%	5%	370	160	700	190	260	TGGCTGCTACTTATGAAGCTGGTGCCTTGTCACTGA								4%	13%	14%

AMACR-NM_001167595.2eU432-NP_001161067.1aa134SL	0					0			2	9%	2%	220	400	630	190	480	TCAACTATTTGGCTTTGTCAGGTGTTCTCTCAAAAA								8%	11%	
AMFR-NM_001144.6eU2041-NP_001135.3aa608SL	0					0			2	2%	1%	470	0	630	240	240	TGAACAAAAGTTCTGAAGATGATGCGGCCTCAGAGA								1%	2%	
AMFR-NM_001144.6eU2074-NP_001135.3aa619SF	0					0			3	12%	1%	530	170	690	340	240	AGAGCTTCCTCCCCTCGGAAGGTGCGTCCTCTGACC								14%	11%	12%
ANAPC11-NM_001002246.2eU136-5'UTR-61	1	4%		4%		0			2	16%	4%	320	150	730	280	370	GCCAACGGAAGGGCGGAGACGGAGTTTCGTCATGTT		4%						19%		13%
ANAPC11-NM_001002248.3eU134-5'UTR-61	0					0			3	21%	9%	320	150	730	280	370	AAGGGCGGGTAGGGCGAGACGGAGTTTCGTCATGTT								12%	29%	24%
ANAPC11-NM_001002249.2eU195-5'UTR-61	0					0			2	31%	1%	320	150	730	280	370	TGCAGACGAGCTGCGGAGACGGAGTTTCGTCATGTT									30%	32%
ANAPC11-NM_016476.11eU166-5'UTR-61	0					0			2	33%	10%	320	150	730	280	410	GATTAGGTTGGCGAAGAGACGGAGTTTCGTCATGTT								40%		26%
ANAPC16-NM_001242546.2eU1263-3'UTR+450	0					0			3	20%	3%	580	260	460	390	250	AAAAAGTTATTTTGCAGATGAATGTGTTTTCAACTC								16%	22%	21%
ANAPC4-NM_001286756.2eU467-NP_001273685.1aa125LL	0					0			2	12%	4%	500	190	740	380	250	GATGGAAGTGACAGTAGAAAGCAGTGTTCTCACATC									15%	9%
ANAPC7-NM_001137664.2eU975-NP_001131136.2aa298FF	0					0			3	14%	4%	380	180	740	350	40	GGATGTTGAGAACCTTGGATGCCGCCTTTTCAATAT								12%	19%	11%
ANKFY1-NM_001257999.3eU5295-3'UTR+1542	0					2	2%	0%	3	8%	2%	520	130	620	250	310	TTGTTCATTCTTTTCTGGATAGTGTGTCCTCTGTAT					2%	3%		6%	8%	10%
ANKLE2-NM_015114.3eU2281-NP_055929.1aa749SS	0					0			3	22%	3%	400	250	610	450	40	GAATTTGCAAAATATAGGACGTAGCGTTTCCAAGAC								24%	18%	23%
ANKLE2-NM_015114.3eU4357-3'UTR+1506	0					0			2	6%	3%	320	340	560	170	580	ATGGATATTCGTAGTGATGATCTGTTTTCTCTAAAA								4%		8%
ANKRD10-XM_024449382.1eU720-3'UTR+82	0					0			3	10%	4%	320	210	710	300	210	CTTGCTGAACAGGTATGGAAGCTGTCTTTGCTGTTA								6%	9%	15%
ANKRD11-NM_001256182.2eU6183-NP_001243111.1aa1884SL	4	5%	1%	6%	1%	0			3	21%	4%	350	210	950	230	410	CTGTCACCCCGTCTCCAGAGGGCGTCTTCTCAAGTT	6%	5%	5%	4%				17%	23%	24%
ANKRD13C-NM_030816.5eU1968-3'UTR+28	0					0			3	14%	5%	210	120	700	400	110	TAACTGACGTGGAAAAGGATGCCGTCTAACCAAGGA								17%	8%	16%
ANKRD13C-NM_030816.5eU2582-3'UTR+642	0					0			3	25%	7%	470	240	750	190	350	TGTTGCAATACATTTCAGAAGGAGCGTTCACTCTGC								26%	17%	31%
ANKRD33B-NM_001164440.2eU2192-3'UTR+309	0					0			3	27%	3%	350	360	770	380	510	TAAAGATCAATTTATCAAAGGGCGTTTTCTCCGTAA								28%	28%	24%
ANLN-NM_001284301.3eU2156-NP_001271230.1aa664SL	0					0			3	7%	1%	420	250	620	380	230	AGGTGATTGTTCGGAAGGAAGATGTTACTTCAAAAC								6%	7%	8%
ANTXR1-NM_032208.3eU2079-3'UTR+63	0					0			2	13%	1%	320	280	710	240	230	AACTTCAGGAGATGTTAGAACAAGTCTTTCCAGTTA									13%	14%
ANTXR1-NM_032208.3eU4813-3'UTR+2797	0					0			3	6%	2%	440	290	400	580	120	CGTATTCCATGTGTTAGATGGAAGCATTTCCTATCC								9%	4%	6%
ANXA7-NM_001156.5eU448-NP_001147.1aa134Q*	0					3	10%	2%	3	20%	3%	310	190	650	310	360	GGTGGCTTTCCTGGAGGACAGATGCCTTCTCAGTAT					11%	7%	11%	16%	22%	22%
AOX1-NM_001159.4eU831-NP_001150.3aa246PS	0					0			3	15%	1%	400	340	690	430	310	TTTGGCAGTGAGAGAATGATGTGGTTTTCCCCCGTG								14%	15%	16%
AP1S2-NM_001272071.2eU710-3'UTR+101	0					0			3	48%	10%	310	70	800	180	250	GTTAGCTCATGGTGTAAGATGATGTCTTGTCAGTAT								58%	38%	49%
AP1S2-NM_003916.5eU701-3'UTR+101	0					0			3	69%	5%	310	70	800	180	250	GTTAGCTCATGGTGTAAGATGATGTCTTGTCAGTAT								65%	68%	74%
AP2B1-NM_001030006.2eU1276-NP_001025177.1aa382II	0					0			3	2%	0%	340	190	620	230	280	AGCTGTGCGGGCCATTGGACGGTGTGCCATCAAGGT								2%	2%	2%
AP2B1-NM_001030006.2eU1720-NP_001025177.1aa530LL	0					0			3	3%	1%	460	200	640	230	260	AGACCGGGGCTATATTTATTGGCGCCTTCTCTCAAC								3%	2%	4%
AP2B1-NM_001030006.2eU1758-NP_001025177.1aa543SF	0					0			3	24%	2%	360	150	790	180	230	ACCCTGTTACAGCTAAAGAAGTAGTCTTGTCTGAGA								25%	22%	25%
AP2B1-NM_001030006.2eU280-NP_001025177.1aa50LL	0					0			3	4%	1%	290	220	410	320	540	TATGACCGTGGGGAAGGATGTTAGTTCTCTCTTTCC								3%	3%	5%
AP2B1-NM_001030006.2eU3843-3'UTR+857	0					0			3	7%	2%	360	50	740	190	380	TGGGGAACAAGAACCCAGATGCTGTCCCCTCACCCC								5%	9%	8%
AP2B1-NM_001030006.2eU990-NP_001025177.1aa287PL	0					0			2	2%	0%	490	220	360	590	190	ACAATATGCTGCTGAAGAAGTTAGCCCCTCCACTTG									2%	2%
AP4E1-NM_001252127.2eU2270-NP_001239056.1aa642PP	4	18%	7%	20%	6%	0			2	15%	6%	250	230	630	570	390	GAAATTGTGGGGGAAAGAAGGCTATCTTCCCAAGAA	14%	20%	26%	12%					11%	19%
APH1A-NM_001077628.3eU604-NP_001071096.1aa133SF	0					0			3	4%	2%	270	280	660	180	520	TCTCCTTCGGTATCATCAGTGGTGTCTTCTCTGTTA								3%	4%	6%
APOBEC3C-NM_014508.3eU322-NP_055323.2aa72LL	0					0			3	10%	2%	500	270	610	530	340	GACCCATTGTCATGCAGAAAGGTGCTTCCTCTCTTG								8%	13%	10%
APTX-NM_001195248.2eU1018-NP_001182177.2aa318RC	0					0			2	5%	0%	370	190	560	280	130	GGGATGCCTGAGCTCTTGAAGCTGCCCCTTCGTTGT								5%	5%	
AQP11-NM_173039.3eU738-NP_766627.1aa233PL	0					0			2	34%	12%	480	140	630	290	140	TACATTTCATGTGTTTTGATGAAGCATTCCCTCAGT								42%		25%
AQR-NM_014691.3eU545-NP_055506.1aa139SL	0					0			3	11%	2%	440	220	690	180	220	CGGCATTAGCTGAAACTGATGGTGAATTTTCACTTC								11%	12%	9%
ARF1-NM_001024226.2eU1661-3'UTR+935	0					0			3	6%	0%	450	130	670	260	280	ACCCCACCTCTGTGTGTGATGTAGCTTTCTCTCCCT								5%	6%	6%
ARFGEF2-NM_006420.3eU1519-NP_006411.2aa447SL	0					0			2	8%	1%	390	190	740	370	310	GTGTGGCCTTGTCCAAAAACGGCGTCTCTTCAGTGC								8%	7%	
ARFIP1-NM_001025593.3eU2226-3'UTR+918	0					0			2	14%	2%	320	280	600	310	320	TAAGCCTTAAATCACAGATATGTGCCTTCTCAGATA								16%		13%
ARHGAP17-NM_001006634.3eU1839-NP_001006635.1aa577PP	0					0			3	4%	1%	240	280	560	430	360	GGGGCCGAGCCCAGGCGACGGCAGTCCTCCCAAACC								3%	4%	5%
ARHGAP29-NM_001328664.2eU417-NP_001315593.1aa68SL	4	41%	12%	47%	2%	0			3	4%	1%	350	210	750	260	230	TGTTACTATATTTGAAAGAAGCCATATTTTCAGACT	45%	49%	48%	24%				3%	4%	5%
ARHGAP5-NM_001030055.2eU167-5'UTR-135	0					0			3	15%	5%	520	200	650	320	190	ATGATCCCTATGATCTTGAAGATGTTTCTGCACAGA								21%	11%	14%
ARHGEF12-NM_001198665.2eU8950-3'UTR+3541	0					0			3	25%	2%	370	330	600	580	220	AAAAAAGTACCTCCTAGAAGGAAGCCTTCCCCACCA								23%	26%	26%
ARHGEF9-NM_001173479.2eU2934-3'UTR+1473	0					0			2	19%	9%	440	290	630	610	230	TGTTCTGGCACTCAAAGAAGGCCGTGTTTCCAACAT								12%		25%
ARID5B-NM_001244638.2eU2734-NP_001231567.1aa871PS	0					0			3	4%	1%	310	370	660	220	420	CAGTACTTGAAAAACCAGACTGTGCTTTCTCCACTC								4%	5%	3%
ARL5A-NM_001037174.2eU696-3'UTR+167	0					0			2	6%	1%	340	70	650	350	190	ACTTATTTGAAAACAAAGATGAAGTCTCACCTTCCA								6%	5%	
ARMC10-NM_001161009.3eU591-NP_001154481.1aa153SF	0					0			3	16%	9%	430	70	780	230	390	ACATCAGTCAAGTATGTGAGGATGTCTTCTCTGGTC								5%	20%	22%
ARPC1A-NM_001190996.2eU453-NP_001177925.1aa93LL	0					0			3	3%	1%	330	290	650	330	320	CGCGCAGCTACTTTTGTGAAGTGGTCCCCCCTAGAG								2%	4%	4%
ASB16-NM_080863.5eU1914-3'UTR+466	2	22%	11%	30%		0			3	25%	4%	220	110	490	200	120	GGCTTCTTGAGACACCTGCTGACCTCTAGCCGGACC			30%	14%				23%	29%	23%
ASCC1-NM_001198798.2eU1491-3'UTR+110	0					0			3	45%	10%	320	290	650	360	180	AGGGATGTGGACATTTGGATGGTGACTTTCCTGGGT								40%	57%	39%
ASMTL-NM_001173473.2eU1161-NP_001166944.1aa314SF	0					0			2	3%	1%	410	90	690	220	180	ACGTCTACCTGGCATCGGATGGCGAATACTCTCTGC								4%		2%
ASNS-NM_001178075.2eU408-NP_001171546.1aa101AV	0					0			3	27%	7%	360	220	710	260	250	AAACAATTTGTATGTTGGATGGTGTGTTTGCATTTG								20%	30%	33%
ASPM-NM_018136.5eU8974-NP_060606.3aa2914II	0					0			2	5%	0%	350	150	790	180	190	AGTCTATTTACAGATCAGAAGCAGTGTTATCATTAT								5%		4%
ASXL2-NM_001369346.1eU1662-NP_001356275.1aa514LF	0					0			2	9%	3%	520	140	780	370	330	AGCCCAGAAAGCCTCAAGAGGAAGTCTTCCCTCACC								11%	7%	
ATAD1-NM_001321967.2eU1207-3'UTR+48	0					0			3	6%	2%	450	90	550	290	310	ACAGTTCAGTGATCTAGTTTGGTGTGTCCTCTTATC								8%	5%	4%
ATAD2-NM_014109.4eU103-NP_054828.2aa5RC	0					0			2	2%	0%	480	360	580	460	280	GGCCAGTAGGGAGAGAAGATGGTGGTTCTCCGCAGC								1%		2%
ATAD2-NM_014109.4eU1424-NP_054828.2aa445PL	0					0			3	20%	1%	490	280	510	610	190	TAGCAGCTCTAAAAGAGATGGTGGTGTTTCCATTAC								18%	21%	20%
ATAD5-NM_024857.5eU5489-NP_079133.3aa1703Q*	0					0			2	5%	1%	530	280	560	230	430	AGTCTTGAGAGTAATGATGGATGGACTTCTCAAAGC									5%	4%
ATAD5-NM_024857.5eU5913-NP_079133.3aa1844PL	0					0			3	5%	1%	260	220	640	320	200	AGACTGTGAATACTTTGGCAGCTGACTTCCCTTAAT								5%	4%	6%
ATF4-NM_001675.4eU40-5'UTR-849	0					0			2	33%	15%	310	130	770	210	390	TACTTTGCCCGCCCACAGATGTAGTTTTCTCTGCGC								23%		43%
ATF4-NM_182810.3eU37-5'UTR-247	0					0			3	30%	2%	310	130	770	210	390	TACTTTGCCCGCCCACAGATGTAGTTTTCTCTGCGC								32%	30%	28%
ATF6B-NM_001136153.2eU1672-NP_001129625.1aa545PS	0					0			3	5%	2%	300	230	690	230	210	CAGAAGTCTCAGCCACGGAAGAAGTCACCTCCAGTT								3%	7%	6%
ATG14-NM_014924.5eU986-NP_055739.2aa326PP	4	28%	14%	34%	11%	0			2	7%	1%	300	230	460	410	290	TCTGTCTCATATACTTGATGTAAATCTTCCCAAAAA	45%	24%	32%	12%					7%	6%
ATG5-NM_001286106.2eU833-NP_001273035.1aa205PS	0					0			3	11%	2%	320	240	680	320	270	GAAAGACCTTTCATTCAGAAGCTGTTTCGTCCTGTG								10%	9%	13%
ATIC-NM_004044.7eU1139-NP_004035.2aa352TI	0					0			3	6%	2%	290	200	740	230	170	TTGCCCCAGGATATGAAGAAGAAGCCTTGACAATAC								4%	8%	7%
ATL3-NM_001290048.2eU2586-3'UTR+667	0					0			2	10%	2%	350	260	690	460	410	TTTGTATTTTTAGTAGAGATGGGGTTTCACCATGTT								12%		9%
ATMIN-NM_001300728.2eU2182-NP_001287657.1aa591TI	0					0			3	10%	2%	300	170	760	210	340	ACACAGAGACCCAAACTGAAGGAGTCTCCACTGCTA								13%	8%	9%
ATM-NM_000051.4eU8144-NP_000042.3aa2665PL	0					0			3	11%	4%	190	250	660	290	410	AACTTAAGAATTTAGAAGATGTTGTTGTCCCTACTA								8%	9%	16%
ATP13A3-NM_001367549.1eU2192-NP_001354478.1aa556SF	0					0			3	17%	2%	330	290	740	80	400	ATTCACTTACAAAAATTGAAGGAGTGCTCTCTGGTG								15%	18%	17%
ATP13A3-NM_001367549.1eU5338-3'UTR+1042	0					0			3	10%	3%	350	240	670	330	210	AATACTGGATTTGAATGGCTGCCGTTTTTTCAAAGC								6%	12%	12%
ATP13A3-NM_001367549.1eU6514-3'UTR+2218	0					0			3	6%	1%	410	160	770	100	410	ACTCAGTGCCATAGATAGACTGAGTCTTCTCTCGTA								4%	7%	7%
ATP2B1-NM_001001323.2eU4041-3'UTR+141	0					0			2	11%	7%	400	220	520	350	320	ATGACACTGATGCCGAAGATGATGCTCCTACAAAAC								16%	6%	
ATP2B4-NM_001001396.3eU3818-NP_001001396.1aa989SL	0					0			3	20%	7%	540	210	630	430	190	GAAAGATCCATGGAGAGAAGAACGTCTTTTCAGGCA								13%	20%	27%
ATP5MC2-NM_001002031.4eU624-3'UTR+12	0					0			3	5%	1%	250	390	730	130	300	CATCCTCTTTGCCATGTGAAGGAGCCGTCTCCACCT								3%	5%	6%
ATP5PD-NM_001003785.2eU404-NP_001003785.1aa117PL	0					0			3	12%	1%	340	240	600	520	240	TGACCATTGAGGACTTGAATGAAGCTTTCCCAGAAA								12%	11%	13%
ATP5PD-NM_006356.3eU476-NP_006347.1aa141PL	0					0			3	12%	1%	340	240	600	520	240	TGACCATTGAGGACTTGAATGAAGCTTTCCCAGAAA								12%	13%	11%
ATP6AP1-NM_001183.6eU1168-NP_001174.2aa384LL	0					0			3	5%	1%	360	120	600	340	540	CAGCAGCCTGAGCAAGAAGGGTAGTCTCCTCGTGGC								7%	4%	5%
ATP6AP1-NM_001183.6eU369-NP_001174.2aa118SF	0					0			2	4%	0%	550	310	640	310	120	TGTTTGGAAACAAGCAGGACAGCGCCTTTTCTAACC								4%		4%
ATP6V0A1-NM_001130020.3eU1524-NP_001123492.1aa464SF	0					0			3	11%	3%	390	350	610	350	430	GATACATTATTTTATTGATGGGTGTGTTCTCCATGT								12%	13%	8%
ATP6V0B-NM_001039457.3eU168-5'UTR-7	0					0			3	6%	2%	310	260	720	140	220	GCTGGCCGTGGGTTCCTGACGGAGACTTCGCCCTTC								9%	4%	6%
ATP6V0B-NM_001294333.2eU217-NP_001281262.1aa46PS	0					0			2	2%	1%	310	260	720	140	220	GATGTGGCATGGTTCCTGACGGAGACTTCGCCCTTC								2%		1%
ATP6V1C1-NM_001695.5eU2052-3'UTR+721	0					0			3	20%	7%	380	210	740	180	100	GGTCAGAACACAAATTTGAAGATGACTTTTCAGTAT								15%	28%	17%

ATP6V1D-NM_015994.4eU309-NP_057078.1aa68SL	4	8%	3%	9%	1%	0	3	70%	1%	480	210	780	270	300	TGGGCGAAGTGATGAGAGAAGCTGCCTTTTCACTAG	10%	9%	8%	3%	70%	71%	69%
ATP6V1H-NM_015941.4eU291-NP_057025.2aa15PL	0					0	2	6%	1%	190	350	560	420	260	ATATCCGAGGTGCTGTGGATGCTGCTGTCCCCACCA						7%	6%
ATPAF2-NM_145691.4eU306-NP_663729.1aa51Q*	0					0	2	6%	3%	450	250	750	410	210	GCCCCGCCGACAGAAAGGAAGAGGTTTTATCAGAAT					8%		4%
ATPSCKMT-NM_001258388.2eU707-3'UTR+41	0					0	2	10%	0%	260	180	580	320	290	GAGTGTTTTTCTGAAATATTGTAGTCTTCCCTGATC					10%		10%
ATRN-NM_001207047.3eU2795-NP_001193976.1aa914Q*	0					0	3	10%	5%	290	200	770	190	320	TCCTATAAAGGACCAGTGAAGATGCCTTCGCAAGCC					6%	16%	9%
ATXN10-NM_001167621.2eU1129-NP_001161093.1aa300SF	0					0	3	14%	2%	260	390	770	80	360	GTGGTTGCGTGAGAGCAGAAGGTGACATCTCCAATG					13%	16%	14%
AVL9-NM_015060.3eU666-NP_055875.1aa149Q*	4	23%	9%	27%	3%	0	3	19%	2%	220	420	780	370	550	CATGCATATTTTGAAGAGAAGGATTTTTCCCAAATT	30%	28%	23%	10%	20%	16%	20%
AZIN1-NM_001301668.3eU333-5'UTR-259	0					0	3	4%	2%	340	380	680	360	300	AATTCCGCCGAAAAAGAGAAGACGCTTTACCCGACT					3%	3%	6%
AZIN1-NM_001301668.3eU61-5'UTR-531	0					0	3	4%	1%	310	70	680	150	140	TTTTCCTTTTTTCTTCTGCCGTCGCCTTCTCTGCCT					3%	3%	5%
B3GNT2-NM_001319075.2eU1365-3'UTR+64	0					0	2	3%	0%	500	70	700	140	270	GTATTTTGAATAGTTCCCATGTTGTGTTCTCACATT					3%		3%
B3GNT2-NM_001319075.2eU997-NP_001306004.1aa297SF	0					0	3	36%	7%	310	140	750	210	370	TGAAGTACTACATCCCAGAAGTTGTTTACTCTGGCC					29%	38%	43%
B4GALT1-NM_001378495.1eU163-NP_001365424.1aa45TI	0					0	2	7%	0%	320	90	690	250	300	GCCGCCTGCCCCAACTGGTCGGAGTCTCCACACCGC						7%	7%
B4GALT1-NM_001378495.1eU3478-3'UTR+2291	0					0	2	8%	0%	290	120	710	80	280	TGCTCCCCCCATTTGCTGACGTAGTGCTCTCATGGG						8%	8%
BAG3-NM_004281.4eU997-NP_004272.2aa234PL	0					0	2	1%	0%	420	230	420	610	230	CCTTCCACCAAGCCCAGAAGACGCACTACCCAGCGC					2%	1%	
BAIAP2-DT-NR_026857.1eU1953-ncr	0					0	3	12%	1%	410	350	700	170	310	GGGAGTTTTGCTAAACAGAAGGAGCCCTTTCTGAGG					12%	11%	13%
BAIAP2-NM_001144888.2eU396-NP_001138360.1aa98HY	0					0	3	7%	3%	490	130	680	180	150	AATCAGCTGGAAGAAATGCTGAAGTCTTTTCACAAC					4%	8%	9%
BAMBI-NM_012342.3eU1172-3'UTR+12	0					0	3	17%	5%	360	160	750	180	190	GAAGCTGGAATTCGTATGACGGAGTCTTATCTGAAC					11%	19%	21%
BAP1-NM_004656.4eU2872-3'UTR+552	1	3%		3%		0	3	6%	1%	500	230	570	220	540	TGTCCCTGTTGTAAGGAAGCCAGGTCTTCTCTCTTC	3%				8%	5%	6%
BASP1-NM_001271606.2eU968-3'UTR+200	0					0	3	14%	6%	260	100	630	270	200	CCCAAGGAAAAATACAGGATGTTGTCCCATCAAGGG					12%	9%	20%
BAZ1A-NM_013448.3eU4064-NP_038476.2aa1261PP	0					0	2	4%	1%	380	160	500	580	220	CTCTCAAGAAGAGGAAGAAGTCAGCCTACCCAAACG						3%	4%
BAZ2A-NM_001300905.2eU1715-NP_001287834.1aa504PL	0					0	2	5%	1%	280	330	660	330	180	CTCCCGTAACTTCCCCAGCAGCTGCCTTTCCAACAG					5%	6%	
BAZ2A-NM_001300905.2eU1863-NP_001287834.1aa553PP	0					0	3	7%	3%	290	280	610	490	330	TATTGCTACCCCAGAAGAAGTTCGTCTTCCCCTCCA					9%	4%	9%
BAZ2A-NM_001300905.2eU7717-3'UTR+1801	0					0	3	8%	2%	420	250	650	310	80	CCTCATAGCCTATTTCTAAAGTCGCCTTTTCTGTCA					8%	7%	10%
BCAM-NM_001013257.2eU774-NP_001013275.1aa236HH	0					0	2	3%	0%	380	100	320	580	330	CCGCAAGGATGACCGAGACGCCAGCTTCCACTGCGC					2%	3%	
BCAR1-NM_001170714.3eU3413-3'UTR+378	0					0	3	3%	0%	360	190	550	290	310	TGACAGGGCCAGTGCAGTTTGGTGTGTCCTCCGCCT					3%	3%	3%
BCKDHB-NM_001318975.1eU2844-3'UTR+1787	0					0	2	36%	23%	540	110	480	350	390	GGACCATTAGCCCACAGATGCATGTATTCTCTCAGA					19%		52%
BCL10-NM_001320715.2eU1283-3'UTR+334	0					0	3	5%	3%	540	370	530	460	370	AATAGACCCTTACTAGGAAGAACGTTTTTTCCTCAG					8%	6%	3%
BCL10-NM_001320715.2eU1886-3'UTR+937	0					0	3	20%	1%	210	290	750	310	510	TGTATTATTTTTGTAGAGACGGTGTTTTGCCATGTT					19%	22%	21%
BCL2L12-NM_001040668.1eU1096-NP_001035758.1aa138PP	4	4%	1%	4%	2%	0	3	60%	5%	290	220	780	240	270	CTTCCTGAGCCGCCTTCGAAGATGTCTTCCCTGCTC	6%	3%	4%	3%	66%	58%	56%
BCL2L12-NM_001282516.1eU1099-NP_001269445.1aa139PP	3	5%	1%	5%	1%	0	3	64%	3%	290	220	780	240	270	CTTCCTGAGCCGCCTTCGAAGATGTCTTCCCTGCTC	5%	4%	4%		67%	64%	61%
BCL2L1-NM_001191.4eU1318-3'UTR+424	0					0	2	2%	0%	430	40	400	320	310	AGATTGCCTTTGTTTTGATGTTTGTGGCCTCAGAAT					3%	2%	
BCL7C-NM_004765.4eU750-NP_004756.2aa206PL	0					0	3	6%	2%	420	210	640	470	350	GTGACACAGAGGACTCGGAGGGTGCCCCCCCACTCA					4%	9%	5%
BCL9-NM_004326.4eU3770-NP_004317.2aa1040PL	0					0	3	19%	4%	440	70	700	310	180	AGACTGTGGCCAGCTCAGATGACGACTCCCCTCCAG					19%	15%	23%
BCORL1-NM_001184772.3eU5967-3'UTR+130	0					0	3	45%	12%	310	200	820	270	120	ATAATACGGACCAACAAGAAGCCGCCTTATCAATGC					44%	33%	57%
BECN1-NM_001313998.2eU1804-3'UTR+321	0					0	3	3%	1%	520	170	550	340	210	AACTGAGTTGAATTGAGATTGATGTGTTTTCACTGG					2%	3%	3%
BEND7-NM_001378149.1eU2360-3'UTR+589	0					0	2	26%	6%	340	300	740	270	460	TCATTGTCCATTATAAAGAAGTGGTTCTCTCTTCCC					30%		21%
BICD1-NM_001003398.3eU1245-NP_001003398.1aa254Q*	0					0	2	12%	6%	190	420	690	380	350	CAAAAGAACAACCTGCGGAAGGAGCTCTCCCAGTAT						7%	16%
BIN1-NM_001320632.2eU1458-NP_001307561.1aa416PL	0					0	3	11%	3%	220	410	660	200	460	AGGAGCTGGAGAAGTGCCGTGGCGTCTTCCCCGAGA					9%	10%	15%
BIN1-NM_001320632.2eU1716-3'UTR+230	0					0	3	5%	3%	400	50	630	250	310	TCACCAGTGTTCCTGAAGCTGCTGTGTCCTCTAGTT					4%	8%	4%
BLCAP-NM_001167820.2eU1472-3'UTR+815	0					0	3	8%	5%	340	160	650	260	440	GCTCTGCAGCATTGAAGTATGGTGTGTTCACGTAGT					5%	13%	5%
BLMH-NM_000386.4eU1294-NP_000377.1aa390TI	4	10%	4%	12%	2%	0	3	63%	1%	300	270	720	380	390	CAGAGAAGGATGATCAGGATGGTGCTTTCACAAAAT	14%	9%	12%	5%	62%	62%	64%
BLOC1S6-NM_001311255.1eU330-NP_001298184.1aa64HY	0					0	2	13%	4%	430	290	510	350	630	GTGGAGCAACTGGCAGAAGGATTGCTTTCTCATTAT						10%	16%
BLOC1S6-NM_012388.4eU240-NP_036520.1aa59HY	0					0	3	15%	4%	430	290	510	350	630	GTGGAGCAACTGGCAGAAGGATTGCTTTCTCATTAT					19%	11%	14%
BMS1-NM_014753.4eU704-NP_055568.3aa212PL	1	3%		3%		0	3	37%	2%	290	170	470	520	390	TAAAACACAGGTTCTGGACGGAAGTTTACCCGGGTG	3%				39%	36%	37%
BNC1-NM_001301206.2eU4688-3'UTR+1479	0					0	2	5%	0%	220	230	540	320	330	TTACCCAGCTGTTTAATATTCCAGTCTTCCCAAAGT					5%		5%
BNIP1-NM_001205.3eU97-NP_001196.2aa28Q*	0					0	3	14%	1%	380	290	710	300	220	AAATTTGACCTGGAGGTGAAGGCGCTTATTCAGGAT					15%	13%	13%
BNIP1-NM_013979.3eU97-NP_053582.2aa28Q*	0					0	3	26%	9%	380	290	710	300	220	AAATTTGACCTGGAGGTGAAGGCGCTTATTCAGGAT					21%	20%	37%
BNIP3L-NM_001330491.2eU1866-3'UTR+1201	0					0	3	7%	3%	300	210	720	220	170	ACTGGTCATTTTCTCATGTAGCTGTCTTTTCAGTTA					7%	5%	10%
BNIP3L-NM_004331.3eU87-NP_004322.1aa4HY	0					0	2	11%	3%	370	180	630	140	270	AGTCCTGCCAGCTGTCCGACAATGTCGTCCCACCTA					9%	13%	
BNIP3-NM_004052.4eU1380-3'UTR+678	4	38%	10%	43%	5%	0	3	7%	3%	270	170	750	190	180	ATGATCAGTTTTCTGCTGAAGGCACCTACTCAGTAT	48%	38%	42%	26%	4%	6%	10%
BOP1-NM_015201.5eU250-NP_056016.1aa61SL	0					0	2	2%	0%	430	280	770	220	350	GCGTCTCCGACAGCGAGGAAAGTGTGTTCTCAGGCC					3%		2%
BPNT1-NM_001286149.2eU1760-3'UTR+850	0					0	2	17%	10%	260	300	680	380	470	TTTGTATTTTTAGTAGAGAAGGTGTTTCACCGTGTT					10%	24%	
BRCA1-NM_007294.4eU1529-NP_009225.1aa472PP	0					0	3	10%	2%	380	240	460	450	510	GAAAACCTATCGGAAGAAGGCAAGCCTCCCCAACTT					9%	12%	8%
BRD3-NM_007371.4eU2580-3'UTR+204	0					0	3	12%	5%	330	170	720	80	470	TGCGTTAGACTTGATGAGAAGGTGTGAACTCTGCAG					11%	7%	17%
BRF2-NM_018310.4eU685-NP_060780.2aa203R*	0					0	3	17%	11%	340	260	680	200	460	TACGTGGAAGACAAAGAGAAGATGCTGTCTCGAACA					12%	10%	29%
BSDC1-NM_001143888.3eU148-NP_001137360.1aa41Q*	0					0	3	36%	25%	210	220	720	310	310	TTTATGAAGCGGGACCTGACGGAGTTTACCCAGGTG					15%	30%	64%
BSDC1-NM_001143888.3eU523-NP_001137360.1aa166Q*	0					0	2	3%	0%	300	320	470	360	490	CCCCCGGAATTGTTTGACGCCTGGCTTTCCCAGTTC					3%		4%
BSDC1-NM_018045.8eU148-NP_060515.3aa41Q*	0					0	3	27%	7%	210	220	720	310	310	TTTATGAAGCGGGACCTGACGGAGTTTACCCAGGTG					20%	32%	29%
BTAF1-NM_003972.3eU2162-NP_003963.1aa619HY	0					0	3	18%	10%	310	70	770	270	140	GCTTGGCTTTGCTTGATGATGCAGCCTTCTCATTTA					14%	29%	9%
BTBD6-NM_001387567.1eU639-NP_001374496.1aa154HY	0					0	3	11%	5%	340	210	640	350	310	CCCCCGGGGGCGACCAGGACGGTGCCCGCCCACAAG					6%	10%	15%
BUB1B-NM_001211.6eU1876-NP_001202.5aa575PL	0					0	3	25%	5%	500	210	650	390	170	AAAGCATCACCTCAAATGAAGATGTGTCTCCAGATG					21%	31%	24%
BUD31-XM_005250671.5eU259-5'UTR-272	0					0	3	7%	2%	290	170	680	170	220	TTGCTGAGGACACCTAAGATGACGCAATCTCCGCGC					6%	9%	6%
C11orf49-NM_001003676.3eU1834-3'UTR+964	0					0	2	21%	0%	290	210	710	210	390	ATATGAAGGGTGGGCCAGAAGATGATTTCACTTGCA					21%		21%
C12orf75-NM_001145199.2eU752-3'UTR+359	1	1%		1%		0	3	6%	1%	440	210	630	100	460	TCAGCACATTTCATGGAGGTCATGTCTTTTCACTGA		1%			5%	6%	7%
C16orf95-NM_001195124.3eU977-3'UTR+107	0					0	2	36%	0%	280	70	790	140	390	AGGAGGCGTCTGTGACAGTCGCTGTCTTCTCAGAAC						36%	36%
C17orf80-NM_001100621.3eU2517-3'UTR+625	0					0	3	33%	2%	310	260	820	210	370	GGGTTGGGAAAATAATTGAAGATGTTTTCTCAAGTC					32%	32%	36%
C18orf25-NM_001008239.3eU3645-3'UTR+2246	0					0	2	9%	2%	170	370	670	270	460	TTTCCCAATTCCACACAGCAGGTGATTTCCCGTGTG						10%	7%
C18orf54-NM_001288980.2eU1791-NP_001275909.1aa493SF	0					0	3	41%	16%	400	260	750	210	340	AGATTAAACAAGTTTCAGAAGATGATTTCTCTAAAT					59%	36%	28%
C1orf52-NR_024113.2eU511-ncr	0					0	2	7%	0%	340	180	700	260	260	GAGACCTACACCACTGAGAAGAAGCCTCCGCCTCCA						7%	7%
C1R-NM_001354346.2eU2020-NP_001341275.1aa648SF	2	2%	1%	2%	1%	0	3	25%	4%	620	110	570	350	470	TCCGGGGAAAGAATAGGATGGATGTGTTCTCTCAAA	3%	2%			22%	29%	24%
C2CD5-NM_001286173.2eU4035-3'UTR+621	0					0	3	24%	5%	240	270	690	80	440	GAAAATAATTTATCATAGCTGGTGTCCTTTCATTGA					23%	21%	30%
C2orf68-NM_001013649.4eU604-3'UTR+21	0					0	3	12%	1%	210	500	780	150	420	CCAACACTGACCATGTTGAAGGCGTTCTCTCCAGGC					11%	12%	14%
C4orf46-NM_001008393.4eU283-NP_001008394.1aa66SL	0					0	3	42%	8%	380	130	740	270	200	TGGAGCTGCAGATCGGAGACGCAGCCTTTTCATTAA					42%	49%	34%
C5orf22-NM_018356.3eU2023-3'UTR+602	0					0	3	19%	4%	350	200	740	390	350	AATTTTATAAAAATAGAGATGGGGTCTCACCATGTT					13%	21%	21%
C5orf51-NM_175921.6eU5163-3'UTR+4254	0					0	2	9%	4%	240	330	600	190	380	ATGTATTATGTATTTATGCTGGTGTTCTTTCATGTT						6%	12%
C6orf120-NM_001029863.2eU1725-3'UTR+801	0					0	2	8%	2%	350	200	710	220	200	TACTATAAAATACTTTGGAAGATGAATTCTCTACCT						9%	6%
C6orf89-NM_001286635.2eU4690-3'UTR+3450	0					0	2	8%	1%	260	60	690	230	230	CGAAGAAGACAGCTGTGGATGTCGTTGACTCAGATG					9%	7%	
C7orf26-NM_001303039.2eU1696-3'UTR+374	0					0	3	40%	4%	480	70	670	350	310	GCCTCTTTCCTCGTGAAGACGATGTGTCCCCGCCAG					40%	44%	35%
C8orf33-NM_023080.3eU1070-3'UTR+355	0					0	3	5%	2%	320	80	680	180	290	TAACAAAGATTAGTGGAGAAGCTGTATAATCGTACA					6%	3%	6%
C8orf82-NM_001001795.2eU473-NP_001001795.1aa93PL	0					0	2	10%	2%	330	300	670	530	240	ACCGCAGCGGGCGCTACGAGGCCGCTTTCCCCTTCC					8%		12%
CALCOCO2-NM_001261390.2eU1927-3'UTR+460	0					0	3	5%	1%	320	360	590	220	350	CCTTCCTCCTCATCCTTGGTGTTGCTTTCTCCAAAT					4%	6%	4%
CALM1-NM_001363669.2eU207-5'UTR-57	0					0	2	5%	2%	530	210	640	360	220	AACAGATTGCTGAATTCAAGGAAGCCTTCTCCCTAT					4%	6%	
CALM1-NM_006888.6eU255-NP_008819.1aa18SF	0					0	3	5%	1%	530	210	640	360	220	AACAGATTGCTGAATTCAAGGAAGCCTTCTCCCTAT					5%	5%	4%

CALR-NM_004343.4eU177-NP_004334.1aa36RC	0					0	3	3%	1%	440	260	560	370	470	CAGTTTCTGGACGGAGACGGGTGGACTTCCCGCTGG					2%	3%	3%
CAMLG-NM_001745.4eU888-NP_001736.1aa272TI	0					0	3	5%	2%	260	150	710	180	390	ATACCTATAGCAAAATGGGCGAAGTCTTCACAGATC					7%	3%	4%
CAMSAP2-NM_001297707.3eU3295-NP_001284636.1aa787TI	0					0	2	5%	1%	370	420	600	420	250	CCCAGAAAAAGAAAATGGAAGCTGCTTTTACCAAAC					5%	6%	
CANT1-NM_001159772.2eU3286-3'UTR+1694	0					0	2	5%	0%	580	140	630	420	0	CACAGCCCAGGTGCCCGGAAGCAGCCTCTTCGCATA					4%	5%	
CANX-NM_001024649.2eU4324-3'UTR+2459	0					0	2	8%	1%	380	130	710	220	110	TCTCACCACGTGAGTTTGATGCAGTCTTTTCTGTCT					7%		9%
CANX-NM_001024649.2eU4824-3'UTR+2959	0					0	2	24%	5%	270	170	570	330	370	GTATGAAGACATATTTGATTGTTGTTTTCTCTTGAT						20%	27%
CAP1-NM_001105530.2eU1921-3'UTR+428	0					0	3	5%	1%	370	290	670	280	330	GCATGAAGGTAGCAGAAGCTGGTGTGTTTCCAGATG					4%	5%	4%
CAPZB-NM_001206540.3eU454-NP_001193469.1aa118SF	0					0	3	2%	1%	320	170	690	210	320	GAGACCTGTATTTTGAAGGTGGCGTCTCATCTGTCT					3%	3%	2%
CARHSP1-NM_001042476.2eU746-NP_001035941.1aa129TI	0					0	3	3%	1%	420	70	710	250	300	AGAAGCTGCAGGCCGTGGAGGTCGTCATCACTCACC					2%	3%	4%
CASC3-NM_007359.5eU281-NP_031385.2aa90SL	0					0	3	19%	6%	260	180	660	190	480	AAGATGGCATTGAAGGTGATGCTGTTCTCTCGGATT					19%	13%	25%
CASC3-NM_007359.5eU3702-3'UTR+1578	3	8%	0%	8%	0%	0	3	77%	3%	360	130	810	210	230	CCCTTTGCCTTAAACCTGAAGATGTCTCCTCAAGCC	8%	9%	8%		78%	73%	79%
CASP2-NM_001224.5eU1698-3'UTR+671	0					0	3	3%	0%	150	80	650	80	400	TGAAGAGCCTGACAAGTGAAGTTGTAAACACAGTGT					2%	3%	3%
CASP4-NM_001225.4eU888-NP_001216.1aa280SL	0					0	3	13%	9%	670	180	680	460	0	ACTCTCCAGCATCCTTGGAAGTGGCCTCTTCACAGT					6%	23%	9%
CASP7-NM_001227.5eU540-NP_001218.1aa158PL	0					0	3	12%	3%	200	210	670	290	330	ATGTAATTTATGGGAAAGATGGTGTCACACCAATAA					14%	9%	13%
CASTOR2-NM_001145064.3eU3413-3'UTR+2142	0					0	2	41%	13%	470	150	620	190	210	TTCTGTCATTTCCCCCTGACGGTGTCACTTCTCTGC						32%	50%
CAT-NM_001752.4eU253-NP_001743.1aa58TI	0					0	3	33%	10%	390	170	690	410	320	GGCCCCTTCTTGTTCAGGATGTGGTTTTCACTGATG					42%	36%	23%
CAV1-NM_001172895.1eU2246-3'UTR+942	0					0	3	2%	0%	370	240	460	610	400	TGGGCTGGCAGTCCTGGAAGCCAGCTTTCCCTGCCT					2%	2%	3%
CAV2-NM_001206747.2eU2923-3'UTR+2390	0					0	3	7%	1%	280	230	640	140	390	CTGGGGGAGGTATATGATGAGCAGACTTCTCGGAAT					7%	7%	8%
CBX6-NM_001303494.2eU3119-3'UTR+1908	4	13%	6%	16%	3%	0	3	77%	5%	470	210	790	220	290	CGTGACTTTTTTGTAAAGAAGTTGTGTTTTCAGAGG	18%	16%	13%	5%	83%	73%	77%
CCAR1-NM_001282959.2eU505-NP_001269888.1aa135FF	0					0	2	6%	2%	610	380	580	180	520	GCAAACCCAGCCTCAGAAGCAGCGTGTTTTCACAGG						4%	7%
CCBE1-NM_133459.4eU5373-3'UTR+4071	0					0	2	3%	0%	570	260	610	430	220	AGTGGGGCTGCAGAGAGAATGGTGCCTTTTCTCACC					3%		3%
CCDC117-NM_001284263.2eU682-NP_001271192.1aa176TI	0					0	3	5%	0%	370	240	670	250	220	GTTTGAAGAGGGAATTTGATGAAGTTTTTACAAAGA					4%	5%	5%
CCDC127-NM_145265.3eU571-NP_660308.1aa167TI	0					0	3	45%	7%	200	260	710	190	450	TTTTGCTGAAAGAATTTGAAGCTGTTCTCACAGAAA					46%	38%	52%
CCDC59-NM_014167.5eU82-NP_054886.2aa24TI	0					0	2	6%	2%	390	270	710	360	440	GTATTGAGGCGCGTGGTGAAGGGGTTTCCACTGTCG					4%	7%	
CCDC6-NM_005436.5eU1613-3'UTR+56	0					0	3	2%	0%	230	210	770	180	300	GTTGGGACTCATCCAATGGAGCCGTCTACTCAACGC					2%	2%	3%
CCDC97-NM_001346100.2eU3132-3'UTR+1607	0					0	3	10%	2%	360	110	700	370	340	CAAAGGAGGCCTGGAAAGATGTTGCTTCCTCAAGGC					11%	8%	11%
CCHCR1-NM_001105563.3eU1391-NP_001099033.1aa397LF	0					0	2	4%	1%	400	300	750	310	530	CTGAACCGCTGGCGGGAGAAGGTGTTTGCCCTCATG						4%	3%
CCN2-NM_001901.4eU632-NP_001892.2aa144PP	0					0	2	2%	0%	250	110	520	410	330	GCCCCTGTGCAGCATGGACGTTCGTCTGCCCAGCCC					2%		2%
CCND3-NM_001760.5eU395-NP_001751.1aa79PL	0					0	3	10%	4%	430	240	740	450	250	AGGAGCAGCGCTGTGAGGAGGAAGTCTTCCCCCTGG					7%	14%	8%
CCNH-NM_001199189.2eU1371-3'UTR+20	0					0	3	76%	3%	270	260	760	210	260	AATCTCTCTAACCATTTGAAGTTGATTTCTCAATGC					74%	75%	79%
CCNYL1-NM_001142300.2eU3314-3'UTR+2130	0					0	3	8%	3%	390	240	530	350	420	TTTGAAATACTAAGAAGAGGTTAGTGTTCTCAACTA					8%	5%	12%
CCP110-NM_001199022.3eU779-NP_001185951.2aa206SL	3	14%	6%	14%	6%	0	3	6%	1%	270	200	730	190	330	AAGAAACTCTTATTTCTGATGGTCCCTTCTCAGTAA	14%	20%	8%		6%	7%	5%
CCT6A-NM_001009186.2eU1534-NP_001009186.1aa484LL	0					0	3	3%	0%	520	190	530	150	520	GATGAGATCATGCGAGCTGGAATGTCTTCTCTGAAA					3%	3%	3%
CCT8-NM_001282907.2eU1484-NP_001269836.1aa354SF	0					0	3	3%	1%	360	270	700	210	200	TTAAGCATGAAAAGGAAGATGGCGCCATTTCTACCA					2%	4%	3%
CCT8-NM_001282907.2eU851-NP_001269836.1aa143SF	0					0	3	2%	1%	500	0	650	210	110	AAAACCTTCGAGATATTGATGAAGTCTCATCTCTAC					2%	2%	3%
CD164-NM_001142401.3eU744-3'UTR+100	0					0	3	4%	2%	300	340	620	420	140	TTAAGATGTCCCACATGGAAGACGCTATTCCAGGAT					2%	6%	4%
CD2AP-NM_012120.3eU1418-NP_036252.1aa321PL	0					0	3	34%	3%	330	370	790	320	310	GCGAACTTAATGGTAAAGAAGGAGTATTTCCAGACA					31%	33%	38%
CD44-NM_000610.4eU4705-3'UTR+2343	0					0	3	3%	1%	410	150	780	180	350	GCCTGAAGTTTATGGAATAAGATGTATTCTCACTCC					2%	5%	3%
CDC25C-NM_001287582.2eU839-NP_001274511.1aa199LL	0					0	2	9%	2%	300	330	650	390	430	GAGATTTCAGATGAATTAATGGAGTTTTCCCTGAAA					11%	7%	
CDC27-NM_001114091.4eU1799-NP_001107563.1aa559SL	0					0	3	6%	2%	320	240	620	280	350	TTTGGCATCTTCAAAAAGATGTTGCTCTTTCAGTTC					5%	5%	9%
CDC37-NM_007065.4eU1276-3'UTR+76	0					0	2	2%	0%	350	190	530	290	240	CCCTTTTCAGAAAACAGATAGATGCCATCTCGCCCG					1%	2%	
CDC42SE2-NM_001038702.2eU1118-3'UTR+464	0					0	3	21%	7%	330	200	570	300	190	ATATCTGCATGCTTTATGAAGTTGTTGCTTCGGTAA					24%	14%	27%
CDC7-NM_003503.4eU91-5'UTR-72	0					0	3	22%	9%	240	270	740	180	400	GCATCTGGGGAGCCACAGAAGTCGTACTCCCTTAAA					12%	24%	29%
CDCA4-NM_017955.4eU568-NP_060425.2aa159HH	0					0	3	10%	4%	370	180	720	350	410	TGGCCCTCGAGAAAACAGAGGAAGCTTTCACAAGTC					8%	15%	7%
CDCA5-NM_080668.4eU1486-3'UTR+665	0					0	3	33%	5%	460	260	740	220	290	TAAGTGTGTACTACACAGAAGCTGTGTTTTCTAGTT					28%	31%	38%
CDCA7-NM_031942.5eU1016-NP_114148.3aa308SS	0					0	3	11%	4%	320	210	710	330	150	TGAAGATGACCTGCCCAGAAGCCGTCGCTCCAGATC					6%	13%	13%
CDCP1-NM_022842.5eU1734-NP_073753.3aa548TM	4	7%	3%	8%	1%	0	3	46%	3%	210	300	810	330	400	TACCTTATTTCAAAGAGGAAGGCGTTTTCACGGTGA	10%	8%	7%	3%	43%	49%	46%
CDH2-NM_001308176.2eU431-NP_001295105.1aa113PL	0					0	3	6%	2%	370	310	730	240	290	AGTCAGCAGAAGTTGAAGAAATAGTGTTCCCAAGAC					4%	6%	7%
CDH2-NM_001792.5eU274-NP_001783.2aa56LF	0					0	2	2%	0%	740	220	380	430	330	TCGAAGGATGTGCATGAAGGACAGCCTCTTCTCAAT					2%	2%	
CDIP1-NM_001199054.2eU639-NP_001185983.1aa144HY	1	6%		6%		0	2	16%	0%	440	160	760	310	390	GAGGGAGCGCCTGTGCAGACGGTGTGTCCCCACTGC	6%				15%	16%	
CDK13-NM_003718.5eU3502-NP_003709.3aa1039TI	0					0	2	15%	8%	300	110	680	280	380	AGCAGATGGGCATGACTGATGATGTTTCCACAATTA						10%	21%
CDK19-NM_001300960.2eU5371-3'UTR+3661	0					0	3	24%	7%	310	130	730	290	370	CTCTATGCCTCTAAAATGATGCTGTTTTCTCTGCTA					17%	31%	25%
CDK4-NM_000075.4eU305-NP_000066.1aa50PP	0					0	3	2%	1%	460	180	730	540	180	CAATGGAGGAGGAGGTGGAGGAGGCCTTCCCATCAG					1%	2%	2%
CDK6-NM_001145306.2eU1212-NP_001138778.1aa250PL	0					0	3	19%	5%	260	400	580	270	370	AAGAAGACTGGCCTAGAGATGTTGCCCTTCCCAGGC					13%	20%	24%
CDK6-NM_001145306.2eU2008-3'UTR+564	0					0	3	12%	8%	310	350	630	380	240	ACAGGCATTTGTTCACTGAAGGTGATTCACCAAAAT					12%	5%	21%
CEBPZOS-NM_001322373.2eU3065-3'UTR+2617	0					0	3	9%	1%	410	190	730	360	410	TTTGTATTTTTAGTAGAGATGGGGTTTCATCATGTT					8%	11%	9%
CELF1-NM_001172640.2eU2686-3'UTR+1077	0					0	2	20%	6%	360	140	780	240	290	TTGATTGAGGTGTCCCAGATGTAGTATTCCCACAGG					24%	16%	
CENPF-NM_016343.4eU112-5'UTR-58	0					0	3	12%	6%	290	200	770	400	470	AGAACTGTACTCTCCGAGAGGTCGTTTTCCCGTCCC					18%	5%	14%
CENPH-NM_022909.4eU1195-3'UTR+399	0					0	3	25%	4%	350	260	640	460	410	TTTTGTATTTTAGTAGAGACGGGGTTTCACCGTGTT					27%	20%	28%
CENPO-NM_001199803.3eU1702-3'UTR+768	0					0	3	23%	7%	260	200	740	200	280	CCTCGCCTCACAAAGCGGAAGCCGTACTCTCGGAGG					15%	25%	28%
CENPQ-NM_018132.4eU194-NP_060602.2aa31SL	0					0	3	30%	2%	400	150	750	260	170	GAAAAAAGGATAATGAGGAAGTTGTGTTGTCAGAGA					28%	32%	31%
CENPQ-XM_005249205.1eU242-XP_005249262.1aa31SL	0					0	3	26%	10%	400	150	750	260	170	GAAAAAAGGATAATGAGGAAGTTGTGTTGTCAGAGA					25%	37%	17%
CENPX-NM_001271006.2eU709-3'UTR+446	0					0	3	64%	3%	340	210	660	510	220	ATGCGATATTCCCCTTGGAAGATGCTTCCCCAGGCT					64%	60%	67%
CENPX-NM_144998.4eU189-NP_659435.2aa58Q*	0					0	3	9%	1%	300	230	740	330	460	GACGTGGACCAGCTGGAGAAGGTGCTTCCGCAGCTG					8%	8%	10%
CEP250-NM_001318219.1eU1250-5'UTR-877	0					0	3	24%	14%	280	210	800	180	380	AGGAATGCGCAAGAGGAGAAGTTGTCTTTACAGCAG					40%	20%	13%
CEP350-NM_014810.5eU3015-NP_055625.4aa890SF	0					0	3	22%	5%	350	210	770	250	370	CTGTGAAAGATGATAATGAAGATGTTTTCTCTGCCA					18%	19%	28%
CEP55-NM_001127182.2eU1038-NP_001120654.2aa286Q*	0					0	3	7%	1%	220	120	700	100	400	GTTCACAATTTAAATCAGCTGTTGTATTCACAAAGA					7%	7%	5%
CEP55-NM_001127182.2eU1459-NP_001120654.2aa426PL	0					0	3	4%	1%	310	120	600	400	120	CTGAAAACAGAGAAAAAGTTGCCGCCTCACCAAAAA					4%	2%	5%
CEP55-NM_018131.5eU290-5'UTR-36	0					0	3	4%	2%	350	150	490	410	210	CGGCTGGGCGCCTCAAGACCGTTGTCTCTTCGATCG					3%	2%	6%
CEP57L1-NM_001350652.2eU248-5'UTR-81	0					0	3	16%	2%	350	260	690	460	410	TTTGTATTTTTAAGAGAGACGGGGTTTCACCATGCT					17%	14%	16%
CEP89-NM_032816.5eU964-NP_116205.3aa294Q*	1	8%		8%		0	3	39%	5%	150	240	880	80	500	AAGCTCAAAGAGGCTGAGAAGGCGTCGTCACAGGAA		8%			35%	44%	37%
CERS2-NM_022075.5eU317-NP_071358.1aa33YY	0					0	3	6%	1%	590	100	440	500	200	CGATCTAGAAGACCGAGATGGACGTGTCTACGCCAA					5%	6%	6%
CERT1-NM_001130105.1eU1144-NP_001123577.1aa336PL	0					0	3	9%	5%	360	330	650	320	100	AAGTGGTAGAAGATGATGAAGATGACTTTCCTACAA					14%	5%	8%
CFL1-NM_005507.3eU785-3'UTR+203	4	1%	0%	1%	0%	0	3	4%	1%	350	230	560	370	220	CCCTCCATCCCTTGACGGTTCTGGCCTTCCCAAACT	1%	1%	1%	0%	4%	4%	5%
CFL2-NM_001243645.2eU385-NP_001230574.1aa60LL	0					0	3	4%	1%	390	140	580	270	300	CCCTACACATCTTTTGTGAAGTTGCTACCTCTGAAT					3%	4%	5%
CGAS-NM_138441.3eU2661-3'UTR+976	0					0	3	14%	3%	350	260	690	500	330	TTTGTATTTTTAGTAAAGATGGGGTTTCACCATGTT					15%	17%	11%
CHAC2-NM_001008708.4eU130-NP_001008708.1aa16PL	0					0	2	5%	1%	430	380	550	640	360	ACGGGTCCCTGATCTGGAAGGTGGATTTCCCCTATC					6%	4%	
CHAMP1-NM_001164144.3eU1557-NP_001157616.1aa414PL	0					0	3	16%	6%	280	220	530	610	460	TGTCTCCTGAACATTGGAAGGCAGTTCCCCCAGTGT					8%	21%	18%
CHAMP1-NM_001164144.3eU1875-NP_001157616.1aa520SF	0					0	2	4%	2%	330	270	490	400	600	CAGCCTCAGATATCTGGAAGCCTGTTCTCTCTATCG					3%		5%
CHD2-NM_001271.4eU3692-NP_001262.3aa1040HH	0					0	3	10%	3%	310	340	630	330	490	AGATGAAGAAGAGCTAGAAGAGCGTCCTCACAAGGA					13%	11%	8%
CHD3-NM_001005271.3eU5338-NP_001005271.2aa1776TI	0					0	2	2%	0%	260	240	770	140	330	TCATGTTCAATATCGCCGATGGTGGCTTCACAGAGC						2%	2%
CHD9-NM_001308319.2eU5512-NP_001295248.1aa1754SL	0					0	3	11%	5%	460	60	720	400	290	TTAAAGATGATATAGAGGATGATGTTTCCTCACCAG					17%	8%	8%
CHEK1-NM_001114121.2eU2535-3'UTR+207	0					0	3	38%	8%	430	320	790	330	450	TTTGTATTTTTAGTAGAGATGGGGTTTTTTCATGTT					39%	44%	29%

CHEK1-NM_001114122.3eU2881-3'UTR+580	0					0			3	19%	4%	300	510	700	250	390	ATTAATTAAAATTGCAAGTAGGTGTTTTTTCCAGTG								16%	23%	18%
CHIC2-NM_012110.4eU847-3'UTR+44	0					0			3	12%	5%	280	250	640	400	210	TATAGAGACTTTCCAAGTATGTTGTCTTTCCAATGG								9%	10%	18%
CHM-NM_000390.4eU741-NP_000381.1aa239R*	0					0			2	14%	4%	230	120	690	180	390	ATTGATTTAGTATCAAAGCTGCTGTATTCTCGAGGA								17%		11%
CHMP5-NM_016410.6eU623-NP_057494.3aa198TI	0					0			3	29%	5%	290	280	680	220	570	CTGCACCTGCAATTCCAGAAGGTGTTCCCACTGATA								27%	25%	34%
CHORDC1-NM_001144073.2eU2677-3'UTR+1640	0					0			2	14%	8%	340	300	690	440	370	TGTTCATACTTCCTTCTGAGGCTGTTTCCCCCTAAA									20%	9%
CHP1-NM_007236.5eU1332-3'UTR+629	0					0			3	7%	3%	430	60	640	380	140	ATTAAATGGGCCTCTGGGATGTTGCCTCTTCAGGAG								5%	10%	6%
CHPF2-NM_001389651.1eU915-5'UTR-616	0					0			2	13%	10%	280	290	660	60	500	GGAGGGGTTCCTGTAGCCGTTGCGTCTTCTCAAACA								6%		21%
CHSY1-NM_014918.5eU3777-3'UTR+776	0					0			3	7%	1%	350	260	690	460	410	TTTGTATTTTTAGTAGAGACGGGGTTTCACCATGCA								6%	8%	7%
CIC-NM_001304815.2eU4291-NP_001291744.1aa1406PS	0					0			3	10%	3%	210	400	770	290	460	AACAAGGGCTTTGGTCGGAAGGTGTTTCACCCTGTG								7%	12%	12%
CIC-NM_001304815.2eU8132-3'UTR+503	0					0			2	9%	4%	260	400	690	420	380	TGGGTTGTACTTTCTAAGAAGGTGATTCCCCCTGCC								6%		12%
CIRBP-NM_001280.3eU165-NP_001271.1aa27SL	0					0			2	3%	1%	530	160	740	350	290	CCAATGAGCAGTCGCTGGAGCAGGTCTTCTCAAAGT								4%		2%
CIT-NM_001206999.2eU221-NP_001193928.1aa46LF	0					0			3	15%	0%	360	80	720	170	350	CCCTTTATGACTCAACAGCAGATGTCTCCTCTTTCC								15%	15%	14%
CKAP2L-NM_001304361.2eU793-NP_001291290.1aa116HY	3	8%	1%	8%	1%	0			3	7%	5%	310	210	660	310	390	GGAGTAAAACCCTCAAGGACGGTTCCCTCTCACTTT	9%	7%	7%					13%	3%	4%
CLDND1-NM_001040181.2eU321-NP_001035271.1aa83TT	0					0			3	13%	3%	310	160	690	310	370	CACAGTGGGATTGTGGAGACGGTGTATCACCATACC								10%	15%	14%
CLK1-NM_001162407.1eU1788-NP_001155879.1aa485Q*	0					0			3	2%	0%	310	220	510	260	460	TGTAAACCTCTGAAGGAATTTATGCTTTCTCAAGAT								2%	2%	2%
CLK2-NM_001294338.2eU317-NP_001281267.1aa5R*	0					0			3	9%	2%	410	50	650	340	140	GGCCTCGCCGCCAGAACGATGCCGCATCCTCGAAGG								12%	9%	8%
CLNS1A-NM_001293.3eU660-NP_001284.1aa194Q*	3	2%	1%	2%	1%	0			3	22%	1%	470	290	510	350	630	ACACTGGAGAGATTAGAAGGAATGCTTTCTCAGTCT	2%	1%	1%					21%	24%	22%
CLPTM1L-NM_030782.5eU1040-NP_110409.2aa261SF	0					0			3	3%	1%	450	100	710	300	170	TCTGGATCCACATGCAGGACGCCGTGTACTCCCTGC								2%	4%	2%
CLPTM1L-NM_030782.5eU1706-NP_110409.2aa483AV	0					0			3	6%	1%	320	230	710	180	150	CTTTCAACACCTTCATTGATGACGTCTTTGCCTTCA								5%	7%	6%
CLSPN-NM_001190481.2eU4396-3'UTR+460	0					0			2	3%	0%	350	260	690	460	410	TTTGTATTTTTAGTAGAGACGGGGTTTCACCATGTT								3%		3%
CLTC-NM_001288653.2eU4531-NP_001275582.1aa1432PS	0					0			3	2%	1%	250	370	640	200	340	TTGTTAAATGATTTGCTGATGGTGCTGTCTCCACGG								2%	3%	2%
CLUH-NM_001366661.1eU416-NP_001353590.1aa105Q*	0					0			2	2%	0%	160	290	660	210	380	ATCGAGCCCTTCTCCCTGCAGGTGTCCCCCCAGGAG								2%	2%	
CLUH-NM_001366661.1eU4331-3'UTR+181	0					0			3	10%	1%	380	360	740	250	390	GGCCTGCCTGCCCCCGCAGAAGGTGTTTTGCGCTGG								9%	11%	9%
CLUH-NM_001366661.1eU4562-3'UTR+412	0					0			3	7%	2%	320	460	550	290	530	CTGAGTGGGTGACGCGCAGACGGGATTTCTCAGGTC								5%	8%	8%
CLU-NM_001831.4eU1445-3'UTR+20	0					0			3	3%	0%	420	190	610	380	160	GGGAGGAGTGAGATGTGGATGTTGCTTTTGCACCTA								3%	2%	3%
CMTM6-NM_017801.3eU802-3'UTR+143	0					0			3	4%	2%	350	220	630	540	290	GCAAGGGGAAGGTCAAGAAGGCAGTTTTATCAATAT								6%	2%	5%
CMTR1-NM_015050.3eU2646-NP_055865.1aa828SF	0					0			2	4%	1%	330	220	750	290	360	AGGACAAGCTGTCCAAGGAGGACGTCCTCTCCTTCA								4%		5%
CNBP-NM_001127192.2eU71-5'UTR-66	0					0			3	3%	1%	170	450	660	320	300	AAACAGCCTCTACCTTGCGAGCCGTCTTCCCCAGGC								3%	3%	2%
CNDP2-NM_001168499.2eU801-NP_001161971.1aa240SF	1	1%		1%		0			3	10%	3%	350	240	840	190	260	TGTCCCTCCATGGCATCGAAGGCGCCTTCTCTGGGT		1%						11%	7%	12%
CNDP2-NM_001370248.1eU120-NP_001357177.1aa5TI	0					0			2	6%	2%	360	250	450	500	380	TGGGGTCTGGTTGAAAGATGGCGGCCCTCACTACCC								4%		7%
CNDP2-NM_001370254.1eU175-5'UTR-925	0					0			2	4%	0%	360	250	450	500	380	TGGGGTCTGGTTGAAAGATGGCGGCCCTCACTACCC								4%		4%
CNOT2-NM_001199302.2eU1010-NP_001186231.1aa183R*	0					0			3	8%	3%	270	200	720	230	260	ATAATATGTATGCCAAAGCAGCAGCCTTCTCGACAG								5%	10%	7%
CNOT6-NM_001370472.1eU4251-3'UTR+2190	4	32%	10%	37%	1%	1	7%		2	5%	1%	350	130	720	300	110	TGTATTTGTTAATGGAGGATGTCATCTTTTCATAGA	36%	38%	39%	17%			7%		5%	6%
CNOT8-NM_001301073.2eU201-5'UTR-15	0					0			3	23%	4%	310	150	850	140	140	TGTAAAACTAGTTAGCTGAAGACGACTTCTCAGGTT								21%	21%	28%
CNOT8-NM_001301075.2eU249-5'UTR-139	0					0			2	33%	1%	310	150	850	140	140	TGTAAAACTAGTTAGCTGAAGACGACTTCTCAGGTT								32%	33%	
CNPPD1-NM_001321389.1eU472-NP_001308318.1aa15SF	4	35%	9%	39%	3%	2	17%	2%	3	37%	10%	370	340	800	230	300	GGCTCCTGCTGGACGAAGAAGGCACCTTCTCCCTCG	37%	42%	38%	22%	16%		19%	41%	44%	25%
CNPPD1-NM_015680.6eU141-NP_056495.4aa15SF	4	33%	14%	40%	5%	2	15%	2%	2	25%	3%	370	340	800	230	300	GGCTCCTGCTGGACGAAGAAGGCACCTTCTCCCTCG	43%	35%	42%	12%		16%	13%	28%		23%
CNPY2-NM_014255.7eU362-NP_055070.1aa57RW	4	24%	9%	29%	0%	1	2%		2	3%	1%	380	330	670	360	270	CCCAAGAAGACCATTCAGATGGGATCTTTCCGGATC	29%	29%	29%	11%			2%	2%	3%	
CNPY4-NM_152755.2eU111-5'UTR-2	1	2%		2%		0			3	29%	12%	310	220	770	260	260	AGCGGTGATTGTTTGTAGACGGCGCTTTGTCATGGG		2%						26%	19%	42%
CNRIP1-NM_001371604.1eU531-NP_001358533.1aa96PL	0					0			3	9%	3%	280	290	730	250	430	CGGGTACATATGACACAGAAGGTGTGACCCCAACGA								10%	6%	11%
COASY-NM_001042529.3eU430-NP_001035994.1aa116TI	0					0			3	27%	2%	340	150	790	140	240	ATCTCGCCCACCCGCCAGAAGTCGTGTTGACAGATT								25%	29%	28%
COG1-NM_018714.3eU2414-NP_061184.1aa800PL	0					0			3	23%	6%	320	370	730	370	300	AAAAACAGATTAAGAAAGAAGGTGCATTTCCAGTCA								18%	29%	22%
COL12A1-NM_004370.6eU7842-NP_004361.3aa2510LL	0					0			2	2%	0%	210	200	720	80	340	TTGTGAAACTGCCACTTCAAGTTGTCCTCTCATTTA								2%	2%	
COL1A1-NM_000088.4eU18-5'UTR-102	0					0			3	7%	3%	510	320	440	670	290	GCCAGTCGTCGGAGCAGACGGGAGTTTCTCCTCGGG								8%	9%	4%
COL1A1-NM_000088.4eU225-NP_000079.2aa36PL	0					0			3	3%	1%	290	140	700	220	220	GCCAAGTCGAGGGCCAAGACGAAGACATCCCACCAA								2%	3%	3%
COL3A1-NM_000090.4eU5182-3'UTR+664	0					0			2	2%	0%	230	200	260	80	180	GCTGGTCAAGATTACTAATATTTGGGAAGGCTTTAA								2%		2%
COL5A1-NM_000093.5eU6443-3'UTR+541	0					0			3	5%	2%	210	360	680	250	280	AGGCCAAATGTCATTCTGCAGGTGCCTTCCCGATGG								6%	2%	5%
COLGALT1-NM_024656.4eU2598-3'UTR+664	0					0			3	2%	1%	350	260	690	460	410	TTTGTATTTTTAGTAGAGACGGGGTTTCACCATGTT								3%	2%	3%
COPA-NM_001098398.2eU341-NP_001091868.1aa87FF	0					0			3	3%	1%	450	210	680	180	260	TTGGAATTACAAGCTTCGGCGCTGTCTTTTCACATT								3%	4%	2%
COPG1-NM_016128.4eU2789-3'UTR+60	0					0			3	9%	3%	410	290	570	270	200	TTCCCCAACACTACCTGGAAGTTGTGCCTTCCTCAT								6%	10%	12%
COPS7A-NM_001164093.2eU1216-3'UTR+18	0					0			3	3%	1%	380	200	550	400	390	CAAGTCGAATTGAAAGGACTGTCGTTTCCTCCCTGG								2%	3%	4%
COPS7B-NM_001282949.3eU2150-3'UTR+1364	0					0			3	46%	10%	260	130	680	420	370	GTGGTTCAGGAAAATGTGATGCTGTTTCCCCATGTT								34%	50%	53%
COPZ1-NM_001271734.2eU1260-3'UTR+762	0					0			2	2%	1%	350	150	530	150	400	CCTGTGGGATCGTTGCCTGGTTTGTCTTCTCTGTGT									2%	3%
COQ10B-NM_001320818.2eU333-NP_001307747.1aa108FF	0					0			3	9%	2%	380	310	580	390	200	AAAGGCATCTTGTACTGATGGGAGACTTTTCAATCA								10%	7%	11%
COQ9-NM_020312.4eU256-NP_064708.1aa77HY	0					0			2	3%	1%	310	200	660	60	450	GGGGCAGAAAAACCTGATCCAGAGTCTTCTCATTCA								2%		3%
CORO1C-NM_001105237.2eU1597-NP_001098707.1aa516SS	0					0			3	27%	1%	400	420	500	490	310	CACAATCTGCAATCAAGATGAGCGTATTTCCAAGTT								25%	28%	27%
COX10-NM_001303.4eU2171-3'UTR+737	0					0			2	7%	3%	420	300	570	380	200	ACATATAGACACTGTTGGAAGCAGTTCCTTCTAAAA								10%		5%
COX5A-NM_004255.4eU533-3'UTR+21	0					0			2	3%	1%	350	470	530	470	330	CAAAGTGTAAACCGCATGGATGGGCTTCCCCAAGGA									4%	2%
COX5B-NM_001862.3eU139-NP_001853.2aa38PP	0					0			3	13%	1%	560	180	740	490	250	GCGCTCCATGGCATCTGGAGGTGGTGTTCCCACTGA								12%	13%	15%
COX6A1-NM_004373.4eU37-NP_004364.2aa8SL	0					0			3	10%	1%	360	90	670	170	390	GATCAAAAATGGCGGTAGTTGGTGTGTCCTCGGTTT								12%	9%	10%
COX6B1-NM_001863.5eU158-NP_001854.1aa22PP	0					2	1%	0%	3	7%	0%	260	300	540	590	200	CAAGACCGCCCCTTTTGACAGCCGCTTCCCCAACCA					1%		1%	7%	6%	6%
CPLANE1-NM_001384732.1eU5336-NP_001371661.1aa1719FF	0					0			3	48%	0%	430	260	790	180	250	CAAGTCCATTAAAACTAGAAGATGTATTTTCAAAGC								48%	48%	48%
CPNE3-NM_003909.5eU1450-NP_003900.1aa432TI	0					0			3	40%	3%	280	160	710	80	440	TGCTTTTGATTATTACTGATGGTGTGATCACAGACC								40%	42%	37%
CPNE3-NM_003909.5eU4561-3'UTR+2792	0					0			3	28%	2%	300	240	700	340	440	TTTTGTAAACTTCCTGGAAAGCTGTCTTCACTAAGT								27%	28%	30%
CPOX-NM_000097.7eU707-NP_000088.3aa200FF	0					0			3	50%	14%	510	310	540	430	370	CAGCTGTGTACTTCAAGATGGGTGTGTTTTCGAAAA								37%	50%	64%
CPSF2-NM_001322270.2eU4405-3'UTR+1956	0					0			3	30%	8%	350	160	760	140	360	CCTCATGCTGTTCTTGTGATGGTGAGTTCTCATGAG								25%	26%	39%
CPSF2-NM_001322270.2eU4634-3'UTR+2185	0					0			3	17%	8%	420	320	690	500	410	TTTGTATTTTTAGTAGAGACGGGGTTTCTCCATGTT								24%	9%	19%
CPZ-NM_001014447.3eU1279-NP_001014447.2aa404PS	0					0			3	13%	4%	460	310	810	210	490	AAGCACCCCCAGGAGGAGAAGATGTTTTCTCCCACG								9%	16%	15%
CREB5-NM_001011666.3eU661-NP_001011666.1aa215Q*	0					0			3	10%	2%	240	70	640	270	220	TCACCAGCAACACAACAGATGCAGCCAACCCAGACA								8%	10%	11%
CREBL2-NM_001310.4eU2068-3'UTR+1408	0					0			3	47%	8%	350	230	730	100	480	TTCCCATGGTTAAAACAGGAGTTGTGTTCTCTGTCG								38%	48%	55%
CRIP2-NM_001270837.2eU949-NP_001257766.1aa216LL	0					0			3	16%	4%	250	240	770	80	440	AAGGTGTACTTCGCTGAGAAGGTGACGTCTCTGGGC								15%	13%	20%
CRNKL1-NM_001278625.2eU1461-NP_001265554.1aa477HY	0					0			3	13%	3%	410	170	740	210	350	CAGTACGAAGAAGAAGTGAAGGCGAATCCACACAAT								16%	10%	13%
CRTAP-NM_001393363.1eU1591-3'UTR+405	0					0			3	2%	1%	300	330	750	390	450	TTTGTATTTTTAGTAGAGACGGGGTTTTGCCATGTT								2%	3%	3%
CRTAP-NM_001393363.1eU1831-3'UTR+645	0					0			2	2%	0%	350	190	660	310	540	TTACCATTTTGGTTTGAAGGGCAGTCTTCTCTGGCT								3%	2%	
CSNK1A1-NM_001025105.3eU4170-3'UTR+2597	0					0			3	18%	7%	290	300	430	430	560	TACAAATGAATATTTGGAGGTTTGCTTTCACAATGA								12%	16%	25%
CSNK1G1-NM_001329606.2eU5734-3'UTR+4133	0					0			3	12%	6%	440	190	860	140	290	ACTTCCATGGCAGCCAAGAAAACGTCTTCTCAGAGG								10%	19%	8%
CSPP1-NM_001291339.2eU3282-NP_001278268.1aa739PL	0					0			3	22%	4%	330	190	750	220	360	CATATCCTGCCATTGAAGATGACGTCCTCCCTCCAC								26%	23%	19%
CTBP2-NM_001083914.3eU250-5'UTR-161	0					0			3	43%	12%	300	310	770	280	390	AATGGTTTTGAACCAATGAAGGTGTATTCCCTTAAA								44%	55%	31%
CTBP2-NM_001329.4eU291-5'UTR-161	1	6%		6%		0			3	36%	14%	300	310	770	280	390	AATGGTTTTGAACCAATGAAGGTGTATTCCCTTAAA		6%						22%	36%	50%
CTDP1-NM_001202504.1eU579-NP_001189433.1aa150HY	3	12%	9%	12%	9%	0			2	12%	2%	330	210	780	350	410	TTTTTAGACCCCGAGAAGAAGCTTTTTTCTCACCGA	22%	6%	8%					11%		14%
CTDSP2-NM_005730.4eU1082-NP_005721.3aa263TI	0					0			2	5%	2%	330	150	760	230	340	AGGAGCTGAGCGGAGCAGAGGACGTCTACACCAGCC								3%		6%
CTDSPL2-NM_016396.3eU2385-3'UTR+816	0					0			3	5%	2%	530	150	680	180	230	AAGATTTAAACCATTGTGATGGCGTATATTCTCAAA								5%	3%	6%
CTNNA1-NM_001290307.3eU3348-3'UTR+734	4	28%	8%	32%	2%	3	1%	0%	3	7%	1%	300	310	820	240	450	AGGAAGCAGTCCCTGGAGAAGGCATGTTCCCAGAAA	34%	30%	34%	16%	1%	2%	1%	6%	8%	8%

CTNND1-NM_001085458.2eU4875-3'UTR+1437	0					0		3	5%	1%	350	240	770	470	290	TCTGAAGGAGTTACAGGGAAGTGGTCTCCCCAATTC							6%	5%	5%
CTSA-NM_000308.4eU1381-NP_000299.3aa446HY	0					0		3	4%	0%	300	420	750	250	500	CAGATTGCCGGCTTCGTGAAGGAGTTCTCCCACATC							4%	4%	5%
CTSA-NM_000308.4eU187-NP_000299.3aa48RC	0					0		3	18%	4%	340	280	710	320	190	CTCCCCGGGCTGGCCAAGCAGCCGTCTTTCCGCCAG							14%	22%	17%
CTSB-NM_001317237.2eU842-NP_001304166.1aa130SF	0					0		3	11%	4%	430	220	710	430	320	ACAAAAACGGCCCCGTGGAGGGAGCTTTCTCTGTGT							7%	11%	14%
CTSC-NM_001814.6eU972-NP_001805.4aa303PL	0					0		3	5%	1%	260	390	850	240	300	AGTATGCTCAAGGCTGTGAAGGCGGCTTCCCATACC							4%	6%	4%
CTSD-NM_001909.5eU1071-NP_001900.1aa335LL	0					0		3	15%	0%	190	290	730	180	540	TACATGATCCCCTGTGAGAAGGTGTCCACCCTGCCC							15%	15%	15%
CTTNBP2NL-NM_018704.3eU2133-3'UTR+21	0					0		3	6%	2%	340	90	720	420	340	CAGCAGCTAGTCCCTAGGAGGGAGTCTCCACGTTTG							6%	9%	4%
CUL1-NM_001370660.1eU2527-NP_001357589.1aa621TI	0					0		3	8%	1%	330	80	660	270	250	TGCTTCAGTACAACACGGAAGATGCCTACACTGTGC							9%	7%	7%
CUL3-NM_001257197.2eU1479-NP_001244126.1aa375LL	0					0		3	6%	1%	420	240	770	220	300	TTATAAACAACACTTGGCAAGGAGACTTCTCACAAA							5%	8%	6%
CUL4A-NM_001008895.4eU2887-3'UTR+592	0					0		2	2%	0%	430	230	440	500	240	AAAAGTTGTAAACTTTGATGGCTGATTTTTCGTAAG							2%		2%
CYB5B-NM_030579.3eU133-NP_085056.2aa23SL	0					0		2	3%	0%	340	80	670	270	100	GCGATGGGAAAGGGCAGGAAGTCGAGACCTCAGTCA							3%	3%	
CYB5B-NM_030579.3eU755-3'UTR+237	0					0		3	9%	1%	420	150	630	240	410	TTCCGTGTTGAACAATTGCCGGTGTTTCCTCTCTTC							8%	10%	9%
CYB5R3-NM_000398.7eU935-NP_000389.1aa286PP	0					0		2	4%	0%	290	230	480	420	440	CCCACCCATGATCCAGTACGCCTGCCTTCCCAACCT							4%		4%
CYCS-NM_018947.6eU118-NP_061820.1aa17Q*	0					0		3	2%	1%	330	140	660	280	210	GGCAAGAAGATTTTTATTATGAAGTGTTCCCAGTGC							3%	2%	2%
CYFIP1-NM_001033028.3eU1992-NP_001028200.1aa564Q*	0					0		3	11%	3%	350	340	680	260	400	GAGTACGCAGAGCTGAAGACGGTGTGCTTCCAGAAC							9%	11%	14%
CYLD-NM_001042355.2eU2419-NP_001035814.1aa726TI	0					0		2	6%	2%	160	250	650	220	550	TGGAAAAAAATGAGAAAGTTGGCGTTCCCACAATTC							4%		7%
CYP51A1-NM_000786.4eU1571-NP_000777.1aa486PP	4	16%	4%	18%	2%	0		2	3%	1%	390	240	440	570	340	TGAATTTGATCTCATTGATGGATACTTTCCCACTGT	15%	19%	19%	10%			2%	4%	
CZIB-NM_001304759.2eU788-3'UTR+303	4	25%	11%	30%	4%	0		3	7%	2%	220	160	730	310	470	AAGAGTATTTTTAAGGAGATGGTATCTCCCCAGTCA	28%	28%	35%	10%			8%	8%	5%
DAG1-NM_001177634.2eU342-5'UTR-382	0					1	25%	2	37%	10%	390	130	680	250	360	ACCTGCGAGTGTATTTGGATAGTGTCTCCACAGAAA						25%	44%		30%
DBNL-NM_001014436.3eU910-NP_001014436.1aa292Q*	0					0		2	4%	1%	190	310	630	340	310	AGGAGCCCCTTCCTGCAGAAGCAGCTCACCCAACCA							3%		5%
DCAF10-NM_001286810.2eU730-NP_001273739.1aa219PL	0					0		3	22%	3%	360	300	730	380	410	CACGAGCCTCTCAAAGAGAAGGAGTTTCACCACGAA							20%	20%	24%
DCAF15-NM_001393637.1eU182-NP_001380566.1aa60RW	0					0		3	15%	7%	300	150	660	480	160	TCCCCTCGCCTCTTCCGGAAGCTGCCTCCCCGGGTG							8%	13%	23%
DCAF7-NM_005828.5eU455-NP_005819.3aa85PL	0					0		3	8%	2%	330	340	740	360	310	TGTGGATCCCTGACACAAAAGGCGTCTATCCAGACC							10%	6%	7%
DCAF7-NM_005828.5eU5509-3'UTR+4279	0					0		2	5%	0%	260	360	730	290	280	TTAAAATATGGAAACATGCAGGTGCCTTCCCAAAGA							5%	5%	
DCPS-NM_014026.6eU289-NP_054745.1aa90Q*	0					0		2	10%	2%	360	210	750	170	200	GCCGTTGTGATCCTGGAGAAGACGCCATTTCAGGTG							9%	12%	
DCTN2-NM_001261412.2eU1554-3'UTR+213	0					0		3	5%	0%	620	150	600	680	250	GGGTTGGTTATCATCAGAAGGTGGTCTCCCCTCAGG							6%	6%	5%
DCTN4-NM_001135643.2eU1504-3'UTR+76	0					0		2	3%	1%	370	200	560	430	120	ACCTGCGTTAAAATGTGGAAGCTGCTGCTTCATTAG							2%	4%	
DCUN1D4-NM_001040402.3eU395-NP_001035492.1aa102SL	0					0		3	7%	1%	420	260	780	270	120	CTAGAATAAAGACTGAAGAAGAAGCCTTTTCAAGTA							7%	7%	6%
DDA1-NM_024050.6eU2076-3'UTR+1675	0					0		3	23%	5%	270	220	830	210	410	TTTCTATTTTTAGTGGAGACGGCGTTTTGTCATGTT							27%	17%	25%
DDR2-NM_001014796.3eU1425-NP_001014796.1aa329PL	0					0		2	18%	17%	190	220	650	260	170	CCTTCCCCCTTGTCCTGGATGACGTCAACCCCAGTG							6%		30%
DDR2-NM_001014796.3eU701-NP_001014796.1aa88Q*	0					0		3	7%	1%	380	290	690	320	210	GTGGAACCTGATGACCTGAAGGAGTTTCTGCAGATT							7%	6%	7%
DDX1-NM_004939.3eU80-NP_004930.1aa5SF	1	1%		1%		0		3	7%	1%	490	250	560	560	260	CGGAGGACGGGGTGAAGATGGCGGCCTTCTCCGAGA			1%				7%	8%	6%
DDX21-NM_001256910.2eU173-5'UTR-48	0					0		2	6%	2%	340	470	680	350	410	AGATAGCAGAAGAGGAAGAAACTGTTTTCCCCAAAG							4%		8%
DDX21-NM_001256910.2eU581-NP_001243839.1aa121SF	4	23%	6%	26%	2%	0		3	68%	1%	400	350	830	300	400	TACCTGTGGAACAAAAAGAAGGCGCTTTCTCTAATT	28%	25%	23%	15%			68%	68%	66%
DDX21-NM_004728.4eU214-NP_004719.2aa53PL	0					0		3	3%	1%	340	470	680	350	410	AGATAGCAGAAGAGGAAGAAACTGTTTTCCCCAAAG							3%	5%	2%
DDX23-NM_004818.3eU198-NP_004809.2aa40PS	0					0		3	2%	0%	290	290	620	290	410	CGGGATAGAGACCGGGACCGGAAGTCTTCCCCATCT							1%	1%	2%
DDX24-NM_020414.4eU588-NP_065147.1aa167Q*	0					0		3	5%	2%	320	160	730	230	340	AATAAAGGGAAAAAAGGGTTGGAGCCTTCTCAGAGC							8%	3%	6%
DDX46-NM_001300860.2eU1839-NP_001287789.1aa560SL	4	24%	10%	30%	2%	0		2	3%	1%	420	170	470	430	250	TTCGTCCTGATCGACAGACGGTTATGTTTTCAGCTA	27%	32%	30%	9%			3%		4%
DDX46-NM_001300860.2eU241-NP_001287789.1aa27PP	0					0		3	32%	2%	380	260	620	300	310	CTCTGGAAGTCGGTCTAGAAGTCGCTCACCCTCAGA							30%	34%	33%
DDX50-NM_024045.2eU489-NP_076950.1aa140SF	4	7%	3%	8%	3%	0		3	46%	7%	360	390	840	230	340	TAACACGTGAACAGAAAGAAGGAGCCTTCTCCAATT	11%	7%	5%	5%			42%	44%	54%
DDX54-NM_001111322.2eU289-NP_001104792.1aa88Q*	0					0		2	2%	0%	380	160	700	290	400	GAGCCGGACACCCGGGAGATGGTGCGTGCCCAGAAC								2%	2%
DDX5-NM_001320595.2eU2320-3'UTR+220	0					0		2	2%	0%	650	130	630	390	190	TTTTGTACTAGAAAATGCAGGCAGTGTTTTCACAAA							2%		2%
DDX5-NM_001320595.2eU2322-3'UTR+222	0					0		3	3%	1%	300	390	650	130	630	TTGTACTAGAAAATGCAGGCAGTGTTTTCACAAAAG							3%	2%	4%
DDX6-NM_001257191.3eU4608-3'UTR+2801	0					0		3	27%	7%	210	210	780	290	380	AAGCATTGTTACTATTGGAAGTTGTTTTCACATTCT							20%	26%	34%
DEDD2-NM_001270615.2eU163-NP_001257544.1aa27HY	0					0		3	8%	2%	430	60	630	310	210	TGCCTGGACTACTACGGGATGCTGTCGCTTCACCGT							10%	8%	5%
DEDD-NM_001039711.2eU1297-3'UTR+159	0					0		3	13%	6%	300	410	730	400	210	AAAGACAAAAGAAAGTGGAAGTGGTATTCCCCACCC							6%	13%	19%
DELE1-NM_001142603.3eU297-NP_001136075.1aa76RC	0					0		2	9%	1%	290	180	620	370	430	TGGAAGGATGCCTTCCAATGGATGTCTTCCCGTGTC								8%	10%
DENND4C-NM_001330640.2eU6962-3'UTR+927	0					0		3	9%	4%	300	310	560	450	280	TACCTGAAAATCTGATGACAGCTGCCTTCCCTAACT							14%	7%	6%
DENR-NM_003677.5eU219-NP_003668.2aa28PL	0					0		3	29%	3%	310	130	700	350	240	GGAACAGTGCCAAGTTAGATGCCGATTACCCACTTC							28%	26%	32%
DERL2-NM_001304777.2eU583-NP_001291706.1aa189PL	0					0		3	40%	4%	370	430	660	360	170	ACATATATTTTTTCTTGGAAGATGTATTTCCCAATC							45%	37%	39%
DERL2-NM_001304777.2eU584-NP_001291706.1aa189PP	0					0		3	14%	3%	420	370	430	660	360	CATATATTTTTTCTTGGAAGATGTATTTCCCAATCA							16%	17%	10%
DERL2-XR_002958015.1eU1346-ncr	0					0		3	58%	15%	370	430	660	360	170	ACATATATTTTTTCTTGGAAGATGTATTTCCCAATC							54%	45%	75%
DERL2-XR_002958015.1eU1347-ncr	0					0		3	32%	20%	420	370	430	660	360	CATATATTTTTTCTTGGAAGATGTATTTCCCAATCA							11%	36%	50%
DGUOK-NM_001318859.2eU114-NP_001305788.1aa28SF	0					0		2	5%	0%	400	250	750	370	350	TGGCCAAGAGCCCACTCGAGGGCGTTTCCTCCTCCA							5%	5%	
DGUOK-NM_001318860.2eU114-5'UTR-97	0					0		2	4%	3%	400	250	750	370	350	TGGCCAAGAGCCCACTCGAGGGCGTTTCCTCCTCCA							2%		6%
DHX33-NM_001199699.2eU447-5'UTR-36	0					0		3	19%	6%	350	0	700	150	230	GCTATACAGTGCGCTTTGATGATGTCACCTCAGAAG							20%	25%	13%
DHX33-NM_020162.4eU608-NP_064547.2aa162SL	0					0		3	18%	3%	350	0	700	150	230	GCTATACAGTGCGCTTTGATGATGTCACCTCAGAAG							18%	21%	15%
DHX9-NM_001357.5eU3110-NP_001348.2aa993SF	0					0		3	6%	2%	310	230	660	230	330	GACCAGATAATAATTTGGATGTTGTTATCTCCCTCC							8%	5%	6%
DIABLO-NM_001278302.1eU1593-3'UTR+480	0					0		3	2%	0%	440	70	570	100	310	ATTTCTTTGTATGTATTTCTGATGTGTTCTCTGATC							2%	2%	2%
DIABLO-NM_001371333.1eU145-NP_001358262.1aa36SL	0					0		3	5%	2%	450	380	510	600	440	TTGTGGCTAACTTTAAGAAGCGGTGTTTCTCAGAAT							3%	5%	7%
DIRAS3-NM_004675.5eU1264-3'UTR+429	0					0		2	17%	10%	290	430	650	370	540	TTAATTGTGTATTTAAGAAAAGTGTTTTCACAACAA							11%	24%	
DISP2-NM_033510.3eU797-NP_277045.1aa232LF	0					0		3	27%	1%	360	340	600	560	160	GCCCTCACAGGCCCCAGGAAGCTGCTTTTCCTTTCC							27%	25%	27%
DKK1-NM_012242.4eU125-5'UTR-31	4	11%	5%	14%	1%	1	1%	3	2%	0%	310	170	530	420	350	CCCAGGCTTGCAAAGTGACGGTCATTTTCTCTTTCT	14%	13%	14%	5%		1%	2%	2%	3%
DLC1-NM_001164271.2eU421-NP_001157743.1aa46SF	0					0		2	3%	0%	480	130	550	260	170	GGTCCCGGCTTGAAGAGTTTGATGTCTTTTCTCCAA							3%		3%
DLG1-NM_001098424.1eU693-NP_001091894.1aa168PL	0					0		2	8%	2%	220	490	630	330	420	CACCAATAAAGCCAACAGAAGCTGTTCTTCCCTCTC							10%		7%
DLG3-NM_001166278.2eU1445-3'UTR+213	0					0		2	26%	6%	290	70	700	210	280	TTCAGTTTATTTTTTGGGATGATGCCATCTCATTCA								21%	30%
DLG5-NM_004747.4eU632-NP_004738.3aa133PS	0					0		2	13%	1%	190	460	730	310	300	AGTGTGGGCACTACCGGGAAGGCGCCGTCCCCACCA							14%		12%
DLGAP5-NM_001146015.2eU987-NP_001139487.1aa288SL	3	3%	2%	3%	2%	0		3	38%	1%	320	210	840	140	270	CAAGTGGAATGAATCCAGATGGAGTCTTATCAAAAA	5%	2%	2%				38%	39%	36%
DMTF1-NM_001142326.2eU3477-3'UTR+1077	0					0		3	10%	2%	440	250	720	300	210	TGTAATTAGATCTTCACAAAGTTGTCTTTTCACTGT							11%	10%	8%
DNAJA1-NM_001314039.2eU1071-NP_001300968.1aa219YY	0					0		3	13%	1%	310	200	650	280	250	TGATCCAAATCAGGAAAGACGGCGCCACTACAATGG							12%	14%	14%
DNAJB11-NM_001378451.1eU882-NP_001365380.1aa235PL	0					0		2	2%	1%	420	350	610	430	100	CTTTGATAATCACTTTTGATGTGGATTTTCCAAAAG							2%	2%	
DNAJB12-NM_001002762.5eU1391-3'UTR+241	0					0		2	23%	3%	150	340	670	220	460	GGTTTCCGCCCTCTAGCGATGCTGTCCTCCCCACTC							21%	25%	
DNAJB1-NM_001300914.2eU1104-NP_001287843.1aa223PL	4	24%	5%	26%	1%	1	1%	3	2%	0%	310	360	700	320	210	ACCTCATTATTGAGTTTGAAGTGATCTTCCCCGAAA	26%	27%	25%	16%	1%		1%	2%	2%
DNAJB1-NM_001300914.2eU1649-3'UTR+490	0					0		3	6%	1%	310	110	610	340	310	TTGCTCTTCTCCTGGAGAATTCTGTCTTCTCTTTGG							6%	6%	7%
DNAJB1-NM_001300914.2eU2108-3'UTR+949	0					0		3	15%	2%	220	190	650	40	500	TTATAATTCCATGTATAGTTGGTGTACACTCAAAAC							13%	15%	17%
DNAJB1-NM_001300914.2eU2137-3'UTR+978	0					0		3	2%	1%	410	120	490	250	440	TCAAAACCTGTCCCCGGCAGCCAGTGCTCTCTGTAT							1%	2%	3%
DNAJB2-NM_001039550.2eU729-NP_001034639.1aa180TT	0					0		2	3%	0%	450	70	590	520	40	TACCACCTTTGTCCAAGGACGCCGCATCACCACACG							3%	3%	
DNAJB4-NM_001317099.2eU717-NP_001304028.1aa183R*	0					0		3	29%	3%	360	180	670	290	240	AGTGGTTGTACCAAACGGATGAAGATTTCTCGAAAA							27%	29%	32%
DNAJC13-NM_001329126.2eU1462-NP_001316055.1aa397SL	4	20%	6%	22%	2%	0		2	20%	9%	280	200	830	180	480	TACATGCAGTAACACAAGATGGTCTCTTCTCAGAAA	21%	25%	21%	11%			26%		14%
DNAJC21-NM_001012339.3eU665-NP_001012339.2aa135PL	0					0		2	3%	2%	210	250	600	390	260	CTGTGTTAGAGGAAGAGGTTGATGATTTCCCAACTT							2%		5%
DNAJC3-NM_006260.5eU2805-3'UTR+1173	4	55%	23%	67%	2%	1	8%	3	23%	6%	320	90	760	250	330	GTTGTTTCATTTAATAAGATGGCATCTCCTCTTGAG	69%	65%	66%	21%	8%		27%	25%	16%
DNAJC9-NM_015190.5eU962-3'UTR+113	0					0		3	6%	1%	280	410	760	360	490	CAATTCAGCTCTTGAGAAAAGGTGTCTTTCCAGCCT							5%	5%	7%
DNM1-NM_001005336.3eU154-NP_001005336.1aa21SF	0					0		2	6%	2%	390	150	720	270	140	CGCTGGTCAACCGGCTGCAAGACGCCTTCTCTGCCA								8%	5%

DNMT3A-NM_001320893.1eU3294-3'UTR+974	0					0			3	25%	4%	490	160	840	190	300	AAGGAGACAGCTTGCAGATGGCGCGTTCTCCACGGT								23%	21%	30%
DNTTIP2-NM_014597.5eU867-NP_055412.2aa270SF	0					0			3	17%	0%	460	230	670	210	260	TCTATAATAATGACTTTGATGATGATTTCTCCCACA								16%	17%	17%
DOCK2-NM_004946.3eU2045-NP_004937.1aa665Q*	0					0			3	23%	9%	360	210	760	230	220	GCCCTCTTCAACATCATGATGGAGCATTCTCAAAGT								33%	23%	15%
DOCK2-NM_004946.3eU894-NP_004937.1aa281TM	0					0			2	18%	2%	430	150	660	470	340	AGATGCTCAACAATCTGAAGGTGGTCTTCACGGATC								17%	19%	
DPAGT1-NM_001382.4eU1265-NP_001373.2aa356TI	0					0			3	20%	9%	300	160	710	180	410	ACCAGAGTGAGACTGAAGATGGTGAATTCACTGAAT								16%	15%	30%
DPH1-NM_001346574.1eU1397-3'UTR+85	0					0			3	6%	1%	270	230	690	290	270	GGTTTTCAGAGCAGGAGGCCGACGTTTTCTCCGCAT								5%	6%	5%
DPM1-NM_001317034.1eU406-NP_001303963.1aa122SL	0					0			3	13%	2%	360	180	650	270	370	ACTACATCATTATTATGGATGCTGATCTCTCACACC								12%	15%	14%
DPM1-NM_003859.3eU374-NP_003850.1aa122SL	0					0			3	18%	4%	360	180	650	270	370	ACTACATCATTATTATGGATGCTGATCTCTCACACC								19%	21%	14%
DPM2-NM_001378437.1eU462-3'UTR+263	0					0			3	6%	2%	280	320	470	470	440	TGCTCAGGGCCTAAAAGATGCTGGTCCTCCCAACCT								8%	4%	7%
DPM2-NM_003863.4eU195-NP_003854.1aa54PL	0					0			3	3%	1%	250	140	620	230	140	TCCTGCCCCGAGCCTATGCTGTCGCCATCCCACTGG								2%	3%	3%
DPY19L4-NM_181787.3eU3655-3'UTR+1384	0					0			3	43%	10%	400	390	520	690	260	AAAGTACACTACATTAGAAGGGAGCTTTTCCGTTGT								55%	38%	38%
DRAM2-NM_001349881.2eU1220-3'UTR+74	0					0			2	9%	3%	420	200	620	300	240	TTGGGGAAAGGTTCACAGAAGTTGCTTATTCTTCTC								7%		11%
DSCC1-NM_024094.3eU498-NP_076999.2aa133PP	0					0			2	6%	2%	190	200	730	220	250	TAATTATTGGGAATTAAGAAGACGTAGACCCAAGTT									4%	8%
DSEL-NM_032160.3eU1303-NP_115536.2aa20SF	0					0			3	27%	4%	390	220	420	430	340	TATTCTTAGCATTATTGATGTTTGCTTTCTCTACTT								24%	26%	31%
DSG2-NM_001943.5eU150-NP_001934.2aa25HH	0					0			2	8%	5%	390	190	720	300	190	GATCTGCTTTAACGTTGGAAGTGGACTTCACTTACA								5%	12%	
DTL-NM_001286229.2eU1960-NP_001273158.2aa412Q*	0					0			3	8%	1%	280	180	590	430	330	GAGAATCCATCTCCACGAAGTCCGTCATCCCAGACA								9%	7%	9%
DTL-NM_001286229.2eU2231-3'UTR+125	0					0			3	24%	5%	380	250	660	470	180	TAACTCTGGTCTTTAAGAAAGCTGCCTTTTCATTTT								26%	18%	28%
DTX3L-NM_138287.3eU2508-3'UTR+196	0					0			2	4%	0%	310	90	800	140	410	GATACTTTTTTTTTTGAGACGGAGTCTGCTCTGTCG								4%		4%
DTX3L-NM_138287.3eU3343-3'UTR+1031	0					0			3	3%	1%	300	330	750	390	450	TTTGTATTTTTAGTAGAGACGGGGTTTTGCCATGTT								2%	3%	2%
DUSP11-NM_003584.2eU399-NP_003575.2aa119SS	0					0			3	5%	1%	620	440	390	650	240	AAAGAAACTTGCTCCAGAAGAATGCTTTTCCCCTTT								5%	4%	5%
DUSP14-NM_007026.4eU819-NP_008957.1aa191HY	0					0			2	2%	0%	300	220	710	150	430	GTTCCCGACGTCTATGAGAAGGAGTCCCGACACCTG								2%		2%
DYNC1LI2-NM_001286157.2eU2496-3'UTR+1230	0					0			2	5%	1%	320	240	650	270	310	TTATGGAAATAAGAGTTTCAGGTGTCTCCCCACCTA									6%	4%
DYNC2I2-NM_052844.4eU566-NP_443076.2aa149LL	3	2%	0%	2%	0%	0			3	27%	1%	310	160	790	140	330	TGGACCGAGCAGCAGCAGATGGTGTCTTGTCTGTAT	2%	2%	1%					26%	28%	27%
DYNLL1-NM_001037494.2eU780-3'UTR+241	0					0			3	9%	1%	460	130	650	210	330	TATTCCATACTTCTGCCCACGTTGTTTTCTCTCAAA								8%	9%	9%
EAPP-NM_001318916.2eU670-3'UTR+177	0					0			3	6%	0%	340	110	650	360	150	AGGCGGGTCCATAAGAAGATGAGGTCTAACCGGGAA								6%	6%	6%
EARS2-NM_001083614.2eU2069-3'UTR+490	0					2	9%	4%	3	7%	1%	330	190	710	340	310	CTTTTGTTGGCTTGCAGACAGCTGTCTTCTCACTGT					6%	12%		7%	6%	7%
EBNA1BP2-NM_001159936.1eU399-NP_001153408.1aa84R*	0					0			3	4%	1%	170	500	680	230	520	ACAGACAGAGAGTTGCAGGATGCGTTTTCCCGAGGG								4%	5%	2%
EBNA1BP2-NM_006824.3eU257-NP_006815.2aa29R*	0					0			3	2%	1%	170	500	680	230	520	ACAGACAGAGAGTTGCAGGATGCGTTTTCCCGAGGG								1%	2%	2%
EDC4-NM_014329.5eU2414-NP_055144.3aa735SL	4	36%	13%	42%	6%	1	9%		3	9%	4%	280	0	730	210	170	GCCGCACTCGTTCCCCTGATGTCATCTCCTCAGCTT	47%	35%	44%	19%			9%	14%	6%	7%
EEF1A1-NM_001402.6eU2834-3'UTR+1382	0					0			2	13%	7%	410	300	710	430	370	TTGGTGTTTTAATAGAGATGAGGTTTTTTCCATGTT								8%		18%
EEF2KMT-NM_201400.4eU278-NP_958802.1aa74LL	0					0			2	5%	0%	460	220	550	300	380	GCCGTCCGTCAAATATGCCCGGTGCTTTCTCTCAGA								4%		5%
EGFR-NM_001346899.2eU4845-3'UTR+1086	0					0			3	23%	4%	430	310	560	420	430	GTTTGTGTTACTTATGGAAGATAGTTTTCTCCTTTT								20%	23%	28%
EHD2-NM_014601.4eU2843-3'UTR+1039	0					0			3	3%	1%	350	260	690	460	410	TTTTGTATTTGAGTAGAGACGGGGTTTCACCATGTT								2%	3%	4%
EIF1AD-NM_001242481.2eU2317-3'UTR+1455	0					0			3	23%	25%	300	330	750	390	450	TTTGTATTTTTAGTAGAGACGGGGTTTTGCCATGTT								8%	10%	52%
EIF2AK2-NM_001135651.3eU1767-NP_001129123.1aa504SL	4	21%	9%	26%	1%	0			3	5%	2%	220	160	760	160	370	TTTTCACAGACCTACGGGATGGCATCATCTCAGATA	26%	26%	25%	8%				6%	7%	3%
EIF2AK2-NM_001135651.3eU882-NP_001129123.1aa209SL	4	6%	3%	7%	1%	0			3	47%	4%	270	240	850	140	280	CTTCTGAATCATCATCTGAAGGTGACTTCTCAGCAG	7%	6%	8%	2%				45%	46%	52%
EIF2D-NM_001201478.2eU289-NP_001188407.1aa56HY	0					0			2	5%	1%	420	80	760	140	310	GAGGAGCTCAACATTGTGAAGTTGTATGCTCACAAA								4%		6%
EIF2S3-NM_001415.4eU110-NP_001406.1aa33HY	4	10%	4%	12%	1%	0			2	1%	1%	310	130	640	300	250	TTGGATGTTACCAAGTTGACGCCACTTTCACACGAA	12%	11%	12%	4%				1%		2%
EIF4B-NM_001300821.3eU1071-NP_001287750.1aa348SF	0					0			3	3%	1%	400	140	650	400	260	CTCGGAGTACTCCTAAGGAAGATGATTCCTCTGCTA								2%	3%	3%
EIF4B-NM_001300821.3eU301-NP_001287750.1aa91PP	0					0			3	5%	1%	220	350	790	240	340	GGAACCCAATATCGACCGGAGCCGTCTTCCCAAATC								5%	6%	6%
EIF4E2-NM_001276336.2eU2706-3'UTR+1965	0					2	21%	1%	3	16%	7%	300	400	590	300	210	TGTCGTTGTAGTATTTGACAGTTGTCTTTTCCATCA					21%		20%	9%	21%	19%
EIF4E-NM_001130679.3eU113-NP_001124151.1aa31PL	0					0			3	4%	2%	320	130	550	510	260	AAACGGAATCTAATCAGGAGGTTGCTAACCCAGAAC								3%	3%	7%
EIF4G1-NM_001194946.2eU3101-NP_001181875.2aa981RC	3	2%	0%	2%	0%	0			3	48%	2%	340	220	790	220	330	AAAATCATTAAAGAAAAGAAGACGTCATCCCGCATC	2%	1%	2%					49%	46%	49%
EIF4G1-NM_001194946.2eU971-NP_001181875.2aa271PS	0					0			3	2%	0%	320	170	600	150	140	CCTTCAGAATCCCAGCCTTCGTCGCCTTCTCCGACC								2%	2%	2%
EIF5B-NM_015904.4eU3036-NP_056988.3aa957PL	0					0			3	2%	0%	230	220	640	220	320	TCCTTGTGGCTTATAAAGAAGATGAAATCCCTGTTC								2%	1%	2%
EIF5-NM_001969.5eU2403-3'UTR+666	0					0			3	9%	2%	230	330	660	370	540	CAGGATTTGAAACTTGGAGAGCTGTTTTCTCATTTC								8%	8%	12%
ELAVL1-NM_001419.3eU543-NP_001410.2aa127SF	4	17%	6%	20%	2%	0			2	2%	0%	490	150	770	140	290	TGACCCAGAAGGACGTAGAAGACATGTTCTCTCGGT	22%	18%	19%	8%				2%	2%	
ELAVL1-NM_001419.3eU545-NP_001410.2aa128RW	0					0			3	2%	0%	210	380	490	150	770	ACCCAGAAGGACGTAGAAGACATGTTCTCTCGGTTT								2%	2%	2%
ELAVL1-NM_001419.3eU904-NP_001410.2aa247FF	0					0			2	2%	0%	340	220	610	270	180	AGGAAACGCCTCCTCCGGCTGGTGCATTTTCATCTA								2%		2%
ELL2-NM_012081.6eU1077-NP_036213.2aa316SF	0					0			3	4%	1%	530	60	640	290	330	CTGTATGTTCTAGTAGAGACGCTGTATCTTCTCCTC								6%	5%	3%
ELL2-NM_012081.6eU2235-3'UTR+182	0					0			3	12%	1%	360	320	570	280	230	TTGCTTCCCTGCATTTCGAAGCTGCTCTTTCTGGTC								11%	12%	14%
ELL-NM_006532.4eU3096-3'UTR+1215	0					0			2	9%	4%	330	350	530	410	560	GGGCATGGTGGGGTCTGAGGCGTGTGTTCCCATTTA								7%		12%
ELMOD2-NM_153702.4eU3970-3'UTR+2966	0					0			3	33%	9%	230	210	780	210	350	TGAAATAACAGGGACCAGCAGCAGTTTTCTCAGGAT								43%	26%	30%
ELOVL5-NM_001242828.2eU2728-3'UTR+1605	0					0			2	3%	1%	330	210	680	140	220	TTGCTAGGTACTAATTTGCAGATGTCTTTACATATT								3%		2%
ELP2-NM_001242875.3eU837-NP_001229804.1aa268LF	0					2	12%	1%	3	45%	4%	530	340	700	220	420	CAAAATGATCAGTTTCAGAAAGTGCTTTCTCTCTGT					11%	13%		48%	40%	46%
ELP2-NM_001324465.2eU642-NP_001311394.1aa203LF	0					3	10%	1%	3	37%	6%	530	340	700	220	420	CAAAATGATCAGTTTCAGAAAGTGCTTTCTCTCTGT					9%	12%	10%	30%	43%	37%
EMC10-NM_175063.6eU1273-3'UTR+470	0					0			2	4%	4%	450	70	720	270	220	CCCATGCAGTCTGGGAACATGCCGCCTTCTCTCCAG								2%	7%	
EMC7-NM_020154.3eU325-NP_064539.1aa101PL	0					0			3	37%	4%	330	210	650	430	130	CTGGATCTTATGTAGTGGAAGTTGTATCTCCAGCTT								35%	35%	42%
EMSY-NM_001300942.2eU4924-3'UTR+760	0					0			3	8%	3%	320	70	690	270	210	TGCCTACGCTTTCCACTGTCGTCGTCTCCCCACTTT								5%	10%	9%
ENO2-NM_001975.3eU838-NP_001966.1aa249SL	0					0			2	7%	4%	350	60	590	350	260	AGATCGTTATTGGCATGGATGTTGCTGCCTCAGAGT								3%		10%
ENY2-NM_001193557.2eU315-NP_001180486.1aa84Q*	0					0			3	5%	1%	360	390	600	350	380	GTACCTGACAGTGTAAAGAAGGAGCTCCTACAAAGA								6%	3%	5%
EOGT-NM_001278689.2eU1416-NP_001265618.1aa317SL	0					0			3	45%	15%	340	270	790	330	350	AAAGGGTATGTTTTAAAGAAGCTGTTTTTTCATTAC								36%	63%	36%
EPAS1-NM_001430.5eU1569-NP_001421.2aa355SF	0					0			2	4%	0%	440	260	730	260	250	GTGAGATTGAGAAGAATGACGTGGTGTTCTCCATGG								4%	5%	
EPHB2-NM_001309192.2eU262-NP_001296121.1aa40PS	0					0			3	24%	8%	360	240	650	340	240	ACTGCTGAGCTGGGCTGGATGGTGCATCCTCCATCA								14%	27%	30%
EPHB4-NM_004444.5eU3776-3'UTR+319	0					0			2	10%	2%	520	270	510	500	360	GTGCCCCCCTCACCTTGATGGGTGCGTTCCCGCAGA								9%	11%	
EPN1-NM_001130071.2eU1037-NP_001123543.1aa165SL	0					0			2	2%	1%	320	70	680	190	180	CCGACCTCACCTACAACGTTGTCGCCTTCTCGGAGA								1%	2%	
EPRS1-NM_004446.3eU4122-NP_004437.2aa1335LL	0					0			3	45%	5%	270	230	780	210	280	AAAATGCAATGATTATCGAAGGCGATTACTCAGTGT								42%	42%	51%
EPS15L1-NM_001258374.3eU684-NP_001245303.1aa220PL	0					0			3	20%	8%	490	220	520	530	390	CACCCTCCAAGAGAAAGAAGACTGTGTTCCCTGGCG								19%	29%	14%
EPS8-NM_004447.6eU3450-3'UTR+732	4	7%	3%	8%	3%	0			3	39%	3%	320	200	700	60	600	AGCACAAATGCGAAAGAGGTCATGTCTTCTCAAAAT	5%	11%	7%	5%				38%	37%	42%
ERBIN-NM_001006600.3eU227-5'UTR-64	0					0			3	9%	4%	250	260	620	250	440	TGAAAAAGCTGCCCTGAGACGGTGTCCCGCCGAAAG								5%	9%	13%
ERC1-NM_001301248.1eU3762-3'UTR+345	0					0			3	10%	1%	520	100	660	410	120	TCTGCCTTTCTGCTTTGGATGTGGTCTCTACACTAA								11%	9%	11%
ERC1-NM_001301248.1eU4603-3'UTR+1186	0					0			3	5%	2%	420	220	660	180	270	GCAGCCCAGTGGCTGCTGAAGTTGTGATCCCTCACT								7%	5%	3%
ERC1-NM_001301248.1eU4896-3'UTR+1479	0					0			2	6%	3%	460	350	660	180	250	CTTCCCCTCCCCGATCTTAAGGTGTGTTTTCTAGAA								8%		4%
ERCC6L2-NM_001010895.4eU146-5'UTR-204	0					0			3	24%	8%	190	190	650	310	210	GCTCTGCCGCCGCTCCGGACGTCGCCCTCCCGTTCT								30%	14%	27%
ERCC6L2-NM_001375291.1eU3805-NP_001362220.1aa1153Q*	0					0			2	20%	2%	270	210	820	250	270	CATGACGTATTTGAGTTGAAGCAGTTTTCTCAGCTG								18%		21%
ERGIC1-NM_001031711.3eU268-NP_001026881.1aa35FF	0					0			3	2%	1%	390	60	560	300	60	GGCCATTATCTCCATCTGCTGCTGCCTCTTCATCCT								2%	3%	2%
ERI2-NM_001142725.2eU1008-NP_001136197.1aa322HH	0					0			2	10%	5%	330	160	660	310	460	AGTAAAAAACAATATAAAAGCAAGTCTTCACAATGT								7%		14%
ERN1-NM_001433.5eU6075-3'UTR+3026	0					0			3	15%	0%	350	260	760	460	510	ATATTTTTTTTAGTAGAGACGGGGTTTCCCCATGTT								16%	15%	15%
ERP44-NM_015051.3eU950-NP_055866.1aa264LF	0					0			3	5%	4%	690	290	440	360	470	GAGGAATTGACAGAAGAAGGACTGCCTTTTCTCATA								3%	3%	9%
ETS1-NM_001143820.2eU915-NP_001137292.1aa277PL	0					0			3	21%	3%	250	150	780	290	250	ACTTTGCTATCAAACAAGAAGTCGTCACCCCAGACA								22%	24%	18%
ETV3-NM_001145312.3eU2622-3'UTR+990	0					0			3	8%	4%	410	320	760	360	150	GGCATGACAACATTATTGAAGAGGTCTTTCCATTAT								4%	9%	12%
EVC-NM_153717.3eU5531-3'UTR+2372	0					0			2	18%	1%	400	260	690	460	410	TATTTTATTTTAGTAGAGACGGGGTTTCACCATACT								19%	18%	
EWSR1-NM_001163285.2eU2232-3'UTR+195	0					1	2%		2	2%	0%	300	350	540	270	400	GTTAAATGGTAGTGTGCGGAGTTTTTTTTTCTTCCT						2%		2%		2%

EXD2-NM_001193360.2eU235-NP_001180289.1aa4Q*	0					0		2	11%	3%	300	80	700	140	180	T A A A G A G T A G A A A A G T C G A A G A T G T C T A G A C A G A A C						13%	8%	
EXOC3-NM_007277.5eU1736-NP_009208.2aa522PL	0					0		3	12%	2%	490	270	640	480	330	T A A A G A A T G A A G T G G A A G A G G G T G T G T C T C C G A G C C						11%	10%	15%
EXOSC1-NM_001318363.2eU173-NP_001305292.1aa51PL	0					0		2	5%	3%	340	380	540	470	230	T G A T G A A G A G C A G C G A G A A T G G C G C G C T T C C A G T G G						4%	7%	
EXOSC9-NM_001034194.2eU1085-NP_001029366.1aa328TI	0					0		3	13%	5%	430	200	690	320	320	A A G C A G A A C C T C C T T C A G A A G T T G T T T C T A C A C C T G						10%	10%	19%
EXOSC9-NM_005033.3eU1085-NP_005024.2aa328TI	0					0		3	22%	10%	430	200	690	320	320	A A G C A G A A C C T C C T T C A G A A G T T G T T T C T A C A C C T G						13%	19%	33%
EXT1-NM_000127.3eU358-5'UTR-424	0					0		3	26%	9%	250	220	720	290	200	A G A G T G G T G G A T G A G T T G A A G T T G C C T T C C C G G G G C						18%	26%	35%
EXT2-NM_000401.3eU1039-NP_000392.3aa328SF	0					0		2	11%	2%	400	270	720	210	440	A A T G C A C C A A C C T C T C A G A G G G T G T C C T T T C T G T C C						13%		10%
EZH2-NM_001203247.2eU821-NP_001190176.1aa229SL	4	30%	10%	35%	3%	0		3	3%	1%	280	190	720	320	230	C T T C T G A T A A A A T T T T T G A A G C C A T T T C C T C A A T G T	38%	32%	34%	15%		4%	2%	4%
EZR-NM_001111077.2eU327-NP_001104547.1aa68Q*	4	1%	1%	2%	0%	0		3	31%	3%	210	240	850	280	390	T G G C T G A A G C T G G A T A A G A A G G T G T C T G C C C A G G A G	1%	2%	1%	1%		29%	34%	29%
EZR-NM_003379.5eU344-NP_003370.2aa68Q*	1	8%		8%		0		3	25%	8%	210	240	850	280	390	T G G C T G A A G C T G G A T A A G A A G G T G T C T G C C C A G G A G	8%					28%	32%	17%
F8A2-NM_001007523.2eU1518-3'UTR+382	0					0		2	20%	1%	330	280	610	310	290	G T G A G G A T T T T A T T T T G C A C G G C G C C C T C C C T C T A A						20%	19%	
FAHD2A-NM_016044.3eU235-NP_057128.2aa22Q*	0					0		2	7%	0%	410	350	710	250	190	G T T C T G C T G C A G G C T C A G A A G T G G C C C T T T C A A C C C						6%		7%
FAHD2B-NM_001320848.2eU378-NP_001307777.1aa22Q*	0					0		2	13%	2%	410	350	710	250	190	G C T C T G C T G C A G G C T C A G A A G T G G C C C T T T C A A C C C						11%		15%
FAH-NM_000137.4eU124-NP_000128.1aa23SL	0					0		3	18%	11%	270	210	780	140	290	C C A T C C A C A A C C T G C C C T A C G G C G T C T T C T C G A C C A						16%	30%	7%
FAM120AOS-NM_001322224.3eU663-NP_001309153.1aa53VV	0					0		3	28%	5%	210	310	720	190	380	G G T G A A A A A G A T T T C C A G A A G C T G T T C T G T C A A T A A						26%	24%	34%
FAM120AOS-NR_136230.2eU1125-ncr	0					0		3	12%	6%	210	310	720	190	380	G G G A T T A C A G A T T T C C A G A A G C T G T T C T G T C A A T A A						10%	19%	8%
FAM13B-NM_001101800.3eU867-NP_001095270.1aa123Q*	0					0		3	50%	2%	310	180	720	300	200	A G T T T A C A T A T T C A C T T G A T G C A G C T T T C T C A A G A T						51%	48%	52%
FAM162A-NM_014367.4eU335-NP_055182.3aa84PL	0					0		3	8%	3%	240	220	650	300	240	C A G G T C G C T T C A A A A A G G A A G A T G A A A T C C C A G A G A						10%	5%	11%
FAM168A-NM_001286050.2eU2966-3'UTR+2023	0					0		3	10%	4%	210	150	740	280	210	G G G T T G G A A G G A G C T C A G A A G C T G T C T A G C C A T T C C						15%	8%	9%
FAM204A-NM_001134672.2eU457-NP_001128144.1aa132Q*	0					0		2	6%	2%	390	270	650	300	240	T C A A A T G A A A C C C A G T G G A A A G A G C T T A C T C A G T A T							5%	7%
FAM204A-XR_001747175.1eU1079-ncr	0					0		3	22%	8%	330	260	570	260	270	C T C T T T G T C C C T T T C C T G A T G G T G T T A C T T C G A T T A						17%	32%	18%
FAM229B-NM_001033564.3eU315-NP_001028736.1aa4Q*	0					0		3	23%	5%	500	220	530	440	180	T C T T T T G G T T T T C A G T G A A G T A T G C C T T T T C A A T T T						18%	25%	27%
FAM32A-NM_014077.4eU518-3'UTR+146	0					0		3	9%	1%	480	150	670	280	230	G C T A C A G A C T G C T T T T C G A A G C T G T G T A C C C T C A T T						7%	10%	10%
FAM53C-NM_001135647.2eU3073-3'UTR+1446	0					0		2	2%	0%	240	130	640	240	250	C A G T G G A A A C A C A T A C T G A A G C T G C C A C C A C A A T A G						2%	2%	
FAM86B1-NM_001083537.4eU1207-3'UTR+304	0					0		3	33%	10%	240	230	650	440	340	A A A A C G T G T G T A T C A A G G A T G A C G T T C C C C C A A C G T						28%	45%	27%
FAM86B2-XM_037916389.1eU1384-3'UTR+304	0					0		2	23%	17%	240	230	650	440	340	A A A A C G T G T G T A T C A A G G A T G A C G T T C C C C C A A C G T						11%	36%	
FAM91A1-NM_001317917.2eU2730-NP_001304846.2aa578SL	3	6%	1%	6%	1%	0		3	65%	4%	320	160	840	180	370	A A A A C T T A A T A T T T A A A G A T G G T G T C T T A T C A G A A T	5%	6%	8%			64%	61%	68%
FANCA-NM_000135.4eU474-NP_000126.2aa148LL	3	15%	1%	15%	1%	0		3	40%	7%	250	220	820	320	390	A C T G T G G A G C A G A G A A A G A A G C T G T C T T C C C T G T T A	16%	13%	15%			39%	34%	47%
FANCM-NM_001308133.2eU2399-NP_001295062.1aa766SL	0					0		2	35%	21%	300	190	690	300	330	A A T C T T A T T T A C A A A T G G A A G A T G T T A C C T C A A C A T							20%	50%
FAR1-NM_032228.6eU652-NP_115604.1aa169PL	0					0		2	5%	1%	380	210	700	320	70	A T C G C A A G C A T A T T G A T G A A G T A G T C T A T C C A C C A C							7%	4%
FARP1-NM_005766.4eU2634-NP_005757.1aa738Q*	0					0		2	6%	1%	330	290	690	320	200	C A C G G T A C G A T G A T C A A G A T G G A G A A T T T C C A G A A G							7%	5%
FARSB-NM_005687.5eU714-NP_005678.3aa232SL	0					0		3	4%	1%	240	310	650	120	310	T T A T C T A T G A T A G C A A T G G T G T C G T C C T T T C A A T G C						3%	6%	4%
FASTKD1-NM_001281476.3eU1670-NP_001268405.1aa444PL	0					0		3	24%	11%	360	280	750	350	210	T G G G G A T A T C C C G A A T T G A A G C C G T T T T A C C A C A G T						37%	17%	18%
FASTKD2-NM_001136193.2eU1118-NP_001129665.1aa309TI	4	8%	5%	9%	4%	0		3	74%	1%	250	150	870	140	440	A G C A A G T T T G G A A A A T A G A A G A T G T C T T C A C A T T A C	14%	9%	5%	3%		73%	74%	75%
FASTKD2-NM_014929.4eU1025-NP_055744.2aa309TI	0					0		2	68%	2%	250	150	870	140	440	A G C A A G T T T G G A A A A T A G A A G A T G T C T T C A C A T T A C						67%	69%	
FASTKD5-NM_021826.5eU2556-NP_068598.1aa756HY	0					0		3	21%	6%	350	210	690	320	330	C G A A C T C G C T T A G A A A A G T T G G C G T T T C T T C A T G A G						15%	24%	25%
FAT1-NM_005245.4eU9324-NP_005236.2aa3038PL	0					0		3	21%	4%	300	330	680	220	180	A T T C A G A C A C T A T T C C T G A A G A C G T C C T T C C T G G A A						18%	22%	25%
FBXL5-XM_017008019.2eU446-5'UTR-712	0					0		3	28%	2%	520	320	690	500	410	T T T G T A T T T T T A T T A G A G A C G G G G T T T C T C C A C G T T						28%	31%	26%
FBXL7-NM_001278317.2eU2694-3'UTR+969	0					0		3	11%	3%	250	70	710	200	270	C C T C T T T T G C C A G G C T G G A T G C T G T C G T C T C T G T A C						8%	11%	15%
FBXO21-NM_015002.3eU1958-3'UTR+78	0					0		3	4%	0%	350	80	800	250	300	A G A A C G G G A C T C C G G A A G A A G A C G T C T C C A C G G A G C						5%	4%	4%
FBXO28-NM_001136115.3eU1492-3'UTR+933	0					0		3	39%	6%	370	280	720	300	210	C C A T A G T A T T G A C T G T A G A A G G A G C C T C T A C A A T A T						39%	34%	45%
FBXO34-NM_017943.4eU729-NP_060413.2aa201AV	0					0		2	5%	0%	320	150	650	260	230	A T G T G A G T G A C A G T G G G G A T G G A G T C T A T G C T G G G A						5%	4%	
FBXO38-NM_001271723.2eU1158-NP_001258652.1aa338SF	0					0		3	42%	7%	380	220	790	220	370	A G C C T T C C C T A A C C A A A G A T G G T G T C T T T T C T G C C C						34%	46%	45%
FBXO38-NM_030793.5eU2138-NP_110420.3aa665LF	0					0		2	6%	2%	400	210	670	420	230	C A T C A G A T G G G C C A G T C G A A G C A G T T T C C C C T C G A G						7%	5%	
FBXO38-XM_017009899.1eU1159-5'UTR-246	0					0		3	43%	12%	380	220	790	220	370	A G C C T T C C C T A A C C A A A G A T G G T G T C T T T T C T G C C C						30%	50%	50%
FBXO41-NM_001080410.4eU6972-3'UTR+3335	0					0		2	13%	3%	510	220	650	620	220	T C T A A G G C G C T A A G T T G A A G G A G G C C T T C C C A G A G T						11%		15%
FBXO7-NM_001033024.2eU803-NP_001028196.1aa237LF	0					0		2	8%	2%	320	90	670	170	390	C G C C T C T T T A A A G A C C A G C T G G T G T A T C C T C T T C T G						10%	6%	
FBXO7-NM_001033024.2eU864-NP_001028196.1aa257PL	0					0		2	4%	1%	190	190	600	310	400	T A C C A G A T G T A T T T G G G T T G G T C G T C C T C C C A T T G G						3%		4%
FCF1-NM_001318508.2eU1863-3'UTR+1153	0					0		3	18%	2%	310	200	670	350	270	C A C C A C G T C C A G C C A C T G A T G C T G T T T T G C C A C T G A						20%	18%	15%
FCGRT-NM_001136019.3eU101-5'UTR-5	0					0		2	7%	1%	210	160	720	240	400	G G A G C G A G G C T G A A G G G A A C G T C G T C C T C T C A G C A T						6%		8%
FCHSD1-NM_033449.3eU2163-3'UTR+40	0					0		2	9%	3%	230	250	560	340	380	A A G C C T T G A C C C C C A G T G A T G C T G C T G T C C C T A T C T						11%	7%	
FDFT1-NM_001287742.2eU2033-3'UTR+77	2	2%	0%	2%	0%	0		3	77%	1%	390	70	730	220	310	T T T T T T C T T T A A G G A T G G A T G T T G T G T T C T C T T T A T		3%	2%			75%	76%	78%
FERMT2-NM_001134999.2eU1191-NP_001128471.1aa351SL	0					0		3	5%	1%	370	180	630	210	170	A A G A A G T T G A T G A A G T T G A T G C T G C C C T T T C A G A C C						6%	3%	5%
FERMT2-NM_001134999.2eU1712-NP_001128471.1aa525RC	0					0		3	7%	3%	320	160	560	350	350	G A T A T A A C T C C T G A A T G T T T G G T G T C T C C C C G C T A T						7%	5%	10%
FERMT2-NM_006832.3eU1712-NP_006823.1aa525RC	0					0		3	7%	1%	320	160	560	350	350	G A T A T A A C T C C T G A A T G T T T G G T G T C T C C C C G C T A T						6%	8%	6%
FGF13-NM_001139501.2eU445-5'UTR-21	0					0		3	37%	12%	320	120	590	380	170	C C A C G T G G A A C T T T C T G G A T G A C G C C C C C C C T G G C A						27%	33%	50%
FIP1L1-NM_001134937.2eU1813-NP_001128409.1aa539R*	0					0		3	5%	2%	320	240	570	260	250	G A G A A A G A G G A A A C C A G A C A T A A G T C T T C T C G A A G T						4%	3%	7%
FKBP15-NM_015258.2eU2813-NP_056073.1aa927Q*	0					0		3	6%	1%	390	150	720	290	220	A T C A T G A A T A C G A T C A A G A T G G T G A C T C T T C A G C T G						6%	5%	6%
FKBP15-NM_015258.2eU582-NP_056073.1aa183SF	0					0		2	5%	0%	440	220	660	200	280	A C A G T A C C T C T T C C C T G G A T G C A G T G C T C T C C C A G G						5%		5%
FKBP9-NM_001284341.2eU3219-3'UTR+1213	0					0		3	4%	1%	500	270	500	460	250	T A T T T C C C T T G G C C G A G A T G G A A G T T T T G T C C C A A T						3%	3%	4%
FKTN-NM_001079802.2eU2914-3'UTR+1313	0					0		2	12%	3%	210	280	630	470	290	A T G T C T G A G T C A G C T A G G A T G C T G T T T A C C C C A T C T						14%		10%
FLNC-NM_001127487.2eU6644-NP_001120959.1aa2138Q*	0					0		3	2%	1%	210	160	810	240	390	C C A G G A A A C T G G T T C C A G A T G G T G T C T G C C C A G G A G						2%	3%	2%
FLOT1-NM_001318875.2eU635-NP_001305804.1aa154Q*	0					0		2	2%	1%	270	210	810	220	510	G C T A A A G C C A A G C A G G A A A A G G T G T C T G C T C A G T A C							1%	2%
FLYWCH1-NM_001308068.2eU3236-3'UTR+674	0					0		2	12%	5%	230	470	640	260	230	T C T C C A C G A T T C C C A C G G C A G G C G T C A T T C C C G A G A						15%	8%	
FMNL3-NM_001367835.1eU1156-NP_001354764.1aa311RW	0					0		2	19%	2%	330	290	660	280	190	C A C C G C T T T G A G A A G C T G A T G G A G T A T T T C C G G A A T						20%	18%	
FN1-NM_001306129.2eU7175-NP_001293058.2aa2303FF	0					0		3	2%	0%	420	250	730	280	110	C C A G T G C T T A G G C T T T G G A A G T G G T C A T T T C A G A T G						2%	1%	2%
FNDC3A-NM_001079673.2eU702-NP_001073141.1aa133PS	0					0		3	17%	5%	300	150	660	340	150	A T T C C T G T C C C A A C T A T G A T G C C G C C T C C A C C A C G T						20%	10%	20%
FOCAD-NM_001375567.1eU545-NP_001362496.1aa160Q*	0					0		3	15%	6%	360	390	660	310	250	G T G T T T T T G C A G C A G C T G A C A G C G T T T T T C C A G C A G						9%	20%	15%
FOSB-NM_001114171.2eU2277-3'UTR+777	0					1	9%	3	13%	4%	230	380	620	250	410	C C A G C C T T G T T T C T G A T G A C A G T G T T A T C C C A A G A C					9%	8%	17%	14%
FOSL2-NM_005253.4eU2210-3'UTR+366	0					0		3	5%	1%	220	440	640	220	440	C A T A C T G T C T C T G G A G T G A T G G T G T C C T T C C C T G C C						4%	6%	5%
FOXJ3-NM_001198850.2eU2091-NP_001185779.1aa607LL	0					0		3	7%	1%	460	180	670	410	220	C C T T C C C A A G C C T T C C A G A T G C G G C G T T C C C T G C C T						7%	7%	8%
FOXJ3-NM_001198850.2eU3248-3'UTR+1107	0					0		2	5%	0%	350	260	690	460	410	T T T T G T A T T T T A G T A G A G A T G G G G T T T C A C C A T G T T						5%		5%
FRA10AC1-NM_001347712.2eU623-NP_001334641.1aa68LL	4	14%	4%	14%	4%	0		2	15%	8%	220	240	800	140	470	G G A A G A A G C A A G A A A T A G A A G G T T T C A T C T C A T A G C	19%	13%	11%	13%		9%		20%
FRMD6-NM_001042481.3eU3424-3'UTR+1077	0					0		3	2%	0%	390	210	570	340	610	T A T A T G T A G A G T T C A G A A T G A C T G T T T T C T C A T G T G						2%	2%	2%
FRMD8-NM_001300832.3eU3373-3'UTR+2021	0					0		3	50%	7%	560	230	700	180	290	G T G T G T C T G A G T A C A C A G A T G A T G T G T T T T C C C T T C						43%	52%	57%
FRS2-NM_001042555.3eU4782-3'UTR+2791	0					0		3	14%	7%	390	230	680	330	400	T C T T C T A A A A A T G A A A A T A T G G T G C C T T C C C T C C C T						12%	22%	7%
FRYL-NM_015030.2eU3100-NP_055845.1aa839Q*	0					0		3	7%	1%	220	50	660	110	380	G C A T A C A C A A G A C T T C A G T T G T T G T C C C C T C A G G T C						8%	5%	7%
FSD1L-NM_001145313.3eU2892-3'UTR+1112	0					0		3	47%	22%	500	250	480	420	500	A G C A G C C A T T C A T T C A G A G G T T T G T T T T C T C T C A G T						24%	67%	50%
FXYD5-NM_001164605.2eU453-NP_001158077.1aa52TI	1	1%		1%		0		3	55%	2%	260	0	770	180	300	T C C C G A C A C G A G C C C C A G A T G C A G T C T A C A C A G A A C			1%			53%	58%	55%
FXYD5-NM_001320912.2eU105-NP_001307841.1aa10LL	0					0		2	4%	0%	330	160	700	60	350	C A T G T C G C C C T C T G G T C G C C T G T G T C T T C T C A C C A T						4%	4%	
FXYD5-NM_001320912.2eU230-NP_001307841.1aa52TI	0					0		3	56%	4%	260	0	770	180	300	T C C C G A C A C G A G C C C C A G A T G C A G T C T A C A C A G A A C						56%	52%	60%
FXYD5-NM_144779.3eU290-NP_659003.1aa52TI	0					0		3	52%	13%	260	0	770	180	300	T C C C G A C A C G A G C C C C A G A T G C A G T C T A C A C A G A A C						67%	45%	44%
FYTTD1-NM_001011537.3eU1595-3'UTR+458	0					0		2	4%	0%	380	130	570	190	220	G T G T T G A T G T A G T C A G T G C C G T T G C C T T T T C T T C A T							4%	3%

G3BP1-NM_005754.3eU2250-3'UTR+717	0					0			3	48%	0%	340	270	750	250	170	GAACTGTTAGTTTTTTTGAAGTAGTTTTTTCATGTT								48%	48%	48%
GALNT2-NM_001291866.2eU4335-3'UTR+2455	0					0			3	6%	2%	270	150	660	220	310	AGCAAAGCGTGCCGAAGTTAGTTGTCTTCTCTGTGC								5%	5%	9%
GALNT6-XM_011537819.3eU358-5'UTR-178	0					0			3	56%	11%	340	290	650	230	390	CGTGAGATTTCTTCACAGATGTTGCCCTCCCCCATT								68%	50%	50%
GANAB-NM_001278192.2eU3251-3'UTR+678	0					0			3	18%	2%	400	180	680	480	320	CCTCTGTCCCTCGGCAGAAAGACGTTTCCACACCTC								16%	20%	18%
GAS5-NR_002578.3eU95-ncr	0					0			3	3%	1%	300	240	560	430	350	TGGATAGCACCTTATGGACAGTTGTGTCCCCAAGGA								2%	3%	3%
GATD3B-NM_001363758.2eU962-3'UTR+140	0					0			3	6%	4%	310	350	700	110	420	TTTCTTTGCCGAAACCTGCAGGCGTTCTCTCTCTAA								3%	5%	11%
GCC1-NM_024523.6eU502-NP_078799.2aa30Q*	0					0			3	22%	6%	370	270	630	550	140	ACTATAGAGACCCAGAAGAAGCAGCTTCTCCAGTAC								29%	20%	17%
GCLC-NM_001197115.2eU1937-NP_001184044.1aa494PL	0					0			3	14%	5%	400	370	700	400	290	CCATCATCAATGGGAAGGAAGGTGTGTTTCCTGGAC								19%	13%	10%
GCN1-NM_006836.2eU1109-NP_006827.1aa364SL	0					0			3	10%	1%	330	290	590	290	480	CTGTTGTAGCCCAGAAGATGAGCGTCCTCTCAGGGA								9%	11%	10%
GCN1-NM_006836.2eU5756-NP_006827.1aa1913SF	0					0			2	3%	3%	420	360	490	360	490	CGTCCCTGCATGTCTGGAAGATTGTTGTCTCCAATA								1%		5%
GCN1-NM_006836.2eU8138-3'UTR+104	0					0			3	7%	2%	630	260	510	580	300	TCCAATGGGGAGCTTGGAAGATGGCGTTCCCAGAAA								4%	7%	9%
GCOM1-NM_001018090.6eU1726-NP_001018100.1aa532SL	0					0			2	4%	1%	560	270	710	330	160	TGCGGAGTTATAATCCAGAAGGGGAGTCTTCAGGGA									3%	5%
GEMIN6-NM_024775.10eU1046-3'UTR+451	0					0			2	29%	12%	390	260	640	460	410	TTTTGTATTTTAGTAGAGATGGGGTTTCACCTGTAT									38%	20%
GEMIN7-NM_001007269.2eU537-3'UTR+15	0					0			2	5%	0%	410	110	640	140	440	TACCTTCAAGCCATAAAGATATTGTGTTCACTTTTC									5%	4%
GEM-NM_005261.4eU1233-3'UTR+103	0					0			3	12%	4%	350	320	670	460	310	ATTGAATACTTAAGTTAGATGTGGTTTCCCCCATTG								15%	14%	7%
GFM1-NM_001308164.2eU2135-NP_001295093.1aa676PL	0					0			2	5%	3%	320	260	580	370	130	AACCTATTATGGCTGTGGAAGTTGTAGCTCCAAATG								3%	6%	
GFPT1-NM_001244710.2eU2222-NP_001231639.1aa689PL	0					0			3	15%	1%	310	200	680	350	260	CTGTGCTGAGAGGCTATGATGTTGATTTCCCACGGA								14%	16%	17%
GFPT1-NM_002056.4eU870-NP_002047.2aa238LL	0					0			3	11%	1%	390	150	640	290	240	AGGCAAAGACAAGAAAGGAAGCTGCAATCTCTCTCG								12%	11%	10%
GFRA1-NM_001145453.4eU5280-3'UTR+3198	0					0			3	50%	6%	470	190	680	250	210	GTAGTAAGAATGTCTTAGAAGTTGTGTCTTCGAGTT								47%	57%	47%
GFRA1-NM_001145453.4eU7868-3'UTR+5786	0					0			3	12%	6%	310	70	770	180	430	ATTTCATGTCCTTCGGAGACGCTGTATTCTCTTTAC								9%	7%	19%
GFUS-NM_001317783.2eU279-NP_001304712.1aa55TM	0					0			2	5%	2%	200	180	640	230	440	TGTTTGTCTCCTCTAAAGACGCCGATCTCACGGATA								6%	3%	
GFUS-NM_003313.4eU233-NP_003304.1aa49TM	0					0			2	7%	0%	200	180	640	230	440	TGTTTGTCTCCTCTAAAGACGCCGATCTCACGGATA								7%	8%	
GGA3-NM_001172703.3eU338-NP_001166174.1aa27PL	0					0			2	12%	8%	380	300	660	430	150	TTTTGAATGAGTTAATCAAAGTCGTCTCTCCAAAGT								7%		18%
GGCX-NM_000821.7eU1281-NP_000812.2aa406RC	0					0			3	16%	7%	290	280	600	270	350	GGCTATTCCTGGGACATGATGGTGCACTCCCGCTCC								9%	15%	24%
GID8-NM_017896.3eU2359-3'UTR+1553	0					0			3	17%	2%	420	260	750	180	250	CTGTGTTGGAGCCTGGCGAAGTTGTGTTTTCAAGCC								15%	18%	18%
GINM1-NM_138785.5eU822-NP_620140.1aa261PL	0					0			2	3%	1%	290	360	530	390	480	ATCTGTGTAGGTTCTGGAGCAACGTTTTCCCAGTAT								2%		3%
GINS1-NM_021067.5eU2422-3'UTR+1683	0					0			3	4%	1%	350	260	640	460	410	TTTGTATTTTTTGTAGAGATGGGGTTTCACCGTGTT								4%	4%	3%
GLCE-NM_001324094.2eU1016-5'UTR-340	0					0			3	24%	8%	300	190	720	360	460	CAGCAACTTTTTGCAGGGAAGATGTTTCCACAACCA								20%	33%	20%
GLCE-NM_001324094.2eU534-5'UTR-822	0					0			2	28%	4%	320	190	660	180	560	AACAATCGTCATTTTGAAGTGTTGTCTTCTCTTACT								30%	25%	
GLDC-NM_000170.3eU3078-NP_000161.2aa950HY	0					0			2	40%	5%	400	80	770	210	280	CCCAGGGTCAATCCGCTGAAGATGTCTCCACACTCC									36%	43%
GLIPR2-NM_001287011.2eU129-NP_001273940.1aa28PL	0					0			3	19%	5%	300	210	660	370	360	ATGAGTACCGGCAGAAGCACGGCGTCCCCCCACTGA								23%	14%	21%
GLOD4-NM_001366247.2eU684-NP_001353176.1aa220SF	0					0			3	4%	2%	390	310	550	190	490	ATGCAGCAGCTTTTGGAAGAATTGCCTTCTCTTGCC								2%	4%	7%
GM2A-NM_000405.5eU1517-3'UTR+903	0					0			3	16%	8%	350	260	690	460	410	TGTGTATTTTTAGTAGAGATGGGGTTTCACCATGTT								25%	11%	12%
GMNN-NM_001251989.2eU173-5'UTR-30	0					0			2	3%	1%	530	230	550	220	500	TGCGTTCGCTACGAGGATTGAGCGTCTCCACCCATC								2%		3%
GMNN-NM_015895.5eU252-5'UTR-83	0					0			3	6%	1%	350	360	500	280	530	GGCGCACAGCCTCTAGACGACTCGCTTTCCCTCCGG								7%	4%	6%
GMPPB-NM_013334.4eU821-NP_037466.3aa199PL	0					0			3	45%	9%	420	370	660	610	290	AGCCTACGTCCATTGAGAAGGAGGTCTTCCCCATTA								34%	51%	48%
GMPPB-NM_021971.4eU821-NP_068806.2aa199PL	0					0			3	42%	18%	420	370	660	610	290	AGCCTACGTCCATTGAGAAGGAGGTCTTCCCCATTA								22%	47%	57%
GNA11-NM_002067.5eU1899-3'UTR+529	0					0			3	8%	2%	460	400	500	600	250	GGCGTGGAGACTCGGAGACGGACGTTTTTCCCCTTT								7%	10%	7%
GNA11-NM_002067.5eU4134-3'UTR+2764	0					0			3	86%	2%	390	210	780	260	80	TAATCCAGACACCGATGGAAGTCGACTTTTCATATC								87%	84%	88%
GNB1-NM_001282538.2eU1072-NP_001269467.1aa185LL	0					0			3	2%	0%	400	190	700	310	420	TGTCTCCTTCTCCAAGAGCGGGCGCCTCCTCCTTGC								3%	2%	2%
GNB4-NM_021629.4eU5292-3'UTR+4102	0					0			2	5%	0%	410	240	590	190	420	GTCATCATTTTAATAGTGACGATGTTCTTTCTGAAA								6%		5%
GNG4-NM_001098721.2eU1159-3'UTR+749	0					0			3	27%	4%	350	260	690	500	330	TTTGTATTTTTAGTAAAGACGGGGTTTCACCATGTT								22%	29%	29%
GNPDA1-NM_005471.5eU1638-3'UTR+729	0					0			2	7%	0%	380	440	700	470	170	CCAAAAGGCAGAAGGTGGAAGCCGTTTTTCCCCCAG									7%	7%
GNPDA1-NM_005471.5eU2054-3'UTR+1145	0					0			3	6%	1%	360	100	660	380	160	TAGGCCTTCATGGAGTGGATGCTGCCTCCTCCTGGC								6%	6%	5%
GOLPH3L-NM_018178.6eU894-NP_060648.2aa240SF	0					0			3	7%	1%	470	210	730	190	390	ACTCCTCTGATGTGCTAGAGAATGTCTTCTCCTCTC								6%	8%	7%
GON4L-NM_001282856.2eU1263-NP_001269785.1aa370SL	0					0			3	22%	6%	410	140	740	320	300	TGGATATCCACCTTGAAGAAGATGATTCCTCAGATG								17%	20%	28%
GON4L-NM_001282856.2eU841-NP_001269785.1aa229FF	0					0			2	6%	1%	520	140	480	580	120	TGAGGAAGAGATAGAAGATGGTGGACTCTTCATTCC									5%	6%
GORASP2-NM_001201428.2eU1247-NP_001188357.1aa341SL	0					0			3	8%	2%	460	0	750	300	240	CAACTACTGCAAAGGCAGACGCTGCCTCCTCACTCA								6%	7%	10%
GOSR1-NM_001007024.2eU338-NP_001007025.1aa34SF	0					0			3	4%	1%	220	320	650	110	500	CAGAATATACCAACAGTGCAGGTGTCCCCTCCTTGA								5%	3%	3%
GOSR1-NM_001007024.2eU3836-3'UTR+3041	0					0			2	3%	0%	250	320	740	390	510	AGGGTACCCTTAAAAGAAAAGAAGTTTTCCCATTGT								4%		3%
GOSR1-NM_001007024.2eU5032-3'UTR+4237	0					0			3	58%	16%	400	140	710	360	330	CATACTTTCCTGTGATGGAAGATGTTTCCTCTTGAA								52%	46%	77%
GOSR1-NM_004871.3eU322-NP_004862.1aa99SF	0					0			2	10%	5%	220	320	650	110	500	CAGAATATACCAACAGTGCAGGTGTCCCCTCCTTGA								13%	6%	
GPD1L-NM_015141.4eU1122-NP_055956.1aa344LF	0					0			3	25%	9%	310	70	750	140	370	AGCAGACCAGTTCAAGAGATGTTGTCTTGTCTTCAG								19%	35%	20%
GPI-NM_000175.5eU2637-3'UTR+889	0					0			2	5%	1%	300	410	750	390	370	TTTGTATTTTTAGTAGTGAAGGGGTTTTACCATGTT								4%	5%	
GPI-NM_000175.5eU3722-3'UTR+1974	0					0			2	3%	0%	350	260	640	460	410	TTTGTATTTTTAGTAGAGACGGGGTTTCACCGTGTT									3%	3%
GPR107-NM_001136557.2eU4358-3'UTR+2528	0					0			2	2%	0%	390	160	730	190	470	TCTCCATCCTGTTCTGTGAGTGTGTCTTCTCTTTCT								2%	3%	
GRB2-NM_002086.5eU1973-3'UTR+992	0					0			2	3%	0%	390	190	630	370	230	CCAGTGGGATTATCGAGCATGTTGTTTTTTCATAGT								3%	3%	
GRB2-NM_002086.5eU474-NP_002077.1aa49PP	4	8%	4%	9%	3%	0			3	4%	1%	250	230	440	640	320	AGAGCTTAATGGAAAAGACGGCTTCATTCCCAAGAA	12%	6%	9%	3%				2%	4%	5%
GRINA-NM_000837.2eU642-NP_000828.1aa121PL	0					3	4%	1%	3	3%	2%	300	260	580	360	250	CCAACCCCTATGGACAGCCACAGGTCTTCCCAGGAC					3%	6%	3%	2%	3%	5%
GRSF1-NM_001098477.2eU875-NP_001091947.1aa238LL	0					0			3	6%	3%	350	110	650	260	330	GAGGCTGCTGATTTTGGAACTACGTCTTCTCTGCAT								3%	9%	7%
GRWD1-NM_031485.4eU1876-3'UTR+512	0					0			3	7%	2%	350	260	690	460	410	TTTGTATTTTTAGTAGAGACGGGGTTTCACCATGTT								7%	9%	6%
GRWD1-NM_031485.4eU3050-3'UTR+1686	0					0			2	17%	1%	170	220	660	200	480	CTTGTCCCCTTCAAGAAGGCCTTGTCTTCCCATCAG								17%		18%
GSDME-NM_001127453.2eU1521-NP_001120925.1aa478PL	0					0			2	5%	1%	390	180	540	200	210	CTGTCATTCTGAAGGACTCTAAAGTCTTCCCACTGC								6%		4%
GSTA4-NM_001512.4eU676-NP_001503.1aa208PS	0					0			3	23%	3%	340	250	700	400	180	CTTGAACCTGGCAGCAAGAAGAAGCCTCCCCCTGAT								20%	26%	23%
GSTCD-NM_001031720.3eU3255-3'UTR+1281	0					0			3	11%	4%	420	320	690	500	410	TTTGTAATTTTAGTAGAGATGGGGTTTCTCCATGTT								7%	11%	14%
GSTM4-NM_000850.5eU827-NP_000841.1aa179PL	0					0			3	8%	4%	450	250	540	380	160	TATTTGAGCCCAACTGCTTGGACGCCTTTCCAAATC								7%	4%	12%
GSTP1-NM_000852.4eU217-NP_000843.1aa62TI	0					0			3	5%	2%	250	310	650	200	340	AGCTCCCCAAGTTCCAGGACGGAGACCTCACCCTGT								3%	6%	6%
GTF2I-NM_001163636.3eU1007-NP_001157108.1aa217PP	0					0			3	5%	1%	280	150	650	330	230	AATACTATCTCCAGGTGGAAGTTGTGGCCCCATCAA								4%	4%	5%
GTF2I-NM_001280800.2eU1007-NP_001267729.1aa217PP	0					0			3	6%	3%	280	150	650	330	230	AATACTATCTCCAGGTGGAAGTTGTGGCCCCATCAA								3%	6%	8%
GTF2IP20-NR_132119.1eU773-ncr	0					0			2	39%	15%	350	220	770	210	560	TTTTTTTTTTTTTTTGAGATGGAGTTTTCACTCTTA								29%	50%	
GTF3C1-NM_001286242.2eU1978-NP_001273171.1aa652TI	0					0			3	6%	1%	390	320	750	330	300	TGGATCAGGAGAAGCAGGAAGGCGTGTCCACCAAGT								5%	7%	7%
GTF3C1-NM_001286242.2eU319-NP_001273171.1aa99PL	0					0			3	4%	1%	330	220	680	410	270	GGGACCCGGTGGCTTTGGAGGATGTCTACCCCATTC								3%	4%	5%
GTF3C3-NM_001206774.2eU1025-NP_001193703.1aa318SL	0					0			3	37%	6%	400	180	720	260	240	CTGCTATTAACATAATTGATGAAGCTTTCTCAAAAC								31%	42%	40%
GTF3C4-NM_012204.4eU2505-NP_036336.2aa790LL	0					0			3	12%	5%	270	380	790	210	490	CCAGAGTTTGATATATAGAAGGTGTTTGCTCCATGA								13%	7%	16%
GTF3C4-NM_012204.4eU4337-3'UTR+1733	0					0			3	8%	2%	540	360	520	600	330	TTGGTGGTAATTTCAAGAAGAGCGTTTATCCACTAT								9%	8%	6%
GTF3C4-NM_012204.4eU7845-3'UTR+5241	0					0			3	48%	2%	370	160	720	140	400	CCCCCCAATCCAGAAAAGTTGGTGACTTCTCTCCAT								46%	47%	50%
GTF3C5-XR_002956826.1eU118-ncr	0					0			2	6%	0%	300	250	650	440	350	AAAAAGTCGAGGACACGGCGGTCGTTTTCCCGAAGA								7%		6%
GTPBP4-NM_012341.3eU2170-3'UTR+219	0					0			3	4%	1%	340	190	670	240	500	TACAAATATTTGAGTAGACAGTGTTTCCACCATTTA								4%	4%	5%
GUSB-NM_000181.4eU1435-NP_000172.2aa469HY	0					0			2	2%	0%	360	200	670	190	450	GCTGGCTACTACTTGAAGATGGTGATCGCTCACACC								2%		3%
H3-NM_005324.5eU338-NP_005315.1aa69Q*	0					0			3	10%	1%	310	330	630	390	170	ACCGAGCTGCTCATCCGGAAGCTGCCCTTCCAGAGG								9%	10%	10%
HABP4-NM_014282.4eU1461-3'UTR+108	0					0			3	63%	3%	320	360	600	400	280	AGACTCTAAGAACAATAGATGTTGCTTTTCCCGTGT								64%	60%	66%
HABP4-NM_014282.4eU1462-3'UTR+109	1	2%		2%		0			3	8%	1%	300	320	360	600	400	GACTCTAAGAACAATAGATGTTGCTTTTCCCGTGTC		2%						9%	8%	7%
HACD3-NM_016395.4eU821-NP_057479.2aa218LL	0					0			2	2%	0%	360	150	640	190	330	GGAGTCACTACGTCACCGGTGCTGCCTTCTCTGATC									3%	2%

HADHB-NM_000183.3eU1010-NP_000174.1aa317AV	0					0		2	2%	1%	370	200	590	300	180	ATTCTTCTTTCTTGACTGATGGTGCATCTGCAATGT							2%		3%
HADHB-NM_000183.3eU1863-3'UTR+378	0					0		3	19%	3%	430	240	690	220	310	GTTCTTATCTGTGTCCTAAAGATGTGTTCTCTATAA							23%	17%	17%
HASPIN-NM_031965.2eU596-NP_114171.2aa188SL	0					0		2	7%	4%	290	250	660	120	470	GGTCCCCAACGCCAAGGGACAGTGTCATCTCGATCG							4%	10%	
HAX1-NM_001018837.2eU748-NP_001018238.1aa218SF	0					0		3	4%	3%	420	280	650	310	100	CAAGACCTCCAGCCCTGGATGATGCCTTTTCCATCC							3%	3%	7%
HCFC1-NM_005334.3eU6488-NP_005325.2aa1837PL	0					0		3	3%	1%	230	200	650	300	340	ACTATTTCCTGCCACCAGATGATGCTGTCCCATCAG							4%	2%	3%
HCFC1-NM_005334.3eU6491-NP_005325.2aa1838SL	0					0		2	3%	1%	310	50	630	230	200	ATTTCCTGCCACCAGATGATGCTGTCCCATCAGACG								2%	3%
HDAC2-NM_001527.4eU330-NP_001518.3aa30PP	0					0		3	2%	0%	320	310	670	300	330	TGGAAATTATTATTATGGACAGGGTCATCCCATGAA							2%	2%	2%
HDAC3-NM_001355039.2eU636-NP_001341968.1aa193HY	4	2%	1%	3%	1%	0		3	33%	1%	370	340	690	260	320	CTCACTGACCGGGTCATGACGGTGTCCTTCCACAAA	4%	3%	2%	2%			32%	33%	33%
HDAC8-NM_001166418.2eU1166-3'UTR+223	0					0		3	12%	5%	300	370	730	290	360	AACTGGACCTAGGGGAAGAAGGAGATATCCCACATT							18%	11%	8%
HDAC9-NM_001321868.2eU7648-3'UTR+4028	0					0		2	30%	4%	250	220	810	250	520	TGAACTATTTTCATAAAGATGGCGTTTTCACTTTCA							33%		27%
HDLBP-NM_001320967.3eU149-5'UTR-29	0					0		2	4%	0%	310	240	610	350	270	TGGCTACTGACCGTTTTGCTGTGGTTTTCCCGGATT							4%		4%
HEATR1-NM_018072.6eU1734-NP_060542.4aa536SL	0					0		3	17%	12%	270	130	700	210	350	TAGGTGATGATAATATAGATGTTGTTTTGTCGGCTA							29%	5%	18%
HEATR1-NM_018072.6eU3224-NP_060542.4aa1033Q*	0					0		2	13%	2%	310	220	800	260	500	CAGGGAGTCAACGGTGAGATGGTGCTTTCTCAGCTA								11%	14%
HEATR5A-NM_015473.4eU1922-NP_056288.2aa590PL	0					0		2	20%	12%	360	330	590	490	410	GAGTTCTGCTGTTGTGGAAGTGTGTCTTTCCAGCAT								11%	29%
HEATR6-NM_022070.5eU1158-NP_071353.4aa384SL	4	6%	3%	7%	3%	0		3	40%	9%	320	90	850	250	370	GTGGAGCTGCAGAAAAAGATGGAGTCTCCTCATCCT	7%	4%	10%	4%			34%	37%	50%
HECTD4-NM_001109662.4eU15299-3'UTR+1709	0					0		3	20%	7%	510	380	700	430	250	AGCCTTTCGATTTATTAGAAGTGGTTTTTCCTCCCT							20%	13%	27%
HECW1-NM_001287059.2eU3397-NP_001273988.1aa1025PP	1	9%		9%		0		2	63%	6%	250	230	710	370	490	CCGAATCCCTCTTCAGAACGGTCGTCTTCCCAATCA			9%				58%	67%	
HELZ2-NM_001037335.2eU4347-NP_001032412.2aa1152SL	0					0		2	2%	1%	400	0	660	340	160	CGTCAGCCATCCCGCTGGACGATGCCTCCTCGGGCC							3%		2%
HELZ-NM_001330447.2eU7285-3'UTR+1273	0					0		2	35%	8%	390	210	670	430	330	TTTGGTAAAATCCAAAGGAAGATGTTTACCCTCTTG							41%		29%
HIBADH-NM_152740.4eU901-NP_689953.1aa269SL	2	3%	0%	3%	0%	0		3	36%	3%	260	200	680	300	420	CTGTACCTGGAGTGATGGATGGCGTTCCCTCGGCTA		3%	2%				39%	34%	34%
HIF1AN-NM_017902.3eU2456-3'UTR+1390	0					0		2	7%	1%	320	280	640	360	80	GCAACTGGTGGAGCATGGAAGTCGAATTTCCTTTTC							7%	8%	
HINT3-NM_138571.5eU1315-3'UTR+558	0					0		2	8%	1%	290	170	440	460	440	TTTTCCTCAAATTTCAGATGCCAGTTTTCACTTTTT							8%		9%
HIP1-NM_001243198.3eU2590-NP_001230127.1aa857HY	0					0		3	11%	3%	350	200	740	220	390	GGGAAATTTGAGGAGCTAATGGTGTGTTCTCATGAA							8%	12%	14%
HIP1R-NM_003959.3eU3279-NP_003950.1aa1062PL	4	35%	11%	40%	4%	0		3	17%	5%	170	160	730	360	290	ACCAGCTTGACAAAAAGGATGGCATCTACCCAGCTC	43%	43%	35%	20%			14%	14%	23%
HIPK1-NM_001369806.1eU2688-NP_001356735.1aa864Q*	0					0		3	13%	3%	210	270	520	250	490	TCCAAGTCCTCTCTAGATGTTCTGCCTTCCCAAGTC							17%	11%	12%
HIPK1-NM_001369806.1eU3928-3'UTR+197	0					0		2	4%	0%	220	190	710	200	250	TCAGTGTTGACTGCATTGTTGTAGTCTTCCCAAAGT								3%	4%
HIPK3-NM_001048200.3eU5440-3'UTR+1344	0					0		3	15%	5%	320	270	650	340	250	GTTATGTCTGTTTAAATGAAGTTGCTTTTACAGCAC							10%	20%	15%
HIVEP2-NM_006734.4eU494-5'UTR-226	0					0		2	19%	5%	410	210	490	530	340	TTGCACTTAGCTTTTGGATGGACGTTTCTACAATGG							23%	16%	
HLA-NM_005516.6eU921-NP_005507.3aa300Q*	0					0		3	2%	0%	210	300	810	360	180	CCCGTCACCCTGAGATGGAAGCCGGCTTCCCAGCCC							1%	2%	2%
HLA-XM_041680767.1eU899-XP_041536701.1aa300Q*	0					0		3	9%	1%	170	300	790	320	340	CCCCTCACCCTGAGATGGGAGCCGTCTTCCCAGCCC							8%	9%	10%
HM13-NM_030789.4eU916-NP_110416.1aa269PL	0					0		3	6%	2%	220	200	730	220	270	CCATGCTGGGACTTGGAGATGTCGTCATTCCAGGGA							4%	8%	5%
HMGB1-NM_001313892.2eU1143-3'UTR+314	2	1%	0%	1%	0%	0		3	40%	2%	380	230	550	460	330	ATTAGTTATATATGGGGATGGTAGTTTTTTCATCTT		1%	1%				41%	40%	38%
HMGN4-NM_006353.3eU1429-3'UTR+940	0					0		3	12%	3%	340	250	700	230	270	TAACATTTTTCATATTCGAAGTGGTTGTCTCTGCAC							9%	13%	14%
HNRNPA0-NM_006805.4eU310-NP_006796.1aa5Q*	2	1%	0%	1%	0%	0		3	21%	2%	270	160	800	140	380	AAGGCCCGGGCCTTGGAGATGGAGAATTCTCAGTTG	1%	2%					19%	23%	21%
HNRNPA2B1-NM_002137.4eU1926-3'UTR+731	0					0		3	17%	3%	430	170	630	410	200	CCTTTTTAGATCTTTTAGATGCTGCTTCTTCAATGC							14%	19%	17%
HNRNPA2B1-XM_017012109.2eU1266-3'UTR+43	0					0		2	35%	10%	430	170	630	410	200	TTGCCATGGATCTTTTAGATGCTGCTTCTTCAATGC							28%		42%
HNRNPA3-NM_001330247.1eU2117-3'UTR+940	0					0		3	2%	0%	430	200	610	440	410	AATTCTGAAAAGGGATGGTGGATGTTTTCCCTCTCC							2%	2%	2%
HNRNPC-NM_001077442.2eU785-NP_001070910.1aa202Q*	0					0		3	4%	0%	240	300	670	340	320	GACCTTCAGGCCATTAAGAAGGAGCTGACCCAGATA							4%	4%	4%
HNRNPC-NM_001077443.2eU746-NP_001070911.1aa189Q*	0					0		2	4%	0%	240	300	670	340	320	GACCTTCAGGCCATTAAGAAGGAGCTGACCCAGATA								5%	4%
HNRNPC-XM_006720125.3eU690-5'UTR-212	4	31%	5%	33%	3%	0		2	5%	1%	260	290	690	310	430	TTAACACTTATGATCGTGATGGCATTTTCCCTGAAA	30%	34%	36%	24%			6%	4%	
HNRNPD-NM_001003810.2eU1226-3'UTR+49	0					0		3	2%	0%	390	360	700	220	190	TATCCCCAACAGGTGGTGAAGCAGTATTTTCCAATT							2%	2%	2%
HNRNPD-NM_031369.3eU1373-3'UTR+49	0					0		3	4%	2%	390	360	700	220	190	TATCCCCAACAGGTGGTGAAGCAGTATTTTCCAATT							6%	3%	4%
HNRNPH1-NM_001257293.2eU1115-NP_001244222.1aa336AV	0					0		3	4%	1%	290	120	760	140	220	GAGTAACTGGTGAAGCAGATGTCGAGTTCGCAACTC							3%	4%	4%
HNRNPH3-NM_001322434.2eU1353-3'UTR+117	0					0		3	7%	2%	630	340	500	270	500	AACTTTATAATGACTGAAGGAATGTGTTTTCAAAAT							8%	8%	5%
HNRNPH3-NM_001322434.2eU808-NP_001309363.1aa205RC	0					0		3	2%	1%	420	250	490	480	270	CATTTCGTACATATGAGAGGGTTGCCTTTTCGTGCA							2%	1%	3%
HNRNPK-NM_001318186.2eU202-NP_001305115.1aa11PL	4	22%	9%	26%	3%	1	1%	3	2%	1%	340	270	680	290	220	TGGAAACTGAACAGCCAGAAGAAACCTTCCCTAACA	30%	25%	25%	10%		1%	2%	3%	2%
HNRNPK-NM_002140.5eU252-NP_002131.2aa11PL	4	23%	9%	27%	3%	1	1%	3	2%	0%	340	270	680	290	220	TGGAAACTGAACAGCCAGAAGAAACCTTCCCTAACA	30%	27%	25%	10%		1%	1%	2%	2%
HOOK3-NM_032410.4eU3743-3'UTR+1432	0					0		3	62%	5%	390	130	780	420	370	TAGGTCACTTAATATAGGAGGATGTTTTCTCATGTA							64%	67%	56%
HOXB2-NM_002145.4eU1085-NP_002136.1aa296SL	3	3%	0%	3%	0%	0		3	41%	7%	310	150	900	140	290	AGCCCGGGCCATTGCCAGAAGACGTCTTCTCGGGGC	3%	3%	3%				34%	40%	48%
HP1BP3-NM_001372052.1eU1004-NP_001358981.1aa264PL	0					0		3	2%	0%	370	190	720	350	380	AACCACAAGTAAAATTGGAGGATGTCCTCCCACTGG							2%	2%	2%
HPF1-NM_017867.3eU168-NP_060337.2aa34SF	0					0		2	6%	1%	210	360	640	190	340	AGAAAAGTAAATTCTGTGAAGCTGATGTCTCCAGTG							6%	7%	
HS1BP3-NM_022460.4eU2237-3'UTR+1023	0					0		2	11%	0%	210	300	690	290	410	AGTGGCCTTTCACCAGTGGAGCTGCCTTCCCAGGGA							11%		10%
HSBP1L1-NM_001136180.2eU354-3'UTR+36	0					0		3	24%	8%	510	190	680	380	290	GCATGTCTGTATTTGGAGATGGGGCCTCTACAGAAG							30%	15%	26%
HSD17B4-NM_000414.4eU1560-NP_000405.1aa494TI	0					0		2	3%	0%	270	180	600	160	290	TACCTAATAGACCTCCTGATGCTGTACTTACAGATA							2%		3%
HSF2-NM_001135564.1eU1766-3'UTR+22	0					0		3	12%	2%	370	250	700	380	90	GATAGCTAAATCCCCAGGAAGTGGACTTTACATGTA							14%	13%	11%
HSP90AA1-NM_001017963.3eU3402-3'UTR+492	4	7%	2%	8%	1%	1	0%	2	0%	0%	570	460	490	390	240	AACTGAAGCAACTTGATGGAAGGATCTCTCCACAGG	8%	8%	7%	5%	0%		0%	0%	
HSP90AB1-NM_001271969.2eU727-NP_001258898.1aa207Q*	0					0		3	16%	1%	350	150	760	190	260	CGGGTCAAAGAAGTAGTGAAGAAGCATTCTCAGTTC							15%	16%	17%
HSPA14-NM_016299.4eU222-NP_057383.2aa42SL	0					0		3	6%	1%	320	60	580	220	220	ACCGAGTTACTCCAGCTGTTGTTGCTTACTCAGAAA							7%	7%	5%
HSPA5-NM_005347.5eU1434-NP_005338.1aa406SF	0					0		3	3%	0%	290	210	620	160	420	ATGGTGCTGCTGTCCAGGCTGGTGTGCTCTCTGGTG							3%	3%	3%
HSPA5-NM_005347.5eU230-NP_005338.1aa5LL	0					0		3	9%	1%	270	250	600	340	350	GCCCAACTGGCTGGCAAGATGAAGCTCTCCCTGGTG							8%	8%	10%
HSPA9-NM_004134.7eU1447-NP_004125.3aa455TI	0					0		3	4%	0%	290	530	710	140	470	TGGGTATTGAAACTCTAGGAGGTGTCTTTACCAAAC							4%	4%	4%
HSPD1-NM_002156.5eU1701-NP_002147.2aa547TI	0					0		3	3%	0%	200	150	750	80	400	CTCTGTTAACTACAGCAGAAGTTGTAGTCACAGAAA							2%	3%	2%
HTATSF1-NM_001163280.2eU999-NP_001156752.1aa243Q*	0					0		2	3%	1%	280	190	740	260	210	TGCAAAGACTATAAGAAGAAGCTGTCTATGCAACAA								3%	2%
HUS1-NM_001363683.2eU375-NP_001350612.1aa53AV	0					0		3	8%	2%	460	150	700	420	290	TCAACGAATTTCAAATGGAGGGTGTCTCTGCAGAAA							10%	7%	9%
HUS1-NM_004507.4eU317-NP_004498.1aa74AV	0					0		3	22%	11%	460	150	700	420	290	TCAACGAATTTCAAATGGAGGGTGTCTCTGCAGAAA							10%	32%	24%
HUWE1-NM_031407.7eU6455-NP_113584.3aa2021TI	0					0		3	18%	1%	390	90	700	260	370	GTCAGGTCTTTGCTGCAGATGGTGCCTCCACTGAGA							17%	16%	19%
HYPK-NM_001199885.1eU438-3'UTR+14	0					0		2	9%	1%	210	270	680	150	440	GAGCTAATAATGACTGAGATGGAGATATCTCGAGCA							9%		8%
HYPK-NM_001199885.1eU525-3'UTR+101	1	10%		10%		0		3	63%	5%	400	310	510	430	460	ATTGCCCTAACCAACTGATGCGTGCTTTCTCAAATA			10%				57%	64%	67%
HYPK-NM_016400.4eU316-NP_057484.4aa98R*	0					0		3	4%	1%	210	270	680	150	440	GAGCTAATAATGACTGAGATGGAGATATCTCGAGCA							5%	4%	3%
HYPK-NM_016400.4eU403-3'UTR+13	2	5%	2%	5%	2%	0		3	64%	4%	400	310	510	430	460	ATTGCCCTAACCAACTGATGCGTGCTTTCTCAAATA	4%		6%				68%	63%	61%
IARS1-NM_001374299.1eU1011-NP_001361228.1aa306TI	0					0		3	7%	1%	290	260	630	460	370	TCCTGAAGTGTAAAGAGAATGGCGCTTTCACTGTGC							5%	6%	8%
IARS1-NM_001374299.1eU1752-NP_001361228.1aa553PL	0					0		2	2%	1%	480	260	560	490	200	AAAACAAGAGGGAGTTTGAGGATGCTTTTCCTGCAG							1%		3%
IARS1-NM_001374299.1eU862-NP_001361228.1aa256LL	0					0		3	5%	1%	390	60	740	300	280	AATTAAAGATGTTGCCAGAGGACGATTACTCATTTT							4%	5%	6%
IARS2-NM_018060.4eU1353-NP_060530.3aa423TI	3	4%	1%	4%	1%	0		3	43%	7%	260	220	820	250	480	ATTGTCTAGTGGACGAAGATGGAGTTTTCACAGATG	3%	5%	4%				38%	50%	40%
IARS2-NM_018060.4eU3286-3'UTR+162	0					0		3	46%	3%	360	280	700	280	230	TCAAAATCAGAATTATAGAAGAAGTATTTCCTGTAA							50%	45%	44%
ICE2-NM_001018089.3eU594-NP_001018099.1aa37FF	0					0		2	14%	0%	580	100	490	460	230	TAATATGCTTTCTGATGATGCCCGTCTCTTCACAGA								14%	14%
IDE-NM_001322793.2eU1426-NP_001309722.1aa451TI	0					0		2	9%	3%	360	240	750	160	410	TGCATTATTATCCCCTAGAAGAGGTGCTCACAGCGG							11%		7%
IDI1-NM_001317955.2eU879-NP_001304884.1aa126PL	0					0		3	5%	1%	450	360	580	520	200	AGCTAGGAATTCCCTTGGAAGAGGTTCCTCCAGAAG							5%	4%	5%
IFT140-NM_014714.4eU1199-NP_055529.2aa291LL	0					0		2	16%	3%	390	110	640	480	220	AGACATCGCTTTGATTGAAGGCAGCCTTCTCGTGAT							18%	14%	
IFT74-NM_001099222.3eU373-NP_001092692.1aa64Q*	0					0		2	13%	3%	280	240	580	260	480	CCCATAGGGACTGGTGGAGTTCTGTCTTCTCAAATC							11%		15%
IGF1R-NM_000875.5eU4612-NP_000866.1aa1190TI	1	5%		5%		0		2	8%	2%	210	310	780	260	380	CTCCTGAGTCCCTCAAGGATGGAGTCTTCACCACTT	5%						6%		9%
IGF2BP2-NM_001007225.3eU303-NP_001007226.1aa74SL	0					0		3	40%	10%	270	140	700	290	260	TGCATGGGAAAATCATGGAAGTTGATTACTCAGTCT							40%	31%	50%
IGF2BP3-NM_006547.3eU1952-NP_006538.2aa524PL	0					0		3	37%	8%	290	280	700	250	410	AGAATTTGTCAAGTGCAGAAGTTGTTGTCCCTCGTG							29%	45%	37%

IGF2BP3-NM_006547.3eU428-NP_006538.2aa16SL	0					0	2	9%	1%	350	90	550	440	210	TCGGAAACCTCAGCGAGAACGCCGCCCCCTCGGACC					10%		8%
IGF2R-NM_000876.4eU3308-NP_000867.3aa1048SL	0					0	3	24%	6%	310	60	720	250	330	TCCGCTTTGTTTGCAATGATGATGTTTACTCAGGGC					25%	18%	29%
IKBIP-NM_201612.4eU541-NP_963906.1aa152Q*	0					0	3	35%	2%	350	180	760	260	200	ATACTGAACAACAGTCTGACGACGCTTTCTCAAGAC					37%	32%	35%
IL10RB-NM_000628.5eU329-NP_000619.3aa77SL	0					0	3	8%	2%	270	350	710	250	380	TGAATACTACCTTGACGGAATGTGATTTCTCAAGTC					6%	9%	10%
IL1A-NM_000575.5eU131-NP_000566.3aa24SS	0					0	2	10%	0%	430	320	370	650	320	TTACAGTGAAAATGAAGAAGACAGTTCCTCCATTGA					10%		10%
IL6-NM_000600.5eU188-NP_000591.1aa42PL	0					0	3	11%	3%	390	70	640	340	220	CAGGAGAAGATTCCAAAGATGTAGCCGCCCCACACA					13%	12%	8%
ILF3-NM_001137673.2eU1743-NP_001131145.1aa504TI	0					0	3	3%	1%	360	180	760	250	360	CCCCTGCCCCAGTGGTAGAAGCTGTCTCCACCCCTA					3%	4%	3%
ILF3-NM_001394808.1eU4222-3'UTR+1135	0					0	2	4%	1%	270	130	670	320	420	AAATTTTGCAATGTTTGGAGGATGTTGTCACAGCTA					4%		3%
ILF3-NM_001394811.1eU1901-NP_001381740.1aa504TI	0					0	3	5%	1%	360	180	760	250	360	CCCCTGCCCCAGTGGTAGAAGCTGTCTCCACCCCTA					5%	6%	4%
ILK-NM_001014794.3eU112-NP_001014794.1aa6TI	4	19%	8%	23%	3%	0	2	3%	0%	400	130	650	290	360	GCCGGGACGCTGCTATGGACGACATTTTCACTCAGT	21%	27%	22%	7%	3%	2%	
ILK-NM_001278441.2eU158-NP_001265370.1aa6TI	4	25%	10%	29%	7%	0	2	5%	0%	400	130	650	290	360	GCCGGGACGCTGCTATGGACGACATTTTCACTCAGT	37%	28%	23%	13%	5%		5%
IMMT-NM_001100170.2eU619-NP_001093640.1aa180SL	0					0	3	10%	1%	400	80	740	260	180	CCACACCTGCACTTTCAGAAGAAGCATCCTCATCTT					10%	11%	9%
IMMT-NM_006839.3eU652-NP_006830.2aa191SL	0					0	3	11%	3%	400	80	740	260	180	CCACACCTGCACTTTCAGAAGAAGCATCCTCATCTT					9%	10%	15%
INA-NM_032727.4eU2653-3'UTR+1079	0					0	2	7%	1%	350	210	590	220	600	AGCCTAGGTCATAAAGAGGCGTTGCTTTCACTCTCC					6%		8%
INO80E-NM_001304562.2eU273-NP_001291491.1aa68SL	0					0	2	2%	0%	480	60	590	330	0	TGCAGTACGAGAACGTGGATGAAGACTCTTCGGACT					2%		2%
INO80-NM_017553.3eU5625-3'UTR+603	0					0	2	3%	0%	250	220	700	320	270	AGGAAGTCTATCCACAGGCAGTTGTGTTCCCAGCAA					3%	3%	
INPP1-NM_001128928.2eU976-NP_001122400.1aa132TI	0					0	3	14%	5%	410	130	610	350	150	CCAGGGTTGTTCATCAGGATGTTGCCTTTACTGACC					19%	10%	14%
INPP5B-NM_001297434.2eU2110-NP_001284363.1aa695R*	0					0	2	12%	3%	280	330	700	250	250	AATGTCTTCCACTACTTGATGGCGTTTTTGCGAGAA					10%	14%	
INPP5E-NM_001318502.2eU3027-3'UTR+669	0					0	3	14%	5%	440	210	650	510	300	CCTCCCCAGCTGAGTAAGAAGCTGCTTCCCCTCCTC					19%	9%	15%
INSIG2-NM_001321329.2eU1042-3'UTR+109	0					0	3	40%	5%	210	300	690	470	270	GACTGTAAAATTGCCAGGATGCAGTTTTCCCCTTGA					38%	45%	36%
INTS1-NM_001080453.3eU4826-NP_001073922.2aa1573PL	0					0	2	7%	1%	300	70	620	480	160	TCTTCTCTGCCACTGCGGATGCTGCCTCCCCGTTTC					7%		8%
INTS7-NM_001199809.2eU1681-NP_001186738.1aa521HY	0					0	3	46%	8%	210	300	820	210	400	TTCTGGCTAAATAGTTTGAAGGAGTTTTCACATGCA					55%	43%	40%
INTS7-NM_001199811.2eU267-NP_001186740.1aa49PP	0					0	3	5%	3%	340	200	470	490	240	TGAACAGTGTGAAGCAGTTGTTCGCTTTCCCAGACT					3%	9%	3%
IP6K1-NM_001242829.2eU916-NP_001229758.1aa71PS	0					0	3	4%	2%	150	450	640	290	420	TCCCTCCCTCCCGAAATGAAGGAGTTCACCCCTGAA					2%	5%	4%
IPO5-NM_002271.6eU2534-NP_002262.4aa767SL	0					0	3	2%	1%	260	200	680	80	280	TTGGTACAGAACCAGATTCAGACGTCCTCTCAGAAA					1%	1%	2%
IPO5-NM_002271.6eU3215-NP_002262.4aa994PL	0					0	3	4%	0%	500	370	690	300	180	CTGACTGTGTAAACGTTGAAGAGGTCCTTCCACACT					5%	4%	5%
IPO5-NM_002271.6eU470-NP_002262.4aa79PL	0					0	3	11%	0%	320	130	660	280	70	TCTTGTCCTCTGCATTTGATGAAGTCTATCCAGCAC					11%	11%	11%
IPO7-NM_006391.3eU1040-NP_006382.1aa310Q*	0					0	3	10%	1%	210	300	720	150	350	GTCCAGCAAGTTTTATTGAAGGTGTTATATCAGTAC					9%	10%	11%
IPO7-NM_006391.3eU3460-3'UTR+231	0					0	2	9%	5%	460	220	740	270	310	GGATGGGAGGTACCTTAGAGGGAGTATTTTCTTTAT					5%		13%
IQSEC1-NM_001134382.3eU812-NP_001127854.1aa133Q*	0					0	3	27%	11%	260	220	730	380	290	GCGGCCCGCACCATCCAGACGGCGTTTCGCCAGTAC					16%	26%	38%
IRAK2-NM_001570.4eU837-NP_001561.3aa267PP	0					0	2	9%	3%	240	120	640	340	240	GTTGCAGATTTGTCTTAGATGCTGCCACCCCAATGT					6%		11%
IREB2-NM_001320941.2eU4853-3'UTR+1909	0					0	3	13%	4%	350	260	690	460	410	TTTGTAGTTTTAGTAGAGATGGGGTTTCACCATGTT					9%	15%	15%
ISCU-NM_001301140.1eU1839-3'UTR+214	0					0	3	52%	9%	500	140	730	370	240	GTCACCTTAGATGTTCAGAAGCCGCTTCCTCTCCAC					41%	56%	57%
ISCU-NM_014301.4eU1849-3'UTR+89	0					0	3	62%	1%	500	140	730	370	240	GTCACCTTAGATGTTCAGAAGCCGCTTCCTCTCCAC					60%	63%	63%
ITGA5-NM_002205.5eU2973-NP_002196.4aa969R*	0					0	2	2%	0%	280	320	580	230	170	GCTGTGTACAAAGCCCTGAAGATGCCCTACCGAATC					2%		2%
ITGBL1-NM_001271755.2eU205-NP_001258684.1aa16LF	0					0	3	21%	5%	150	280	670	140	360	TTCAGGAACTTCTTGCTGCTGGCGTCCTCCCTTCTC					18%	26%	19%
ITPR2-NM_002223.4eU1426-NP_002214.2aa338PL	0					0	2	20%	3%	270	320	670	290	420	AAGGAAAAAATGTGAGAGATGGAGTCCCTCCAACTT						21%	18%
ITPR2-NM_002223.4eU6715-NP_002214.2aa2101PL	0					0	3	23%	4%	380	330	650	460	270	ATGATGAGGGTGGAGATGATGGTGTTTCTCCAAAAG					19%	26%	24%
ITSN1-NM_001001132.2eU3397-NP_001001132.1aa1051PL	0					0	2	9%	3%	210	230	700	320	330	GAACAGTGGGCGACAAGGCCGGAGTCTTCCCTTCTA						11%	7%
ITSN1-XM_011529693.3eU3205-XP_011527995.1aa971SF	0					0	2	21%	3%	340	50	630	240	310	CTTCAAGGCCCATCTCAGATGCTGCCCCCTCTGTGA					24%	19%	
JADE1-NM_001287437.2eU225-NP_001274366.1aa7PP	0					0	2	19%	11%	330	140	770	420	220	CGGGGAGATCATGAAACGAGGTCGCCTTCCCAGCAG					27%		11%
JAK1-NM_001320923.2eU4066-3'UTR+384	0					0	2	3%	1%	440	440	310	610	280	CAAAATAATATCTGAAGATGATTGCTTTTCCCTGCT						2%	4%
JKAMP-NM_001098625.3eU510-NP_001092095.1aa146PL	1	9%		9%		0	3	76%	8%	310	150	810	320	170	CAGTACACTGTACTCATGAAGCCGTCTACCCACTAT	9%				71%	71%	86%
JOSD1-NM_001360235.2eU2151-3'UTR+764	0					0	3	8%	3%	420	320	690	500	410	TTTGTATTTTTAGTAGAGATGGGGTTTCTCCATGTT					4%	9%	10%
JPT2-NM_144570.3eU3161-3'UTR+2554	0					0	2	9%	6%	320	240	740	180	330	TTAGCCAGAGGATGCCAAAATTAGTCTTCTCAAAGC						5%	13%
JRKL-NM_001261833.2eU312-NP_001248762.1aa28SS	0					0	3	26%	4%	440	440	440	620	360	AATAAAGAAACTTGAAGACGGAGGTTCTTCCAAACA					29%	21%	26%
KARS1-NM_001130089.2eU1369-NP_001123561.1aa416TI	0					0	2	3%	1%	310	170	680	180	250	CAGAGGGCCAAGCCTACGATGTTGACTTCACCCCAC						2%	4%
KCNE1-NM_000219.6eU945-NP_000210.2aa129PS	0					0	3	46%	18%	290	290	720	290	300	AACACACACCTTCCTGAGACGAAGCCTTCCCCATGA					25%	57%	55%
KCTD10-NM_001317395.2eU2274-3'UTR+1291	4	4%	2%	5%	2%	0	3	24%	4%	400	360	730	140	500	GGCGCAGCGCCTATGTAGGACGGGTGTTCTCAGTGA	3%	4%	7%	3%	28%	24%	20%
KCTD1-NM_001136205.2eU1066-NP_001129677.1aa169PL	0					0	3	23%	3%	370	280	750	280	230	GTGACAAATCCTTGATAGAAGAAGTATTTCCAGAGA					22%	26%	21%
KCTD5-NM_018992.4eU774-3'UTR+43	0					0	3	4%	0%	210	290	480	390	350	TGACAGCTGAAGAAATGATTTACGTTTTCCCGAGAT					4%	4%	4%
KDM2A-NM_001256405.2eU553-NP_001243334.1aa120PL	0					0	2	3%	2%	260	120	580	360	240	TAACACCTGTGAGGCCAGCTGCTGCCTCCCCGATTG						2%	5%
KDM3B-NM_016604.4eU3344-NP_057688.3aa1078SF	0					0	2	3%	1%	310	310	770	250	270	CAGAAGAGATGGGTGATGAAGAAGTTTTCTCCTGGT					4%		2%
KDM5B-NM_001314042.1eU5838-NP_001300971.1aa1558PL	0					0	2	6%	1%	270	160	690	270	390	GGTTTCATCAGGTCTGTGTTGGTGTCTCCCCAGAGA					5%		7%
KHSRP-NM_001366299.1eU3911-3'UTR+1557	0					0	3	11%	1%	300	130	600	220	250	CCCCCTCCCAGGACTGGTCTGTTGTCTTTTCATCTG					11%	11%	10%
KHSRP-NM_001366299.1eU486-NP_001353228.1aa126Q*	0					0	3	5%	1%	210	300	810	320	280	GATCAACCGGAGAGCAAGAAGCTGGCTTCCCAGGGA					6%	6%	4%
KIAA0753-NM_001351225.2eU3356-NP_001338154.1aa660TI	0					0	3	24%	7%	290	250	770	180	400	TGTGCGAAGATTATGCAGAAGCTGTGTTCACCTCAG					19%	32%	20%
KIAA1191-NM_001079684.3eU2597-3'UTR+1436	0					0	2	4%	0%	540	280	490	660	200	TACCTTGTCTGTATAAGAAGCTGGCCTTTCCATAGA					4%	5%	
KIAA1522-NM_001198972.2eU1035-NP_001185901.1aa298HY	0					0	2	16%	4%	260	140	660	280	270	CAGGCCACCTACCTGTCGAAGTTGATTCCACATGCT					18%	13%	
KIF15-NM_020242.3eU4288-3'UTR+49	0					0	3	23%	3%	570	200	640	320	190	TAGGCATCACCTTGTTTGAAGATGTTTCTTCTCTTT					27%	23%	21%
KIF18A-NM_031217.4eU2400-NP_112494.3aa754PL	0					0	3	9%	4%	380	280	600	300	280	GTCTGAAAATGTTGTGTGAAGTAGCTATCCCTCATA					8%	14%	5%
KIF18A-NM_031217.4eU301-NP_112494.3aa54FF	0					0	2	7%	1%	420	380	490	540	290	TGATCCCAAACAAGAAGAAGTCAGTTTTTCCATGG					7%	8%	
KIF18B-NM_001264573.2eU3463-3'UTR+801	0					0	3	6%	0%	450	390	750	520	490	TTCGTCTTTTTAATAGAGAGGGGGTTTTTCCATGTT					6%	6%	7%
KIF1C-NM_006612.6eU3743-3'UTR+76	0					0	3	2%	1%	350	130	660	470	380	GAAGGGAAGACGCCCGAGACGCTGCTTCCCCAGAAG					2%	2%	3%
KIF20A-NM_005733.3eU1779-NP_005724.1aa556SF	0					0	2	2%	1%	210	300	690	160	240	ATGATGATATTGAAAATGAAGCTGACATCTCCATGT					2%		3%
KIF22-NM_001256269.2eU2003-NP_001243198.1aa581LL	0					0	3	7%	1%	310	340	680	220	380	GGCATAACGGGGAAACAGATGGAGTCCTTCCTGAAG					6%	8%	7%
KIF22-NM_001256269.2eU2069-3'UTR+13	0					0	3	8%	2%	380	40	650	330	210	CGCTGTGGCGCCTCCTGACCGTCGTCTCCTCACTCC					10%	6%	8%
KIF2A-NM_001098511.3eU2195-NP_001091981.1aa643PL	0					0	3	8%	5%	520	210	740	390	150	TTTGTGAACAAAATGAAGAAGAAGTCTCTCCACAGT					7%	14%	4%
KIFBP-NM_015634.4eU1851-NP_056449.1aa607LL	1	2%		2%		0	3	21%	7%	460	250	570	150	540	AGAACTTAGTAAAGAGATGGTTAGTCTTCTCCCAAC			2%		21%	28%	13%
KIRREL1-NM_001286349.2eU6693-3'UTR+4687	0					0	2	4%	1%	290	120	650	290	380	GCTTTTGGAGCTCTTCAGATGCTGTCCCCCCTGAGA					5%		4%
KIRREL1-NM_018240.7eU310-NP_060710.3aa93SF	0					0	3	7%	3%	380	120	640	120	390	ACAACCTGGAGATCACAGATGCTGAGCTCTCTGACG					9%	9%	4%
KLC1-NM_001394833.1eU2422-3'UTR+148	0					0	2	11%	3%	390	430	700	190	270	TGACGTCCACCAACCTTGGAGGTGCCTTTTCAAAAC						14%	9%
KLC2-NM_001134774.2eU202-NP_001128246.1aa8RW	0					0	2	7%	2%	260	150	670	320	370	GCCACAGCCATGGCCATGATGGTGTTTCCGCGGGAG					9%		6%
KLC2-NM_001134775.2eU704-NP_001128247.1aa152PL	0					0	3	3%	1%	430	200	700	400	100	AGATCCGCAAGTTGGATGAAGACGCCTCCCCTAACG					3%	4%	4%
KLF10-NM_001032282.4eU1958-3'UTR+277	0					0	3	2%	1%	280	40	700	160	300	ATTTTTTGAAGGTTTCAGATGAGGTCAACACAGGTA					2%	3%	2%
KLF5-NM_001286818.2eU1017-NP_001273747.1aa274PP	0					0	3	7%	1%	200	290	680	250	370	ACCTGTCAGATACAATAGAAGGAGTAACCCCGATTT					6%	7%	8%
KLF5-NM_001286818.2eU2207-3'UTR+911	0					0	2	4%	0%	220	210	700	290	410	TAAAAAGAAGGACTTAGGGTGTCGTTTTCACATATG						4%	4%
KLF6-NM_001160124.2eU2083-3'UTR+1162	0					0	3	13%	1%	350	250	770	210	410	AAAACCACTAACGCTTAGAAACTGTTTTCTCATGCA					14%	11%	13%
KLHL15-NM_030624.3eU2788-3'UTR+717	0					0	3	16%	7%	570	250	590	100	400	ACTTGTTGAATTTTAGATGTAACGTCTTTTCACATA					18%	22%	9%
KLHL29-NM_052920.2eU3702-3'UTR+319	0					0	3	18%	9%	340	370	740	290	430	TTTTCAACTGGGAGAGAGAAGCTGTTTTTTCCTTCC					20%	7%	26%
KLHL42-NM_020782.2eU4828-3'UTR+3212	0					0	3	14%	5%	510	130	710	260	170	GCCTATTGAGCATTGTGGATGATGTGTTTTCAGATT					9%	18%	14%
KLHL42-NM_020782.2eU6125-3'UTR+4509	0					0	3	23%	9%	320	270	680	300	340	AATGACTTGTACGGTTGGCTGGTGCTTTCTCAAATG					14%	24%	32%
KMT2C-NM_170606.3eU357-NP_733751.2aa47PL	0					0	2	33%	24%	340	220	690	470	360	GGGGCCGGCCTCGCAAAGATGGCGCTTCCCCTTTCC					17%	50%	

KMT5A-NM_001324504.2eU1181-3'UTR+145	0					0			3	12%	3%	520	210	660	270	100	GTTTTTAAAAAGTATATTAAGATGCCTTTTCACTGT								15%	12%	9%
KNL1-NM_144508.5eU178-NP_653091.3aa6SL	0					0			2	15%	3%	610	190	690	410	190	AGTTTTCTTCAAAAATGGATGGGGTGTCTTCAGAGG								17%		13%
KNL1-NM_144508.5eU2755-NP_653091.3aa865SL	0					0			2	10%	6%	430	210	460	300	250	GACCAAAAATTGATAAGACTATTGTATTTTCAGAAG								14%	6%	
KPNA2-NM_001320611.2eU665-NP_001307540.1aa15HH	0					0			3	12%	2%	300	70	580	310	340	GAATGCTAATACACCAGCTGCCCGTCTTCACAGATT								11%	14%	10%
KPNA2-NM_002266.4eU174-NP_002257.1aa15HH	1	1%		1%		0			3	15%	2%	300	70	580	310	340	GAATGCTAATACACCAGCTGCCCGTCTTCACAGATT		1%						13%	17%	13%
KPNA5-NM_001366304.1eU441-NP_001353233.1aa106TI	0					0			2	15%	3%	320	200	620	300	320	TACCCATTCCAGAGGAAGAAGTTGTTACTACAGATA								13%		17%
KPNB1-NM_001276453.2eU3700-3'UTR+856	0					0			3	3%	1%	410	420	650	370	120	TATCCTGATCTTTTCATCAAGGCGCCTTTCCTAATA								4%	4%	2%
KRIT1-NM_001350671.1eU1701-NP_001337600.1aa70LL	0					0			2	11%	0%	270	150	830	220	210	AGGAGATTCAGAATTACTAAGCCGTCTTCTCAGTGA								11%		12%
KRT80-NM_001081492.2eU1046-NP_001074961.1aa312Q*	0					0			3	6%	1%	320	220	690	270	280	CTCAATGTGCGCATCCAGAAGCTGCGGTCCCAGATC								6%	8%	6%
LAMA1-NM_005559.4eU9441-3'UTR+136	0					0			3	15%	7%	360	130	660	180	420	AAGTGTTTCTGGGGAGAGATGTTGTGTTTACGTTAC								7%	21%	18%
LAMC1-NM_002293.4eU1692-NP_002284.3aa465CC	0					0			3	3%	1%	450	260	710	300	170	ATGTAATATTGAAACAGGAAGATGTGTTTGCAAAGA								3%	3%	4%
LAMP1-NM_005561.4eU2364-3'UTR+914	0					0			3	2%	1%	350	300	550	350	320	GCTGTGTACAATGTGAGATCGGTGCGTTCTCCTGAT								1%	3%	2%
LAP3-NM_015907.3eU352-NP_056991.2aa28SS	0					0			3	5%	1%	390	220	660	310	310	CGTGAGACGTTTCGGGAGCCGGAGTCTCTCCACCGC								5%	4%	6%
LAPTM4B-NM_018407.6eU665-NP_060877.4aa148SL	0					0			3	27%	1%	250	70	730	80	370	CTAATTTTCCCTACAGAGATGATGTCATGTCAGTGA								25%	28%	27%
LARP1-NM_001367713.1eU3610-3'UTR+696	0					0			3	10%	1%	320	340	600	340	410	ACTCCCCAGCCCAGAGAGACGCTGCTTTTACCAGGA								10%	9%	10%
LARS1-NM_001317964.2eU2314-NP_001304893.1aa719LL	0					0			2	2%	1%	440	250	480	430	330	TGTGGAAGCCATGGCAGATGCAGGTATTCTCCGTCT									3%	2%
LARS1-NM_001317964.2eU2386-NP_001304893.1aa743AA	0					0			2	3%	1%	240	310	730	300	260	CAACTGGGACAGCCTAAGAAGTGGTCCTGCCAGCAC									2%	3%
LATS1-NM_001350339.2eU6423-3'UTR+2443	0					0			3	7%	2%	350	260	690	460	410	TTTGTATCTTTAGTAGAGATGGGGTTTCGCCATGTT								7%	5%	9%
LATS2-NM_014572.3eU2736-NP_055387.2aa764PL	0					0			3	4%	1%	470	220	580	530	370	TGAGCCTGCTGATCCGGATGGAGGTCTTCCCTGAGC								3%	3%	4%
LCMT1-NM_016309.3eU513-NP_057393.2aa124PL	0					0			2	6%	2%	360	250	660	370	140	TCCCAAGTAAATATTTTGAGGTTGACTTTCCAATGA								4%		7%
LDHA-NM_001135239.2eU386-NP_001128711.1aa96PP	0					0			3	4%	1%	350	370	740	390	470	CTACGTGGCTTGGAAGATAAGTGGTTTTCCCAAAAA								3%	4%	5%
LDHA-NM_001165414.2eU746-NP_001158886.1aa183PP	0					0			3	5%	1%	350	370	740	390	470	CTACGTGGCTTGGAAGATAAGTGGTTTTCCCAAAAA								3%	6%	5%
LETM1-NM_012318.3eU3636-3'UTR+1210	0					0			3	7%	1%	430	260	670	150	280	GAAAGCAGCTGCACTCAGACAATGCCTTCTCCATTA								5%	6%	8%
LGMN-NM_001008530.3eU1538-NP_001008530.1aa418LF	0					0			3	13%	3%	300	80	750	250	330	CTGGTCAACCTTTGTGAGAAGCCGTATCCGCTTCAC								9%	15%	13%
LGMN-NM_001008530.3eU615-NP_001008530.1aa110PL	0					0			2	3%	1%	470	130	650	410	490	CGAAGGACTACACTGGAGAGGATGTTACCCCACAAA									4%	3%
LHPP-NM_001167880.2eU363-NP_001161352.1aa108SL	0					0			2	22%	15%	260	140	740	120	320	CATACCTGCTCATCCATGACGGAGTCCGCTCAGAAT								11%		33%
LIG1-NM_000234.3eU264-NP_000225.1aa35PS	0					0			3	5%	2%	350	290	670	200	450	TCCAATAGCAGCAGAGAGACGGAGCCCCCTCCAAAG								3%	4%	7%
LIG4-NM_001098268.2eU2552-NP_001091738.1aa779SL	0					0			3	12%	7%	390	110	700	390	290	CAGACTTGAACCAACTGAAGGAAGTATTCTCAGGAA								15%	5%	17%
LIMA1-NM_001113546.2eU1155-NP_001107018.1aa344PL	0					0			3	12%	6%	300	150	710	310	160	TCCGTTCCACCCCTGCCGAAGATGACTCCCCAGGTG								18%	9%	8%
LIMA1-NM_001113546.2eU1900-NP_001107018.1aa592FF	0					0			3	2%	0%	420	190	690	250	190	TTCTTCACTGAAGGAAAGAAGCCGCCCATTCACTGT								2%	1%	2%
LIN54-NM_001115007.3eU1512-NP_001108479.1aa444Q*	4	33%	15%	39%	11%	0			2	8%	5%	220	250	720	120	340	CAAACAGCAGCCAAGACGAAGTTATCCTCTCAAATT	52%	35%	31%	14%				4%		12%
LINC00641-NR_038970.1eU3715-ncr	1	6%				0			3	20%	2%	260	220	690	380	470	TTGTTATTTTTAGTAGAGACGGTGTTTCACCATGTT				6%				20%	18%	21%
LINC02987-NR_110687.1eU1517-ncr	4	51%	17%	59%	12%	0			3	89%	10%	260	220	760	380	570	TTTGTCTTTTTAGTAGAGACGGTGTTTCCCCATGTT	72%	52%	52%	30%				100%	87%	81%
LMBRD1-NM_001363722.2eU244-5'UTR-81	0					0			3	61%	6%	260	230	720	210	400	AAAGTCGGCGGGAAAGTGAAGTTGTCTCCACCATAA								61%	56%	67%
LMBRD1-NM_018368.4eU269-NP_060838.3aa47TI	0					0			3	63%	1%	260	230	720	210	400	AAAGTCGGCGGGAAAGTGAAGTTGTCTCCACCATAA								62%	62%	64%
LMF2-NM_001363816.2eU2230-3'UTR+10	0					0			2	3%	1%	460	250	730	180	350	ACCACCCGGCGAAAGAAGTAGCTGTGTTCTCCCAGC								3%	4%	
LMO7-NM_001306080.2eU4404-NP_001293009.1aa1359SL	0					0			2	4%	0%	470	140	670	300	120	ATGATATACCAAAGACAGAAGAAGCATCTTCAGGTT								4%		4%
LMTK2-NM_014916.4eU133-5'UTR-185	0					0			3	8%	2%	330	260	600	440	310	CGCAGGCGGGCGGGAAGGATGGTGTTTCTGCGACTG								7%	7%	10%
LOC107987147-XR_001747447.2eU4286-ncr	0					0			2	10%	2%	200	200	700	250	420	AGAATTAAGTCCACAGTGAAGTTGTCCCCCCATCCC								8%	11%	
LOC730101-NR_024403.1eU561-ncr	0					0			2	16%	8%	440	200	650	420	300	TGGGGCCTGCGCCCTGAGAGGTTGCCTTTCCTTTTC								11%	22%	
LOXL2-NM_002318.3eU516-NP_002309.1aa89SF	0					0			3	5%	2%	310	220	760	140	220	AGTGGGGCACCGTGTGCGATGACGACTTCTCCATCC								6%	6%	2%
LPCAT3-NM_005768.6eU823-NP_005759.4aa257LL	4	24%	11%	30%	2%	2	4%	1%	3	5%	1%	540	80	520	500	310	CAGCCCCCACATCACAGAAGACTATCTCCTCACTGA	27%	31%	31%	8%		5%	4%	4%	5%	6%
LRATD2-NM_174911.5eU4158-3'UTR+2684	0					0			2	35%	5%	250	200	720	400	340	GGAAATAGATCTTTTCAGATGCTGCTTTCCCATGTA								32%	38%	
LRIF1-NM_001006945.2eU1130-3'UTR+114	2	2%	1%	2%	1%	0			3	39%	13%	420	230	540	460	390	AAGTGTCTTTGAATATGAAGCTTGTTTTTTCATCAG	1%	2%						33%	32%	54%
LRIF1-NM_001006945.2eU219-5'UTR-97	0					0			2	3%	0%	510	110	630	470	170	CGCTTCCCACTTTTCTGGATGAGGTGTCCCCTTTCT								3%		3%
LRIF1-NM_001006945.2eU895-NP_001006946.1aa194PL	4	46%	16%	54%	5%	3	2%	0%	3	5%	2%	210	280	700	310	370	TCAATAAAAATTATACCGAAGATATTTTCCCAGTGA	59%	50%	51%	23%	2%	2%	3%	5%	3%	7%
LRP6-NM_002336.3eU4510-NP_002327.2aa1401RC	0					0			3	9%	2%	390	0	630	330	280	TACTTTATCTGCCAGAGGATGTTGTGTCCACGTATG								7%	11%	8%
LRRC20-NM_001278211.2eU3003-3'UTR+2253	0					0			3	63%	16%	270	310	770	290	330	TGAAGGGGCAGCTTCTGGAAGTTGTTTTCTCCTCTG								62%	48%	79%
LRRC41-NM_006369.5eU2474-NP_006360.3aa727SL	0					0			2	8%	1%	320	210	740	250	330	CTGGCCTGCTGGCCTTGGCAGATGTTTTCTCAGAGG								7%		8%
LRRC47-NM_020710.3eU2068-3'UTR+294	0					0			3	18%	4%	350	260	690	460	410	TTTGTATTTTTAGTAGAGACGGGGTTTCACCATTTT								16%	15%	23%
LRRC58-NM_001099678.2eU682-NP_001093148.1aa191LL	0					1	10%		2	10%	0%	400	190	590	350	360	ATCCCACCAGAATTAGGAAATCTGCCTTCTCTGAAT							10%	9%		10%
LRRFIP1-NM_001137552.2eU2735-3'UTR+57	0					0			3	8%	1%	570	340	790	260	270	ACAGGAAGTCTCAGTGTGAAGGGGTCTTTTCTCTCC								10%	7%	7%
LRWD1-NM_001317721.2eU1584-NP_001304650.1aa338SF	0					0			3	5%	1%	360	150	770	180	210	GGAGGGTGTGTGAAGTGGAATTCGTCTTCTCTGAGG								6%	5%	4%
LSM14B-NM_144703.3eU1898-3'UTR+548	3	7%	3%	9%	0%	0			3	51%	6%	300	410	820	470	390	GGTGGGTGGAGGGCACGGAAGGGGTTTTCCCATGGA		9%	9%	4%				57%	46%	48%
LSM5-NM_001130710.2eU424-3'UTR+140	0					0			3	57%	2%	330	150	720	240	230	TTAAAGGGAAGTGCTTTGAAGATGTGTACCCATTTT								57%	55%	59%
LSM8-NM_016200.5eU1250-3'UTR+899	0					0			3	55%	22%	250	80	820	80	250	CCATAAACCTGATTTCAGAAGACGTCAACTCAGTTT								55%	78%	33%
LTA4H-NM_000895.3eU1773-NP_000886.1aa569RW	0					0			2	3%	1%	250	170	550	510	310	GCAACTGAACAAGGAAGAATGAAGTTTACCCGGCCC									4%	2%
LTBP4-NM_001042544.1eU3402-NP_001036009.1aa1134PP	0					0			3	29%	3%	440	240	690	360	270	GTTTGACCCCATGACTGGACGCTGTGTTCCCCCACG								28%	32%	27%
LTV1-NM_001329953.2eU195-5'UTR-623	0					0			3	3%	1%	400	340	590	220	460	TTTATAGAGAAGAAGAAAGCTGTGTCTTTTCACTTG								2%	5%	3%
LY6E-NM_002346.3eU111-5'UTR-6	4	4%	2%	5%	1%	0			2	1%	0%	210	310	510	330	480	TGTGACCTCCAGGCAGGACGGCCATCCTCTCCAGAA	6%	3%	4%	2%				1%		2%
LYRM2-NM_020466.5eU242-NP_065199.1aa71Q*	0					0			3	6%	3%	310	110	660	290	220	GAAGAGGATACAATCCGGATGATGATTACTCAAGGC								8%	3%	7%
MAGEA3-NM_005362.4eU909-NP_005353.1aa248Q*	0					0			2	2%	0%	190	310	630	380	370	ATCTTGGGGGATCCCAAGAAGCTGCTCACCCAACAT								2%	3%	
MAML1-NM_014757.5eU1201-NP_055572.1aa304PS	4	50%	13%	57%	4%	0			3	14%	2%	170	420	660	190	500	CAGGACATTAATATTAAGACGGAATTCTCTCCAGCA	60%	54%	55%	31%				14%	12%	17%
MAMLD1-NM_001177465.3eU3864-3'UTR+590	0					0			3	36%	1%	270	210	780	250	250	TAATGAGCTTTACAGCAGAAGCCGTTTTATCGTTAA								36%	35%	38%
MANF-NM_006010.6eU207-NP_006001.5aa52SL	4	29%	8%	33%	3%	0			3	3%	0%	370	70	720	190	340	AGGACCTCAAAGACAGAGATGTCACATTCTCACCAG	37%	31%	32%	17%				2%	3%	3%
MAP1B-NM_001324255.2eU4550-NP_001311184.1aa1446PL	0					0			2	5%	1%	450	130	650	350	250	TTCCAGAGCCAACAACAGATGATGTGTCTCCATCTC								4%	6%	
MAP1B-NM_001324255.2eU5651-NP_001311184.1aa1813SF	0					0			2	5%	1%	390	250	670	220	220	CTACCAAGACCCCTGAAGATGGTGACTATTCCTATG									6%	4%
MAP1S-NM_001308363.2eU3315-NP_001295292.1aa1027PL	0					0			3	13%	4%	290	140	720	410	140	GCATGGTGTCCATGCAGGATGACGCCTTCCCGGCCT								15%	10%	16%
MAP2K4-NM_001281435.2eU1601-3'UTR+358	0					0			3	10%	3%	320	260	670	370	430	GGAGATGCAGCTACTGGAATGGTGTTTTGTCAGACT								14%	9%	8%
MAP2K4-NM_001281435.2eU645-NP_001268364.1aa212PL	0					0			3	33%	11%	310	260	640	290	310	ATGTATATAGTGTATTAGATGATGTTATTCCAGAAG								40%	20%	38%
MAP3K13-NR_038322.2eU1239-ncr	4	27%	12%	30%	13%	0			3	5%	1%	270	190	650	300	300	GTCCTGTTACACAGTTGGAAGTCATTCCCTCAGATT	43%	30%	18%	16%				5%	5%	6%
MAP3K1-NM_005921.2eU2414-NP_005912.1aa797LF	0					0			2	22%	2%	330	280	640	380	340	GTTGAAATCAGGTATAAGAAGCTGCTGTCCCTCTTA								21%	23%	
MAP3K2-NM_001371910.2eU5374-3'UTR+2925	1	4%		4%		2	7%	0%	3	6%	1%	210	100	440	330	340	AATGATATGGATCAACAAATGCAATTTGAACTTTTT		4%			7%		7%	6%	5%	6%
MAP4K4-NM_001242559.2eU1069-NP_001229488.1aa232PL	0					0			3	11%	2%	380	350	620	370	450	CAGCCATTGAGATGGCAGAAGGTGCTCCCCCTCTCT								12%	13%	9%
MAPK1-NM_002745.5eU2600-3'UTR+1312	0					0			2	3%	1%	360	170	540	340	330	TCGGTGTTCTGTCCCAGTGCGGTGCCTCCTCTTGAC								2%	4%	
MAPK1-NM_002745.5eU4389-3'UTR+3101	0					0			3	7%	1%	390	250	680	330	330	ATTTCAGCCATTCAGAGGAAACTGTTTTCTCTTTAT								8%	6%	6%
MAPK7-NM_002749.4eU1553-NP_002740.2aa472SL	2	4%	0%	4%	0%	0			3	22%	6%	300	160	780	170	400	CTGCCCCACCAAAGAAAGATGGTGCCATCTCAGACA	4%		3%					15%	28%	22%
MARCHF5-NM_017824.5eU1543-3'UTR+378	0					0			2	2%	0%	360	260	570	210	450	TTTTGTACATGTTCATGTTTAGTGTTTTCTCAGAAT									3%	2%
MARS1-NM_004990.4eU1924-NP_004981.2aa634SF	0					1	2%		3	4%	1%	390	360	620	340	340	TTCGGCCTGAGGGCCAGGACAGTGCTTTCTCCTGGA						2%		3%	5%	4%
MAT2A-NM_005911.6eU968-NP_005902.1aa283SL	0					0			3	5%	2%	340	380	740	190	430	GTTGGGGTGCTCATGGAGGAGGTGCCTTTTCAGGAA								3%	6%	5%
MATR3-NM_001194954.2eU1314-NP_001181883.1aa323LF	0					0			3	4%	1%	510	160	290	580	180	GCAAGTCACAGTCGTCGATGCCAGCTTCTTCTTGAA								4%	4%	3%
MATR3-NM_001194954.2eU2769-NP_001181883.1aa808LF	0					0			3	2%	0%	430	150	650	260	320	AAAACAGGGTTTTACTGTAAGCTGTGTTCACTCTTT								2%	2%	2%
MATR3-NM_001194956.2eU344-NP_001181885.1aa35LF	0					0			3	7%	2%	510	160	290	580	180	GCAAGTCACAGTCGTCGATGCCAGCTTCTTCTTGAA								7%	5%	8%

MBIP-NM_001144891.2eU918-NP_001138363.1aa290PL	4	47%	6%	48%	6%	1	9%		2	25%	2%	380	300	650	430	150	A T A A A A T C C T T G A A T T G G A A G G C A T C T C T C C T G A A T	56%	45%	45%	43%			9%		26%	23%
MBIP-NM_016586.3eU918-NP_057670.2aa290PL	4	52%	11%	56%	10%	2	7%	1%	3	21%	5%	380	300	650	430	150	A T A A A A T C C T T G A A T T G G A A G G C A T C T C T C C T G A A T	67%	52%	49%	40%	6%		7%	15%	22%	25%
MBNL1-NM_001314057.2eU3074-3'UTR+1703	0					0			3	7%	0%	210	200	690	220	490	A A T C G T G T T A A G A A A G T A A T G G T G A C T T C A C A T G T T								7%	7%	6%
MBNL2-NM_001306070.2eU110-5'UTR-695	0					0			2	4%	1%	270	210	680	400	300	T G A G A A C T A T T T A C A T G G A A G T T T C T T T C T C A T G A T								3%		5%
MBNL2-NM_001306070.2eU723-5'UTR-82	0					0			3	4%	2%	250	270	700	300	440	C A C C T T C A G A C T T A C A T G T G G G A G T T T T C A C A A C A G								3%	6%	3%
MBTPS1-NM_003791.4eU2181-NP_003782.1aa550SF	0					0			2	8%	2%	360	250	760	190	240	A G A A C G G A G A C A A C A T T G A A G T T G C C T T C T C C T A C T								10%	6%	
MCCC2-NM_001363147.1eU2012-3'UTR+348	0					0			3	4%	1%	440	250	670	300	210	A T T G A G A T G A T T A A T A T A A A G T T G T A T T T T C A C T G A								3%	4%	4%
MCCC2-NM_001363147.1eU643-NP_001350076.1aa186PL	0					0			3	8%	3%	410	280	670	280	250	C A T A C T T A C C T C G A C A A G C A G A T G T G T T T C C A G A T C								8%	5%	11%
MCCC2-NM_022132.5eU643-NP_071415.1aa186PL	0					0			3	13%	4%	410	280	670	280	250	C A T A C T T A C C T C G A C A A G C A G A T G T G T T T C C A G A T C								8%	16%	15%
MCM10-NM_018518.5eU3435-3'UTR+686	0					0			2	8%	5%	360	170	840	180	430	A T A T A T A T A G A G A G A A A G A A G G T G T C T G C T C T T A C A									5%	12%
MCM5-NM_006739.4eU482-NP_006730.2aa135SS	0					0			3	8%	1%	300	290	340	480	310	G G T C A T G C T C A A G T C G G A C G C C A G C C C T T C C A G C A T								8%	10%	7%
MCM6-NM_005915.6eU1809-NP_005906.2aa577LL	4	24%	9%	28%	3%	0			3	3%	1%	310	150	780	140	350	T T C C C T C G A T G A T A T C A G A A G A T A T C T T C T C T T T G C	30%	25%	30%	10%				3%	4%	3%
MCM8-NM_001281520.2eU1214-NP_001268449.1aa261PP	0					0			2	4%	0%	440	220	660	360	330	T C C T C T T C C A G A T G G A A A A T A C A G T C T T C C C A C A A A									4%	4%
MCM8-NM_001281520.2eU1590-NP_001268449.1aa387LF	0					0			3	7%	4%	240	270	660	150	470	T G T A A G C A T G G A A T G T T G A T G G A G T T C T C A C T T A A A								5%	11%	4%
MCRS1-NM_001012300.1eU146-NP_001012300.1aa49SF	0					0			2	8%	4%	590	230	530	510	300	A G T C A C T G G C A G G G C A G A A G C G A G C C T C C T C C C A G G									5%	12%
MCRS1-NM_006337.5eU312-NP_006328.2aa36SF	0					0			3	5%	1%	590	230	530	510	300	A G T C A C T G G C A G G G C A G A A G C G A G C C T C C T C C C A G G								6%	5%	4%
MDM1-NM_001205028.3eU1906-NP_001191957.1aa597PS	0					1	13%		2	13%	3%	270	340	600	310	320	C C A A C A A A T C C C C C T G G A C A G T T G C C T T C T C C A C C A					13%				15%	11%
MDN1-NM_014611.3eU17490-3'UTR+512	0					0			3	6%	1%	590	220	700	310	290	T C G T T T C T G G A G A G T A T G A G G G T G T G T T T T C T T A T T								7%	6%	5%
ME2-NM_001168335.2eU1400-NP_001161807.1aa399FF	0					0			2	3%	0%	480	130	640	350	150	A A T T G G A G T T G C A G G T G C T G G C C G T C T T T T C A C T C C								3%	2%	
MEAF6-NM_001270875.3eU895-3'UTR+299	0					0			2	6%	1%	470	300	470	400	510	G T T T C T C C C T T C T T A G A A T G A A T G T C T C C C C C A A A A								7%	5%	
MED12-NM_005120.3eU5770-NP_005111.2aa1871R*	0					0			3	9%	4%	190	250	630	270	310	G T G C G C T T A C C A A T G C A G A A G C T G C C C A C C C G A C C A								4%	11%	12%
MED12-NM_005120.3eU65-5'UTR-96	0					0			2	28%	12%	170	170	720	160	280	C C C C T T G T T G T C T C T C G G C C G C C G T C C T C T C A A C C A									37%	20%
MED15-NM_001003891.3eU1813-NP_001003891.1aa582LL	0					0			3	4%	1%	520	190	670	430	170	A T T C T G A C A G A C C C C T C G A A G C G G T G T C C C C T G A A G								3%	4%	4%
MED1-NM_004774.4eU1536-NP_004765.2aa445LL	0					0			3	10%	4%	480	250	580	290	500	T G G G C T T C T C C A A T T T G A A G T G T G T C C T C T C T C A G A								8%	15%	7%
MED23-NM_001270521.2eU261-NP_001257450.1aa23PL	0					0			3	7%	2%	490	380	640	480	220	T G A A A A C G G A A G T T A T A G A A G A G G C T T T C C T G G C A								6%	9%	7%
MEGF8-NM_001271938.2eU890-NP_001258867.1aa94PL	0					0			3	16%	9%	360	160	660	350	240	A C T A C C T G T T C G T G T A T G A C G G T G A C T C C C C G C G A G								6%	24%	18%
MEGF9-NM_001080497.3eU3390-3'UTR+1469	0					0			3	63%	23%	390	380	700	250	250	G T T A G T T C T T G C T T T T T G A T G G A G T T T T T T C C T A G A								43%	88%	60%
MEPCE-NM_001194990.2eU1765-3'UTR+40	0					0			2	2%	0%	310	120	650	300	390	T A A A C A G A A A G T G T G A A G A G G C T G C C C T C G C T G C T C								2%		2%
MERTK-NM_006343.3eU3458-3'UTR+329	0					0			3	74%	18%	340	320	750	250	230	A A C A T T T G T A C A G A G T T G A A G T T G T T T T T C A A G T T								59%	70%	94%
MESD-NM_015154.3eU273-NP_055969.1aa82PL	0					0			3	4%	2%	270	480	660	330	330	A A G A T G A T G A C A T T G A A G A A G G A G A T C T T C C A G A G C								6%	3%	3%
METAP2-NM_001317182.2eU114-NP_001304111.1aa29SF	0					0			2	2%	0%	340	280	660	310	320	A T C C A G A C G A C A G G G A A G A A G G A G C T G C C T C T A C G G								2%		2%
METTL15-NM_001113528.2eU3226-3'UTR+1684	0					0			3	28%	8%	260	400	670	240	460	C T G A A T G T A C T G G G T T G A A T G G T G T C C T C T C C A A A A								21%	26%	36%
METTL16-NM_024086.4eU1240-NP_076991.3aa370FF	0					0			3	3%	1%	510	270	480	480	240	T C C C T G T G G A A A A G A G G A A G T C A G C C T T T C C T A A C								4%	2%	3%
METTL18-NM_001320199.2eU250-NP_001307128.1aa4Q*	0					0			2	16%	5%	400	180	670	250	290	T T G T G T G C A T T T A A T A A G A G G A T G A C C T T T C A G T T T								13%	20%	
METTL6-NM_001301790.2eU235-5'UTR-34	0					0			2	4%	1%	390	150	630	250	160	G A T T C A T A T T A T T T G G T G A T G G A G A A T C T T C A G C T G								4%		5%
MFSD10-NM_001120.5eU1204-NP_001111.3aa314LF	0					0			3	9%	5%	510	340	660	430	310	A G T A G C C T A C A G C A G G G G A A G A T G T T T T T C C T C A T C								4%	7%	15%
MFSD14B-NM_032558.3eU685-NP_115947.2aa145SL	0					0			3	2%	1%	230	160	660	180	290	T T G C G A T G A T T T C T G T G T C T G G A G T C T T C T C G G T C A								3%	2%	2%
MFSD5-NM_001170790.2eU1442-NP_001164261.1aa432SF	0					0			2	10%	1%	370	110	660	180	250	T G T C C C T T G C T G T G C T C A T C G T C G T C T T C T C T C T C T								10%		9%
MGA-NM_001080541.3eU1432-NP_001074010.2aa415SL	0					0			2	17%	3%	400	90	560	390	310	A A G A G A C A G A T G A A G A G A C G G A T G T A T A C T C A A A C A								15%		19%
MGAT5-NM_001371457.1eU533-NP_001358386.1aa12Q*	2	18%	9%	18%	9%	0			3	36%	8%	260	200	800	160	340	G C T C T C T T C A C T C C G T G G A A G T T G T C C T C T C A G A A G		11%	24%					43%	27%	38%
MGC70870-NR_003682.1eU399-ncr	0					0			3	74%	19%	300	220	770	320	390	A A T G G T T T T G A A C C A A A G A A G C T G T A T T C C C T A A A								81%	88%	52%
MGME1-NM_001310338.2eU202-NP_001297267.1aa7Q*	0					0			3	4%	1%	370	190	680	230	250	A A A C A A A T C T G A A T G A A G A T G A A G T T A T T T C A G A C C								3%	5%	4%
MGST3-NM_004528.4eU81-NP_004519.1aa5SF	4	4%	2%	5%	1%	0			3	33%	3%	340	210	550	370	460	G G T G A T C C A G A C G C A A G A T G G C T G T C C T C T C T A A G G	6%	4%	5%	2%				37%	32%	31%
MIA2-NM_001247988.1eU1863-NP_001234917.1aa537PS	0					0			3	25%	8%	360	160	670	400	290	T G G G A C C A G G A C C G T A G G A T G A T G T T C C T C C G C C A								29%	16%	30%
MICAL3-NM_001122731.2eU2626-NP_001116203.1aa851TI	0					0			2	9%	3%	290	90	680	320	230	C C A A A G G A C C C C T G C A G G A T G G C G C C A C C A C A G A T G								8%	11%	
MICAL3-NM_015241.3eU5589-NP_056056.2aa1745SF	0					0			2	7%	0%	350	210	650	390	370	A A C C C A A G T C C C T G T G G A A G T C C G T C T T C T C C G G G T								7%		7%
MICAL3-NM_015241.3eU8354-3'UTR+1990	0					0			3	17%	3%	390	150	730	170	280	T C A T C C T T G T C T G T C C A G A A G C T G C C A T C T C T C T C C								21%	15%	16%
MICA-NM_000247.3eU82-NP_000238.1aa15PL	4	12%	4%	14%	1%	0			2	4%	0%	170	230	660	280	290	C G G T C T T C C T G C T T C T G G C T G G C A T C T T C C C T T T T G	14%	14%	15%	6%				4%		4%
MICB-NM_001289160.2eU1996-3'UTR+836	0					0			2	5%	1%	350	310	690	460	410	T T T G T A T T T T T G T A G A G A C G G G G T T T C G C C A A G T T									4%	5%
MICOS10-NM_001032363.4eU162-NP_001027535.1aa32SL	0					0			3	11%	1%	410	330	690	130	500	G T A C T G G T T T T G G A T T A G G A A T T G T T T T C T C A C T T A								9%	11%	12%
MICU1-NM_001195518.2eU186-NP_001182447.1aa39LL	0					0			2	4%	1%	320	370	650	320	220	A T C C G G C G A A G A C T A A T G A T G G T G G C T T T C C T G G G A								3%		4%
MICU2-NM_152726.3eU278-NP_689939.1aa86RC	0					0			3	19%	2%	520	180	640	330	150	G G A A T A A T A T A T A T T G G G A A A C C G T C T C T T C G T A A G								17%	21%	18%
MIGA1-NM_001270384.2eU2230-3'UTR+321	0					0			2	15%	3%	490	200	470	540	260	A T T G G T T T G T C T T C T T G A A G C T G T C A T T C T C A A A A G								13%		17%
MIR4458HG-NR_039989.1eU199-ncr	0					0			3	32%	8%	520	140	680	330	190	A C T A T T T T G A G C A A A A A G A A G C A G T G T C T T C G G A A C								33%	23%	39%
MIS18A-NM_018944.3eU1241-3'UTR+504	0					0			3	13%	2%	400	190	480	450	400	G A T A A G A A A A A T C T T A G A G G T T T G T C T A C C C A C A G T								15%	12%	13%
MKNK1-NM_001135553.4eU231-5'UTR-25	4	69%	22%	80%	6%	3	59%	8%	3	69%	2%	280	210	830	140	470	G G G A T T T C A A G T T A C A G A T G G T A T C T T C T C A A A A G	87%	76%	77%	36%	50%	65%	61%	71%	67%	70%
MKNK1-NM_001377343.1eU189-5'UTR-56	4	63%	20%	72%	8%	3	63%	20%	3	67%	8%	280	210	830	140	470	G G G A T T T C A A G T T A C A G A T G G T A T C T T C T C A A A A G	64%	78%	75%	35%	78%	40%	70%	67%	75%	60%
MKRN1-NM_013446.4eU1795-3'UTR+185	0					0			3	6%	2%	210	280	530	390	420	C A C C T T T C C C T G A G G A G T G T G T T G T T T T C C C T G T T G								7%	7%	4%
MMP24OS-NM_001355003.2eU309-NP_001341932.1aa64LL	0					0			2	26%	12%	400	190	490	500	360	G T G G G G G C C G C T G G A C G A C G T G C G C T T C C T C A T C G C								34%		17%
MMP24OS-NM_001355004.2eU272-NP_001341933.1aa64LL	0					0			3	22%	5%	400	190	490	500	360	G T G G G G G C C G C T G G A C G A C G T G C G C T T C C T C A T C G C								22%	17%	27%
MOB1A-NM_001317110.2eU3652-3'UTR+2799	4	41%	13%	47%	4%	3	1%	0%	3	9%	1%	470	80	760	250	250	T T C C C C C T T G C T A A T C A G A A G C C A T C T C C T C T C A G T	52%	44%	45%	21%	2%	1%	2%	7%	9%	10%
MORC4-NM_001085354.3eU1120-NP_001078823.1aa293Q*	0					0			3	51%	15%	260	240	790	280	360	A T T T T T C T G C G T C A A A A G A A G G T G A C T A C C C A G A T G								33%	59%	60%
MORF4L1-NM_001265603.2eU960-NP_001252532.1aa228PL	0					0			2	2%	1%	490	360	470	530	130	T C A G T G C C A G C G A T T A T G A A G T G G C T C C T C C T G A G T								3%	2%	
MRE11-NM_001330347.2eU2053-NP_001317276.1aa632R*	0					1	6%		2	7%	4%	260	310	560	490	220	G C C T T T A A A T C T A C A A G A C A G C A G C C T T C C C G A A A T					6%			5%	10%	
MRE11-NM_005591.4eU2056-NP_005582.1aa633R*	0					2	8%	0%	2	7%	2%	260	310	560	490	220	G C C T T T A A A T C T A C A A G A C A G C A G C C T T C C C G A A A T						8%	9%		6%	9%
MRGBP-NM_018270.6eU485-NP_060740.1aa145SL	0					0			3	7%	1%	380	190	710	250	230	A C C C C C A C A A T G G G G C T G A C G A T G T T T T T C A T C T T								6%	7%	8%
MRPL13-NM_014078.6eU556-NP_054797.2aa167PL	0					0			3	5%	3%	250	310	690	370	260	A G T A C A C A C A A G A A G A A A T A G A C G C C T T C C C A A G A T								3%	8%	6%
MRPL18-NM_001318817.2eU258-NP_001305746.1aa46PL	0					0			3	37%	5%	250	150	700	330	270	T G G A C C C T G T G G A A A A T G A A G C T G T C G C C C C A G A A T								42%	38%	32%
MRPL18-NM_014161.5eU258-NP_054880.2aa46PL	0					0			3	40%	5%	250	150	700	330	270	T G G A C C C T G T G G A A A A T G A A G C T G T C G C C C C A G A A T								41%	45%	34%
MRPL18-NR_134862.1eU279-ncr	0					0			3	40%	3%	250	150	700	330	270	T G G A C C C T G T G G A A A A T G A A G C T G T C G C C C C A G A A T								38%	40%	43%
MRPL18-NR_134863.1eU279-ncr	0					0			3	46%	11%	250	150	700	330	270	T G G A C C C T G T G G A A A A T G A A G C T G T C G C C C C A G A A T								44%	36%	57%
MRPL18-NR_134866.1eU279-ncr	0					0			2	35%	11%	250	150	700	330	270	T G G A C C C T G T G G A A A A T G A A G C T G T C G C C C C A G A A T								27%	43%	
MRPL19-NM_014763.4eU5644-3'UTR+4740	0					0			3	41%	5%	260	70	660	350	130	T A T A T G T T G G T C T G T T T G A T G C T G T C T C A C C A G T T T								46%	36%	41%
MRPL44-NM_022915.5eU447-NP_075066.1aa146PL	0					0			3	13%	5%	380	150	700	240	100	G C C T T A C A C A G T T T C T T G A A G A C G A G T A C C C A G A C A								8%	13%	18%
MRPL46-NM_022163.4eU231-NP_071446.2aa74LL	0					0			3	12%	3%	370	150	730	250	400	A C C C C A T T G C A G G A A G A G A T G G C G T C T C T A C T G C A G								14%	8%	14%
MRPL49-NM_004927.4eU989-3'UTR+462	0					0			2	2%	0%	350	260	690	460	410	T T T G T A T T T T T A G T A G A G A T G G G G T T T C A C C A T G T T								2%	2%	
MRPL51-NM_016497.4eU679-3'UTR+166	0					0			3	34%	11%	560	60	590	340	190	A C T T T T G T T T T C C T C T A G A T G C T G C C T C T A C T C A T G								28%	28%	46%
MRPL52-NM_001318870.2eU491-3'UTR+132	0					0			2	15%	1%	410	150	510	440	270	A T G C T T C A T C C A G C A G A A G A T A G C C T C A C G T T C C								15%		14%
MRPL54-NM_172251.3eU297-NP_758455.1aa93PL	0					0			3	4%	1%	380	70	570	360	160	A T G T A C C C C T G A A A C C G G A T G C T G A G T A C C C T G A A T								5%	4%	3%
MRPS10-NM_018141.4eU1792-3'UTR+1174	0					0			3	12%	3%	360	300	360	650	240	A C T C T G T T T G T T A G G A G A T G T C T G C T T T T C C A T G A A								16%	11%	9%
MRPS16-NM_016065.4eU1404-3'UTR+828	0					0			3	8%	4%	350	260	690	460	410	T T T G T A T T T T T A G T A G A G A T G G G G T T T C A C C A T G T T								7%	5%	12%
MRPS16-NM_016065.4eU1537-3'UTR+961	0					0			3	9%	0%	350	260	640	460	410	T T T G T A T T T T T A G T A G A G A T G G G G T T T C A C C G T G T T								9%	9%	9%
MRPS23-NM_016070.4eU730-3'UTR+154	0					0			2	3%	1%	440	110	470	280	320	T T G A T C T C T A A G G T A T T A T G T T T G C C T T C T C T T A G T									2%	3%

MRPS2-NM_001371401.1eU1416-3'UTR+297	0					0			3	3%	1%	230	200	600	380	340	TGGCCAAGCTGCTAGAAGATGCTGCTGTCCCTGTGA							3%	3%	4%
MRPS30-NM_016640.4eU566-NP_057724.2aa184LF	0					0			3	7%	2%	250	160	610	140	390	TTGCCCTTCCTGGATCAGCTGGTGTCAACCCTCGTG							5%	7%	9%
MRPS31P5-NR_002816.2eU3021-ncr	0					0			3	41%	7%	420	250	820	260	250	CACAAGAGTATCTCAAGGAAGTAGTCTTCTCCCACA							33%	42%	48%
MRS2-NM_001286265.2eU1449-3'UTR+111	0					0			3	27%	14%	320	340	600	300	350	AGGCACTATGGCCCTTGCGAGTTGCTTTCTCAAACT							24%	42%	14%
MRTFB-NM_001308142.2eU1784-NP_001295071.1aa537Q*	0					0			3	37%	8%	250	70	770	230	190	ACCGAGATTATGACCATGATGTCGCCTTCACAGTTC							35%	29%	45%
MSANTD3-NM_001198812.1eU1605-3'UTR+207	0					0			3	46%	6%	380	300	670	220	430	TTATGTTTTTAAATACAGAAATTGCTTTCACAAATT							40%	51%	48%
MSANTD4-NM_001318747.1eU2971-3'UTR+1331	0					0			2	8%	2%	380	510	680	300	240	TTTTCCTTCAGCTCTCTGAAGGTGCTTTTTCCACAA							6%		9%
MSANTD7-NM_001378785.1eU222-5'UTR-249	0					0			3	7%	2%	320	60	580	220	220	ACCGAGTTACTCCAGCTGTTGTTGCTTACTCAGAAA							6%	9%	6%
MSI2-NM_001322250.2eU647-NP_001309179.1aa171PL	4	26%	11%	28%	12%	0			2	6%	1%	350	220	780	280	330	AGAAAGCTCAGCCGAAAGAAGTCATGTTCCCACCTG	15%	29%	39%	20%			5%	6%	
MSL3P1-NR_024322.1eU1845-ncr	2	22%	1%	22%	1%	0			3	17%	9%	310	160	680	250	280	AGCTATAATTTGCTTCTGACGGTTCCTTCTCTGTGA	22%	21%					10%	27%	15%
MSN-NM_002444.3eU390-NP_002435.1aa68Q*	0					0			3	30%	2%	210	240	790	280	320	TGGCTGAAACTCAATAAGAAGGTGACTGCCCAGGAT							31%	31%	28%
MTCH1-NM_001271641.2eU544-NP_001258570.1aa174PL	0					0			3	3%	1%	640	320	520	600	390	TGACTCGGGGTAGCATGAAGAAGGTTTTCCCTCCAG							3%	2%	3%
MTF1-NM_005955.3eU4412-3'UTR+2037	0					0			2	11%	5%	420	320	690	500	410	TTTGTATTTTTAGTAGAGACGGGGTTTCTCCATGTT							15%		8%
MTF1-NM_005955.3eU6131-3'UTR+3756	0					0			3	18%	6%	190	240	550	460	350	GCAATATGTGTAGCTTGATGGTCGTTGTCCCATGTG							22%	11%	22%
MTHFD1-NM_001364837.1eU2933-NP_001351766.1aa953PL	0					0			3	21%	3%	270	300	720	340	430	AAGATCTGAAACTAATAGTAGGAGTTTCCCCAGAAG							23%	18%	23%
MTHFD1-NM_005956.4eU3027-3'UTR+144	1	2%		2%		0			3	28%	6%	270	300	720	340	430	AAGATCTGAAACTAATAGTAGGAGTTTCCCCAGAAG	2%						23%	27%	34%
MTHFS-NM_006441.4eU153-NP_006432.1aa43HY	0					0			2	14%	13%	360	300	740	310	380	TCCCGCGTACTGAGCCAGAAGGTGATTGCCCACAGT							23%		6%
MTMR1-NM_001306144.3eU989-NP_001293073.1aa285SL	0					0			2	6%	2%	370	230	670	160	290	TGCCAACTAGTGTAAAAGATGATGACCTTTCAAAAG							8%	5%	
MTMR4-NM_001378066.1eU4520-3'UTR+740	0					0			3	8%	1%	330	110	630	370	290	AGTTATTTTGAGCTGTGAAGGCAGTCGTCTCTGTTA							7%	8%	8%
MTMR4-NM_001378066.1eU4769-3'UTR+989	0					0			2	9%	4%	400	0	580	300	160	ATGTTGTCTTTCCCTTGGCTGATGCCTCCTCTGGGT							7%		12%
MTO1-NM_001123226.2eU422-NP_001116698.1aa102RC	0					0			3	7%	3%	540	150	520	190	600	GAAGTAGATGCCTTGGATGGCCTGTGTTCTCGCATC							4%	7%	10%
MTOR-NM_001386500.1eU8270-3'UTR+472	0					0			2	2%	1%	250	220	650	160	400	ACTGTAAAGACACAGAAGATGCTGACCTCACCCCTG							2%		3%
MTRF1L-NM_001114184.3eU624-NP_001107656.1aa200HH	0					0			3	6%	1%	550	150	660	470	340	TAGGCACATGAAATTTGAAGGAGGTGTTCACAGAGT							7%	5%	5%
MTRF1L-NM_001301047.3eU516-NP_001287976.1aa164HH	0					0			2	13%	2%	550	150	660	470	340	TAGGCACATGAAATTTGAAGGAGGTGTTCACAGAGT							15%		12%
MTRFR-NM_001143905.2eU279-NP_001137377.1aa34PS	0					0			3	14%	11%	150	350	620	250	330	CGGCTTTGGGAGAAGCTGACGTTGTTATCCCCAGGA							7%	8%	27%
MTRFR-NM_152269.5eU224-NP_689482.1aa34PS	0					0			2	17%	9%	150	350	620	250	330	CGGCTTTGGGAGAAGCTGACGTTGTTATCCCCAGGA								10%	23%
MUL1-NM_024544.3eU1366-3'UTR+180	0					0			3	3%	0%	320	350	610	490	100	CATGCCTCCAATGGCAGGATGCTGCCTTTCCCACCT							2%	3%	3%
MVK-NM_000431.4eU1673-3'UTR+388	0					0			2	8%	4%	530	200	590	440	360	GGGCCTGGTTCCCGGAGAAGTGTGCCTTCTCTCTCC							5%	10%	
MX2-NM_002463.2eU1934-NP_002454.1aa612HY	0					0			3	7%	3%	210	200	690	120	270	ACGCCTTCACAGAATATGAAGTTGAACTCTCATTTT							5%	6%	10%
MXD4-NM_006454.3eU846-3'UTR+87	0					0			2	25%	3%	560	170	350	610	120	CAGTTCTCCTTCAGTTGACGCCAGCCTCTCCACAGG							24%		27%
MXRA7-NM_001008528.3eU1641-3'UTR+1007	0					0			3	17%	7%	260	220	690	380	410	TCTGTATTTTTAGTAGAGATGGAGTTTCACCATGTT							22%	9%	20%
MYBBP1A-NM_001105538.2eU830-NP_001099008.1aa257SF	0					0			3	10%	2%	440	40	540	550	220	TGGTGAATGTGCTGAAGATGGCCGCCTCCTCTGTGA							9%	8%	12%
MYCBP2-NM_015057.5eU12345-NP_055872.4aa4009SF	0					0			3	21%	6%	490	80	700	300	160	ATTCTCAGCCAAATGATGAAGATGCCTCCTCTGATG							23%	26%	14%
MYH10-NM_001256012.3eU6116-NP_001242941.1aa2005Q*	0					0			2	3%	1%	240	70	640	340	260	ACCAGTGATGTCAACGAGACGCAGCCACCCCAGTCA							2%	4%	
MYL6-NM_021019.5eU57-NP_066299.2aa5TI	0					0			3	2%	0%	300	300	470	350	450	AGAGCTGAGCAGTCAAGATGTGTGACTTCACCGAAG							2%	2%	2%
MYNN-NM_018657.5eU167-NP_061127.1aa23LL	0					0			2	6%	2%	520	210	560	500	470	ACTGAACAAACAGCGGGAAGCAGGTTTTCTCTGTGA							5%		7%
MYO10-NM_012334.3eU6236-NP_036466.2aa1919SF	0					0			2	4%	0%	400	80	760	330	210	TGGACATGTGGATTAAGGAAGAAGTCTCCTCTGCTC							4%		4%
MYO1B-NM_001130158.3eU3639-NP_001123630.1aa1136PL	0					0			3	18%	1%	280	280	600	300	260	AAAACAACCGTCTCCTTGAAGTTGCTGTCCCTTAAC							17%	18%	19%
MYO1B-NM_001130158.3eU3656-3'UTR+13	0					0			2	3%	0%	460	130	620	300	300	GAAGTTGCTGTCCCTTAACTGGCGCCTCCTCTCTAC							2%		3%
MYO1E-NM_004998.4eU218-5'UTR-155	0					0			2	7%	2%	300	140	690	230	320	ACCGCCAATCCCGCCTGGATGGAGTCCCCTCTGGGT							9%	6%	
MYOF-NM_013451.4eU1273-NP_038479.1aa378SL	0					0			3	12%	2%	500	70	770	270	240	AGGACATCCCCCAGATGGATGATGCCTTCTCACAGA							11%	14%	11%
MYSM1-NM_001085487.3eU713-NP_001078956.1aa235Q*	0					0			3	36%	6%	280	280	750	100	520	ATCACAGATGAGGTGGACGAGTTGTCTTCTCAAACA							30%	41%	37%
NAA50-NM_001308445.2eU1736-3'UTR+929	0					0			3	16%	3%	270	300	770	210	490	CCCTAAATAAACCTGAAGCAGGTGTTTTCTCTTGGA							20%	14%	15%
NAP1L4-NM_001369375.1eU346-NP_001356304.1aa12SL	1	7%		7%		0			3	18%	2%	470	300	660	340	360	CAGATCACAGTTTTTCAGATGGGGTTCCTTCAGATT	7%						19%	17%	19%
NAP1L4-NM_001369376.1eU271-NP_001356305.1aa12SL	0					0			2	20%	7%	470	300	660	340	360	CAGATCACAGTTTTTCAGATGGGGTTCCTTCAGATT							16%	25%	
NAP1L4-NM_001369380.1eU128-NP_001356309.1aa12SL	0					0			3	21%	3%	470	300	660	340	360	CAGATCACAGTTTTTCAGATGGGGTTCCTTCAGATT							23%	17%	22%
NASP-NM_001195193.2eU2028-NP_001182122.1aa641SL	4	3%	1%	4%	1%	0			3	40%	2%	360	150	740	330	420	CCAGTAGAAAGCCAACAGACGGTGCTTCCTCATCAA	3%	5%	4%	1%			37%	41%	41%
NASP-XM_011541509.2eU2290-XP_011539811.1aa707SL	2	7%	2%	7%	2%	0			3	46%	17%	360	150	740	330	420	CCAGTAGAAAGCCAACAGACGGTGCTTCCTCATCAA		8%	6%				29%	44%	63%
NAV3-NM_001024383.2eU1821-NP_001019554.1aa418SF	0					0			2	13%	2%	510	130	650	270	100	GTGATGGTGGGAAGGATGATGATGCCTTTTCTGAAT							14%	11%	
NBAS-NM_015909.4eU5557-NP_056993.2aa1843PS	0					0			2	4%	0%	300	470	510	480	380	ATCCCTGAAAAGGATGGACAGATGCTTTCCCCAAGC							4%		4%
NBL1-NM_001204084.3eU2107-3'UTR+1301	0					0			3	9%	1%	230	70	620	100	370	ACAGTCTCCCTGATGGACCCTCAGTCTTCTCATGAA							8%	9%	10%
NBN-NM_001024688.3eU730-NP_001019859.1aa110Q*	0					0			3	31%	6%	350	130	750	340	230	AAAGCAGTTGAGTCCAAGAAGCAGCCTCCACAAATT							38%	32%	25%
NCAPD2-NM_014865.4eU4173-NP_055680.3aa1366SL	0					0			3	15%	0%	270	240	790	220	310	CTTCCAAAAAGAAACCCAAAGTTGTCTTCTCAAGTG							15%	16%	15%
NCAPH-NM_015341.5eU168-NP_056156.2aa35PL	0					0			2	6%	2%	370	280	680	290	510	GTGCCTCCTCTCCTTCAGAGCGTGTGTTCCCGATGC								7%	4%
NCEH1-NM_001146276.3eU1684-3'UTR+350	0					0			3	19%	7%	220	390	680	330	320	TTAGGACGTTCTTTTTAGAAGCAGTTCTTCCAGCTC							13%	18%	27%
NCKAP1-NM_013436.5eU2021-NP_038464.1aa555Q*	0					0			3	3%	1%	300	140	690	280	400	TATAGTCGTGCTTTTGAGAAGATGTTTCAACAGTGT							2%	2%	4%
NCL-NM_005381.3eU334-NP_005372.2aa68PL	0					0			3	4%	0%	300	180	640	550	310	CAACCTCAGCAAAGAAGGTGGTCGTTTCCCCAACAA							4%	4%	4%
NCLN-NM_001321463.2eU153-NP_001308392.1aa18LL	0					0			3	8%	1%	310	240	830	140	190	GTGCTGGAGAACATGCTGAAGGCGTCTTGTCTGCCG							7%	8%	9%
NCOA4-NM_001145260.2eU3485-3'UTR+1371	0					0			3	3%	1%	320	370	660	410	160	GGTGGGCAAAAACAATGTAAGGAGCCTTTCCAGTTA							3%	4%	4%
NCOA6-NM_001242539.2eU2105-NP_001229468.1aa686Q*	0					0			3	18%	3%	250	280	680	360	340	AGGATGACCCCACCCAAGCAGATGCTTTCCCAGCAG							17%	21%	15%
NCOA7-NM_001122842.3eU5192-3'UTR+2224	0					0			3	31%	4%	390	120	770	180	350	TTGTGAGAGCATATCCAGATGCTGTGTTCTCTATGT							29%	36%	29%
NDC1-NM_001168551.2eU933-NP_001162023.1aa300Q*	4	8%	3%	9%	2%	0			3	72%	1%	310	180	720	260	260	TTAGCCTTGCAGGACCTGATGTTGCTTTCTCAATAT	11%	8%	9%	3%			71%	71%	72%
NDE1-XM_017023349.2eU1083-XP_016878838.1aa329HY	2	4%	1%	4%	1%	0			3	35%	6%	400	150	810	310	270	AAGCGCCTGGAATTTGGGAAGCCGCCTTCACACATG	4%		3%				34%	30%	41%
NDE1-XM_017023353.2eU1215-3'UTR+157	0					0			3	30%	14%	400	150	810	310	270	AAGCGCCTGGAATTTGGGAAGCCGCCTTCACACATG							43%	30%	15%
NDFIP1-NM_030571.4eU1287-3'UTR+425	0					0			3	4%	0%	410	430	780	370	390	ATAATGCTGGCCATTTTAAAGGGGTTTTCTCAAAAG							4%	4%	3%
NDUFB2-NM_004546.3eU367-3'UTR+12	0					0			3	20%	1%	210	170	730	80	350	TCCTGATGATGAAGACTGAAGGTGTAGACTCAGCCT							20%	19%	19%
NDUFB2-NM_004546.3eU91-NP_004537.1aa18FF	0					0			3	7%	2%	510	130	740	440	80	GTCTTTCGCTCGCGTTGGAGGCCGCCTTTTCAGAAG							8%	8%	4%
NDUFB7-NM_004146.6eU143-NP_004137.2aa26PL	4	31%	9%	36%	2%	0			3	8%	1%	350	180	350	540	260	AGCCCGACCCCCTGCAGATGCCAACCTTCCCGCCAG	37%	35%	34%	18%			9%	7%	9%
NDUFS1-NM_001199981.2eU1118-NP_001186910.1aa337PL	0					0			3	14%	0%	340	410	780	320	250	CTGACACCTTATGCACTGAAGAGGTCTTCCCCACTG							14%	13%	14%
NDUFS1-NM_001199981.2eU1952-NP_001186910.1aa615PL	0					0			3	3%	2%	460	260	650	430	70	AAGTAAGGAACAGATTGGAAGAAGTCTCTCCTAATC							1%	5%	2%
NDUFS3-NM_004551.3eU302-NP_004542.1aa95PL	4	6%	2%	7%	1%	0			3	49%	2%	150	230	800	180	290	AGGTCTGTATCCATCCTGATGGCGTCATCCCAGTGC	8%	5%	7%	3%			50%	47%	51%
NDUFS3-NM_004551.3eU852-3'UTR+39	0					2	5%	2%	3	16%	3%	430	250	680	150	200	AACGCATGTGGATCCTAGACAGCGCCTTATCTATGA					4%	6%	17%	19%	13%
NDUFV2-NM_021074.5eU287-NP_066552.2aa81PL	0					0			3	5%	1%	180	390	600	290	380	CAGAAGGCCATAAAGCAGCAGCTGTTCTTCCAGTCC							4%	4%	6%
NEAT1-NR_028272.1eU1540-ncr	0					0			2	2%	1%	520	110	580	340	140	TGGGGTAAAGGTTTTCAGATGCTGCATCTTCTAAAT							1%		2%
NECAP2-NM_001145277.2eU801-NP_001138749.1aa255HY	0					0			3	11%	1%	300	280	710	350	390	CCTCATGTGACTTCTGGGAAGGCGCTCCCTCATCTG							12%	10%	13%
NECAP2-NM_001145278.2eU799-3'UTR+47	0					0			3	15%	8%	300	280	710	350	390	CCTCATGTGACTTCTGGGAAGGCGCTCCCTCATCTG							22%	15%	6%
NECTIN3-XM_017006123.1eU6925-3'UTR+5069	0					0			3	37%	12%	210	270	780	240	270	CAAATGCTAGCAGCCCTGAAGTTGTATTCCCAATTA							23%	44%	43%
NEIL2-NM_001135746.3eU1649-3'UTR+566	0					0			3	9%	3%	350	270	800	320	390	TGTTTACTTTTTAGAGAGATGGGGTCTTGCCATATT							13%	8%	7%
NENF-NM_013349.5eU413-NP_037481.1aa130TI	0					0			3	4%	1%	390	260	740	300	260	AGGAACTGGAGGCCCTGGATGAGGTCTTCACCAAAG							5%	3%	5%
NET1-NM_001047160.3eU1084-NP_001040625.1aa316VV	0					0			3	37%	5%	360	130	720	190	0	TTTCCTAGATATCCCTCGAAGTCGCCTAGTCAAATA							32%	39%	41%
NEU1-NM_000434.4eU1480-3'UTR+187	0					0			3	21%	4%	320	390	670	160	400	TCCTTTAGGACTGAAAGCTAGGCGTCCTCTCCCACA							16%	24%	22%
NEU1-NM_000434.4eU1522-3'UTR+229	0					0			3	5%	1%	410	280	550	480	210	TCCTGCCCTCATCTGAGAATACTGTCTTTCCATATG							3%	5%	6%
NEURL4-NM_001005408.2eU5088-3'UTR+382	0					0			3	21%	2%	380	370	640	380	160	TCTCCCTGTGCCCACATGAAGCTGCTTTTTCCGGGG							21%	20%	23%

NEXN-NM_001172309.2eU1069-NP_001165780.1aa294PL	0					0			3	37%	6%	350	70	710	310	240	GGAGAAATATGGTAGTAGATGATGACTCCC	C	AGAGA								35%	32%	43%
NEXN-NM_001172309.2eU259-NP_001165780.1aa24SF	0					0			3	15%	2%	520	190	640	370	130	TTGCTTCTGATGATGAGGAAGATGTATCTT	C	TAAAG								13%	16%	15%
NEXN-NM_144573.4eU451-NP_653174.3aa88SF	0					0			3	15%	5%	520	190	640	370	130	TTGCTTCTGATGATGAGGAAGATGTATCTT	C	TAAAG								11%	21%	15%
NFKB2-NM_001077494.3eU910-NP_001070962.1aa214SF	0					0			2	4%	2%	440	210	540	330	330	CCTTCTCCCTGCCCCTGAAGCCAGTCATCT	C	CCAGC								5%	3%	
NFX1-NM_001318758.2eU319-NP_001305687.1aa88Q*	0					0			3	15%	2%	280	280	760	240	190	AGCAAACCTAAGAGTCAGCAGACGTCTTTC	C	AGTCC								13%	16%	15%
NFYA-NM_002505.5eU2726-3'UTR+1510	0					0			2	12%	1%	390	70	560	400	240	ATGATATTTCAGAAGAGTTGGCTGCCTTCT	C	TGGAA									12%	13%
NFYC-NM_001142587.2eU1670-3'UTR+493	0					0			3	3%	1%	440	270	610	300	240	GAATTAGGTGAATGTGTGTAGCTGCTTTTT	C	ACTCG								2%	4%	4%
NGDN-NM_001042635.2eU147-NP_001036100.1aa45PL	0					0			2	4%	2%	320	270	550	330	220	TGACACAAAAAGTTCAAGCTGGTGCCTATC	C	TACAG								5%		2%
NHP2-NM_001034833.2eU525-3'UTR+184	4	41%	14%	48%	1%	3	7%	1%	3	19%	2%	170	230	790	240	330	TCCTGCCCACCCACACTGACGGCATCTTCC	C	AGTTC	49%	49%	47%	20%	8%	6%	8%	19%	18%	21%
NHP2-NM_017838.4eU631-3'UTR+101	4	46%	13%	52%	2%	3	7%	3%	3	26%	5%	170	230	790	240	330	TCCTGCCCACCCACACTGACGGCATCTTCC	C	AGTTC	55%	52%	51%	27%	4%	9%	8%	23%	23%	31%
NIBAN1-NM_052966.4eU1530-NP_443198.1aa460PS	0					0			2	5%	1%	210	430	630	320	420	GCAGTGTTCACTTTTGAGCAGTTGCTTTCC	C	CACAT									5%	4%
NIBAN2-NM_001035534.3eU143-NP_001030611.1aa17Q*	0					0			3	13%	9%	270	330	580	360	280	AAAACCGGGAAGATCCTGACGGAGTTCCTC	C	AGTTC								6%	11%	23%
NIBAN2-NM_022833.4eU287-NP_073744.2aa30Q*	0					0			3	19%	1%	270	330	580	360	280	AAAACCGGGAAGATCCTGACGGAGTTCCTC	C	AGTTC								20%	18%	19%
NIP7-NM_001199434.2eU1567-3'UTR+1107	0					0			3	16%	7%	270	210	780	250	410	TATTACCTTCTTTTTTACAAGCTGTTTTCT	C	AGTAT								9%	15%	23%
NISCH-XM_006712955.3eU1703-5'UTR-209	0					0			3	51%	13%	270	260	820	250	330	CAACTTGTAGCCCCTCTGAAGCTGTTTTCT	C	AACTG								40%	65%	48%
NKAPD1-NM_001082970.2eU485-5'UTR-143	0					0			2	18%	2%	640	170	570	520	260	GCAGAGTAACCAGTGGGAAGGCTGCGTTTT	C	ACGAA								16%	19%	
NKAP-NM_024528.4eU708-NP_078804.2aa188RC	0					0			3	16%	6%	250	150	690	280	190	TCAGAAGAAGAAAAAAAGAAGAAGTCTAGC	C	GTTCA								12%	23%	14%
NKTR-NM_001349124.2eU3464-NP_001336053.1aa1116PP	0					0			2	6%	0%	490	270	570	450	290	AAACATTCAGCACGTTGAAGAAAGTGTTCC	C	AATGG									6%	6%
NLGN2-NM_020795.4eU3214-3'UTR+295	0					0			3	21%	7%	500	340	510	430	590	GGTATTTTCCCGCGTGGAGGTGTGCTTTCT	C	ACAAC								18%	15%	29%
NLGN2-NM_020795.4eU961-NP_065846.1aa184HY	0					0			2	7%	2%	330	190	610	460	270	CCTGGGAAGAAGCCTGTGATGCTGTTTCTC	C	ATGGC								5%		8%
NMD3-NM_015938.5eU2431-3'UTR+800	4	59%	17%	67%	6%	3	5%	2%	3	21%	2%	370	300	770	210	510	TTTTGTTTATAAATTGAGAAGGAATTTTCT	C	TCTAT	73%	62%	67%	35%	7%	5%	3%	23%	22%	20%
NME6-NM_001308426.2eU226-NP_001295355.1aa68FF	0					0			3	14%	7%	460	370	660	500	370	GTTTTACCGAGAGCATGAAGGGCGTTTTTT	C	TATCA								10%	10%	22%
NMNAT1-NM_001297778.1eU1488-3'UTR+489	0					0			2	38%	1%	420	200	690	250	360	TGTTTATTTGTTTTTGAGATGGAGTCTCTA	C	TAAAA								38%	37%	
NMNAT1-NM_001297778.1eU3154-3'UTR+2155	0					0			3	11%	2%	450	380	640	480	300	AGGCCTTTCAGATAAGAGAAGTGGCTTTTC	C	TTGGT								13%	10%	10%
NMT1-NM_021079.5eU950-NP_066565.1aa313HY	0					0			3	4%	1%	250	370	550	290	630	CCACGGAAGCTGATTGAAGTGAAGTTCTCC	C	ACCTG								4%	3%	4%
NOCT-NM_012118.4eU1740-3'UTR+243	0					0			3	5%	2%	350	260	640	460	410	TTTGTATTTTTAGTAGAGACGGGGTTTCAC	C	GTGTT								4%	8%	3%
NOL4L-NM_001256798.2eU2995-3'UTR+509	0					0			3	43%	8%	430	110	600	600	310	TGTGTCTGTCTGATCAGAAGGATGTATCCC	C	ATACC								33%	44%	50%
NOM1-NM_001353366.2eU1588-NP_001340295.1aa521SL	0					0			3	15%	4%	450	130	690	300	280	GTTTTTCATTGAGGAAAGATGATGCTTTAT	C	ACTTA								13%	12%	20%
NOM1-NM_001353366.2eU5292-3'UTR+2680	0					0			3	5%	2%	380	270	640	290	200	GTTTGAGGTACAGAATTGAAGCTGATTTTT	C	TGCAA								5%	3%	7%
NOM1-XR_927512.3eU1575-ncr	0					0			2	27%	3%	450	130	690	300	280	GTTTTTCATTGAGGAAAGATGATGCTTTAT	C	ACTTA									29%	25%
NONO-NM_001145408.2eU2435-3'UTR+752	4	63%	19%	73%	2%	3	6%	1%	3	20%	0%	360	210	770	250	390	CCCCTTCATGTCCTAAAGAAGACATTTTCT	C	TTAGA	74%	73%	70%	35%	7%	7%	6%	21%	21%	20%
NONO-NM_001145408.2eU773-NP_001138880.1aa169SF	0					0			3	7%	1%	460	210	690	310	40	TGTCCAACGAACTGCTGGAAGAAGCCTTTT	C	TGTGT								6%	6%	8%
NOP2-NM_001033714.3eU2661-3'UTR+77	0					0			3	17%	3%	470	120	720	280	320	GGAACTCTTGCCTCTGTGAGGATGCCTTCT	C	TACTG								14%	17%	20%
NOP2-NM_001033714.3eU293-NP_001028886.1aa46RC	0					0			2	5%	1%	350	0	690	310	250	AGTGACGAAAATTCCAAGAGGCTGTCTAGT	C	GTGCT									5%	4%
NOP56-NM_006392.4eU1644-NP_006383.2aa539PS	0					0			3	4%	1%	400	180	720	260	220	ACCAGTATTCCCAAGAGGAAGAAGTCTACA	C	CCAAG								4%	6%	4%
NORAD-NR_027451.1eU5202-ncr	0					0			3	7%	3%	500	110	540	270	250	TCAGTCCCATAAAATTGGATGTTGTGCCTA	C	ACACA								6%	10%	4%
NPAT-NM_001321307.1eU5510-3'UTR+1103	0					0			2	19%	2%	310	360	640	350	410	GGAGAGTGAGAGATGAGAGAGGTGCCTTCT	C	TGTAT									17%	20%
NPDC1-NM_015392.4eU1045-NP_056207.3aa286TM	0					0			3	37%	14%	210	160	720	260	270	CGGATGAGGAGAATGAGGACGGAGACTTCA	C	GGTGT								44%	47%	21%
NPEPPS-NM_001330257.2eU221-NP_001317186.1aa56PL	0					0			3	8%	4%	260	220	600	270	230	CCTTCGAGCGGCTGCCTGCCGATGTCTCCC	C	CATCA								4%	11%	9%
NPEPPS-NM_006310.4eU372-NP_006301.3aa60PL	0					0			3	7%	4%	260	220	600	270	230	CCTTCGAGCGGCTGCCTGCCGATGTCTCCC	C	CATCA								7%	3%	12%
NQO1-NM_000903.3eU1771-3'UTR+825	0					0			3	15%	5%	260	220	730	380	410	TTTGTATTTTTAGTAGAGACGGCGTTTCAC	C	ATGTT								16%	20%	10%
NR2C1-NM_003297.4eU2328-3'UTR+270	0					0			3	10%	4%	300	80	690	190	230	TGGTGAAGATGATACCTGAAGCTGTCACCT	C	TTGAT								9%	7%	14%
NR3C1-NM_000176.3eU5191-3'UTR+2368	0					0			2	12%	4%	290	260	640	280	290	GGAATGTAATATTTTAAGATGGTGTGTAAC	C	CGGCT								9%		15%
NRGN-NM_001126181.2eU436-3'UTR+56	0					0			3	11%	3%	420	70	610	480	140	GTTCCCGAGGAGAGATGGATGCCGCGTCCC	C	TTCGC								10%	9%	15%
NRGN-NM_006176.3eU433-3'UTR+53	0					0			3	9%	3%	420	70	610	480	140	GTTCCCGAGGAGAGATGGATGCCGCGTCCC	C	TTCGC								9%	6%	11%
NRXN3-NM_001105250.3eU2370-3'UTR+78	0					0			2	45%	7%	440	160	690	160	280	ACGCCTATGAATCTTTGGACGGTGAGATCT	C	ACAGA									40%	50%
NSD2-NM_001042424.3eU1367-NP_001035889.1aa396PP	0					0			3	3%	0%	410	310	550	540	360	CCCCAAGTCTGTGAGAGAAGAGTGCATTCC	C	ATGAA								3%	3%	3%
NSFL1C-NM_001206736.2eU1307-3'UTR+163	2	2%	0%	2%	0%	0			3	46%	1%	310	270	480	380	490	TCTTAGATCTTGGTTGGACGTTTGTTTTCT	C	CTTAG	2%		2%					47%	45%	47%
NT5C2-NM_001134373.3eU1571-NP_001127845.1aa504LF	2	3%	1%	3%	1%	0			3	32%	6%	360	90	800	210	410	CACGTAGATATCAATGAGATGGAGTCTCCT	C	TTGCC		2%	4%					25%	35%	37%
NT5C3A-NM_001002009.3eU1100-NP_001002009.1aa291SF	0					0			2	9%	4%	290	130	670	130	180	TACAAGATGAATCATTAGAAGTAGCCAACT	C	TATTT								12%	7%	
NT5C3B-NM_052935.5eU615-NP_443167.4aa192LL	0					0			2	3%	0%	520	400	560	500	390	CTACATGGATTTTAATGAAGATGGTTTTCT	C	CAGGG								3%	4%	
NT5DC3-NM_001031701.3eU3180-3'UTR+1455	0					0			3	20%	7%	420	320	690	500	410	TTTGTATTTCTAGTAGAGATGGGGTTTCTC	C	ATGTT								25%	13%	24%
NTPCR-NM_032324.3eU506-NP_115700.1aa136PS	0					0			3	10%	2%	210	220	720	280	310	ATTCAAGCTGTTCGTCAGACGCTGTCTACC	C	CAGGG								12%	8%	11%
NUBP1-NM_001278506.2eU1093-3'UTR+141	4	23%	6%	26%	1%	0			2	4%	0%	320	210	720	300	200	AGATGCTGGTGTGGTCCGAAGCCACTTTCT	C	AGAGA	27%	27%	25%	14%					4%	4%
NUCB1-NM_006184.6eU1976-3'UTR+518	0					0			3	3%	2%	340	330	640	150	500	ACCAGTGATGCCTCAAAGACAGTGTCCCCT	C	CACAG								2%	2%	6%
NUCKS1-NM_022731.5eU5895-3'UTR+4954	1	3%				1	2%		2	2%	0%	370	200	420	230	230	AAAAGAAGCCTTTATTGTTGAGCATGTTGG	C	ATTGT				3%			2%	1%		2%
NUDT19-NM_001105570.2eU2671-3'UTR+1403	0					0			3	10%	2%	350	260	690	460	410	TTTGTATTTTTAGTAGAGACGGGGTTTCAC	C	ATGTT								11%	8%	11%
NUDT21-NM_007006.3eU3283-3'UTR+2469	0					0			3	5%	1%	520	230	490	460	310	CCCTTTGGAGAAGAGTGAAGTTTGTTTTTT	C	GCCAT								3%	6%	5%
NUFIP1-NM_012345.3eU330-NP_036477.2aa99LF	0					0			2	5%	1%	260	150	470	270	460	GCGCAACCCCCCTTCGACGCCCAGTCTCCC	C	TTGAT									5%	6%
NUFIP2-NM_020772.3eU3300-3'UTR+1123	2	5%	2%	5%	2%	0			3	66%	3%	420	300	830	180	330	AATAAACCCTGACTGTAGAAGGTGTGTTTT	C	ATGCA		7%	4%					69%	66%	63%
NUMA1-NM_001286561.2eU854-NP_001273490.1aa190SF	0					0			3	3%	1%	480	40	580	550	240	GCTTCCTAGAGCTACAGAAGGTTGCCTCCT	C	TTCCA								4%	2%	4%
NUP107-NM_001330192.2eU838-NP_001317121.1aa212AV	0					0			2	3%	1%	290	280	730	220	350	AGTCTGCATTAGAAGAGGAAAGTGTATTCG	C	AGTTA									2%	3%
NUP155-NM_001278312.2eU458-NP_001265241.1aa110PL	0					0			3	4%	1%	470	270	570	450	470	TGCAGTGTAATTGCATGATGGGTGTGTTCC	C	TCCTA								3%	4%	4%
NUP210-XM_005264994.3eU2974-XP_005265051.1aa961Q*	0					0			3	47%	13%	390	130	820	340	490	TCCAGCTGTCTTGCAAAGAGGATGTTTTCT	C	AGCGC								58%	50%	33%
NUP43-NM_198887.3eU659-NP_942590.1aa209Q*	0					0			3	6%	1%	360	280	680	310	380	GATTTCAGACAACAAGGAAATGAGCCTTCT	C	AGATA								7%	6%	6%
NUP58-NM_001008564.2eU235-NP_001008564.1aa29SF	0					0			2	7%	1%	230	400	770	170	560	CCGGCGGGACCAGCACAGGCGGCGTTTTCT	C	CTTCG									6%	8%
NUP62-NM_153719.4eU202-5'UTR-211	0					0			2	9%	5%	520	170	500	390	270	CAGGCGTGCCGAGGTGGATGTCCGTCTTTT	C	TCTGT								5%		12%
NUP98-NM_001365125.2eU2263-NP_001352054.1aa694HY	0					0			2	3%	1%	420	370	630	180	400	CTGGAAGGAAGCAGTGAAGAAACGTCTTTT	C	ATGAT									4%	2%
NUP98-NM_001365125.2eU3438-NP_001352054.1aa1085PP	0					0			2	7%	2%	200	230	630	380	360	AAGTGTCTCTGTGCAAGAATGTCGTACTCC	C	AGAGC								5%		8%
NUP98-NM_001365126.2eU3396-NP_001352055.1aa1071PP	0					0			3	8%	3%	200	230	630	380	360	AAGTGTCTCTGTGCAAGAATGTCGTACTCC	C	AGAGC								10%	10%	4%
NUS1-NM_138459.5eU3170-3'UTR+2086	0					0			3	6%	1%	270	200	690	220	190	CTAGGATTCTGATTTCAGATGTAGTCATTC	C	AGAAC								6%	6%	5%
NUS1-NM_138459.5eU779-NP_612468.1aa193PS	0					0			3	23%	5%	250	400	680	200	460	AATTGCCATTTGGCAGTGAAGGTGCTGTCT	C	CGGAA								21%	19%	28%
NUSAP1-NM_001243142.2eU520-NP_001230071.1aa148SL	0					0			3	6%	1%	320	100	640	520	330	AGCACCAAGAAGCTGAGAATGCTGTTTCCT	C	AGGTA								7%	6%	5%
NUSAP1-NM_001243143.2eU478-NP_001230072.1aa134SL	0					0			2	9%	3%	320	100	640	520	330	AGCACCAAGAAGCTGAGAATGCTGTTTCCT	C	AGGTA									11%	7%
NUSAP1-NM_001243144.2eU454-NP_001230073.1aa126SL	0					0			3	7%	2%	320	100	640	520	330	AGCACCAAGAAGCTGAGAATGCTGTTTCCT	C	AGGTA								5%	7%	10%
NUTF2-NM_001322038.2eU347-NP_001308967.1aa59LF	0					0			3	5%	0%	250	150	690	240	290	AAAGCTGCCATTGTGGAGAAGTTGTCTAGC	C	TTCCG								5%	5%	5%
NXF1-NM_001081491.2eU3775-3'UTR+2620	0					0			3	5%	0%	240	200	690	290	320	ATCGTAGTCATGCCTCAGAAGCAGTCCCCC	C	TGTAA								5%	5%	6%
NXN-NM_001205319.1eU2060-3'UTR+1015	0					0			2	9%	3%	350	260	690	460	410	TTTGTATTTTTAGTAGAGATGGGGTTTCAC	C	ATGTT								7%		11%
OAS1-NM_001032409.3eU763-NP_001027581.1aa229Q*	0					0			3	9%	2%	300	80	680	320	240	TGTAAGAAGAAGCTTGGGAAGCTGCCACCT	C	AGTAT								6%	10%	11%
OAS3-NM_006187.4eU3497-3'UTR+176	0					0			2	2%	0%	190	330	560	300	450	CATGTGTGTGTTTTAGTGAATCTGCTCTCC	C	AGCTC								2%	2%	
OAS3-NM_006187.4eU5793-3'UTR+2472	0					0			3	2%	1%	280	290	370	500	460	GACTTGACTGCATAAAGATGTCAGTTCTCC	C	TGAGT								2%	2%	1%
OAT-NM_000274.4eU1266-NP_000265.1aa396R*	0					0			3	12%	3%	410	280	640	330	260	AAAGATTGGGATGCTTGGAAGGTGTGTCTA	C	GACTT								9%	15%	13%
OBI1-NM_024546.4eU1989-NP_078822.3aa652Q*	0					3	18%	3%	3	19%	4%	290	330	730	270	430	CCAAGCTGCTTGTTTCAGACAGAGTTTTCC	C	AGGGC					14%	20%	20%	15%	23%	20%

OCLN-NM_001205254.2eU3545-3'UTR+1797	0					0			2	5%	1%	420	320	690	500	410	T T T G T A T T T T T A G T A G A G A T G G G G T T T C T C C A T G T T									6%	5%
ODF2-NM_001242352.2eU3586-3'UTR+870	0					0			2	13%	1%	210	220	640	340	390	A T T T C A T T T T A G C T C A G G T T G G T G C T T T C A C A T T G T								13%		14%
OFD1-NM_001330209.2eU531-NP_001317138.1aa69LL	0					0			2	14%	4%	470	240	500	560	390	T C G G T C C A T T T C A G T A G A A G G A G C T C C C T C T T A A T									12%	17%
OGFRL1-NM_001324266.2eU6978-3'UTR+5493	0					0			3	19%	4%	430	250	620	270	380	T T A A T T A A A A A A T T A G G G A A G T T G T T C A T T C A C A G C								14%	22%	20%
OPA1-NM_001354663.2eU3802-3'UTR+688	0					0			3	21%	2%	330	130	740	210	410	C T T A C C T A T T T G A T T C A G T T G C T G T T T T C T C A C T C T								19%	20%	23%
ORC1-NM_001190818.2eU309-NP_001177747.1aa43TT	0					0			3	9%	4%	280	270	410	610	340	A A T G T G T G T G A A A A C A G A A G G T T G T T C C A C C G A G A T								5%	10%	12%
ORC1-NM_001190818.2eU986-NP_001177747.1aa269SL	0					0			3	2%	0%	450	230	680	430	260	C A G G A A G A A T A A A A C G G A A A G T G G C C T T C T C G G A G A								2%	2%	2%
ORC1-NM_001190819.2eU312-NP_001177748.1aa43TT	0					0			2	5%	1%	280	270	410	610	340	A A T G T G T G T G A A A A C A G A A G G T T G T T C C A C C G A G A T								4%	6%	
OSBPL6-NM_001201480.2eU1104-NP_001188409.1aa197HH	0					0			3	15%	5%	300	170	490	420	440	T G T G A G A T C A C C A A G A G A T G C T A G T T T T C A C A T A T T								19%	15%	10%
OSBPL9-NM_001330580.2eU2421-3'UTR+253	4	5%	2%	5%	1%	0			3	68%	5%	310	170	820	140	350	T C A G G T T T T G A A A A T C A G A T G A T G T C T T C T C C T T T T	5%	7%	4%	3%				68%	64%	73%
OSER1-NM_016470.8eU617-NP_057554.4aa167SF	0					0			3	2%	0%	530	40	580	500	220	T C C C A T C A G A G A G T A A G A A G G A A G A C T C C T C T G A C G								2%	2%	2%
OSMR-NM_001323505.2eU1420-NP_001310434.1aa431SL	0					0			3	12%	5%	350	190	630	310	290	A G A A C T T C A C C A C A C T T G A A G C T G C T C C C T C A G A G G								7%	17%	12%
OTUD6B-AS1-NR_110438.1eU320-ncr	0					0			3	14%	5%	520	120	650	220	320	C A G C G C T A A A T A A T C G A G A A A A T G C C T C C T C T T G A A								9%	18%	15%
OTUD6B-NM_001286745.3eU1462-3'UTR+399	0					0			3	51%	9%	250	260	720	310	230	T C A A T G T A A T T T G T C T T G A A G A A G T T T A C C C A A T A T								61%	50%	43%
OTUD7B-NM_020205.4eU374-NP_064590.2aa10SL	0					0			2	7%	1%	260	180	640	270	340	A C A T G A C C C T G G A C A T G G A T G C T G T T C T G T C A G A T T								7%	6%	
OTULIN-NM_138348.6eU2205-3'UTR+1038	0					0			2	6%	2%	460	90	670	380	400	C T T T T C A T G C T G T G T T G G A G G G T G T G T C C A C T G C C A									5%	7%
OVCA2-NM_080822.3eU277-NP_543012.1aa84SF	0					0			3	6%	3%	270	230	690	290	270	G G T T T T C A G A G C A G G A G G C C G A C G T T T T C T C C G C A T								4%	9%	5%
OXR1-NM_001198532.1eU1121-NP_001185461.1aa341TI	0					0			2	13%	2%	380	150	780	140	320	C T G A G G A G T C T C T T T C T G A A G A T G T G T T C A C A G A A T									14%	11%
OXR1-NM_181354.4eU432-NP_851999.2aa85Q*	0					0			3	50%	11%	550	210	510	390	290	G A C A A G A A A G A T G G A A G A C G A A T G T C T T T T C A G A A A								38%	60%	53%
P3H1-NM_001146289.2eU1493-NP_001139761.1aa479SF	0					0			2	4%	0%	300	160	740	160	330	C C C A G C G G G T G G T G A T G G A C G G C G T A A T C T C T G A C C								4%		4%
P4HA1-NM_000917.4eU257-NP_000908.2aa42LL	0					0			2	4%	1%	320	160	640	100	480	A T C C A T A C T G A G A A A G A T C T G G T G A C T T C T C T G A A A								3%	4%	
PABPC1-NM_002568.4eU1451-NP_002559.2aa316PS	0					0			3	2%	0%	350	490	720	250	350	G A T G A T G A A C G T C T C C G G A A A G A G T T T T C T C C A T T T								2%	2%	2%
PACC1-NM_001198862.2eU512-NP_001185791.1aa128SL	0					0			2	24%	4%	470	150	650	350	290	T C A G C A A G G C C T G C C T G A A G A A C G T C T T C T C G G T C C								27%	22%	
PAFAH1B1-NM_000430.4eU556-NP_000421.1aa5Q*	0					0			3	19%	2%	240	290	690	340	420	C T T G A C A T T A C A G C C A A G A T G G T G C T G T C C C A G A G A								18%	17%	20%
PAFAH1B1-XM_017024702.2eU242-5'UTR-99	0					0			2	13%	1%	240	290	690	340	420	C T T G A C A T T A C A G C C A A G A T G G T G C T G T C C C A G A G A								14%	13%	
PAICS-NM_001079524.2eU1149-NP_001072992.1aa366LF	0					0			3	2%	0%	360	190	660	180	460	G A C T G G G G A G T T C A G G A T G T G T G G T C T T C T C T T C G A								2%	2%	2%
PAK1-NM_001128620.2eU1121-NP_001122092.1aa186PL	0					0			2	9%	3%	380	130	550	410	220	A T G A G G A T G A T G A T G A T G A T G A T G C T A C C C C A C C A C								10%		7%
PAN2-NM_001127460.4eU534-NP_001120932.2aa63SF	3	8%	2%	9%	2%	0			3	39%	6%	360	170	800	220	350	A A T C A G T G C A C A T A A T G G A A G G T G T C T A C T C T G A A T		8%	11%	7%				43%	32%	42%
PAPSS1-NM_005443.5eU1305-NP_005434.4aa417AV	0					0			3	5%	1%	280	130	720	220	170	T T A A A G A T A T G A A T G C T G A T G C T G T C T T T G C A T T T C								4%	5%	5%
PARD3B-NM_001302769.2eU4191-3'UTR+48	0					0			3	30%	7%	310	220	800	260	350	C A G C C C G G T C C A G A A A G G A A G G T G T C T A C T C T A C C T								38%	25%	27%
PARD6B-NM_032521.3eU3568-3'UTR+2242	0					0			3	36%	5%	430	220	650	460	410	C A A A T G A C T C C G C T T T G A A G G A T G T T T T C T C T A T A T								30%	40%	38%
PARN-NM_001134477.3eU1910-NP_001127949.1aa559PS	1	2%		2%		0			3	21%	5%	310	450	790	300	360	A A A T T A A A A A G A A T G A A G A A G G A G C T T T C T C C A G C A		2%						27%	20%	17%
PARN-NM_001134477.3eU2529-3'UTR+557	0					0			3	24%	8%	250	130	740	200	300	G C T T G T C C T T C C T T C A G G A A G A T G T C C A C T C A T G C C								16%	25%	31%
PARP14-NM_017554.3eU6487-3'UTR+1036	0					0			2	8%	0%	460	230	650	500	330	T T T G T A T T T T T A G T A G A G A C G A G G T T T C T C C A T G T T									8%	8%
PATJ-NM_001350145.3eU3776-NP_001337074.2aa1222TI	0					0			3	34%	10%	410	310	690	270	230	G T G A T G A A A A T G A A G A A G A A G A T G C C T T T A C C G A C C								38%	22%	42%
PATL1-NM_152716.3eU2845-3'UTR+390	4	8%	4%	10%	0%	1	3%		3	78%	2%	340	270	750	250	270	C C T T C C C T C A T C C T A T T G A A G T T G T T T T T T C A T T G C	10%	9%	10%	3%		3%		80%	76%	77%
PAWR-NM_001354732.2eU1585-3'UTR+389	4	30%	8%	34%	5%	0			2	6%	1%	210	70	650	270	330	A G T T C A A A T A C T T T T G G G A T G T T A C C T T C A C A T G T C	35%	39%	29%	20%				6%		5%
PCGF1-NM_032673.3eU40-NP_116062.2aa5Q*	0					0			2	29%	19%	320	90	890	250	330	C T G G G A T C A G C C T T T A A G A T G G C G T C T C C T C A G G G G								16%		43%
PCLAF-NM_001029989.3eU762-3'UTR+494	4	44%	18%	53%	6%	0			3	21%	16%	350	110	540	350	430	A T G T T G G A A A A C C T T G G A T G G A T A T C T T C T C T T T A A	59%	47%	52%	17%				8%	38%	16%
PCLO-NM_014510.3eU16353-3'UTR+1253	0					0			2	8%	1%	110	510	710	140	480	A G C A C A G A C A C A G C A T A G C A G G A G T C C T C C C C A T T C								9%		7%
PCLO-NM_033026.6eU15737-3'UTR+16	0					0			2	23%	10%	310	190	700	300	390	A C T C A A A C G C A T T G A A G A A A C A T G T C T T C T C A G G G T									30%	16%
PCMTD2-NM_001104925.2eU1387-3'UTR+235	0					0			3	11%	3%	420	330	570	560	410	A G A C C T T C A T T G C A T A G A A G A T T G T T T T C C C A A A G T								8%	14%	10%
PC-NM_000920.4eU3127-NP_000911.2aa1009SL	0					0			3	25%	11%	330	200	750	160	340	G G G A G G A G G T G A C G C C G G A A G A T G T G C T C T C A G C A G								14%	26%	35%
PCNP-NM_001320395.1eU2258-3'UTR+1743	0					0			2	8%	1%	530	260	420	390	300	A G A G A G A A A T G A C T G A G A T G A A T G T C T T T A C T A A A G								8%	8%	
PCNP-NM_001320395.1eU307-NP_001307324.1aa62PL	0					0			2	5%	0%	110	240	660	220	290	G A T C A G C T G A A G A A G A A G C T G C C G A C C T C C C A A C A A								5%	5%	
PCNP-NM_001320399.1eU433-NP_001307328.1aa42PL	0					0			2	6%	1%	110	240	660	220	290	G A T C A G C T G A A G A A G A A G C T G C C G A C C T C C C A A C A A								6%	7%	
PCNX1-NM_001308160.2eU8742-3'UTR+1657	0					0			3	37%	4%	320	390	700	390	430	T G A G G T G A C A G A A G A G A G A A G C T G T T T T T C C T A C C A								35%	41%	36%
PCNX2-NM_001328607.3eU231-5'UTR-18	1	64%		64%		1	33%		2	41%	27%	170	340	320	260	350	C G C G C A G G G T G C A G C G G G G C G C T C A C C A G C C C C G G G	64%				33%			21%		60%
PCYT1A-NM_001312673.2eU727-NP_001299602.1aa202TI	4	32%	8%	36%	2%	0			3	12%	4%	220	170	770	210	480	C T C C A A C A C A G A G G A C A G A A G G T A T C T C C A C A T C A G	38%	35%	34%	21%				13%	15%	7%
PDCD6-NM_001267556.2eU749-3'UTR+104	0					0			3	8%	3%	260	310	590	330	380	G A G C A C A G G T G C A G T T A G A T G C T G T T C T T C C T T T A G								7%	5%	11%
PDCL3-XM_017004890.1eU1746-3'UTR+785	1	14%		14%		0			3	52%	10%	320	150	730	280	410	T T T G T A C T T T T A G C A G A G A C G G A G T T T C A T C A T G T T			14%					62%	43%	50%
PDE12-NM_001322177.2eU3947-3'UTR+2422	0					0			2	11%	1%	400	340	740	330	290	T T A G T A A G T T T G A A A A T G A A G G G G T T T T A T C T G C A T									10%	12%
PDE1C-NM_001191056.3eU2672-3'UTR+298	0					0			2	21%	5%	500	260	740	380	180	A A A C A G A A A C A A A A C A T G A A G G G G C A T C C T C A A A A T									18%	25%
PDE1C-NM_001191057.4eU4120-3'UTR+1976	0					0			2	13%	9%	540	130	660	350	250	G G T A T T G A G A A T T T A A T G A G G C T G T G T T T T C T T G G C								7%		19%
PDE6D-NM_002601.4eU318-NP_002592.1aa50PP	0					0			2	11%	0%	370	230	610	490	370	C C C T G G T G T G G A G C A T G A A G C C C G T G T T C C C A A G A A									11%	11%
PDHA1-NM_000284.4eU2942-3'UTR+1658	0					0			2	6%	3%	360	190	730	260	390	A G C A T A T T C A C A C A T A A A A A G T T G T A T T C T C T T A T A								4%	8%	
PDIK1L-NM_001243532.2eU2389-3'UTR+1087	0					0			3	37%	6%	310	130	730	250	370	C C T T T C A T T T T G G C A C T G A T G C T G T T T T C T C T T G T C								33%	35%	44%
PDK1-NM_001278549.2eU1253-NP_001265478.1aa398LL	0					0			3	47%	5%	250	270	730	280	270	T A C T T C C A A G G A G A C C T G A A G C T G T A T T C C C T A G A G								47%	52%	42%
PDLIM5-NM_001011513.4eU5431-3'UTR+3871	0					0			3	4%	1%	480	280	680	240	430	T T G T C A T C C C C A T T G A A G G G A G A G C C T T C T C A G A C A								3%	3%	6%
PDPR-NM_001322117.1eU1222-NP_001309046.1aa105SL	0					0			3	27%	5%	360	90	550	430	220	T G A C C A T T G A G C A G A A G A T G G C A G A C T A C T C A A A C A								22%	27%	31%
PDS5A-NM_001100399.2eU2984-NP_001093869.1aa830PL	0					0			3	2%	0%	480	250	670	500	230	A A C T G T G G T C T C C A G A T G A A G A G G T T T C C C C T G A A G								2%	2%	2%
PDXDC1-NM_001285444.2eU2429-NP_001272373.1aa752SL	0					0			3	16%	6%	400	130	720	80	310	A A G G G G T C C C A C A C C C A G A A G A T G A C C A C T C A C A G G								18%	20%	9%
PDZD11-NM_001370174.1eU1010-3'UTR+434	0					0			3	12%	1%	310	230	730	250	540	T T T T G A G A C T C C A G T G A G A C G C T G T T T T C A C C C C T T								10%	11%	13%
PEBP1-NM_002567.4eU549-NP_002558.1aa155RC	0					0			3	6%	1%	230	420	640	220	430	C A C C G T G G C A A A T T C A A G G T G G C G T C C T T C C G T A A A								5%	8%	7%
PEG10-NM_001040152.2eU1470-3'UTR+58	0					0			3	23%	5%	540	0	630	300	140	T A A T A A G G T C C C C A C A A G A T G A T G C C T C A T C T C C A C								28%	23%	18%
PEG10-NM_001040152.2eU2519-3'UTR+1107	0					0			2	5%	3%	260	150	620	260	270	C A G T A C C C G C C G C C A C A G C C G C C G C C T C C A C C A C C A								3%		7%
PEG10-NM_001040152.2eU3267-3'UTR+1855	0					2	2%	0%	3	9%	1%	380	390	680	200	530	C A T C T C A G G C C T G A A A A G A A A G T G C T C T C T C A G A T G					2%		2%	8%	10%	9%
PEG10-NM_001040152.2eU3302-3'UTR+1890	0					0			2	2%	0%	250	70	740	140	400	G G A C T C T T G C A T G T T A A G A C T A T G T C T T C A C A T C A T									2%	2%
PERP-NM_022121.5eU874-3'UTR+213	0					0			3	13%	5%	400	340	590	180	540	T T T A T T A T G T T T T G T G A A G T T G T G T C T T T T C A C T A A								8%	18%	12%
PES1-NM_001243225.2eU493-NP_001230154.1aa139SF	0					0			3	11%	4%	290	270	650	210	270	A T G C C C T G C G G G A C C T G G A C G A T G C C C T C T C C A T G T								15%	11%	8%
PEX3-NM_003630.3eU2037-3'UTR+677	0					0			3	49%	18%	370	60	720	250	290	G A G G C T A A A A T T G T G T T G A T G C T G T T T A C T C A C C T A								52%	67%	30%
PFN2-NM_002628.5eU810-3'UTR+348	0					0			2	2%	1%	380	300	550	260	290	C G G T T G T A C A A T C T C T G A T C A G T G T C T T T T C A G C A C									3%	1%
PGD-NM_001304451.2eU848-NP_001291380.1aa265AV	0					0			3	3%	1%	420	210	750	220	330	C C G T C A C C C T C A T T G G A G A A G C T G T C T T T G C T C G G T								2%	2%	4%
PGGT1B-NM_005023.4eU748-NP_005014.2aa241SL	1	3%		3%		0			3	62%	5%	430	270	790	250	250	G T C T G A T G G G T A A A C T A G A A G A A G T T T T T T C A G A A A		3%						60%	68%	58%
PGGT1B-XR_001742121.1eU712-ncr	0					0			3	69%	13%	430	270	790	250	250	G T C T G A T G G G T A A A C T A G A A G A A G T T T T T T C A G A A A								57%	83%	67%
PHAF1-NM_001320540.2eU2574-3'UTR+1225	0					0			3	22%	2%	170	270	670	360	390	C C C G A C T T T T G T G A C T G A C T G G T G T C T T C C C A T T T G								20%	23%	25%
PHC1-NM_004426.3eU2328-NP_004417.2aa654TI	0					0			3	20%	3%	300	0	770	210	480	A C T C T G A G G A G G A G A G A G A T G A T G T C T C C A C A T T G G								17%	24%	19%
PHC3-NM_001308116.2eU1594-NP_001295045.1aa509PS	0					0			3	12%	7%	210	170	730	80	260	A T T G C A A G T C C T C C A C A G A T G T C G A C A T C T C C T C C A								8%	20%	7%
PHF12-NM_001033561.2eU4293-3'UTR+720	0					0			3	11%	8%	480	310	690	340	470	T A C A G T A C T A C A T G T G G T A G A G C G T T T T C T C A A A T T								8%	6%	20%
PHF19-NM_001286840.1eU1470-NP_001273769.1aa468TI	0					0			3	5%	2%	310	100	700	210	230	G C C A G A C C T T C T T C T C A G A T G T C G A C T C C A C C G A C G								2%	5%	7%
PHF6-NM_001015877.2eU1872-3'UTR+572	0					0			2	5%	0%	240	300	580	230	490	T T C A T T T G G T T C C T G T T A A A G T T G T T C T C A C T G A C C								5%	5%	
PHKB-NM_001031835.3eU182-NP_001027005.1aa10SF	0					0			3	15%	6%	350	70	710	120	210	A A A T G G C C T G C T C A C C T G A T G C A G T C G T C T C T C C G T								21%	13%	10%
PHKB-NM_001031835.3eU185-NP_001027005.1aa11PL	0					0			2	15%	1%	290	210	610	350	70	T G G C C T G C T C A C C T G A T G C A G T C G T C T C T C C G T C T T								16%	15%	

PHKB-NM_001031835.3eU2600-NP_001027005.1aa816SF	0					0			3	13%	8%	340	260	740	190	350	G T G C C T T T G G G C A T G A A G A A G A A G T T A T C T C T A A T C								8%	9%	22%
PHLDB2-NM_001134437.2eU589-NP_001127909.1aa111SS	0					0			2	3%	0%	400	260	560	380	150	T T T G G G A A C C A G T G T C A G A A G C A G C C C C T C C T T A G C								3%	3%	
PHOSPHO2-NM_001199290.3eU1101-NP_001186219.1aa247LL	0					0			3	25%	7%	410	80	790	270	280	A G C C T T A G G C C T T C A A A G A A G C T G C C T G C T C A C C G A								28%	18%	30%
PIAS3-NM_006099.3eU289-NP_006090.2aa66PP	0					0			3	18%	5%	290	300	670	360	240	G A T C A A A G A G C T T T A C C G A C G A C G C T T C C C C G G A A								14%	24%	18%
PICALM-NM_007166.4eU1605-NP_009097.2aa434PL	0					0			2	3%	1%	300	250	590	270	100	C T A C T G T A G A T G C T G T T G A T G A T G C C A T T C C A A G C T								2%	4%	
PIGK-NM_005482.3eU2370-3'UTR+1154	0					0			3	35%	6%	380	190	660	250	230	T G C T A T G A C C A A C C C C T G A T G T T G T T T T T T C T T T T C								37%	41%	29%
PIGW-NM_001346754.2eU2466-3'UTR+337	0					0			2	6%	2%	350	330	750	390	450	T T T A T A T T T T T A G T A G A G A T G G G G T T T T G C C A T A T T								5%		7%
PIM3-NM_001001852.4eU2030-3'UTR+873	0					0			2	8%	3%	410	410	520	520	400	G T G T G C A G G C A T C G C A G A T G G G G G T T C T T T C A G T T C								6%		10%
PINK1-NM_032409.3eU1766-NP_115785.1aa559LL	0					0			3	14%	2%	400	320	570	280	270	T G T G T G G A A A C A A A A A T G A A G A T G C T C T T T C T G G C T								15%	11%	15%
PIP4K2C-NM_001146258.2eU300-NP_001139730.1aa68PS	0					0			2	5%	2%	260	340	540	440	240	T C G A T C A A T G A G C T C A G C C A G G T G C C T C C C C C G G T G								3%		6%
PKN3-NM_001317926.2eU2383-NP_001304855.1aa657PL	0					0			3	11%	2%	380	240	740	370	310	T C A T G A T G C A G A T C C A C G A G G A T G T C T T C C C C G A G C								8%	10%	13%
PKP4-NM_001005476.4eU1602-NP_001005476.1aa444RC	0					0			2	3%	1%	360	220	710	150	330	G A G C T C C A A G G A T C G C A G A C G G C G T T G T A T C G C A C A									2%	3%
PKP4-NM_001005476.4eU343-NP_001005476.1aa24TI	0					0			2	4%	1%	340	80	660	420	210	A A C C A C A G A C C C G C C A G G A A G C T G C C T C C A C T G G C C								4%		3%
PLAA-NM_001031689.3eU2032-NP_001026859.1aa612PL	0					0			3	5%	1%	400	240	620	280	250	C T A T T A A C T G T C C T G A A G A T A T T G T C T T T C C T G C A C								4%	5%	4%
PLAAT3-NM_007069.3eU308-5'UTR-117	0					0			3	12%	8%	190	220	680	270	260	T C A C G C C C T G C C C A A C A G A A G C C G C A G T C C C G T G G G								10%	6%	20%
PLAGL2-NM_002657.3eU4939-3'UTR+3231	0					0			2	9%	3%	230	310	690	290	330	T C C C C C C T C C C T T T T T G G T A G C T G T T T T C T C C G T C C									11%	6%
PLAUR-NM_001005376.3eU394-NP_001005376.1aa115LL	0					0			3	5%	1%	310	190	680	260	230	G G C T G T C A C C T A T T C C C G A A G C C G T T A C C T C G A A T G								5%	5%	5%
PLEC-NM_000445.5eU709-NP_000436.2aa220FF	0					0			3	3%	1%	470	150	520	590	80	C A C C T C C A G C T G G A G A G A C G G C C G C C T C T T C A A T G C								3%	3%	4%
PLEKHM2-NM_015164.4eU788-NP_055979.2aa183PP	0					0			3	8%	2%	410	220	600	540	220	G T A C C T G C T G G A C T T T G A A G A C C G C C T T C C C A G C T C								7%	7%	10%
PLEKHM2-XM_005245790.4eU799-XP_005245847.1aa183PP	0					0			3	12%	3%	410	220	600	540	220	G T A C C T G C T G G A C T T T G A A G A C C G C C T T C C C A G C T C								9%	13%	15%
PLEKHM3-NM_001080475.3eU193-5'UTR-266	0					0			2	46%	11%	220	70	710	100	320	T G C T T T G A C A A G T A C T T G C T G T T G T C T T C A C A G A G G								38%	54%	
PLIN2-NM_001122.4eU1788-3'UTR+378	0					0			3	24%	3%	250	280	820	220	540	G C T C T G G T G T G A T C T G A A A A G G C G T C T T C A C T G C T T								23%	22%	28%
PLOD2-NM_000935.3eU1388-NP_000926.2aa398TI	0					0			3	8%	3%	260	180	710	210	360	A C T T T A G T G T G G A T G C A G A T G T T G T T T T G A C A A A T C								8%	6%	11%
PLPBP-NM_001349346.2eU2322-3'UTR+1449	4	24%	9%	28%	5%	0			3	24%	6%	320	220	770	270	430	T T G T T T T T T A T T T T C C A G A T G G C T T T T T C T C T T A T T	34%	24%	26%	13%				30%	19%	24%
PLPP3-NM_003713.5eU1403-NP_003704.3aa286LL	0					0			3	45%	5%	270	250	640	340	350	G A C C T C T T C A A G A C T A A G A C G A C G C T C T C C C T G C C T								40%	44%	50%
PLPP5-NM_001102559.2eU336-NP_001096029.1aa104TI	0					0			2	12%	2%	330	410	670	340	280	G C C T T G C C C T G G C T C T G A A T G G C G T C T T T A C C A A C A								11%	14%	
PLS1-NM_001145319.2eU621-NP_001138791.1aa156FF	0					0			3	31%	3%	460	260	510	350	230	A C C C A T G A A T C C C A A T G A T G A T A G T C T T T T C A A G T C								29%	29%	35%
PLXNA1-NM_032242.4eU9135-3'UTR+3198	0					0			2	7%	4%	470	280	450	470	430	G A A C C C C C T C C C A G A A G A A G A G C G A T C C C T C G G A C G									10%	5%
PLXNB2-NM_001376864.1eU1412-NP_001363793.1aa427SL	4	12%	8%	15%	7%	1	4%		3	7%	2%	370	90	750	260	260	A G G T G T A C C T C A C C C C A G A T G G C A C C T C C T C A G A G T	22%	16%	8%	4%	4%			5%	6%	9%
PMS2CL-NR_002217.1eU1597-ncr	0					0			3	34%	5%	380	250	730	140	460	A T T T T T A T G T T T T G A A A G A C A G A G T C T T C A C T A A C C								39%	29%	33%
PMS2-NM_000535.7eU2687-3'UTR+68	0					3	32%	7%	3	34%	5%	380	250	730	140	460	A T T T T T A T G T T T T G A A A G A C A G A G T C T T C A C T A A C C					26%	31%	40%	36%	29%	38%
PNISR-NM_001322405.2eU271-NP_001309334.1aa24Q*	0					0			2	9%	2%	220	200	650	340	150	T T G A A C C A G C A A C A A T G G A T G C A G T C A T T C C A G C A C								8%	11%	
PNISR-NM_001322410.2eU275-NP_001309339.1aa24Q*	0					0			3	8%	1%	220	200	650	340	150	T T G A A C C A G C A A C A A T G G A T G C A G T C A T T C C A G C A C								8%	8%	7%
PNO1-NM_001329916.2eU1393-3'UTR+680	0					0			3	7%	2%	350	260	690	460	410	T T T G T A T T T T T T G T A G A G A T G G G G T T T C G C C A T G T T								9%	6%	6%
PODXL-NM_001018111.3eU363-NP_001018121.1aa32Q*	0					0			3	8%	3%	200	190	620	190	210	T C G T C G C C G T C G C C G T C G C C G T C G C C C T C C C A G A A T								9%	5%	10%
POGLUT2-NM_001318732.2eU1453-NP_001305661.1aa116TI	0					0			3	32%	2%	300	280	670	300	430	A A C A C C C A G A A C T C A T A G A C G C T G C T T T C A C C A A C T								29%	33%	33%
POGLUT2-NM_001318732.2eU443-5'UTR-665	0					0			3	16%	5%	220	350	680	220	260	G A C C C G G G C T A A A A G C A G A C G T C G T C C T T C C C G C C C								14%	12%	21%
POGLUT3-NM_001363503.2eU839-NP_001350432.1aa274PL	0					0			3	30%	4%	220	300	690	220	440	G C T C T C T G G A T T C A A G A G A T G T T G T C C T T C C A A C G T								35%	26%	30%
POLA1-NM_001330360.2eU5338-3'UTR+887	0					0			2	3%	0%	460	240	510	450	310	G C A A A A T G T A T T T C A G T A T G G C A G T C T T T C C T C T C T								3%	3%	
POLA2-NM_002689.4eU708-NP_002680.2aa126SF	0					0			3	6%	5%	370	310	470	450	400	C T T C A A A G G G T T C T C A G A A G C G A G C T A T C T C T A C C C								2%	5%	12%
POLD3-NM_001363597.2eU1303-NP_001350526.1aa299PL	0					0			3	20%	6%	370	200	760	320	190	A A T C T G A T A G C A G T G A A G A T G A A G T C T T T C C A G A C T								15%	17%	27%
POLDIP2-NM_001290145.2eU198-NP_001277074.1aa40SL	2	1%	0%	1%	0%	0			3	11%	2%	370	160	720	150	380	T C T G T G C G G C G G C C G G A G C T G G A G C C T T C T C G C C A G	1%	1%						14%	9%	10%
POLDIP2-NM_015584.5eU198-NP_056399.1aa40SL	0					0			3	13%	2%	370	160	720	150	380	T C T G T G C G G C G G C C G G A G C T G G A G C C T T C T C G C C A G								12%	15%	11%
POLK-NM_001345921.2eU1391-NP_001332850.1aa435PL	0					0			3	7%	2%	430	260	610	390	200	T G C T A A A A A C A G A A A T T G A T G C T G A T T T T C C A C A T C								7%	6%	9%
POLR1C-NM_001318876.2eU34-NP_001305805.1aa5Q*	0					0			3	20%	9%	270	240	890	180	300	n n C G T G G A G A G A T T G A A G A T G G C G G C T T C T C A G G C G								17%	31%	13%
POLR1D-NM_001206559.2eU487-NP_001193488.1aa11LF	0					0			3	13%	7%	480	0	670	290	170	A T G G G A C C C A T G G G T T G G A T G A A G T G T C C T C T T G C T								6%	14%	19%
POLR1D-NM_152705.3eU207-NP_689918.1aa39LF	0					0			3	19%	3%	480	0	670	290	170	A T G G G A C C C A T G G G T T G G A T G A A G T G T C C T C T T G C T								19%	17%	22%
POLR1F-NM_001002926.2eU1574-3'UTR+536	0					0			2	4%	1%	210	70	630	100	260	A G T T T G C C A A A G A A G T T T C T G A A G T C T T C A C A G C T C									3%	4%
POLR1G-NM_001297590.3eU2179-3'UTR+587	0					0			2	11%	1%	450	260	690	460	410	T A T T T T T T T T T A G T A G A G A T G G G G T T T C A C C A C G T T								12%		11%
POLR2B-NM_000938.3eU281-NP_000929.1aa64PL	0					0			3	5%	1%	390	370	510	490	130	C T G T T C A A A G A A T T G T G G A A G A C G C T C C T C C T A T A G								3%	6%	5%
POLR2B-NM_000938.3eU407-NP_000929.1aa106SL	0					0			3	8%	1%	470	260	600	310	470	C T A C C C A T T G G G A A A G A G A T G G T G C T C C T T C A C C A A								7%	9%	7%
POLR2G-NM_002696.3eU338-NP_002687.1aa90TI	0					0			3	10%	1%	340	130	620	270	380	T T A A A G G G G A G G T C G T G G A T G C T G T T G T C A C T C A G G								10%	8%	10%
POLR2K-NM_005034.4eU289-3'UTR+29	0					0			3	15%	3%	460	230	490	350	490	G A A T G C T G G G A A T T C A G A G G A A T G T C T T C A C T T A T A								17%	12%	15%
POLR3GL-NM_032305.3eU462-NP_115681.1aa114PP	0					0			2	10%	4%	170	340	680	200	380	C G A T T G G A A C C C T G A T T G G C G G C G T C T A C C C C G G G A								13%		7%
POLR3H-NM_001018050.4eU1399-3'UTR+415	0					0			3	16%	2%	310	200	640	390	270	C A G G A G A A A A A C T T T A G A T G G G A G T C T T G T C A T C A A								15%	15%	18%
POMGNT1-NM_001243766.2eU1615-NP_001230695.2aa322LL	0					0			3	9%	2%	370	120	600	290	270	C C C A A T T A C C T G T A C A G G A T G C T G C G C T C T C T G C T T								11%	7%	8%
POP5-NM_015918.4eU705-3'UTR+173	0					0			3	17%	6%	370	310	650	230	190	G A G T A C T C A G T T A A A C T G A A G T T G C C T T T A C C T A G C								10%	19%	21%
PPARD-NM_001171818.2eU2889-3'UTR+1158	0					0			3	34%	5%	310	260	570	380	130	C A G G C C C C A C T C C C C C T G A A G C T G C C C C T C C A G C A C								30%	32%	40%
PPIA-NM_001300981.2eU1625-3'UTR+926	0					0			3	16%	7%	410	190	730	360	330	T T T G T A T T T T T A G T A T A G A T G G G G T T T C A T C A T T T T								9%	22%	16%
PPIA-NM_001300981.2eU404-NP_001287910.1aa8TI	0					0			3	3%	0%	270	240	690	220	420	C A G G G T T T A T G T G T C A G G G T G G T G A C T T C A C A C G C C								2%	3%	2%
PPIA-NM_021130.5eU247-NP_066953.1aa68TI	0					0			3	3%	0%	270	240	690	220	420	C A G G G T T T A T G T G T C A G G G T G G T G A C T T C A C A C G C C								2%	3%	3%
PPID-NM_005038.3eU371-NP_005029.1aa88SL	0					0			3	6%	2%	280	290	730	220	310	A G A A A T T T A T G A T T C A G G G T G G A G A C T T C T C A A A T C								6%	4%	8%
PPIF-NM_005729.4eU295-NP_005720.1aa72PL	0					0			2	3%	1%	200	270	680	260	310	T G G T G C T G G A G C T G A A G G C A G A T G T C G T C C C A A A G A									2%	3%
PPIF-NM_005729.4eU682-NP_005720.1aa201TI	0					0			3	7%	1%	360	150	550	330	440	G T G G G A G G A C A T C C A A G A A G A T T G T C A T C A C A G A C T								8%	5%	7%
PPIG-NM_004792.3eU6203-3'UTR+3729	0					0			3	75%	8%	230	120	790	140	390	T G A A G T T T A G A A A A A A A G C T G T T G T C T T C T C A A T T G								68%	83%	73%
PPIL1-NM_016059.5eU1322-3'UTR+790	0					0			2	5%	0%	260	200	790	80	440	C C A T G A A T C C T C C T T G A G A A G T A G T A C T C T C A G A A A								5%		5%
PPM1D-NM_003620.4eU1774-NP_003611.1aa518PS	0					0			3	5%	2%	250	180	690	80	230	A T G G A C C A A A A A A A T T T G A A G A T G T C A A C T C C T G G C								3%	6%	7%
PPP1CC-NM_002710.4eU1169-3'UTR+13	0					0			3	5%	2%	310	130	750	210	340	A A G C A A G C A A A G A A A T A G A T G T C G T T T T G A C A C T G C								3%	6%	4%
PPP1R12A-NM_001143885.2eU2279-NP_001137357.1aa717TT	0					0			2	3%	1%	200	430	670	290	320	T G A G A A A A A C A A T A G G A A G A A G T C G T T C T A C C C G A A C									2%	4%
PPP1R12B-NM_001197131.1eU7564-3'UTR+6977	4	12%	5%	11%	5%	0			3	63%	13%	290	270	660	490	430	T T T A T A G C T T A A A A C A G A A G T G T G T C T T C C C A G C A C	12%	15%	6%	16%				69%	48%	71%
PPP1R13L-NM_001142502.2eU884-NP_001135974.1aa265Q*	0					0			3	9%	2%	340	150	810	190	300	C T G G A C G T G G C G T A C G A G A A G A A G C C T T C G C A G A C A								11%	9%	7%
PPP1R15B-NM_032833.5eU1647-NP_116222.4aa414PL	0					0			2	6%	0%	220	570	660	220	330	T T G A T T A C T C A T A C C T A G A A G G T G A C C T T C C C A T T T								6%		6%
PPP1R15B-NM_032833.5eU1648-NP_116222.4aa414PP	4	15%	9%	18%	8%	0			3	6%	2%	380	220	570	660	220	T G A T T A C T C A T A C C T A G A A G G T G A C C T T C C C A T T T C	27%	16%	11%	5%				4%	6%	8%
PPP1R9B-NM_032595.5eU1327-NP_115984.3aa388SL	0					0			2	8%	4%	530	150	640	510	260	A G G T A G A T G A A T C C A A G A A G G A G G A C T T C T C G G A G G									11%	6%
PPP2R5C-NM_001161725.2eU2076-3'UTR+223	0					0			3	17%	4%	150	210	760	120	420	C A G A G C C G C G G G T T G A C G A C G G T G T C C T C G C A G T G T								13%	15%	21%
PPP4R1-NM_001042388.3eU2649-NP_001035847.1aa858PP	0					0			3	3%	1%	370	220	480	500	400	C C A G A C T G T C A T T G A G G A T G A C T G C C T T C C C A T G G A								4%	3%	2%
PPP4R1-NM_001042388.3eU89-NP_001035847.1aa5SL	0					0			3	6%	2%	440	200	490	450	330	G A G G G G G C G A C C A C A A G A T G G C G G A C C T C T C G C T G C								5%	6%	8%
PPP4R2-NM_001318025.2eU1057-NP_001304954.1aa277TI	0					0			2	2%	0%	330	70	610	190	150	A T C A A G A A A A A G A A T C T G A T G A T G C C T T A A C T G T G A								2%		2%
PPP4R3A-NM_001284280.2eU1301-NP_001271209.1aa213SF	4	54%	13%	61%	3%	0			3	8%	3%	400	150	690	140	270	A T C G A A C T G C T C T T T T T G A A G T T A T G T T C T C T G A A G	60%	64%	58%	34%				7%	11%	6%
PPP4R3B-NM_001122964.3eU2562-NP_001116436.3aa742PL	0					0			3	4%	1%	370	340	690	350	240	T G G A A A A A C C T A A G C C A G A A G A T G A T T T T C C A G A T A								3%	6%	5%
PPP6R1-NM_014931.4eU172-5'UTR-419	0					0			2	4%	1%	330	240	720	500	180	C C G T T T G G A C C G C G G A G G A G G T C G C C T T C C C C T C C C								4%		3%
PPRC1-NM_001288727.2eU3465-NP_001275656.1aa1146LL	0					0			3	29%	10%	260	330	640	380	280	C A C C C A G C C C G T C T A A G G A A G C T G T C C T T C C T G C C T								17%	36%	34%
PPRC1-NM_001288728.2eU3364-NP_001275657.1aa1026LL	0					0			3	26%	6%	260	330	640	380	280	C A C C C A G C C C G T C T A A G G A A G C T G T C C T T C C T G C C T								19%	30%	29%

PRDX6-NM_004905.3eU505-NP_004896.1aa148LF	0					0			3	28%	2%	420	210	650	320	130	GGTCCTGATAAGAAGCTGAAGCTGTCTATCCTCTAC								28%	26%	30%
PRDX6-NM_004905.3eU686-NP_004896.1aa208TI	0					0			3	4%	1%	260	430	740	300	380	CCAAAAAACTTTTCCCGAAAGGAGTCTTCACCAAAG								3%	4%	5%
PREPL-NM_001042385.2eU829-NP_001035844.1aa265LL	0					2	14%	4%	3	55%	8%	380	180	640	440	310	CCTTTATCTTACAAAAGACAGTCGTTTCCTCACCAT					11%	17%		48%	54%	63%
PREPL-NM_001042386.2eU829-NP_001035845.1aa265LL	0					0			3	60%	8%	380	180	640	440	310	CCTTTATCTTACAAAAGACAGTCGTTTCCTCACCAT								60%	52%	68%
PREX1-NM_020820.4eU6261-3'UTR+1142	4	34%	9%	39%	5%	0			2	17%	4%	280	160	730	140	280	CTGTGATTTATTTTCTTGATGGTAACTTCTCAGAGC	41%	33%	42%	22%				14%		20%
PRKAA1-NM_001355028.2eU3781-3'UTR+1807	0					0			3	32%	3%	310	0	740	190	250	CAATAGATGTAAATGAAGATGTAGTCACCTCAGATA								31%	30%	35%
PRKAG1-NM_001206709.2eU494-NP_001193638.1aa153SL	1	3%		3%		0			3	49%	7%	390	60	700	290	130	CTAATGCCAGCTTGTTTGATGCTGTCTCTTCATTAA		3%						41%	53%	52%
PRKAG2-NM_001040633.2eU1443-NP_001035723.1aa327FF	0					0			3	13%	2%	510	150	340	510	120	AGTGAATATATCTCCAGATGCAAGCCTCTTCGATGC								13%	15%	10%
PRKCA-NM_002737.3eU256-NP_002728.2aa6PL	0					0			3	10%	4%	210	200	650	350	310	GGTTGGGGGGGACCATGGCTGACGTTTCCCCGGGCA								6%	15%	10%
PRKCA-NM_002737.3eU6575-3'UTR+4317	0					0			3	24%	5%	270	210	730	250	330	TTTTTTTTTTTTTGCTGGAATTTGTTTTCTCAGTAC								23%	20%	30%
PRKD1-NM_001330069.2eU736-NP_001316998.1aa134SL	0					0			2	16%	2%	360	190	800	220	150	AGGAAGGCGATCTTATTGAAGTGGTCTTGTCAGCTT								18%	14%	
PRKD3-NM_005813.6eU5500-3'UTR+2019	0					0			3	18%	6%	330	140	770	330	360	TTATTTTCCCTGTGATAGAGGATGACTTCCCAGTTT								24%	13%	18%
PRKDC-NM_001081640.2eU1252-NP_001075109.1aa414LL	0					0			2	3%	0%	360	290	400	250	490	CGACCGTGTTTATCAGATGCCAAGCTTCCTCCAGTC								3%	3%	
PRKDC-NM_001081640.2eU5657-NP_001075109.1aa1883RC	0					0			3	13%	1%	370	150	610	100	560	TACTATAAGATTCTAGACGTGATGTATTCTCGCCTT								14%	13%	12%
PRORP-NM_001256678.2eU520-NP_001243607.1aa48LF	0					0			3	13%	6%	440	130	770	300	450	GGCATCAGGAACCAGCAGAGGTTGTTTTCTCTTAAA								11%	10%	20%
PRPF19-NM_014502.5eU1611-NP_055317.1aa470HY	1	1%		1%		0			3	50%	3%	490	210	630	400	180	TACATCTGCAAACAATGGACGGAGATTCTTCACTTT		1%						48%	49%	53%
PRPF4B-NM_003913.5eU1234-NP_003904.3aa370LF	0					0			2	5%	2%	510	80	510	540	380	CGTGATAAATCAAGAAGAAGCAGGTCTCCACTTTTG								6%	4%	
PRPF8-NM_006445.4eU600-NP_006436.3aa167PP	0					0			2	2%	0%	270	550	680	230	640	TAGGAGGCATTTCAAGAGGATGCGTTTCCCCCCTTT									2%	2%
PRR14-NM_001320464.3eU945-NP_001307393.1aa289SL	0					0			2	8%	1%	460	150	570	340	220	CAATGAAGCTGGAGTTGAAGATCGCCATCTCAGAGG								8%	7%	
PRRC2A-NM_004638.4eU2177-NP_004629.3aa645PS	0					0			3	28%	1%	260	240	720	220	310	GGCTACCCCAAATATCAGAAGTCGTTGCCTCCTCGT								28%	29%	28%
PRRC2A-NM_004638.4eU2180-NP_004629.3aa646RC	0					0			3	39%	1%	320	0	620	260	240	TACCCCAAATATCAGAAGTCGTTGCCTCCTCGTTTC								38%	40%	39%
PRRC2A-NM_004638.4eU2426-NP_004629.3aa728PS	0					0			3	18%	2%	360	160	610	360	260	AACTTTGATCCCCGATGGATGATGATTCCTCCTTAT								16%	17%	20%
PRRC2B-NM_001384818.1eU1926-NP_001371747.1aa565Q*	4	8%	3%	9%	3%	1	2%		3	4%	1%	220	240	810	310	410	TGGTCTCCAAGTGCTGAGAAGGCATCTCCCCAGGAA	12%	8%	7%	4%			2%	4%	5%	3%
PRRC2C-NM_001387844.1eU7029-NP_001374773.1aa2256RC	0					0			3	6%	3%	360	90	760	180	380	TTCAGCCTCACCTTCAAGATGGAGTCTGCACGCAAA								3%	6%	8%
PRSS23-NM_001293179.2eU3581-3'UTR+2311	0					0			3	28%	10%	410	210	640	520	60	GTTGTATTAAGTCAGAGGAAGATGCCTCTCCATTTT								17%	36%	31%
PRSS23-NM_001293179.2eU633-NP_001280108.1aa172TI	0					0			3	5%	0%	270	160	550	330	510	GCACCCTGGTGGCAGAGAAGCATGTCCTCACAGCTG								5%	4%	5%
PRXL2C-NM_153698.2eU2565-3'UTR+1848	0					0			3	14%	7%	330	280	690	460	330	TTCTATTTTTTAGTAAAGATGGAGTTTCTCCATGTC								9%	10%	22%
PSEN2-NM_000447.3eU111-5'UTR-274	0					0			2	15%	3%	420	160	440	500	360	GAAGGAACCTGAGACAGAAGCTAGTCCCCCCTCTGA								13%	17%	
PSMA1-NM_002786.4eU1024-3'UTR+145	0					0			3	3%	1%	430	470	450	460	330	TTTAGGAATCAGTCCAGATGTGAGTTTTTTCCAAGC								4%	3%	3%
PSMA1-NM_002786.4eU779-NP_002777.1aa231PL	0					0			3	10%	1%	450	130	610	390	130	TTACAATCTATGATGATGATGATGTGTCTCCATTCC								11%	11%	9%
PSMA2-NM_002787.5eU1322-3'UTR+601	0					0			2	20%	4%	220	140	690	170	360	ATGGTTTGCTGGTTCCTGTTGGTGTCTCCACAAGTA								17%		23%
PSMA5-NM_002790.4eU107-5'UTR-13	0					0			3	8%	0%	370	100	620	260	240	CTCAGAATCCCCGCGTAGCCGCTGCCTCCTCCTACC								8%	9%	8%
PSMB10-NM_002801.4eU184-NP_002792.1aa28PL	0					0			2	11%	4%	150	360	700	220	400	AAAGAAATGCATCATTGGAACGCGTCCTCCCGGGGC									13%	8%
PSMB7-NM_002799.4eU368-NP_002790.1aa117PP	0					0			3	2%	0%	300	140	710	330	330	GCTCCACTCCCTCTCCACTGGCCGTCTTCCCAGAGT								2%	1%	1%
PSMD10-NM_002814.4eU1314-3'UTR+611	0					0			3	9%	5%	400	140	550	260	580	TATGTATTTGTTTTTGATGGGTTGTTTACTCTGAAA								3%	11%	11%
PSMD10-NM_002814.4eU810-3'UTR+107	1	2%		2%		0			3	27%	4%	410	130	780	250	310	AGACATCATCTATGAATGATGAAGTTTTCTCACCTT	2%							27%	31%	23%
PSMD11-NM_001270482.2eU3663-3'UTR+2380	0					0			2	14%	2%	320	340	740	250	540	TAGTTTTGGAGTAGAGGGGAGATGTTTTCTCAAACT									15%	12%
PSMD13-NM_002817.4eU180-NP_002808.3aa36Q*	0					0			3	5%	2%	340	80	660	190	210	GAGGAGCTCTACACGAAGAAGTTGTGGCATCAGCTG								7%	3%	4%
PSME3IP1-NM_001354078.1eU2313-3'UTR+1447	1	5%		5%		0			3	40%	10%	250	230	800	240	300	TCTGGTGCCCATTGGCAGATGGCGACTTCCCTGCAC			5%					36%	52%	32%
PSME3-NM_001267045.2eU2747-3'UTR+1420	0					0			3	3%	1%	310	140	790	400	270	TGTAGAGAGGGAGTCAGGATGCTGTCTTCCCACGAA								2%	3%	5%
PSMG4-NM_001128591.2eU435-NP_001122063.1aa120PL	0					0			3	57%	5%	470	380	470	690	280	ACAGGATCAAGGAAGAGATGGAGGCTTTCCCCGAAA								56%	52%	62%
PSPH-NM_001370503.1eU603-5'UTR-45	0					0			3	9%	4%	420	340	580	330	430	ATCTAGGGAAGGAACAGAAGAGTGTCGTCTCCTAGA								7%	7%	13%
PTBP2-NM_001300985.2eU254-NP_001287914.1aa58SF	0					0			3	32%	3%	510	260	510	390	310	AAGGAGAAGATAAAATGGATGGTGCTCCTTCTCGTG								35%	28%	32%
PTCH1-NM_000264.5eU7294-3'UTR+2045	0					0			2	19%	7%	350	220	760	240	140	GGATGATGCAGAATTCTGAAGTCGACTTCCCTCTGG								24%		14%
PTEN-NM_000314.8eU2338-3'UTR+281	4	48%	9%	52%	4%	0			3	30%	8%	210	290	670	400	400	CAATTAGGATTAATAAAGATGGCACTTTCCCGTTTT	56%	50%	48%	36%				35%	33%	21%
PTGES2-NM_001256335.2eU1183-3'UTR+334	3	3%	0%	3%	0%	0			3	3%	1%	380	220	670	360	250	CCTCGACAGGACAGCAGGACGGTTTGTTTTCAGTGG	3%	3%	4%					3%	3%	4%
PTK7-NM_001270398.2eU3045-NP_001257327.1aa992SF	0					0			2	5%	2%	390	210	760	310	320	CCCCCGAGGCCATCCTGGAGGGTGACTTCTCTACCA								6%		4%
PTPN11-NM_001330437.2eU1675-NP_001317366.1aa504Q*	0					0			3	7%	1%	380	160	730	130	360	GTTCCCAAAACCATCCAGATGGTGCGGTCTCAGAGG								8%	6%	7%
PTPN1-NM_001278618.2eU940-NP_001265547.1aa223HY	1	2%		2%		0			3	8%	3%	350	370	760	440	280	TCCGTGCAGGATCAGTGGAAGGAGCTTTCCCACGAG			2%					7%	5%	11%
PTPN1-NM_002827.4eU237-NP_002818.1aa31PL	0					0			3	3%	1%	250	270	640	200	360	AGGATATCCGACATGAAGCCAGTGACTTCCCATGTA								3%	2%	4%
PTPRJ-NM_002843.4eU2312-NP_002834.3aa653PL	0					0			2	11%	2%	410	280	590	400	0	TAAGTTGGCAGAACTTTGATGACGCCTCTCCCACGT									10%	13%
PTPRJ-NM_002843.4eU5010-3'UTR+642	0					0			2	9%	3%	400	180	410	540	390	CAGTAGCTTAGCATCAGAGGTTTGCTTCCTCAGTAA								8%	11%	
PTPRM-NM_001105244.2eU4989-3'UTR+88	0					0			3	23%	9%	310	120	810	140	340	GGTATTTGTGCAAAAGAGATGAAGACTTCTCAATAT								17%	19%	33%
PTTG1IP-NM_001286822.2eU455-NP_001273751.1aa127LL	0					0			3	2%	1%	330	280	500	210	610	CGGAGAGCTGCCCCTGAAGGACAGTCCTCTCGTCTT								2%	3%	2%
PTTG1-NM_001282382.1eU738-NP_001269311.1aa112PL	1	1%		1%		0			3	55%	2%	410	130	640	330	140	GCTCTGTTCCTGCCTCAGATGATGCCTATCCAGAAA			1%					57%	52%	56%
PTTG1-NM_001282382.1eU917-NP_001269311.1aa172PS	0					0			3	2%	1%	290	350	690	130	350	GGCCCCCCTTCACCTGTGAAGATGCCCTCTCCACCA								2%	1%	3%
PTTG1-NM_004219.4eU383-NP_004210.1aa112PL	0					0			3	57%	14%	410	130	640	330	140	GCTCTGTTCCTGCCTCAGATGATGCCTATCCAGAAA								43%	57%	71%
PUF60-NM_001136033.3eU891-NP_001129505.1aa243SF	0					0			3	3%	1%	470	210	640	330	210	AGGCCCAGTCGTCCCAAGATGCTGTGTCTTCCATGA								5%	2%	3%
PURA-NM_005859.5eU1995-3'UTR+953	0					0			3	15%	9%	230	300	790	170	410	CTGTTTGCTTTGACTGAGTAGGCGTTTTGTCATTAT								14%	7%	25%
PURA-NM_005859.5eU3533-3'UTR+2491	0					0			3	28%	7%	320	370	430	400	440	AGTGTCCTTTCTTTGAGATGCGAGTTCTTTCATGAG								33%	20%	32%
PXDC1-NM_183373.4eU556-NP_899229.2aa125TI	0					0			3	47%	4%	230	300	660	160	390	GTAAATATTCTAGATCGGAAGTTGTGCTCACCTTCT								43%	47%	50%
PXDC1-XM_011514393.3eU954-XP_011512695.1aa64TI	0					0			3	45%	5%	230	300	660	160	390	GTAAATATTCTAGATCGGAAGTTGTGCTCACCTTCT								40%	44%	50%
PXDC1-XR_001743252.2eU1254-ncr	0					0			3	41%	21%	230	300	660	160	390	TCCATTATTCTAGATCGGAAGTTGTGCTCACCTTCT								18%	56%	50%
PYGL-NM_001163940.2eU1173-NP_001157412.1aa365R*	0					0			3	11%	1%	300	240	620	310	450	CCCGTGGACCTGGTGGAGAAGCTGCTCCCTCGACAT								10%	11%	12%
PYGL-NM_001163940.2eU1620-NP_001157412.1aa514Q*	0					0			3	2%	1%	270	130	740	210	350	GTGAAGCAGGAGAATAAGCTGAAGTTTTCTCAGTTC								1%	2%	2%
QDPR-NM_000320.3eU1190-3'UTR+419	0					0			3	78%	14%	210	130	780	210	390	GTCATGTTTATGTCATAGACGTAGTTTTCGCATCCT								70%	93%	70%
QSOX1-NM_001004128.3eU2293-3'UTR+439	0					0			3	9%	2%	330	170	470	550	430	CTTAGCTGCAGGAGAAGATGGCTGCTTTCACTTCCC								9%	7%	11%
QSOX1-NM_002826.5eU3026-3'UTR+743	0					0			3	78%	3%	330	170	470	550	430	CTTAGCTGCAGGAGAAGATGGCTGCTTTCACTTCCC								81%	77%	77%
R3HCC1L-NM_001256619.2eU120-5'UTR-199	0					0			3	24%	8%	210	290	750	310	410	CATTGCTGTTTTCCTGAGACGGAGTTTTGCCATGTT								20%	18%	33%
R3HDM2-NM_001330121.2eU3916-3'UTR+454	0					0			3	25%	2%	260	220	690	380	410	TTTGTATTTTTAGTAGAGACGGAGTTTCACCATGTT								22%	27%	25%
RAB11FIP5-NM_001371272.1eU5096-3'UTR+950	0					0			3	4%	0%	310	220	690	160	210	GGGGTCAAGAGCCCCTGGATGTCGACCTCTCCCGCT								4%	4%	3%
RAB1A-NM_004161.5eU84-5'UTR-109	0					0			3	4%	1%	280	290	310	510	450	GGACGGAGTAGGGGAAGACGTTTGCTCTCCCGGAAC								4%	5%	4%
RAB21-NM_014999.4eU1374-3'UTR+429	0					0			2	2%	0%	350	260	690	460	410	TTTGTATTTTTAGTAGAGACGGGGTTTCACCAGGTT									2%	2%
RAB22A-NM_020673.3eU7291-3'UTR+6444	0					0			2	20%	7%	370	150	760	180	180	TTACCGTCTCTGTGATTGAAGCAGACTTCTCGCTTT								16%	25%	
RAB23-NM_001278666.2eU1127-3'UTR+235	0					0			3	7%	3%	450	190	540	470	140	TAATTTTTTTTACATTAGACGAAGCTTCTCCTGTTT								10%	8%	4%
RAB2B-NM_001163380.2eU1780-3'UTR+1100	0					0			2	14%	1%	440	150	590	610	310	CCTAAGAATTCTTGTAGAAGGCAGTTTCCTCTACTT									15%	13%
RAB3GAP1-NM_001172435.2eU2961-NP_001165906.1aa982SL	0					0			3	12%	3%	340	300	700	190	180	AAGAGGACTTTAGACTTGCAGGTGCCTTTTCATCAG								12%	9%	16%
RAB3GAP1-NM_012233.3eU2940-NP_036365.1aa975SL	0					0			2	14%	2%	340	300	700	190	180	AAGAGGACTTTAGACTTGCAGGTGCCTTTTCATCAG								13%		16%
RAB3GAP2-NM_012414.4eU2242-NP_036546.2aa709PL	0					0			3	23%	11%	330	230	700	280	370	GATTTTCTGATGATAAAGATGGTGTGTTGCCTGTAA								14%	19%	35%
RAB3GAP2-NM_012414.4eU5616-3'UTR+1318	0					0			3	8%	3%	570	160	480	570	270	AAGTCATTGTGATACAGAAGTTTGTTTCTTCACTTC								5%	9%	11%
RAB3IP-NM_001024647.3eU712-NP_001019818.1aa125PL	4	43%	12%	49%	3%	0			3	9%	1%	280	280	680	400	170	TAGACAAAATCTACCAGGAAGATATCTTTCCATGTT	48%	52%	47%	25%				7%	9%	10%
RABEP1-NM_001083585.3eU168-5'UTR-37	0					0			3	12%	4%	400	130	610	590	160	AGCCCGCGGGAGCCCAGGACGCCGCTTCCCCGCCCA								12%	8%	15%
RABEP1-NM_001083585.3eU5240-3'UTR+2547	1	8%		8%		0			3	59%	4%	400	220	800	210	350	CATTTGTTAACTGTACTGAAGGTGTGTCCTCAAGAA		8%						58%	55%	63%

RABGAP1-NM_012197.4eU1927-NP_036329.3aa602Q*	0					0			3	3%	1%	260	280	730	430	290	TACCGCATTCTTATCACAAAGGAGTCTCCCCAGGAC								2%	2%	4%
RABIF-NM_002871.5eU1793-3'UTR+1370	3	19%	2%	19%	2%	0			2	16%	10%	360	260	480	540	320	CCTACTCCCCTGGAAAGATGGCTTCCTTCTCCAATT	19%	18%	21%					24%	9%	
RABL2B-NM_001003789.3eU1588-3'UTR+705	0					0			2	20%	6%	350	260	690	460	410	TTTGTATTTTTAGTAGAGACGGGGTTTCACCATGTT								16%		24%
RAC1-NM_006908.5eU897-3'UTR+109	0					0			3	5%	2%	310	240	690	190	350	CAAAAAAAAAAAACAACGGTGGAGCCTTCGCACTCA								3%	5%	8%
RACGAP1-NM_001126103.3eU995-NP_001119575.1aa235TI	0					0			3	3%	1%	380	130	670	250	320	ATGATGGCGGGCCCATCGAAGCTGTGTCCACTATTG								2%	5%	3%
RAD1-NM_002853.4eU168-NP_002844.1aa4LL	0					0			3	17%	7%	500	110	520	320	130	GGGCCGAATGCGCAGTGGACGATGCCCCTTCTGACC								16%	12%	25%
RAD21-NM_006265.3eU3482-3'UTR+1388	0					0			3	18%	3%	210	470	690	350	390	ATGATGTGCCTGTAAACCAAGGAGTTTTCCCCGTTT								16%	21%	18%
RAD23B-NM_001244713.1eU198-NP_001231642.1aa19PL	0					0			3	4%	0%	360	320	620	490	220	TTGAATCTGAAAAGGGGAAAGATGCCTTTCCAGTAG								3%	4%	4%
RAD23B-NM_002874.5eU470-NP_002865.1aa40PL	0					0			3	2%	1%	360	320	620	490	220	TTGAATCTGAAAAGGGGAAAGATGCCTTTCCAGTAG								2%	2%	3%
RAD51AP1-NM_001130862.2eU718-NP_001124334.1aa223SF	0					0			3	2%	1%	350	120	720	180	180	GTGAGAGTGAGGATAATGACGAAGACTTCTCTATGA								2%	2%	3%
RAD51C-NM_058216.3eU814-NP_478123.1aa258RC	0					0			3	4%	1%	400	220	480	210	460	TTTCGTCATGACCTAGATGACCTGTCTCTTCGTACT								4%	6%	4%
RAD9A-NM_004584.3eU300-NP_004575.1aa81LL	0					0			3	12%	1%	360	200	670	280	110	CTGCGCTGTAAGATCCTGATGAAGTCTTTCCTGTCT								13%	13%	11%
RAI1-NM_030665.4eU4310-NP_109590.3aa1276PS	0					0			3	8%	1%	540	170	820	270	390	TCCCCCACCCTCTTCAAGAGGATGTCTTCTCCCAAG								8%	7%	8%
RALBP1-NM_006788.4eU2314-3'UTR+112	0					0			3	12%	3%	310	280	780	220	470	CTCGCTGTACATTCTGTAAAGGTGTCTTCTCTTCTC								13%	9%	14%
RALY-NM_007367.4eU1316-3'UTR+130	0					0			3	2%	0%	420	270	740	220	260	CCAGAGGAAAGCTGGCAGCAGGCGCCTCCTCCCCCA								2%	1%	1%
RANBP2-NM_006267.5eU3259-NP_006258.3aa1045TM	0					0			3	20%	1%	210	160	720	180	370	CAAATTTCAAATCAAATGATGGTGACTTCACGTTTT								20%	19%	21%
RAP1A-NM_001010935.3eU935-3'UTR+113	0					0			3	4%	1%	540	310	540	550	60	TCAAAAATAAAAAATCTGAAGAGGCTTCTCCTGTTT								4%	3%	4%
RAPGEF1-NM_001304275.2eU1186-NP_001291204.1aa314PS	0					0			2	5%	0%	200	330	650	190	340	CCACCCAAGAAAAGACAGTCGGCGCCGTCCCCTACC								5%	5%	
RAPGEF1-NM_001304275.2eU833-NP_001291204.1aa196SL	0					0			2	5%	0%	340	170	770	160	250	CTGACCAAGTGATGCTGGAAGGCGTGAACTCAGAAG								5%		5%
RASAL2-NM_004841.5eU4307-3'UTR+742	0					0			3	15%	3%	350	200	660	390	370	TCCTCTGTCTACAGCAGATGGGAGTCTTCTCTTTTA								16%	11%	17%
RASSF1-NM_001206957.2eU1237-3'UTR+290	1	1%		1%		3	14%	2%	3	32%	5%	320	170	650	370	210	CAGCAGGAGTGGTGCAGACAGAAGTCTCCTCAATTT		1%			12%	16%	13%	30%	28%	38%
RASSF8-NM_001164746.2eU5141-3'UTR+3340	0					0			3	12%	4%	290	70	740	220	360	GCCTTAATTTATATAACGATGCTGTGTTCACATCCC								8%	15%	14%
RBBP8-NM_002894.3eU1750-NP_002885.1aa472PL	0					0			2	2%	0%	410	320	620	360	380	AAGCTTCTTTTGATAAAGAAAATGCTTTCCCTTTTC									2%	2%
RBBP8-NM_002894.3eU817-NP_002885.1aa161PL	0					0			3	5%	0%	310	340	680	410	170	CTGAGCTTGAATGTGAGGAAGACGTTATTCCAGATT								4%	5%	5%
RBM15B-NM_013286.5eU6487-3'UTR+3699	0					0			3	28%	6%	310	290	860	220	350	TCTCCTTGCCTCTCCTGGAAGGTGTCTTCTCTATGG								30%	22%	33%
RBM17-NM_001145547.2eU757-NP_001139019.1aa38TI	0					0			3	5%	1%	460	220	480	530	440	CTCAGCTTCAGGTGAAGAAGGCAGCTCTCACTCAGG								5%	6%	4%
RBM19-NM_001146698.2eU2350-NP_001140170.1aa751SL	0					0			3	10%	5%	590	220	630	430	190	CAACAGAAGAGAAGCTGAAGGAAGTGTTTTCAAAAG								6%	16%	8%
RBM22-NM_018047.3eU90-NP_060517.1aa5LL	0					0			3	28%	5%	250	210	770	120	370	CCTCCGGACGCTCTCAAGATGGCGACCTCTCTGGGT								26%	24%	34%
RBM22-XR_001742145.2eU117-ncr	0					0			3	31%	6%	250	210	770	120	370	CCTCCGGACGCTCTCAAGATGGCGACCTCTCTGGGT								24%	37%	30%
RBM26-XM_005266491.5eU6327-3'UTR+3022	0					0			2	21%	6%	360	140	710	360	270	CTTTTGTGTAATTGAATGAAGCAGTTTCCTCTGCAT								16%		25%
RBM33-NM_053043.3eU2176-NP_444271.2aa654Q*	0					0			3	20%	7%	210	130	690	190	200	GTCCCGCCCCCTCCTTTGATGCCGATGTCTCAGCCA								17%	14%	28%
RBM33-NM_053043.3eU4109-3'UTR+380	0					0			3	8%	3%	210	210	700	210	320	ATTAACGTCACAAGTTCTAAGTAGTTTTCACAGCAA								8%	5%	11%
RBM33-NM_053043.3eU7160-3'UTR+3431	0					0			2	15%	6%	210	150	800	200	350	GAGGCCTGGGGATTGGGGAAGCTGTCATCTCAGTGT								10%	19%	
RBM39-NM_001242599.2eU2397-3'UTR+537	4	49%	16%	57%	4%	3	9%	1%	3	23%	4%	300	270	720	180	250	TCAAAATTATATTTCTTGATGGTATCTTTTCAATTA	53%	61%	56%	26%	8%	10%	8%	28%	22%	21%
RBM7-NM_001286045.2eU2571-3'UTR+1761	0					0			2	18%	7%	500	260	690	460	410	TTTGTATTTTTAGTAGAGATGGGGTTTCACCACATT									23%	13%
RBM8A-NM_005105.5eU136-NP_005096.1aa26HH	0					0			3	2%	0%	420	290	470	360	430	CATGGATGAGGATGGGGACGAGAGCATTCACAAACT								2%	3%	2%
RBMS2-NM_002898.4eU5650-3'UTR+4222	0					0			2	10%	4%	400	330	750	390	450	TTTTTATTTTTAGTAGAGATGGGGTTTTACCACGTT								7%		12%
RC3H1-NM_001300850.1eU7530-3'UTR+4038	0					0			2	17%	11%	470	260	690	230	180	TAGCCAATAACTTCTGTGAAGTTGCCTTTTCTAATG								25%		9%
RC3H2-NM_001100588.3eU5301-3'UTR+1408	0					0			3	11%	1%	360	140	710	210	370	TTTTAATTAACTTCTGTGAAGTTGTTTACTCTGAAA								13%	11%	10%
RC3H2-NM_001100588.3eU8497-3'UTR+4604	0					0			3	17%	3%	150	210	690	220	500	ATGTAAATGAAATTAAAGATGGTGTACACCCATCAT								20%	16%	15%
RCAN1-NM_001285389.2eU344-NP_001272318.1aa99PL	0					0			3	5%	3%	360	150	590	380	160	TGGGATGGAAACAAGTGGAAGATGCGACCCCAGTCA								3%	4%	9%
RCC1L-NM_001363447.2eU1429-NP_001350376.1aa244PL	0					0			2	2%	1%	360	350	600	270	280	AAGGTCCAAACCTAGTGGAAAGTGCCGTCCCTGAAA									2%	1%
RCC1-NM_001048194.4eU1286-NP_001041659.1aa398FF	0					0			3	9%	1%	560	170	540	390	310	GTATGCTGTGACCAAGGATGGTCGTGTTTTCGCCTG								10%	8%	9%
RCC2-NM_001136204.3eU1080-NP_001129676.1aa353SF	0					0			3	7%	3%	350	300	650	390	410	TGGTCCTGGACTCCCAGAAGCGAGTCTTCTCCTGGG								7%	10%	5%
RCC2-NM_001136204.3eU1479-NP_001129676.1aa486SL	4	31%	10%	36%	3%	3	5%	1%	3	21%	1%	280	220	780	290	350	AGGAGGTAAAGACTCTGGATGGCATTTTCTCAGAGC	39%	32%	36%	17%	5%	6%	3%	20%	21%	22%
RCL1-NM_001286699.2eU409-NP_001273628.1aa12SL	0					0			3	40%	6%	360	250	820	290	350	CTCCCGGAGGAGGAGGCGAAGTGGTTTTCTCATGTC								46%	41%	34%
RCL1-NM_005772.5eU657-NP_005763.3aa170SL	0					0			3	44%	8%	360	250	820	290	350	CTCCCGGAGGAGGAGGCGAAGTGGTTTTCTCATGTC								38%	41%	52%
RCN1-NM_002901.4eU1214-3'UTR+95	0					0			3	12%	2%	340	220	670	140	390	CAATTGTCTAATTTACAGCAGTTGTGATCCCACAAA								14%	10%	11%
RDH13-XM_011526408.3eU2086-3'UTR+1157	0					0			2	28%	1%	330	280	730	420	410	TTTGTATTTTTAGTAGAGATGGCGTTTCTCCATGTT								29%		27%
RDM1-NM_001034836.2eU305-NP_001030008.1aa87Q*	0					0			2	31%	3%	430	270	690	420	100	CAAAAGGCATGCGACCGGAAGCAGCTTTTTCAGAAA								30%	33%	
RELB-NM_006509.4eU2236-3'UTR+380	0					0			2	6%	0%	250	320	580	240	290	GGGGGGTAGGTTGGTTGTTCAGAGTCTTCCCAATAA								6%		6%
REXO1-NM_020695.4eU562-NP_065746.3aa146PL	0					0			2	8%	2%	470	140	730	420	240	CCACTGTGGGCCCGGACGAGGATGCCTTCCCACTGG								6%		9%
RFC2-NM_001278791.2eU1116-NP_001265720.1aa232SF	0					0			2	2%	0%	430	170	680	160	250	CTCACATGAAAATAGCGGAAGGAGTGAACTCTCTTT									2%	2%
RFC3-NM_002915.4eU1998-3'UTR+897	0					0			3	21%	1%	250	120	740	290	290	ATCCCAATCAAGGTATAGATGCCGTCACCCCAAAAA								20%	21%	22%
RFC3-NM_002915.4eU918-NP_002906.1aa296LL	0					0			2	3%	1%	630	400	590	230	390	TCCTCCTGAGATAATAATGAAGGGCCTTCTCTCAGA									4%	3%
RFC4-NM_002916.5eU631-NP_002907.1aa143PS	0					0			3	7%	4%	340	180	710	330	250	GGAAGTCGCTCAGATGGGAAGCCGTGTCCGCCTTTT								10%	3%	9%
RFTN1-NM_015150.2eU1348-NP_055965.1aa361TI	0					0			3	42%	6%	310	140	720	320	340	ATGGAGTATTCATCTTTGAAGCTGTTTCCACAGAAG								48%	43%	36%
RFXANK-NM_001278727.2eU623-NP_001265656.1aa59SS	0					0			2	11%	7%	540	240	370	490	310	TGTGAATCCTGAACCGGATGCCAGTGTTTCCTCTCC								6%	16%	
RFXANK-NM_001278728.2eU351-NP_001265657.1aa59SS	0					0			3	23%	6%	540	240	370	490	310	TGTGAATCCTGAACCGGATGCCAGTGTTTCCTCTCC								24%	29%	18%
RFXANK-NM_001370235.1eU602-NP_001357164.1aa59SS	0					0			2	20%	6%	540	240	370	490	310	TGTGAATCCTGAACCGGATGCCAGTGTTTCCTCTCC								16%		24%
RGPD6-NM_001123363.4eU6400-3'UTR+904	0					0			2	4%	1%	280	290	680	280	250	TTGAGTTGAGGTGATTTCATGGTGTCTTTCCAGGCT								3%	4%	
RHBDD2-NM_001040456.3eU414-NP_001035546.1aa122SL	0					0			2	4%	1%	580	0	650	340	170	TCATCTTCCTGTCATTCGAGGCTGTGTCATCACTGT								3%	5%	
RHNO1-NM_001252499.3eU1131-3'UTR+270	0					0			3	19%	5%	420	230	650	500	330	TTTGTATTTTTAGTAGAGATGTGGTTTCTCCATGTT								13%	20%	24%
RHNO1-NM_001252499.3eU178-NP_001239428.1aa12Q*	0					0			3	14%	4%	340	140	480	420	260	CCTCCCAGAAAAAAACGCCGCCAGCCTTCCCAGAAA								10%	14%	17%
RHOT1-NM_001033566.3eU334-NP_001028738.1aa48PL	0					0			3	10%	5%	250	70	620	210	310	AAGAAATCACCATTCCAGCTGATGTCACCCCAGAGA								5%	9%	15%
RHOT2-NM_001352275.2eU1045-NP_001339204.1aa326SL	0					0			2	7%	1%	350	210	730	130	370	AGCACGACCAGGACCGCGACGGCGCCCTCTCGCCCG								6%		8%
RHOT2-NM_001352275.2eU228-NP_001339204.1aa54HY	0					0			3	15%	4%	340	290	720	230	470	GCGGACGTCACCCCGGAGAAGGTGCCCACCCACATC								15%	11%	20%
RHPN1-NM_052924.3eU661-NP_443156.2aa183LL	0					0			2	23%	10%	500	190	490	500	440	CCAGCTGTGCTTCCTGGATGCGCGCTTCCTCACCCC								16%	30%	
RICTOR-NM_001285439.2eU4797-NP_001272368.1aa1592SF	0					0			3	7%	3%	440	280	680	300	380	CTATCCATAATCCTTTAGAAGTGGTTCCCTCTAAGT								4%	10%	8%
RIN2-NM_001242581.2eU2183-NP_001229510.1aa716PS	0					0			3	9%	4%	250	250	770	140	350	AAGTTCATGACCATGCAGAAGATGTATTCGCCGGAA								12%	11%	5%
RIN3-NM_001319987.2eU1075-NP_001306916.1aa317SS	0					0			3	14%	4%	400	250	640	370	120	TCCCACTGCCCCTCCCAGACGCCGCGTTTCCGAGAG								10%	18%	14%
RIOK3-NM_001348193.2eU1646-NP_001335122.1aa498PL	0					0			2	2%	0%	300	210	570	290	150	CTTCATTTTTGAAAGATGATGGAGACCCACCACTAC								1%		2%
RIOK3-NM_003831.5eU323-NP_003822.2aa57PL	0					0			3	5%	1%	330	260	620	390	250	TGCAGTTAGAAGAAGAAGCTGCCGTTTTCCTGAAG								5%	4%	6%
RIOX1-NM_024644.5eU1329-NP_078920.2aa415SF	0					0			3	11%	4%	350	140	690	200	320	ACCAAGCTGAATGCCAGGATGGAGTCCACTCTCTGC								9%	15%	9%
RMND1-NM_001271937.2eU524-NP_001258866.1aa136SF	0					0			3	6%	1%	440	320	500	510	340	ATGATGCCATTCTAGAGAAGTTTGCTTTCTCCAATG								7%	5%	7%
RND3-NM_001254738.1eU2430-3'UTR+1417	0					0			3	10%	2%	230	370	660	220	340	TTCGTTTTTGAAGTGTTGAAGCTGTGCTCCCCTTAA								9%	12%	10%
RND3-NM_005168.5eU2309-3'UTR+1417	0					0			3	10%	1%	230	370	660	220	340	TTCGTTTTTGAAGTGTTGAAGCTGTGCTCCCCTTAA								8%	9%	11%
RNF114-NM_018683.4eU2214-3'UTR+1511	0					0			3	4%	1%	300	190	690	230	300	GTTCACTGTAATTTCTGGAAGAAGTTCACTCAGACC								4%	3%	5%
RNF114-NM_018683.4eU906-3'UTR+203	0					0			3	3%	1%	260	360	700	360	310	CCCTTTGGGCTCTTGCCAAAGCTGTCTTCCCCTACT								3%	2%	3%
RNF115-NM_014455.4eU475-NP_055270.1aa85Q*	0					0			2	5%	2%	380	240	670	250	310	TGGGGCCATTTGGATCACACGATGTTTTTCAAGAT								3%	6%	
RNF121-NM_001300926.2eU1181-3'UTR+489	0					0			3	7%	1%	310	280	770	270	260	CATGACATTTTTGGTTTAAAGGAGCCTTCTCATCTC								7%	6%	7%
RNF146-NM_001242844.2eU966-NP_001229773.1aa129SF	0					0			3	23%	7%	470	420	640	380	160	GCACTAGTAGAGAGCTGGAAGATGCTTTTTCCAAAG								18%	31%	20%
RNF167-NM_001320356.2eU1524-3'UTR+55	0					0			3	3%	0%	310	180	620	400	250	GTGACCTATTTGCACAGACCGTCGTCTTCCCTCCAG								3%	3%	4%

RNF167-NM_001320360.2eU541-5'UTR-23	1	1%		1%		0			3	7%	3%	410	120	590	340	350	GCTCCTTGCTACCAGGACGCGCGGCCTCCTCAGCCT			1%					8%	10%	4%
RNF20-NM_019592.7eU213-NP_062538.5aa42SL	0					0			3	15%	0%	350	360	740	210	380	TGGAAACAATTAAGCTAGGAGGTGTCTCTTCAACGG								14%	15%	15%
RNF20-NM_019592.7eU2892-NP_062538.5aa935TI	0					0			3	6%	1%	310	290	550	350	390	GTAACATGCGTAAAAAGGATGCTGTTCTTACTAAGT								5%	7%	6%
RNF214-NM_001077239.2eU950-NP_001070707.1aa302Q*	0					0			3	9%	1%	330	360	790	290	330	AAGATAGAGAAAGAGAAGAAGGAGTTTTTGCAGAAG								10%	9%	8%
RNF216-NM_001377156.1eU1972-NP_001364085.1aa579Q*	0					0			3	7%	3%	290	350	670	370	530	CCAACCAGTGAGCTGGAGAAGGTGCTCCCCCAGACC								4%	7%	10%
RNF26-NM_032015.5eU690-NP_114404.1aa32LF	0					0			2	3%	1%	250	280	630	140	420	TTGGACCTCAACTTCCTGCTGGTGTCCTCCCTCCTG									4%	2%
RNF34-NM_001256858.2eU1357-3'UTR+719	0					0			3	69%	5%	340	300	790	180	370	ACATTGTTTTGTATAGTGAAGGTGTATTTTCAGTGC								66%	67%	75%
RNH1-NM_002939.4eU697-NP_002930.2aa93Q*	0					0			3	5%	3%	400	260	560	330	210	CCCTCCTGCAAGATCCAGAAGCTGAGCCTCCAGAAC								4%	8%	4%
RNPEP-NM_001319182.2eU1023-NP_001306111.1aa307LL	0					0			3	2%	1%	390	220	530	260	430	GGGTGATCAGGATCAGTTTGACAGTTTTCTCAAGGC								2%	2%	3%
ROCK2-NM_001321643.2eU7741-3'UTR+3551	0					0			3	5%	1%	260	200	630	80	500	AAATTATAAATATTTGATCAGCTGTCCTCTCTGAAA								4%	7%	6%
RP2-NM_006915.3eU3016-3'UTR+1905	0					0			3	7%	2%	470	390	750	430	410	TTTGTATTTTTAGTTGAGATGGGGTTTTTCCACGTT								8%	4%	9%
RP9-NM_203288.2eU1094-3'UTR+396	0					0			3	47%	22%	270	160	740	220	290	GGTCCCATAGACATCAGGCTGGAGTCTTCTCTGTTG								27%	44%	71%
RPA1-NM_001355120.2eU1273-NP_001342049.1aa379SS	0					0			3	5%	1%	370	220	590	480	80	AGTCTCTGATTTCGGTGGACGGAGCCTCTCCGTGCT								5%	4%	6%
RPA3-NM_002947.5eU1632-3'UTR+94	0					0			2	2%	1%	320	220	510	220	340	TTGTTTGAGGGAGAGATTTCTGTGCTTTCTCATATT								3%		2%
RPL10A-NM_007104.5eU527-NP_009035.3aa165LL	0					0			3	18%	2%	250	300	710	190	390	ATCAAGTTCCAAATGAAGAAGGTGTTATGTCTGGCT								17%	16%	20%
RPL14-NM_001034996.3eU674-NP_001030168.1aa181Q*	4	2%	0%	2%	0%	0			3	2%	1%	260	240	710	350	320	AAAAAGGCTCCAGCCCAGAAGGTTCCTGCCCAGAAA	2%	2%	2%	1%				3%	1%	2%
RPL22-NM_000983.4eU641-3'UTR+232	0					0			3	7%	1%	410	320	690	270	430	ATACCAGAGTAGAAGGAGAGGGTGACTTTACCGAAC								8%	7%	7%
RPL35A-NM_000996.4eU168-NP_000987.2aa35AV	0					0			3	2%	0%	310	330	710	210	410	CAGCTCTTCTTAAAATTGAAGGTGTTTACGCCCGAG								1%	1%	2%
RPL5-NM_000969.5eU939-NP_000960.2aa288LL	0					0			3	2%	0%	490	180	450	420	570	TCGGGTAGCTCAAAAAGAGGCAAGCTTCCTCAGAGC								2%	2%	2%
RPL8-NM_000973.5eU515-NP_000964.1aa160SL	4	5%	2%	6%	1%	3	0%	0%	3	2%	0%	360	40	610	500	350	TGCCCTCCGGCTCCAAGAAGGTTATCTCCTCAGCCA	7%	6%	6%	3%	0%	0%	0%	2%	2%	2%
RPN2-NM_001135771.3eU1466-NP_001129243.1aa480SL	0					0			3	2%	1%	340	240	580	390	230	TCATCAAGTTCCCTGAGGAAGAAGCTCCCTCGACTG								3%	3%	2%
RPRD1A-NM_001303413.2eU169-NP_001290342.1aa5SF	0					0			2	7%	4%	440	110	470	400	300	AACGTGGTGTCGGAGGGATGTCAGCCTTCTCTGAGG									5%	10%
RPRD1B-NM_021215.4eU1645-3'UTR+462	0					0			3	7%	3%	380	280	430	650	280	AGGCACTTGTCCTGGAGACGTTGGCTTTCCCAGCTG								7%	10%	3%
RPS10-NM_001202470.3eU1093-3'UTR+179	0					0			2	6%	0%	510	320	640	380	300	GCTGTTTGGTGTTAAGTGATGGGGCTTTTTCTTCTG								6%	6%	
RPS13-NM_001017.3eU95-NP_001008.1aa23PP	0					0			3	9%	1%	480	70	620	400	140	GTCGGCTTTACCCTATCGACGCAGCGTCCCCACTTG								8%	9%	9%
RPS14-NM_001025070.2eU122-5'UTR-130	0					0			2	5%	2%	410	370	550	610	270	GGGGCTTGGGACGAGAGACGGGGGTCTTTCCGTGGG								4%	6%	
RPS14-NM_001025071.2eU122-5'UTR-136	0					0			2	5%	1%	410	370	550	610	270	GGGGCTTGGGACGAGAGACGGGGGTCTTTCCGTGGG								6%		4%
RPS21-NM_001024.4eU291-NP_001015.1aa80SL	4	12%	4%	14%	2%	0			3	1%	0%	220	210	760	80	290	TCCGATTGGCCAAGGCCGATGGCATCGTCTCAAAGA	12%	16%	13%	6%				1%	1%	1%
RPS21-XM_024451959.1eU1513-3'UTR+360	4	18%	3%	19%	1%	0			3	2%	0%	220	210	760	80	290	TCCGATTGGCCAAGGCCGATGGCATCGTCTCAAAGT	19%	20%	20%	13%				3%	2%	2%
RPS4X-NM_001007.5eU182-NP_000998.1aa43PP	1	0%		0%		3	2%	1%	3	10%	2%	420	300	670	360	510	CGGTCCCCACAAGTTGAGAGAGTGTCTCCCCCTCAT	0%				1%	2%	1%	10%	9%	12%
RPTOR-NM_001163034.2eU4551-3'UTR+225	0					0			2	12%	5%	310	310	770	240	280	AGGGGAGGGCTCGGGTTGACGGTGGCTTCCCACTGA								9%	16%	
RRAGA-NM_006570.5eU1380-3'UTR+205	0					0			3	10%	0%	370	260	620	160	170	GCTACCCAGTAGAGCTTGAAGCTGACCTTTCTGAGA								9%	10%	10%
RRBP1-NM_001042576.2eU3414-3'UTR+167	0					0			3	7%	0%	270	330	670	220	230	AGTCTCTTAGAGCCACAGAAGTAGACCTTCCAGAGC								7%	7%	7%
RRM1-NM_001033.5eU812-NP_001024.1aa200HY	0					0			3	2%	0%	440	100	760	380	430	TATAATCTTCTTTCTGAGAGGTGGTTTACTCATGCT								2%	3%	2%
RRP1B-NM_015056.3eU839-NP_055871.1aa245SF	0					0			3	14%	1%	250	210	690	80	430	AGAAGACAAAAGTGGGTGATGGTGACCTCTCTGCTG								15%	13%	15%
RRP1-NM_003683.6eU1028-NP_003674.1aa325YY	0					1	4%		3	16%	3%	460	320	730	210	320	CACCCCTTCTCAGAACAGAAAGCGTCTCTACAAAGT						4%		14%	19%	15%
RSRC1-NM_001271834.2eU2069-3'UTR+1127	0					0			3	16%	6%	350	260	690	460	410	TTTGTGTTTTTAGTAGAGATGGGGTTTCACCATTTT								15%	11%	22%
RSRC2-NR_036434.2eU334-ncr	0					0			3	4%	2%	300	0	630	210	240	GAAGAGACTGAACTGAAGACGCTGCAGACTCAGATA								3%	6%	4%
RTEL1-NM_001283009.2eU264-5'UTR-63	0					0			2	8%	2%	540	390	620	290	260	GAGACCCTAAGATGTTCGGAGTGGTTTTTTCGCACA								9%		7%
RTKN2-NM_145307.4eU2231-3'UTR+167	0					0			2	25%	4%	400	250	750	330	280	AAACATTTTATAATGAAGAAGTGGATTTCTCTTTTA									23%	28%
RTKN-NM_001015055.2eU1698-NP_001015055.1aa526PL	0					0			2	34%	9%	250	120	660	370	300	CCCGAACCTTTTTCCTGGATGCTGTCCCCCCAGACC								40%	27%	
RTN4-NM_001321859.2eU1085-NP_001308788.1aa156SL	0					0			3	19%	5%	520	140	690	290	130	AATTGGTTAAAGAGGATGAAGTTGTGTCTTCAGAAA								24%	15%	18%
RUNDC1-NM_001321381.3eU2384-3'UTR+524	0					0			2	10%	4%	280	90	530	440	470	GTCTCAAAAAAAAAAAGAAGGTTGTACCCACAGTAT								7%		13%
RUVBL1-NM_001319084.2eU495-NP_001306013.1aa136TI	0					0			3	4%	1%	200	240	690	120	330	AGACCAAGGAAGTTTATGAAGGTGAAGTCACAGAGC								4%	4%	3%
RWDD2B-NM_001320724.2eU477-NP_001307653.1aa74LL	0					0			3	9%	3%	310	210	650	210	460	GTATCTGACGAAAAAATGGCGATGTTTTCTCTGGCC								12%	9%	6%
RXRA-NM_001291920.2eU5459-3'UTR+3229	0					0			2	8%	4%	340	210	630	420	270	TTGTGTTTATTTTTAATCAAGACGTTTCCCCTGTTT								5%		11%
RYBP-NM_012234.6eU134-5'UTR-51	0					0			2	32%	4%	470	280	640	500	210	TCCGCGACGACTCCCCGGACGGCGTTTCTCCTCCGA									35%	30%
S100A11-NM_005620.2eU241-NP_005611.1aa51TI	0					0			3	4%	0%	260	200	650	230	330	GCTTCATGAATACAGAACTAGCTGCCTTCACAAAGA								3%	4%	3%
S1PR1-NM_001320730.2eU1424-3'UTR+19	0					0			3	16%	1%	250	200	700	300	300	TCTTCTTCCTAGAACTGGAAGCTGTCCACCCACCGG								15%	17%	16%
SACM1L-NM_001319071.2eU1668-NP_001306000.1aa533SL	0					0			3	19%	3%	340	180	620	270	140	CTTTGCCTATTATCATGGTTGTTGCCTTTTCAATGT								16%	18%	22%
SACS-NM_001278055.2eU9407-NP_001264984.1aa2973RC	0					0			2	12%	6%	310	150	690	310	180	ACTAATTGCCATATTGGGAAGCTGCCTTGTCGTCTG									8%	17%
SAMD9L-NM_001303496.3eU4647-NP_001290425.1aa1206YY	0					0			3	6%	1%	550	210	550	510	240	TTTCTTGGGTGAAATAGAAGTTGGTCTTTACACTAT								8%	5%	6%
SAMD9-NM_001193307.2eU5247-3'UTR+352	0					0			3	14%	4%	480	130	600	390	350	CCAGGCATGGATTGAAGAAGATTGTCTACTCAAATG								9%	16%	17%
SAP130-NM_001145928.2eU2473-NP_001139400.1aa777SL	0					0			3	5%	1%	280	230	590	130	250	GTCCCCCGTCACAACCAGCCGTTGCCCTTTCAACCA								5%	4%	5%
SAP18-NM_001366643.2eU490-NP_001353572.1aa93PL	0					0			2	3%	0%	300	190	730	320	290	AACTGACAAGCTTAGTAAAAGAAGTCTACCCAGAAG								3%		3%
SAP18-NM_005870.4eU329-NP_005861.2aa97PL	0					0			3	2%	1%	300	190	730	320	290	AACTGACAAGCTTAGTAAAAGAAGTCTACCCAGAAG								2%	3%	2%
SARS1-NM_001330669.1eU608-NP_001317598.1aa170HY	0					0			3	23%	1%	250	200	750	200	280	GGTGATTGTACAGTCAGGAAGAAGTACTCTCATGTG								21%	24%	24%
SARS1-NR_034072.1eU2512-ncr	0					0			2	16%	9%	320	150	730	280	410	TGTGTATTTTTTGTAGAGATGGAGTTTCGTCATGTT								10%	23%	
SAV1-NM_021818.4eU349-NP_068590.1aa4R*	0					0			3	5%	1%	360	290	480	590	340	CCTCGGGCCTCGTCGAGAAGGATGCTGTCCCGAAAG								6%	5%	4%
SCAF11-NM_004719.3eU410-NP_004710.2aa56PL	0					0			3	11%	2%	330	420	650	470	200	ATTGTCTATTAGAAAAAGAAGTTGGTTTTCCAGAAA								10%	11%	13%
SCAF11-XM_024449274.1eU455-5'UTR-1131	0					0			2	18%	1%	330	420	650	470	200	ATTGTCTATTAGAAAAAGAAGTTGGTTTTCCAGAAA								17%		18%
SCAF11-XM_024449274.1eU588-5'UTR-998	0					0			2	11%	2%	300	380	600	220	320	ATAACCAGAGCAGATACGATAGTGTCCTTCCATTTT									9%	12%
SCAF8-NM_001286188.1eU319-NP_001273117.1aa92LL	0					0			3	14%	6%	260	300	570	320	360	GTGAAGACCTTCAATAGCGAGTTGTATTCCCTGAAT								11%	10%	21%
SCAF8-NM_014892.5eU525-NP_055707.3aa14LL	0					0			3	20%	5%	260	300	570	320	360	GTGAAGACCTTCAATAGCGAGTTGTATTCCCTGAAT								25%	14%	21%
SCAMP3-NM_005698.4eU1180-NP_005689.2aa324SF	0					0			2	4%	3%	280	310	740	100	350	CCCAGCAAGAATTTGCTGCTGGTGTCTTCTCCAACC								2%		6%
SCAMP4-NM_001329539.2eU1403-3'UTR+679	0					0			3	3%	1%	460	200	640	490	290	AAAACAGGGATCCCTGGCAGGCTGTCTTTCCACGCC								2%	4%	3%
SCCPDH-NM_016002.3eU941-NP_057086.2aa309LL	4	50%	15%	57%	3%	3	6%	1%	3	14%	2%	280	240	810	220	220	TGTGAGGTTTGGAATTGGAAGGCAACTTCTCATAAA	59%	60%	53%	27%	7%	5%	7%	13%	16%	13%
SCD-NM_005063.5eU1465-3'UTR+113	0					0			3	3%	1%	190	130	610	290	290	ACCAGGATGCTAAAGATGATGATGTTAACCCATTCC								2%	3%	2%
SCD-NM_005063.5eU2775-3'UTR+1423	4	8%	3%	10%	2%	0			3	9%	0%	440	300	740	410	230	GAAATGAGTAAAAACTAGAAGGCTTCTCTCCACAGT	11%	10%	8%	4%				8%	9%	9%
SCD-NM_005063.5eU5090-3'UTR+3738	0					0			3	54%	0%	540	130	690	320	260	TGCCACGGAAACATACAGAGGATGCCTTTTCTGTGA								54%	54%	54%
SCN9A-NM_001365536.1eU1892-NP_001352465.1aa532PS	0					0			2	14%	6%	440	170	720	370	390	AGGCGAGCACATGAAAAGAGGTTGTCTACCCCCAAT								18%	9%	
SCO1-NM_004589.4eU963-3'UTR+31	0					0			2	8%	4%	310	70	730	260	210	CCAAAGCAGTGTTGCTGGATGCAGTATTCTCTTGCT								5%		11%
SCRN1-NM_001145513.1eU2049-3'UTR+627	3	3%	1%	3%	0%	0			3	38%	7%	210	220	870	320	350	CATCCTGGGCTCCTGAAGAAGCTGTCTTCCCATTTT		3%	4%	2%				37%	46%	31%
SDC4-NM_002999.4eU1655-3'UTR+1018	0					0			3	15%	3%	450	140	650	370	220	TGGCCTTGGCCCTTTCTGAAGCTGCTTCCTCTTATA								12%	18%	17%
SDC4-XM_011528977.2eU1510-3'UTR+1018	0					0			3	50%	7%	450	140	650	370	220	TGGCCTTGGCCCTTTCTGAAGCTGCTTCCTCTTATA								56%	43%	51%
SDCBP-NM_001007067.2eU754-NP_001007068.1aa233LL	0					0			2	2%	0%	540	230	820	180	290	AGATAGCTCTGCAGCCAGAAATGGTCTTCTCACGGA								2%	2%	
SDF4-NM_016176.6eU586-NP_057260.3aa101SF	4	5%	2%	6%	1%	0			2	1%	0%	390	320	510	390	190	GGAGCCGGAGGAAGCTGATGGTCATCTTTTCCAAGG	7%	6%	6%	2%				1%		1%
SDHA-NM_001294332.2eU846-NP_001281261.1aa270LL	0					0			3	2%	0%	480	230	560	520	310	GGAAGGATGTCGTGGAGAGGGAGGCATTCTCATTAA								2%	2%	1%
SDHB-NM_003000.3eU270-NP_002991.2aa86TI	0					0			3	4%	2%	370	140	590	290	150	TAATCAAGATTAAGAATGAAGTTGACTCTACTTTGA								5%	5%	2%
SDSL-NM_001304993.2eU162-NP_001291922.1aa15HY	0					0			2	7%	2%	380	350	670	170	270	GTGGCAGAGCATGCCAAGCAGGAGCCCTTTCACGTG								8%	5%	
SEC14L1-NM_001039573.3eU900-NP_001034662.3aa209PL	0					0			3	5%	1%	160	140	680	220	250	AACAAGCAGCGTCCATGGCCGTCGTCATCCCAGAAG								4%	6%	5%
SEC14L1-NM_001143998.2eU3568-3'UTR+1146	4	54%	21%	64%	4%	0			3	21%	5%	220	240	790	180	380	AACTTAGTTTGAGTAATGAAGGAATCTTCACAGAAG	60%	68%	65%	24%				16%	25%	21%
SEC16A-NM_001276418.2eU6045-NP_001263347.1aa1901PL	0					0			3	7%	2%	340	280	650	350	290	CTGGAGTATGGCATCAGGATGGAGCCCTCCCGCAGC								4%	8%	8%

SEC22B-NM_004892.6eU378-NP_004883.3aa80PL	3	3%	1%	3%	1%	0		3	50%	5%	340	270	720	330	280	GTTATTTGGTTTTATGTGAAGCTGCCTTCCCTAAGA	3%	4%	2%			45%	52%	53%
SEC24D-NM_001318066.2eU3362-3'UTR+33	0					0		3	13%	7%	320	250	610	330	140	GAAACTTCTCTGTCATTGATGTTGCATTTCCAAGGA						15%	5%	18%
SEC61A2-NM_001142627.3eU1042-NP_001136099.1aa311R*	0					0		3	19%	8%	340	110	690	220	210	CTGTATGTTATTTCCCAGATGCTGTCTGTTCGATTT						16%	13%	28%
SEC62-NM_003262.4eU290-NP_003253.1aa92HY	0					0		3	44%	3%	440	270	790	330	290	TGCAACAGGCTTTTAAAGAAGCAGTTTTTTCACCGA						45%	41%	45%
SECISBP2L-NM_001193489.2eU6278-3'UTR+2825	0					0		3	12%	2%	410	190	670	420	270	TTTACTGGCTTTGACTGGAGGTTGTTTTTGCAGAGA						14%	12%	10%
SEL1L3-NM_001297592.2eU2384-NP_001284521.1aa777PL	3	18%	2%	18%	2%	0		2	8%	3%	210	230	740	320	290	TTGGAGTCCTGCATTTGGATGGCATCTTCCCTGGAG	20%	19%	16%			6%	10%	
SEL1L3-NM_001297592.2eU327-NP_001284521.1aa91PP	0					0		3	48%	12%	250	220	710	290	220	TGTTTCATCTGAGTTCAGAAGTAGCATTCCCGTGTA						44%	40%	62%
SELENOH-NM_001321335.2eU412-NP_001308264.1aa103RC	0					0		3	19%	1%	480	260	670	320	380	CTCTGGACTGGGATTAAGAAGGGGCCCCCACGCAAA						19%	18%	20%
SELENOH-NM_170746.4eU412-NP_734467.1aa103RC	0					0		3	15%	3%	480	260	670	320	380	CTCTGGACTGGGATTAAGAAGGGGCCCCCACGCAAA						19%	14%	14%
SELENON-NM_020451.3eU1901-3'UTR+90	0					0		2	12%	3%	400	150	670	170	250	GCCCATTTCAGACTGCAGATGCCGCCCACTCCCACC						9%		14%
SELENOW-NM_003009.4eU175-NP_003000.1aa31PL	0					0		3	3%	1%	330	320	710	240	280	AGCTCAAGAAGAAGTTAGAAGATGAGTTCCCCGGCC						2%	4%	2%
SEM1-NM_006304.2eU241-NP_006295.1aa53SF	0					0		3	6%	1%	480	120	760	230	320	GGGATGATGACAATGTAGAGGATGACTTCTCTAATC						5%	5%	7%
SEMA3C-NM_001350120.2eU1359-NP_001337049.1aa417TI	0					0		2	8%	1%	230	130	660	150	460	CCACCAAGGAGTTCCCAGATGATGTTGTCACTTTTA						8%	8%	
SEMA3C-NM_001350120.2eU667-NP_001337049.1aa186FF	0					0		3	7%	2%	320	290	580	400	270	GTGTGAATCTGGAAAAGGACGCTGCTCTTTCAACCC						4%	8%	8%
SENP7-NM_001077203.3eU2954-NP_001070671.1aa958RC	0					0		3	18%	6%	410	180	750	360	390	CTTCCAATTCATTTGGAGAAGTGGTTTCCTCGTCAT						11%	20%	22%
SEPTIN11-NM_001306147.2eU4927-3'UTR+3265	0					0		2	5%	1%	210	340	730	270	490	AATTTATTGGCAGTTCAGATTGTGTTTTCCCAACTT						6%		4%
SEPTIN6-NM_015129.6eU3076-3'UTR+1708	0					0		3	32%	6%	370	290	460	610	380	ACTTCTCTTAAGCCAAGAAGTTTGCTTTCCCTAGCT						36%	25%	33%
SEPTIN9-NM_001113491.2eU1048-NP_001106963.1aa337PP	0					0		2	2%	0%	420	370	450	570	350	GGTGCAGCCCACCTCAGAGGAGCGCATCCCCAAGAC							2%	2%
SERINC1-NM_020755.4eU309-NP_065806.1aa80PL	0					0		3	12%	4%	190	410	620	450	450	GATTTTGTGAGAATGAGAAAGGTGTTGTCCCTTGTA						9%	10%	16%
SERPINB1-NM_030666.4eU2183-3'UTR+983	0					0		3	41%	12%	370	390	750	430	450	TTTGTATTTTTAGTAGAGATGGGGTTTTTCCATGTT						31%	38%	55%
SERPINE2-NM_001136528.2eU746-NP_001130000.1aa179TI	0					0		2	6%	1%	230	360	660	80	510	TGTCCCCAGATCTTATTGATGGTGTGCTCACCAGAC							6%	6%
SERTAD2-NM_014755.3eU5441-3'UTR+4198	0					0		3	44%	2%	250	110	580	430	400	GTTACATCGGAACCCAGATGGCTGTATTCACATGTA						42%	46%	43%
SESTD1-NM_178123.5eU440-NP_835224.3aa27LL	0					0		2	31%	15%	340	90	730	150	370	TTCAGGAGGAAAAGACAGACGGAGTGGCCTCATTTT						20%		42%
SETD2-NM_001349370.3eU1772-NP_001336299.1aa489PP	0					0		3	58%	4%	250	350	660	290	520	AAATGAAGCAATTAAAAGATGTTGTTCTCCCCCTAA						63%	58%	54%
SETD5-NM_001080517.3eU1204-NP_001073986.1aa222TI	0					0		3	6%	1%	380	260	670	300	250	TATGGACTGACCAGTATGAAGAAGCTTTCACTAATC						5%	6%	8%
SETDB1-NM_001145415.2eU2491-NP_001138887.1aa775PP	0					0		2	18%	4%	520	240	600	490	390	CCAGTACAAGAGACTAGAAGAGTGTCTACCCACAGG							16%	21%
SF3A1-NM_005877.6eU188-NP_005868.1aa29SL	0					0		3	9%	2%	520	190	670	340	80	CCAAACAGCCCACAGAAGAAGAAGCATCTTCAAAGG						8%	8%	11%
SF3A1-NM_005877.6eU3169-3'UTR+685	0					0		3	3%	1%	230	230	600	310	320	CATGCAAACCAAGGATGGATGGTGCAGTCCCTGTGT						4%	2%	4%
SF3A3-NM_001320830.2eU1268-NP_001307759.1aa404SF	0					0		3	3%	1%	290	310	640	200	300	CTAATGTGACACAGATTGAAGATGCTGTCTCCTTGT						2%	3%	3%
SF3B1-NM_012433.4eU1255-NP_036565.2aa409PL	4	29%	9%	33%	4%	0		3	5%	1%	300	140	700	280	350	TTTCTGATGAGGAATTAGATGCTATGTTCCCAGAAG	36%	34%	29%	17%		5%	5%	6%
SF3B3-NM_012426.5eU3118-NP_036558.3aa979R*	0					0		2	2%	0%	360	320	570	400	250	GTCTATGACCTGGGAAAGAAGAAGTTACTCCGAAAA							2%	2%
SFT2D1-NM_145169.3eU612-3'UTR+68	0					0		3	10%	4%	550	400	390	610	240	GTACTCTATGTTTGGTGAAGTTTGCTTTTCCCCATA						16%	8%	8%
SGCB-NM_000232.5eU199-NP_000223.1aa53HH	0					0		3	32%	3%	470	130	600	420	340	ATACATTCCGATTGATGAAGATCGTCTCCACAAAAC						30%	29%	35%
SGCB-NM_000232.5eU3479-3'UTR+2482	0					0		2	8%	4%	350	260	690	460	410	TTTGTATTTTTAGTAGAGACGGGGTTTCACCATGTT						5%		10%
SH3BP2-NM_001122681.2eU711-NP_001116153.1aa218SF	1	14%		14%		0		3	40%	6%	480	110	550	440	380	CAGTGCCCACGCCCAGGAAGCCAGCCTTCTCTGACA	14%					35%	46%	38%
SH3KBP1-NM_001024666.3eU934-NP_001019837.1aa282PL	0					0		3	5%	2%	300	490	690	200	580	GAGAGCTGAACGGCAGACGAGGCGTGTTCCCCGATA						5%	3%	7%
SIK2-NM_015191.3eU4921-3'UTR+2025	4	18%	7%	21%	6%	0		3	72%	10%	300	330	820	430	310	ATTTGTTTAGACTGAAGGAAGACGTTTTCCCAAAAT	18%	27%	16%	11%		61%	75%	81%
SIKE1-NM_001102396.2eU4768-3'UTR+4054	0					0		3	26%	8%	420	320	690	500	290	TTTTTGTGTTTTTAGTAGATGGGGTTTCTCCATGTT						23%	19%	34%
SIN3A-NM_001145357.2eU2138-NP_001138829.1aa642RC	0					0		3	22%	8%	390	280	710	360	320	GTTCTGGAAGCAATACAGAAGAAGCTTTCCCGCTTG						21%	31%	16%
SIN3B-NM_001297595.2eU160-NP_001284524.1aa46TI	0					0		3	24%	5%	280	300	730	130	340	AGCTGCCGGTGCACGTAGAAGACGCCCTCACCTATC						19%	24%	30%
SINHCAF-NM_001135811.2eU1226-3'UTR+271	0					0		3	13%	3%	450	260	690	460	410	TTTTTAGTTTTAGTAGAGACGGGGTTTCACCACGTT						17%	11%	12%
SINHCAF-NM_001135811.2eU2320-3'UTR+1365	0					0		3	3%	0%	330	190	490	350	530	ACTTACTAAAAGAAAAGAGGTTTGTGTTCACATTAA						3%	4%	3%
SIRT1-NM_001142498.2eU1547-NP_001135970.1aa372SF	0					0		3	6%	2%	310	170	790	180	190	TATATTCAGACTCTGAAGATGACGTCTTATCCTCTA						4%	8%	6%
SIX5-NM_175875.5eU3040-3'UTR+412	0					0		2	8%	3%	110	270	670	110	580	TAATAGCTCTGCCGATAGCTGGTGTTGTCACAACTG						10%	5%	
SKAP2-NM_001303468.2eU638-5'UTR-86	0					0		3	13%	7%	250	290	670	80	550	ATTTGAATGGCAGAAACGGTGGTGTGCTCTCAGTAA						10%	9%	21%
SKP2-NM_001243120.2eU1208-3'UTR+28	3	7%	4%	7%	4%	0		3	48%	2%	430	150	690	370	210	TGAAGTATTTATTGCAGGATGGTGTCTCTTCTTTAG	11%	6%	4%			48%	47%	51%
SLC15A4-NM_145648.4eU1024-NP_663623.1aa337Q*	0					1	13%	3	10%	2%	410	380	620	320	250	GCTTTGATACCTTACTGGACAGTGTATTTCCAAATG					13%	8%	11%	11%
SLC16A1-NM_001166496.2eU1229-NP_001159968.1aa88VV	0					0		3	4%	0%	270	80	730	220	110	CCTGGTGAATAAATATGGAAGTCGTATAGTCATGAT						4%	4%	3%
SLC20A1-NM_005415.5eU2124-NP_005406.3aa556SL	0					0		3	3%	2%	440	330	650	280	400	ATTTGGTTTATGACACAGGAGATGTTTCTTCAAAAG						3%	1%	4%
SLC20A2-NM_001257180.2eU1223-NP_001244109.1aa253SL	0					0		3	35%	6%	410	300	770	300	360	CAGGCAAATTACAAAAAGAAGGTGCTTTATCACGAG						29%	34%	41%
SLC23A2-NM_005116.6eU679-NP_005107.4aa98PL	0					0		3	12%	7%	310	420	570	360	380	ACATGATTTATACCATAGAAGATGTTCCTCCCTGGT						7%	20%	10%
SLC25A16-NM_001324312.2eU1164-3'UTR+68	0					0		3	36%	13%	440	270	750	330	170	ACAACATACGAACTTATGAAGCAGTTTTTTCACCTC						24%	50%	33%
SLC25A16-NM_001324314.2eU1318-NP_001311243.1aa232HY	0					0		2	18%	1%	440	270	750	330	170	ACAACATACGAACTTATGAAGCAGTTTTTTCACCTC						18%	19%	
SLC25A17-NM_006358.4eU183-NP_006349.1aa29PS	0					0		2	13%	0%	520	420	610	250	350	GGAAGCGTGACAGCAATGACAGTGTTTTTTCCCCTG						13%	14%	
SLC25A26-NM_001164796.1eU924-NP_001158268.1aa155PL	0					0		2	11%	1%	310	310	740	200	350	GGCTGGCAGGATTATTTGCAGGTGTCTTCCCTCGAA							11%	12%
SLC25A6-NM_001636.4eU1346-3'UTR+345	0					0		3	13%	8%	250	240	610	470	390	TTTATTTAAAACAAAAGAATCACGTTTTCCCATTTG						6%	14%	21%
SLC30A7-NM_001144884.2eU183-NP_001138356.1aa4LL	0					0		2	12%	3%	280	210	640	320	490	GCCCTTCGCCGGGCAGAGAAGATGTTGCCCCTGTCC						14%	10%	
SLC30A7-NM_001144884.2eU5825-3'UTR+4521	0					0		3	13%	7%	430	270	690	230	160	TGCATGATTTTTATGATTGACGGAGCATTTCAAATC						13%	20%	6%
SLC35A2-NM_001042498.3eU411-NP_001035963.1aa134SF	0					0		2	6%	0%	340	120	570	250	200	TGCAGAATAACCTCCAGTATGTTGCCATCTCTAACC						6%		6%
SLC35A5-NM_001348908.2eU207-5'UTR-167	0					0		3	20%	1%	360	440	430	460	520	ACTGTCTCAGCTCTAGGATGTGCGTTCTTCCACTAG						21%	19%	19%
SLC35B2-NM_001286509.2eU1521-3'UTR+108	3	3%	1%	3%	1%	0		3	37%	5%	230	300	780	210	450	GGCTGAAAGGGCAAAATGCAGGTGTTTTCTCAGTAT	4%		3%	2%		39%	32%	41%
SLC35B4-NM_032826.5eU612-NP_116215.1aa145Q*	0					0		2	6%	0%	170	310	770	240	400	ACTTTTATGTCAGCAAAGCAGGTGACTTCCCAGTCC							7%	6%
SLC35C1-NM_001145265.2eU2428-3'UTR+1229	3	17%	5%	17%	5%	0		3	54%	12%	410	160	800	190	400	CTGTTTCCCGATCCATAGATGGTGCCTTCTCGCTGT	22%	13%	17%			44%	67%	50%
SLC35D2-NM_001286990.2eU366-NP_001273919.1aa97LL	0					0		2	6%	2%	360	180	630	400	410	GATAAGAAAATTCCTGTAAAGCTGTTTCCTCTGCCT						5%		8%
SLC35D2-NM_007001.3eU510-NP_008932.2aa145LF	0					0		2	8%	1%	400	150	730	260	340	GAAACCATCATACTTGGGAAGCAGTATTCACTCAAC						9%		8%
SLC35E2B-NM_001110781.3eU3666-3'UTR+1954	0					0		3	25%	7%	410	210	750	210	310	TGTGCTCACTGCGCTGCGAAGATGTTTTGTCACGTG						22%	33%	19%
SLC39A6-NM_012319.4eU325-NP_036451.4aa30PL	0					0		3	19%	8%	360	380	630	400	380	CCCTTCATGAACTAAAAGCAGCTGCTTTCCCCCAGA						22%	11%	26%
SLC43A3-NM_001278201.2eU1443-NP_001265130.1aa369LL	0					0		2	5%	1%	290	160	720	190	280	GCGGTGGCCCTCTGCTCGACGGTGCCTTCGCTGGCC							5%	5%
SLC52A2-NM_001363118.2eU470-5'UTR-95	0					0		3	25%	3%	290	150	820	140	340	GGCGGTCCCGCTGGCTAGAAGAAGTCTTCACTTCCC						22%	29%	24%
SLC5A6-NM_021095.4eU2786-3'UTR+422	0					0		3	23%	5%	340	210	610	550	220	TCTCCACCATAAAACTGGAAGCTGCTTCCCCTGTAG						26%	16%	26%
SLC7A11-NM_014331.4eU3108-3'UTR+1322	0					0		3	39%	14%	210	200	710	350	310	AGTGTTTGTGTTCAGAAGATGTTGTTTTGCCAGTAT						28%	55%	36%
SLC7A1-NM_003045.5eU4417-3'UTR+2140	0					0		3	2%	0%	550	400	620	400	300	CTCATTTTGTAGATTGAGAAGGTGCGTCTCCCCTGC						1%	2%	1%
SLC9A6-NM_001042537.2eU3889-3'UTR+1715	0					0		3	41%	7%	570	190	430	470	260	CGTGTATGTTTTACATGATGTGTGCCTCTTCACGCA						37%	50%	38%
SLFN11-NM_001104587.2eU756-NP_001098057.1aa104YY	2	3%	1%	3%	1%	0		3	57%	10%	280	360	750	370	400	TGAGACCAAGCAACAAGGAAGGTGTTTTTACATTTT		2%	3%			51%	69%	52%
SLX4IP-NM_001009608.3eU2422-3'UTR+1017	0					0		2	25%	4%	330	270	560	540	340	GCAGTTCAAATTTCAAGATGGGAGCCTTCCCTTTAC						22%	27%	
SMAD1-NM_001003688.1eU991-NP_001003688.1aa251LL	0					0		3	51%	12%	340	160	700	400	180	CCGATGGACACAAACATGATGGCGCCTCCCCTGCCC						38%	62%	54%
SMAD2-NM_001003652.4eU2057-3'UTR+527	0					0		2	8%	4%	320	340	640	390	180	GATTTTCAAATTATTTAGAAGCAGATTTTCCTGTAG						11%	5%	
SMAD3-NM_001145102.2eU2132-3'UTR+817	1	5%		5%		0		3	41%	10%	430	150	570	150	500	CAAAGAGATTCGAATGACGGTAAGTGTTCTCATGAA		5%				47%	29%	47%
SMARCA2-NM_001289396.1eU4325-NP_001276325.1aa1372PP	0					0		2	11%	1%	370	190	690	400	330	AAAAGCTAAGAAGAGAAGAGGCCGCCCTCCCGCTGA							10%	11%
SMARCA2-NM_001289397.2eU4036-NP_001276326.1aa1314PP	0					0		2	8%	5%	370	190	690	400	330	AAAAGCTAAGAAGAGAAGAGGCCGCCCTCCCGCTGA							11%	4%
SMARCA4-NM_001128844.3eU1165-NP_001122316.1aa305PS	0					0		3	16%	4%	260	310	650	420	300	CCCACGAGCACCCCTCAGAAGCTGATTCCCCCGCAG						20%	17%	12%
SMARCA4-NM_001128847.4eU186-NP_001122319.1aa4PS	0					0		2	10%	1%	250	280	690	80	380	CTGTCTGCAGCTCCCGTGAAGATGTCACTCCCAGAC						10%	9%	
SMARCAD1-NM_001128429.3eU566-NP_001121901.1aa104PL	0					0		3	63%	20%	310	180	760	420	310	TGTCTTCTGATAGTGAAGATGTCGTTTCCCCAAATT						45%	59%	85%

SMARCC1-NM_003074.4eU1253-NP_003065.3aa386PL	0					0			3	5%	1%	270	480	630	260	260	CTGTACCCAATATAGAAGAAGTAGTACTTCCCAAAA								5%	6%	4%
SMARCC1-NM_003074.4eU1254-NP_003065.3aa386PP	0					0			3	3%	1%	430	270	480	630	260	TGTACCCAATATAGAAGAAGTAGTACTTCCCAAAAA								5%	2%	2%
SMC2-NM_001042550.2eU3673-NP_001036015.1aa1134HY	0					0			3	13%	1%	390	0	700	230	280	ACCCAAAACATTGGACAGATGCTGCGTACTCATTTC								14%	11%	14%
SMC2-NM_001042550.2eU3773-NP_001036015.1aa1167TI	1	3%		3%		0			3	51%	4%	330	210	650	400	320	TCAAAACCAAGTTTGTGGATGGTGTTTCTACAGTAG		3%						48%	54%	53%
SMC5-NM_015110.4eU132-NP_055925.2aa11PS	0					0			3	12%	3%	300	230	770	120	250	ATGGCGACTCCGAGCAAGAAGACGTCAACTCCAAGC								11%	9%	16%
SMCHD1-NM_015295.3eU6457-3'UTR+89	0					0			3	2%	1%	340	230	740	370	300	CTGTTTCAGAAGACCAAGAGGGTGACTTACCAGACT								2%	3%	2%
SMDT1-NM_033318.5eU164-NP_201575.3aa32SF	0					0			3	8%	1%	210	320	720	190	400	GGCCTAGCTTGAGGAAAGATGGCGATGTCTCCGCCG								8%	9%	8%
SMG1-NM_015092.5eU13720-3'UTR+2367	0					0			2	14%	2%	430	250	660	220	340	ACTATAGATAGTATTCAGAAATTGCTTTCTCTTTGG								16%	13%	
SMG1P3-NR_027155.2eU160-ncr	0					0			2	9%	2%	340	70	700	360	300	CGCAGGGCCCTGGAAGAGACGTCGCCTCCCCTTCAT								8%	11%	
SMPD4BP-NR_026922.1eU740-ncr	0					0			3	6%	2%	220	350	710	220	300	TACCTGCCTGGTCCTGTGAAGGCGTCCATCCAGGAG								5%	4%	8%
SMPD4-NM_017751.4eU640-NP_060221.2aa162Q*	0					0			3	6%	1%	220	350	710	220	300	TACTTGCCTGGTCCTGTGAAGGCGTCCATCCAGGAG								6%	5%	7%
SMTN-NM_001207018.2eU2839-NP_001193947.1aa921SL	0					0			3	5%	2%	480	110	770	360	180	ACCGACGCCAGAACTTCGAGGTGGCCTTCTCATCTG								3%	6%	4%
SMU1-NM_018225.3eU2192-3'UTR+584	0					0			3	15%	8%	350	260	690	460	410	TTTGTATTTTTAGTAGAGATGGGGTTTCACCATGTT								18%	5%	21%
SNAP47-NM_001323930.2eU385-NP_001310859.1aa62HY	0					0			3	6%	2%	210	320	850	180	350	AGCATAGTTGAGATCAAGAAGGAGGCTTCACATTTT								5%	5%	7%
SNAPC5-NM_001329614.2eU337-NP_001316543.1aa14HY	3	13%	5%	12%	7%	0			3	30%	11%	420	70	870	180	430	TCTAGAAGAGGGGATGAGATGCTGTCTTCTCACACT		17%	8%	14%				21%	42%	28%
SNAPC5-NM_001329615.2eU185-NP_001316544.1aa52HY	4	10%	3%	11%	4%	0			3	34%	3%	420	70	870	180	430	TCTAGAAGAGGGGATGAGATGCTGTCTTCTCACACT	13%	7%	13%	7%				32%	37%	34%
SNHG12-NR_024127.2eU438-ncr	0					0			3	67%	3%	380	360	720	250	360	AGACTCTTAAGATGACAGAAGGTGATTTTTCTGGTG								66%	65%	70%
SNHG12-NR_146383.1eU438-ncr	0					0			3	69%	31%	380	360	720	250	360	AGACTCTTAAGATGACAGAAGGTGATTTTTCTGGTG								89%	86%	33%
SNHG1-NR_003098.2eU457-ncr	0					0			3	4%	2%	390	450	720	180	260	TGTCTTGGAGGACATCAGAAGGTGAATTTTCCAAGT								4%	3%	6%
SNHG29-NR_027159.1eU565-ncr	4	24%	9%	28%	1%	0			3	6%	1%	300	180	580	530	350	GCATCTCAGCCCAAGAGAAGGTTATATTCCCAGAGG	29%	27%	29%	11%				8%	6%	6%
SNHG29-NR_027160.1eU688-ncr	4	23%	7%	27%	2%	0			2	7%	2%	300	180	580	530	350	GCATCTCAGCCCAAGAGAAGGTTATATTCCCAGAGG	24%	28%	27%	13%				8%	5%	
SNIP1-NM_024700.4eU2217-3'UTR+962	0					0			3	18%	5%	360	230	660	270	460	TTAAAAAGTTGTTGAGAGAGGTTGTATTTACCTTCC								16%	15%	23%
SNIP1-NM_024700.4eU341-NP_078976.2aa93RC	0					0			3	26%	7%	590	40	550	460	330	AAGGCCTCAGGGAGAAGAAGCAAGTCTCCTCGCAGT								18%	33%	27%
SNRNP200-NM_014014.5eU190-NP_054733.2aa27LL	0					0			3	2%	1%	330	220	500	320	420	GAATCTTGTGCTCCAAGCTGACCGTTCTCTCATTGA								2%	3%	1%
SNRNP25-NM_024571.4eU254-NP_078847.2aa22SF	0					0			2	3%	0%	400	180	690	160	170	CGATCCAGGTTACTCTGGAAGAAGTCAACTCCCAAA								3%	3%	
SNRNP48-NM_152551.4eU940-NP_689764.3aa294R*	0					0			2	5%	1%	250	290	520	60	590	GGTGGAAGCTATTTGGATGCTGAGTGTTCACGACAT									4%	6%
SNRNP70-NM_001301069.2eU1498-3'UTR+15	0					0			2	2%	0%	290	270	790	210	400	GGCTGCGCCGGAGTGAAGAGGTCGTCCTCTCCATCT									2%	2%
SNRPC-NM_003093.3eU490-NP_003084.1aa151RW	0					0			3	4%	1%	250	140	630	170	310	CCTCCTGCCCGTCCCATGATGGTGCCCACTCGGCCC								5%	3%	4%
SNRPF-NM_003095.5eU289-NP_003086.1aa56SF	0					0			3	9%	2%	350	220	680	260	300	ATACAGAAGAATACATAGATGGAGCTTTGTCTGGAC								8%	8%	11%
SNW1-NM_001318844.2eU223-NP_001305773.1aa65PL	0					0			3	5%	1%	330	510	610	400	330	TAGAGGATTTTGGAGATGGAGGTGCTTTTCCAGAGA								5%	3%	6%
SNX14-NM_001297614.3eU1308-NP_001284543.1aa390HY	0					0			3	5%	2%	390	100	660	370	330	GAATTATCAAATGATGAAATGCTGTCTCTTCATGAA								5%	4%	7%
SNX14-NM_001297614.3eU1759-NP_001284543.1aa540PL	0					0			3	9%	1%	370	270	590	500	160	AAGGTATTGTTGTAATGGAAGATGATTCTCCAGTGG								8%	10%	9%
SNX17-NM_001267059.2eU613-NP_001253988.1aa162SF	0					0			3	34%	6%	420	220	690	350	120	TAGTTCGAGAAAAAGAGGATGGAGCCTTTTCTTTTG								35%	40%	28%
SNX19-NM_001301089.2eU606-NP_001288018.1aa74RC	0					1	6%		2	18%	8%	500	190	700	280	390	ATTGTGGACACCTTGAAGACAGCGTTTCCTCGCTCT							6%		13%	23%
SNX3-NM_001300928.2eU420-NP_001287857.1aa101HY	0					0			3	52%	5%	210	110	710	300	400	TCCTCTGGCACAGAACGAACGTTGTCTTCACATGTT								48%	50%	58%
SNX3-NM_001300929.2eU479-NP_001287858.1aa120HH	1	1%		1%		0			3	50%	4%	210	110	710	300	400	TCCTCTGGCACAGAACGAACGTTGTCTTCACATGTT			1%					49%	47%	54%
SNX3-NM_003795.6eU545-NP_003786.1aa142HH	0					0			3	68%	17%	210	110	710	300	400	TCCTCTGGCACAGAACGAACGTTGTCTTCACATGTT								57%	58%	88%
SNX4-NM_003794.4eU1813-3'UTR+430	0					1	5%		3	16%	5%	270	290	690	290	420	CCACTAATCTGTTATAGGGAGTTGTTTTCTCTTGCC							5%	13%	14%	23%
SNX5-NM_001282454.2eU469-NP_001269383.1aa37SF	0					0			2	2%	1%	710	180	500	500	350	ATCTCGCTGTGTTTAAGAAGACTGTGTCCTCCCATG								1%	2%	
SNX5-NM_001282454.2eU471-NP_001269383.1aa38HY	0					0			3	11%	2%	150	280	710	180	500	CTCGCTGTGTTTAAGAAGACTGTGTCCTCCCATGAA								9%	13%	10%
SOAT1-NM_001252511.2eU1763-3'UTR+21	0					0			3	4%	1%	320	160	580	320	290	CGTGTTTTAGAAGCTTGGACTTTGTTTCCTCCTTGT								3%	5%	4%
SOAT1-NM_001252511.2eU473-NP_001239440.1aa70LL	0					0			3	8%	3%	340	350	640	280	350	AGGAAAGATTTTTATTGCAAGGCGCTCTCTCTTAGA								8%	11%	6%
SOAT1-NM_003101.6eU532-NP_003092.4aa128LL	0					0			2	27%	5%	340	350	640	280	350	AGGAAAGATTTTTATTGCAAGGCGCTCTCTCTTAGA									30%	23%
SOCS6-NM_004232.4eU1098-NP_004223.2aa309PL	0					0			2	6%	2%	410	180	590	330	200	GCCCGAGAGCGGGTCACGATGATGTCCCTCCACTCT									4%	7%
SOCS7-NM_014598.4eU2413-3'UTR+266	0					0			2	10%	5%	400	80	700	290	120	GGCTCCTTGGAAAACTGGAAGAAGTCTCAACACTGT									7%	13%
SOD2-NM_000636.4eU859-3'UTR+116	0					0			3	20%	5%	410	60	590	260	120	CTATTGTAGCATTTCTTGATGTTGCTTAGTCACTTA								14%	25%	21%
SP100-NM_001080391.2eU1133-NP_001073860.1aa346SL	3	2%	0%	2%	0%	0			2	2%	0%	170	150	800	140	290	ACGTTGATGAGCCCTTAGAAGTCTTCATCTCAGCAC	1%	2%	2%					2%		2%
SP100-NM_003113.4eU3042-3'UTR+306	3	5%	3%	5%	3%	0			3	48%	3%	480	230	550	460	370	ATTAGTTGTATATGAGGATGGTAGTTTTTTCACCTT	8%	3%	4%					50%	48%	45%
SP110-NM_001185015.2eU834-NP_001171944.1aa223LL	2	1%	0%	1%	0%	0			3	17%	3%	420	80	500	220	430	AGAAGACTCAGAAGAGATGCCCAGCCTCCTCACTAG	1%		1%					14%	20%	17%
SP1-NM_001251825.2eU6824-3'UTR+4494	0					0			3	4%	1%	340	330	470	530	500	TTGATACCTCTGCTGGGATGTGTGCTTTCCCATATC								4%	4%	5%
SPAG9-NM_001130527.3eU4188-3'UTR+40	0					0			3	5%	1%	430	140	730	370	150	AAACAGGTGGAGATGGGGAAGCCGTCTCTTCTGCAT								4%	5%	5%
SPARC-NM_001309443.2eU3038-3'UTR+2065	4	31%	11%	36%	5%	3	1%	0%	3	2%	1%	360	130	630	380	310	ACATACCTAGATCTCCAGATGTCATTTCCCCTCTCT	41%	31%	36%	16%	1%	2%	2%	1%	2%	3%
SPATS2L-NM_001100422.1eU2296-3'UTR+207	0					0			3	9%	2%	500	130	590	220	260	ATGGCTTTGCTTGATCTGTTGATGCTTTCTCTCATC								8%	8%	11%
SPCS2-NM_014752.3eU278-NP_055567.2aa86TT	0					0			3	2%	1%	340	110	480	570	80	GAATTTTGGTCTAATTGATGGTCGCCTCACCATCTG								1%	2%	3%
SPCS2-NM_014752.3eU589-NP_055567.2aa190TI	0					0			3	8%	2%	340	200	760	260	270	GAACAAAGCAGCAGCGGGAAGCCGAGTTCACAAAGT								9%	5%	9%
SPDYE2-XM_017012223.1eU4800-3'UTR+3107	2	43%	24%	60%		2	45%	46%	2	27%	7%	160	370	500	150	520	TGGGTGTCCTCAGACCACATGTACTTCTCACCAACT	60%			26%	13%		78%	22%		32%
SPG21-NM_001127889.5eU227-NP_001121361.1aa46LL	0					0			3	4%	1%	220	290	720	40	500	CGCGGGCCCCCGAAGTATCAGGTGTCCTCTCATATT								5%	4%	3%
SPIN1-NM_006717.3eU350-NP_006708.2aa33HY	0					0			3	3%	1%	430	260	740	300	220	TCTGCCAACATGATGAAGAAGAGGACATCCCACAAA								2%	4%	3%
SPIN1-NM_006717.3eU3875-3'UTR+2833	4	7%	3%	8%	2%	0			3	78%	1%	280	430	730	350	360	CAAATGTAGTGCCAACAGAAGGTGATTTTCCAGTTG	10%	9%	6%	3%				77%	79%	79%
SPIN1-NM_006717.3eU826-NP_006708.2aa191RR	4	4%	3%	4%	3%	0			2	2%	0%	380	150	570	600	260	CTTAGATGATTACAAAGAAGGCGACCTTCGCATTAT	8%	3%	2%	2%					2%	2%
SPIN4-NM_001012968.3eU560-NP_001012986.2aa16AV	0					0			3	9%	4%	390	90	800	210	330	TGCCTCCGATGGGGGTAGATGGCGTGTCCGCATACC								13%	9%	4%
SPINDOC-NM_138471.3eU1044-NP_612480.1aa271PP	0					0			3	7%	1%	440	170	410	680	240	GGTCCGACACTTCACTGACGGCAGCTTCCCCGCCGG								6%	8%	6%
SPINDOC-XM_005273783.3eU1126-XP_005273840.1aa271PP	0					0			2	9%	3%	440	170	410	680	240	GGTCCGACACTTCACTGACGGCAGCTTCCCCGCCGG								7%	11%	
SPIRE1-NM_001128626.2eU1796-NP_001122098.1aa519Q*	0					0			2	9%	4%	330	110	440	590	180	ACACCCCAGCCAGAGAGACGGCAGCCACCCCAGAGA									6%	12%
SPRED2-NM_001128210.2eU337-NP_001121682.1aa55LL	0					0			2	11%	5%	400	250	760	420	200	CCCCGAAGGCAATGGACGAAGCGGCTTTCTCATCCA								15%		8%
SPRYD7-NM_020456.4eU273-NP_065189.1aa56AA	0					0			2	6%	0%	290	130	610	520	230	AAGAATATGTGGAACAGGAGGTTGTTTAGCCAGCGC									6%	6%
SREBF1-NM_001005291.3eU390-NP_001005291.1aa74PL	0					0			3	24%	4%	330	270	670	240	360	AGCTTATCAACAACCAAGACAGTGACTTCCCTGGCC								24%	19%	28%
SREBF1-NM_001388385.1eU300-NP_001375314.1aa44PL	0					0			3	21%	12%	330	270	670	240	360	AGCTTATCAACAACCAAGACAGTGACTTCCCTGGCC								22%	9%	33%
SREBF2-NM_004599.4eU1781-NP_004590.2aa539LF	0					0			3	17%	1%	390	0	660	270	160	GGGGGCTGGTTTGACTGGATGATGCCTACTCTTCTC								18%	18%	16%
SREK1-NM_001077199.3eU3657-3'UTR+1705	0					0			3	5%	3%	410	140	630	340	120	AAGCCTGATTTGATTATGAAGCTGCTTAATCACTCT								3%	5%	8%
SRP14-NM_001309434.1eU640-3'UTR+103	2	1%	0%	1%	0%	0			3	37%	3%	220	300	780	170	580	TTTCTGGTTCCCCCACAGTAGGTGTTTTCACATAAG		1%	1%					34%	37%	39%
SRP14-NM_003134.6eU579-3'UTR+103	0					0			3	36%	5%	220	300	780	170	580	TTTCTGGTTCCCCCACAGTAGGTGTTTTCACATAAG								36%	40%	31%
SRP19-NM_001204193.2eU198-NP_001191122.1aa36PP	0					0			3	19%	2%	310	240	740	390	300	GAAGACCATCGCAGAGGGAAGGCGAATCCCCATAAG								17%	18%	21%
SRP19-NM_003135.3eU198-NP_003126.1aa36PP	0					0			2	29%	3%	310	240	740	390	300	GAAGACCATCGCAGAGGGAAGGCGAATCCCCATAAG								27%		31%
SRPRA-NM_001177842.2eU2857-3'UTR+933	0					0			2	13%	3%	340	110	650	530	310	ATTAGTGCAATAAGGTGAAGGCTGTCTGCCCAGAGA								11%		15%
SRRM1-NM_001303448.2eU1028-NP_001290377.1aa335PS	0					0			2	2%	1%	560	230	490	510	280	AGAAGAACTCCGCCAAGAAGAATGCCTCCTCCACCA									2%	2%
SRRM1-NM_001303448.2eU586-NP_001290377.1aa187SS	0					0			3	8%	3%	360	490	610	290	250	CAGATCTCCTTCCCCTAGAAGACGATCTTCCCCTGT								5%	8%	11%
SRRM1-NM_001303448.2eU587-NP_001290377.1aa188PS	4	9%	3%	10%	2%	0			3	3%	1%	420	360	490	610	290	AGATCTCCTTCCCCTAGAAGACGATCTTCCCCTGTC	10%	12%	8%	4%				2%	2%	4%
SRRM2-NM_016333.4eU2691-NP_057417.3aa817SS	0					0			3	3%	0%	270	130	630	330	150	ATCTAGAACGCCACCCAGACGCAGTCGCTCCAGTTC								4%	3%	3%
SRSF1-NM_001078166.2eU377-NP_001071634.1aa90R*	0					0			2	2%	0%	330	280	630	320	400	GATGGGTACCGTCTGCGGGTGGAGTTTCCTCGAAGC									2%	2%
SRSF1-NM_001078166.2eU5247-3'UTR+4532	0					0			3	20%	6%	170	220	700	250	580	TTGTCTTGGTCATTAGGGCTGGTGTTTTCACATTTC								14%	19%	27%
SRXN1-NM_080725.3eU840-3'UTR+411	0					0			3	36%	7%	590	340	500	600	310	GTGGTTTAAACAAACAGAAGATTGTTTTTCCACATA								38%	28%	41%
SSH1-NM_018984.4eU3759-3'UTR+522	0					0			3	9%	2%	410	520	700	310	250	GGAATAATGCTCTGTCTGAAAGCGTTTTTCCAAAAA								7%	12%	9%

SSR1-NM_001292008.2eU1060-3'UTR+324	0					0			2	3%	0%	350	160	570	250	170	AGAATACTAAATATAATGCTGTTGTCTCTTCCTTTT								3%	3%	
SSR1-NM_001292008.2eU2133-3'UTR+1397	0					0			2	3%	0%	450	410	590	440	150	AAACGAAATACACACAGAAAAAAGTCTTTCCAAACT								3%		3%
SSR4-NM_001204526.1eU282-NP_001191455.1aa55SF	0					0			3	2%	1%	280	280	640	160	170	CCTACTACACCACTTCTGACGCTGTCATTTCCACTG								2%	3%	2%
SSX2IP-NM_001166293.2eU1304-NP_001159765.1aa375SL	0					0			3	23%	8%	350	150	750	120	270	TGGAAGGTTTTAATGATGAAGATGTAATCTCACGAC								20%	17%	32%
SSX2IP-XM_005270429.2eU1339-XP_005270486.1aa375SL	0					0			3	37%	9%	350	150	750	120	270	TGGAAGGTTTTAATGATGAAGATGTAATCTCACGAC								46%	38%	27%
ST6GALNAC4-NM_175039.4eU1142-NP_778204.1aa284SF	0					0			2	4%	1%	540	250	700	550	290	ACCGCTTCATCACTGAGAAGGCGGTCTTCTCCCGCT								3%		5%
STAG1-NM_005862.3eU3883-NP_005853.2aa1206R*	0					0			3	8%	2%	240	190	670	180	350	CCCATCTTTGAAGATGTGATGATGTCATCCCGAAGC								11%	8%	7%
STAG2-NM_001042749.2eU1686-NP_001036214.1aa406TI	0					0			2	2%	0%	230	260	660	150	510	TTGTTTTACAGAGTAGTGAAGAAGTTCTCACTGCAG								2%	2%	
STAM-NM_001324282.2eU1405-NP_001311211.1aa406LF	0					0			3	7%	2%	420	150	780	140	430	TACAGTCTTCCCCCGGAGCAGCTGTCTTCTCTCAGC								5%	7%	9%
STARD3NL-NM_001363340.2eU242-5'UTR-50	3	5%	1%	5%	1%	0			3	43%	17%	310	240	780	260	350	AGTTTGGATAAGTTTAGCAAGGTGTCTTCTCTTTAG	5%	4%		4%				33%	32%	62%
STARD7-NM_001385622.1eU897-NP_001372551.1aa199RC	0					0			3	19%	1%	420	190	670	360	490	TACAGTGACAATCCCCAAACGGTGTTTCCTCGCTAC								17%	20%	19%
STAT1-NM_001384880.1eU390-NP_001371809.1aa27PP	3	1%	0%	1%	0%	0			3	19%	3%	330	280	490	520	390	GGTTCACCAGCTTTATGATGACAGTTTTCCCATGGA	1%	1%	1%					16%	18%	21%
STAT1-NM_001384881.1eU473-NP_001371810.1aa29PP	0					0			2	17%	12%	330	280	490	520	390	GGTTCACCAGCTTTATGATGACAGTTTTCCCATGGA								25%	9%	
STAT2-NM_001385110.1eU3854-3'UTR+1274	0					0			3	7%	1%	420	320	690	500	410	TTTGTATTTTTAGTAGAGACGGGGTTTCTCCATGTT								6%	8%	6%
STAT2-NM_001385110.1eU4095-3'UTR+1515	1	2%		2%		0			2	2%	0%	300	230	650	360	250	TGTTGTTTACCTGAAAGGAAGTTTTCTATTCGTTGG		2%							2%	2%
STAT3-NM_001369512.1eU2350-NP_001356441.1aa729RC	0					0			2	3%	0%	350	120	530	210	300	AGCAATACCATTGACCTGCCGATGTCCCCCCGCACT								3%	3%	
STAT5B-NM_012448.4eU1172-NP_036580.2aa340Q*	0					0			3	22%	5%	360	80	790	300	260	AGCACGTTCATCATTGAGAAGCAGCCTCCTCAGGTC								16%	22%	26%
STEEP1-NM_001170569.1eU691-NP_001164040.1aa128SL	0					0			2	5%	0%	300	140	630	280	340	AGGAGATTGAGGCTAGGGAAGTTGCTGACTCATATG								5%	5%	
STIL-NM_001048166.1eU228-NP_001041631.1aa25HY	4	42%	11%	46%	8%	0			3	13%	8%	380	290	620	450	180	AGGTTTCCTTCAAGCAGGATGGTACCTTTCCACTTT	51%	38%	50%	28%				4%	17%	17%
STK40-NM_001282546.2eU2691-3'UTR+1158	0					0			3	5%	1%	300	180	510	450	310	CCGAGATTTTGAAGTGGATGCCCGTCTTGCCAGAAA								6%	5%	4%
STMN1-NM_001145454.3eU232-NP_001138926.1aa47LL	4	5%	2%	6%	1%	0			3	4%	0%	250	370	670	460	360	CTTTCCCCTCCAAAGAAGAAGGATCTTTCCCTGGAG	7%	5%	5%	3%				3%	4%	3%
STMN1-NM_005563.4eU232-NP_005554.1aa47LL	3	6%	0%	6%	0%	0			2	6%	0%	250	370	670	460	360	CTTTCCCCTCCAAAGAAGAAGGATCTTTCCCTGGAG	7%	6%	6%						5%	6%
STOM-NM_001270526.2eU1486-3'UTR+829	0					0			2	3%	1%	350	260	690	460	410	TATTGTTTTTTAGTAGAGACGGGGTTTCACCATGTT									4%	3%
STRIP2-NM_020704.3eU2508-NP_065755.1aa823Q*	0					0			3	11%	3%	170	420	660	370	590	GAGCTCTGGCTCGAGAGAGAGGTGTTTTCACAGCCC								9%	9%	15%
STRN-NM_003162.4eU6844-3'UTR+4428	0					0			3	68%	18%	370	210	680	400	140	TTCATTTTATTTACTCAGAAGTTGCCTCTCCATTAC								88%	53%	64%
STT3B-NM_178862.3eU2356-NP_849193.1aa761TI	0					0			3	8%	1%	360	210	700	310	90	TTAAATTCAAACATTTGGAAGAAGCCTTTACATCAG								7%	9%	7%
STT3B-NM_178862.3eU3147-3'UTR+592	0					0			3	4%	1%	370	320	660	320	240	ACTGTTTGCTCTGAGAAGAAGCTGCTGTTTCAAAGA								5%	3%	5%
STX12-NM_177424.3eU849-NP_803173.1aa254LL	0					0			3	6%	3%	440	210	640	320	210	CAGAAAAAATCTCGCAAGAAGATGTGTATCCTGGTG								3%	10%	6%
STX16-NM_001001433.3eU4648-3'UTR+2946	0					0			2	5%	2%	350	170	520	170	490	GTCTCTGATTTAACGGATTCACTGTTTTCTCTGCTA								6%	4%	
STX2-NM_001351049.2eU1516-3'UTR+531	0					0			3	32%	1%	300	330	750	390	450	GTTTTCTTTTTAGTAGAGACGGGGTTTTACCATGCT								32%	31%	32%
STXBP1-NM_001032221.6eU3562-3'UTR+1650	0					0			3	11%	1%	250	280	670	350	340	GGACCTTGTTTGTCGAAGATGTTGGTTTCCCTTTCT								12%	10%	12%
STXBP1-NM_001032221.6eU384-NP_001027392.1aa86SF	0					0			2	3%	1%	430	90	520	370	320	TCATCACTCCATCCGAGAAGTCCGTCCACTCTCTCA								2%	4%	
SUCLA2-NM_003850.3eU375-NP_003841.1aa124SF	0					0			3	8%	3%	570	210	560	420	350	GCCTCAAAGGAGGAGTGAAGATAGTTTTCTCTCCAG								4%	10%	9%
SUDS3-NM_022491.3eU1643-3'UTR+519	0					0			2	4%	0%	310	70	710	140	360	CAGAGGTCCTAGCAGGAGATGATGAATTCTCGTGGC								4%	4%	
SUGP2-NM_001017392.5eU3766-3'UTR+425	4	16%	10%	18%	11%	0			3	64%	3%	270	220	810	210	530	ATGTGGAAACGATCACAGATGGTGTTTTCTCGTTGT	30%	17%	8%	11%				67%	61%	65%
SUGT1-XM_017020365.1eU137-5'UTR-83	0					0			2	24%	11%	340	280	570	460	270	TTTAGGACGGAAGCTCGGTTGGTGTTTCTCCAGAAG									17%	32%
SUGT1-XM_017020365.1eU561-XP_016875854.1aa115RC	0					0			2	44%	23%	410	190	680	400	290	GGACAAAAATTAGATAAGACGGGGTTTCATCGTGTT								27%		60%
SUN1-NM_001130965.3eU1871-NP_001124437.1aa608Q*	0					0			3	13%	5%	210	270	780	280	270	ATCGTGAACAGCGCCTTGAAGCTGTATTCCCAAGAT								13%	19%	8%
SUN2-NM_001199579.2eU2359-NP_001186508.1aa696Q*	0					0			2	5%	1%	470	230	470	240	410	TTCACTTACGATCAGGACGGCGAGCCTATTCAGACG								5%	6%	
SUPV3L1-NM_001301683.2eU553-5'UTR-35	0					0			3	17%	11%	430	250	440	420	190	TGCTGATTATGGACTTGATGCTCGTCTCTTCCACCA								9%	14%	29%
SUPV3L1-NM_001323586.2eU418-5'UTR-78	0					0			2	26%	1%	430	250	440	420	190	TGCTGATTATGGACTTGATGCTCGTCTCTTCCACCA								25%		26%
SUPV3L1-NM_001323587.2eU418-5'UTR-551	0					0			2	37%	26%	430	250	440	420	190	TGCTGATTATGGACTTGATGCTCGTCTCTTCCACCA								19%		56%
SUSD1-NM_001282640.2eU1984-NP_001269569.1aa641SF	0					0			2	14%	10%	360	330	730	330	240	CATTTTCTTGTGATTCTGAAGGCGCTTCCTCCTTCT								7%	21%	
SUSD5-NM_015551.2eU2409-3'UTR+506	0					0			2	33%	6%	260	220	690	380	410	TTTATATTTTTAGTAGAGATGGAGTTTCACCATGTT								38%		29%
SV2A-NM_001328674.2eU455-NP_001315603.1aa59PL	0					0			2	9%	0%	290	140	680	280	200	TTGAGGAGGAGGATGATGATGATGACTTCCCTGCTC								10%	9%	
SVIP-NM_001320340.1eU799-3'UTR+342	0					0			3	16%	5%	300	210	720	140	170	CTCTAAGATTAAAATTCGCAGTTGTCTTTTCATCCT								13%	23%	14%
SYAP1-NM_032796.4eU5708-3'UTR+4536	0					0			3	29%	2%	300	200	610	560	390	TTGTATTTTTTGTAGAGATGGGTGTCTCCCCATGTT								28%	31%	26%
SYDE2-NM_032184.2eU2520-NP_115560.1aa834LL	0					0			2	20%	3%	460	200	560	240	250	AAAGAAAATATAGGACTGATGGTGCCCCTTCTGATA									18%	22%
SYDE2-NM_032184.2eU3370-NP_115560.1aa1117SL	0					0			2	16%	4%	420	110	630	230	200	TAAATGATGTGGATTATGATGATGTCCCTTCAGAAG								19%	13%	
SYNCRIP-NM_001159673.2eU199-5'UTR-39	0					1	1%		3	4%	1%	480	310	590	150	530	TTCTTCAACAGTTTAAAGACAGTGATCTCTCTCATG							1%	3%	4%	5%
SYNCRIP-NM_001159674.2eU359-NP_001153146.1aa86SF	0					1	2%		3	5%	1%	480	310	590	150	530	TTCTTCAACAGTTTAAAGACAGTGATCTCTCTCATG							2%	6%	4%	5%
SYNJ1-NM_001160302.1eU6402-3'UTR+2389	4	28%	14%	35%	7%	2	6%	2%	3	16%	3%	270	150	780	140	330	AAAATACTATATTTATAGAAGTAATCTTCTCTGTTA	30%	31%	43%	8%	4%	8%		13%	17%	17%
SYNRG-NM_001163544.3eU2699-NP_001157016.1aa879PL	0					0			2	5%	1%	350	390	580	340	350	TAACGACCTTTGTAAGTGAAGATGCTCTTCCAGAGA								5%	6%	
SYPL1-NM_001381910.1eU1093-3'UTR+188	0					0			3	2%	1%	500	190	650	100	270	TCCTAGAAAGTTAAGATGTAAATGTATTCTCACATG								3%	2%	2%
SZRD1-NM_001114600.3eU175-NP_001108072.1aa53PP	4	3%	2%	4%	2%	0			3	45%	1%	470	220	430	460	340	GCCCATTGTGATTCAGGACGATAGCCTTCCCGCGGG	5%	5%	2%	1%				45%	46%	46%
SZRD1-NM_001271869.2eU172-NP_001258798.1aa52PP	4	5%	1%	5%	1%	0			3	72%	6%	470	220	430	460	340	GCCCATTGTGATTCAGGACGATAGCCTTCCCGCGGG	6%	4%	6%	4%				66%	79%	72%
SZRD1-NR_073502.2eU125-ncr	4	4%	3%	5%	3%	0			3	47%	4%	470	220	430	460	340	GCCCATTGTGATTCAGGACGATAGCCTTCCCGCGGG	8%	5%	3%	2%				43%	47%	50%
SZRD1-NR_073503.2eU122-ncr	3	4%	2%	4%	2%	0			3	45%	5%	470	220	430	460	340	GCCCATTGTGATTCAGGACGATAGCCTTCCCGCGGG	5%	5%	2%					43%	50%	41%
TAB2-NM_001292034.3eU1991-NP_001278963.1aa533TI	0					0			3	4%	1%	400	0	670	270	210	TGAGTATGGGATCTGATGATGCTGCCTACACACAAG								3%	3%	5%
TAB3-NM_152787.5eU6231-3'UTR+3569	0					0			3	41%	2%	250	130	780	210	320	TTGTAAAGGTTAATTCTGATGACGTTTTCACATTCA								39%	43%	41%
TACC1-NM_001122824.2eU4620-3'UTR+3085	0					0			2	4%	1%	330	250	570	250	600	CTCTGGAAATACCGGGAAGCCCAGTTTTCTCACGTG								5%	3%	
TACC3-NM_006342.3eU296-NP_006333.1aa58Q*	0					0			3	13%	3%	390	300	780	220	180	AACCTGGCCAAAGCTATGAAGGTGACTTTTCAGACA								14%	10%	14%
TAF15-NM_003487.4eU817-NP_003478.1aa244TI	0					0			3	7%	1%	540	150	650	420	380	TTGTGCAAGGACTTGGGGAGGGTGTGTCTACAGATC								9%	6%	7%
TAF15-XM_024450959.1eU761-5'UTR-69	0					0			2	16%	4%	540	150	650	420	380	TTGTGCAAGGACTTGGGGAGGGTGTGTCTACAGATC									19%	13%
TAF1D-NM_024116.4eU983-NP_077021.1aa261SF	0					0			3	18%	5%	400	260	680	340	240	AAGAGGATATTACTGAAGAAGCTGCTTTGTCTAAAA								20%	13%	22%
TAF1D-NR_146090.2eU983-ncr	0					0			3	22%	4%	400	260	680	340	240	AAGAGGATATTACTGAAGAAGCTGCTTTGTCTAAAA								22%	26%	18%
TAF1D-NR_146091.2eU983-ncr	0					0			3	16%	4%	400	260	680	340	240	AAGAGGATATTACTGAAGAAGCTGCTTTGTCTAAAA								13%	20%	14%
TAF2-NM_003184.4eU2148-NP_003175.2aa624PL	0					0			2	3%	1%	300	130	570	500	260	TGGATCTTTCTGCAATGGATGCTGATTCCCCTTTGC								3%	2%	
TAF8-NM_138572.3eU2166-3'UTR+1215	0					0			2	7%	0%	490	250	810	330	350	TCTTACCCCTTCTGGAAGAAGAGGTTTTCTCTGACA									7%	7%
TAF8-NM_138572.3eU3981-3'UTR+3030	0					0			2	10%	2%	350	260	690	460	410	TTTGTATTTTTGGTAGAGACGGGGTTTCACCAGGTT									9%	12%
TAOK1-NM_020791.4eU7655-3'UTR+3893	0					0			3	20%	2%	340	230	750	220	190	TGAGAAGGTGGTCATTAGATGCAGTCTTTTCCTTTT								19%	19%	23%
TAOK2-NM_001252043.2eU4349-3'UTR+164	0					0			2	9%	2%	440	0	670	390	340	CCCTTCGGACCTCTAGACAGGCAGCCTCCTCAGCTG								7%		10%
TAP1-NM_000593.6eU897-NP_000584.3aa277Q*	3	1%	0%	1%	0%	0			3	16%	2%	280	400	750	350	410	GCTGTCCTGCGCCAGGAGACGGAGTTTTTCCAACAG	1%	1%	1%					17%	14%	17%
TARS3-NM_152334.3eU736-NP_689547.2aa217SF	0					0			2	7%	2%	620	270	670	410	80	ACCTGGACCGCCCATTGGAAGGGGACTCTTCTCTAG								6%		9%
TASOR2-NM_001321783.2eU1219-NP_001308712.2aa174SL	0					0			2	9%	2%	250	200	740	80	200	TGAAAGATTTGAAAGTTGAAGATGACATCTCAATGA								11%		8%
TASOR2-NM_001321783.2eU1711-NP_001308712.2aa338PL	0					0			2	9%	6%	440	430	540	600	370	AAATGTTGAAAGCAAAGAAGAGAGTTTTTCCATTGA								13%	5%	
TBC1D15-NM_001146213.3eU505-NP_001139685.2aa161PL	0					0			3	43%	5%	190	250	660	290	340	TATTCTGTCTAAAGGATGACGTCGTTCTCCCTGCTC								40%	39%	49%
TBL1XR1-NM_001321193.3eU5794-3'UTR+3989	0					0			2	6%	1%	450	70	730	270	420	GTAGAAGAGAGAAGAAAGAGGAAGTGTTCACTTTTT								6%		5%
TBL2-NM_001362660.2eU303-NP_001349589.1aa5HY	0					0			2	43%	4%	270	70	810	140	320	CCGCACAGGACTCAACAGATGTTGACTTCTCATCCC								46%		40%
TBX18-NM_001080508.3eU1328-NP_001073977.1aa304TI	0					0			3	11%	7%	290	240	780	140	400	AGGCATTCTCCTTTCCAGAAACTGTCTTCACAACCG								5%	10%	19%
TCAF1-NM_001206938.2eU802-NP_001193867.2aa213SF	0					0			3	8%	3%	300	300	690	80	350	CAGTGTTGGTTAGTTGTGAAGATGACCTCTCCGACG								8%	6%	12%
TCF12-NM_001306219.3eU1754-3'UTR+51	0					0			3	15%	3%	210	390	770	80	470	TATCAGTAGGCTAGATAGAAGGTGACCTCTCCTCAT								14%	18%	14%
TCP1-NM_001008897.2eU1700-NP_001008897.1aa396SF	0					0			3	41%	0%	250	110	640	230	440	AACATGGAAGTTATGAAGATGCTGTTCACTCTGGAG								41%	41%	42%

TDP1-NM_001008744.2eU647-NP_001008744.1aa199SL	0					0			3	26%	11%	430	160	400	490	390	T A T C T C C T T T A T T T G G G A C G C T T G T T T C T T C A G C T C								22%	38%	18%
TEAD1-NM_021961.6eU8049-3'UTR+6147	0					0			3	7%	2%	410	250	690	370	310	T T A C T G T G C T T T G T T T G A A A G T A G T T T T C T C T C T C A								5%	8%	8%
TENM3-NM_001080477.4eU4697-NP_001073946.1aa1516PL	0					0			3	37%	21%	450	260	590	440	60	A C T C T A T G A A C T T C T A T G A A G T T G C G T C T C C A A C T G								25%	25%	62%
TEX10-NM_001161584.2eU1172-NP_001155056.1aa341PL	0					0			2	4%	0%	420	260	580	330	200	T A A T T G A A T G C T G G G T T G A A G C T G T A C C T C C A C A A C									4%	4%
TEX10-NM_001161584.2eU2929-3'UTR+37	0					0			3	40%	9%	490	200	690	250	190	C A T A G T A C C T C C T G T T T G A A G T T G T T T A T T C A C A T C								31%	38%	49%
TFAM-NM_001270782.2eU151-NP_001257711.1aa5R*	4	2%	1%	2%	1%	0			3	20%	1%	380	330	640	420	330	G C C T C A T C C A C C G G A G C G A T G G C G T T T C T C C G A A G C	3%	2%	2%	1%				19%	22%	20%
TFDP1-NM_007111.5eU710-NP_009042.1aa170YY	0					0			3	16%	2%	450	200	620	340	210	T G A C C A G A A A A A C A T A A G A C G G C G C G T C T A C G A T G C								15%	14%	18%
TGFB2-NM_001135599.4eU4581-3'UTR+1886	0					0			3	4%	1%	450	120	800	290	250	T T T G C C A C A T C A T T G C A G A A G A G G T A T C C T C A T G C T								4%	5%	5%
TGFBI-NM_000358.3eU1681-NP_000349.1aa538TI	0					0			3	2%	1%	210	170	810	220	380	C G G A G A C C C T C A A C C G G G A A G G A G T C T A C A C A G T C T								2%	2%	3%
TGFBR2-NM_001024847.2eU813-NP_001020018.1aa144PL	0					0			3	27%	6%	420	240	590	550	200	A T G A C T T T A T T C T G G A A G A T G C T G C T T C T C C A A A G T								34%	23%	24%
TGFBRAP1-NM_001142621.3eU3465-3'UTR+757	0					0			3	13%	6%	190	430	640	340	530	G A G A A C G C T C C A A G A G A G A A G C T G C T C T C C C C T C C T								7%	18%	15%
TGOLN2-NM_001206844.2eU3547-3'UTR+2380	0					0			3	4%	1%	390	190	620	290	230	A T T T T G C T T C T C T C T T G G A T T T T G T T T T T T C A G A T C								5%	3%	3%
TGOLN2-NM_001206844.2eU4792-3'UTR+3625	0					0			3	16%	2%	390	270	720	260	250	G G A A A G C T T A T T T T C T G C A T G G T G T C T T T T C A A A G A								14%	19%	16%
TGOLN2-NM_001206844.2eU5684-3'UTR+4517	2	2%	0%	2%	0%	0			2	1%	0%	510	320	380	330	490	T T T T C C C A G T T A A T A G A T G G T C A C T G T T T C C A C A A T	2%	2%						1%	1%	
THAP10-NM_020147.4eU225-NP_064532.1aa22RC	0					0			2	9%	0%	360	340	570	420	180	A A C A C C A C C A A G T C T G G G A A G T C G C T G T T C C G C T T T									9%	9%
THBS1-NM_003246.4eU3640-NP_003237.2aa1154SF	0					0			3	3%	0%	500	70	530	230	390	C T G G T G G T A G A C T A G G G T T G T T T G T C T T C T C T C A A G								3%	3%	4%
THBS2-NM_001381939.1eU5190-3'UTR+1700	0					0			3	8%	2%	170	350	650	240	440	T T T A C T T T T T T G C G T G T G G A G C T G T A T T C C C G A G A C								8%	10%	6%
THRAP3-NM_001321471.2eU440-NP_001308400.1aa37LL	0					0			2	2%	0%	350	260	680	190	270	T T C G A A G T C T C G G T C C C G A A G C C G A T C T C T C T C T C G									2%	2%
THRB-NM_000461.5eU4766-3'UTR+3067	0					0			2	35%	2%	290	280	550	280	400	A C T T T G C T C A A T T T A G A C G T G C A G T A T T T C C A G A A A								33%	36%	
THSD1-NM_018676.4eU2552-NP_061146.1aa777PS	0					0			3	65%	5%	420	250	820	300	250	C A C A A G A G T G T C T C A A G G A A G C A G T C T T C T C C C A T A								65%	60%	70%
THUMPD1-NM_017736.5eU1499-3'UTR+358	0					0			3	48%	4%	280	360	750	370	340	G A A G T T T T C A T T C T A A G G A A G G A G T T T T T A C A T G C C								51%	49%	43%
TIFA-NM_052864.3eU1115-3'UTR+277	1	15%		15%		0			3	80%	4%	400	200	860	180	210	A A A G T A T C C T T T G A G A T G A A G T C G T G T T C T C A A A A T	15%							80%	76%	84%
TIGAR-NM_020375.3eU2537-3'UTR+1665	3	58%	14%	58%	14%	0			2	9%	4%	340	280	720	280	150	T C C A T T C C C A C C A T A C T G A A G T C A T C T T T C C A C T T T	71%	43%	59%					12%	6%	
TIMMDC1-NM_016589.4eU1229-3'UTR+184	0					0			3	13%	2%	270	120	630	120	420	T T T T A A C T A A G A A T G G G G C T G T T G T A C T C T C A C T T T								12%	12%	15%
TIMP3-NM_000362.5eU1750-3'UTR+817	0					0			3	3%	0%	580	280	420	490	250	T C A G G T G A A A G A C T G A G A T G A A T G T C T T T C C T C T C C								3%	3%	2%
TKT-NM_001064.4eU1441-NP_001055.1aa454TI	0					0			2	2%	0%	320	210	620	260	380	C T G T C T T T T A C C C A A G T G A T G G C G T T G C T A C A G A G A								2%		1%
TMA16-NM_018352.3eU957-3'UTR+233	0					0			3	8%	2%	310	140	680	210	270	G A T A G A T G T G A T C T G C A G A A G T T G T T T A G T C T T C A T								10%	7%	8%
TMBIM1-NM_001321427.2eU2614-3'UTR+1021	1	2%		2%		0			3	42%	9%	410	240	800	260	280	A G G G A G G G T C C T A A G A G G A A G G T G A C T T C T C T C T G T		2%						32%	48%	46%
TMCO1-NM_019026.6eU51-5'UTR-68	0					0			2	5%	2%	250	210	770	330	310	T G G T G G C G G A G T C T A C G G A A G C C G T T T T C G C T T C A C									6%	3%
TMED10-NM_006827.6eU3561-3'UTR+2868	0					0			3	3%	1%	380	430	490	450	220	T T T C T T T C C T A T T T C T G A C T G G T G C C T T T C C C C T T T								2%	4%	4%
TMED5-NM_001167830.2eU281-NP_001161302.1aa37TI	0					0			2	2%	0%	290	300	670	100	270	G C T T C A C A C C T T C C C T C G A T A G C G A C T T C A C C T T T A								2%	2%	
TMED5-NM_001167830.2eU458-NP_001161302.1aa96TI	0					0			3	7%	3%	190	200	640	150	510	T T G A A C A A A G A A A A T C A G A T G G A G T T C A C A C T G T A G								6%	4%	10%
TMED5-NR_030761.2eU458-ncr	0					0			2	41%	21%	150	200	640	150	510	T T G A A C A A A G A A A A T C A G A T G G A G T T C A C A C G T G T A									26%	56%
TMED7-NM_181836.6eU2971-3'UTR+2052	0					0			2	5%	2%	510	100	450	390	260	T A A C T G G T T A C A T T T T G A T G G T T G T C T A T A C T C A A C								7%		4%
TMEM123-NM_052932.3eU1093-3'UTR+324	0					0			3	6%	0%	280	190	670	240	300	C A T A T G A C C A G T A A T T G A A A G A C G T C A T C A C T G A A A								5%	6%	6%
TMEM123-NM_052932.3eU816-3'UTR+47	0					0			3	9%	2%	250	170	630	170	210	A G G A T G G A A T A C A G A T T G A T G C T G C C C T A T C A A T T A								8%	7%	11%
TMEM126B-NM_001193537.3eU737-NP_001180466.1aa171Q*	0					0			3	8%	1%	400	180	690	340	100	A G G G T T T T A A T C C A T T G G A T G A C G C T T T G T C A A A C A								8%	9%	7%
TMEM128-NM_001297551.2eU478-NP_001284480.1aa153Q*	0					0			2	13%	2%	170	130	600	270	330	T C G T T T T T C A C T C C A T T G T T G T T G T T T A C C C A G T T T								15%		12%
TMEM14C-NM_001165258.2eU820-3'UTR+251	1	1%		1%		0			3	58%	4%	380	130	710	180	210	C T T G A T T C T T G T A T A T T G A T G T T G T C T T T T C T T T C T			1%					58%	54%	61%
TMEM170A-NM_001304996.2eU264-NP_001291925.1aa59LF	0					0			2	10%	0%	370	200	660	180	470	G G T G T A T T C C T G T G G G C A C T G G T G T C T T C T C T C T T C								9%		10%
TMEM178B-NM_001195278.2eU7518-3'UTR+6386	0					0			3	16%	2%	400	200	610	330	120	G T C T G T G T T G T G A G C G T G A C G A A G C C T T T C C T G T G A								15%	13%	18%
TMEM181-NM_001376817.1eU1939-3'UTR+269	0					0			3	21%	5%	540	300	540	390	270	T T T T A C A G A G A T T T C A G A T G A G C G T G T T T T C A G T G A								17%	20%	26%
TMEM199-NM_152464.3eU2286-3'UTR+1635	0					0			2	14%	7%	330	310	610	490	430	C A G T T C T C A G C A T A T A G A A G G G A G C T C T C T C A T C T G									19%	9%
TMEM201-NM_001010866.4eU3829-3'UTR+2630	0					0			3	82%	2%	420	180	710	300	170	C T A G A T G T C G T G T T T T G G A T G C T G T G T T T T C A A T A A								80%	81%	84%
TMEM237-NM_001044385.3eU1780-3'UTR+450	0					0			3	44%	6%	510	170	540	390	290	G A A T A A A T T G A A A T A T G A A G T T T G T C T T T T C T G A A A								50%	43%	38%
TMEM245-NM_032012.4eU2686-3'UTR+12	0					0			3	13%	1%	450	100	670	360	230	G A A A T C T T C A G T A G G T T G A T G T G G T T T C C T C T G C A G								14%	12%	13%
TMEM250-NM_001256526.2eU2568-3'UTR+1657	0					0			3	11%	3%	370	110	640	320	410	A A T C T T T T G C A C T A A G T C A T G C T G T T T C C T C A A A G A								8%	14%	13%
TMEM258-NM_014206.4eU97-NP_055021.1aa19PL	0					0			3	2%	1%	320	320	620	280	310	A T A C C A G C C C A G T G A A C C C A G C T G T C T T C C C C C A T C								1%	3%	3%
TMEM30A-NM_001143958.2eU2548-3'UTR+1416	0					0			3	4%	1%	360	100	510	340	610	C A T G T T T G T G T A G A A G A A T G C C T G T T T A C T C A G A G T								4%	5%	3%
TMEM33-NM_018126.3eU6095-3'UTR+5295	0					0			3	8%	2%	450	170	510	430	250	A T A G G A A A A A A T T A T A G A T G G A T G T C T T T G C T G A A A								10%	6%	8%
TMEM39A-NM_018266.3eU576-NP_060736.1aa74LL	0					0			3	9%	3%	390	130	540	400	340	T C C T G A C T T G C C T G T G G A T G G G A G C C T A C T C T T T G A								13%	7%	7%
TMEM40-NM_001284406.2eU699-NP_001271335.1aa178HY	0					0			2	13%	4%	240	510	540	270	540	C T G A A T A T A A A G A A A G A T G A T G A G T T T T T C C A T T T C									15%	10%
TMEM41B-NM_015012.4eU2256-3'UTR+1228	0					0			3	17%	6%	350	260	690	460	410	T T T G T G T T T T T A G T A G A G A C G G G G T T T C A C C A T G T T								15%	12%	23%
TMEM59-NM_001305049.1eU214-5'UTR-210	0					0			2	17%	3%	280	180	540	430	330	C G C C A G C C T A G G A G A A G A A G T T C G T A G T C C C A G A G G								14%	19%	
TMEM65-NM_194291.3eU2273-3'UTR+1006	4	54%	17%	61%	8%	3	12%	6%	3	30%	12%	230	70	830	180	330	T T T T T T T T C C C T C C A A A G A T G T C A T C T T C T C A T C T G	61%	53%	70%	31%	6%	13%	18%	21%	43%	26%
TMEM65-NM_194291.3eU3167-3'UTR+1900	0					0			3	36%	7%	290	90	490	330	320	C C C T A A C T T G T G T A T T G A T G G C A G T C C A C T C T G T T T								42%	36%	29%
TMEM69-NM_016486.4eU305-NP_057570.2aa39Q*	0					0			3	7%	3%	370	210	620	350	130	A C A G A T A T A C T T T C T C T C A A G A T G T C T C T C C A G C A A								9%	4%	7%
TMEM87B-NM_001329914.2eU1909-NP_001316843.1aa529PL	0					0			3	13%	3%	260	310	580	340	290	T T C C C T C T T C A T T C A C A G A T G T A G C T C T T C C A G T G T								16%	11%	12%
TMPO-NM_001032283.3eU4062-3'UTR+2419	0					0			2	3%	0%	280	180	700	180	170	T T C C T G A T T A A A T T G T A G A T G T A G A C T T T A C A A T A T								3%	3%	
TNC-NM_002160.4eU5468-NP_002151.2aa1719SL	4	34%	8%	38%	4%	3	3%	1%	3	13%	1%	280	210	780	330	310	C C A T G G G C T C C C C A A A G G A A G T C A T T T T C T C A G A C A	42%	38%	35%	23%	3%	3%	4%	13%	13%	12%
TNC-XM_005251975.4eU3557-XP_005252032.1aa1082SL	4	36%	12%	42%	2%	0			2	25%	4%	280	210	780	330	310	C C A T G G G C T C C C C A A A G G A A G T C A T T T T C T C A G A C A	44%	42%	41%	18%					27%	22%
TNFRSF10B-NM_003842.5eU3869-3'UTR+2409	0					0			3	7%	3%	240	270	650	160	320	A T A G T G T G T C A C T C G T G G T T G G T G T C C T T T C A T G C T								5%	10%	5%
TNIP2-NM_001161527.2eU899-NP_001154999.1aa156RW	0					0			2	3%	0%	150	420	640	370	350	G A G C C C G A G C T G A T G A G G A A G G A G A T C T C C C G G C T C								3%		4%
TNKS2-NM_025235.4eU2264-NP_079511.1aa667PS	0					0			3	25%	4%	270	250	860	180	350	T G T T T A G C C A G A G T G A A G A A G T T G T C T T C T C C T G A T								22%	26%	29%
TNPO1-NM_001364292.3eU4828-3'UTR+1852	0					0			2	3%	1%	320	320	490	330	370	C T G C T G G T C A G A G A T G A A G C C A C G C C T T T C C A T T T T									3%	4%
TNRC6B-NM_001024843.2eU15190-3'UTR+11815	0					0			3	26%	4%	320	320	600	300	210	T T T T T T T T T C T T A T A C T T G A A G T T G C T T T A C G A T A T								24%	23%	30%
TOLLIP-NM_001318512.2eU1075-3'UTR+266	0					0			3	9%	4%	410	260	440	520	310	G C T C T C C T G T C C C T G G G A C G T G C G T C T C T C C T T C T C								9%	13%	5%
TOMM20-NM_014765.3eU218-NP_055580.1aa32PP	0					0			3	2%	1%	320	120	650	290	290	C T A C T T C G A C C G C A A A A G A C G A A G T G A C C C C A A C T T								1%	3%	2%
TOMM34-NM_006809.5eU223-NP_006800.2aa53LL	0					0			3	26%	2%	590	200	560	390	330	T T C T T C A G A C C C A G A A G A A G A A A G T G T T C T C T A C T C								25%	25%	28%
TOMM70-NM_014820.5eU903-NP_055635.3aa255Q*	4	12%	5%	14%	3%	0			2	2%	0%	220	0	690	250	220	A A G A A T C G T G A A C C T C T G A T G C C A T C T C C A C A G T T T	12%	18%	13%	5%				2%	2%	
TOMM7-NM_019059.5eU299-3'UTR+61	0					0			3	8%	1%	430	80	710	220	270	A A T C A G C G G A C A G C A T G G A A G A T G T G T G C T C T G G C T								6%	8%	8%
TOP2A-NM_001067.4eU4115-NP_001058.2aa1337SL	0					0			3	7%	2%	360	130	760	210	280	C A A T G G A T T T G G A T T C A G A T G A A G A T T T C T C A G A T T								10%	7%	6%
TOP3A-NM_001320759.2eU2320-NP_001307688.1aa631PP	0					0			2	3%	0%	390	240	600	340	300	A A A G T T A A A G T T T A A G C G C G G T A G C C T T C C C C C G A C								2%		3%
TOR1AIP2-NM_001199260.2eU1655-NP_001186189.1aa282Q*	0					0			3	12%	2%	460	230	450	630	330	T T C C T G T G G C A G A G A G G A C G G A A G T T T C T C C A G A A G								13%	14%	10%
TOR1AIP2-NM_001349931.2eU2922-3'UTR+1737	0					0			3	17%	4%	350	260	690	460	410	T T T G T A T T T T T A G T A G A G A T G G G G T T T C A C C A T T G T								21%	14%	15%
TOR1A-NM_000113.3eU995-NP_000104.1aa315SL	0					1	4%		3	4%	3%	480	260	730	380	390	C A T T T T T C C C C A A A G A G G A G A G A G T T T T C T C A G A T A					4%			2%	3%	7%
TOR2A-NM_001085347.3eU1395-3'UTR+405	0					0			2	12%	1%	270	240	770	150	390	G T G T C C A T A T C A A G C C A G G T G G A G C C T T C T C A G T T T									11%	13%
TPCN1-NM_001143819.3eU2354-NP_001137291.1aa730SF	0					0			3	7%	2%	450	170	770	230	290	A C T G G T A C A T C A T C A T G G A A G G C G T C A C C T C T C A G A								5%	7%	8%
TPCN1-NM_001143819.3eU2857-3'UTR+25	0					0			2	10%	6%	430	230	650	450	110	A C C T A G C C C A G C G C C C G A A A G C C G T C T C T T C T A T G C									6%	14%
TPGS2-NM_001271951.2eU934-NP_001258880.1aa233HH	0					0			2	6%	3%	450	190	680	140	400	T A T C A A A A T G A A T A G A A G A A A A T G T G T T C A C T G G A A								5%		8%
TPM3-NM_001043351.2eU1243-3'UTR+397	0					0			3	7%	0%	440	300	780	260	180	T C C C T T T C T C A C T T T T G G A A G G T G A C T T T T C A C C T T								7%	7%	7%
TPM3-NM_001043351.2eU1726-3'UTR+880	0					0			3	10%	1%	290	260	640	240	260	T A C C C T T T T C T G G G T T T G A A G C T G C T G T C T C T A G A A								10%	10%	11%
TPM3-NM_001043351.2eU965-3'UTR+119	3	1%	0%	1%	0%	0			3	27%	2%	390	270	590	550	420	G G C T G A T C T T T A A C T G G A A G G C T G C T T T C T C C T T T C	1%	2%		1%				26%	27%	29%

TPM3-NM_001278188.2eU831-3'UTR+119	0					0			2	25%	6%	390	270	590	550	420	GGCTGATCTTTAACTGGAAGGCTGCTTTCTCCTTTC								29%	21%	
TPP1-NM_000391.4eU3175-3'UTR+1461	0					0			3	11%	2%	400	60	670	410	330	AGTAAGCTTATCTCCCTGAGGCTGTTTCCTCGTCTG								12%	8%	12%
TPT1-NM_001286272.2eU587-NP_001273201.1aa158PL	0					0			3	12%	1%	330	160	650	370	310	TATTGGACTACCGTGAGGATGGTGTGACCCCATATA								13%	11%	13%
TPT1-NM_001286273.2eU513-NP_001273202.1aa124PL	0					0			3	13%	0%	330	160	650	370	310	TATTGGACTACCGTGAGGATGGTGTGACCCCATATA								13%	12%	13%
TPT1-NM_003295.4eU587-NP_003286.1aa158PL	0					0			2	16%	3%	330	160	650	370	310	TATTGGACTACCGTGAGGATGGTGTGACCCCATATA								14%		18%
TRAIP-NM_005879.3eU1268-NP_005870.2aa386PL	0					0			2	10%	1%	250	280	640	330	280	AGCCAGATGAGGAACTGGTTGGTGCCTTCCCTATTT								11%		9%
TRAIP-NM_005879.3eU1483-NP_005870.2aa458LF	0					1	6%		2	8%	3%	530	200	710	190	360	CAGAGGGTGAGGGTGAAGACAGTGCCTTCTCTCTTC							6%	6%	10%	
TRAPPC10-NM_001351709.1eU3086-3'UTR+241	0					0			3	9%	3%	360	60	700	300	160	TCCCCATGCTGGTGTCCGACGCCGCTTACTCAGAGC								9%	12%	7%
TRIB1-NM_001282985.2eU475-NP_001269914.1aa74SL	0					0			2	11%	1%	400	130	690	300	240	ACATAATGAAGGGGGAAGATGATGCTTTGTCAGACA								10%		11%
TRIM14-NM_014788.4eU1531-3'UTR+168	4	2%	1%	2%	1%	0			2	5%	2%	220	390	710	140	420	CACGCCTACAGTGCTTTGAAGGTTTCCTCTCCTAGG	2%	3%	3%	1%				3%	7%	
TRIM16L-NM_001037330.3eU1684-3'UTR+392	0					0			2	7%	0%	350	260	690	460	410	TTTGTATTTTTAGTAGAGATGGGGTTTCACCATGTT								7%	7%	
TRIM23-NM_001656.4eU175-NP_001647.1aa39SF	0					0			3	26%	1%	420	210	750	180	250	TAGAGTGTGGAGTTTGTGAAGATGTCTTTTCTTTGC								27%	25%	25%
TRIM26-NM_001242783.2eU222-5'UTR-36	0					0			2	13%	5%	190	350	750	80	490	GAGCAAGTCCTCCAGGAGAAGAAGTCCTCACCAGTG								9%	17%	
TRIM26-XM_005249374.2eU327-5'UTR-36	0					0			2	16%	5%	190	350	750	80	490	GAGCAAGTCCTCCAGGAGAAGAAGTCCTCACCAGTG								19%		12%
TRIM28-NM_005762.3eU2954-NP_005753.1aa754PS	0					0			3	11%	0%	360	180	730	150	390	CGTGCCCGCCTCCAGGAGAAGTTGTCACCTCCCTAC								11%	11%	11%
TRIM28-XM_024451309.1eU2377-XP_024307077.1aa791PS	0					0			3	23%	11%	360	180	730	150	390	CGTGCCCGCCTCCAGGAGAAGTTGTCACCTCCCTAC								16%	36%	19%
TRIM36-NM_001300752.2eU2420-3'UTR+211	0					0			2	27%	3%	370	470	630	190	420	TCACAAATGATGAAAGTGAAGATGTTCTTTCCAAGT								29%		25%
TRIM44-NM_017583.6eU1115-NP_060053.2aa260Q*	0					0			3	5%	1%	320	200	650	290	280	GTAACTCGGGACCAAATGAAGATGTTTATACAGCAG								6%	5%	4%
TRIM8-NM_001345950.1eU1525-NP_001332879.1aa468Q*	0					0			2	5%	1%	270	250	610	170	350	TCCGGCCACTTTCCCTGGACAGTGCCCTCGCAGGAG									5%	4%
TRIP10-NM_001288962.2eU434-NP_001275891.1aa118Q*	0					0			3	4%	1%	300	380	580	390	170	GAGATGAAACAGGAGAGGAAGATGCACTTCCAAGAA								3%	5%	5%
TRIP6-NM_003302.3eU1650-3'UTR+108	0					0			3	32%	5%	310	110	740	260	390	GTATGACTTTGTCACCAAATGCTGTCTTCTCTTTCT								30%	28%	37%
TRMT10B-NM_001286951.2eU701-NP_001273880.1aa150Q*	0					0			2	32%	17%	330	350	750	160	260	GATGAAAGCATTCAGAAGAAGGTGACATTTCAAAAG									44%	20%
TRNT1-NM_001302946.2eU181-NP_001289875.2aa35PS	0					0			3	16%	6%	350	250	640	170	240	CAGTATCTATTCACAATGAAGTTGCAGTCTCCCGAA								19%	19%	8%
TSC22D2-NM_001303264.2eU1190-NP_001290193.1aa46SF	0					0			3	9%	1%	490	100	800	300	330	CGGACGAGTCACGCACAGAGGACGTCTCCTCCGAGA								9%	8%	9%
TSC22D2-NM_001303264.2eU3317-NP_001290193.1aa755SL	0					0			3	6%	3%	480	40	520	340	270	CGCAACCTCCACAGCAGCCGAATGTCTCCTCAGCAT								3%	7%	8%
TSC2-NM_000548.5eU5392-NP_000539.2aa1761SF	0					0			3	16%	2%	360	230	740	350	100	GCCAGCGGATCTGCGAGGAAGCCGCCTACTCCAACC								14%	15%	18%
TSNAX-NM_005999.3eU1979-3'UTR+964	0					0			3	75%	3%	310	280	710	260	340	AATTTGACAGGTGCCCAGATGTTGCTTTCTCCATTT								78%	73%	75%
TSNAX-NM_005999.3eU972-NP_005990.1aa277SL	0					0			3	11%	4%	420	210	670	220	170	TTCCAAAACATATGTTGGCAGATGTGTTTTCAGTTA								8%	16%	10%
TSPAN17-NM_001006616.3eU281-NP_001006617.2aa50SL	0					0			3	12%	7%	340	360	660	440	500	TCTGGGCCTGGGGTGAGAAGGGCGTTCTCTCGAACA								5%	18%	14%
TSPAN3-NM_001168412.2eU1483-3'UTR+758	0					0			3	7%	2%	290	150	670	80	270	GACTAGCAAAATAGTATGTAGATGTGATCTCAGTTG								4%	8%	7%
TSPAN3-NM_005724.6eU304-NP_005715.1aa50TM	0					0			3	10%	3%	430	80	670	140	280	ACTATGACCACTTCTTTGAAGATGTGTACACGCTCA								10%	7%	13%
TSPYL1-NM_003309.4eU2759-3'UTR+1345	0					0			2	2%	0%	440	270	470	400	390	TGACCTAATTTATGTGGAAGATTGTTATTTCATTAG								3%		2%
TSPYL2-NM_022117.4eU1069-NP_071400.1aa313Q*	0					0			3	10%	2%	270	330	640	300	280	TCCATGGGCTACAAAATGAAGCTGTACTTCCAGACT								8%	12%	11%
TSPYL4-NM_021648.5eU3555-3'UTR+2243	0					0			3	32%	2%	460	130	690	210	410	TTGTAAACAAAATGTAAGTCGATGTTTTCTCTCAAT								32%	29%	33%
TSR2-NM_001346789.2eU2958-3'UTR+2373	0					0			2	11%	1%	350	260	690	460	410	TTTGTATTTTTAGTAGAGACGGGGTTTCACCATGTT									10%	12%
TTBK2-NM_173500.4eU4991-3'UTR+848	0					0			3	19%	1%	360	260	710	170	290	TAATAGCCTTCAAGATAGAAGATGCCCTTTCATCTC								19%	19%	17%
TTC12-NM_001318533.2eU975-NP_001305462.1aa308SS	4	12%	2%	12%	2%	0			3	60%	11%	430	430	790	390	390	TGACAACGAGGTCATAAGAAGGTGTTTTTCCACAGC	15%	11%	11%	11%				50%	71%	60%
TTC26-NM_001144920.3eU2625-3'UTR+1081	0					0			3	12%	10%	350	260	640	460	410	TTTGTATTTTTAGTAGAGACGGGGTTTCACCGTGTT								8%	5%	23%
TTC37-NM_014639.4eU1252-NP_055454.1aa328SL	0					0			3	36%	4%	280	320	720	270	420	AAATGCATAGACCTAAAGAAGCTGTTCTTTCATGCA								31%	38%	39%
TTF1-NM_001205296.2eU758-NP_001192225.1aa177Q*	4	7%	4%	8%	4%	0			3	69%	5%	300	150	810	350	310	GAAGAAGTGATTCTGAAGAAGATGTCTCCCCAGGAG	12%	4%	8%	4%				74%	67%	65%
TTF1-NR_134525.2eU2132-ncr	1	14%		14%		0			3	66%	3%	300	150	810	350	310	GAAGAAGTGATTCTGAAGAAGATGTCTCCCCAGGAG		14%						69%	64%	67%
TTLL12-NM_015140.4eU3203-3'UTR+1203	0					0			3	16%	1%	510	130	550	480	60	GCAAAGCTGCACCTGATGATGCTGCCTCTCCTCTCT								17%	16%	16%
TTL-NM_153712.5eU3091-3'UTR+1768	0					0			3	4%	2%	250	150	720	320	310	ATCTGATCGGATCCTGGGAAGATGTATACCCAGTTA								4%	7%	3%
TUBA1A-NM_001270399.2eU1917-NP_001257328.1aa419SL	0					0			3	22%	6%	470	300	730	300	180	TTGGGGAGGGGATGGAGGAAGGTGAGTTTTCAGAGG								22%	16%	29%
TUBA1B-NM_006082.3eU1351-NP_006073.2aa419SL	0					0			3	19%	1%	470	300	770	300	120	TGGGTGAGGGGATGGAGGAAGGCGAGTTTTCAGAGG								19%	19%	20%
TUBA1C-NM_001303114.1eU1494-NP_001290043.1aa489SL	0					0			3	23%	2%	470	300	770	300	120	TGGGTGAGGGGATGGAGGAAGGCGAGTTTTCAGAGG								21%	25%	23%
TUBA1C-NM_032704.5eU62-5'UTR-40	0					0			3	2%	0%	390	110	540	190	140	GTTAGTGGGAGATCCTTGTTGCCGTCCCTTCGCCTC								3%	2%	2%
TUBB2B-NM_178012.5eU1715-3'UTR+267	0					0			3	44%	9%	250	250	670	240	540	ATAAACGTTTGTGTCGGAATGGTGTCCTCTCTTTCT								55%	36%	43%
TUBGCP4-NM_001286414.3eU1029-NP_001273343.1aa259LL	0					0			2	12%	8%	250	330	730	350	270	ATGCTGGCACCATCTCTGAAGCAGTTTTCCCTACGA									6%	17%
TULP3-NM_001160408.2eU1301-NP_001153880.1aa415TI	0					0			3	15%	2%	430	70	820	140	340	AGTTTGGACGTGTGGCAGATGACGTGTTCACACTGG								12%	16%	16%
TULP3-NM_003324.5eU1301-NP_003315.2aa415TI	0					0			3	14%	5%	430	70	820	140	340	AGTTTGGACGTGTGGCAGATGACGTGTTCACACTGG								18%	16%	9%
TULP4-NM_001007466.3eU7702-3'UTR+4113	0					0			3	31%	13%	260	170	800	250	380	GTACTGATTCTAGGTAAGAAGGAGTCTCCACGGGTG								19%	29%	44%
TUSC1-NM_001004125.3eU892-3'UTR+130	0					0			2	11%	1%	350	230	640	330	280	CCGCGGATACCTTGGACTACGGTGCCTTCCCGCCCC								12%	11%	
TVP23B-NM_001316919.1eU1564-NP_001303848.1aa50Q*	1	2%		2%		0			3	40%	3%	210	380	760	280	400	TGGGTGTTTGAATCTAGAAAGGAGTCCTCTCAAGAG			2%					36%	43%	41%
TVP23B-NM_001316923.2eU306-NP_001303852.1aa28Q*	0					0			3	49%	10%	210	380	760	280	400	TGGGTGTTTGAATCTAGAAAGGAGTCCTCTCAAGAG								50%	38%	58%
TVP23C-NM_001135036.2eU375-NP_001128508.1aa114Q*	1	5%		5%		1	4%		3	42%	9%	210	380	760	280	400	TGGGTGTTTGAATCTAGAAAGGAGTCCTCTCAAGAG	5%						4%	49%	32%	44%
TWNK-NM_001163812.2eU2565-3'UTR+148	0					0			2	13%	1%	270	310	740	240	350	ACCGCTTTGATGGAGATGTAGGTGTCTTCCCGCTTG								12%	13%	
TWSG1-NM_020648.6eU2060-3'UTR+1203	0					0			3	26%	7%	300	260	640	190	440	TTAGAATCTCTATTCAAGATGATGTTCACTCCAAAT								20%	24%	34%
TXNDC12-NM_015913.4eU1273-3'UTR+659	0					0			3	3%	1%	410	240	460	570	460	GACTCGGCTCCAGGTGGAAGCATGCTTTCCCTTGTT								2%	4%	4%
TXNDC12-NM_015913.4eU279-NP_056997.1aa62HY	0					0			3	6%	2%	390	210	630	250	200	GCCAGTGGACTGCCCCTGATGGTGATTATTCATAAA								9%	5%	5%
TXNDC12-NM_015913.4eU751-3'UTR+137	3	3%	1%	3%	1%	0			3	51%	5%	430	290	730	210	290	CATTCCTACCTACACTGGAAGGAGCGCTCTCACTGT	3%	3%	2%					48%	49%	57%
TXNDC5-NM_001145549.4eU2443-3'UTR+1142	0					0			2	2%	0%	530	90	420	420	290	GGCTGTGGCAGCTGTTGATGCCAGTGTCCTCTAACT								1%		2%
TXNIP-NM_006472.6eU85-5'UTR-230	4	3%	1%	3%	1%	0			2	3%	0%	320	300	810	310	470	CCAGCAATTGGGGGAAAGAAGGCTTTTTCTCTGAAT	2%	4%	3%	3%				3%		3%
TXNL4A-NM_001303471.3eU1023-3'UTR+274	0					0			2	29%	2%	320	360	640	390	310	TACCCTTGGAGTAAAACGAAGGTGTTTATCCTGTGA									28%	30%
TXN-NM_001244938.2eU155-NP_001231867.1aa28SL	0					0			3	25%	4%	230	150	770	100	280	CAGGTGATAAACTTGTAGTAGTTGACTTCTCAGCCA								25%	29%	21%
TXN-NM_003329.4eU155-NP_003320.2aa28SL	0					0			3	23%	1%	230	150	770	100	280	CAGGTGATAAACTTGTAGTAGTTGACTTCTCAGCCA								21%	23%	24%
U2SURP-NM_001080415.2eU3483-3'UTR+334	0					0			3	4%	1%	310	360	620	320	350	GACTTAGCAAAAATAATACAGATGTCTTCCCCCCTT								4%	3%	4%
UAP1-NM_001324113.2eU1520-NP_001311042.1aa404SF	0					0			3	3%	2%	560	310	680	180	260	TATATGAAGTATTGCGAGAAGATGAGTTTTCCCCAC								2%	2%	5%
UAP1-NM_001324113.2eU1522-NP_001311042.1aa405PS	0					0			3	3%	2%	170	640	560	310	680	TATGAAGTATTGCGAGAAGATGAGTTTTCCCCACTA								5%	2%	3%
UAP1-NM_001324113.2eU490-NP_001311042.1aa61HY	0					0			3	1%	0%	370	150	700	140	250	AAGGCCATTGAAGGTTTTAACCAGTCTTCTCACCAA								1%	1%	2%
UBA52-NM_001033930.3eU229-NP_001029102.1aa56LL	0					0			3	4%	1%	480	160	480	380	410	CGGCAAACAGCTGGAGGATGGCCGCACTCTCTCAGA								5%	4%	4%
UBA52-NM_001321018.2eU673-5'UTR-88	1	8%		8%		0			3	55%	4%	260	220	690	380	410	TTTGTAGTTTTAGTAGAGACGGAGTTTCACCATGTT	8%							59%	55%	52%
UBA52-NM_003333.5eU207-NP_003324.1aa56LL	0					0			2	6%	1%	480	160	480	380	410	CGGCAAACAGCTGGAGGATGGCCGCACTCTCTCAGA								5%	7%	
UBA6-NM_018227.6eU2579-NP_060697.4aa848SL	0					0			3	5%	3%	400	220	480	410	260	CCAAAAGTGACCTTCAGATGGCAGTGCTTTCATTTG								8%	4%	4%
UBA6-NM_018227.6eU4922-3'UTR+1727	4	10%	4%	12%	4%	0			2	8%	4%	340	430	750	450	370	AAAAGGACTTTTGCAAAGAAGGCTTTTTTCCACGTT	15%	12%	7%	7%				5%	11%	
UBALD1-NM_145253.3eU284-NP_660296.1aa65PS	0					0			2	5%	0%	370	220	600	180	380	AGCCACCATCACCACCAGATGATGTGCACCCCCGCC									4%	5%
UBALD2-NM_182565.4eU351-NP_872371.1aa65PS	0					0			2	14%	3%	480	150	640	80	380	AGCCACCACCACCACCAGATGATGTGCACTCCCAGC								16%	12%	
UBAP1-NM_001171201.1eU1586-NP_001164672.1aa526Q*	4	2%	1%	2%	1%	0			3	42%	2%	390	210	690	360	210	TGTTCAGAAGAAAAGATGATGGAGTTTCTTCAGTTA	3%	2%	2%	3%				40%	44%	42%
UBAP2L-NM_001127320.3eU2374-NP_001120792.1aa754SF	0					0			3	2%	0%	360	280	590	210	310	CTGCACCTCCCCCAGTGGTCAGTGTCTCCTCCAGTC								2%	2%	2%
UBAP2L-NM_001330730.1eU3333-NP_001317659.1aa1056Q*	0					0			2	36%	8%	320	150	760	190	220	ATTCTGACCCCCCATCAGCAGCCGCATTCTCAGATC								42%		30%
UBAP2L-NM_001375617.1eU3224-NP_001362546.1aa1055Q*	0					0			3	17%	6%	320	150	760	190	220	ATTCTGACCCCCCATCAGCAGCCGCATTCTCAGATC								13%	15%	24%
UBB-NM_001281716.2eU377-NP_001268645.1aa38PL	4	28%	7%	31%	1%	3	1%	0%	3	4%	0%	210	450	600	440	420	CCAAGATCCAGGATAAGGAAGGCATTCCCCCCGACC	32%	32%	30%	18%	1%	1%	1%	4%	4%	4%
UBB-NM_001281716.2eU463-NP_001268645.1aa67LL	0					0			3	3%	0%	190	240	730	180	330	TCTGACTACAACATCCAGAAGGAGTCGACCCTGCAC								3%	3%	3%

UBB-NM_001281716.2eU605-NP_001268645.1aa114PL	4	21%	6%	24%	3%	3	1%	0%	3	4%	0%	280	450	620	330	340	C C A A G A T C C A G G A T A A A G A A G G C A T C C C T C C C G A C C	27%	25%	21%	13%	1%	2%	1%	4%	4%	4%
UBB-NM_001281716.2eU606-NP_001268645.1aa114PP	4	1%	1%	2%	0%	0			3	3%	0%	230	280	450	620	330	C A A G A T C C A G G A T A A A G A A G G C A T C C C T C C C G A C C A	2%	2%	1%	1%				3%	2%	3%
UBB-NM_001281716.2eU691-NP_001268645.1aa143LL	0					0			3	1%	0%	190	240	730	180	330	T C T G A C T A C A A C A T C C A G A A G G A G T C G A C C C T G C A C								2%	1%	2%
UBB-NM_001281716.2eU833-NP_001268645.1aa190PL	4	46%	13%	52%	3%	3	4%	0%	3	12%	1%	210	390	690	290	440	C C A A G A T C C A A G A T A A A G A A G G C A T C C C C C C G A C C	55%	53%	49%	27%	4%	4%	4%	11%	12%	12%
UBB-NM_001281717.1eU790-NP_001268646.1aa190PL	4	51%	23%	62%	5%	0			2	16%	1%	210	390	690	290	440	C C A A G A T C C A A G A T A A A G A A G G C A T C C C C C C G A C C	57%	67%	63%	18%					17%	16%
UBB-NM_001281720.2eU230-NP_001268649.1aa67LL	0					0			3	9%	2%	190	240	730	180	330	T C T G A C T A C A A C A T C C A G A A G G A G T C G A C C C T G C A C								8%	11%	7%
UBB-NM_001281720.2eU372-NP_001268649.1aa114PL	4	28%	7%	31%	4%	1	3%		3	6%	1%	280	450	620	330	340	C C A A G A T C C A G G A T A A A G A A G G C A T C C C T C C C G A C C	36%	29%	29%	18%	3%			5%	7%	5%
UBB-NM_018955.4eU213-NP_061828.1aa38PL	4	27%	6%	30%	0%	3	1%	0%	3	3%	0%	210	450	600	440	420	C C A A G A T C C A G G A T A A G G A A G G C A T T C C C C C G A C C	30%	30%	31%	18%	1%	1%	1%	4%	3%	4%
UBB-NM_018955.4eU299-NP_061828.1aa67LL	0					0			3	3%	1%	190	240	730	180	330	T C T G A C T A C A A C A T C C A G A A G G A G T C G A C C C T G C A C								4%	2%	2%
UBC-NM_021009.7eU1092-NP_066289.3aa342PL	4	5%	1%	6%	1%	0			3	1%	0%	320	350	580	410	260	C A A A G A T C C A A G A C A A G G A A G G C A T C C C T C C T G A C C	6%	6%	5%	4%				1%	1%	1%
UBC-NM_021009.7eU1320-NP_066289.3aa418PL	4	5%	1%	5%	0%	3	0%	0%	3	1%	0%	320	350	580	410	260	C A A A G A T C C A A G A C A A G G A A G G C A T C C C T C C T G A C C	6%	5%	5%	3%	0%	1%	0%	1%	1%	1%
UBC-NM_021009.7eU1548-NP_066289.3aa494PL	4	4%	1%	4%	1%	2	0%	0%	3	1%	0%	320	350	580	410	260	C A A A G A T C C A A G A C A A G G A A G G C A T C C C T C C T G A C C	5%	4%	4%	3%	0%	0%		1%	1%	1%
UBC-NM_021009.7eU1776-NP_066289.3aa570PL	4	4%	1%	4%	0%	0			3	1%	0%	320	350	580	410	260	C A A A G A T C C A A G A C A A G G A A G G C A T C C C T C C T G A C C	5%	4%	4%	2%				1%	1%	1%
UBC-NM_021009.7eU180-NP_066289.3aa38PL	4	17%	3%	19%	1%	3	1%	0%	3	3%	0%	320	350	580	410	260	C A A A G A T C C A A G A T A A G G A A G G C A T C C C T C C T G A C C	19%	19%	18%	12%	1%	1%	1%	2%	3%	3%
UBC-NM_021009.7eU2004-NP_066289.3aa646PL	4	4%	1%	4%	0%	0			3	0%	0%	320	350	580	410	260	C A A A G A T C C A A G A T A A G G A A G G C A T C C C T C C T G A T C	4%	4%	4%	2%				0%	0%	1%
UBC-NM_021009.7eU494-NP_066289.3aa143LL	0					2	1%	0%	3	3%	0%	330	280	710	200	330	T C T G A C T A C A A C A T C C A G A A A G A G T C T A C C C T G C A C						1%	1%	3%	3%	3%
UBC-NM_021009.7eU864-NP_066289.3aa266PL	4	5%	1%	5%	0%	0			3	0%	0%	320	410	530	480	320	C A A A G A T C C A A G A C A A G G A A G G C A T T C C T C C T G A C C	5%	5%	5%	3%				0%	0%	0%
UBC-NM_021009.7eU950-NP_066289.3aa295LL	0					0			3	3%	1%	330	280	710	200	330	T C T G A C T A C A A C A T C C A G A A A G A G T C T A C C C T G C A C								3%	2%	3%
UBE2D3-NM_003340.6eU224-5'UTR-263	0					0			3	10%	2%	260	480	610	380	320	T C C G A T C T G T T T A G T A A G A A G G T G C T G T T C C G A G A A								9%	9%	12%
UBE2E3-NM_001278554.2eU1115-3'UTR+46	4	10%	4%	12%	2%	0			3	5%	0%	330	240	910	140	370	T G C A G T G T G A A G G A G C A G A A G G C A T C T T C T C A C T G T	13%	10%	14%	5%				5%	5%	6%
UBE2G2-NM_001202489.2eU2585-3'UTR+2097	0					0			3	17%	7%	290	210	730	130	450	T A T T T G C A G G C A A A T G A G A T G G A G C C C T C T C T G T A A								10%	18%	24%
UBE2H-NM_001202498.2eU1377-3'UTR+799	0					0			2	3%	1%	590	60	610	410	300	A G G A G A A A A G T G A C T G G G A C G A T G C T T C C T C T C A T C								2%		3%
UBE2H-NM_001202498.2eU2554-3'UTR+1976	0					0			3	5%	1%	270	70	750	300	270	A C T T C A C T C T T A C C T A G C A C G C T G T C T T C T C A T T G C								6%	5%	3%
UBE2I-NM_003345.5eU472-NP_003336.1aa108TI	0					0			3	5%	2%	230	240	410	340	480	A G G A G G A C A A G G A C T G G A G G C C A G C C A T C A C A A T C A								4%	7%	6%
UBE2K-NM_001111112.2eU2521-3'UTR+1855	2	6%	2%	6%	2%	0			3	9%	4%	290	280	570	560	270	G A T T G T G T G A A A A T G T G G A T G G T T T T C T C C A G T G T		5%	7%					12%	5%	9%
UBE2L6-NM_004223.5eU119-NP_004214.1aa20PS	0					0			3	10%	6%	340	300	700	360	180	G A G C T G G A G G A T C T T C A G A A G A A G C C T C C C C C A T A C								8%	17%	6%
UBE2Q1-NM_017582.7eU669-NP_060052.3aa187SL	0					0			3	10%	2%	560	140	730	370	70	C A G A G C A G T G C A C A C A G G A A G A C G T G T C T T C A G A A G								7%	11%	11%
UBE2V2-NM_003350.3eU38-NP_003341.1aa5TI	0					0			3	8%	1%	390	80	570	580	340	T C G G G C T G C A G G A G A A G A T G G C G G T C T C C A C A G G A G								8%	8%	7%
UBLCP1-NM_145049.5eU104-5'UTR-15	0					0			2	10%	5%	410	180	620	530	380	A T T T T T T T T T C T G A A G G A A A G C T G C T T C C T C A T A T G								6%	13%	
UBN2-NM_173569.4eU3358-NP_775840.3aa1037PL	0					0			2	16%	3%	390	160	510	610	240	T C A A A T C T C C C T T C T C G A T G G C T G C C T C C C C A A A A C								14%	19%	
UBOX5-NM_001267584.2eU131-5'UTR-12	4	34%	14%	39%	10%	1	8%		2	20%	7%	210	200	720	160	340	G C T G C T T C T G A T G C T T G G A A G A T A T C C T C T C A G C C A	38%	50%	31%	17%	8%				25%	15%
UBOX5-NM_001267584.2eU4210-3'UTR+2530	0					0			2	28%	7%	530	250	800	380	150	A A G A C T T C C C C C A A A A G G A A G T G G T C T T T T C A C T A C								23%		33%
UBQLN2-NM_013444.4eU2281-3'UTR+159	0					0			2	11%	3%	270	370	620	330	300	A A T A T G A T G C A T T T T A A G A T G G A G T C C C T C C C T C C T									9%	13%
UBR3-NM_172070.4eU2354-NP_742067.3aa767TI	0					0			3	12%	4%	260	410	700	240	380	A T G A G A G G T C G A T G T T A G A A G G C G C T C T T A C A T T T C								7%	13%	15%
UBR5-NM_001282873.2eU8250-NP_001269802.1aa2595SL	0					0			3	7%	1%	270	130	780	250	330	C T C A G A G T T C A G A T G C T G A T G C T G T T T T C T C A G C A A								5%	8%	7%
UCHL1-NM_004181.5eU593-NP_004172.2aa182PS	0					0			3	22%	2%	540	310	400	400	260	C T C T A T G A A C T T G A T G G A C G A A T G C C T T T T C C G G T G								21%	21%	24%
UCHL5-NM_001199261.3eU2745-3'UTR+1658	0					0			2	20%	9%	500	340	550	270	480	A A A C A A T T C A A C A G A G A A G G A A A G T C T T T T C A A C A A								13%	26%	
UFD1-NM_001362910.2eU1247-NP_001349839.1aa289SF	0					0			2	6%	2%	310	130	590	220	400	A T G A A G C T G G A G G C A G A T T C G T C G C T T T C T C T G G A G									7%	4%
UHMK1-NM_001184763.1eU7901-3'UTR+6786	4	18%	6%	21%	2%	2	4%	1%	2	2%	0%	280	300	700	330	450	A T G T G T G T C A G T A C C A A A A A G T T A T T T T C T C A A A T C	23%	20%	20%	9%	3%	5%		2%	2%	
UMPS-NM_000373.4eU400-NP_000364.1aa127TI	0					0			2	6%	2%	190	360	660	150	420	A A A C C T G T T T A A T C A T T G A A G A T G T T G T C A C C A G T G								4%		8%
UQCC1-NM_018244.5eU291-NP_060714.3aa94TI	0					0			3	19%	3%	260	270	640	340	430	A G C C T G T T G A G G A G A A G G T T G G T G C T T T C A C A A A G A								21%	20%	16%
UQCRFS1-NM_006003.3eU981-3'UTR+123	0					0			2	3%	2%	370	180	670	180	210	T G A A A T A T G T A A G A A T T G A T G A T G T A T T T G C A A A C A								1%	4%	
URGCP-NM_001204871.2eU545-3'UTR+191	0					0			3	3%	1%	370	80	690	290	280	T G T G C C G T G G T C C T G A G G C A G T T G T C T C C A C A C A A G								4%	4%	2%
USO1-NM_001290049.2eU381-NP_001276978.1aa46LL	0					0			2	8%	2%	380	170	500	170	360	G G A T G A T C G A A G A A A T G C T G T T C G T G C T C T C A A A T C									9%	6%
USP19-NM_001199160.2eU647-NP_001186089.1aa148TI	0					0			3	32%	5%	300	130	720	300	390	T G C A G C T G G A G G A T G T A G A T G C T G C T T T C A C A G A T A								37%	34%	26%
USP28-NM_001301029.2eU4005-3'UTR+1007	0					0			3	19%	6%	400	260	690	460	410	T T T G T A T T T T T A A T A G A G A T G G G G T T T C A C C A T A T T								25%	14%	16%
USP33-NM_001377432.1eU2014-NP_001364361.1aa556SL	0					0			3	8%	2%	250	250	460	360	540	T C A T G G A A T A T G T G A A G A G G T T T G T T G T C T C A T G T G								6%	10%	9%
USP36-NM_001321291.2eU2327-NP_001308220.1aa580SF	0					0			2	4%	1%	250	170	710	160	460	G C T C C T G G G A C A G C A G G G A T G T T G T C C T C T C T A C C T									5%	4%
USP38-NM_001290325.1eU2975-NP_001277254.1aa814TI	0					0			3	47%	3%	300	70	720	140	330	C A G A A A G T T G G T C T G T A G A T G T T G A C T T C A C T G A T C								50%	46%	45%
USP40-NM_001365479.2eU4758-3'UTR+929	1	7%		7%		0			2	13%	8%	440	530	690	350	520	A A G A A A A T G T A A A C T A A G G A A G G G T T T T C C C A C T T A	7%							7%		18%
USP40-NM_001365479.2eU4873-3'UTR+1044	0					0			2	8%	2%	320	390	450	530	180	A T T G A T T T G G G A C A T A G A C G G T T G A C T T T C C C A G A C								9%		6%
USP46-NM_001134223.2eU501-NP_001127695.1aa91PL	0					0			3	19%	6%	210	230	720	260	330	C C A C A C A G A A G A A G A A G G T T G G C G T C A T C C C A C C A A								20%	24%	13%
USP47-NM_001282659.2eU3594-NP_001269588.1aa1127AV	0					0			2	3%	1%	450	180	660	220	250	C A T G C A A G T T T C T G C T A G A T G C T G T G T T T G C T A A A G									2%	4%
USP49-NM_001286554.2eU4216-3'UTR+1913	0					0			2	13%	3%	210	250	750	120	420	G C C A G A G G C T C T G A T G T G A A G C T G T A C T C T C A A G A T								12%	15%	
USP5-NM_001098536.2eU128-NP_001092006.1aa33SF	0					0			3	2%	0%	390	260	710	280	260	A C C G G G T C C A C A A A G A C G A G T G C G C C T T C T C C T T C G								2%	1%	2%
USP9X-NM_001039590.3eU3583-NP_001034679.2aa920LL	0					0			3	11%	1%	310	120	780	180	270	T A C A A T T G G T T C A G T A C G A C G A T G T A T T C T C A A T C G								11%	11%	9%
UTP11-NM_016037.4eU341-NP_057121.2aa91PL	0					0			2	5%	1%	290	150	650	370	210	T T A T T A A G G A G A C T A A G G A A G A A G T A A C C C C A G A A C								5%	6%	
UTP14A-NM_001166221.2eU1841-NP_001159693.1aa598LL	0					0			3	8%	2%	270	210	800	290	320	G C C C A G T G C C A A G A A A A G A C G C C G G T T T C T C A T T A A								10%	8%	6%
UVRAG-NM_001386671.1eU782-NP_001373600.1aa195SF	0					0			2	10%	1%	350	70	700	140	270	A C T G T G T T C G C A A T T C T T A C G A T G T C T T C T C T T T G C									9%	11%
VAMP8-NM_003761.5eU360-NP_003752.2aa100SF	0					0			2	3%	1%	360	200	600	230	320	T C A T T G T G C T C T T T G C C A C T G G T G C C T T C T C T T A A G								2%	4%	
VAPA-NM_003574.6eU345-NP_003565.4aa35TI	0					0			3	3%	0%	320	110	630	190	240	T C A A A G G C C C C T T C A C A G A T G T A G T C A C T A C A A A T C								3%	3%	3%
VAPB-NM_001195677.2eU4505-3'UTR+3974	0					0			3	12%	3%	400	290	900	140	450	A T G T T T G C T G T T G T T G A G A A G G A G T G T T C T C A A A G A								13%	9%	15%
VARS1-NM_006295.3eU757-NP_006286.1aa173R*	0					0			2	3%	1%	280	250	530	330	100	G C G G C T G T C A C A G C C T T G C T G C T G C C T T T C C G A T A C								3%	4%	
VCAN-NM_001164097.2eU5792-NP_001157569.1aa1836Q*	0					0			2	39%	8%	320	150	870	140	350	G C A G T T T C A A C A T T T G C G A A G T T G T C T T C T C A G A C A									33%	45%
VEZT-NM_001352088.2eU1698-NP_001339017.1aa552LL	0					0			3	11%	3%	350	130	700	230	100	T G T A C A G T A G T A C C T T T G A A G C A G C C T A C T C T A C A C								9%	15%	10%
VIPAS39-NM_001193314.2eU2537-3'UTR+548	0					0			3	31%	3%	550	200	660	280	250	A T G C C A C A C A T G G C C T A G A C G T T G T G T T T C C T C A G C								30%	28%	34%
VIPAS39-NM_001193314.2eU2539-3'UTR+550	0					0			3	11%	2%	280	230	550	200	660	G C C A C A C A T G G C C T A G A C G T T G T G T T T C C T C A G C C A								9%	13%	11%
VKORC1L1-NM_001284342.3eU2495-3'UTR+1663	0					0			2	3%	0%	170	330	530	430	490	G T C A A G A T T T C A T C A A G A C T T G T G T T T T C C C A T G A A								3%	3%	
VKORC1L1-NM_001284342.3eU4978-3'UTR+4146	2	7%	1%	6%		0			3	76%	12%	230	270	710	260	340	T G T A G A A G T T A C C T G T G G A A G T T G T G C T C C C A T T A T		6%		7%				63%	85%	82%
VKORC1L1-NM_001284342.3eU882-3'UTR+50	0					0			3	4%	1%	320	380	730	210	370	A A A A A T C C G G T A A A T T A G A A G G G G C C C T C G C T A T T T								3%	3%	6%
VKORC1-NM_001311311.2eU366-NP_001298240.1aa100HY	0					0			3	48%	7%	260	160	740	310	410	C T A C A G C T A T T G T T A G A G A T G G T G T C T C G C C A T G T T								50%	40%	55%
VPS18-NM_020857.3eU3670-3'UTR+429	0					0			3	6%	3%	390	150	800	190	220	C C A G C T C T T C T C T C A G A A G A A G C C T T C T C T T C C T								3%	8%	5%
WAPL-NM_001318328.2eU1644-NP_001305257.1aa398LL	0					0			2	3%	0%	430	150	590	410	330	T C C T G C A G A T T T G G G A G A A G C T G G T C G T C T C A G A A A								3%	3%	
WAPL-NM_001318328.2eU3890-NP_001305257.1aa1147SL	0					0			3	6%	3%	340	350	820	180	200	T G C G G G A A T A T C T G C C A G A A G G A G A C T T T T C A A T A A								7%	3%	9%
WAPL-NM_001318328.2eU524-NP_001305257.1aa25SF	0					0			3	23%	4%	430	280	710	180	110	A T G G C A G T T C A A A A T T C G A T G A A G T C T T T T C C A A C A								27%	18%	24%
WARS2-NM_001378231.1eU125-NP_001365160.1aa39SF	0					0			3	7%	3%	420	360	530	430	350	T C C A G A A A G A C A G C A A G A A G C G A G T A T T T T C C G G C A								9%	7%	4%
WASF2-NM_001201404.3eU1176-3'UTR+105	0					0			3	35%	3%	450	80	740	210	240	T T G A G T A C A G T G A C T C A G A A G A T G A C T C C T C T G A A T								32%	35%	37%
WASF2-NM_006990.5eU1691-NP_008921.1aa489SF	0					0			3	44%	3%	450	80	740	210	240	T T G A G T A C A G T G A C T C A G A A G A T G A C T C C T C T G A A T								40%	46%	46%
WASHC2A-NM_001005751.3eU3951-NP_001005751.1aa1300SF	4	15%	4%	17%	3%	0			2	2%	0%	270	170	740	220	250	T A T T T G A T G A T G A T A T G G A T G A C A T C T T C T C C T C T G	16%	14%	20%	10%					2%	2%
WASHC2C-NM_001169106.2eU3765-NP_001162577.1aa1238SF	4	15%	4%	16%	3%	0			2	3%	0%	270	220	740	220	250	T A T T T G A T G A T G A T A T G G A T G A C A T C T T C T C C A C T G	18%	13%	18%	10%				3%	3%	
WASHC4-NM_001293640.2eU1914-NP_001280569.1aa615PL	0					0			3	8%	1%	170	350	670	280	360	G T T T T T T A T A C T G G C A T C G A G C T G T C T T C C C A A T T T								9%	9%	6%
WASHC4-NM_001293640.2eU5534-3'UTR+1939	0					0			2	6%	1%	420	190	600	330	160	G T T A A T A T G T A C A T T T G G A T G A T G A T T T T T C T T G A T								6%		5%
WASHC5-NM_001330609.2eU1365-NP_001317538.1aa310SL	0					0			3	3%	1%	340	130	680	140	170	A G C G G A T G A C T G A G C T T G C T G A T G T C T T T T C A G G A G								3%	4%	3%

WBP1-NM_012477.4eU698-NP_036609.1aa178SF	3	5%	2%	5%	2%	0	3	43%	12%	470	330	750	360	370	TTGAAGGAACAAATGTGGAAGGTGTTTCCTCCCACC	7%	4%	4%		39%	33%	57%
WBP4-NM_007187.5eU1226-NP_009118.1aa354PL	0					0	3	2%	0%	280	160	690	250	330	CTCTTGGAGTTATGGCAGATGGAGTGGCCCCAGTCT					2%	1%	2%
WDHD1-NM_001008396.3eU1222-NP_001008397.1aa286HH	0					0	3	8%	1%	330	160	600	520	310	GGAGGAAGATGGTCAAGAAGGCAGCATTCACAATCT					8%	10%	7%
WDR11-NM_018117.12eU4125-3'UTR+391	0					0	3	5%	1%	320	340	660	250	420	ATAGTAAATGAAATTTGTGAGATGTTTTCTCAAATA					4%	6%	5%
WDR12-NM_001371664.1eU866-NP_001358593.1aa124SL	0					0	2	5%	1%	400	60	720	490	310	CCCTCTCTGGCCACATGGAGGCAGTTTCCTCAGTTC					4%		6%
WDR19-NM_001317924.2eU3793-NP_001304853.1aa1134HY	0					0	3	8%	1%	340	90	720	290	360	ATGTTGAAAGATGACTGGACGGTGTGTCCACATTGT					8%	8%	7%
WDR26-NM_001115113.3eU1056-NP_001108585.2aa122Q*	0					0	2	5%	1%	280	370	690	460	350	AGCAGCCTCAAGAAGAAGAAGCGGCTCTCCCAGTCA						5%	4%
WDR33-NM_001006622.3eU1003-NP_001006623.1aa274RW	0					0	2	6%	5%	190	450	450	250	700	TGCTGGCACCTTTCTGAAGTAGTGTTGTCCCGGTAT						9%	3%
WDR33-NM_018383.5eU5611-3'UTR+1417	0					0	2	30%	4%	360	220	690	380	410	TGTGTGTTTTTTGTAGAGATGGAGTTTCACCACTTT					27%		33%
WDR3-NM_006784.3eU2647-NP_006775.1aa857FF	0					0	3	5%	0%	430	150	550	300	140	TGATGTTGAACTTATATGCCGGTGCCTCTTCTTCCT					5%	5%	4%
WDR4-NM_001260474.2eU152-NP_001247403.1aa36FF	0					0	2	5%	2%	550	140	390	510	120	CATAGCAAGCAGTGATGATGACAGCCTCTTCATCTA					3%	6%	
WDR55-NM_017706.5eU2055-3'UTR+894	0					0	3	10%	3%	420	320	690	500	410	TTTGTATTTTCAGTAGAGACGGGGTTTCTCCATGCT					7%	9%	13%
WDR5-NM_001384409.1eU1040-NP_001371338.1aa306SL	0					0	3	44%	4%	290	120	790	80	330	AACTACAAGGCCACACAGATGTCGTGATCTCAACAG					39%	47%	46%
WDR75-NM_001303096.2eU2126-NP_001290025.1aa627PP	4	5%	1%	5%	1%	0	3	49%	1%	560	220	620	490	290	CAAACAGCTGCTAGCAGAAGAAAGTCTTCCCACAAC	5%	6%	4%	4%	48%	49%	51%
WDR76-NM_001167941.2eU1142-NP_001161413.1aa343SS	0					0	3	21%	2%	460	340	640	440	220	GGTGTATAGAAATGAAAGAAGTAGCTTTTCCTCCTT					19%	20%	23%
WDR76-NM_001167941.2eU1174-NP_001161413.1aa354TI	0					0	3	7%	2%	380	80	700	260	290	CCTTCGACTTCTTGGCAGAAGATGCCTCCACTTTAA					7%	5%	9%
WDR76-NM_001167941.2eU2237-3'UTR+435	0					0	3	14%	1%	410	190	730	360	410	TTTGTATTTTTAGTAGAGATGGGGTTTCATCATGTT					13%	15%	13%
WDR82-NM_025222.4eU791-NP_079498.2aa168SF	0					0	2	2%	0%	260	170	650	40	310	AAGGGTTAATTTTCGCTGCAGGTGTCAACTCTGAAA					2%	2%	
WEE1-NM_001143976.2eU2364-3'UTR+310	0					0	3	11%	3%	280	510	710	360	410	GCTGTTGCATTGTAATAAAAGGTGTCTTTCCCTGTA					13%	7%	12%
WHAMM-NM_001080435.3eU980-NP_001073904.1aa294SF	0					0	2	13%	9%	250	200	790	160	350	GGACCAGACGGGCTGAAGAAGCTGTCGTCTCTATTC						7%	19%
WHAMMP1-NR_036650.1eU633-ncr	0					0	3	29%	7%	250	260	740	230	410	GGACCAGACGGGCTGAAGAAGCTGTTGTCTCTATTC					33%	22%	33%
WNK1-NM_001184985.2eU1650-NP_001171914.1aa221LL	0					0	3	7%	2%	350	220	600	510	320	CGTGGGAATGTCTAACGATGGCCGCTTTCTCAAGTT					7%	9%	5%
WNK4-NM_001321299.2eU2110-NP_001308228.1aa368R*	0					0	3	8%	3%	220	390	620	260	330	CAGACCCATAACAGCAAGATGGTGACCTTCCGATTT					5%	11%	9%
WRN-NM_000553.6eU374-NP_000544.2aa45PL	0					0	3	15%	7%	190	370	650	180	270	TTCGGAAGAGTGTTTTTGAAGATGACCTCCCCTTCT					23%	10%	13%
WRN-NM_000553.6eU4368-NP_000544.2aa1376PP	0					0	3	9%	2%	210	360	690	310	540	ATGTGATGTCAACAAAAGGAGATGTTTTCCCGGTTC					7%	11%	10%
WSB1-NM_001348350.2eU297-NP_001335279.1aa5PP	0					0	2	7%	1%	390	380	420	410	570	AGACGGTGCCCCATAGATGGCCAGCTTTCCCCCGAG						7%	6%
WTAP-NM_001270531.2eU1117-NP_001257460.1aa304PL	0					0	3	3%	0%	360	280	650	280	140	GGGAGGGCAACACAACCGAAGATGACTTTCCTTCTT					3%	2%	3%
WTAP-NM_001270531.2eU735-NP_001257460.1aa177Q*	0					0	3	4%	1%	270	240	530	590	360	GAGAATCAAGAGCTTGGAAGGCAGCTGTCCCAGGGA					4%	4%	3%
WTAP-NM_152857.3eU920-3'UTR+258	0					0	2	4%	2%	440	220	670	320	180	CGTCGGACTATTCCGAAGAAGTGGCCACCTCCGAAA					3%		6%
WWC1-NM_001161661.2eU3870-3'UTR+2	0					0	3	18%	9%	410	0	730	140	150	TCCCAGCTCTCTCTGCAGATGACGTCTAATCGCCAG					11%	28%	16%
XBP1-NM_001079539.2eU1749-3'UTR+573	0					0	3	13%	6%	310	490	670	420	380	TGCTAGTGTAGCTTCTGAAAGGTGCTTTCTCCATTT					19%	14%	6%
XPO6-NM_001270940.2eU4597-3'UTR+486	0					0	3	5%	1%	230	130	620	170	330	TTGCAAAGACTCACGTTTTTGTTGTTTTCTCATCAT					4%	4%	6%
XPOT-NM_007235.6eU3359-NP_009166.2aa958Q*	0					0	3	3%	1%	270	520	600	370	420	GTTTTTAAAAATTACTTAAAGGTGTTCTTCCAGAGA					3%	2%	5%
XPOT-NM_007235.6eU5730-3'UTR+2354	0					0	3	24%	3%	250	410	690	470	530	ATCAATTCCATATATGGAAAGGTGTTTTCCCTTTCA					22%	27%	23%
XPOT-NM_007235.6eU770-NP_009166.2aa95Q*	0					0	2	3%	0%	260	120	660	350	240	TCATGGCTGCAAGCTCAGATGCTGAATCCCCAACCA						3%	3%
XRCC3-NM_001100118.2eU728-NP_001093588.1aa147PL	1	4%		4%		0	3	19%	10%	440	220	760	370	180	CCGTCTACATCTGCACGGAAGACGCCTTCCCGCACA			4%		13%	13%	30%
XRN1-NM_001282857.2eU9664-3'UTR+4486	0					0	2	8%	1%	290	120	680	120	360	GTTTAATGTGAGTAATAGATGCTGTGCTGTCATTGA					9%		7%
YIPF2-NM_001321439.2eU1904-3'UTR+794	0					0	2	7%	5%	420	270	520	580	340	AGAGGCAGGGGCCGAGGACGGAGGCCTTCCCGACAG					3%		11%
YIPF3-NM_015388.4eU556-NP_056203.2aa146PL	4	28%	11%	33%	3%	0	2	8%	0%	360	280	480	490	260	CCATGATCCCTATCAAGATGGTCAACTTCCCCCAGA	37%	32%	31%	12%	8%	8%	
YIPF4-NM_032312.4eU839-NP_115688.1aa196TI	0					0	2	4%	1%	600	180	650	330	120	TGGTGGTTGGATCATTTGAAGTGGTGTCTACACTTA					3%		5%
YTHDC1-NM_001031732.4eU1883-NP_001026902.1aa516Q*	0					0	3	7%	1%	510	150	550	440	280	AAAATGCGTCACAAGAGAAGAATGCATTCTCAGCCC					6%	8%	8%
YWHAB-NM_003404.5eU133-5'UTR-149	0					0	3	7%	1%	440	190	750	280	250	GTAGTGGGCTTAGGAAGGAAGAGGTCATCTCGCTCG					6%	7%	7%
YWHAB-NM_139323.4eU133-5'UTR-54	0					0	3	4%	1%	440	190	750	280	250	GTAGTGGGCTTAGGAAGGAAGAGGTCATCTCGCTCG					4%	5%	2%
YWHAG-NM_012479.4eU2759-3'UTR+1832	0					0	3	5%	1%	390	280	640	360	180	TACCAGAAGAACTAGTAGAAGAAGCTTTGCCGCTTT					5%	4%	6%
YWHAG-NM_012479.4eU3115-3'UTR+2188	0					0	3	3%	0%	410	170	570	390	130	GAAATGCACTTGCCTATGATACTGTCTCTCCAGTGA					2%	3%	3%
ZBED1-NM_001171135.2eU3140-3'UTR+687	0					0	2	10%	1%	370	470	690	330	120	ACATTCTGGGTGGCGTCGAAGGAGCCTTTCCCGATC						9%	10%
ZBTB1-NM_001123329.2eU264-NP_001116801.1aa23LL	0					0	2	7%	1%	400	300	690	490	390	GCTAAACAACCAAAGAGAATGGGGTTTTCTCTGTGA					8%	6%	
ZBTB40-NM_001083621.2eU1266-NP_001077090.1aa252SL	4	10%	4%	11%	3%	0	3	26%	4%	320	240	910	180	390	ATTTTCTTCTAGAAAATGAAGGTGTCTTCTCAGATG	14%	8%	12%	6%	30%	22%	27%
ZBTB4-NM_001128833.2eU2733-NP_001122305.1aa826SL	0					0	2	9%	5%	400	40	520	420	290	AGGAAGCCCCCCAAGAGATGCAAGTCTCCTCATCCA						5%	12%
ZBTB4-NM_001128833.2eU3469-3'UTR+171	0					0	3	35%	4%	210	200	780	310	430	CAGGCTGCTTTGTGTGAGATGTAGTTTTCCCATCTC					39%	35%	32%
ZC3H12C-NM_033390.2eU6342-3'UTR+3670	0					0	2	21%	11%	350	280	650	350	330	TGTGTTTTGAAAACTCTCAAGCTGTTTTCCCTCTGA						29%	13%
ZC3H12C-NM_033390.2eU7884-3'UTR+5212	0					0	2	14%	1%	510	480	640	480	180	GTTACTGTTATGTATTAGAAGTGGCTTTTCCCCCTA					15%		13%
ZC3HAV1-NM_001363491.2eU1524-NP_001350420.1aa379PS	0					0	3	3%	1%	420	320	720	210	530	CAAGGCGCCAGGAGAAAGACTGTGTTTTCTCCCACG					2%	3%	4%
ZC3HAV1-NM_001363491.2eU4598-3'UTR+1134	4	13%	2%	14%	1%	0	3	10%	3%	230	300	860	270	550	ATTAATATGATTACAGAGAAGGCTTTTTCTCAGGTC	15%	15%	13%	10%	8%	10%	14%
ZCCHC24-NM_153367.4eU3381-3'UTR+2471	0					0	3	60%	12%	210	410	590	430	410	CGATCTAGTTGGGAGATAATGGTGTTTACCCCATAT					73%	50%	56%
ZCCHC2-NM_017742.6eU2613-NP_060212.4aa738PL	0					0	2	11%	1%	260	280	640	220	170	ATGGTGCCCAGAAGTCTGAAGTTGTCGTTCCTGCAC					12%	10%	
ZCCHC4-NM_001318148.2eU340-NP_001305077.1aa102LL	0					0	3	15%	5%	380	110	370	610	220	GAAGCTCATAACCGAAGATGTCAGCCTCCCCTGTCC					21%	10%	14%
ZDHHC3-NM_001135179.2eU1651-3'UTR+485	0					0	3	10%	1%	330	270	410	610	340	ATAAAACCTCACCGAAGATGGATGCTTACCCCTTGA					10%	11%	10%
ZDHHC8-NM_001185024.2eU952-NP_001171953.1aa270LF	0					0	2	10%	4%	410	280	690	370	40	CCGCTCGCGGTGAGTTTGAAGCCGCCTTTCCTTAGG					7%		13%
ZDHHC9-NM_001008222.3eU1412-NP_001008223.1aa312PP	0					0	2	7%	1%	150	270	740	260	210	GCCACTGGAGGAAAGTGGAAGTCGACCTCCCAGTAC					7%	6%	
ZFHX4-NM_024721.5eU1856-NP_078997.4aa479SF	0					0	2	17%	5%	480	230	690	230	240	CGGGAGATGAGGATGAAGAAGATGCGTACTCCAATG					14%	20%	
ZFP62-NM_001172638.2eU3535-3'UTR+775	0					0	2	15%	5%	450	190	780	300	260	TGATGAGGGATAACCTGGAAGTGGTATTCACACATT					19%	11%	
ZFP91-NM_001197051.2eU2145-3'UTR+216	0					0	3	9%	3%	280	280	670	180	260	GCTCCCCTTATAAAATTGATGTTGTCTTTACCAGAA					6%	11%	9%
ZFYVE26-NM_015346.4eU1195-NP_056161.2aa352PL	0					0	3	24%	1%	300	270	810	280	260	CACTAACATTGTTGAAAGAAGAAGACTTCCCAAATC					23%	24%	25%
ZKSCAN1-NM_001287054.3eU4793-3'UTR+2853	0					0	3	17%	4%	550	210	700	180	170	CCTTGAAGCAGCTATTTGAAGATGTGTTTTCTGAGG					20%	13%	19%
ZMAT2-NM_144723.3eU904-3'UTR+290	0					0	3	11%	1%	380	250	680	490	300	GAAAAACCTCCCCTTCTGAGGCTGCTTTCCCAAAAC					11%	13%	11%
ZMIZ2-NM_001300959.2eU2695-NP_001287888.1aa858LL	0					0	2	4%	0%	310	290	500	220	550	CCTACGAACAACAATGACGACCTGCTTTCTCTGTTT					3%		4%
ZMPSTE24-NM_005857.5eU2769-3'UTR+1305	0					0	3	19%	2%	210	150	710	180	410	TGCTCCGCTGACATTTGGGTGATGTCTTCACATGGA					19%	18%	21%
ZMYM3-NM_001171162.1eU4275-NP_001164633.1aa1176R*	0					0	3	6%	0%	210	320	660	190	560	ACACTCCTCCCCAACAATACGGTGTTCTCTCGAGTG					5%	6%	6%
ZMYM3-NM_001171162.1eU6064-3'UTR+1238	0					0	3	51%	21%	150	260	700	190	540	CCCTTGTGTTGTAGTGAGAAGCTGTTGTCACGACTA					39%	40%	75%
ZMYM6-NM_007167.4eU4071-NP_009098.3aa1289SL	0					0	3	46%	12%	380	130	700	270	180	CAACTGTTTATTTATGTGATGCTGCCTTTTCAGCTT					33%	50%	56%
ZNF106-NM_001284306.2eU2768-NP_001271235.1aa813LL	0					0	2	5%	1%	360	140	730	210	260	CCTCTGCCTCCACAGTAGATGGCGAATCCTCTATGC					6%	4%	
ZNF131-NM_001297548.3eU1997-NP_001284477.1aa456SL	0					0	3	23%	7%	320	320	680	270	280	CAGAGCGTCTAGTAACGGAAGAAGTTCTTTCAGTAG					16%	23%	30%
ZNF200-NM_001145446.2eU1350-3'UTR+37	0					0	3	16%	4%	300	200	630	270	300	GAACCCTTTCTGGGTCTGATGGTGCTGCCTCAACCT					12%	18%	19%
ZNF212-NM_012256.4eU1409-NP_036388.2aa427LL	0					0	2	6%	3%	220	200	650	220	500	CATCCCTTGGAGGAAAAGCCGGAGTTCCCTCATCTG					4%		8%
ZNF260-NM_001012756.3eU4750-3'UTR+2664	0					0	3	13%	2%	470	370	690	500	410	TTTGTATTTTTAGTAGAGATGGGGTTTCTCCAAAGG					14%	14%	11%
ZNF274-NM_001278734.2eU1610-NP_001265663.1aa348Q*	0					0	3	5%	2%	270	90	850	140	330	GACACAGTGTTGAAGCAGATGGAGTCTGCTCAGGAA					6%	7%	3%
ZNF367-NM_153695.4eU1696-3'UTR+305	0					0	3	6%	0%	150	260	710	270	390	GTTTAATGCATTCATTGGAAGCAGTTCTCACAGGAA					5%	6%	6%
ZNF367-NM_153695.4eU2794-3'UTR+1403	0					0	3	2%	0%	360	250	620	260	250	TTTAGCTTTTTTGAGTTGAAGACGTTCCTACAGATG					2%	2%	2%
ZNF408-NM_001184751.2eU1660-NP_001171680.1aa526PL	0					0	2	6%	2%	400	240	600	250	280	ACCGCTGCCCACACTGTGCCGATGCCTTCCCCCAGC					5%		7%
ZNF410-NM_001242924.2eU2243-3'UTR+486	0					0	3	20%	2%	420	260	690	300	100	CAACAGAGCTTCACCAGGAAGTTGAGTTTTCAAGAT					21%	22%	18%
ZNF436-NM_001077195.2eU1155-NP_001070663.1aa208HH	0					0	2	7%	1%	190	360	660	230	390	GTGTGGGAAAAGTTTTGGAAGAAGTTCTCACCTGAT					7%	7%	
ZNF445-NM_001369454.1eU5292-3'UTR+2005	0					0	2	8%	1%	470	190	670	190	240	CAGCTGTTCATGGCACTGAAGATGTCCCTTCACTGT					9%		8%

ZNF451-NM_001031623.3eU2201-NP_001026794.1aa712PL	0					0			3	8%	3%	320	340	650	350	200	A A A C T T C A A T T A A A A C C G A A G A T G A T T T C C A G T A A							11%	8%	6%
ZNF451-NM_001257273.2eU1721-NP_001244202.1aa552PL	0					0			2	14%	7%	460	260	560	640	200	C C T A T A G A T A T A G A A A G A A G G G A G A C T C T C C A G A A C							10%	19%	
ZNF461-NM_001297623.3eU134-5'UTR-96	0					0			2	9%	1%	380	310	470	370	340	T C C G A A T C A G A G C T T G G A C C A G A G C C T T C C C T G A G A								10%	8%
ZNF461-NM_001297623.3eU1756-NP_001284552.1aa510HY	0					0			3	31%	2%	320	230	770	290	290	A A A C C T T A T C A A T G C G G G A A G G C G T T T A A T C A T A G A							33%	29%	30%
ZNF462-NM_001347997.2eU4898-NP_001334926.1aa1560Q*	0					0			2	8%	4%	250	260	660	420	560	C C A T G T G A A T T T T G T G G A C G G G C G T T T T C A C A G G G C							10%	5%	
ZNF473-NM_001006656.4eU2238-NP_001006657.1aa684TI	0					3	5%	0%	2	6%	3%	440	300	690	100	420	T T A A A T G T A G T A A G T G T G A C A G A G T C T T C A C C C A G A				6%	5%	5%	4%		8%
ZNF518A-NM_001278524.2eU822-5'UTR-83	0					0			2	41%	20%	520	140	700	250	220	G A A A A A T C C T G T A T A T T G A A G A T G T C T C T A C A C A G T							56%	27%	
ZNF518B-NM_001375816.1eU4914-3'UTR+973	0					0			2	14%	0%	410	70	780	260	270	T C C A A A G A C C A T G G G T G G A T G C T G T C T T C T C T C T T C								14%	14%
ZNF566-NM_001145343.1eU1270-NP_001138815.1aa379Q*	3	11%	1%	11%	1%	0			2	9%	1%	280	240	820	280	370	G A A T G T A A G A T A T G T G G G A A G G C T T A T T C T C A G A G T	12%	11%	10%				8%	10%	
ZNF592-NM_014630.3eU240-5'UTR-93	0					0			3	12%	9%	330	260	580	490	270	A A G G G A G A A G A C A G A A G G A A G A C G C T C C C C C G T A C G							5%	10%	22%
ZNF598-NM_178167.4eU1411-NP_835461.2aa446PL	0					0			2	5%	2%	250	290	680	360	180	C C A G C C C G A A G C T C A A G G A C G A A G A C T T C C C C A G C C								3%	6%
ZNF623-NM_001082480.2eU881-NP_001075949.1aa178HY	0					0			3	18%	2%	420	300	830	290	330	A A A T G T G C G C A G T G T G G G A A G G C G T T T T G T C A C A G T							15%	19%	19%
ZNF638-NM_001014972.3eU3685-NP_001014972.1aa1133PP	0					0			3	4%	2%	350	270	500	430	400	A C C T A A T G A G C T T G A A G A A G A A A G T A C T C C C A G C A T							3%	4%	6%
ZNF639-NM_001375800.1eU440-5'UTR-186	0					0			3	27%	6%	350	260	760	460	470	T T T G T A T T T T C A G T C G A G A C G G G G T T T C C C C A T G T T							20%	31%	29%
ZNF664-NM_001204298.2eU1521-NP_001191227.1aa182Q*	0					0			3	6%	2%	260	280	700	230	360	A A A T G T T A T G A G T G T G G G A A G G C G T T C A G T C A G A G T							4%	7%	8%
ZNF670-NM_001204220.2eU1677-3'UTR+295	0					0			3	19%	10%	320	230	730	220	380	A G T G G T T C A T T G T T A A A G A A G G T G T C T A T A C T T T A G							12%	16%	30%
ZNF689-NM_138447.3eU710-NP_612456.1aa121PL	0					0			3	14%	2%	430	180	610	530	290	G A A A G A A G A A T G G G G A G A A G G A A G T A T T C C C G C C T A							12%	14%	15%
ZNF697-NM_001080470.2eU4946-3'UTR+2695	0					0			3	12%	3%	470	170	350	410	350	T A A A T G C A T T C A G G A G G T T G T T T G T T T C T T C T A T C T							11%	10%	16%
ZNF71-NM_001370214.1eU2657-3'UTR+1057	0					0			2	27%	5%	250	200	530	310	480	G T A G T G C T T G T G T C T T G A T G T G T G T C T T C A C G T G G T								31%	23%
ZNF736-NM_001170905.3eU5870-3'UTR+4396	0					0			2	35%	14%	470	60	650	290	130	A T T T A A C T C T T A C A T T T G A T G C T G T G T C T T C A T T T C							25%		44%
ZNF740-NM_001004304.4eU1000-NP_001004304.1aa193LL	0					0			2	15%	11%	350	260	610	300	560	C A G T C C A A G A C T T C C G A C G G G C A G T T T T C T C T A T A G							7%	22%	
ZNF765-NM_001040185.3eU4054-3'UTR+2364	0					0			2	11%	5%	420	320	690	500	410	T T T G T A T T T T T A G T A G A G A T G G G G T T T C T C C A T G T T							8%		15%
ZNF780B-NM_001005851.3eU5120-3'UTR+2530	0					0			2	30%	16%	300	70	750	80	470	T A A G T A A T C T A T G G A G A G A C G T T G T C A T C T C T G A A A							19%		41%
ZNF865-NM_001195605.2eU540-NP_001182534.1aa143PL	0					0			3	20%	6%	250	350	630	400	260	C C C C T C C T C C C C T C T T T G A C G C T G C T T C C C C A C T C							26%	18%	15%
ZNRF1-NM_032268.5eU1685-3'UTR+353	0					0			2	19%	4%	310	330	570	430	450	A A A A A A G T C T A G T G T C G A C T G G T G T T T T C C C T C G T G							16%		21%
ZRANB2-NM_005455.5eU582-NP_005446.2aa181SL	0					0			3	6%	2%	260	230	650	230	330	A G G A T G A G G A T G A A G A T G A C G C T G A T C T C T C A A A A T							3%	7%	7%
ZSCAN12-NM_001163391.2eU1681-NP_001156863.1aa508Q*	0					0			3	22%	3%	320	280	840	290	390	A A A T G T G A T A A G T G T G G G A A G G C G T T T A C T C A A A G A							22%	20%	25%
ZSCAN12-NR_028077.2eU3726-ncr	0					0			3	20%	10%	350	260	690	460	410	T T T G T A T T T T T A G T A G A G A C G G G G T T T C A C C A T G T T							31%	13%	16%
ZSCAN23-XM_011514406.2eU1848-3'UTR+499	0					0			2	18%	5%	260	130	810	290	400	C A T G A G G T T T A A G G A A A G A A G C T G T C T C C A C A A C A T								15%	21%
ZSWIM7-NM_001042697.2eU308-NP_001036162.1aa66YY	0					0			3	39%	12%	360	450	740	260	240	A A T C T C A T C A C C C A G T G G A A G G C G T G T T T A C C A G G T							26%	48%	44%
ZSWIM8-NM_001242487.2eU4898-NP_001229416.1aa1542PL	0					0			2	8%	2%	210	140	620	320	290	T G C C T C A C A T G C C C C G G C C T G C C G T C T T C C C T G T G C							10%		7%
ZYX-NM_001010972.2eU358-NP_001010972.1aa94PL	0					0			3	2%	1%	310	390	680	330	370	A T G C A G A G G G T G C T C T G G G A G G T G C C T T C C C G C C G C							2%	2%	3%

Supplementary Table S5

PPR65 off-targets detected in the IMR-90 transcriptome. Off-targets appearing in at least two replicates of HA-His$_6$-MBP-PPR65 in the transcriptome of IMR-90. The off-target label reveals the gene ID, the transcript accession with the position of the edited C, the corresponding protein accession with the position of the affected amino acid and the induced amino acid change. Off-targets in non-coding regions are labeled with ‘npc’. Shown are the number of replicates, in which the site was called (Rep.) and the mean C-U conversion rates (%CU) with standard deviations (STABW). Nucleotides from position -30 up- to +5 downstream of the editing sites are listed and nucleotide shading is based on the PPR-RNA binding code with perfect matches in green and moderate matches (transition) in yellow (see Figure 1). Conversion rates of individual replicates (labeled as in Supplementary table S3) are depicted.

>total: 7

Offtarget	Rep.	%CU	STABW	Binding region	% editing in individual replicates		
					I-9	I-10	I-12
TCP11L1-NM_001145541.1eU481-NP_001139013.1aa89-Q*	2	8%	1%	G C C C A T G A A A T T G T A G T A A A T G G A G A C T T T C A G A T T	7%	9%	
NDUFAF3-NM_199069.2eU523-npc	3	6%	2%	T G T C A T G A A G G C C G A G T A A C T G G A G C T G C T C T C A T C	8%	6%	4%
EML4-NM_001145076.3eU1496-NP_001138548.2aa413-LF	2	3%	1%	T G T T T A G C A T T C T T G G G G A A T G G A G A T G T T C T T A C T		3%	2%
MRPL14-NM_001318767.2eU572-npc	2	2%	0%	C T G G T T G C A G G A C T C G T G A A T G G A G C A G T T C T G A G A		2%	2%
SPDYE16-XM_017012887.2eU206-npc	3	13%	5%	G G A G A A A A T C A G A A A G A T G G C T T A G A G A A G C C A G C A	9%	18%	12%
HMGN1-XM_011529543.2eU1210-npc	2	7%	1%	C A G C C T C T G A T G A A G C A G G A G A G A A A G A A G C C A A G T	6%	7%	
EPB41L1-XM_024451852.1eU174-npc	2	6%	3%	C C C G C G C C G C C C C G C C C C G C G G C C T G C C T G C C A G A G		8%	4%

References

1. Lenz,H., Hein,A. and Knoop,V. (2018) Plant organelle RNA editing and its specificity factors: enhancements of analyses and new database features in PREPACT 3.0. *BMC Bioinformatics*, **19**, 255.
2. Barkan,A., Rojas,M., Fujii,S., Yap,A., Chong,Y.S., Bond,C.S. and Small,I. (2012) A combinatorial amino acid code for RNA recognition by pentatricopeptide repeat proteins. *PLoS Genet.*, **8**, e1002910.
3. Oldenkott,B., Yang,Y., Lesch,E., Knoop,V. and Schallenberg-Rüdinger,M. (2019) Plant-type pentatricopeptide repeat proteins with a DYW domain drive C-to-U RNA editing in *Escherichia coli*. *Commun. Biol.*, **2**, 85.
